# Supplementary material for: Direct conversion of various phosphate sources to a versatile P-X reagent [TBA][PO2X2] via redox-neutral halogenation
Source: Nat Commun. 2025 Feb 26;16:2004. doi: 10.1038/s41467-025-57255-1 (PMC11865473; doi:10.1038/s41467-025-57255-1)
Supplement: Supplementary file 1 — Supplementary Information [file 41467_2025_57255_MOESM1_ESM.pdf]

Supplementary Information

Direct conversion of various phosphate sources to a versatile P-X reagent [TBA][PO_2X_2] via redox-neutral halogenation

Yaling Tian[1, §], Dong-ping Chen[1, §], Yao Chai[1], Ming Li[1], Xi-Cun Wang[1], Zhengyin Du[1], Xiaofeng Wu[1,2], and Zheng-Jun Quan[1*]

[1] Gansu International Scientific and Technological Cooperation Base of Water-Retention Chemical Functional Materials, College of Chemistry and Chemical Engineering, Northwest Normal University, Lanzhou, Gansu 730070, P.R. China

[2] Materials Innovation Factory, and Department of Chemistry, University of Liverpool, Liverpool, UK L69 7ZD

[§]These authors contributed equally: Yaling Tian, Dong-ping Chen

Corresponding Emails: quanzhengjun@hotmail.com

Content

1 General Information 2

1.1 General Remarks 2

2 Experimental procedures 3

2.1 Preparation of P(V)-Cl reagents. 3

2.2 Preparation of P(V)-F reagents. 5

2.3 Scale-up experiments of raw materials [TBA][PO_2Cl_2] and [TBA][PO_2F_2]. 9

2.4 Stability tests for 1a [TBA] [PO_2Cl_2] and 1c [TBA] [PO_2F_2]. 10

3 Application of the P-X reagents 12

4 Research on reaction mechanism 17

4.1 Experimental verifications 17

4.2 Synthesis of 1a using Vilsmeier reagent 22

4.3 Recovery experiment of cyanuric acid 23

5 Control experiments of "$Na_2S_2O_3 \cdot 5H_2O$" for the synthesis of I-1 24

6 Density Functional Theory (DFT) Calculations 26

6.1. Computational details 26

6.2 Free energy profiles for the reaction pathways 27

7 X-ray Crystallographic Data 29

8 Characterization Data of the Products 34

8.1 Copies of NMR spectra about P(V)-X reagents 34

8.2 Copies of NMR spectra about corresponding product 35

9 Copies of NMR Spectra 63

10 References 190

1 General Information

1.1 General Remarks

All the chemical reagents were purchased from commercial sources and used as received unless otherwise indicated. [TBA][H_2PO_4] was used after vacuum-drying at 110 °C for 12 h. Diatomaceous earth, 4 Å molecular sieve, and basic alumina were all dried by dynamic vacuum heating to 200 °C for at least 48 h before usage.

THF were dried over Na and stored with molecular sieves (4 Å). CH_3CN and DCM were distilled in the present of CaH_2 and stored with molecular sieves (4 Å). $CDCl_3$ was stored with molecular sieves (4 Å). All solvents used were anhydrous and oxygen-free, obtained by bubble degassing with argon. To ensure proper usage, it is recommended that all glassware be oven-dried for a minimum of 6 hours at temperatures exceeding 120 °C. All the other materials unless otherwise mentioned were used as received.

^{1}H NMR, ^{31}P NMR, ^{19}F NMR and ^{13}C NMR data analyses were performed with Varian Mercury-400 Plus, Agilent Technologies DD2 (600 MHz) instrument respectively. $CDCl_3$, DMSO-d_6, and Acetone-d_6 was employed as solvents and tetramethylsilane (TMS) as the internal standard. Chemical shifts were reported in units (ppm) by specifying TMS resonance in the ^{1}H NMR spectrum as 0.00 ppm. The data of ^{1}H NMR was reported as follows: chemical shift, multiplicity (s = singlet, d = doublet, t = triplet, m = multiplet, and br = broad), coupling constant (*J* values) in Hz and integration. The chemical shift for ^{13}C NMR spectra was recorded in ppm from TMS using the central peak of $CDCl_3$ (77.0 ppm), DMSO-d_6 (39.5 ppm), Acetone-d_6 (29.8 ppm) as the internal standard. High-Resolution Mass Spectrometry (HRMS) was obtained using a Q-Exactive instrument equipped with an ESI source from the thermofisher, and the type of mass analyzer used for HRMS measurements was a quadrupole mass filter. X-ray single crystal diffraction data were collected on a Bruker APEX-II CCD diffractometer equipped with a liquid nitrogen cryogenic device. Flash chromatography was performed using 200-300 mesh silica gel with the indicated solvent system according to standard techniques. Melting points were measured with an XT-4 apparatus. Column chromatography was generally performed on silica gel (200–300 mesh) and TLC analyses were conducted on silica gel GF254 plates.

The inclusion of the tetrabutylammonium salt (TBA) group in the structure of the compound has the potential to affect the mass spectrometry data. Consequently, we did not utilize high-resolution mass spectrometry in the detection process. Instead, we determined the main compound structure by means of single crystal structure analysis.

2 Experimental procedures

2.1 Preparation of P(V)-Cl reagents.

Table S1 Conditional screening for synthetic [TBA][PO_2Cl_2] 1a[a].

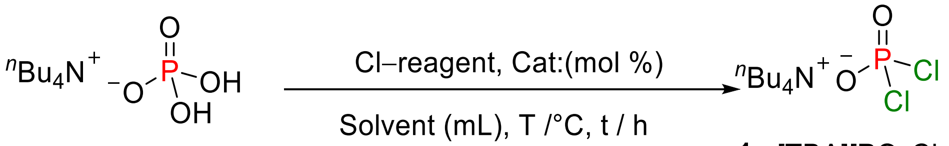

1a [TBA][PO_2Cl_2]

Entry	"P" source	"Hal" agent	Solvent	Cat.	T/℃	Time/ h	Yield%[b]
1	[TBA][H_2PO_4]	TCT (76 mol%)	CH_3CN	\	80	24	27
2	[TBA][H_2PO_4]	TCT (76 mol%)	CH_3CN	Fpyr(5 mol%)	80	24/12	94/93
3	[TBA][H_2PO_4]	TCT (76 mol%)	CH_3CN	Fpyr(10 mol%)	80	12	98
4	[TBA][H_2PO_4]	TCT (76 mol%)	CH_3CN	Fpyr(15 mol%)	80	12	95
5	[TBA][H_2PO_4]	TCT (76 mol%)	CH_3CN	DMF(5 mol%)	80	12	67
6	[TBA][H_2PO_4]	TCT (76 mol%)	CH_3CN	DMF(10 mol%)	80	12	80
7	[TBA][H_2PO_4]	TCT (76 mol%)	CH_3CN	DMF(20 mol%)	80	12	83
8	[TBA][H_2PO_4]	TCT (76 mol%)	CH_3CN	DMF(30 mol%)	80	12	85
9[c]	[TBA][H_2PO_4]	TCT (76 mol%)	CH_3CN	DMF (40 mol%)	80	12	92
10	[TBA][H_2PO_4]	TCT (76 mol%)	CH_3CN	DMF (50 mol%)	80	12	95
11	[TBA][H_2PO_4]	TCT (76 mol%)	CH_3CN	Fpyr(5 mol%)	60	12	95
13	[TBA][H_2PO_4]	TCT (76 mol%)	CH_3CN	Fpyr(5 mol%)	40	12	93
14	[TBA][H_2PO_4]	TCT (76 mol%)	CH_3CN	Fpyr(5 mol%)	rt	12	96
15	[TBA][H_2PO_4]	TCT (76 mol%)	CH_3CN	DMF (40 mol%)	60	12	90
16	[TBA][H_2PO_4]	TCT (76 mol%)	CH_3CN	DMF(40 mol%)	40	12	85
17	[TBA][H_2PO_4]	TCT (76 mol%)	CH_3CN	DMF(40 mol%)	rt	12	77
18	[TBA][H_2PO_4]	TCT (76 mol%)	CH_3CN	Fpyr(5 mol%)	rt	3	76
19	[TBA][H_2PO_4]	TCT (76mol%)	CH_3CN	Fpyr(5 mol%)	rt	6	87
20[d]	[TBA][H_2PO_4]	TCCA (76 mol%)	CH_3CN	Fpyr(5 mol%)	80	12	25
21[e]	[TBA][H_2PO_4]	NCS (2.5 eq.)	CH_3CN	Fpyr(5 mol%)	80	12	15
22[f]	[TBA][H_2PO_4]	BzCl (2.5 eq.)	CH_3CN	Fpyr(5 mol%)	80	12	56
23	[TBA][H_2PO_4]	PCl_5(0.5 eq.)	CH_3CN	Fpyr(5 mol%)	80	12	32
24[g]	[TBA][H_2PO_4]	BTC (76 mol%)	CH_3CN	Fpyr(5 mol%)	80	12	22
25	[TBA][H_2PO_4]	TCT (76 mol%)	DCE	Fpyr(5 mol%)	80	12	trace
26	[TBA][H_2PO_4]	TCT (76 mol%)	CH_2Cl_2	Fpyr(5 mol%)	rt	12	23

[a] Standard conditions: [TBA][H_2PO_4] (3 mmol), TCT (76 mol%), FPyr (5 mol%), CH_3CN (2 mL), rt, 12 h. [b] Isolated yield. [c] [TBA][H_2PO_4] (3 mmol), TCT (76 mol%), DMF (40 mol%), CH_3CN (2 mL), rt ,12 h. [d] TCCA = Trichloroisocyanuric acid. [e] NCS = N-Chlorosuccinimide. [f] BzCl = Benzoyl chloride. [g] BTC = Bis(trichloromethyl)carbonate.

Table S2 [TBA][PO_2Cl_2] was prepared by using various P-source [a].

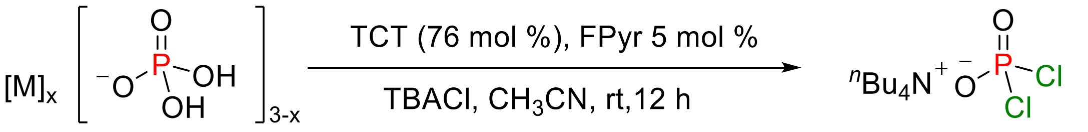

1a [TBA][PO_2Cl_2]

M = $^{n}Bu_4N^+$, K^+, Ca^{2+}, Na^+...

x = 1, 2

Entry	"P" source	"Hal" agent	TBA salt	(Cat.)	Yield%[b]
1	[TBA][H_2PO_4]	TCT	\	DMF	93
2	K_3PO_4	TCT	TBACl	DMF	83
3	$CaHPO_4$	TCT	TBACl	DMF	69
4[c]	$Na_3P_3O_9$	TCT	TBACl	DMF	76
5[d]	P_2O_5	TCT	TBACl	DMF	98
6	$CN_3H_6.H_2PO_4$	TCT	\	DMF	86
7	$NH_4H_2PO_4$	TCT	TBACl	DMF	82
8[e]	PPi	TCT	TBACl	DMF	78
9[e]	$Na_4O_7P_2$	TCT	TBACl	DMF	N.D.
10[e]	$Ca_3O_8P_2$	TCT	TBACl	DMF	N.D.
11	$Ca(H_2PO_4)_2$	TCT	TBACl	DMF	N.R.
12	$Ca(H_2PO_4)_2{\cdot}H_2O$	TCT	TBACl	DMF	N.R.
13	KH_2PO_4	TCT	TBACl	DMF	83
14	$Na_3PO_4{\cdot}12H_2O$	TCT	TBACl	DMF	N.D.
15	$Na_2HPO_4{\cdot}12H_2O$	TCT	TBACl	DMF	N.D.
16	NaH_2PO_4	TCT	TBACl	DMF	89

[a] Standard conditions: other "P" source (3 mmol), TCT (76 mol%), TBACl (1.0 eq.), FPyr (5 mol%), CH_3CN (2 mL), rt, 12 h. [b] Isolated yield. [c] $Na_3P_3O_9$ (3 mmol), TCT (2.0 eq.), TBACl(3.0 eq.), Fpyr (5 mol%), CH_3CN (2 mL), rt, 12 h. [d] P_2O_5 (3 mmol), TCT (3.0 eq.), TBACl (4.0 eq.), Fpyr (40 mol%), CH_3CN(2 mL), rt, 12 h. [e] $H_4P_2O_7$, $Ca_3P_2O_8$ and $Na_4P_2O_7$ (3 mmol), TCT (1.5 eq.), TBACl (2.0 eq.), DMF (40 mol%), CH_3CN(2 mL), rt, 12 h. $H_4P_2O_7$ = Pyrophosphoric acid, $Na_4P_2O_7$ = Sodium pyrophosphate.

Table S3 General procedure for the preparation of [TBA][PO_2Cl_2].

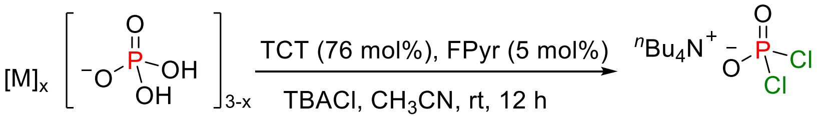

M = $^{n}Bu_4N^+$, K^+, Ca^{2+}, Na^+...

x = 1, 2

1a [TBA][PO_2Cl_2]

Method 1: [TBA][PO_2Cl_2] was prepared using [TBA][H_2PO_4] as P-source.

A solution of the [TBA][H_2PO_4] (3 mmol) and TCT (2.28 mmol, 76 mol %) and FPyr (0.15 mmol, 5 mol%) in CH_3CN (2 mL) was stirred at room temperature for 12 h. The precipitate was removed by filtering. The obtained filtrate was concentrated in vacuo and the resulting crude product was purified by recrystallization with DCM and petroleum ether to provide product [TBA][PO_2Cl_2] **1a** (96%) as a white solid.

Compound 1a, physical state: white solid; **TLC** R_f = 0.35 (DCM: MeOH = 20:1); **^{1}H NMR** (400 MHz, Chloroform-*d*) δ = 3.23 – 3.17 (m, 8H), 1.64 – 1.54 (m, 8H), 1.42 – 1.33 (m, 8H), 0.92 (t, *J* = 7.2, 12H). **^{31}P NMR** (162 MHz, Chloroform-*d*) δ = -7.23. **^{13}C NMR** (151 MHz, Chloroform-*d*) δ = 58.4, 23.6, 19.4, 13.3.

Method 2: [TBA][PO_2Cl_2] was prepared using different P-source.

[M][H_2PO_4] salts (M = K^+, Na^+, NH_4^+) (3 mmol) and TCT (76 mol %) and FPyr (5 mol%) in CH_3CN (2 mL) were stirred at room temperature for 12 hours. The precipitate was removed by filtering. The obtained filtrate was concentrated in vacuo and the resulting crude product was purified by recrystallization with DCM and petroleum ether to provide product [TBA][PO_2Cl_2] **1a** as a white solid (Table S2).

Method 3: [CN_3H_6][PO_2Cl_2] was prepared using [CN_3H_6][H_2PO_4] as P-source.

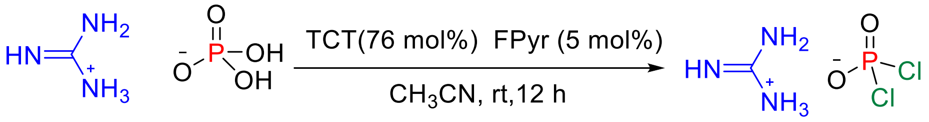

1b

A solution of the guanidine phosphate (3 mmol) TCT (2.28 mmol, 76 mol %), and FPyr (0.15mmol, 5 mol%) in CH_3CN (2 mL) was stirred at room temperature for 12 h. The precipitate was removed by filtering. The obtained filtrate was concentrated in vacuo and the resulting crude product was purified by recrystallization with DCM and petroleum ether to provide product **1d** as a white solid.

Compound 1d, physical state: white solid; **TLC** R_f = 0.28 (DCM:MeOH = 20:1); **^{31}P NMR** (162 MHz, Chloroform-*d*) δ = -4.45. **^{13}C NMR** (151 MHz, DMSO-d_6) δ = 158.7.

2.2 Preparation of P(V)-F reagents.

Table S4 [TBA][PO_2F_2] was prepared using [TBA][H_2PO_4] as P-source[1-8].

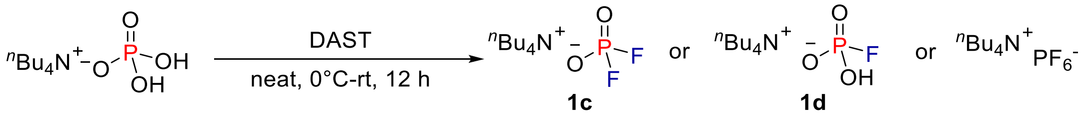

Initial attempt:

[TBA][H_2PO_4] (3 mmol) was put into a round-bottom flask at 0 °C, followed by DAST (3 mmol, 1.0 eq.) slowly added to it and stirred vigorously, after the end of the addition, the temperature was raised to room temperature and the reaction was continued for 12 h. Finally, the reaction system was dissolved with DCM, then the precipitate was removed by filtering the

obtained solution was concentrated, and the mixed spectrum was obtained by dissolving it with $CDCl_3$ (Figure S1).

Table S5 Conditional screening for synthetic [TBA][PO_2F_2][a].

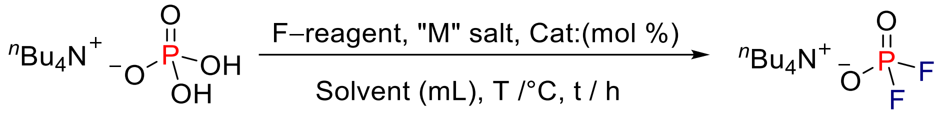

Entry	"P" Source	"F" reagent	Additive	Cat.	T/ ℃	Yield%
1	[TBA][H_2PO_4]	DAST	\	\	0-rt	--[b]
2	[TBA][H_2PO_4]	Cyanuric fluoride	\	DMF	rt	83
3	[TBA][H_2PO_4]	Cyanuric fluoride	\	FPyr	rt	88
4	[TBA][H_2PO_4]	Xtalfluor-E	\	DMF	rt	67
5	[TBA][H_2PO_4]	Fluolead	\	DMF	rt	43
6	[TBA][H_2PO_4]	TBAF	\	DMF	80	N.D
7	[TBA][H_2PO_4]	NaF (2 eq.)	\	DMF	80	N.D
8	[TBA][H_2PO_4]	KF (2 eq.)	\	\	80	N.R
9	[TBA][H_2PO_4]	KF (2 eq.)	18-C-6 (2 eq.)	\	80	N.R
10	[TBA][H_2PO_4]	KF (2 eq.)	FPyr(10 mol%)	\	80	N.R
11	$CaHPO_4$	DAST	TBAF·$3H_2O$	\	0-rt	89

Reaction conditions for **1c**: [a] Direct deoxyfluorination: [TBA][H_2PO_4] (3 mmol), cyanuric fluoride (76 mol%), FPyr (5 mol%), CH_3CN (2 mL), rt, 12 h. [b] See Table S4 and Figure S1.

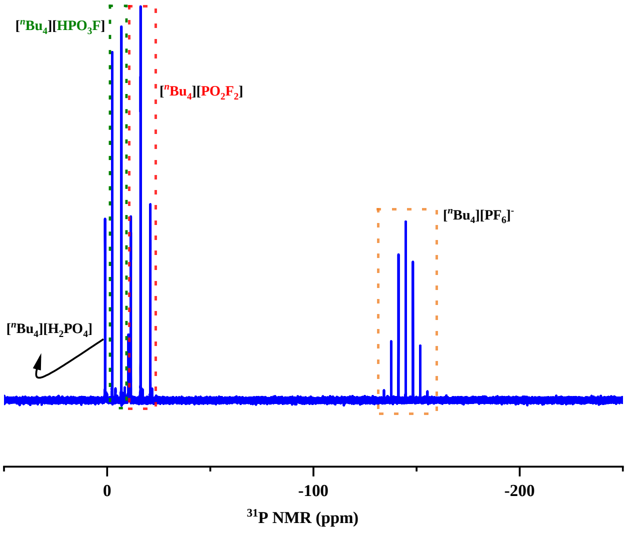

Figure S1 ^{31}P NMR spectrum in $CDCl_3$ of direct deoxyfluorination mixed system on 3 mmol scale.

Table S6 [TBA][PO_2F_2] was prepared using other P-source[a,b].

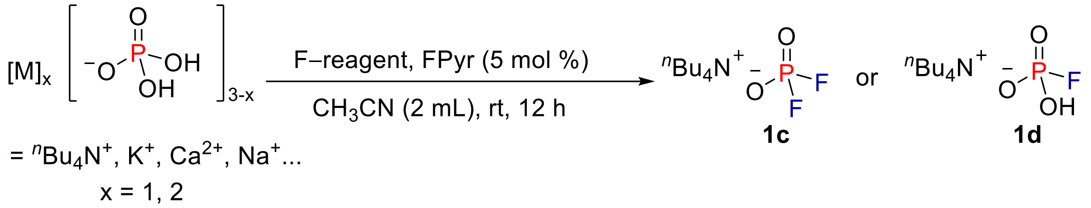

Entry	"P" Source	"F" reagent	Additive	T	Time	Yield %	
						1c	1d
1	K_3PO_4	Cyanuric fluoride	TBAF·3H_2O	rt	12 h	78	\
2[c]	$Na_3P_3O_9$	Cyanuric fluoride	TBAF·3H_2O	rt	12 h	Trace	86
3	$NH_4H_2PO_4$	Cyanuric fluoride	TBAF·3H_2O	rt	12 h	67	\
4	$H_4P_2O_7$	Cyanuric fluoride	TBAF·3H_2O	rt	12 h	70	\
5	KH_2PO_4	Cyanuric fluoride	TBAF·3H_2O	rt	12 h	84	\
6	NaH_2PO_4	Cyanuric fluoride	TBAF·3H_2O	rt	12 h	75	\
7	$CaHPO_4$	Cyanuric fluoride	TBAF·3H_2O	rt	12 h	72	\
8	P_2O_5	Cyanuric fluoride	TBAF·3H_2O	rt	12 h	86	\

Produced conditions for **1c**: [a] [TBA][H_2PO_4] (3 mmol), cyanuric fluoride (76 mol%), FPyr (5 mol%), CH_3CN (2 mL), rt, 12 h. [b] Other P-sources (3 mmol), cyanuric fluoride (76 mol%), FPyr (5 mol%), TBAF·3H_2O (K_3PO_4, $NH_4H_2PO_4$, KH_2PO_4, NaH_2PO_4, $CaHPO_4$: 1.0 eq.; $H_4P_2O_7$: 2.0 eq.; $Na_3P_3O_9$: 3.0 eq.; P_2O_5: 4.0 eq. Cyanuric fluoride ($H_4P_2O_7$: 1.5 eq.) (TCT CH_3CN (2 mL), rt, 12 h. [c] Produced conditions for **1d**: $Na_3P_3O_9$ (3 mmol), Cyanuric fluoride (2 eq.), TBAF·3H_2O (3.0 eq.), FPyr (5 mol%), CH_3CN (2 mL), rt, 12 h.

Table S7 General procedure for the preparation of [TBA][PO_2F_2].

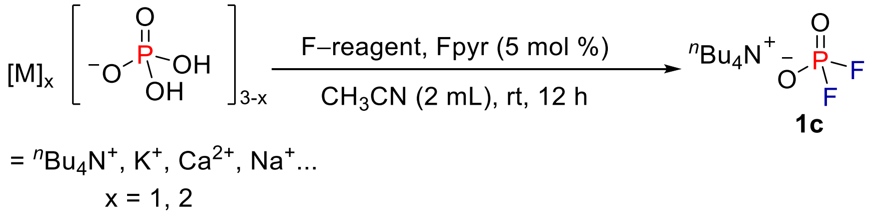

Method 1: [TBA][PO_2F_2] was prepared using $CaHPO_4$ as a P-source.

A solution of the $CaHPO_4$ (3 mmol) and TBAF·3H_2O (3 mmol, 1.0 eq.) at 0 °C in CH_3CN (2 mL), followed by DAST (3 mmol, 1.0 eq.) was slowly added to it and stirred vigorously, after the end of the addition, the temperature was raised to room temperature and the reaction was continued for 12 h. The precipitate was removed by filtering. The obtained filtrate was

concentrated in vacuo and the resulting crude product was purified by recrystallization with DCM and petroleum ether to provide product [TBA][PO_2F_2] **1c** as a white solid.

Compound 1c, physical state: white solid; **TLC** R_f = 3.0 (DCM: MeOH = 20:1); **^{1}H NMR** (400 MHz, Chloroform-d) δ = 3.25 – 3.18 (m, 8H), 1.66 – 1.56 (m, 8H), 1.44 – 1.36 (m, 8H), 0.96 (t, J=7.3, 12H). **^{31}P NMR** (162 MHz, Chloroform-*d*) δ = -7.86 – -24.48 (t, *J*= 957.42). **^{19}F NMR** (376 MHz, Chloroform-*d*) δ = -79.33 (d, *J*=948.3). **^{13}C NMR** (151 MHz, Chloroform-*d*) δ = 58.5, 23.7, 19.5, 13.4.

Method 2: [TBA][PO_2F_2] was prepared using [TBA][H_2PO_4] as P-source.

A solution of [TBA][H_2PO_4] (3 mmol), cyanuric fluoride (2.28 mmol, 76 mol%), and FPyr (0.15 mmol, 5 mol%) in CH_3CN (2 mL) was stirred at room temperature for 12 hours. The precipitate was removed by filtration, and the filtrate was concentrated under reduced pressure. The crude product was purified by recrystallization from DCM and petroleum ether to yield [TBA][PO_2F_2] **1c** as a white solid.

Method 3: [TBA][PO_2F_2] were prepared using different P-source.

A solution of the different "P" source ($CaHPO_4$, K_3PO_4, P_2O_5, $Na_3P_3O_9$, PPi, and various [M][H_2PO_4] salts (M = K^+, Na^+, NH_4^+) (3 mmol), cyanuric fluoride (x mol %), TBAF·3H_2O (y eq.) and FPyr (0.15 mmol, 5 mol%) in CH_3CN (2 mL) was stirred at room temperature for 12 h. The precipitate was removed by filtering. The obtained filtrate was concentrated in vacuo and the resulting crude product was purified by recrystallization with DCM and petroleum ether to provide product [TBA][PO_2F_2] **1c** as a white solid (Table S6).

Table S8 [TBA][PO_2F_2] was prepared by "F-Cl" exchange.

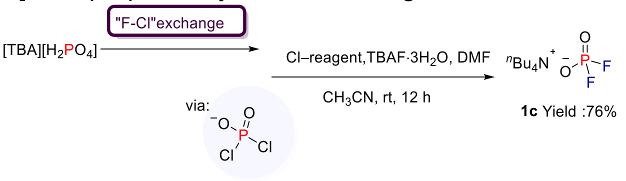

Entry	"F" reagent	Additive	Time	Yield (%)
1	NaF (2 eq.)	TCT (76 mol %)	12 h	trace
2	KF (2 eq.)	TCT (76 mol %)	12 h	--
3	TBAF·3H_2O (2 eq.)	TCT (76 mol %)	12 h	76
4	CsF (2 eq.)	TCT (76 mol %)	12 h	--

[a] F-Cl exchange: [TBA][H_2PO_4] (3 mmol), TCT (76 mol%), TBAF·3H_2O (2.0 eq.), CH_3CN (2 mL), rt, 12 h.

General procedure for the preparation of [TBA][PO_2F_2] by "F-Cl" exchange.

A solution of the [TBA][H_2PO_4] (3 mmol), TCT (2.28 mmol, 76 mol %), TBAF·3H_2O (7.5 mmol, 2.5 eq.), and FPyr (0.15 mmol, 5 mol%) in CH_3CN (2 mL)was stirred at room temperature for 12 h. The precipitate was removed by filtering. The obtained filtrate was concentrated in vacuo and the resulting crude product was purified by recrystallization with DCM and petroleum ether to provide product [TBA][PO_2F_2] **1c** as a white solid.

2.3 Scale-up experiments of raw materials [TBA][PO_2Cl_2] and [TBA][PO_2F_2].

Table S9 General procedure for the preparation of [TBA][PO_2Cl_2].

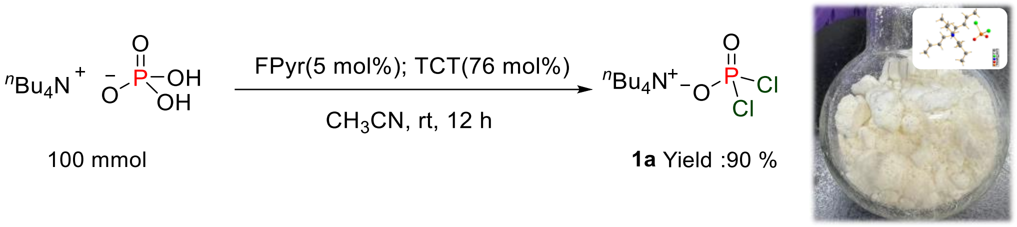

A solution of the [TBA][H_2PO_4] (33.9 g, 100 mmol), and TCT (14.0 g, 76 mmol, 76 mol%) and FPyr (0.48 mL, 5 mmol, 5 mol%) in CH_3CN (40 mL) was stirred at room temperature for 12 h. The precipitate was removed by filtering. The obtained filtrate was concentrated in vacuo and the resulting crude product was purified by recrystallization with DCM and petroleum ether to provide product [TBA][PO_2Cl_2] **1a** (33.7 g, 90%) as a white solid.

Table S10 General procedure for the preparation of [TBA][PO_2F_2].

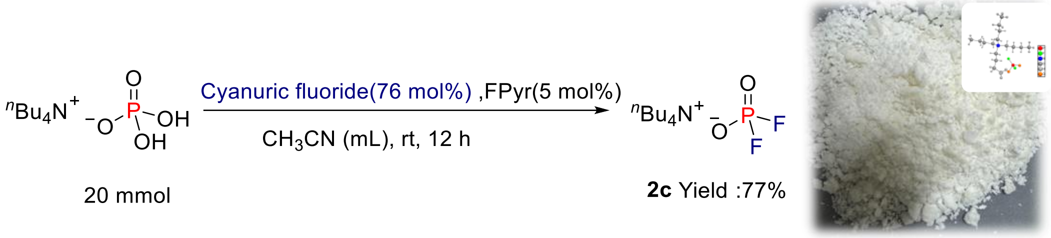

A solution of the [TBA][H_2PO_4] (33.9 g, 20 mmol) and Cyanuric fluoride (1.3 mL, 15.2 mmol,76 mol%) and FPyr (0.09 mL, 1 mmol, 5 mol%) in CH_3CN (10 mL) was stirred at room temperature for 12 h. The precipitate was removed by filtering. The obtained filtrate was concentrated in vacuo and the resulting crude product was purified by recrystallization with DCM and petroleum ether to provide product [TBA][PO_2F_2] **1c** (5.3 g, 77%) as a white solid.

2.4 Stability tests for 1a [TBA] [PO_2Cl_2] and 1c [TBA] [PO_2F_2].

Table S11 Comparison of the stability of **1a** and **1c** under different conditions

Equal amounts of **1a** and **1c** were subjected to regular phosphorus spectrum monitoring under different conditions to verify the stability of the compounds. The specific monitoring experiments are illustrated in the figures below:

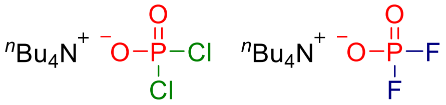

1a **1c**

**1a** stability	**1a** in D_2O	0 h					
	**1a** in $CDCl_3$	0-3 d	3-9 d				
	**1a** in Air	0-3 d	3-7 d				
	**1a** in a closed environment	0-3 d	3-6 d	6-12 d	12-18 d	18-24 d	24-30 d
**1c** stability	**1c** in $CDCl_3$	0-6 month					
	**1c** in D_2O	0-3 d	3-6 d	6-12 d	12-18 d	18-24 d	24-30 d

Note: stabilization relatively stable decomposition

a: $PO_2Cl_2^-$ is not stable in the presence of water, as it decomposes rapidly in deuterated water to form phosphate. However, in deuterated chloroform, the compound dimerizes slowly, indicating that it is more stable in organic solvents.

b: We have monitored the stability of $PO_2Cl_2^-$ in air and found that it also decomposes slowly under these conditions, with complete decomposition occurring after 6 days. In a sealed environment, however, the compound remains stable for up to 30 days without any signs of decomposition.

c: Regarding $PO_2F_2^-$, we have observed that it is more stable than $PO_2Cl_2^-$, both in deuterated chloroform and deuterated water. Our monitoring experiments indicate that the phosphorus fluoride compound can remain stable in deuterated chloroform for up to six months and is also stable in deuterated water.

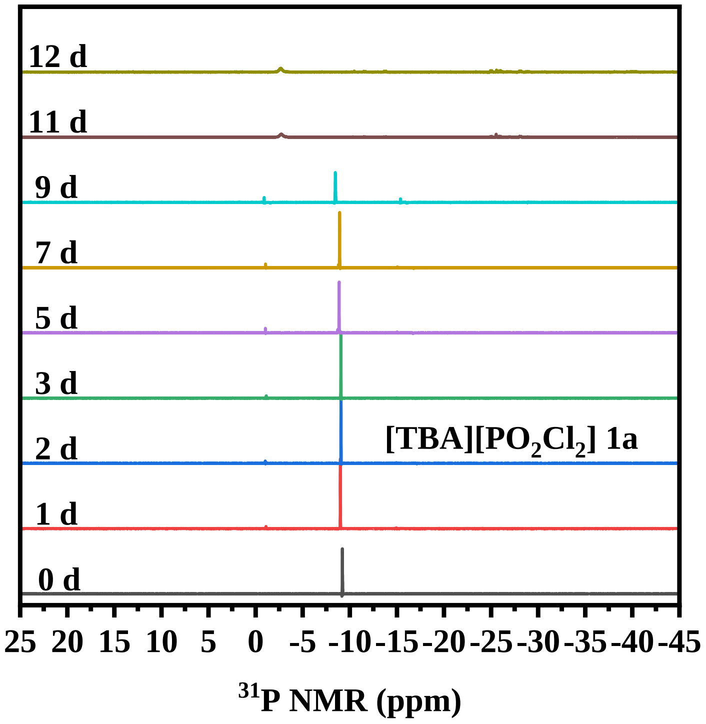

Figure S2 Monitoring the stability of [TBA][PO_2Cl_2] in $CDCl_3$

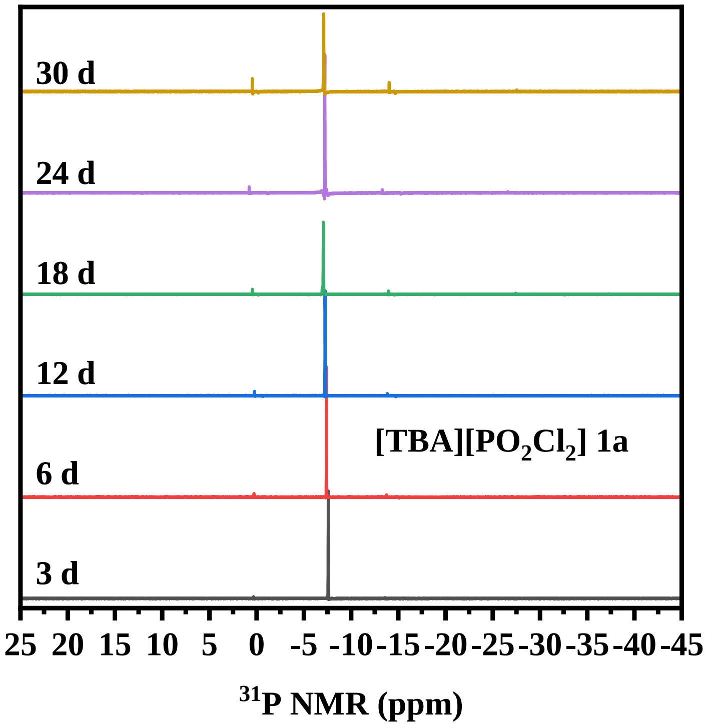

Figure S3 Monitoring the stability of [TBA][PO_2Cl_2] in enclosed environment

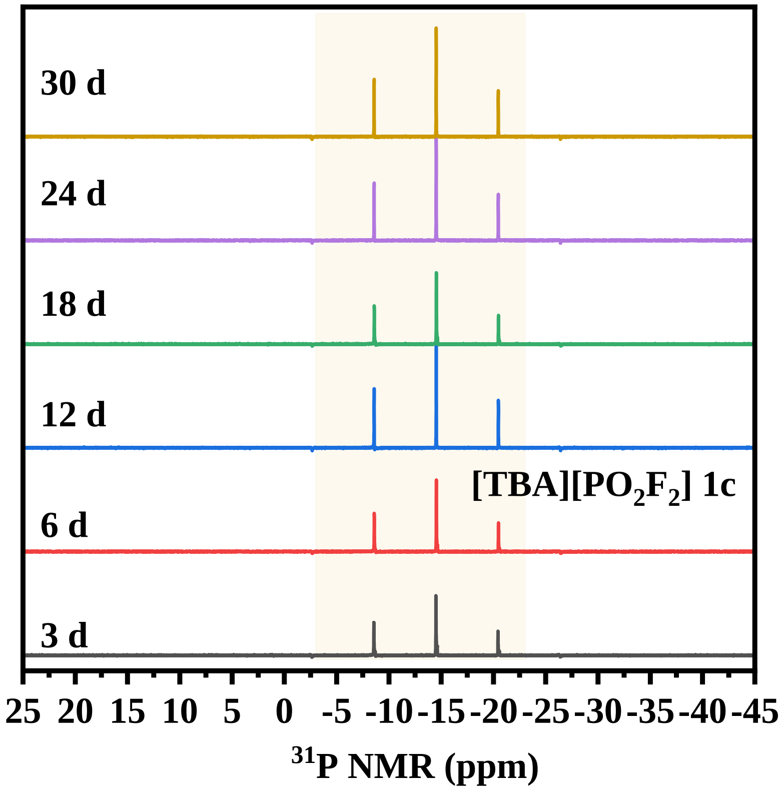

Figure S4 Monitoring the stability of [TBA][PO_2F_2] in D_2O

3 Application of the P-X reagents

Table S12 Conditional screening of [TBA][PO_2Cl_2] reaction with oxygen nucleophiles.

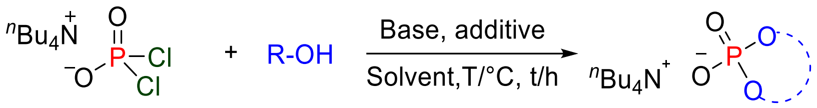

Entry	[TBA][PO_2Cl_2] (mmol)	R-OH (mmol)	Solvent (1 mL)	Base	Additive (2.5 eq.)	T/ ℃	t / h	Yield (%)
1	0.2	0.5	THF	NaOH	$Na_2S_2O_3.5H_2O$	40	1	76
2	0.2	0.5	THF	\	$Na_2S_2O_3.5H_2O$	40	1	N.D.
3	0.2	0.5	THF	NaOH	\	40	1	52
4	0.2	0.5	CH_2Cl_2	NaOH	$Na_2S_2O_3.5H_2O$	40	1	trace
5	0.2	0.5	CH_3CN	NaOH	$Na_2S_2O_3.5H_2O$	40	1	trace
6	0.2	0.5	MeOH	NaOH	$Na_2S_2O_3.5H_2O$	40	1	N.D.
7	0.2	0.5	Acetone	NaOH	$Na_2S_2O_3.5H_2O$	40	1	16

8	0.2	0.5	DME	NaOH	$Na_2S_2O_3.5H_2O$	40	1	23
9	0.2	0.5	DCE	NaOH	$Na_2S_2O_3.5H_2O$	40	1	22
10	0.2	0.5	Dioxane	NaOH	$Na_2S_2O_3.5H_2O$	40	1	34
11	0.2	0.2	THF	NaOH	$Na_2S_2O_3.5H_2O$	40	1	21
12	0.2	0.3	THF	NaOH	$Na_2S_2O_3.5H_2O$	40	1	37
13	0.2	0.5	THF	KOH	$Na_2S_2O_3.5H_2O$	40	1	N.R.
14	0.2	0.5	THF	CH_3ONa	$Na_2S_2O_3.5H_2O$	40	1	N.R.
15	0.2	0.5	THF	Et_3N	$Na_2S_2O_3.5H_2O$	40	1	N.D.

[a] Standard conditions: [TBA][PO_2Cl_2] (0.2 mmol), R-OH (0.5 mmol, 2.5 eq.), NaOH (0.5 mmol, 2.5 eq.), $Na_2S_2O_3.5H_2O$ (0.5 mmol, 2.5 eq.) in THF (1 mL), at 40 ℃ for 1 h.

Table S13 General procedure of [TBA][PO_2Cl_2] reaction with oxygen nucleophiles.

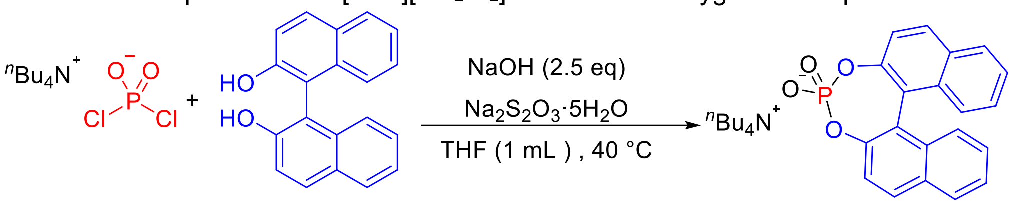

[TBA][PO_2Cl_2] (0.2 mmol) was added in a solution of bisnaphthol (0.5 mmol, 2.5 eq.), NaOH (0.5 mmol, 2.5 eq.) and $Na_2S_2O_3{\cdot}5H_2O$ (0.5 mmol, 2.5 eq.) in 1 mL THF and the reaction mixture stirred at 40℃ for 1 h. The aqueous layer was extracted with methylene chloride (3 × 10.0 mL) and the organic extracts were combined, dried over anhydrous sodium sulfate and concentrated in vacuo. The residue was purified by column chromatography (silica gel, eluent DCM : MeOH = 100:1 ~ 20:1) to yield the desired product **I-1**.

Compound I-1, Physical state: White solid; **TLC:** R_f = 0.44 (DCM/MeOH = 20:1); Yield: 76% **^{1}H NMR (400 MHz, Chloroform-*d*)** δ = 7.84 – 7.80 (m, 4H), 7.46 (d, *J*=8.8, 2H), 7.33 – 7.27 (m, 4H), 7.15 (t, *J*=7.2, 2H), 2.86 – 2.82 (m, 8H), 1.26 – 1.21 (m, 8H), 1.14 – 1.09 (m, 8H), 0.76 (t, *J*=7.2, 12H). **^{31}P NMR** (162 MHz, Chloroform-*d*) δ = 6.21. **^{13}C NMR** (101 MHz, Chloroform-*d*) δ = 150.4 (d, *J*=9.0), 132.5 (d, *J*=1.0), 130.6 (d, *J*=1.), 129.6, 128.1, 126.8, 125.6, 124.2, 122.8 (d, *J*=3.0), 122.3 (d, *J*=2.0), 57.8, 23.5, 19.4, 13.5.

Table S14 General procedure of [TBA][PO_2Cl_2] reaction with sulfur nucleophiles.

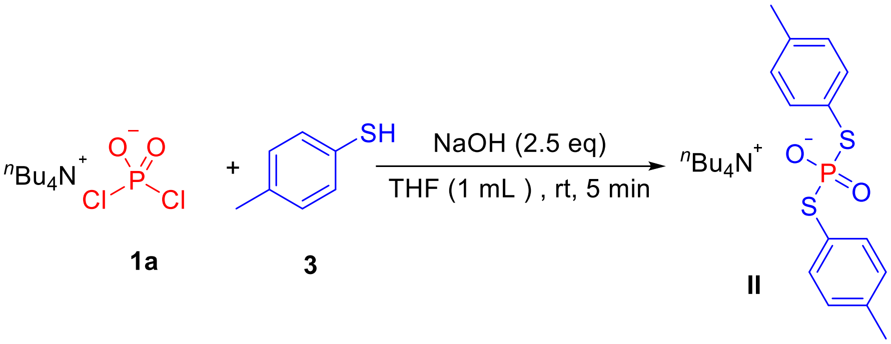

[TBA][PO_2Cl_2] (0.2 mmol) was added in a solution of *p*-toluenethiol (0.5 mmol, 2.5 eq.), NaOH (0.5 mmol, 2.5 eq.) in 1 mL THF and the reaction mixture stirred at room temperature for 5 min and water (10.0 mL) was added. The aqueous layer was extracted with methylene chloride (3 × 10.0 mL) and the organic extracts were combined, dried over anhydrous sodium sulfate and concentrated in vacuo. The residue was purified by column chromatography (silica gel, eluent DCM: MeOH = 500:1) to yield the desired product **II-1**.

Compound II-1, physical state: White solid; **TLC:** R_f = 0.72 (DCM/MeOH = 100:1); Yield: 72%. ^{1}**H NMR** (400 MHz, Chloroform-*d*) δ = 7.47 (d, *J*=6.8, 4H), 6.96 (d, *J*=8.0, 4H), 3.16 – 3.10 (m, 8H), 2.24 (s, 6H), 1.54 – 1.45 (m, 8H), 1.37 – 1.29 (m, 8H), 0.91 (t, *J*=7.2, 12H). ^{31}**P NMR** (162 MHz, Chloroform-*d*) δ = 24.45. ^{13}**C NMR** (151 MHz, Chloroform-*d*) δ = 135.7, 133.2 (d, *J*=4.5), 131.4 (d, *J*=4.5), 128.8, 58.5, 23.9, 21.0, 19.6, 13.7.

Table S15 General procedure of [TBA][PO_2Cl_2] reaction with nitrogen nucleophiles.

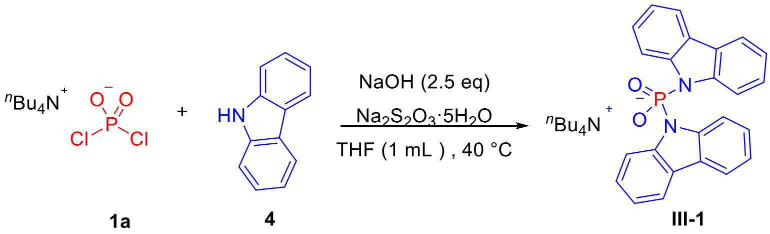

[TBA][PO_2Cl_2] (0.2 mmol) was added in a solution of carbazole (0.5 mmol, 2.5 eq.), NaOH (0.5 mmol, 2.5 eq.) and $Na_2S_2O_3$·5H_2O (0.5 mmol, 2.5 eq.) in 1 mL THF and the reaction mixture stirred at 40℃ for 3 h. The aqueous layer was extracted with methylene chloride (3 × 10.0 mL) and the organic extracts were combined, dried over anhydrous sodium sulfate and concentrated in vacuo. The residue was purified by column chromatography (silica gel, eluent DCM: MeOH = 100:1~20:1) to yield the desired product **III-1**.

Compound III-1, physical state: White solid; R_f = 0.42 (DCM/MeOH = 20:1); Yield: 88%. ^{1}**H NMR** (400 MHz, Chloroform-*d*) δ = 8.38 (d, *J*=8.4, 4H), 7.83 (d, *J*=7.6, 4H), 7.18 (t, *J*=7.2, 4H),

7.07 (t, *J*=7.2, 4H), 2.50 – 2.46 (m, 8H), 1.06 – 0.96 (m, 8H), 0.94 – 0.86 (m, 8H), 0.72 (t, *J*=7.2, 12H). **^{31}P NMR** (162 MHz, Chloroform-*d*) δ = -13.39. **^{13}C NMR** (151 MHz, Chloroform-*d*) δ = 142.3 (d, *J*=4.5), 125.5, 124.9 (d, *J*=7.5), 119.7, 118.9, 115.6, 57.8, 23.4, 19.2, 13.5.

Table S16 General procedure of [TBA][PO_2Cl_2] reaction with carbon nucleophiles.

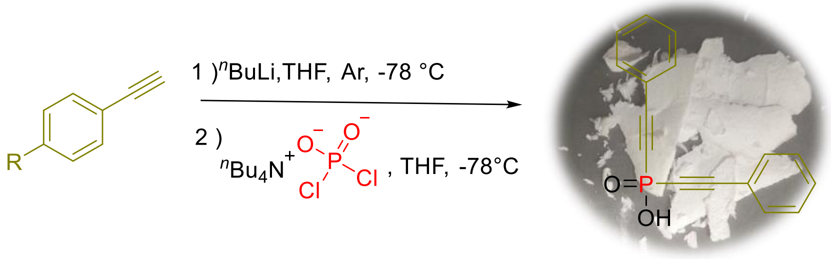

Method A: For example, the synthesis of IV-1. Under an inert atmosphere, a solution of phenylacetylene (0.5 mmol) in THF (2 mL) was prepared at 0 °C. An equivalent amount of n-butyl lithium (^{n}BuLi) (1 M in diethyl ether) was slowly added, and the mixture was vigorously stirred for 2 hours. Subsequently, [TBA][PO_2Cl_2] (0.2 mmol) was added, and the reaction was allowed to warm to room temperature and stirred for an additional hour. A saturated aqueous solution of ammonium chloride (10.0 mL) was then added, The aqueous layer was extracted with DCM (3 × 10.0 mL) and the organic extracts were combined, dried over anhydrous sodium sulfate and concentrated in vacuo. The residue was purified by column chromatography (silica gel, eluent DCM: MeOH = 10:1) to yield the desired product **IV-1**.

Compound IV-1, physical state: White solid; **TLC** R_f = 0.32 (DCM/MeOH = 10:1); Yield: 78%. **^{1}H NMR** (400 MHz, DMSO-*d*6) δ = 7.46 – 7.43 (m, 4H), 7.38 (s, 6H).**^{31}P NMR** (162 MHz, DMSO-*d*6) δ = -32.91. **^{13}C NMR** (151 MHz, Chloroform-*d*) δ = 136.7, 134.3, 133.9, 126.9 (d, *J*=4.5), 98.7 (d, *J*=208.5), 96.0 (d, *J*=37.5).

The synthesis of IV-4. In an inert gas environment, a solution of the bromobenzene (0.5 mmol) at 0 °C in THF (2 mL), followed by an equal amount of n-butyl lithium (^{n}BuLi) (1 M in diethyl ether) slowly added to it and stirred vigorously for 2 h. Following this, [TBA][PO_2Cl_2] (0.2 mmol) was added. Once the addition was complete, the reaction was allowed to warm to room temperature for 1 h, and saturated water solution of ammonium chloride (10.0 mL) was added. The aqueous layer was extracted with DCM (3 × 10.0 mL) and the organic extracts were combined, dried over anhydrous sodium sulfate, and concentrated in vacuo. The residue was purified by column chromatography (silica gel, eluent DCM: MeOH = 10:1~5:1) to yield the desired product **IV-4**

Compound IV-4, physical state: White solid; **TLC:** R_f = 0.14 (DCM/MeOH = 10:1); Yield: 59%. ^{1}H NMR (400 MHz, DMSO-d_6) δ = 7.67 – 7.63 (m, 4H), 7.22 (m, 6H). ^{31}P NMR (162 MHz, DMSO-d_6) δ = 11.27. ^{13}C NMR (151 MHz, DMSO-d_6) δ = 131.67 (d, *J*=8.4), 128.72, 127.53 (d, *J*=11.0), 120.79.

Table S17 Examples of cation exchange of organophosphate salts. [11, 12]

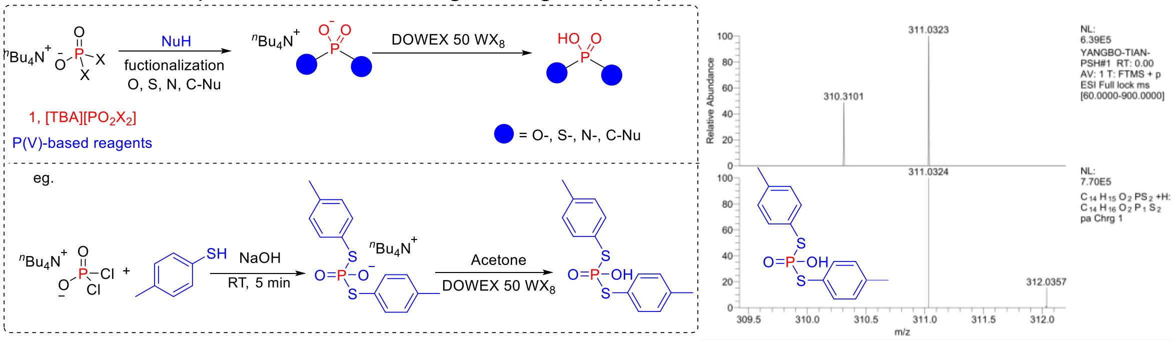

[TBA][PO_2Cl_2] (0.2 mmol) was added in a solution of *p*-toluenethiol (0.5 mmol, 2.5 eq.), NaOH (0.5 mmol, 2.5 eq.) in 1 mL THF and the reaction mixture stirred at room temperature for 5 minute and saturated salt water (10.0 mL) was added. The aqueous layer was extracted with methylene chloride (3 × 10.0 mL) and the organic extracts were combined, dried over anhydrous sodium sulfate and concentrated in vacuo. After that, acetone was added as the solvent, cation exchange resin (DOWEX 50 WX8) (15 mg) was added and stirred at room temperature for 30 min, cation exchange resin was removed by filtering, concentrated filtrate, The residue was purified by column chromatography (silica gel, eluent DCM: MeOH = 20:1) to yield the desired product **II-1a**.

Compound II-Ia, physical state: White solid; **TLC** (DCM : MeOH = 20:1): R_f = 0.6; Yield: 82%. **^{1}H NMR** (400 MHz, DMSO-d_6) δ = 7.35 (d, *J*=7.6, 4H), 7.02 (d, *J*=7.6, 4H), 2.25 (s, 6H). **^{31}P NMR** (162 MHz, DMSO-d_6) δ = 21.60. **^{13}C NMR** (151 MHz, DMSO-d_6) δ = 135.8, 133.1 (d, *J*=4.5), 131.8 (d, *J*=4.5), 129.2, 21.1.

Table S18 General Procedure for acidification reaction.

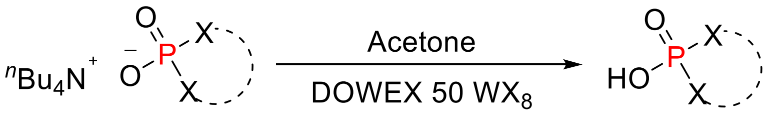

I, II, III

X = O, S, N

Method A, two-step procedures: A solution of product **I**, **II**, or **III** (0.2 mmol) and cation exchange resin (DOWEX 50 WX8) (15 mg) in acetone (2 mL) was stirred at room temperature

for 30 minutes. The resin was removed by filtration, and the filtrate was concentrated under reduced pressure. The crude product was purified by recrystallization from DCM and petroleum ether to yield products **I-a**, **II-a**, or **III-a** as white solids.

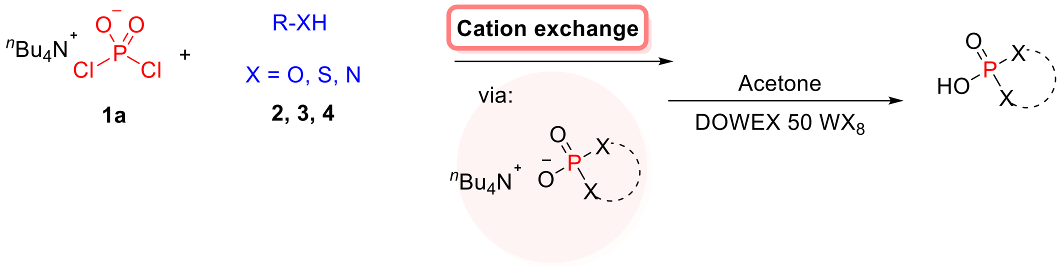

Method B, in one-pot procedure: [TBA][PO_2Cl_2] (0.2 mmol) was added in a solution of the compound (**2** or **3** or **4**) (0.5 mmol, 2.5 eq.), NaOH (0.5 mmol, 2.5 eq.) in 1 mL THF and the reaction mixture stirred at rt – 40 °C for 5 min – 3 h (Table S12-Table S14). After the reaction was over and saturated salt water (10.0 mL) was added. The aqueous layer was extracted with DCM (3 × 10.0 mL) and the organic extracts were combined, dried over anhydrous sodium sulfate and concentrated in vacuo. After that, acetone was added as the solvent, and cation exchange resin (DOWEX 50 WX8) (15 mg) was added and stirred at room temperature for 30 min, Cation exchange resin was removed by filtering. The obtained filtrate was concentrated in vacuo and the resulting crude product was purified by recrystallization with methylene chloride and petroleum ether to obtain product **I-a**, **II-a**, **III-a**, as a white solid.

4 Research on reaction mechanism

4.1 Experimental verifications

4.1.1 Control experiments

By controlling the time variable and using different TBACl stoichiometry, ^{31}P NMR monitoring was performed during the reaction to generate **1a** to facilitate the observation of potential intermediates. The results show that by controlling the time, a new shift of the phosphorus spectrum is detected at 28 ppm, which may be tripolyphosphate. Secondly, through the TBACl amount control experiment, a peak of 11 ppm phosphate dimer was obtained. Therefore, we speculate that oligophosphates may have lower energy than a single phosphate molecule and are more likely to participate in the reaction to obtain the target product. As shown in Table S19-S20 and Figure S5-S6:

A: effect of variable time

Table S19 ^{31}P NMR monitoring was carried out for the process of reaction for **1a**

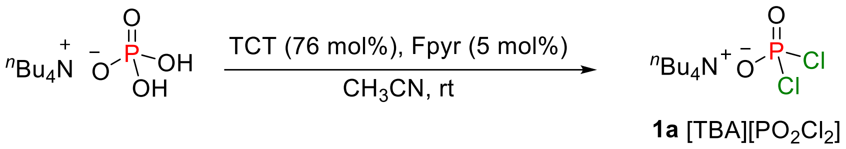

Under standard conditions, we performed a ^{31}P NMR tracking experiment on the reaction process of generating 1a. The same amount of reaction system was taken at 3 h, 6 h, and 12 h and dissolved in $CDCl_3$ to perform phosphorus spectrum analysis. The results are as follows :

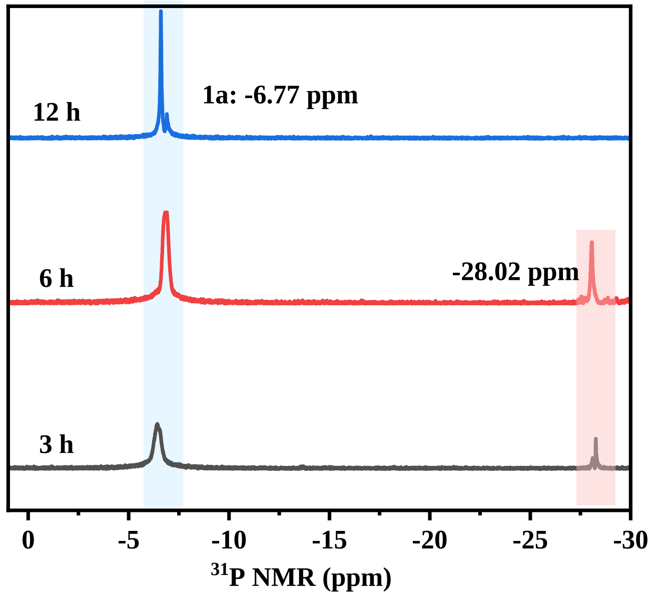

Figure S5 The change of 1a ^{31}P NMR (in $CDCl_3$) with time

B: Effect of TBAC's amount

Table S20 ^{31}P NMR monitoring with the addition of TBACl was used for the mixing reaction process.

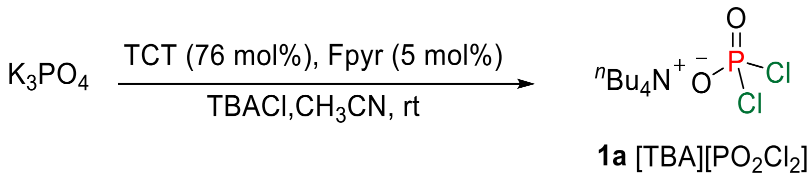

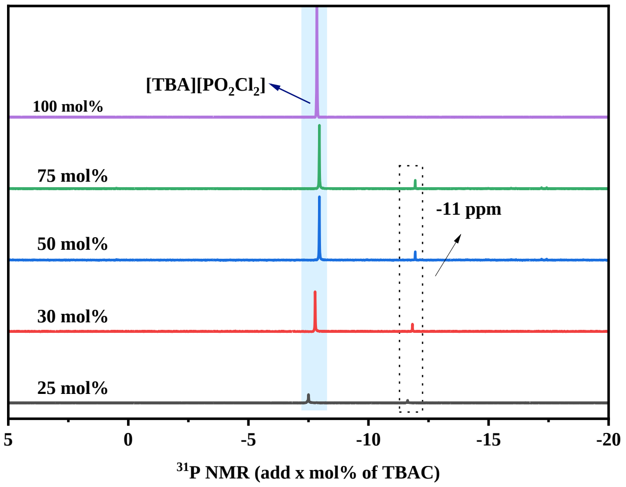

Figure S6 ^{31}P NMR monitoring the effect of TBAC in the reaction for **1a**

4.1.2 Spectrometric detection of the intermediates

Table S21 Experiment detecting the intermediates.

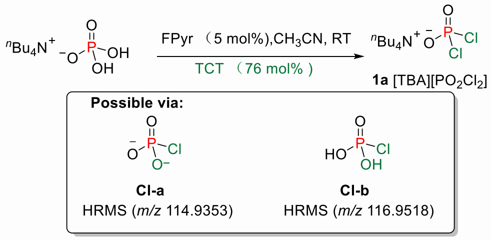

The FPyr, intermediate B, compound CA, and monochlorination products **Cl-a** $[M+H]^+$ (*m/z* 114.9353) and **Cl-b** $[M+H]^+$ (*m/z* 178.9520) were successfully detected by high-resolution massspectrometry (HRMS), dipolyphosphoric acid is also found by high-resolution massspectrometry (HRMS), laying the data support for the proposed mechanism. By monitoring the formation of **1a**, new phosphorus chemical shifts of − 28 ppm and − 11 ppm were generated. − 28 ppm phosphorus easily hydrolyzed and polymerized into other − 12 ppm and − 19 ppm phosphates during the separation process. Through hydrogen spectrum analysis, it was found that there were only tetrabutyl and active hydrogen peaks. We speculate that these substances may be oligomeric products of some phosphates.[15, 16]

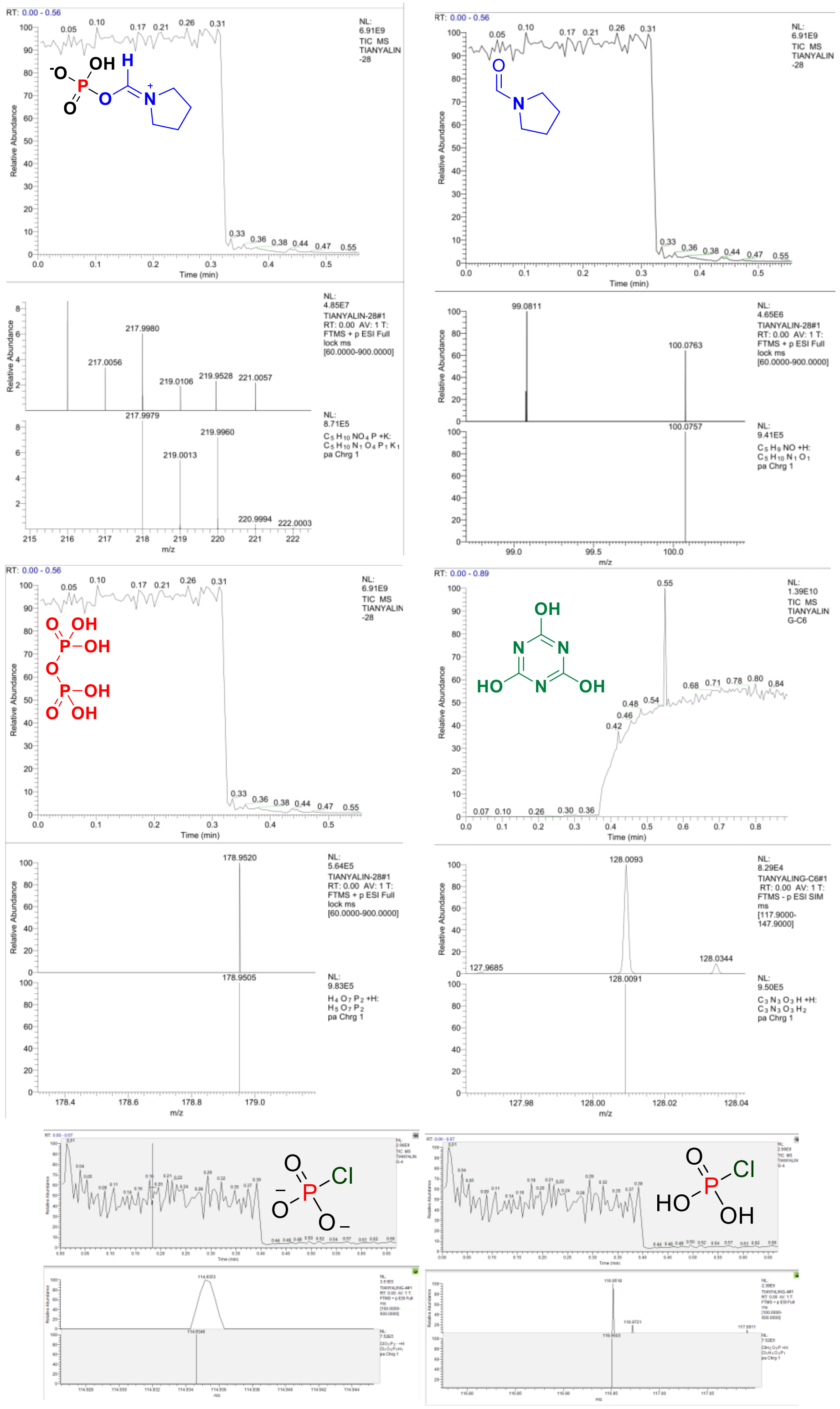

Figure S7 HRMS of intermediate conversion

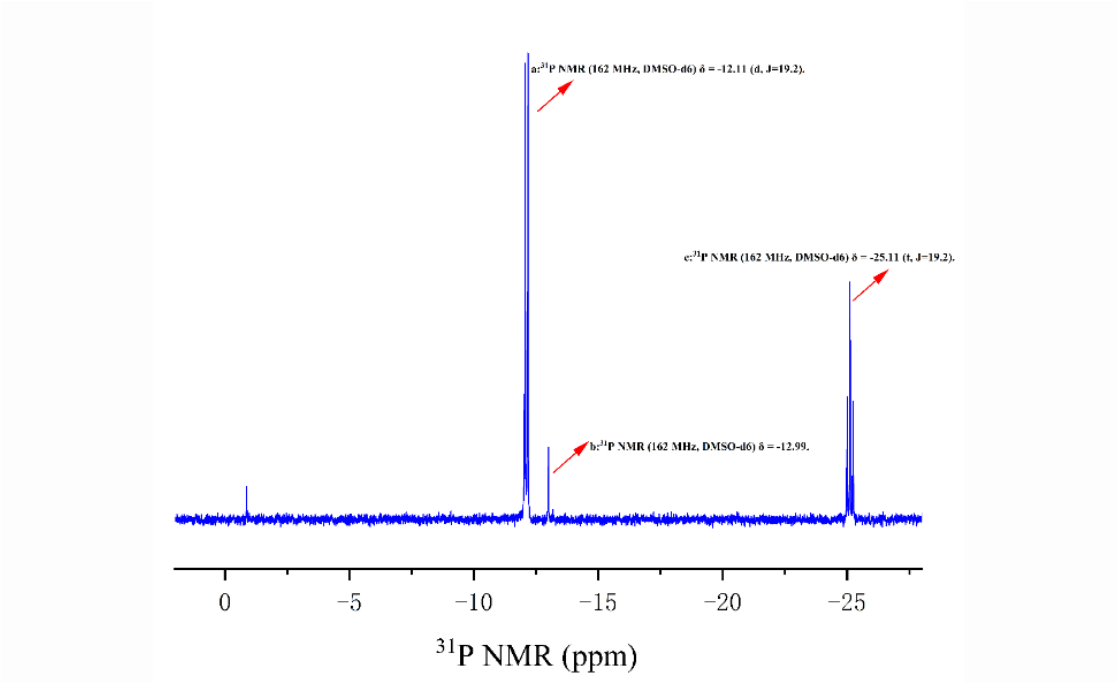

Figure S8 ^{31}P NMR spectra in DMSO-d$_6$ of intermediate conversion

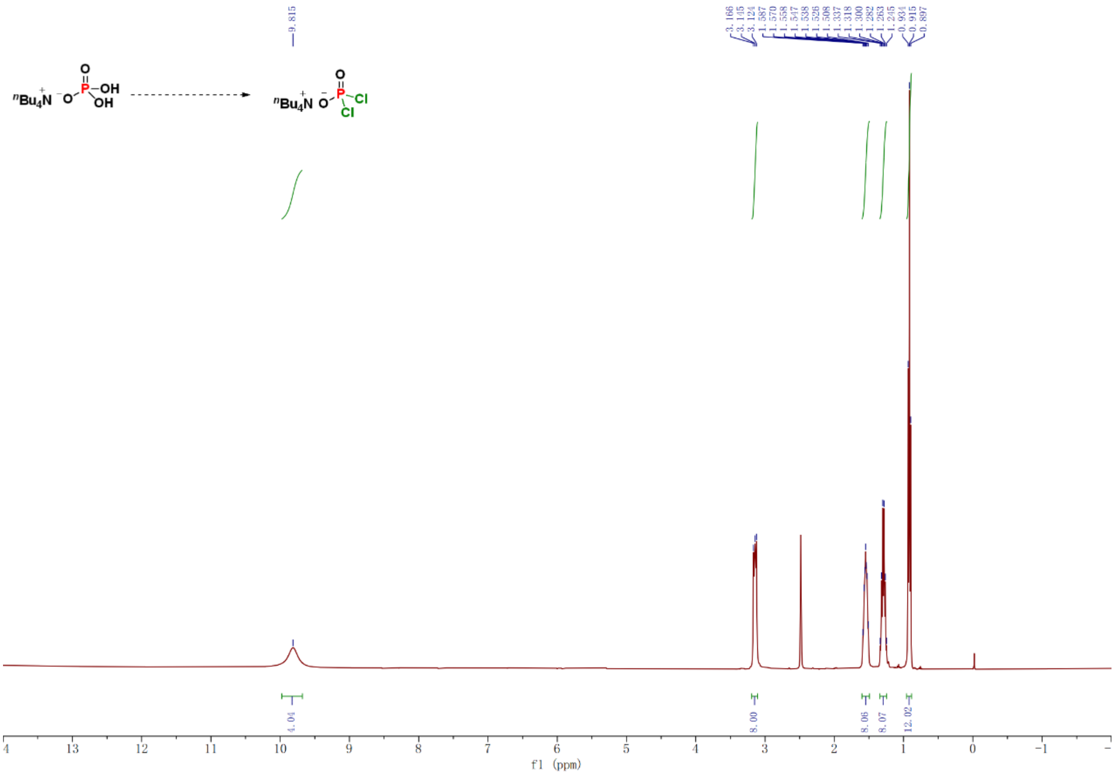

Figure S9 ^{1}H NMR spectra in DMSO-d$_6$ of intermediate conversion

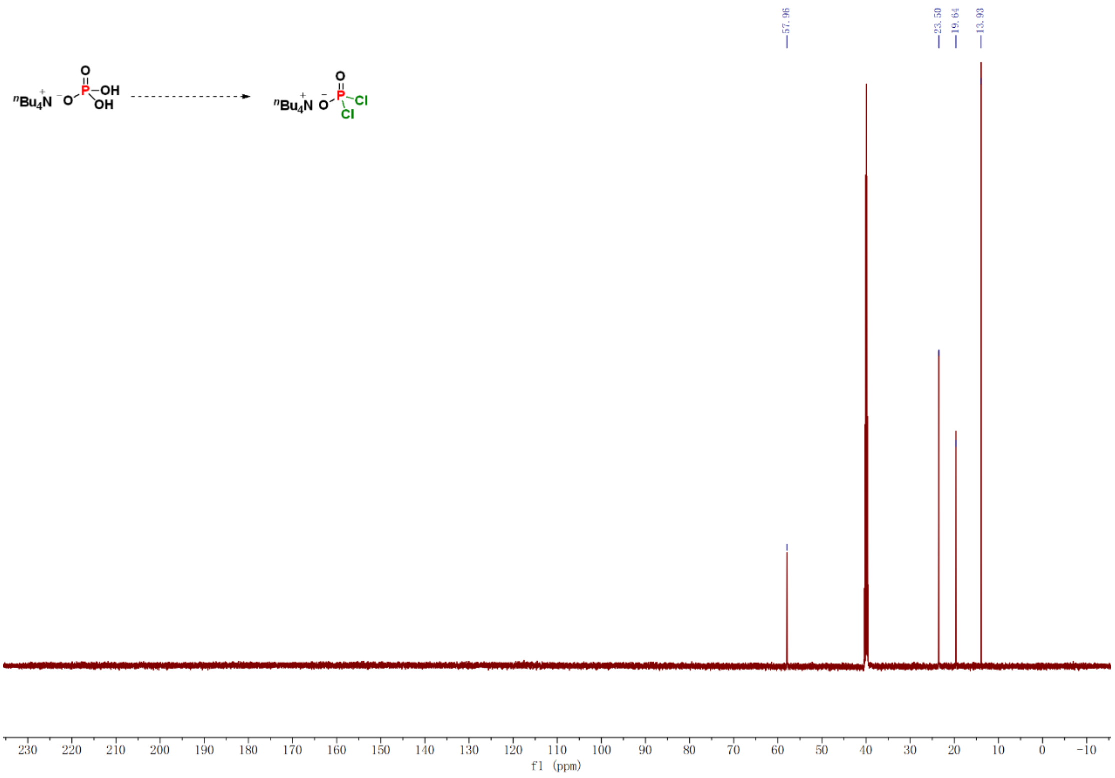

Figure S10 ^{13}C NMR spectra in DMSO-d_6 of intermediate conversion

Taking potassium phosphate (K_3PO_4) as an example, the effect of TBAC on the reaction system was studied by NMR and IR. The experimental results show that the addition of TBAC will affect the changes in NMR and IR peaks of phosphate ions in the reaction mixture. The experimental results are as follows:

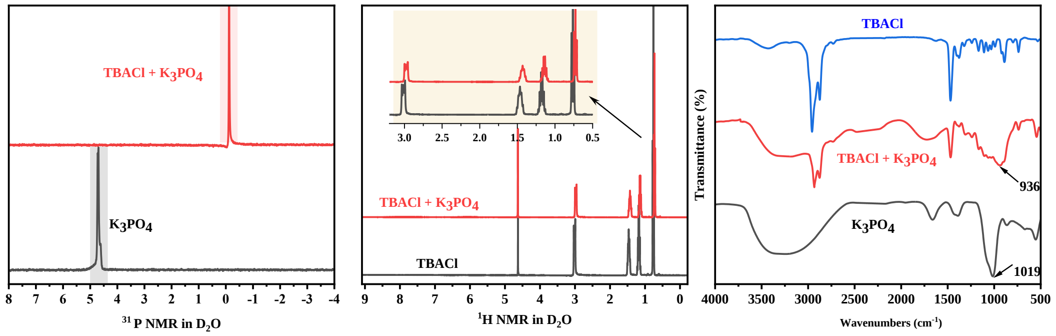

Figure S11 Effect of TBACl on NMR and IR of K_3PO_4.

4.2 Synthesis of 1a using Vilsmeier reagent

According to the contents of S2.1 and Table S3, the experimental results showed that the corresponding chlorination products could also be obtained by activating [TBA][H_2PO_4] with

Vilsmeier reagent alone. The product **1a** was obtained by using 10 mmol [TBA][H_2PO_4], and the yield was 85 %.

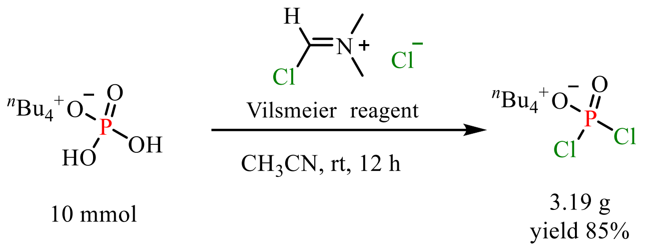

4.3 Recovery experiment of cyanuric acid

We conducted experiments using oxalyl chloride, thionyl chloride, and triphosgene as chlorination agents. The results indicated that thionyl chloride($SOCl_2$) and phosphorus oxychloride($POCl_3$) could serve as chlorine reagents under unoptimized conditions, achieving a CA recovery.

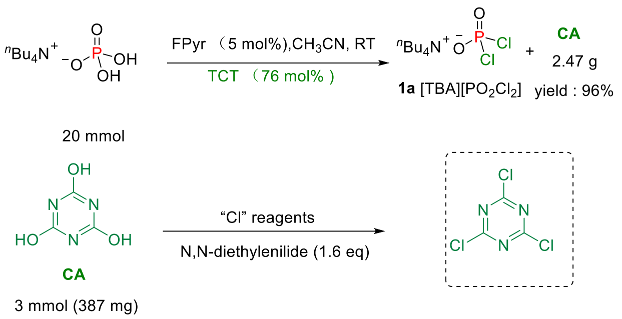

Table S22 Recovery experiment of cyanuric acid.

Entry	CA	"Cl" reagent	Additive	T/℃	solvent	Yield
1	3 mmol	$SOCl_2$(3.3 eq)	N,N-Diethylaniline	30	CH_2Cl_2	57
2	3 mmol	BTC (1.5 eq)	N,N-Diethylaniline	30	CH_2Cl_2	N.R
3	3 mmol	$(COCl)_2$(3.3 eq)	N,N-Diethylaniline	30	CH_2Cl_2	N.R
4	3 mmol	$SOCl_2$(3.3 eq)	N,N-Diethylaniline	30	CH_3CN	N.R
5	3 mmol	BTC (1.5 eq)	N,N-Diethylaniline	30	CH_3CN	N.R
6	3 mmol	$(COCl)_2$(3.3 eq)	N,N-Diethylaniline	30	CH_3CN	N.R
7	18 mmol	$POCl_3$(2.5 eq)	N,N-Diethylaniline	105	\	76
8	3 mmol	$(COCl)_2$(3.3 eq)	DMF	30	CH_2Cl_2	\
9	3 mmol	$(COCl)_2$(3.3 eq)	DMF	30	CH_3CN	trace

A solution of the [TBA][H_2PO_4] (33.9 g, 20 mmol), and TCT (2.8 g, mmol, 76 mol%) and FPyr (0.096 mL, 1 mmol, 5 mol%) in CH_3CN (20 mL) was stirred at room temperature for 12

hours. The precipitate was obtained by filtration, washed with dichloromethane, and then recrystallized to obtain cyanuric acid with a yield of 96 %. Subsequently, $SOCl_2$ (1.14 mL, 3.3 eq) was slowly added to a mixture of cyanuric acid (387 mg, 3 mmol) and N,N-diethylenilide (0.75 mL, 1.6 eq) and reacted at 30 °C for 6 h to obtain recovered cyanuric chloride in a yield of 57%.

5 Control experiments of "$Na_2S_2O_3 \cdot 5H_2O$" for the synthesis of I-1

Performed under the conditions described in Section S3 Table.S12, the synthesis of I-1 was monitored by ^{31}P NMR. By controlling the time, the reaction was terminated at the corresponding time period, and then 0.5 mL of the reaction mixture was taken and $CDCl_3$ was added. The possible intermediates in the reaction process were monitored by ^{31}P NMR. However, the experimental results showed that no new phosphorus shift was observed during the reaction, but the addition of $Na_2S_2O_3 \cdot 5H_2O$ obviously promoted the reaction and improved the conversion rate. As shown in Table S23-S24 and Figure S12.

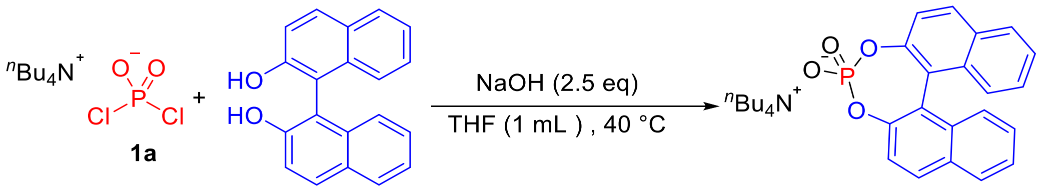

Table S23 Without "$Na_2S_2O_3 \cdot 5H_2O$" for the synthesis of I-1[a]

Entry	**1a**	**2**	Solvent(1 mL)	$Na_2S_2O_3 \cdot 5H_2O$	Base(2.5 eq)	T/ ℃	**Time**
1	0.2 mmol	0.5 mmol	THF	\	NaOH	40	**5 min**
2	0.2 mmol	0.5 mmol	THF	\	NaOH	40	**15 min**
3	0.2 mmol	0.5 mmol	THF	\	NaOH	40	**30 min**
4	0.2 mmol	0.5 mmol	THF	\	NaOH	40	**6 h**

[a] Standard conditions: **1a** (0.2 mmol), binaphthol (2.5 eq., 0.5 mmol), NaOH (2.5 eq., 0.5 mmol), THF (1 mL), 40 °C, 1-6 h.

Table S24 Add "$Na_2S_2O_3{\cdot}5H_2O$" for the synthesis of **I-1**[a]

Entry	**1a**	**2**	Solvent(1 mL)	$Na_2S_2O_3{\cdot}5H_2O$	Base(2.5 eq)	T/ ℃	**Time**
1	0.2 mmol	0.5 mmol	THF	0.5 mmol	NaOH	40	**5 min**
2	0.2 mmol	0.5 mmol	THF	0.5 mmol	NaOH	40	**15 min**
3	0.2 mmol	0.5 mmol	THF	0.5 mmol	NaOH	40	**30 min**
4	0.2 mmol	0.5 mmol	THF	0.5 mmol	NaOH	40	**1 h**

[a] Standard conditions: **1a** (0.2 mmol), Binaphthol (2.5 eq., 0.5 mmol), NaOH (2.5 eq., 0.5 mmol), $Na_2S_2O_3{\cdot}5H_2O$ (2.5 eq., 0.5 mmol), THF (1 mL), 40 °C, 5 min - 1 h.

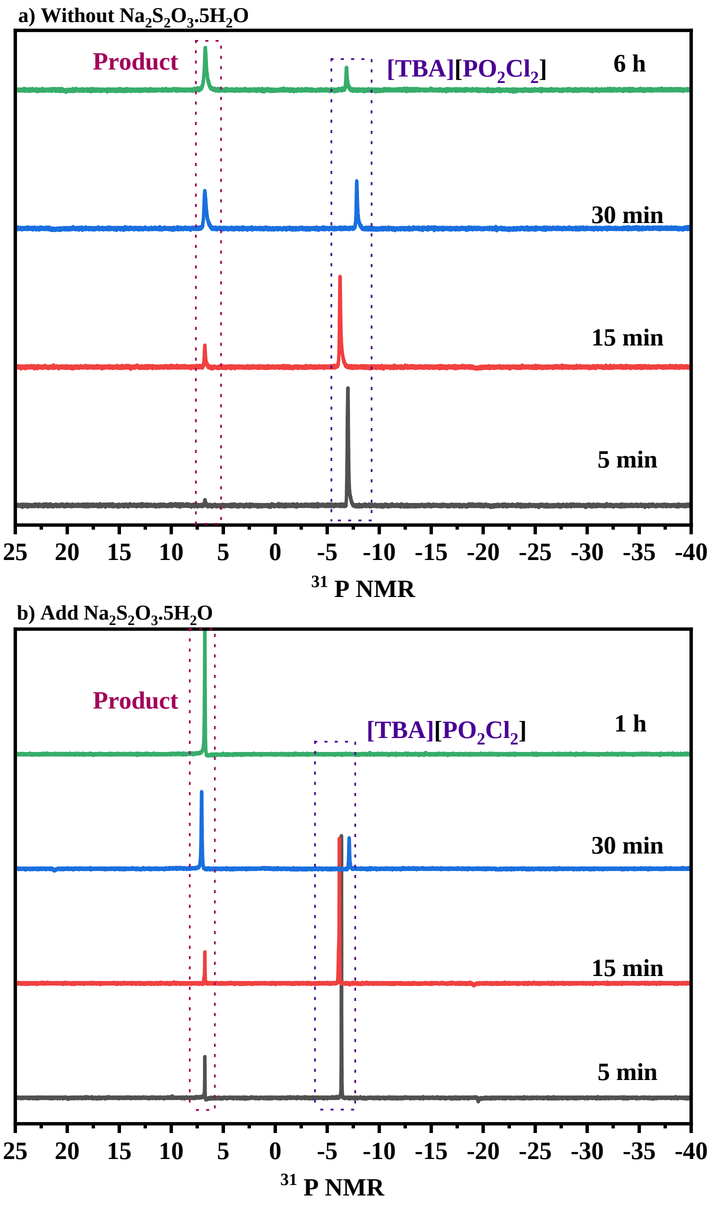

Figure S12 The addition of " $Na_2S_2O_3{\cdot}5H_2O$ " was compared with the ^{31}P NMR of **I-1**.

6 Density Functional Theory (DFT) Calculations

6.1. Computational details

All the calculations were performed using the Gaussian 09 and ORCA[17] programs. All of the structures were fully optimized with the B3LYP[18] method and 6-31+G(d) basis set[19, 20]. The solvation energy corrections were calculated at the M05-2x[5]/6-31G(d) level with the SMD solvation model (acetonitrile) minus electronic energy in the gas phase, respectively. The thermal correction to Gibbs free energy calculated by M06-2x/6-31+G(d) at 298.15 K in gas phase, respectively. The B3LYP/6-31+G(d) calculated imaginary frequencies for the transition states in gas phase. The single point energy was obtained at DLPNO-CCSD(T)/Aug-cc-pvTZ level[21]. Vibrational frequency calculations were performed to ensure that a transition state has only one imaginary frequency and a local minimum has no imaginary frequency. Transition states connecting relevant minima were further examined by running intrinsic reaction coordinate (IRC)[22] calculations.

Table S25 M062x absolute calculation energies (Hartree), energy corrections (Hartree), and imaginary frequency (cm^{-1})

	E(DLPNO CCSD(T)/aug−cc−pvdz)	G(corr−solv)	G−(corr−ther)	Freq(cm^{-1})
DMF	−248.1049622	−0.00739	0.073417	
TCT	−1657.292850	−0.00900	0.001867	
IntD	−1273.293425	−0.01498	0.015661	
HCl	−460.3371430	−0.00309	−0.011220	
$H_2PO_4^-$	−642.8167369	−0.09236	0.007083	
TS1	−1905.364375	−0.03593	0.097503	−94.1
Int0	−1445.421019	−0.09364	0.100250	
TS2	−1905.681616	−0.09544	0.104996	−706.9
Int1	−632.4391922	−0.09376	0.072451	
TS3	−1275.406739	−0.02668	0.101157	−215.8
Int2	−1275.410157	−0.02483	0.099580	
TS4	−1275.398492	−0.03887	0.096516	−151.7
Int3	−1275.461694	−0.03151	0.094345	
TS5	−1275.452206	−0.03336	0.095053	−518.9
Int4	−815.4854246	−0.09436	0.102510	
TS6	−1275.421861	−0.03039	0.101857	−177.1
Int5	−1027.353032	−0.01462	0.004173	
Int6	−1026.827675	−0.08490	−0.005780	

[a]The solvation energy corrections calculated at the M05-2x/6-31G(d) level with the SMD solvation model (acetonitrile) minus electronic energy in gas phase. [b]The thermal correction to Gibbs free energy

calculated by B3LYP/6-31+G* at 298.15K in gas phase. [c]The B3LYP/6-31+G* calculated imaginary frequencies for the transition states in gas phase.

6.2 Free energy profiles for the reaction pathways

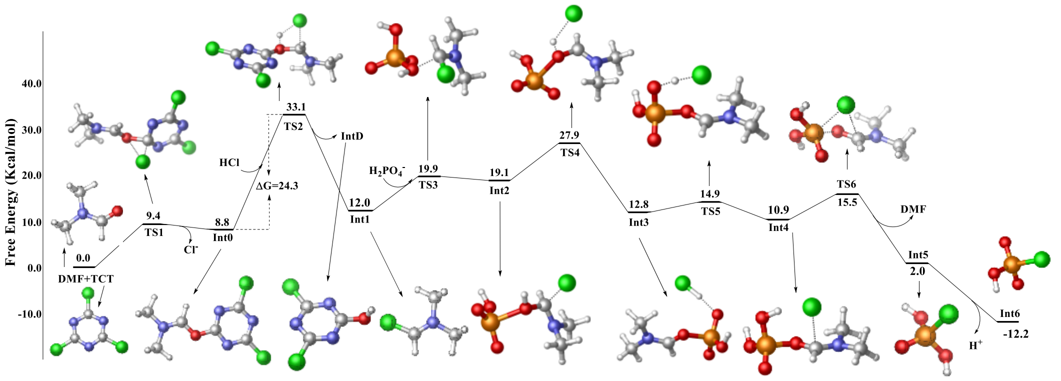

Figure S13 Free energy profiles for the reaction pathways. Computed the favorable free energy profiles for the tentative reaction pathways at the DLPNO-CCSD(T)/Aug-cc-pvTZ//SMD-M05-2x/6-31G(d)//B3LYP/6-31+G* level under 298.15K. The relative free energies are given in kcal/mol.

Density functional theory (DFT) calculations were used to study the reaction mechanisms. The detailed information for the reaction mechanism is shown in Fig. 13. The results were better when using a low catalytic amount of FPyr during the experiment, but to simplify the process, we chose DMF for the theoretical calculation. Firstly, DMF and TCT undergo nucleophilic attack to form **Int0** through transition state **TS1**with an energy barrier of 9.4 kcal/mol. Then the key intermediate Vilsmeier reagent **Int1** can be obtained through the transition state **TS2** from **Int0** in the presence of **Cl⁻**, with an energy barrier of 24.3 kcal/mol. Subsequently, the **Int1** (12.0 kcal/mol) and $H_2PO_4^-$ can generate **Int2** (19.1 kcal/mol) through **TS3** with an energy barrier of 7.9 kcal/mol. Secondly, the formation of the key **Int4** needs to cross the energy barriers of 8.8 and 2.1 kcal/mol through the two processes of (**Int2−TS4−Int3**) and (**Int3−TS5−Int4**), respectively. **Int4** passes through the transition state **TS6** to obtain **Int5**, and the energy barrier is 4.6 kcal/mol. With the elimination of one molecule of DMF, **Int5** is further dehydrogenated to obtain the key monochloro intermediate **Int6**. Finally, the target product **1a** was further chlorinated by the intermediate.

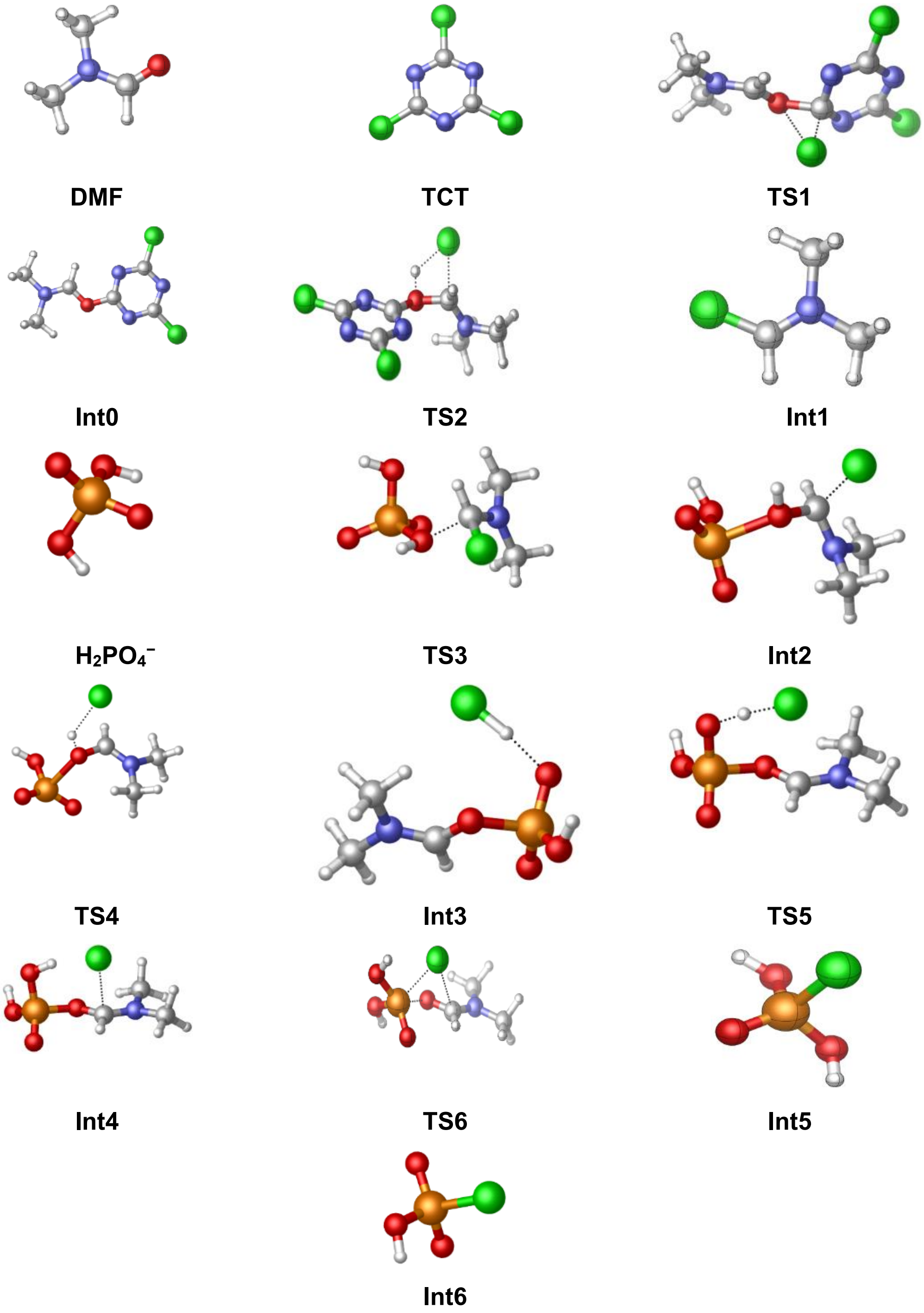

Figure S14 The optimized structures at the B3LYP/6-31+G* level.

7 X-ray Crystallographic Data

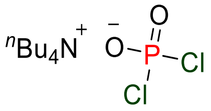

1a

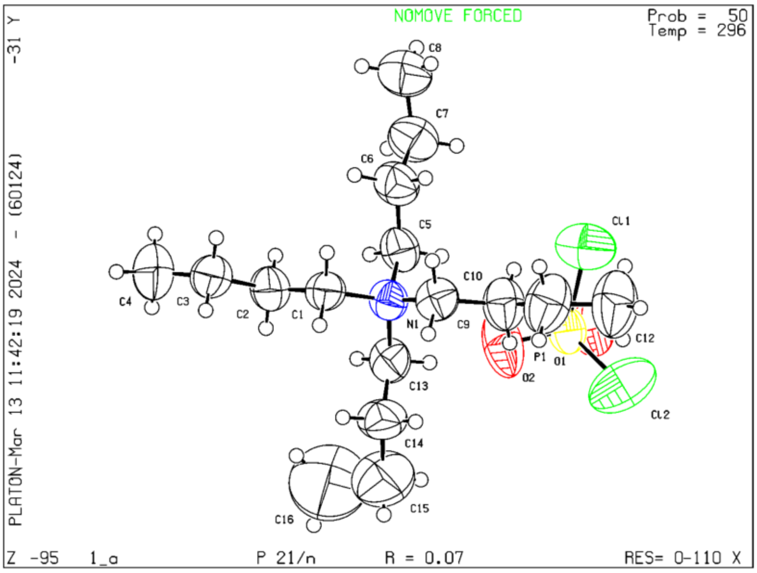

Bond precision: C-C = 0.0074 A Wavelength=0.71073

Cell:	a=10.2286(19)	b=12.961(2)	c=16.698(3)
	alpha=90	beta=92.173(3)	gamma=90
Temperature:	296 K		

	Calculated	Reported
Volume	2212.1(7)	2212.1(7)
Space group	P 21/n	P 21/n
Hall group	-P 2yn	-P 2yn
Moiety formula	C16 H36 N, Cl2 O2 P	?
Sum formula	C16 H36 Cl2 N O2 P	C16 H36 Cl2 N O2 P
Mr	376.33	376.33
Dx,g cm-3	1.130	1.130
Z	4	4
Mu (mm-1)	0.372	0.372
F000	816.0	816.0
F000'	817.84	
h,k,lmax	12,16,20	12,16,20
Nref	4473	4460
Tmin,Tmax	0.915,0.928	0.640,0.745
Tmin'	0.894	

Correction method= # Reported T Limits: Tmin=0.640 Tmax=0.745
AbsCorr = MULTI-SCAN

Data completeness= 0.997 Theta(max)= 26.259

R(reflections)= 0.0738(2535) wR2(reflections)= 0.2322(4460)

S = 1.012 Npar= 203

Figure S15 X-ray Crystallographic Data of **1a** (CCDC 2235755)

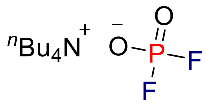

1c

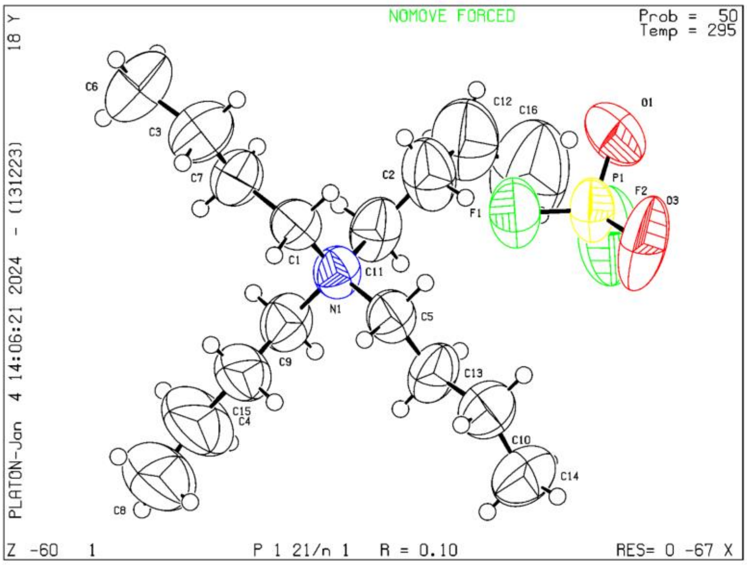

Bond precision: C-C = 0.0095 A Wavelength=0.71073

Cell:	a=9.9975(5)	b=12.8203(6)	c=16.5564(9)
	alpha=90	beta=93.191(2)	gamma=90
Temperature:	295 K		

	Calculated	Reported
Volume	2118.76(19)	2118.76(19)
Space group	P 21/n	P 1 21/n 1
Hall group	-P 2yn	-P 2yn
Moiety formula	C16 H36 N, F2 O2 P	F2 O2 P, C16 H36 N
Sum formula	C16 H36 F2 N O2 P	C16 H36 F2 N O2 P
Mr	343.43	343.43
Dx,g cm-3	1.077	1.077
Z	4	4
Mu (mm-1)	0.152	0.152
F000	752.0	752.0
F000'	752.77	
h,k,lmax	11,15,19	11,15,19
Nref	3728	3712
Tmin,Tmax	0.962,0.989	0.676,0.746
Tmin'	0.961	

Correction method= # Reported T Limits: Tmin=0.676 Tmax=0.746
AbsCorr = MULTI-SCAN

Data completeness= 0.996 Theta(max)= 24.998

R(reflections)= 0.0989(1802) wR2(reflections)= 0.3543(3712)

S = 1.142 Npar= 203

Figure S16 X-ray Crystallographic Data of **1c** (CCDC 2325998)

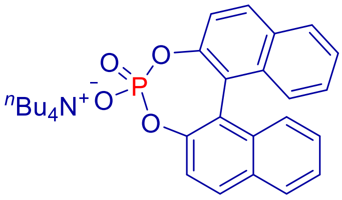

compound I-1

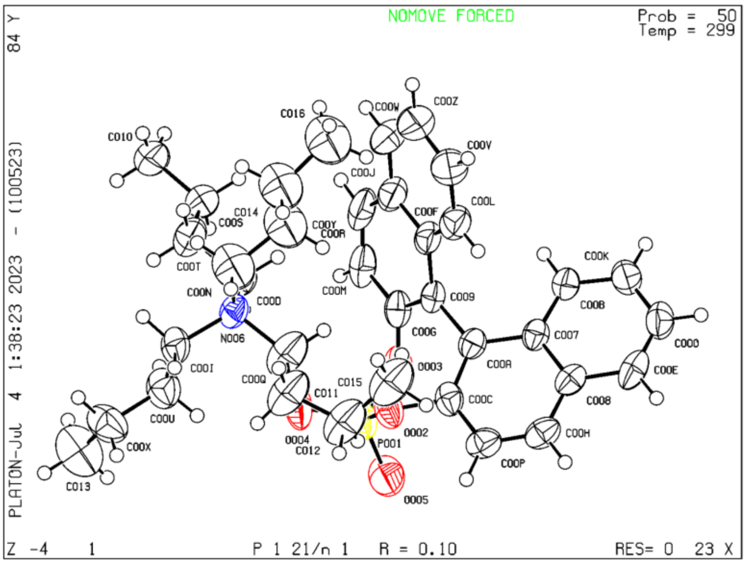

Bond precision: C-C = 0.0091 A Wavelength=0.71073

Cell: a=9.6031(5) b=20.2906(10) c=17.0728(9)
alpha=90 beta=93.087(1) gamma=90

Temperature: 299 K

	Calculated	Reported
Volume	3321.9(3)	3321.9(3)
Space group	P 21/n	P 1 21/n 1
Hall group	-P 2yn	-P 2yn
Moiety formula	C20 H12 O4 P, C16 H36 N	C20 H12 O4 P, C16 H36 N
Sum formula	C36 H48 N O4 P	C36 H48 N O4 P
Mr	589.72	589.72
Dx,g cm-3	1.179	1.179
Z	4	4
Mu (mm-1)	0.121	0.121
F000	1272.0	1272.0
F000'	1272.92	
h,k,lmax	12,25,21	12,25,21
Nref	6775	6660
Tmin,Tmax	0.988,0.994	0.693,0.746
Tmin'	0.964	

Correction method= # Reported T Limits: Tmin=0.693 Tmax=0.746
AbsCorr = MULTI-SCAN

Data completeness= 0.983 Theta(max)= 26.372

R(reflections)= 0.1038(3226) wR2(reflections)= 0.3486(6660)

S = 1.025 Npar= 383

Figure S17 X-ray Crystallographic Data of **I-1** (CCDC 2279841)

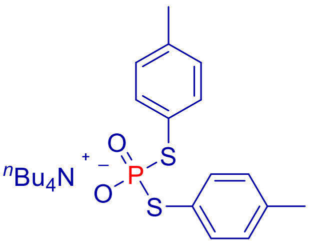

Compound **II-1**

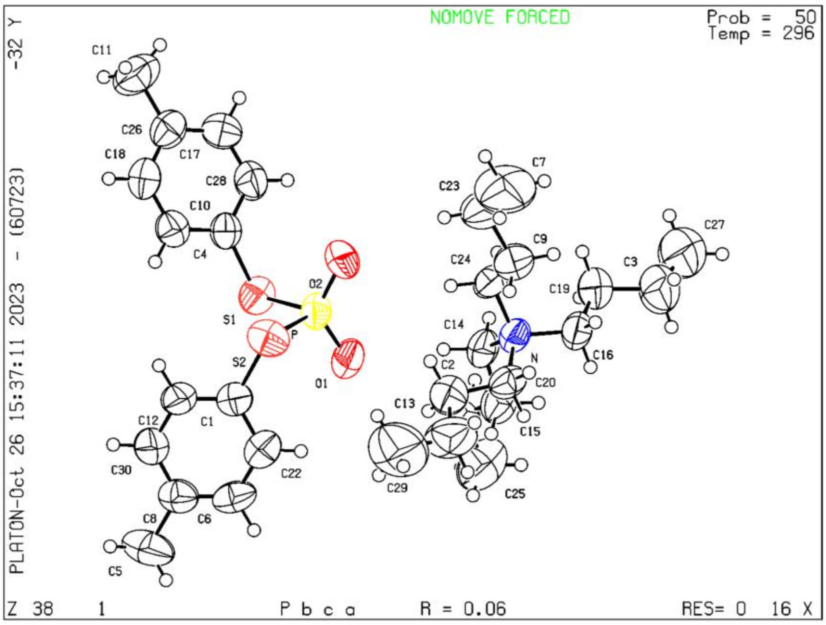

Bond precision: C-C = 0.0063 A Wavelength=0.71073

Cell:	a=16.0288(10)	b=19.0271(11)	c=21.6677(14)
	alpha=90	beta=90	gamma=90
Temperature:	296 K		

	Calculated	Reported
Volume	6608.3(7)	6608.2(7)
Space group	P b c a	P b c a
Hall group	-P 2ac 2ab	-P 2ac 2ab
Moiety formula	C14 H14 O2 P S2, C16 H36 N	C14 H14 O2 P S2, C16 H36 N
Sum formula	C30 H50 N O2 P S2	C30 H50 N O2 P S2
Mr	551.80	551.80
Dx,g cm-3	1.109	1.109
Z	8	8
Mu (mm-1)	0.234	0.234
F000	2400.0	2400.0
F000'	2403.55	
h,k,lmax	20,24,28	20,24,28
Nref	7584	7573
Tmin,Tmax	0.941,0.993	0.704,0.746
Tmin'	0.941	

Correction method= # Reported T Limits: Tmin=0.704 Tmax=0.746
AbsCorr = MULTI-SCAN

Data completeness= 0.999 Theta(max)= 27.499

R(reflections)= 0.0597(3289) wR2(reflections)= 0.1998(7573)

S = 1.007 Npar= 331

Figure S18 X-ray Crystallographic Data of **II-1** (CCDC 2303988)

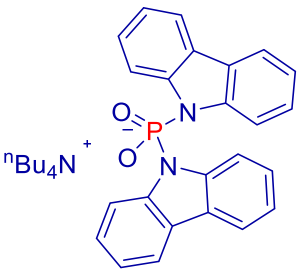

Compound III-1

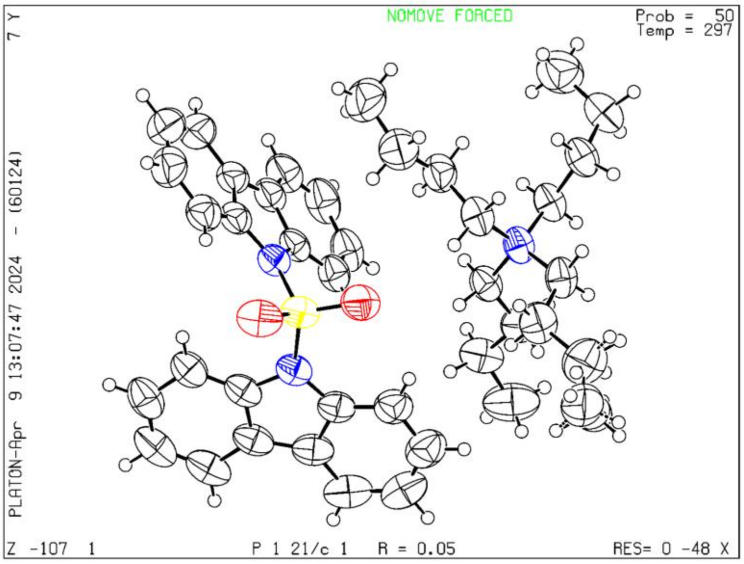


```
Bond precision:   C-C = 0.0033 A          Wavelength=1.54178

Cell:             a=22.4637(5)    b=8.8317(2)       c=18.2807(4)
                  alpha=90        beta=93.987(1)    gamma=90
Temperature:      297 K

                 Calculated                   Reported
Volume           3617.98(14)                  3617.98(14)
Space group      P 21/c                       P 1 21/c 1
Hall group       -P 2ybc                      -P 2ybc
Moiety formula   C24 H16 N2 O2 P, C16 H36 N   C24 H16 N2 O2 P, C16 H36 N
Sum formula      C40 H52 N3 O2 P              C40 H52 N3 O2 P
Mr               637.82                       637.81
Dx,g cm-3        1.171                        1.171
Z                4                            4
Mu (mm-1)        0.955                        0.955
F000             1376.0                       1376.0
F000'            1380.64
h,k,lmax         27,10,22                     27,10,22
Nref             7128                         7001
Tmin,Tmax        0.892,0.909                  0.553,0.754
Tmin'            0.892

Correction method= # Reported T Limits: Tmin=0.553 Tmax=0.754
AbsCorr = MULTI-SCAN

Data completeness= 0.982          Theta(max)= 72.164

                                             wR2(reflections)=
R(reflections)= 0.0480( 5421)                0.1457( 7001)

S = 1.104                 Npar= 430
```

Figure S19 X-ray Crystallographic Data of **III-1** (CCDC 2347060)

8 Characterization Data of the Products

8.1 Copies of NMR spectra about P(V)-X reagents

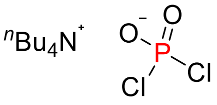

1a

Compound 1a, physical state: white solid; **TLC** R_f = 0.35 (DCM: MeOH = 20:1); **^{1}H NMR** (400 MHz, Chloroform-*d*) δ = 3.23 – 3.17 (m, 8H), 1.64 – 1.54 (m, 8H), 1.42 – 1.33 (m, 8H), 0.92 (t, *J* = 7.2, 12H). **^{31}P NMR** (162 MHz, Chloroform-*d*) δ = -7.23. **^{13}C NMR** (151 MHz, Chloroform-*d*) δ = 58.4, 23.6, 19.4, 13.3.

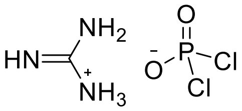

1b

^{31}P NMR (162 MHz, Chloroform-*d*) δ = -4.45.

^{13}C NMR (151 MHz, DMSO-*d*6) δ = 158.7.

Compound 1d, physical state: white solid; **TLC** R_f = 0.28 (DCM:MeOH = 20:1); **^{31}P NMR** (162 MHz, Chloroform-*d*) δ = -4.45. **^{13}C NMR** (151 MHz, DMSO-*d*6) δ = 158.7.

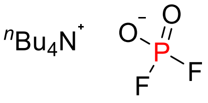

1c

Compound 1c, physical state: white solid; **TLC** R_f = 3.0 (DCM:MeOH = 20:1);

^{1}H NMR (400 MHz, Chloroform-d) δ = 3.25 – 3.18 (m, 8H), 1.66 – 1.56 (m, 8H), 1.44 – 1.36 (m, 8H), 0.96 (t, J=7.3, 12H).

^{31}P NMR (162 MHz, Chloroform-*d*) δ = -7.86 – -24.48 (t, *J*= 957.42).

^{19}F NMR (376 MHz, Chloroform-*d*) δ = -79.33 (d, *J*=948.3).

^{13}C NMR (151 MHz, Chloroform-*d*) δ = 58.5, 23.7, 19.5, 13.4.

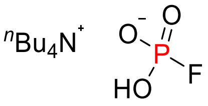

1d

Compound 1c, physical state: white solid; **TLC** R_f = 1.8 (DCM: MeOH = 20:1); **^{1}H NMR** (400 MHz, Chloroform-*d*) δ = 3.12 – 3.01 (m, 8H), 1.40 – 1.25 (m, 8H), 1.18 – 1.07 (m, 8H), 0.65 (t, *J*=7.2, 12H).

^{31}P NMR (162 MHz, Chloroform-*d*) δ = -3.39 (d, *J*=892.6).

^{13}C NMR (151 MHz, Chloroform-*d*) δ = 58.4, 23.6, 19.4, 13.3.

8.2 Copies of NMR spectra about corresponding product

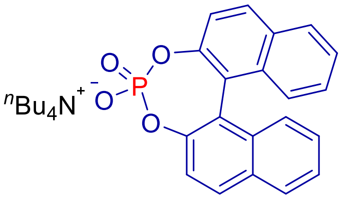

Compound I-1

Physical state: White solid; **TLC:** R_f = 0.44 (DCM/MeOH = 20:1); Yield: 76%

^{1}H NMR (400 MHz, Chloroform-*d*) δ = 7.84 – 7.80 (m, 4H), 7.46 (d, *J*=8.8, 2H), 7.33 – 7.27 (m, 4H), 7.15 (t, *J*=7.2, 2H), 2.86 – 2.82 (m, 8H), 1.26 – 1.21 (m, 8H), 1.14 – 1.09 (m, 8H), 0.76 (t, *J*=7.2, 12H).

^{31}P NMR (162 MHz, Chloroform-*d*) δ = 6.21.

^{13}C NMR (101 MHz, Chloroform-*d*) δ = 150.4 (d, *J*=9.0), 132.5 (d, *J*=1.0), 130.6 (d, *J*=1.), 129.6, 128.1, 126.8, 125.6, 124.2, 122.8 (d, *J*=3.0), 122.3 (d, *J*=2.0), 57.8, 23.5, 19.4, 13.5.

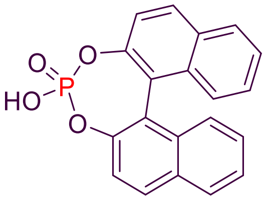

Compound I-1a

Physical state: White solid; **TLC:** R_f = 0.21 (DCM/MeOH = 10:1 ~ 5:1); Yield: 94%[a] /62%[b].(The yields are the reactions step-by-step. [b]The yields are the reactions in one pot.

^{1}H NMR (400 MHz, DMSO-*d*$_6$) δ = 8.00 (t, *J*=8.4, 4H), 7.43 – 7.37 (m, 4H), 7.32 – 7.25 (m, 2H), 7.19 (d, *J*=8.4, 2H).

^{31}P NMR (162 MHz, DMSO-*d*$_6$) δ = 5.25.

^{13}C NMR (151 MHz, DMSO-*d*$_6$) δ = 150.4 (d, *J*=9.0), 132.4, 130.8, 130.2, 128.8, 126.5, 126.4, 124.9, 123.0, 122.1.

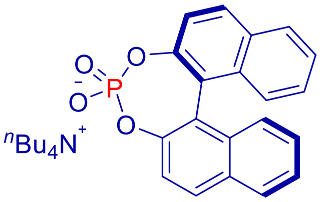

Compound I-2

Physical state: White solid; **TLC:** R_f = 0.44 (DCM/MeOH = 20:1); Yield: 87%.

^{1}H NMR (400 MHz, Chloroform-*d*) δ = 7.85 – 7.77 (m, 4H), 7.45 (d, *J*=8.8, 2H), 7.32 – 7.28 (m, 2H), 7.17 – 7.10 (m, 2H), 2.79 – 2.68 (m, 8H), 1.21 – 1.13 (m, 8H), 1.09 – 1.01 (m, 8H), 0.73 (t, *J*=6.8, 12H).

^{31}P NMR (162 MHz, Chloroform-*d*) δ = 5.93.

^{13}C NMR (151 MHz, Chloroform-*d*) δ = 150.1 (d, *J*=9.0), 132.4, 130.7, 129.7, 128.1, 126.8, 125.6, 124.3, 122.6 (d, *J*=1.5), 122.2 (d, *J*=1.5), 57.5, 23.3, 19.3, 13.4.

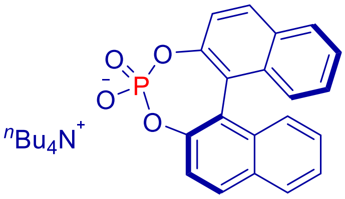

Compound I-3

Physical state: White solid; **TLC:** R_f = 0.44 (DCM/MeOH = 20:1); Yield: 74%.

^{1}H NMR (400 MHz, Chloroform-*d*) δ = 7.75 (d, *J*=8.4, 4H), 7.46 (d, *J*=8.8, 2H), 7.28 – 7.26 (m, 2H), 7.13 (t, *J*=7.6, 2H), 2.71 – 2.55 (m, 8H), 1.18 – 1.02 (m, 8H), 1.02 – 0.90 (m, 8H), 0.67 (t, *J*=7.2, 12H). **^{31}P NMR** (162 MHz, Chloroform-*d*) δ = 6.01.

^{13}C NMR (151 MHz, Chloroform-*d*) δ = 150.3 (d, *J*=9.0), 132.5, 130.6, 129.5, 128.1, 126.8, 125.6, 124.2, 122.7 (d, *J*=3.0), 122.3 (d, *J*=1.5), 57.7, 23.4, 19.3, 13.5.

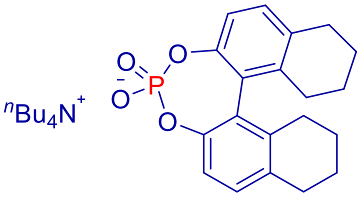

Compound I-4

Physical state: White solid; **TLC:** R_f = 0.34 (DCM/MeOH = 20:1); Yield: 71%.

^{1}H NMR (400 MHz, Chloroform-*d*) δ = 6.97 (s, 4H), 3.23 – 3.16 (m, 8H), 3.00 (s, 2H), 2.82 – 2.62 (m, 6H), 2.27 – 2.17 (m, 2H), 1.76 – 1.69 (m, 6H), 1.58 – 1.48 (m, 9H), 1.38 – 1.31 (m, 8H), 0.93 (t, *J*=7.2, 13H).

^{31}P NMR (162 MHz, Chloroform-*d*) δ = 3.42.

^{13}C NMR (151 MHz, Chloroform-*d*) δ = 149.4 (d, *J*=9.0), 137.1, 132.6 (d, *J*=1.5), 128.9, 127.77, 118.8 (d, *J*=3.0), 58.5, 29.1, 27.8, 23.9 (d, *J*=3.0), 22.8, 22.6, 19.6, 13.6.

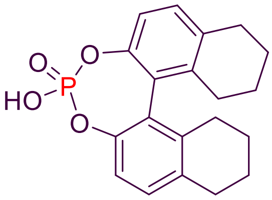

Compound I-4a

Physical state: White solid; **TLC:** R_f = 0.20 (DCM/MeOH = 10:1 ~ 5:1); Yield: 86%

^{1}H NMR (400 MHz, DMSO-*d*$_6$) δ = 7.00 (d, *J*=8.4, 2H), 6.79 (d, *J*=8.4, 2H), 2.79 – 2.56 (m, 6H), 2.11 (d, *J*=16.0, 2H), 1.72 (s, 6H), 1.49 – 1.41 (m, 2H).

^{31}P NMR (162 MHz, DMSO-*d*$_6$) δ = 2.32.

^{13}C NMR (151 MHz, DMSO-*d*$_6$) δ = 149.8 (d, *J*=9.0), 136.8, 132.3, 128.9, 127.9, 119.4 (d, *J*=3.0), 28.9, 27.8, 22.7, 22.5.

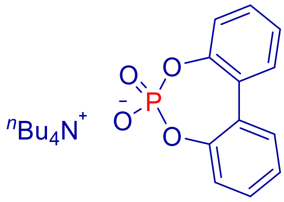

Compound I-5

Physical state: White solid; **TLC:** R_f = 0.34 (DCM/MeOH = 20:1); Yield: 70%.

^{1}H NMR (400 MHz, Chloroform-*d*) δ = 7.39 (d, *J*=7.6, 2H), 7.25 – 7.15 (m, 4H), 7.11 (t, *J*=7.2, 2H), 3.07 – 3.01 (m, 8H), 1.44 – 1.36 (m, 8H), 1.24 – 1.17 (m, 8H), 0.84 (t, *J*=7.2, 12H).

^{31}P NMR (162 MHz, Chloroform-*d*) δ = 5.46.

^{13}C NMR (101 MHz, Chloroform-*d*) **δ =** 151.2, 151.1, 130.3, 128.8, 123.6, 122.1, 58.2, 23.7, 19.5, 13.6.

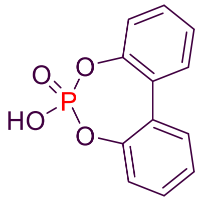

Compound I-5a

Physical state: White solid; **TLC:** R_f = 0.23 (DCM/MeOH = 10:1 ~ 5:1); Yield: 87%.

^{1}H NMR (400 MHz, DMSO-*d*6) δ = 7.48 (d, *J*=8.0, 2H), 7.35 (t, *J*=7.6, 2H), 7.20 (t, *J*=7.6, 2H), 7.06 (d, *J*=8.4, 2H).

^{31}P NMR (162 MHz, DMSO-*d*6) δ = 4.52.

^{13}C NMR (151 MHz, DMSO-*d*6) δ = 151.3, 151.2, 130.4, 129.3, 124.1, 122.2 (d, *J*=4.5).

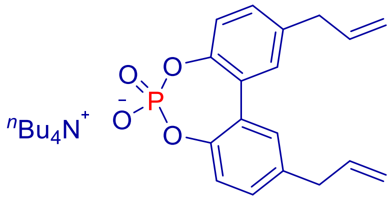

Compound I-6

Physical state: White solid; **TLC:** R_f = 0.18 (DCM/MeOH = 20:1); Yield: 73%.

^{1}H NMR (400 MHz, Chloroform-*d*) δ = 7.20 (s, 2H), 7.13 – 7.04 (m, 4H), 5.99 – 5.86 (m, 2H), 5.11 – 4.99 (m, 4H), 3.36 (d, *J*=6.8, 4H), 3.16 – 3.10 (m, 8H), 1.51 – 1.44 (m, 8H), 1.30 – 1.24 (m, 8H), 0.88 (t, *J*=7.2, 12H).

^{31}P NMR (162 MHz, Chloroform-*d*) δ = 5.59.

^{13}C NMR (101 MHz, Chloroform-*d*) δ = 149.5 (d, *J*=7.0), 149.5, 137.6, 135.1, 130.1, 128.9, 128.8, 121.9, 115.5, 58.3, 39.6, 23.8, 19.5, 13.6.

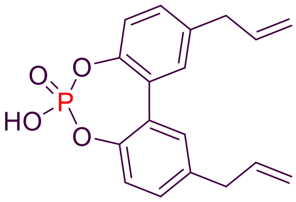

Compound I-6a

Physical state: White solid; **TLC:** R_f = 0.10 (DCM/MeOH = 10:1 ~ 5:1); Yield: 84%.

^{1}H NMR (400 MHz, DMSO-d_6) δ = 7.25 (s, 2H), 7.14 (d, *J*=6.0, 2H), 6.96 (d, *J*=8.0, 2H), 6.03 – 5.92 (m, 2H), 5.13 – 5.01 (m, 4H), 3.39 (d, *J*=6.8, 4H).

^{31}P NMR (162 MHz, DMSO-d_6) δ = 4.49.

^{13}C NMR (151 MHz, DMSO-d_6) δ = 149.6 (d, *J*=9.0), 138.3, 135.4, 130.2, 129., 129.1, 122.0, 116.1, 39.3.

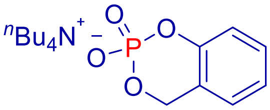

Compound I-7

Physical state: White solid; **TLC:** R_f = 0.23 (DCM/MeOH = 10:1 ~5:1); Yield: 76%.

^{1}H NMR (400 MHz, Chloroform-*d*) δ = 6.98 (t, *J*=7.6, 1H), 6.81 (d, *J*=7.6, 1H), 6.75 (d, *J*=7.6, 1H), 6.67 (d, *J*=8.0, 1H), 5.06 (d, *J*=12.4, 2H), 3.16 – 3.11 (m, 8H), 1.49 – 1.44 (m, 8H), 1.29 – 1.25 (m, 8H), 0.83 (t, *J*=7.2, 12H).

^{31}P NMR (162 MHz, Chloroform-*d*) δ = -7.78.

^{13}C NMR (151 MHz, Chloroform-*d*) δ = 153.0 (d, *J*=6.0), 127.7, 124.8, 122.7, 120.7, 118.4 (d, *J*=8.0), 66.7 (d, *J*=6.0), 58.6, 23.9, 19.6, 13.6.

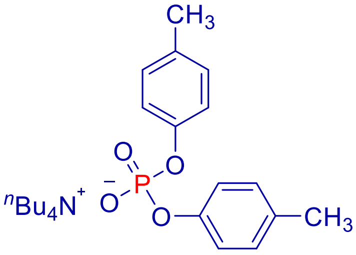

Compound I-8

Physical state: White solid; **TLC:** R_f = 0.32 (DCM/MeOH = 20:1); Yield: 65%.

^{1}H NMR (400 MHz, Chloroform-*d*) δ = 7.12 (d, *J*=8.0, 4H), 6.93 (d, *J*=8.1, 4H), 3.16 – 3.10 (m, 8H), 2.20 (s, 6H), 1.52 – 1.44 (m, 8H), 1.30 (q, *J*=7.4, 8H), 0.89 (t, *J*=7.3, 12H).

^{31}P NMR (162 MHz, Chloroform-*d*) δ = -10.24.

^{13}C NMR (151 MHz, Chloroform-*d*) δ = 151.61, 131.11, 129.25, 120.06 (d, *J*=5.1), 58.56, 23.94, 20.60, 19.64, 13.64.

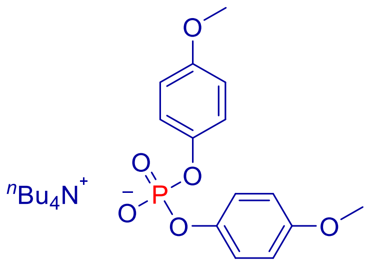

Compound I-9

Physical state: White solid; **TLC:** R_f = 0.33 (DCM/MeOH = 20:1); Yield: 73%.

^{1}H NMR (400 MHz, Chloroform-*d*) δ = 7.14 (d, *J*=8.8, 4H), 6.70 (d, *J*=8.4, 4H), 3.69 (s, 6H), 3.19 – 3.14 (m, 8H), 1.56 – 1.48 (m, 8H), 1.37 – 1.29 (m, 8H), 0.91 (t, *J*=7.2, 12H).

^{31}P NMR (162 MHz, Chloroform-*d*) δ = -9.80.

^{13}C NMR (151 MHz, Chloroform-*d*) δ = 154.7, 147.6 (d, *J*=7.5), 121.0 (d, *J*=4.5), 113.9, 58.5, 55.5, 23.8, 19.6, 13.6.

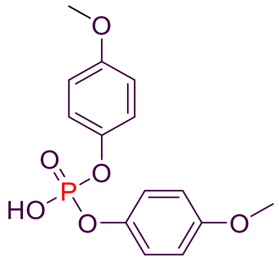

Compound I-9a

Physical state: White solid; **TLC:** R_f = 0.13 (DCM/MeOH = 10:1 ~ 5:1); Yield: 81%.

^{1}H NMR (400 MHz, DMSO-*d*6) δ = 7.02 (d, *J*=8.8, 4H), 6.79 –6.77 (m, 4H), 3.67 (s, 6H).

^{31}P NMR (162 MHz, DMSO-*d*6) δ = -10.89.

^{13}C NMR (151 MHz, DMSO-*d*6) δ = 154.9, 147.4, 121.0 (d, *J*=4.5), 114.4, 55.7.

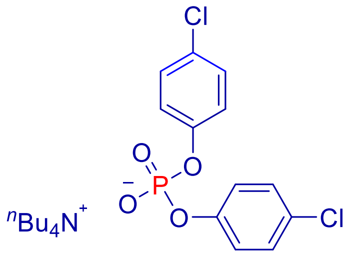

Compound I-10

Physical state: White solid; **TLC:** R_f = 0.25 (DCM/MeOH = 20:1); Yield: 77%.

^{1}H NMR (400 MHz, Chloroform-*d*) δ = 7.14 (d, *J*=8.4, 4H), 7.06 (d, *J*=6.0, 4H), 3.18 – 3.13 (m, 8H), 1.56 – 1.47 (m, 8H), 1.36 – 1.27 (m, 8H), 0.91 (t, *J*=7.2, 12H).

^{31}P NMR (162 MHz, Chloroform-*d*) δ = -10.85.

^{13}C NMR (151 MHz, Chloroform-*d*) δ = 152.3, 128.7, 127.1, 121.5 (d, *J*=4.5), 58.6, 23.8, 19.6, 13.5.

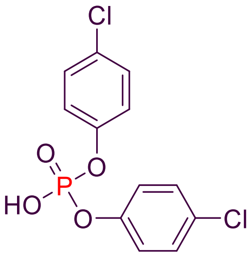

Compound I-10a

Physical state: White solid **TLC:** R_f = 0.15 (DCM/MeOH = 10:1 ~ 5:1); Yield: 89%

^{1}H NMR (400 MHz, DMSO-d_6) δ = 7.25 (d, *J*=8.8, 4H), 7.12 (d, *J*=9.2, 4H).

^{31}P NMR (162 MHz, DMSO-d_6) δ = -11.66.

^{13}C NMR (151 MHz, DMSO-d_6) δ = 153.0 (d, *J*=6.0), 129.1, 126.2, 121.9 (d, *J*=4.5).

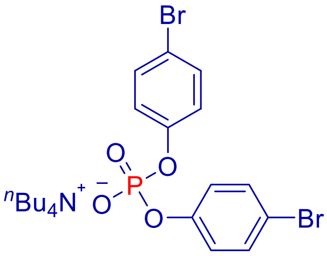

Compound I-11

Physical state: White solid; **TLC:** R_f = 0.27 (DCM/MeOH = 20:1); Yield: 82%.

^{1}H NMR (400 MHz, Chloroform-*d*) δ = 7.25 (d, *J*=9.2, 4H), 7.10 (d, *J*=8.4, 4H), 3.16 –3.07 (m, 8H), 1.56 – 1.47 (m, 8H), 1.35 – 1.27 (m, 8H), 0.92 (t, *J*=72, 12H).

^{31}P NMR (162 MHz, Chloroform-*d*) δ = -11.07.

^{13}C NMR (151 MHz, Chloroform-*d*) δ = 152.8 (d, *J*=3.0), 131.7, 122.0 (d, *J*=6.0), 114.8, 58.5, 23.8, 19.6, 13.6.

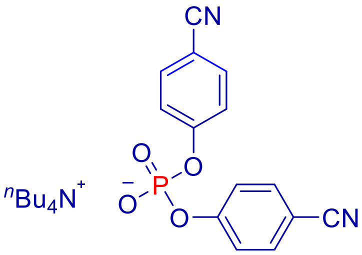

Compound I-12

Physical state: White solid; **TLC:** R_f = 0.16 (DCM/MeOH = 20:1); Yield: 89%.

^{1}H NMR (400 MHz, Chloroform-*d*) δ = 7.37 (d, *J*=8.4, 4H), 7.28 (d, *J*=8.8, 4H), 3.18 – 3.13 (m, 8H), 1.58 – 1.50 (m, 8H), 1.36 – 1.26 (m, 8H), 0.88 (t, *J*=7.2, 12H).

^{31}P NMR (162 MHz, Chloroforcm-*d*) δ = -12.41.

^{13}C NMR (151 MHz, Chloroform-*d*) δ = 157.1, 133.3, 120.8 (d, *J*=6.0), 119.1, 105.4, 58.7, 23.8, 19.6, 13.5.

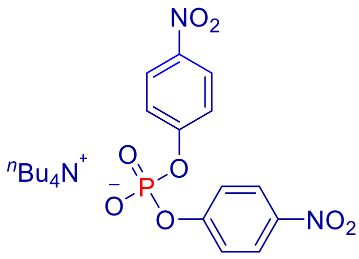

Compound I-13

Physical state: White solid; **TLC:** R_f = 0.23 (DCM/MeOH = 20:1); Yield: 69%.

^{1}H NMR (400 MHz, Chloroform-*d*) δ = 8.06 (d, *J*=8.4, 4H), 7.39 (d, *J*=8.8, 4H), 3.25 –3.21 (m, 8H), 1.65 – 1.57 (m, 8H), 1.39 – 1.33 (m, 8H), 0.93 (t, *J*=7.2, 12H).

^{31}P NMR (162 MHz, Chloroform-*d*) δ = -12.68.

^{13}C NMR (151 MHz, Chloroform-*d*) δ = 159.1 (d, *J*=7.5), 142.5, 125.1, 120.1 (d, *J*=6.0), 58.8, 23.9, 19.6, 13.5.

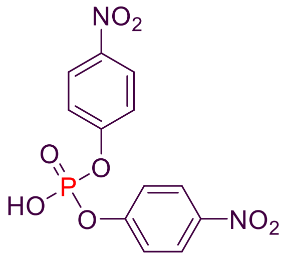

Compound I-13a

Physical state: White solid; **TLC:** R_f = 0.11 (DCM/MeOH = 10:1 ~ 5:1); Yield: 86%.

^{1}H NMR (400 MHz, DMSO-*d*6) δ = 8.15 (d, *J*=9.2, 4H), 7.37 (d, *J*=9.2, 4H).

^{31}P NMR (162 MHz, DMSO-*d*6) δ = -13.15.

^{13}C NMR (151 MHz, DMSO-*d*6) δ = 159.6, 142.4, 125.7, 120.6 (d, *J*=6.0).

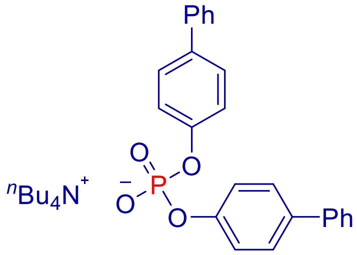

Compound I-14

Physical state: White solid; **TLC:** R_f = 0.34 (DCM/MeOH = 20:1); Yield: 86%.

^{1}H NMR (400 MHz, Chloroform-*d*) δ = 7.48 (d, *J*=8.4, 4H), 7.40 (d, *J*=8.4, 4H), 7.35 (t, *J*=5.6, 4H), 7.27 – 7.22 (m, 2H), 3.29 (t, 11H), 1.92 – 1.54 (m, 12H), 1.39 (h, *J*=7.4, 14H), 0.95 (t, *J*=7.3, 21H).

^{31}P NMR (162 MHz, Chloroform-*d*) δ = -10.51.

^{13}C NMR (151 MHz, Chloroform-*d*) δ = 141.06, 134.97, 128.58, 127.53, 126.70, 126.53, 120.56, 120.53, 58.79, 24.05, 19.69, 13.65.

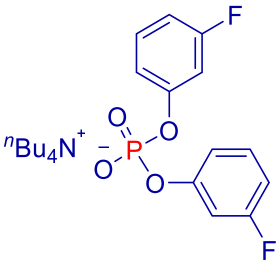 **Compound I-15**

Physical state: White solid; **TLC:** R_f = 0.45 (DCM/MeOH = 20:1); Yield: 85%.

^{1}H NMR (400 MHz, Chloroform-*d*) δ = 7.14 – 7.07 (m, 2H), 7.04 – 6.97 (m, 4H), 6.64 (t, *J*=8.0, 2H), 3.20 – 3.15 (m, 8H), 1.55 – 1.51 (m, 8H), 1.35 – 1.30 (m, 8H), 0.90 (t, *J*=6.8, 12H).

^{31}P NMR (162 MHz, Chloroform-*d*) δ = -11.50.

^{19}F NMR (376 MHz, Chloroform-*d*) δ = -112.65 – -112.80 (m).

^{13}C NMR (151 MHz, Chloroform-*d*) δ = 162.9 (d, *J*=243), 157.5 – 151.4 (m), 129.4 (d, *J*=9.0), 116.0 (dd, *J*=4.5, 3.0), 109.1 (d, *J*=21.0), 107.8 (dd, *J*=24.0, 6.0), 58.5, 23.8, 19.6, 13.5.

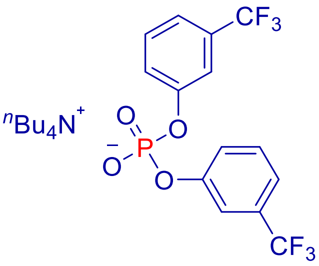 **Compound I-16**

Physical state: White solid; **TLC:** R_f = 0.47 (DCM/MeOH = 20:1); Yield: 74%.

^{1}H NMR (400 MHz, Chloroform-*d*) δ = 7.42 (d, *J*=10.0, 4H), 7.23 (t, *J*=7.6, 2H), 7.15 (d, *J*=8.0, 2H), 3.18 – 3.10 (m, 8H), 1.57 – 1.44 (m, 8H), 1.33 – 1.22 (m, 8H), 0.86 (t, *J*=7.2, 12H).

^{31}P NMR (162 MHz, Chloroform-*d*) δ = -11.32.

^{19}F NMR (376 MHz, Chloroform-*d*) δ = -62.55.

^{13}C NMR (151 MHz, Chloroform-*d*) δ = 153.7 (d, *J*=7.5), 135.2 – 129.3 (m), 126.7 – 120.8 (m), 123.7 (d, *J*=4.5), 119.1 – 119.0 (m), 117.1 (t, *J*=4.5), 58.5, 23.7, 19.51, 13.3.

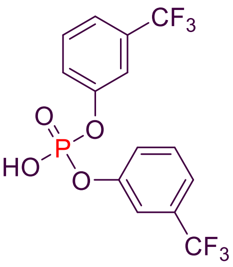 **Compound I-16a**

Physical state: White solid; **TLC:** R_f = 0.33 (DCM/MeOH = 10:1 ~ 5:1); Yield: 77%.

^{1}H NMR (400 MHz, DMSO-*d*6) δ = 7.46 (d, *J*=8.4, 4H), 7.37 – 7.27 (m, 4H).

^{31}P NMR (162 MHz, DMSO-*d*6) δ = -11.99.

^{19}F NMR (376 MHz, DMSO-d_6) δ = -61.19.

^{13}C NMR (151 MHz, DMSO-d_6) δ = 154.4 (d, *J*=7.5), 130.6, 130.1 (d, *J*=31.5), 129.1, 126.3 – 122.7 (m), 119.0 (d, *J*=4.5), 116.7 (t, *J*=3.0).

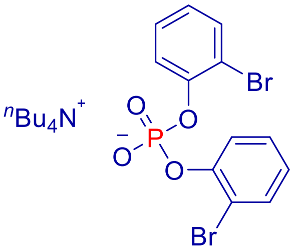

Compound I-17

Physical state: White solid; **TLC:** R_f = 0.47 (DCM/MeOH = 20:1); Yield: 74%.

^{1}H NMR (400 MHz, Chloroform-*d*) δ = 7.78 – 7.75 (m, 2H), 7.41 – 7.26 (m, 2H), 7.14 – 7.10 (m, 2H), 6.80 – 6.76 (m, 2H), 3.20 – 3.14 (m, 8H), 1.54 – 1.50 (m, 8H), 1.31 – 1.28 (m, 8H), 0.88 (t, *J*=7.2, 12H).

^{31}P NMR (162 MHz, Chloroform-*d*) δ = -11.83.

^{13}C NMR (151 MHz, Chloroform-*d*) δ = 150.7 (d, *J*=3.0), 132.5, 128.0, 123.1, 121.4, 114.1 (d, *J*=10.5), 58.5, 23.8, 19.6, 13.6.

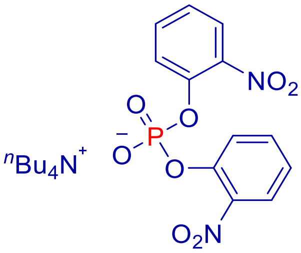

Compound I-18

Physical state: White solid; **TLC:** R_f = 0.38 (DCM/MeOH = 20:1); Yield: 78%.

^{1}H NMR (400 MHz, Chloroform-*d*) δ = 7.84 (d, *J*=8.4, 2H), 7.70 (d, *J*=8.0, 2H), 7.46 – 7.40 (m, 2H), 7.03 (t, *J*=6.8, 2H), 3.26 – 3.21 (m, 8H), 1.62 – 1.57 (m, 8H), 1.38 – 1.33 (m, 8H), 0.93 (t, *J*=7.2, 12H).

^{31}P NMR (162 MHz, Chloroform-*d*) δ = -12.76.

^{13}C NMR (101 MHz, Chloroform-*d*) δ = 133.7, 124.5, 122.1, 58.7, 23.9, 19.6, 13.6.

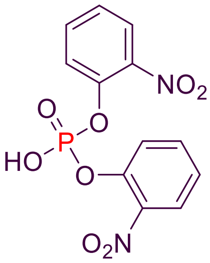

Compound I-18a

Physical state: White solid; **TLC:** R_f = 0.13 (DCM/MeOH = 10:1 ~ 5:1); Yield: 57%.

¹H NMR (400 MHz, DMSO-d_6) δ = 7.76 (d, *J*=8.0, 2H), 7.63 – 7.59 (m, 2H), 7.57 – 7.52 (m, 2H), 7.15 (t, *J*=7.6, 2H).

³¹P NMR (162 MHz, DMSO-d_6) δ = -13.03.

¹³C NMR (151 MHz, DMSO-d_6) δ = 146.4, 133.7, 130.0, 124.8, 124.6, 122.6 (d, *J*=3.0).

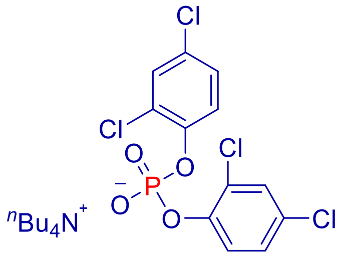

Compound I-19

Physical state: White solid; **TLC:** R_f = 0.40 (DCM/MeOH = 20:1); Yield: 75%.

¹H NMR (400 MHz, Chloroform-*d*) δ = 7.69 (d, *J*=8.8, 2H), 7.24 (d, *J*=2.4, 2H), 7.05 (dd, *J*=8.8, 2.8, 2H), 3.18 – 3.15 (m, 8H), 1.56 – 1.52 (m, 8H), 1.33 – 1.29 (m, 8H), 0.90 (t, *J*=7.2, 12H).

³¹P NMR (162 MHz, Chloroform-*d*) δ = -11.75.

¹³C NMR (151 MHz, Chloroform-*d*) δ = 148.6 (d, *J*=6.0), 129.1, 127.3, 127.1, 125.4 (d, *J*=7.5), 122.3 (d, *J*=3.0), 58.6, 23.8, 19.6, 13.5.

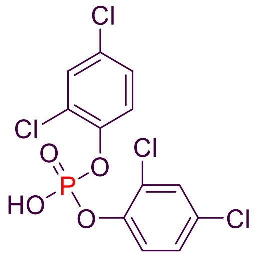

Compound I-19a

Physical state: White solid; **TLC:** R_f = 0.25 (DCM/MeOH = 10:1 ~ 5:1); Yield: 85%.

¹H NMR (400 MHz, DMSO-d_6) δ = 7.64 (d, *J*=9.2, 2H), 7.49 (d, *J*=2.0, 2H), 7.30 – 7.27 (m, 2H).

³¹P NMR (162 MHz, DMSO-d_6) δ = -12.44.

¹³C NMR (151 MHz, DMSO-d_6) δ = 149.1 (d, *J*=6.0), 129.2, 127.9, 126.5, 125.1 (d, *J*=7.5), 122.7 (d, *J*=1.5).

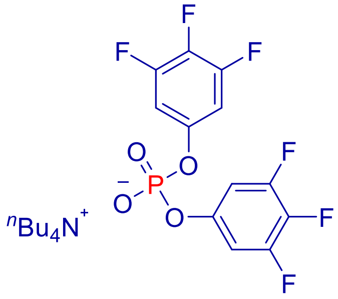

Compound I-20

Physical state: White solid; **TLC:** R_f = 0.35 (DCM/MeOH = 20:1); Yield: 92%.

^{1}**H NMR** (400 MHz, Chloroform-*d*) δ = 6.91 – 6.86 (m, 4H), 3.19 – 3.15 (m, 8H), 1.55 (m, 8H), 1.40 – 1.26 (m, 8H), 0.90 (t, *J*=7.2, 12H).

^{31}**P NMR** (162 MHz, Chloroform-*d*) δ = -11.85.

^{19}**F NMR** (376 MHz, Chloroform-*d*) δ = -135.08 – -135.16 (m), -169.60 – -169.74 (m).

^{13}**C NMR** (151 MHz, Chloroform-*d*) δ = 151.7 – 151.3 (m), 150.6 – 149.5 (m), 148.7, 136.7 (t, *J*=15.0), 135.0 (t, *J*=15.0), 110.8 – 103.1 (m), 58.6, 23.8, 19.5, 13.4.

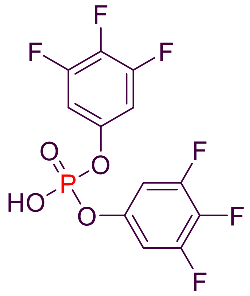

Compound I-20a

Physical state: White solid; **TLC:** R_f = 0.19 (DCM/MeOH = 10:1 ~ 5:1); Yield: 77%.

^{1}**H NMR** (400 MHz, DMSO-d_6) δ = 7.10 (dd, *J*=10.4, 6.4, 4H).

^{31}**P NMR** (162 MHz, DMSO-d_6) δ = -12.44.

^{19}**F NMR** (376 MHz, DMSO-d_6) δ = -135.40 – -135.53 (m), -170.16.

^{13}**C NMR** (151 MHz, DMSO-d_6) δ = 182.8 – 143.1 (m), 140.2 – 128.9 (m), 105.2 (t, *J*=4.5), 105.1 (t, *J*=4.5).

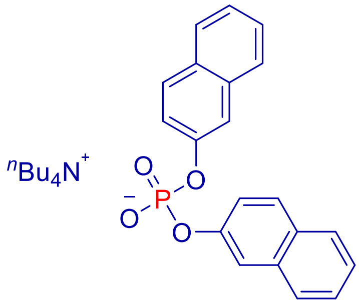

Compound I-21

Physical state: White solid; **TLC:**R_f = 0.48 (DCM/MeOH = 20:1); Yield: 61%.

^{1}**H NMR** (400 MHz, Chloroform-*d*) δ = 7.78 (s, 2H), 7.70 – 7.61 (m, 6H), 7.42 (dd, *J*=9.2, 2.4, 2H), 7.33 (t, *J*=7.6, 2H), 7.27 (t, *J*=6.8, 2H), 2.99 – 2.94 (m, 8H), 1.38 – 1.30 (m, 8H), 1.24 – 1.18 (m, 8H), 0.83 (t, *J*=7.2, 12H).

^{31}**P NMR** (162 MHz, Chloroform-*d*) δ = -10.33.

^{13}**C NMR** (151 MHz, Chloroform-*d*) δ = 151.8 (d, *J*=7.5), 134.3, 129.7, 128.4, 127.3, 127.2, 125.6, 123.8, 121.8 (d, *J*=6.0), 115.7 (d, *J*=4.5), 58.2, 23.7, 19.5, 13.5.

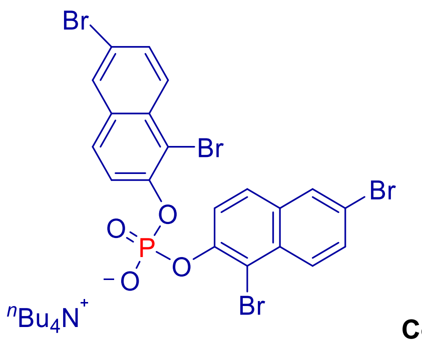

Compound I-22

Physical state: White solid; **TLC:** R_f = 0.47 (DCM/MeOH = 20:1); Yield: 59%.

^{1}H NMR (400 MHz, Chloroform-*d*) δ = 8.11 (d, *J*=9.2, 2H), 8.00 (d, *J*=9.2, 2H), 7.86 (s, 2H), 7.56 (d, *J*=8.8, 2H), 7.50 (dd, *J*=9.2, 2.0, 2H), 3.11 – 3.06 (m, 8H), 1.47 – 1.42 (m, 8H), 1.23 – 1.19 (m, 8H), 0.80 (t, *J*=7.2, 12H).

^{31}P NMR (162 MHz, Chloroform-*d*) δ = -7.38.

^{13}C NMR (151 MHz, Chloroform-*d*) δ = 149.7 (d, *J*=6.0), 131.5 (d, *J*=4.5), 130.1, 129.6, 128.4, 127.0, 122.8, 118.4, 111.6, 111.6, 58.5, 23.8, 19.6, 13.6.

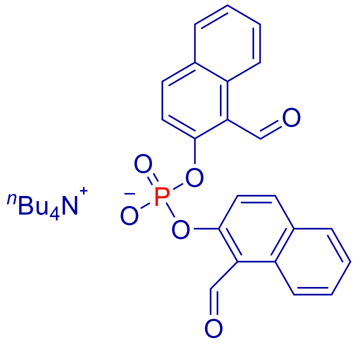

Compound I-23

Physical state: White solid; **TLC:** R_f = 0.32 (DCM/MeOH = 20:1); Yield: 62%.

^{1}H NMR (400 MHz, Chloroform-*d*) δ = 10.96 (s, 2H), 9.26 (d, *J*=8.8, 2H), 7.97 (d, *J*=8.8, 2H), 7.87 (d, *J*=9.2, 2H), 7.77 (d, *J*=8.0, 2H), 7.60 – 7.55 (m, 2H), 7.45 – 7.40 (m, 2H), 3.16 – 3.11 (m, 8H), 1.55 – 1.50 (m, 8H), 1.31 – 1.27 (m, 8H), 0.90 (t, *J*=7.2, 12H).

^{31}P NMR (162 MHz, Chloroform-*d*) δ = -11.29.

^{13}C NMR (151 MHz, Chloroform-*d*) δ = 193.2, 160.9, 136.5, 131.2, 130.0, 128.9, 128.1, 125.1, 125.1, 121.5, 58.6, 23.8, 19.6, 13.5.

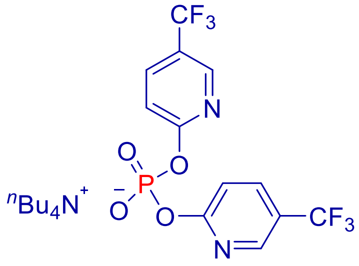

Compound I-24

Physical state: Yellow solid; **TLC:** R_f = 0.28 (DCM/MeOH = 20:1); Yield: 66%.

^{1}H NMR (400 MHz, Chloroform-*d*) δ = 8.43 (s, 2H), 7.82 – 7.80 (m, 2H), 7.52 (d, *J*=8.8, 2H), 3.31 – 3.26 (m, 8H), 1.68 – 1.42 (m, 8H), 1.39 – 1.34 (m, 8H), 0.94 (t, 12H).

^{31}P NMR (162 MHz, Chloroform-*d*) δ = -14.44.

^{13}C NMR (151 MHz, Chloroform-*d*) δ = 162.3, 145.2, 145.2, 136.2, 136.2, 121.7, 114.0, 114.0, 58.7, 23.9, 19.6, 13.5.

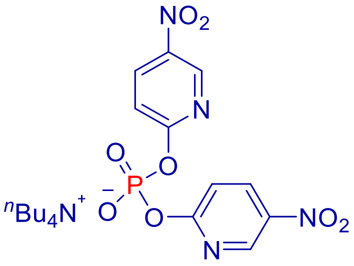

Compound I-25

Physical state: Yellow solid; **TLC:** R_f = 0.11 (DCM/MeOH = 20:1); Yield: 78%.

^{1}H NMR (400 MHz, Chloroform-*d*) δ = 9.09 (d, *J*=3.2, 2H), 8.36 (dd, *J*=9.2, 2.8, 2H), 7.57 (d, *J*=9.2, 2H), 3.32 – 3.31 (m, 8H), 1.67 – 1.65 (m, 8H), 1.43 – 1.42 (m, 8H), 0.99 (t, *J*=5.2, 12H).

^{31}P NMR (162 MHz, Chloroform-*d*) δ = -15.25.

^{13}C NMR (151 MHz, Chloroform-*d*) δ = 141.0, 128.5, 127.5, 126.7, 120.5 (d, *J*=4.5), 58.7, 24.0, 19.6, 13.6.

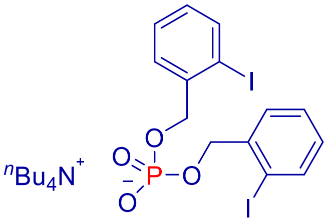

Compound I-26

Physical state: White solid; **TLC:** R_f = 0.46 (DCM/MeOH = 20:1); Yield: 63%.

^{1}H NMR (400 MHz, Chloroform-*d*) δ = 7.63 (t, *J*=8.8, 4H), 7.20 (t, *J*=7.2, 2H), 6.85 – 6.79 (m, 2H), 4.85 (d, *J*=6.4, 4H), 3.23 – 3.19 (m, 8H), 1.56 – 1.51 (m, 8H), 1.34 – 1.30 (m, 8H), 0.88 (t, *J*=8.3, 12H).

^{31}P NMR (162 MHz, Chloroform-*d*) δ = -0.39.

^{13}C NMR (151 MHz, Chloroform-*d*) δ = 141.9 (d, *J*=9.0), 138.2, 128.4, 128.2, 127.7, 96.1, 70.7 (d, *J*=4.5), 58.5, 23.8, 19.6, 13.6.

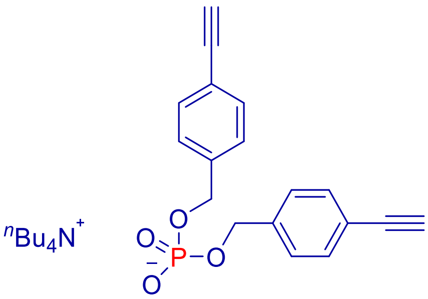

Compound I-27

Physical state: White solid; **TLC:** R_f = 0.44 (DCM/MeOH = 20:1); Yield: 65%.

^{1}H NMR (400 MHz, Chloroform-*d*) δ = 7.36 (d, *J*=7.6, 4H), 7.30 (d, *J*=8.0, 4H), 4.88 (d, *J*=6.4, 4H), 3.24 – 3.22 (m, 8H), 3.02 (s, 2H), 1.58 – 1.55 (m, 8H), 1.39 – 1.36 (m, 8H), 0.93 (t, *J*=6.8, 12H).

^{31}P NMR (162 MHz, Chloroform-*d*) δ = -0.08.

^{13}C NMR (151 MHz, DMSO-*d*6) δ = 141.1, 131.8, 127.4, 120.6, 83.9, 80.8, 65.8, 57.9, 23.5, 19.6, 13.93

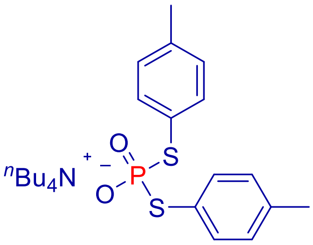

Compound II-1

Physical state: White solid; **TLC:** R_f = 0.72 (DCM/MeOH = 100:1); Yield: 72%.

^{1}H NMR (400 MHz, Chloroform-*d*) δ = 7.47 (d, *J*=6.8, 4H), 6.96 (d, *J*=8.0, 4H), 3.16 – 3.10 (m, 8H), 2.24 (s, 6H), 1.54 – 1.45 (m, 8H), 1.37 – 1.29 (m, 8H), 0.91 (t, *J*=7.2, 12H).

^{31}P NMR (162 MHz, Chloroform-*d*) δ = 24.45.

^{13}C NMR (151 MHz, Chloroform-*d*) δ = 135.7, 133.2 (d, *J*=4.5), 131.4 (d, *J*=4.5), 128.8, 58.5, 23.9, 21.0, 19.6, 13.7.

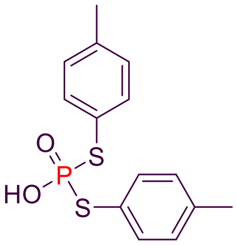

Compound II-1a

Physical state: White solid; **TLC:** R_f = 0.62 (DCM/MeOH = 20:1); Yield: 82%.

^{1}H NMR (400 MHz, DMSO-*d*6) δ = 7.35 (d, *J*=7.6, 4H), 7.02 (d, *J*=7.6, 4H), 2.25 (s, 6H).

^{31}P NMR (162 MHz, DMSO-*d*6) δ = 21.60.

^{13}C NMR (151 MHz, DMSO-*d*6) δ = 135.8, 133.1 (d, *J*=4.5), 131.8 (d, *J*=4.5), 129.2, 21.1.

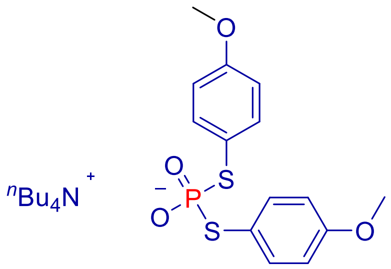

Compound II-2

Physical state: White solid; **TLC:** R_f = 0.68 (DCM/MeOH = 100:1); Yield: 73%.

^{1}H NMR (400 MHz, Chloroform-*d*) δ = 7.42 (d, *J*=8.4, 4H), 6.65 (d, *J*=8.4, 4H), 3.66 (s, 6H), 3.22 – 3.18 (m, 8H), 1.55– 1.49 (s, 8H), 1.35– 1.29 (m, 8H), 0.90– 0,86 (m, 12H).

^{31}P NMR (162 MHz, Chloroform-*d*) δ = 26.46.

^{13}C NMR (151 MHz, Chloroform-*d*) δ = 158.4, 134.9 (d, *J*=4.5), 132.4, 113.7, 58.7, 55.1, 24.0, 19.6, 13.6.

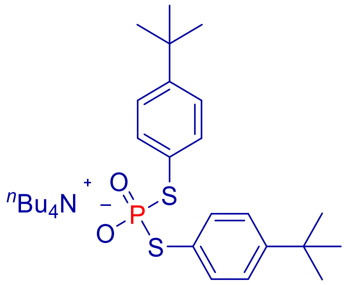

Compound II-3

Physical state: White solid; **TLC:**R_f = 0.66 (DCM/MeOH = 100:1); Yield: 57%.

^{1}H NMR (400 MHz, Chloroform-*d*) δ = 7.49 (d, *J*=6.8, 4H), 7.18 (d, *J*=6.0, 4H), 3.22 – 3.17 (m, 8H), 1.58 – 1.51 (m, 8H), 1.40 – 1.32 (m, 8H), 1.24 – 1.21 (m, 18H), 0.92 (t, *J*=7.2, 12H).

^{31}P NMR (162 MHz, Chloroform-*d*) δ = 26.04.

^{13}C NMR (151 MHz, Chloroform-*d*) δ = 148.9, 133.0 (d, *J*=4.5), 131.1, 125.2, 58.6, 34.3, 31.3, 24.0, 19.6, 13.7.

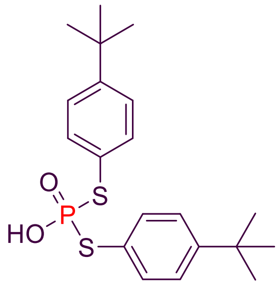

Compound II-3a

Physical state: White solid; **TLC:** R_f = 0.45 (DCM/MeOH = 20:1); Yield: 74%.

^{1}H NMR (400 MHz, DMSO-*d*6) δ = 7.40 (d, *J*=7.6, 4H), 7.24 (d, *J*=8.0, 4H), 1.25 (s, 18H).

^{31}P NMR (162 MHz, DMSO-*d*6) δ = 21.30.

^{13}C NMR (151 MHz, DMSO-*d*6) δ = 149.0, 133.0 (d, *J*=4.5), 131.8 (d, *J*=6.0), 125.4, 34.5, 31.5.

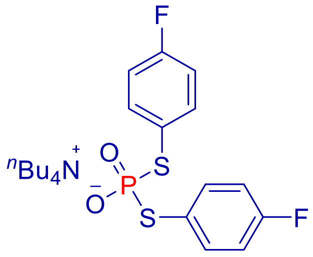

Compound II-4

Physical state: White solid; **TLC:** R_f = 0.71 (DCM/MeOH = 100:1); Yield: 89%.

^{1}H NMR (400 MHz, Chloroform-*d*) δ = 7.55 – 7.52 (m, 4H), 6.85 (t, *J*=8.8, 4H), 3.20 – 3.11 (m, 8H), 1.59 – 1.52 (m, 8H), 1.39 – 1.33 (m, 8H), 0.93 (t, *J*=7.2, 12H).

^{31}P NMR (162 MHz, Chloroform-*d*) δ = 24.41.

^{13}C NMR (151 MHz, Chloroform-*d*) δ = 161.9 (dd, *J*=243.0, 1.5), 135.0 (dd, *J*=7.5, 4.5), 129.8–129.7 (m), 115.1-114.9 (m), 58.7, 23.9, 19.6, 13.6.

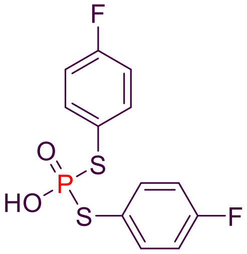

Compound II-4a

Physical state: White solid; **TLC:** R_f = 0.61 (DCM/MeOH = 20:1); Yield: 76%.

^{1}H NMR (400 MHz, DMSO-d_6) δ = 7.52 – 7.45 (m, 4H), 7.05 (t, *J*=8.8, 4H).

^{19}F NMR (376 MHz, DMSO-d_6) δ = -116.34.

^{31}P NMR (162 MHz, DMSO-d_6) δ = 21.07.

^{13}C NMR (151 MHz, DMSO-d_6) δ = 161.6 (d, *J*=241.5), 135.1 (dd, *J*=7.5, 4.5), 133.0 – 129.2 (m), 115.5 (d, *J*=21.0).

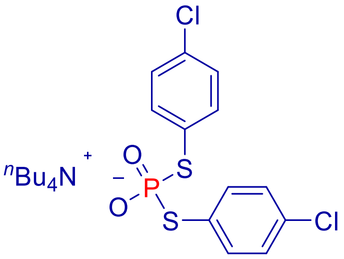

Compound II-5

Physical state: White solid; **TLC:** R_f = 0.71 (DCM/MeOH = 100:1); Yield: 87%.

^{1}H NMR (400 MHz, Chloroform-*d*) δ = 7.43 (d, *J*=8.4, 4H), 7.28 – 7.23 (m, 4H), 3.21 – 3.17 (m, 8H), 1.58 – 1.53 (m, 9H), 1.38 – 1.34 (m, 8H), 0.95 – 0.92 (m, 12H).

^{31}P NMR (162 MHz, Chloroform-*d*) δ = 22.98.

^{13}C NMR (151 MHz, Chloroform-*d*) δ = 134.6 (d, *J*=4.5), 133.9 (d, *J*=6.0), 131.1, 120.4 (d, *J*=3.0), 58.7, 23.9, 19.6, 13.6.

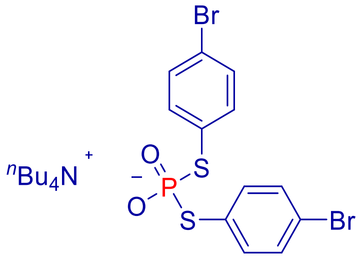

Compound II-6

Physical state: White solid; **TLC:** R_f = 0.71 (DCM/MeOH = 100:1); Yield: 83%.

^{1}H NMR (500 MHz, Acetone-*d*$_6$) δ = 7.55 (d, *J*=6.4, 4H), 7.34 (d, *J*=6.8, 4H), 3.47 – 3.43 (m, 8H), 1.83 – 1.78 (m, 8H), 1.45 – 1.41 (m, 8H), 0.97 (t, *J*=6.0, 12H).

^{31}P NMR (202 MHz, Acetone-*d*$_6$) δ = 18.34.

^{13}C NMR (151 MHz, Acetone-*d*$_6$) δ = 135.9 (d, *J*=4.5), 134.3 (d, *J*=4.5), 130.7, 119.2 (d, *J*=3.0), 58.5 – 58.4 (m), 23.7, 19.4, 13.1.

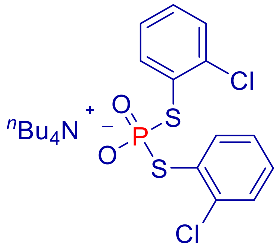

Compound II-7

Physical state: White solid; **TLC:** R_f = 0.73 (DCM/MeOH = 100:1); Yield: 88%.

^{1}H NMR (400 MHz, Chloroform-*d*) δ = 8.18 (d, *J*=8.0, 2H), 7.23 (d, *J*=8.0, 2H), 7.10 (t, *J*=7.2, 2H), 7.02 (t, *J*=7.6, 2H), 3.21 – 3.06 (m, 8H), 1.63 – 1.45 (m, 8H), 1.42 – 1.26 (m, 8H), 0.92 (t, *J*=7.2, 12H).

^{31}P NMR (162 MHz, Chloroform-*d*) δ = 22.40.

^{13}C NMR (151 MHz, Chloroform-*d*) δ = 135.1 (d, *J*=6.0), 134.5 (d, *J*=4.5), 133.9 (d, *J*=4.5), 128.9, 127.0, 126.6, 58.7, 24.0, 19.6, 13.6.

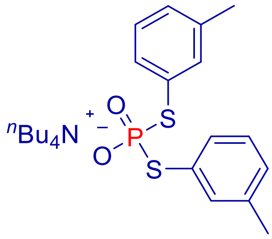

Compound II-8

Physical state: White solid; **TLC:** R_f = 0.56 (DCM/MeOH = 100:1); Yield: 61%.

^{1}H NMR (400 MHz, Chloroform-*d*) δ = 7.46 (d, *J*=7.6, 2H), 7.34 (s, 2H), 7.06 (d, *J*=7.6, 2H), 6.93 (d, *J*=7.2, 2H), 3.18 – 3.02 (m, 8H), 2.22 (s, 6H), 1.60 – 1.44 (m, 8H), 1.43 – 1.21 (m, 8H), 0.92 (t, *J*=7.2, 12H).

^{31}P NMR (162 MHz, Chloroform-*d*) δ = 27.18.

^{13}C NMR (151 MHz, Chloroform-*d*) δ = 137.4, 133.7, 130.3, 127.9, 127.1, 58.4, 23.8, 21.2, 19.5, 13.5.

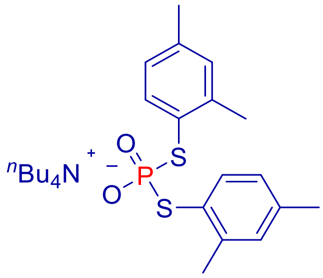

Compound II-9

Physical state: White solid; **TLC:** R_f = 0.56 (DCM/MeOH = 100:1); Yield: 61%.

^{1}H NMR (400 MHz, Chloroform-*d*) δ = 7.61 (d, *J*=8.0, 2H), 6.73 (s, 2H), 6.66 (d, *J*=8.0, 2H), 3.00 – 2.96 (m, 8H), 2.08 (s, 6H), 2.06 (s, 6H), 1.37 – 1.33 (m, 8H), 1.21 – 1.17 (m, 8H), 0.77 (t, *J*=8.0, 12H).

^{31}P NMR (162 MHz, Chloroform-*d*) δ = 27.37.

^{13}C NMR (151 MHz, Chloroform-*d*) δ = 139.5, 135.6, 134.0, 130.2, 126.4, 58.2, 23.8, 21.1, 20.8, 19.5, 13.5.

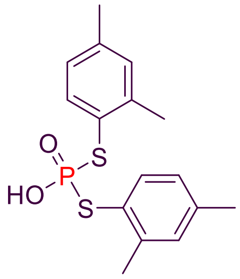

Compound II-9a

Physical state: White solid; **TLC:** R_f = 0.44 (DCM/MeOH = 20:1); Yield: 81%.

^{1}H NMR (400 MHz, DMSO-*d*6) δ = 7.62 – 7.60 (m, 2H), 6.93 (s, 2H), 6.87 – 6.84 (m, 2H), 2.21 (s, 6H), 2.17 (s, 6H).

^{31}P NMR (162 MHz, DMSO-*d*6) δ = 22.33.

^{13}C NMR (151 MHz, DMSO-*d*6) δ = 139.5 (d, *J*=6.0), 135.8 (d, *J*=1.5), 134.2 (d, *J*=3.0), 131.0 (d, *J*=6.0), 130.6, 126.7, 21.3, 21.0.

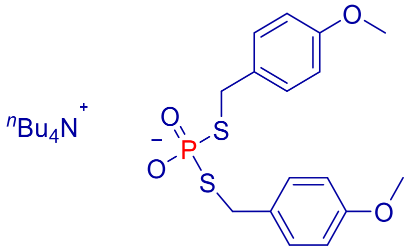

Compound II-10

Physical state: White solid; **TLC:** R_f = 0.69 (DCM/MeOH = 100:1); Yield: 65%.

¹H NMR (400 MHz, Chloroform-*d*) δ = 7.21 (d, *J*=8.4, 4H), 6.72 (d, *J*=8.4, 4H), 4.00 – 3.96 (m, 4H), 3.71 (s, 6H), 3.22 – 3.17 (m, 8H), 1.56 – 1.53 (m, 8H), 1.37 – 1.34 (m, 8H), 0.93 – 0.90 (m, 12H).

³¹P NMR (162 MHz, Chloroform-*d*) δ = 31.40.

¹³C NMR (151 MHz, Chloroform-*d*) δ = 158.1, 130.1, 113.8, 113.5, 58.7, 55.2, 36.4, 24.0, 19.7, 13.7.

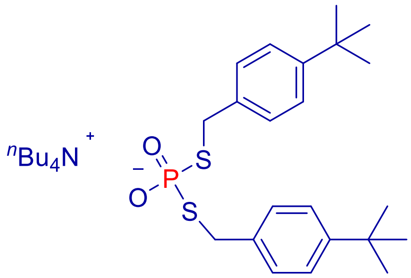

Compound II-11

Physical state: White solid; **TLC:** R_f = 0.66 (DCM/MeOH = 100:1); Yield: 68%.

¹H NMR (400 MHz, Chloroform-*d*) δ = 7.23 (s, 8H), 4.04 (d, *J*=8.4, 4H), 3.23 (s, 8H), 1.61 – 1.53 (m, 8H), 1.42 – 1.32 (m, 8H), 1.25 (s, 18H), 0.92 (t, *J*=7.2, 12H).

³¹P NMR (162 MHz, Chloroform-*d*) δ = 30.98.

¹³C NMR (151 MHz, Chloroform-*d*) δ = 149.0, 136.7, 128.6, 125.0, 58.6, 36.6, 34.3, 31.3, 24.0, 19.6, 13.7.

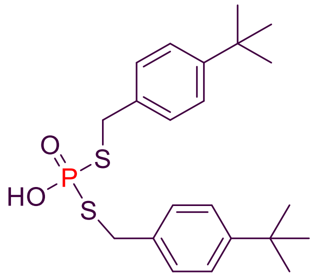

Compound II-11aa

Physical state: White solid; **TLC:** R_f = 0.57 (DCM/MeOH = 20:1); Yield: 76%.

¹H NMR (400 MHz, DMSO-*d*₆) δ = 7.26 (d, *J*=8.0, 4H), 7.20 (d, *J*=8.0, 4H), 3.83 (d, *J*=8.4, 4H), 1.24 (s, 18H).

³¹P NMR (162 MHz, DMSO-*d*₆) δ = 27.63.

¹³C NMR (151 MHz, DMSO-*d*₆) δ = 149.1, 137.1 (d, *J*=6.0), 128.8, 125.3, 36.3 (d, *J*=3.0), 34.5, 31.6.

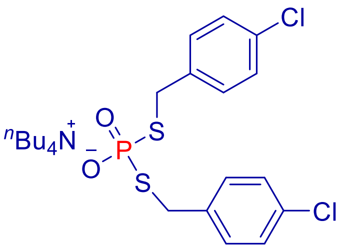

Compound II-12

Physical state: White solid; **TLC:** R_f = 0.78 (DCM/MeOH = 100:1); Yield: 72%.

^{1}H NMR (400 MHz, Chloroform-*d*) δ = 7.21 (d, *J*=8.0, 4H), 7.12 (d, *J*=8.4, 4H), 3.94 (d, *J*=9.6, 4H), 3.19 – 3.14 (m, 8H), 1.56 – 1.51 (m, 8H), 1.37 – 1.31 (m, 8H), 0.90 (t, *J*=7.2, 12H).

^{31}P NMR (162 MHz, Chloroform-*d*) δ = 29.92.

^{13}C NMR (151 MHz, Chloroform-*d*) δ = 138.7 (d, *J*=6.0), 131.8, 130.4, 128.0, 58.6, 36.1 (d, *J*=3.0), 23.9, 19.6, 13.6.

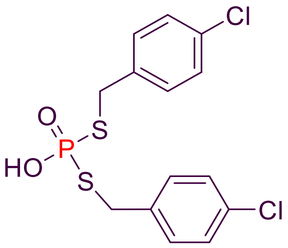

Compound II-12a

Physical state: White solid; **TLC:** R_f = 0.47 (DCM/MeOH = 20:1); Yield: 67%.

^{1}H NMR (400 MHz, DMSO-d_6) δ = 7.34 – 7.26 (m, 8H), 3.81 (d, *J*=10.0, 4H).

^{31}P NMR (162 MHz, DMSO-d_6) δ = 27.02.

^{13}C NMR (151 MHz, DMSO-d_6) δ = 139.72 (d, *J*=4.5), 131.32, 131.04, 128.48, 35.78 (d, *J*=3.0).

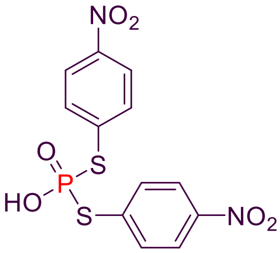

Compound II-13a

Physical state: White solid; **TLC:** R_f = 0.46 (DCM/MeOH = 20:1); Yield: 78% [b].([b]The yields are the reactions in one-pot.)

^{1}H NMR (400 MHz, DMSO-d_6) δ = 8.03 (d, *J*=8.8, 4H), 7.75 (d, *J*=8.8, 4H).

^{31}P NMR (162 MHz, DMSO-d_6) δ = 16.22.

^{13}C NMR (151 MHz, DMSO-d_6) δ = 145.9, 144.7 (d, *J*=6.0), 132.2 (d, *J*=4.5), 123.6.

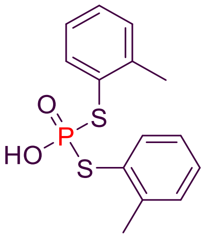

Compound II-14a

Physical state: White solid; **TLC:** R_f = 0.44 (DCM/MeOH = 20:1); Yield: 74%[b].([b]The yields are the reactions in one pot.)

^{1}H NMR (400 MHz, DMSO-*d*$_6$) δ = 7.76 (d, *J*=7.2, 2H), 7.13 – 7.03 (m, 6H), 2.16 (s, 6H).

^{31}P NMR (162 MHz, DMSO-*d*$_6$) δ = 21.44.

^{13}C NMR (151 MHz, DMSO-*d*$_6$) δ = 156.7, 134.0, 134.0, 129.9, 126.8, 126.0, 21.2.

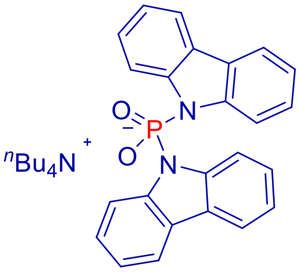

Compound III-1

Physical state: White solid; **TLC:** R_f = 0.42 (DCM/MeOH = 20:1); Yield: 88%.

^{1}H NMR (400 MHz, Chloroform-*d*) δ = 8.38 (d, *J*=8.4, 4H), 7.83 (d, *J*=7.6, 4H), 7.18 (t, *J*=7.2, 4H), 7.07 (t, *J*=7.2, 4H), 2.50 – 2.46 (m, 8H), 1.06 – 0.96 (m, 8H), 0.94 – 0.86 (m, 8H), 0.72 (t, *J*=7.2, 12H).

^{31}P NMR (162 MHz, Chloroform-*d*) δ = -13.39.

^{13}C NMR (151 MHz, Chloroform-*d*) δ = 142.3 (d, *J*=4.5), 125.5, 124.9 (d, *J*=7.5), 119.7, 118.9, 115.6, 57.8, 23.4, 19.2, 13.5.

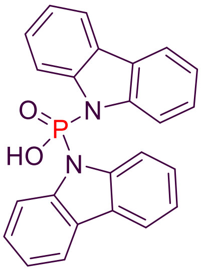

Compound III-1a

Physical state: White solid; **TLC:** R_f = 0.25 (DCM/MeOH = 10:1); Yield: 86%.

^{1}H NMR (400 MHz, DMSO-*d*$_6$) δ = 8.28 (d, *J*=8.4, 2H), 7.95 (d, *J*=7.6, 2H), 7.20 (t, *J*=8.4, 2H), 7.08 (t, *J*=6.8, 2H).

^{31}P NMR (162 MHz, DMSO-*d*$_6$) δ = -14.95.

^{13}C NMR (151 MHz, DMSO-*d*$_6$) δ = 142.3 (d, *J*=4.5), 125.7, 124.6 (d, *J*=6.0), 120.0, 119.7, 115.5.

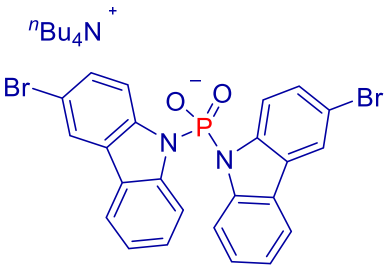

Compound III-2

Physical state: White solid; **TLC:**R_f = 0.41 (DCM/MeOH = 20:1); Yield: 86%.

^{1}H NMR (400 MHz, Chloroform-*d*) δ = 8.28 – 8.24 (m, 4H), 7.92 – 7.75 (m, 4H), 7.19 – 7.07 (m, 6H), 2.67 – 2.63 (m, 8H), 1.19 – 1.13 (m, 8H), 1.01 (d, *J*=7.4, 9H), 0.75 (t, *J*=7.2, 12H).

^{31}P NMR (162 MHz, Chloroform-*d*) δ = -13.76.

^{13}C NMR (151 MHz, Chloroform-*d*) δ = 142.4, 140.9, 128.2, 126.8 (d, *J*=7.5), 126.4, 123.8 (d, *J*=6.0), 121.7, 120.2, 119.1, 116.9, 115.4, 112.9, 58.2, 23.5, 19.3, 13.4.

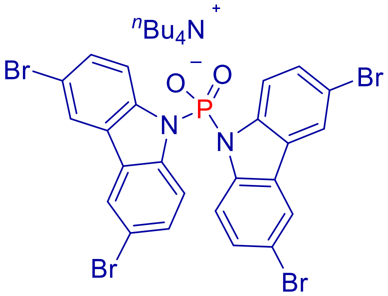

Compound III-3

Physical state: White solid; **TLC:**R_f = 0.34 (DCM/MeOH = 20:1); Yield: 76%.

^{1}H NMR (400 MHz, Chloroform-*d*) δ = 8.07 (s, 4H), 7.76 (s, 4H), 7.16 – 6.93 (m, 4H), 2.66 – 2.62 (m, 8H), 1.22 – 1.14 (m, 8H), 1.01 – 0.96 (m, 8H), 0.74 (t, *J*=7.2, 12H).

^{31}P NMR (162 MHz, Chloroform-*d*) δ = -13.82.

^{13}C NMR (151 MHz, Chloroform-d) δ = 141.0, 129.0, 125.6 (d, J=6.0), 122.0, 116.8, 113.4, 58.56, 23.6, 19.4, 13.4.

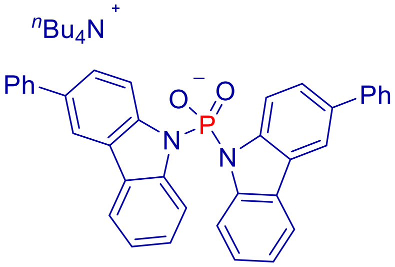

Compound III-4

Physical state: White solid; **TLC:**R_f = 0.35 (DCM/MeOH = 20:1); Yield: 91%.

^{1}H NMR (400 MHz, DMSO-*d*$_6$) δ = 8.43 (d, *J*=8.8, 2H), 8.35 – 8.32 (m, 4H), 8.11 (d, *J*=7.6, 2H), 7.73 (d, *J*=7.2, 4H), 7.59 (dd, *J*=8.8, 2.0, 2H), 7.43 (t, *J*=7.6, 4H), 7.30 – 7.24 (m, 4H), 7.14 (t, *J*=7.2, 2H), 3.09 – 3.05 (m, 8H), 1.49 – 1.45 (m, 8H), 1.25 – 1.19 (m, 8H), 0.87 (t, *J*=7.2, 12H).

^{31}P NMR (162 MHz, DMSO-*d*$_6$) δ = -14.95.

¹³C NMR (151 MHz, DMSO-d_6) δ = 142.8 (d, *J*=4.5), 141.9 (d, *J*=4.5), 141.3, 132.4, 129.2, 127.0, 126.9, 126.0, 125.4 (d, *J*=7.5), 124.8 (d, *J*=6.0), 124.8, 120.3, 120.1, 117.8, 115.8, 115.6, 57.9, 23.4, 19.6, 13.9.

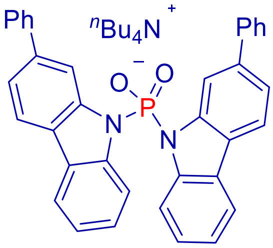

Compound III-5

Physical state: White solid; **TLC:**R_f = 0.37 (DCM/MeOH = 20:1); Yield: 74%.

¹H NMR (400 MHz, Chloroform-*d*) δ = 8.66 (d, *J*=8.0, 2H), 8.16 (s, 2H), 7.83 (d, *J*=7.2, 2H), 7.76 (d, *J*=8.0, 2H), 7.23 (d, *J*=8.0, 2H), 7.15 – 7.14 (m, 10H), 6.98 (s, 4H), 2.35 – 2.31 (m, 8H), 0.92 – 0.85 (m, 8H), 0.83 – 0.76 (m, 8H), 0.55 (t, *J*=7.2, 12H).

³¹P NMR (162 MHz, Chloroform-*d*) δ = -11.78.

¹³C NMR (151 MHz, Chloroform-*d*) δ = 143.2, 142.3, 141.5, 138.3, 128.3, 127.1, 126.3, 126.1, 124.5 (d, *J*=7.5), 124.4 (d, *J*=7.5), 120.0, 119.2, 119.2, 119.0, 115.8, 113.5, 57.6, 23.2, 19.0, 13.3.

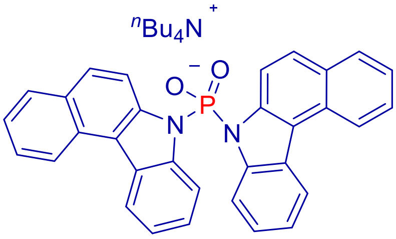

Compound III-6

Physical state: White solid; **TLC:** R_f = 0.34 (DCM/MeOH = 20:1); Yield: 83%.

¹H NMR (400 MHz, Chloroform-*d*) δ = 8.77 (d, *J*=8.8, 2H), 8.64 (d, *J*=8.4, 2H), 8.59 – 8.55 (m, 2H), 8.39 – 8.33 (m, 2H), 7.83 (d, *J*=8.0, 2H), 7.60 (d, *J*=9.2, 2H), 7.55 (t, *J*=7.2, 2H), 7.35 (t, *J*=7.6, 2H), 7.22 – 7.20 (m, 4H), 2.22 – 2.18 (m, 8H), 0.81 – 0.69 (m, 16H), 0.57 (t, *J*=6.8, 12H).

³¹P NMR (162 MHz, Chloroform-*d*) δ = -13.86.

¹³C NMR (151 MHz, Chloroform-*d*) δ = 141.7 (d, *J*=4.5), 140.4 (d, *J*=6.0), 129.3 (d, *J*=6.0), 128.7, 126.4, 126.15, 125.5 (d, *J*=9.0), 124.2, 123.0, 122.9, 120.9, 120.5, 117.0, 116.0, 57.6, 23.1, 19.1, 13.3.

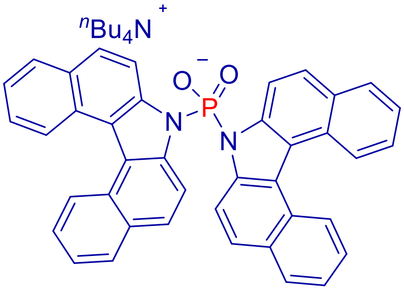

Compound III-7

Physical state: White solid; **TLC:** R_f = 0.42 (DCM/MeOH = 20:1); Yield: 95%.

^{1}H NMR (400 MHz, Chloroform-*d*) δ = 8.91 (d, *J*=8.4, 4H), 8.86 (d, *J*=8.4, 4H), 7.82 (d, *J*=8.4, 4H), 7.59 (d, *J*=8.8, 4H), 7.47 (t, *J*=7.7, 4H), 7.37 (t, *J*=7.2, 4H), 2.00 – 1.93 (m, 8H), 0.59 – 0.49 (m, 16H), 0.42 – 0.39 (m, 12H).

^{31}P NMR (162 MHz, Chloroform-*d*) δ = -14.03.

^{13}C NMR (151 MHz, Chloroform-*d*) δ = 139.8 (d, *J*=6.0), 130.0, 128.5, 128.3, 126.0, 125.2, 124.4, 123.2, 119.1 (d, *J*=7.5), 116.7, 57.4, 22.9, 18.8, 13.1.

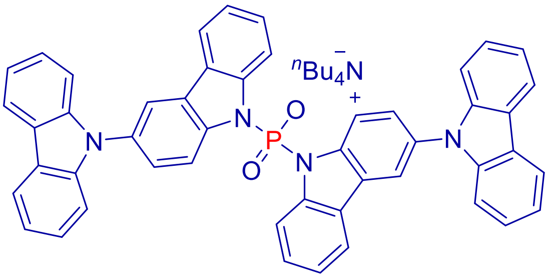

Compound III-8

Physical state: White solid; **TLC:** R_f = 0.22 (DCM/MeOH = 20:1); Yield: 90%.

^{1}H NMR (400 MHz, Chloroform-*d*) δ = 8.62 (d, *J*=8.8, 2H), 8.54 (d, *J*=8.4, 2H), 8.15 (d, *J*=7.6, 4H), 8.07 (s, 2H), 7.88 (d, *J*=6.8, 2H), 7.42 (d, *J*=8.4, 2H), 7.35 (s, 10H), 7.25 (d, *J*=4.4, 4H), 7.19 – 7.15 (m, 2H), 3.12 – 3.08 (m, 8H), 1.52 – 1.44 (m, 8H), 1.26 – 1.20 (m, 8H), 0.85 (t, *J*=7.2, 12H).

^{31}P NMR (162 MHz, Chloroform-*d*) δ = -12.63.

^{13}C NMR (151 MHz, Chloroform-*d*) δ = 142.9, 141.7, 1414.2, 130.0, 128.0, 126.5, 125.7, 125.0, 124.9, 123.0, 120.5, 120.1, 119.5, 119.4, 118.1, 116.3, 115.5, 109.9, 58.7, 23.8, 19.5, 13.5.

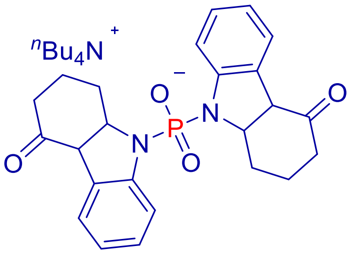

Compound III-9

Physical state: White solid; **TLC:** R_f = 0.18 (DCM/MeOH = 20:1); Yield: 78%.

^{1}H NMR (400 MHz, Chloroform-*d*) δ = 8.34 (d, *J*=8.0, 2H), 8.12 (d, *J*=7.2, 2H), 7.15 – 7.08 (m, 4H), 3.11 – 3.07 (m, 8H), 2.92 (t, *J*=6.4, 4H), 2.35 (t, *J*=6.4, 4H), 1.96 (t, *J*=6.4, 4H), 1.53 – 1.30 (m, 8H), 1.28 – 1.22 (m, 8H), 0.86 (t, *J*=7.2, 12H).

^{31}P NMR (162 MHz, Chloroform-*d*) δ = -15.17.

^{13}C NMR (151 MHz, Chloroform-*d*) δ = 195.4, 155.9 (d, *J*=4.5), 139.4 (d, *J*=6.0), 125.9 (d, *J*=7.5), 123.1, 122.4, 120.6, 115.5 – 115.4 (m), 58.6, 37.9, 34.7, 24.2, 23.8, 23.6, 19.5, 13.5.

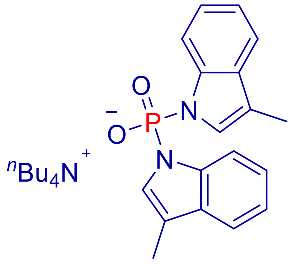

Compound III-10

Physical state: White solid; **TLC:** R_f = 0.41 (DCM/MeOH = 20:1); Yield: 92%.

^{1}H NMR (400 MHz, Chloroform-*d*) δ = 8.17 (d, *J*=8.4, 2H), 7.48 (s, 2H), 7.33 (d, *J*=8.0, 2H), 7.06 (t, *J*=7.2, 2H), 6.99 (t, *J*=6.4 2H), 3.04 – 3.01 (m, 8H), 2.18 (s, 6H), 1.46 – 1.31 (m, 8H), 1.29 – 1.24 (m, 8H), 0.91 (t, *J*=7.3, 12H).

^{31}P NMR (162 MHz, Chloroform-*d*) δ = -15.27.

^{13}C NMR (151 MHz, Chloroform-*d*) δ = 137.9 (d, *J*=4.5), 131.0 (d, *J*=9.0), 127.9 (d, *J*=4.5), 121.4, 119.2, 117.8, 114.7, 111.7 (d, *J*=6.0), 57.7, 23.5, 19.3, 9.6.

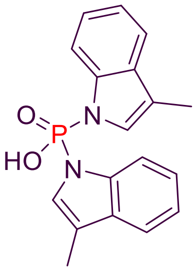

Compound III-10a

Physical state: White solid; **TLC:** R_f = 0.23 (DCM/MeOH = 10:1); Yield: 89%[a] / 77[b]. ([a]The yields are the reactions by step-by-step. [b]The yields are the reactions in one-pot.)

^{1}H NMR (400 MHz, DMSO-d_6) δ = 8.01 (d, *J*=8.0, 2H), 7.37 (s, 2H), 7.29 (d, *J*=7.6, 2H), 7.01 – 6.98 (m, 2H), 6.91 (t, *J*=6.8, 2H), 2.12 (s, 6H).

^{31}P NMR (162 MHz, DMSO-d_6) δ = -17.15.

^{13}C NMR (151 MHz, DMSO-d_6) δ = 137.9 (d, *J*=3.0), 130.9 (d, *J*=7.5), 128.0 (d, *J*=6.0), 121.5, 119.4, 118.2, 114.9, 111.1 (d, *J*=6.0), 9.9.

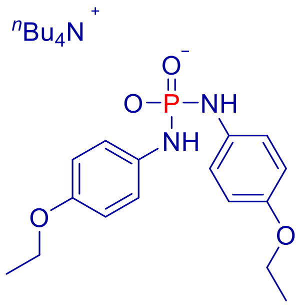

Compound III-11

Physical state: White solid; **TLC:** R_f = 0.19 (DCM/MeOH = 20:1); Yield: 67%.

^{1}H NMR (400 MHz, Chloroform-*d*) δ = 6.84 (d, *J*=8.4, 4H), 6.51 (s, 4H), 5.20 (s, 1H), 3.76 (s, 4H), 3.60 (s, 1H), 3.13 – 3.06 (m, 8H), 1.49 – 1.40 (m, 8H), 1.31 – 1.22 (m, 14H), 0.89 (t, *J*=7.2, 12H).

^{31}P NMR (162 MHz, Chloroform-*d*) δ = -4.84.

^{13}C NMR (151 MHz, Chloroform-*d*) δ = 151.5, 137.7, 117.7, 114.9, 63.7, 58.4, 23.8, 19.6, 14.9, 13.6.

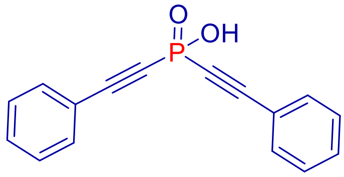

Compound IV-1

Physical state: White solid; **TLC:** R_f = 0.32 (DCM/MeOH = 10:1); Yield: 78%.

^{1}H NMR (400 MHz, DMSO-*d*6) δ = 7.46 – 7.43 (m, 4H), 7.38 (s, 6H).

^{31}P NMR (162 MHz, DMSO-*d*6) δ = -32.91.

^{13}C NMR (151 MHz, DMSO-*d*6) δ = 136.7, 134.3, 133.9, 126.9 (d, *J*=4.5), 98.7 (d, *J*=208.5), 96.0 (d, *J*=37.5).

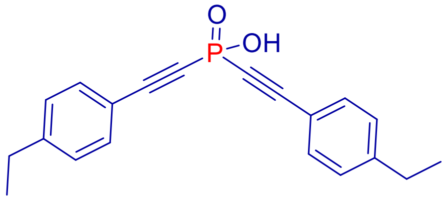

Compound IV-2

Physical state: White solid; **TLC:** R_f = 0.32 (DCM/MeOH = 10:1); Yield: 75%.

^{1}H NMR (400 MHz, DMSO-*d*6) δ = 7.35 (d, *J*=8.0, 4H), 7.21 (d, *J*=8.4, 4H), 2.62 – 2.57 (m, 4H), 1.15 (t, *J*=7.6, 6H).

^{31}P NMR (162 MHz, DMSO-*d*6) δ = -33.15.

^{13}C NMR (151 MHz, DMSO-d_6) δ = 145.4, 131.9, 128.6, 119.6, 94.5, 91.1 (d, J=36.0), 28.4, 15.6.

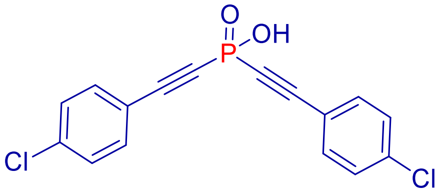

Compound IV-3

Physical state: White solid; **TLC:** R_f = 0.34 (DCM/MeOH = 10:1); Yield: 83%.

^{1}H NMR (400 MHz, DMSO-d_6) δ = 7.47 – 7.41 (m, 8H).

^{31}P NMR (162 MHz, DMSO-d_6) δ = -33.84.

^{13}C NMR (151 MHz, DMSO-d_6) δ = 134.6, 133.9, 129.5, 120.4, 93.5 (d, J=219.0), 91.1 (d, J=40.5).

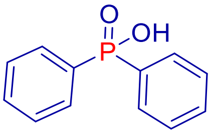

Compound IV-4

Physical state: White solid; **TLC:** R_f = 0.14 (DCM/MeOH = 10:1); Yield: 59%.

^{1}H NMR (400 MHz, DMSO-d_6) δ = 7.67 – 7.63 (m, 4H), 7.22 (m, 6H).

^{31}P NMR (162 MHz, DMSO-d_6) δ = 11.27.

^{13}C NMR (151 MHz, DMSO-d_6) δ = 131.67 (d, J=8.4), 128.72, 127.53 (d, J=11.0), 120.79.

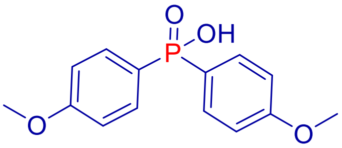

Compound IV-5

Physical state: White solid; **TLC:** R_f = 0.35 (DCM/MeOH = 5:1); Yield: 72%.

^{1}H NMR (400 MHz, DMSO-d_6) δ = 7.52 (t, J=9.1, 4H), 6.79 – 6.69 (m, 4H), 3.67 (s, 6H).

^{31}P NMR (162 MHz, DMSO-d_6) δ = 10.86.

^{13}C NMR (151 MHz, DMSO-d_6) δ = 133.12 (d, J=9.8), 131.67 (d, J=8.6), 127.54 (d, J=11.0), 112.97 (d, J=12.3), 55.29.

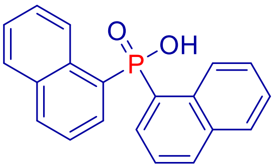

Compound IV-6

Physical state: White solid; **TLC:** R_f = 0.15 (DCM/MeOH = 10:1); Yield: 64%.

^{1}H NMR (400 MHz, DMSO-d_6) δ = 8.97 (d, J=8.3, 2H), 8.26 (s, 2H), 7.78 – 7.73 (m, 4H), 7.43 (s, 2H), 7.32 – 7.25 (m, 4H).

^{31}P NMR (162 MHz, DMSO-d_6) δ = 9.40.

^{13}C NMR (151 MHz, DMSO-d_6) δ = 133.90, 133.73, 133.54, 132.11 (d, J=6.3), 129.72, 129.18, 128.28, 125.11 (d, J=11.9).

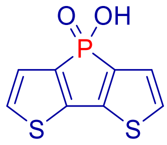

Compound IV-7

Physical state: Yellowish solid; **TLC:** R_f = 0.08 (DCM/MeOH = 10:1); Yield: 36%.

^{1}H NMR (400 MHz, DMSO-d_6) δ = 7.86 (s, 2H), 7.47 (s, 2H).

^{31}P NMR (162 MHz, DMSO-d_6) δ = 10.74.

^{13}C NMR (151 MHz, DMSO-d_6) δ = 145.41 (d, J=134.2), 139.54 (d, J=27.6), 126.69 (d, J=13.8), 125.69 (d, J=13.4).

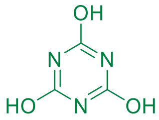

1,3,5-Triazine-2,4,6-(1H,3H,5H)-trion

Physical state: White solid; **TLC:** R_f = 0.12 (PE/EA = 1:1);

^{1}H NMR (400 MHz, DMSO-d_6) δ = 11.14 (s, 3H).

^{13}C NMR (151 MHz, DMSO-d_6) δ = 150.3.

9 Copies of NMR Spectra

^{1}H NMR (400 MHz, $CDCl_3$)

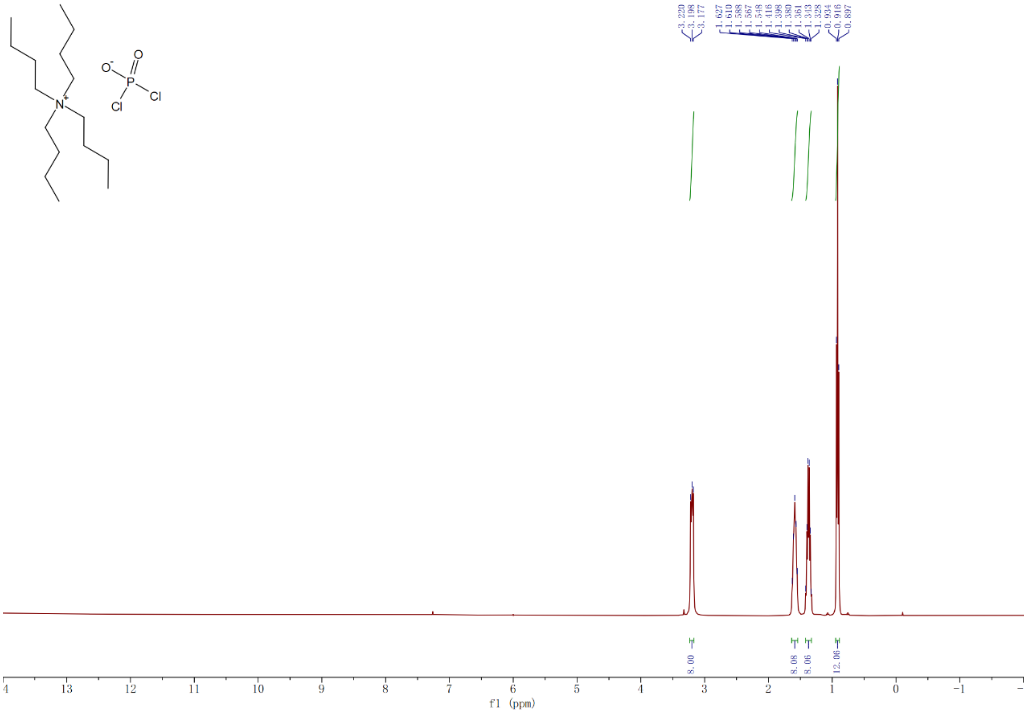

^{31}P NMR (400 MHz, $CDCl_3$)

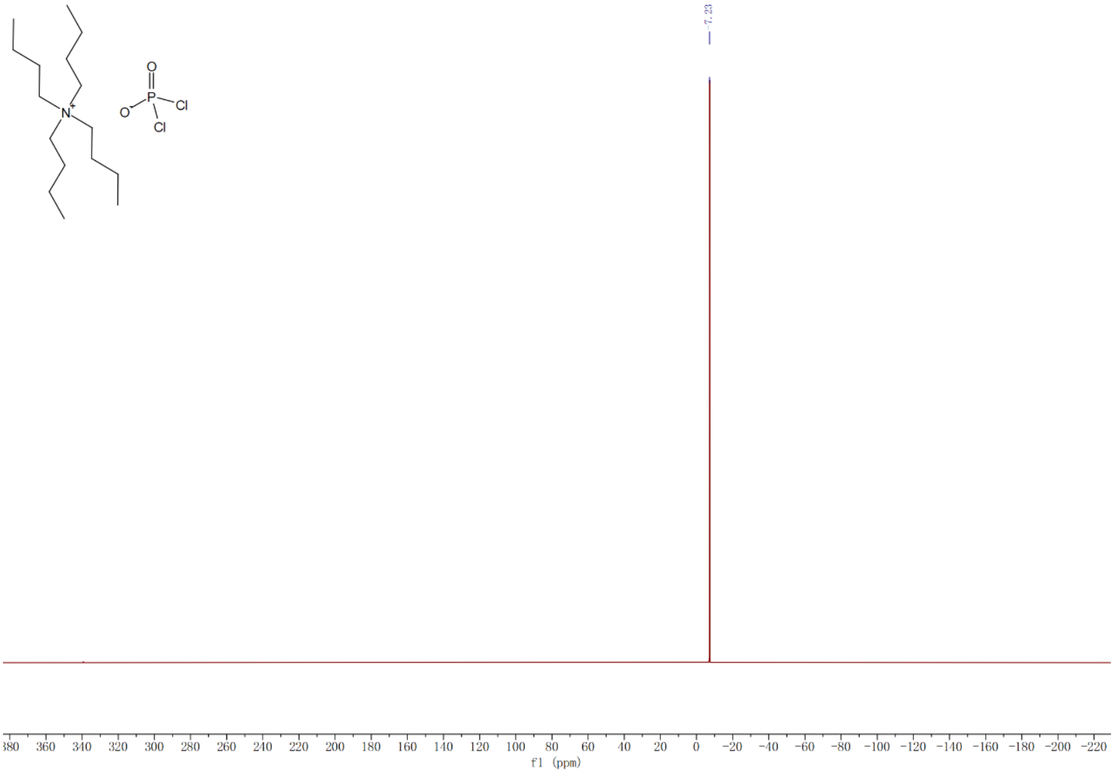

^{13}C NMR (600 MHz, CDCl$_3$)

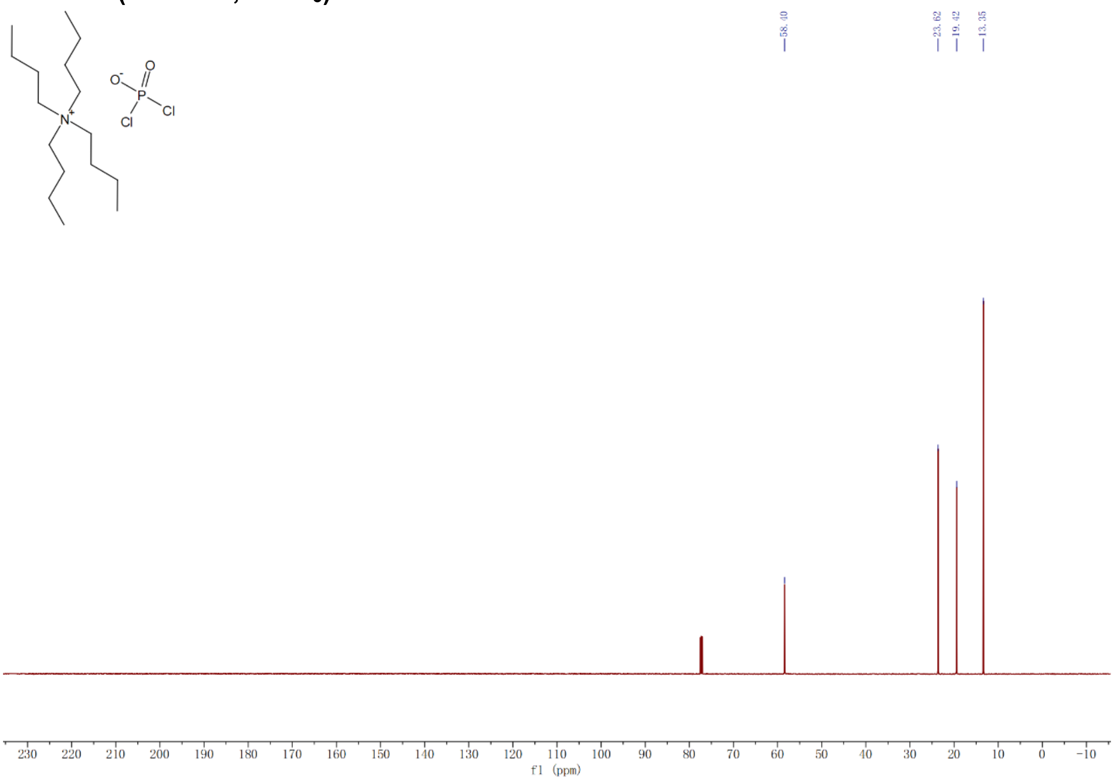

^{31}P NMR (400 MHz, CDCl$_3$)

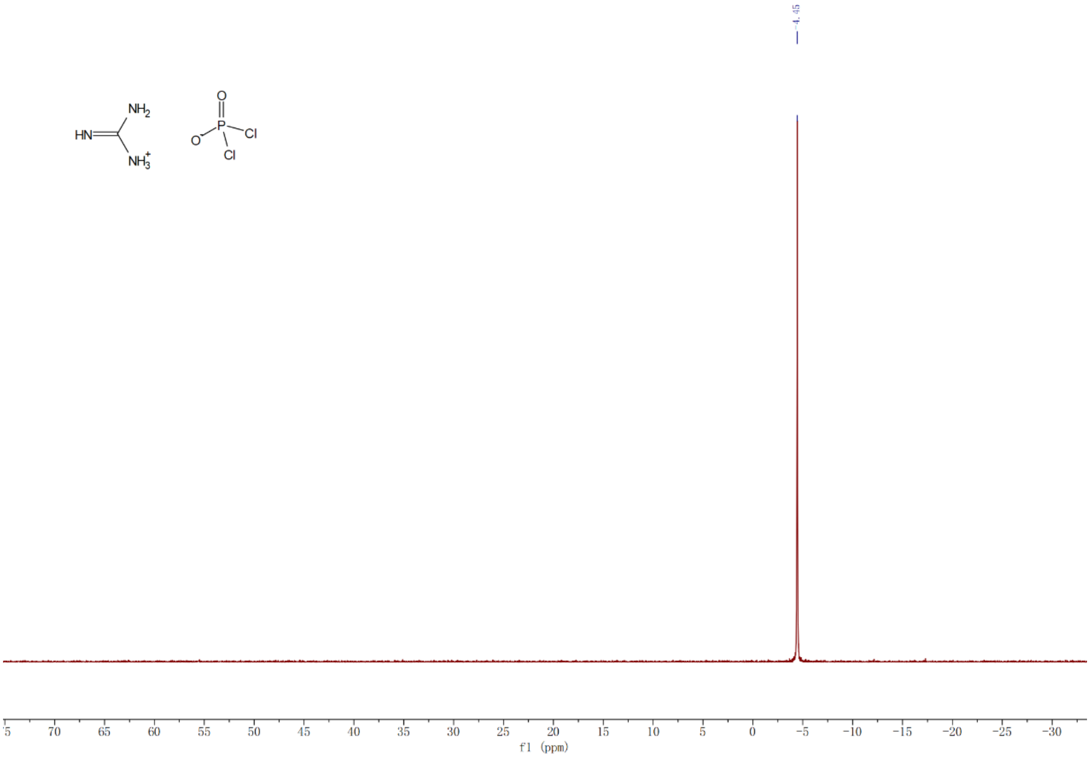

^{13}C NMR (600 MHz, DMSO-D$_6$)

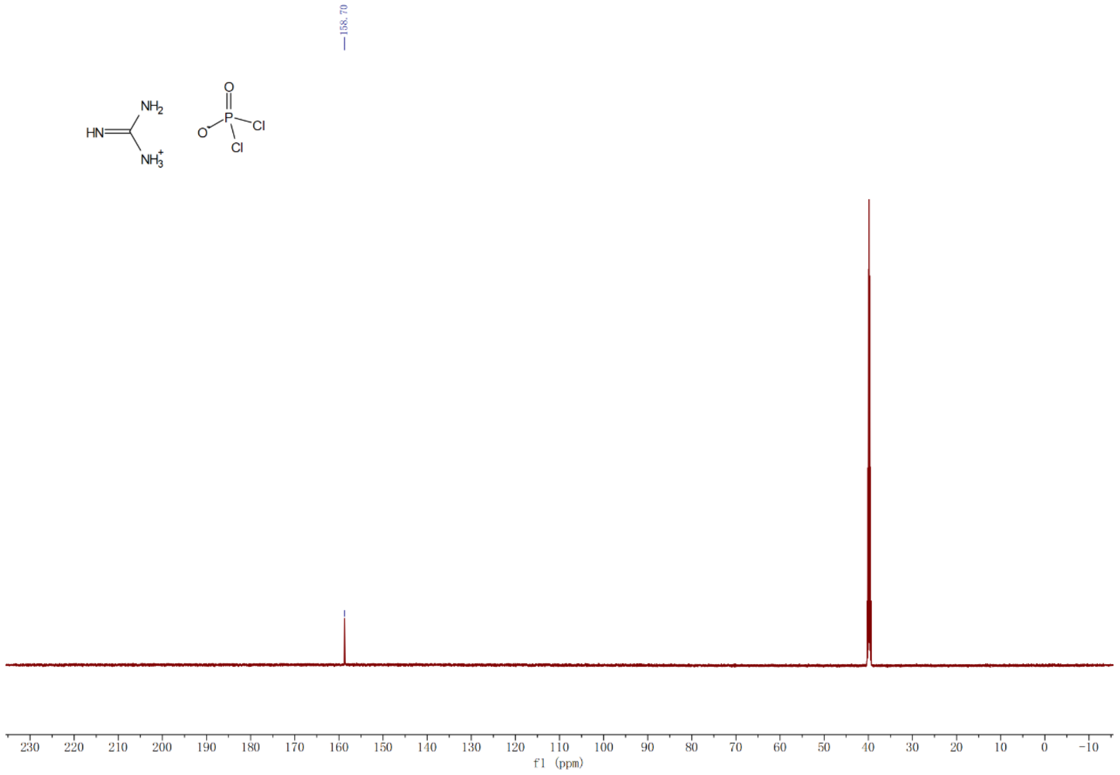

^{1}H NMR (400 MHz, CDCl$_3$)

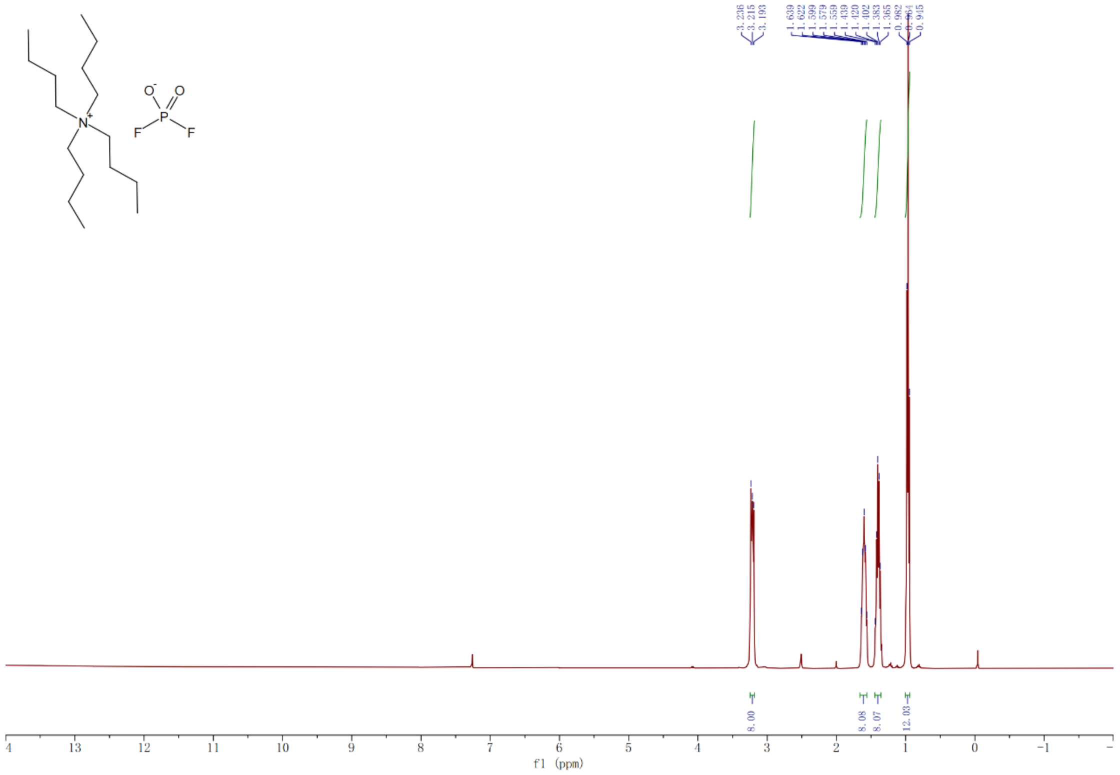

^{31}P NMR (400 MHz, $CDCl_3$)

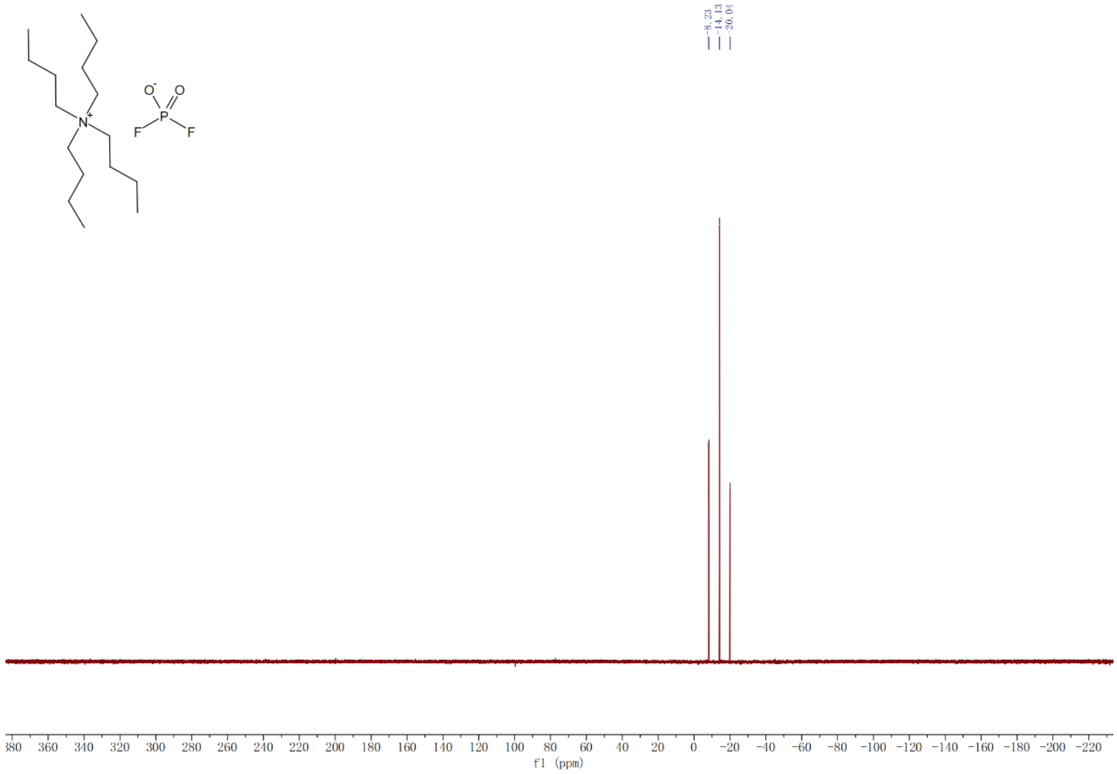

^{19}F NMR (400 MHz, $CDCl_3$)

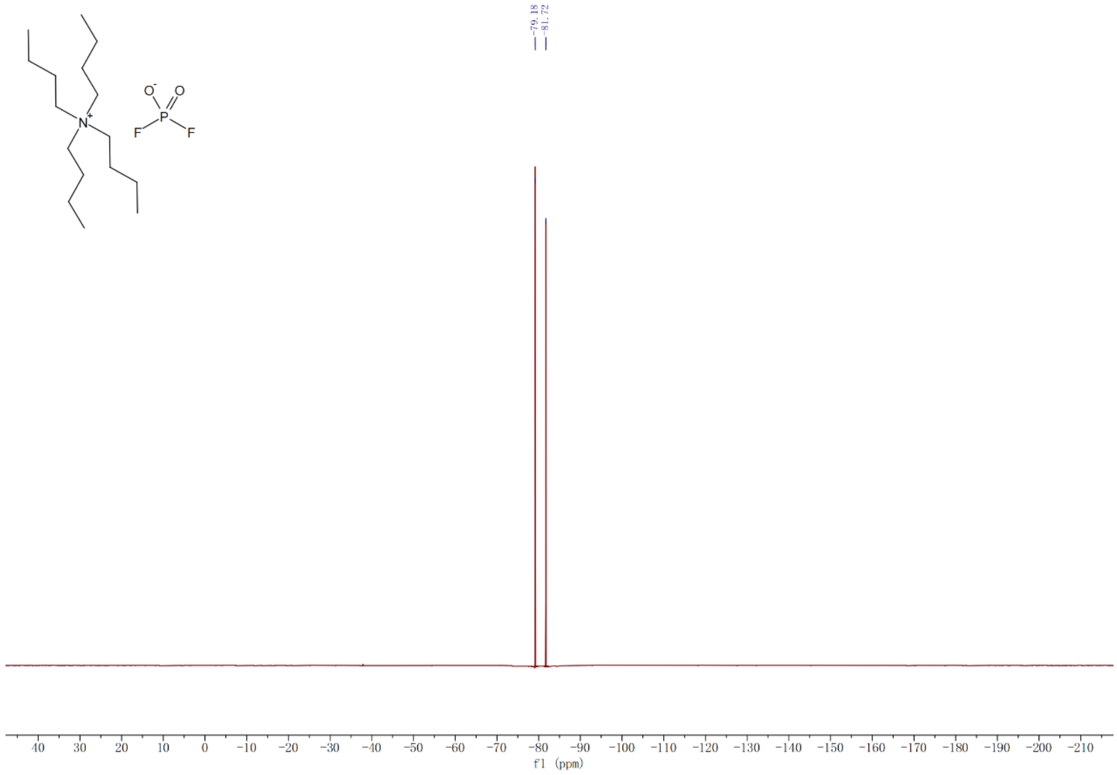

[13] C NMR (600 MHz, $CDCl_3$)

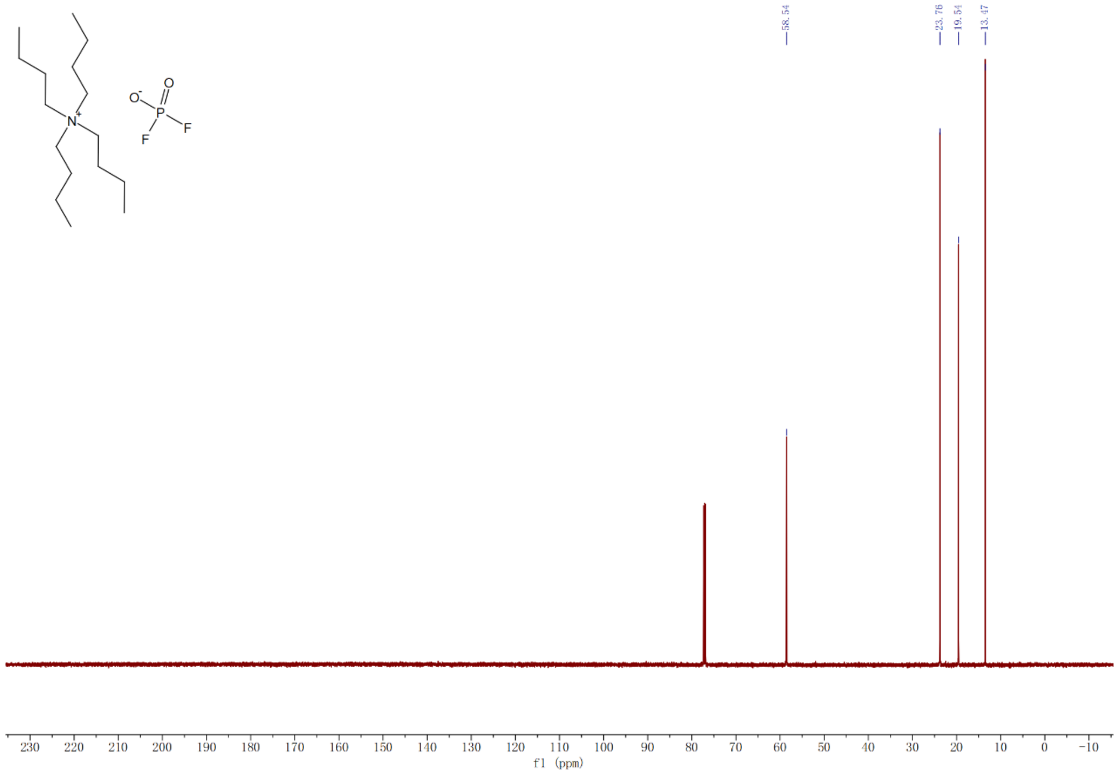

[1] H NMR (400 MHz, $CDCl_3$)

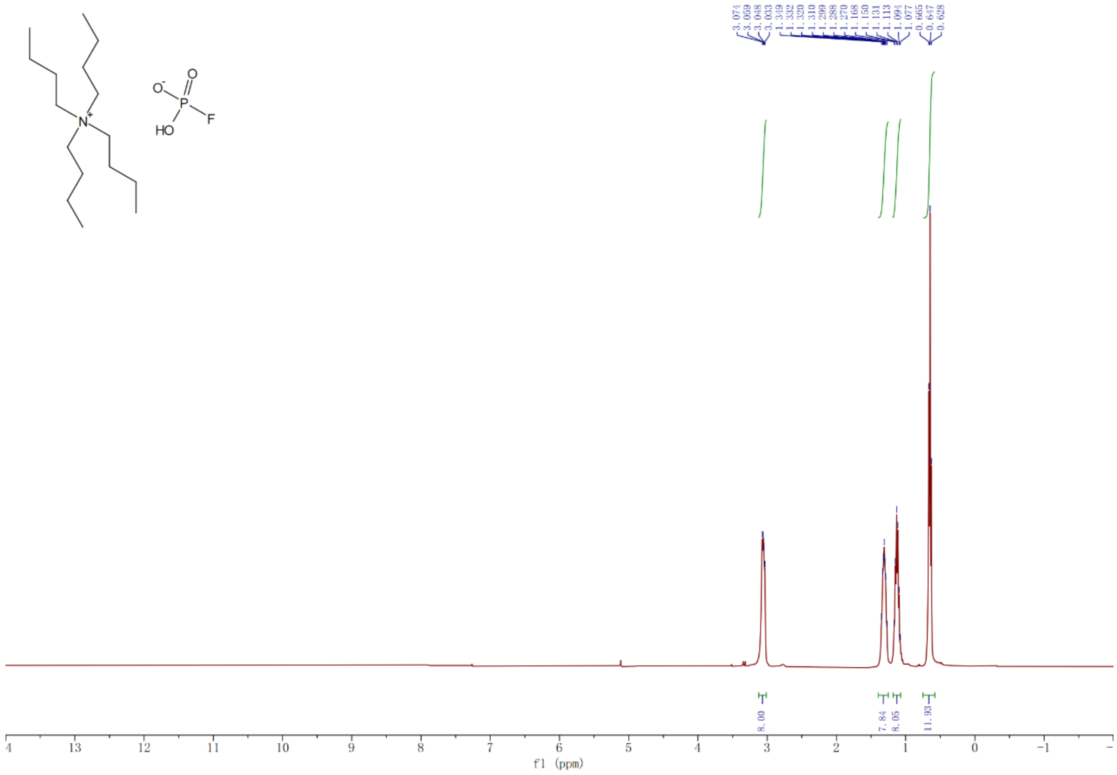

^{31}P NMR (400 MHz, $CDCl_3$)

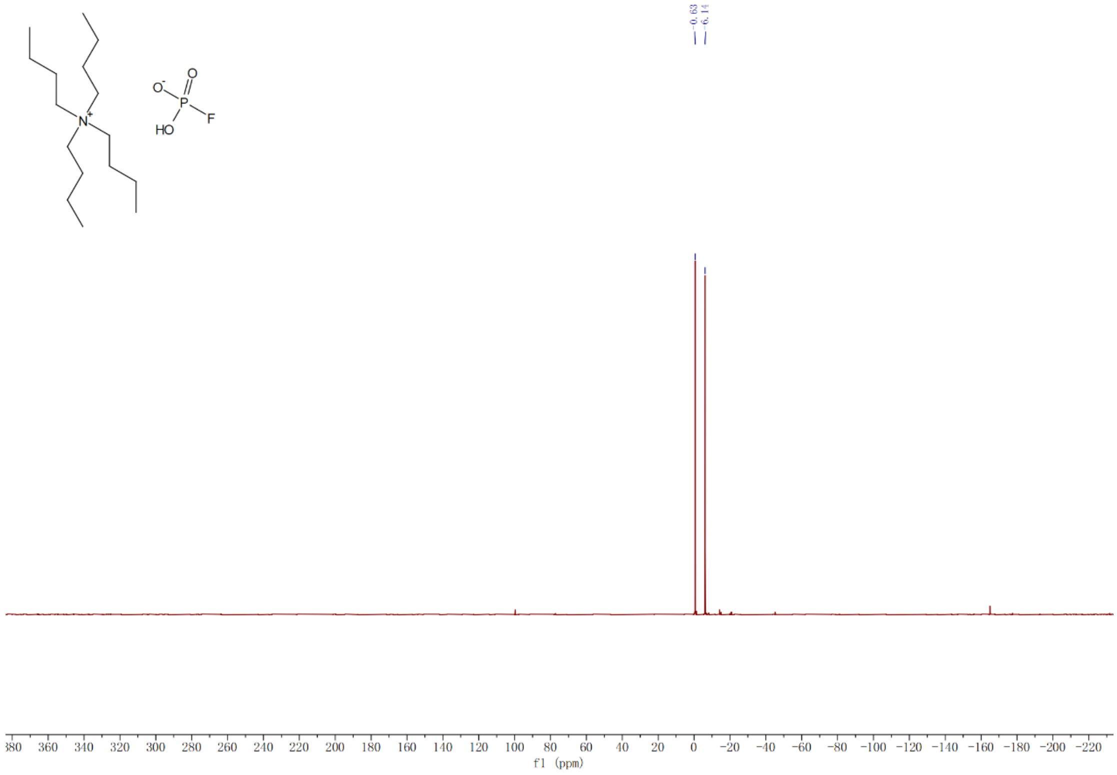

^{13}C NMR (600 MHz, $CDCl_3$)

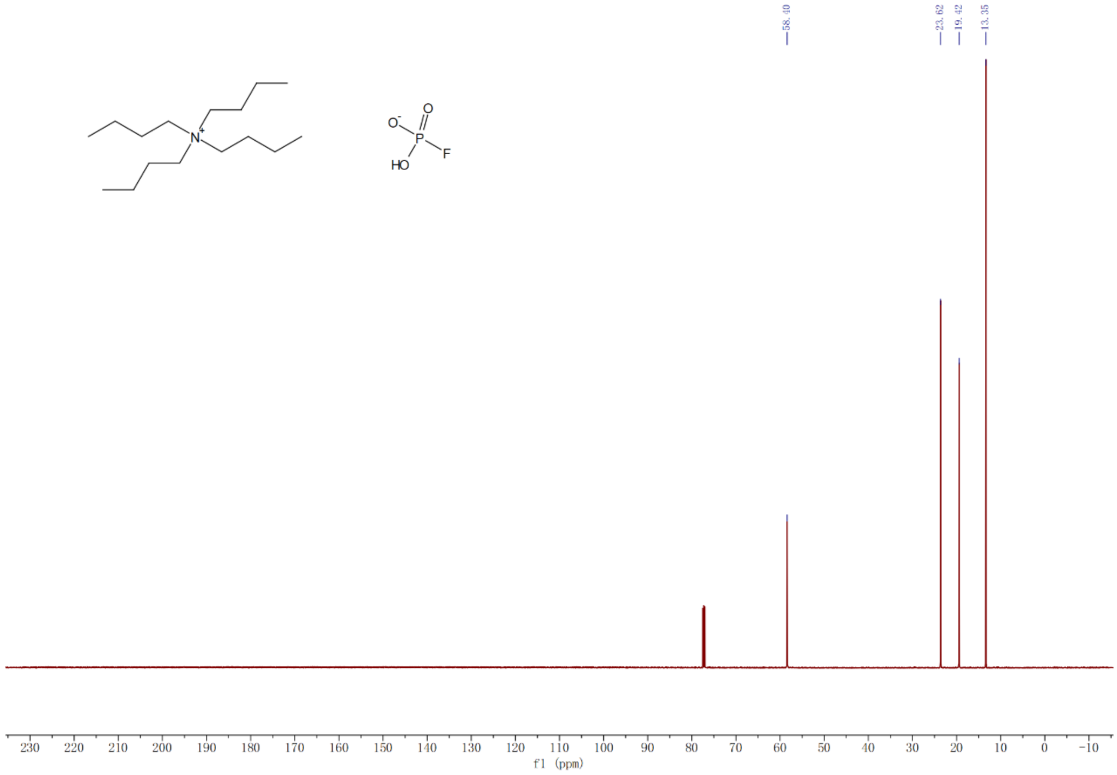

^{1}H NMR (400 MHz, CDCl$_3$)

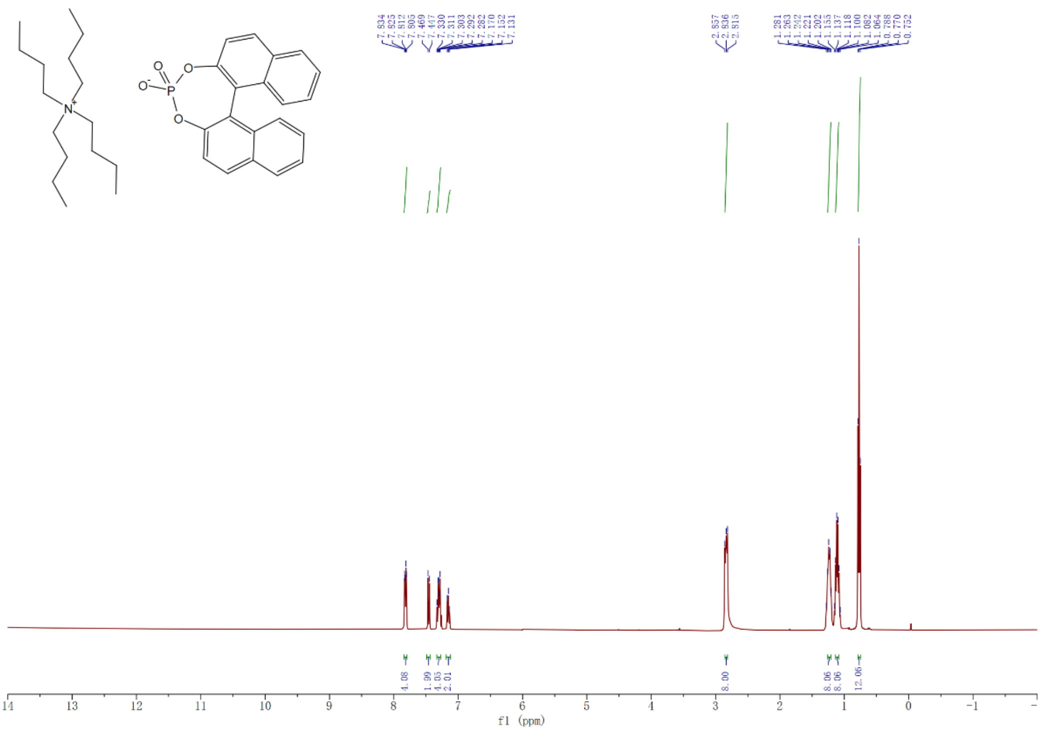

^{31}P NMR (400 MHz, CDCl$_3$)

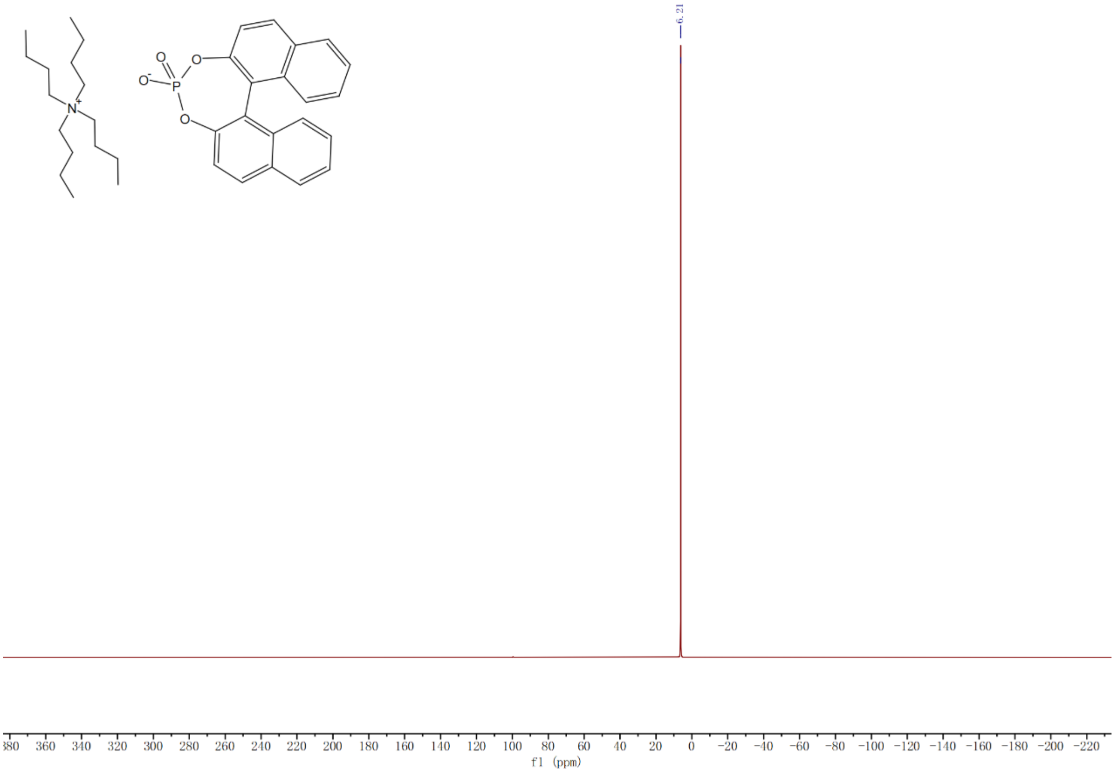

^{13}C NMR (600 MHz, $CDCl_3$)

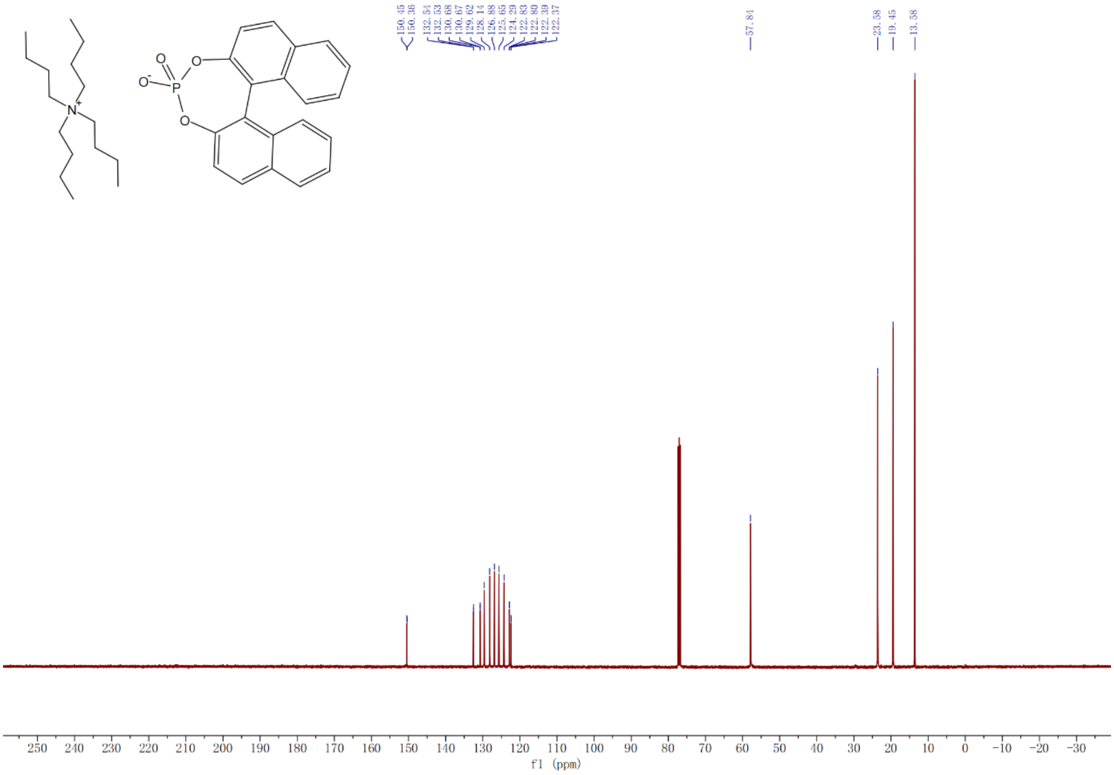

^{1}H NMR (400 MHz, DMSO-D_6)

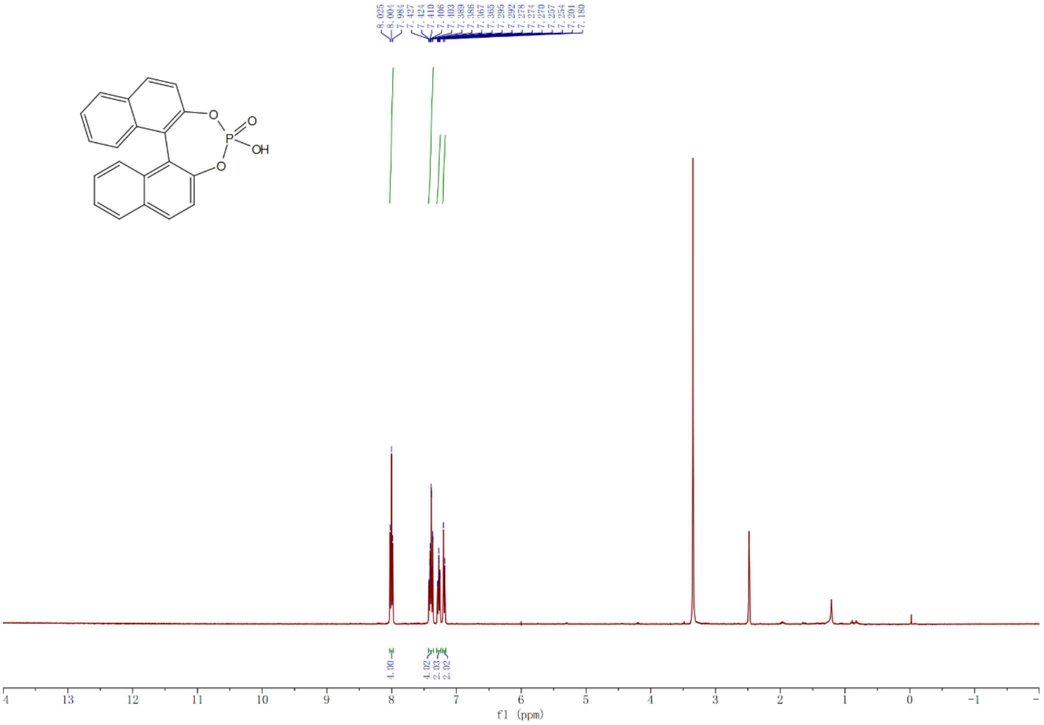

^{31}P NMR (400 MHz, DMSO-D$_6$)

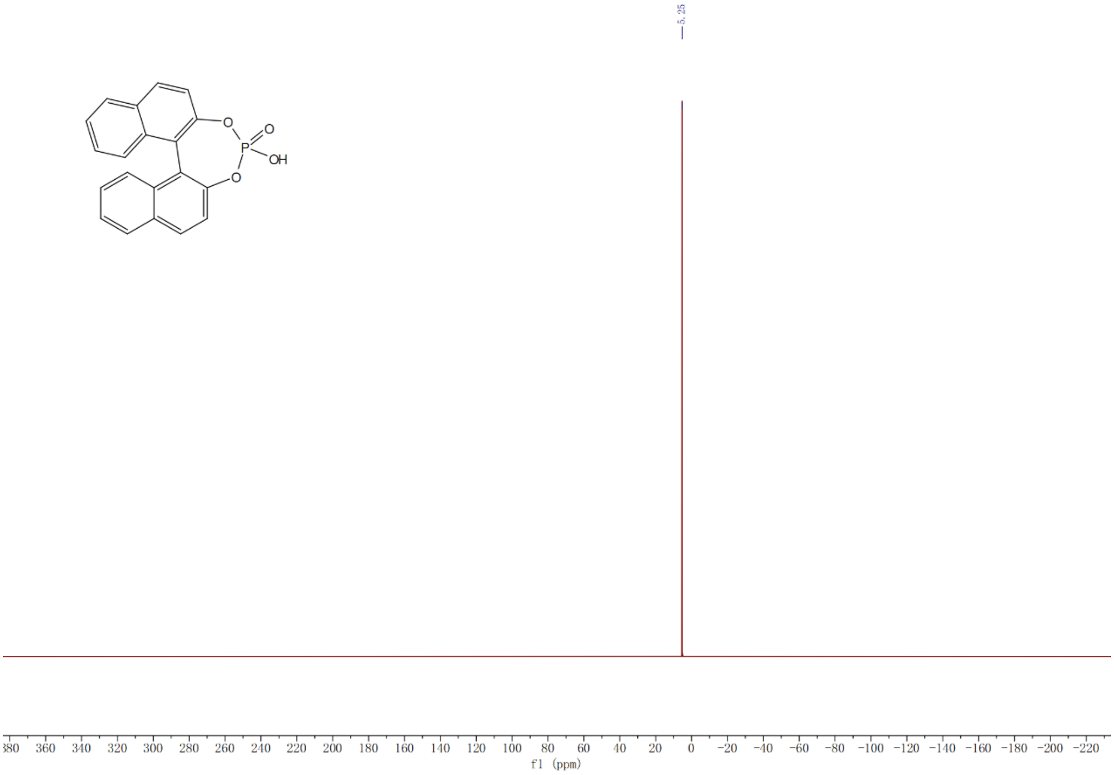

^{13}C NMR (600 MHz, DMSO-D$_6$)

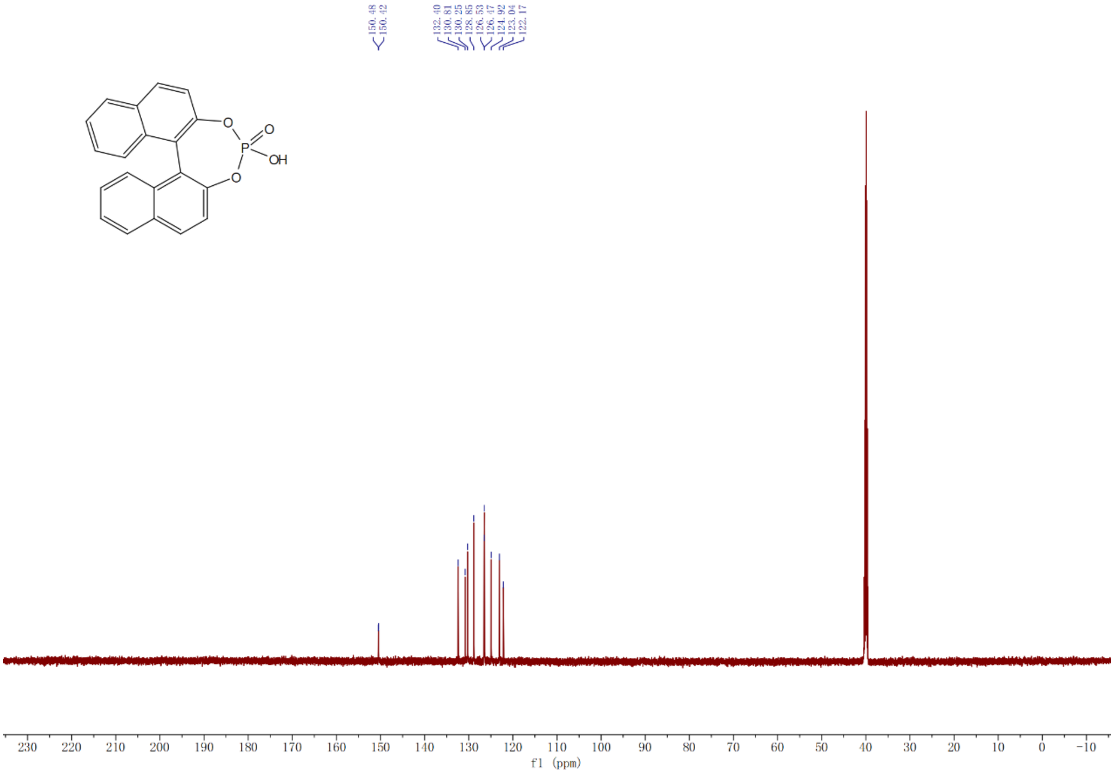

^{1}H NMR (400 MHz, $CDCl_3$)

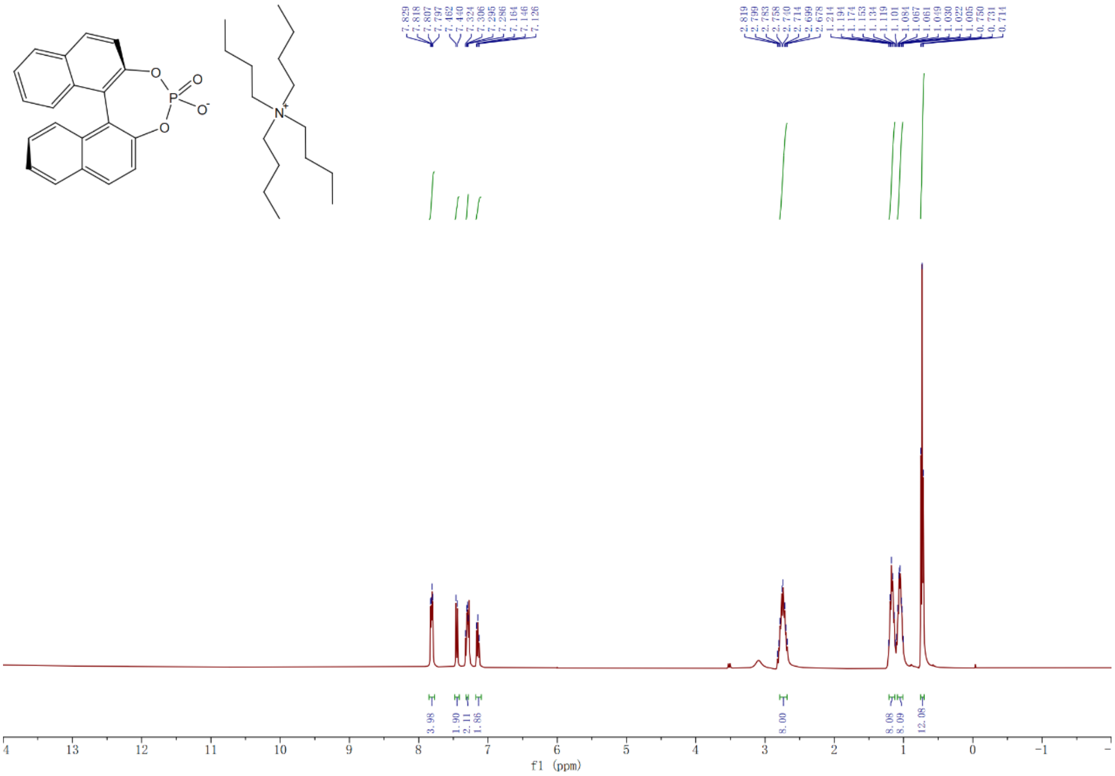

^{31}P NMR (400 MHz, $CDCl_3$)

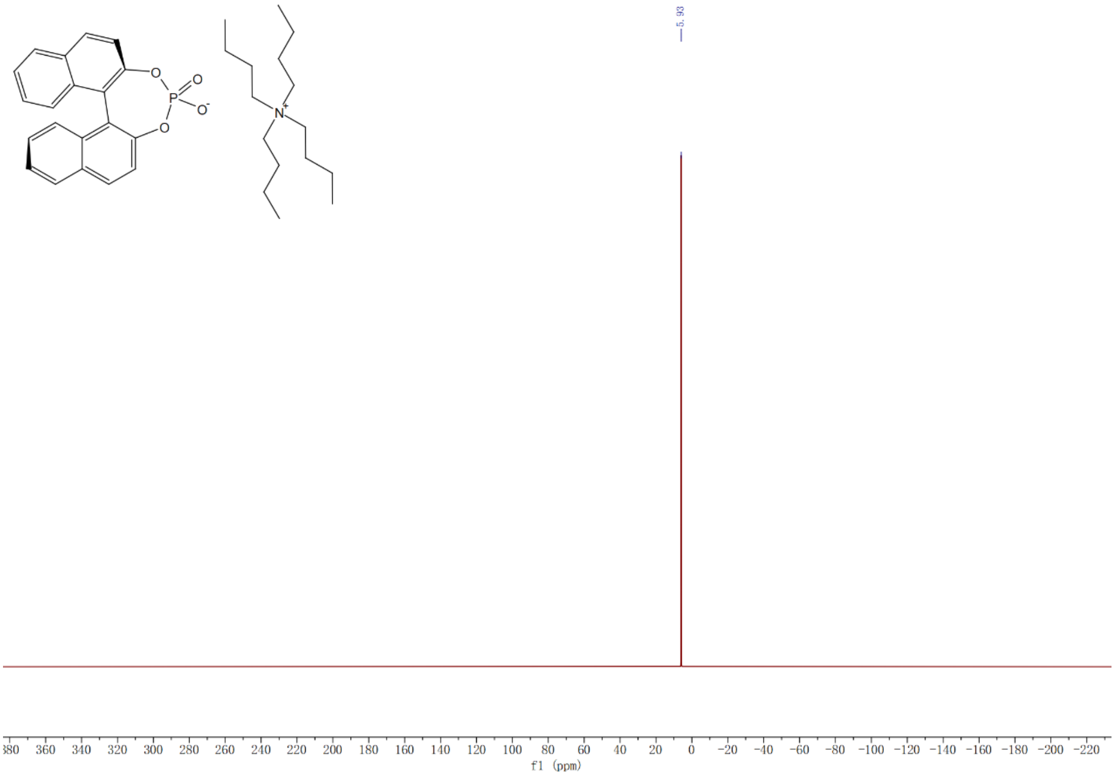

^{13}C NMR (600 MHz, $CDCl_3$)

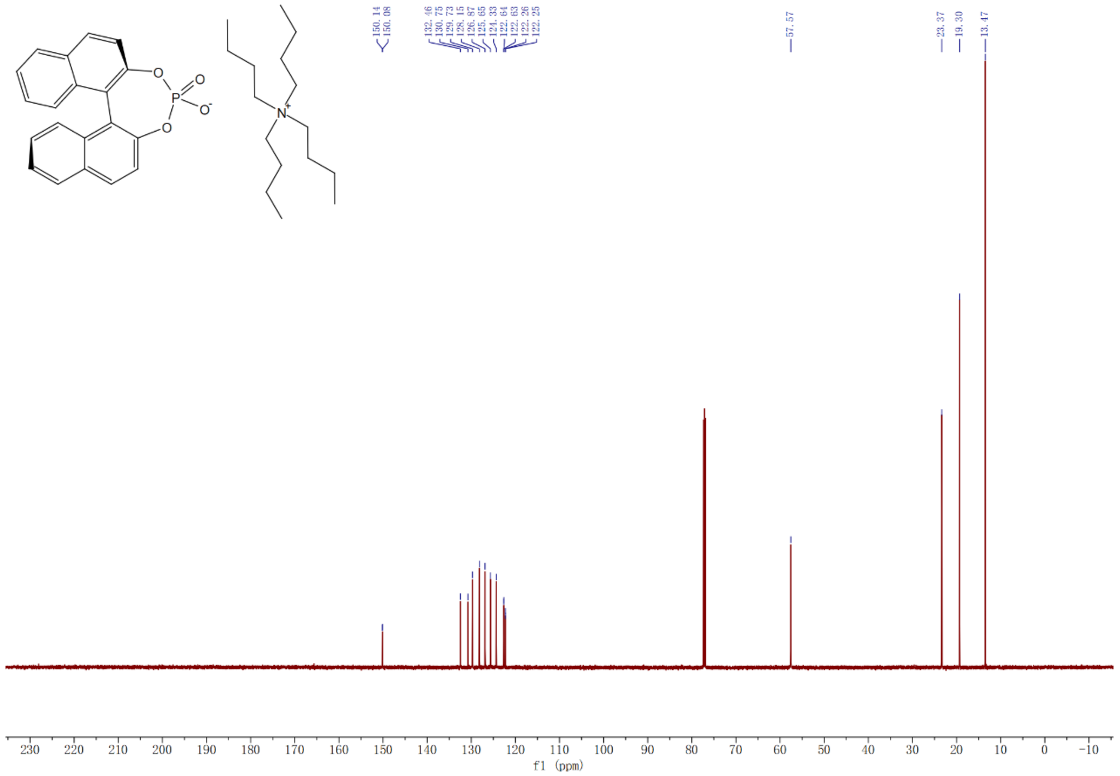

^{1}H NMR (400 MHz, $CDCl_3$)

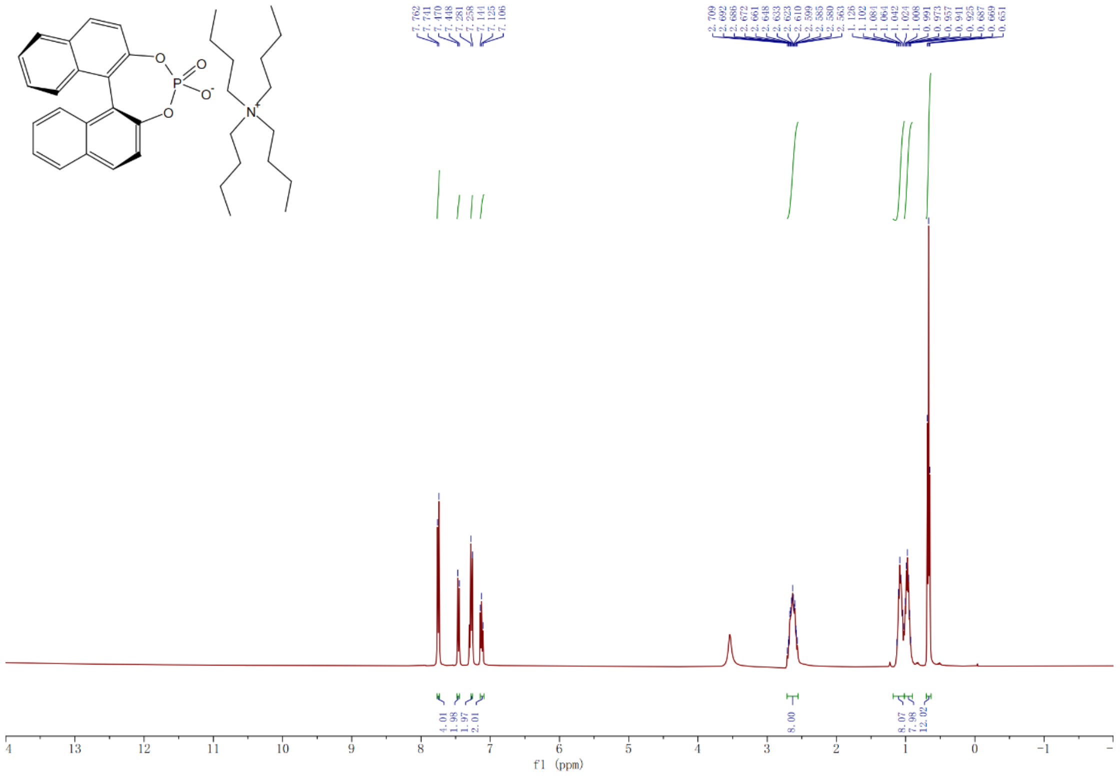

^{31}P NMR (400 MHz, CDCl$_3$)

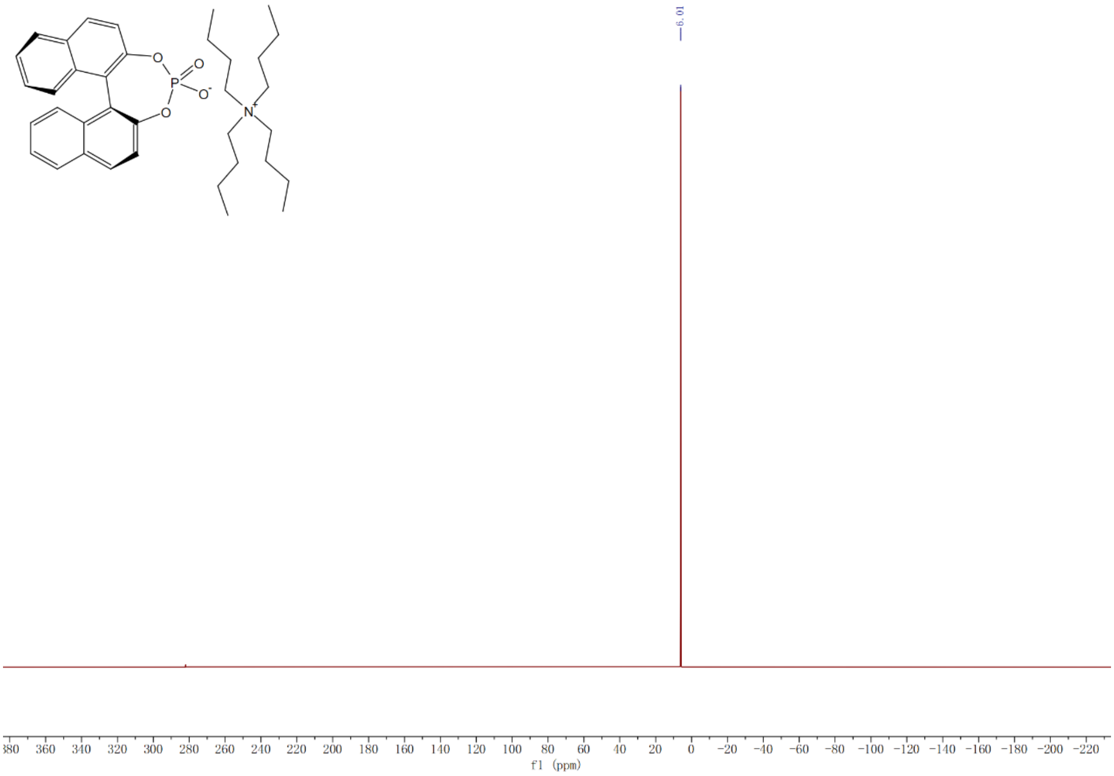

^{13}C NMR (600 MHz, CDCl$_3$)

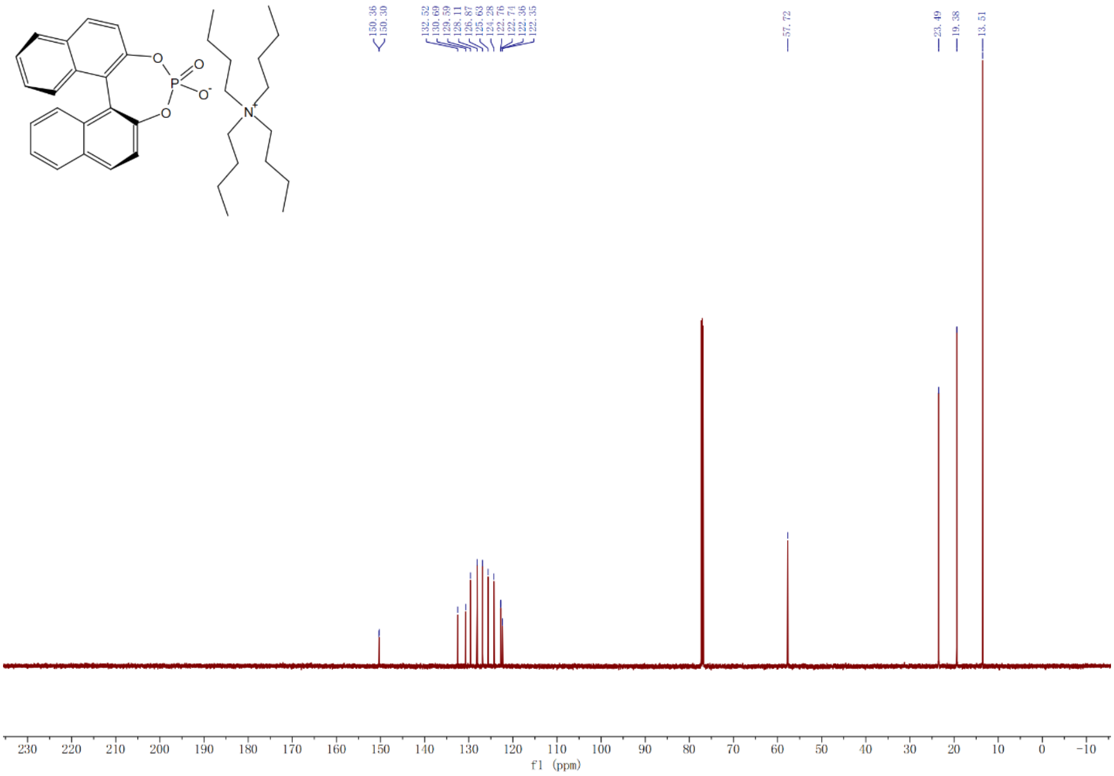

^{1}H NMR (400 MHz, $CDCl_3$)

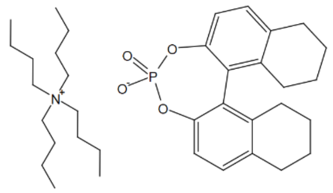

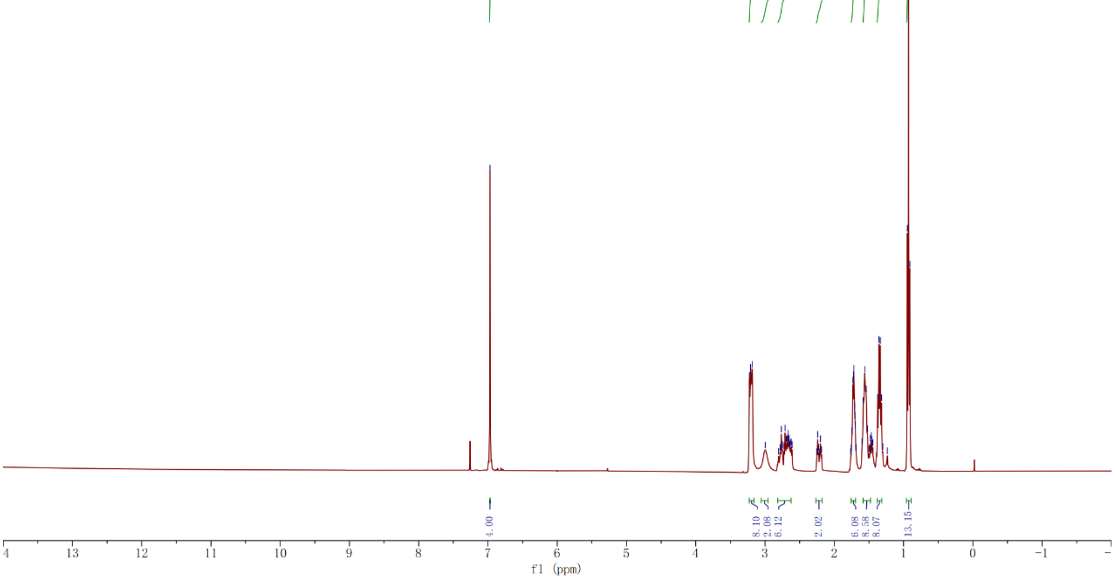

^{31}P NMR (400 MHz, $CDCl_3$)

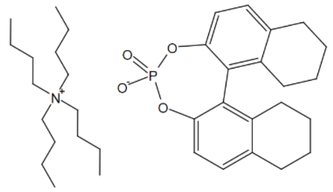

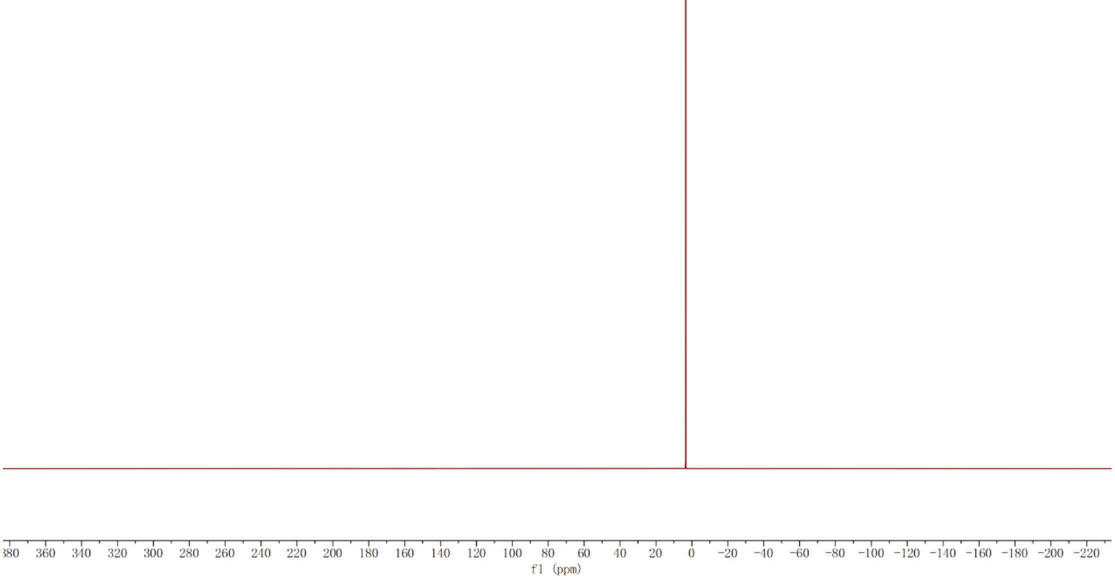

^{13}C NMR (600 MHz, $CDCl_3$)

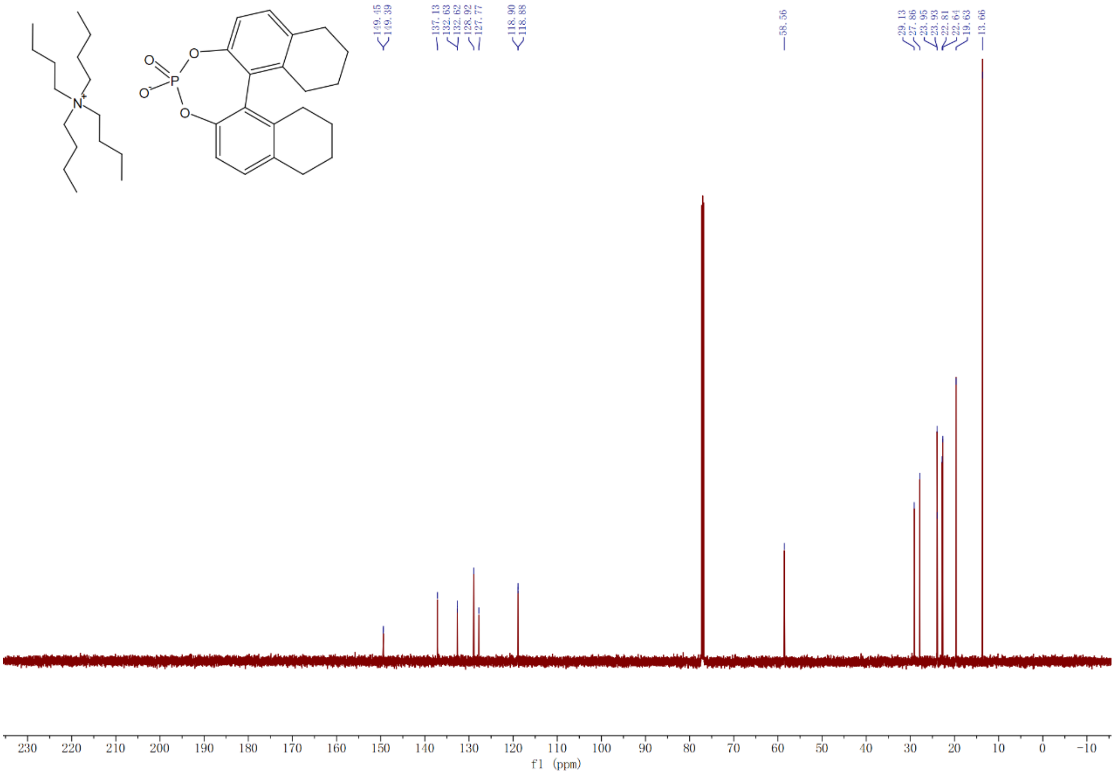

^{1}H NMR (400 MHz, DMSO-D_6)

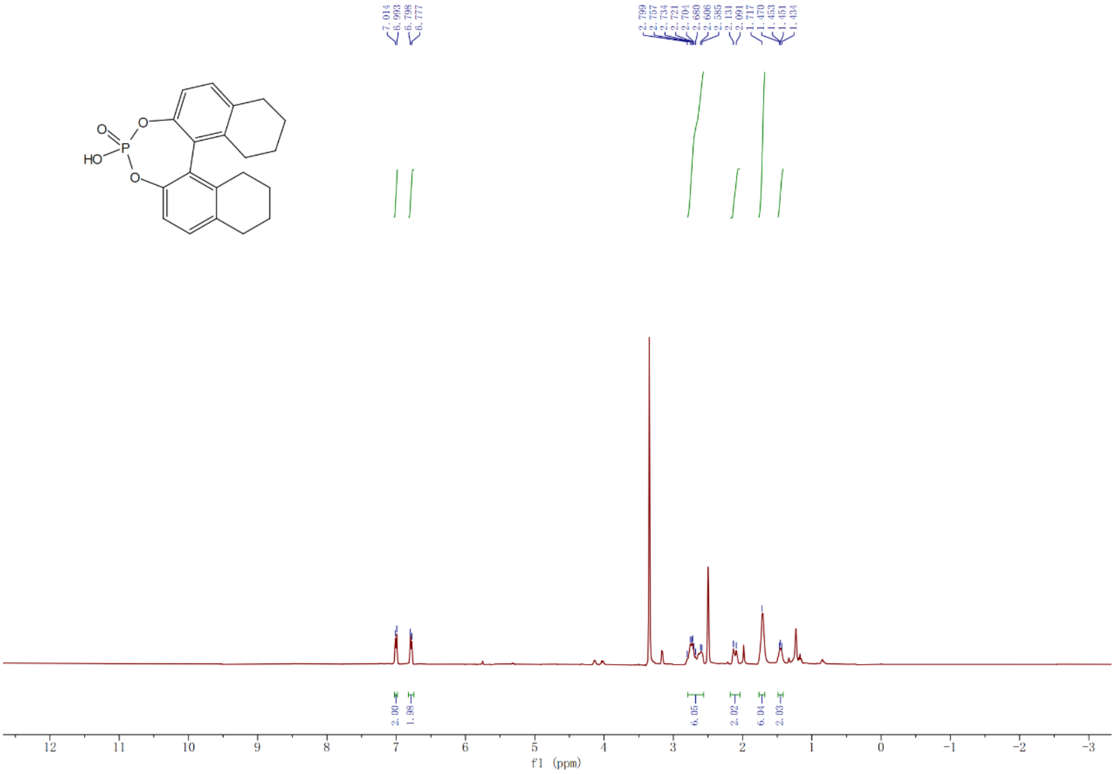

^{31}P NMR (400 MHz, DMSO-D$_6$))

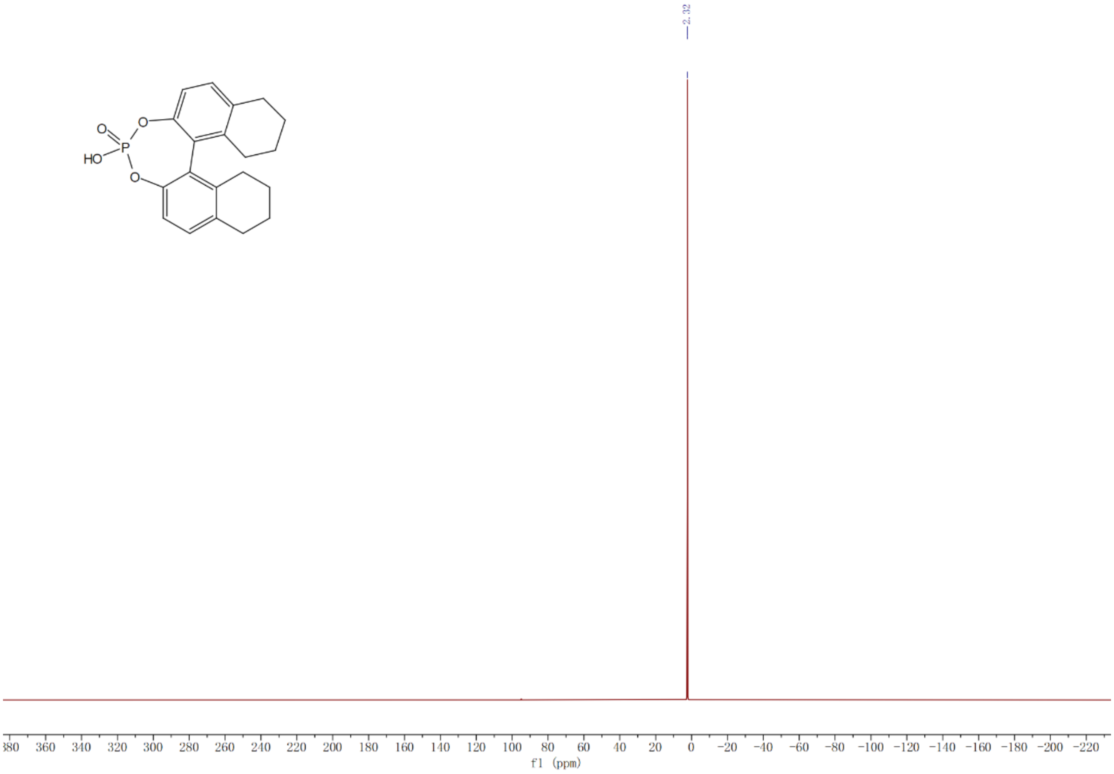

^{13}C NMR (600 MHz, DMSO-D$_6$)

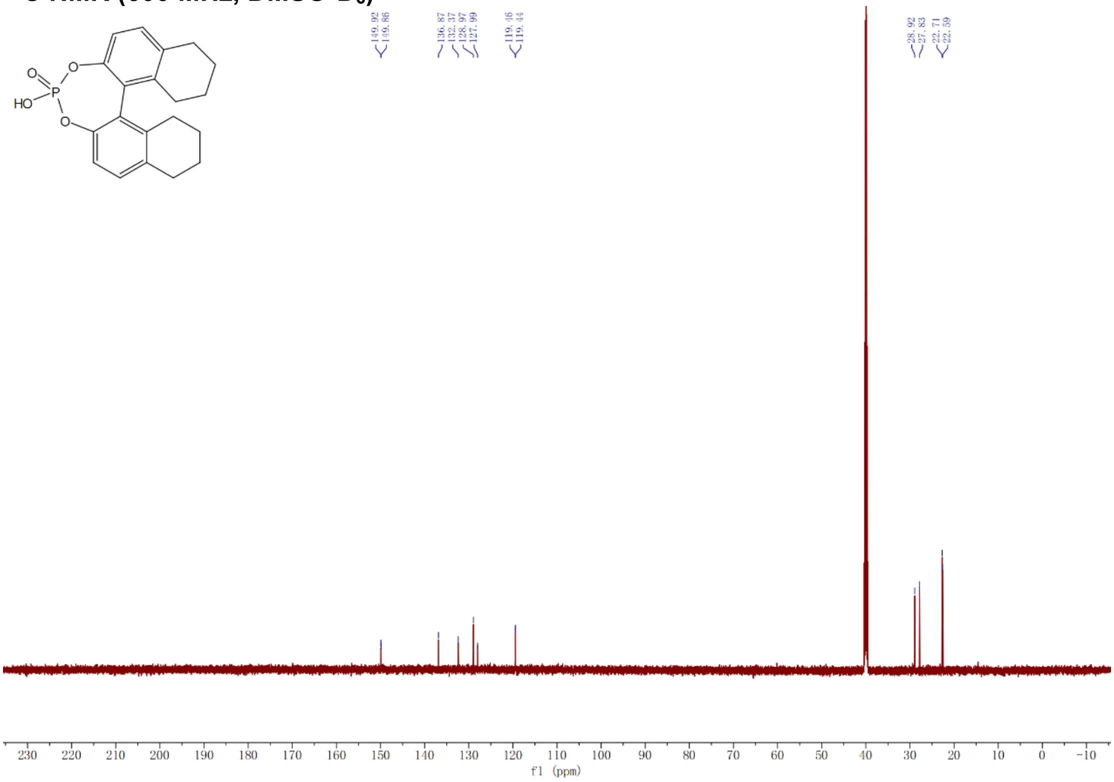

^{1}H NMR (400 MHz, $CDCl_3$)

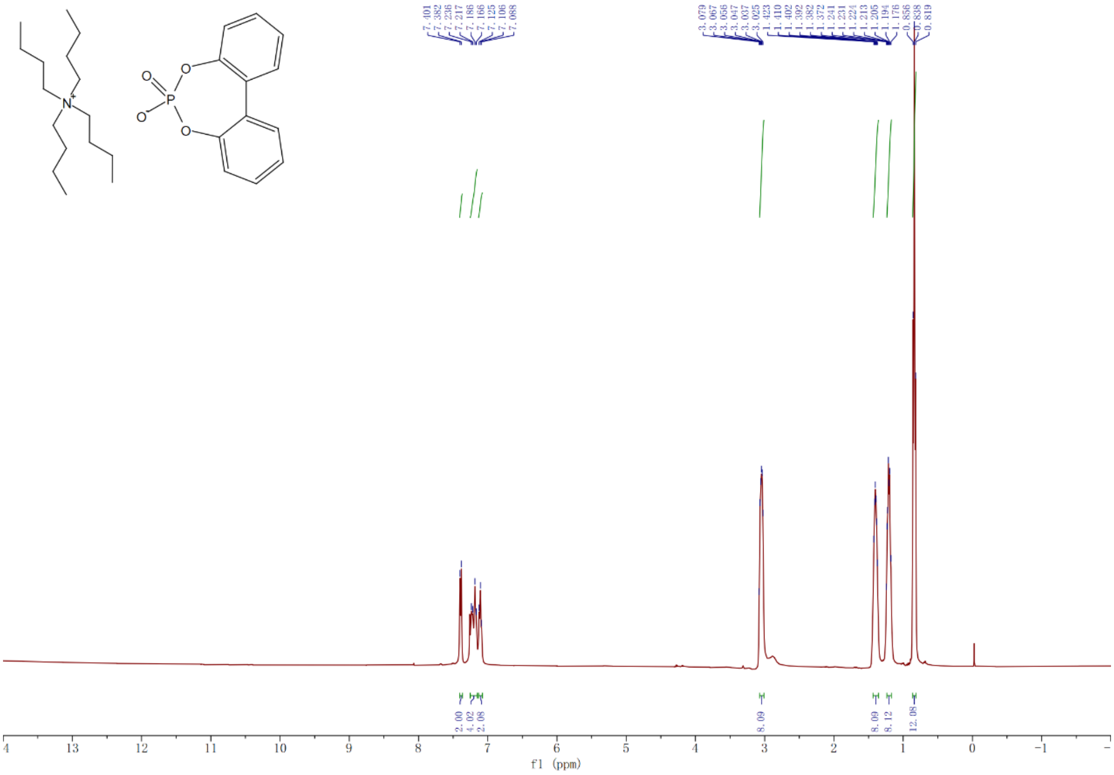

^{31}P NMR (400 MHz, $CDCl_3$)

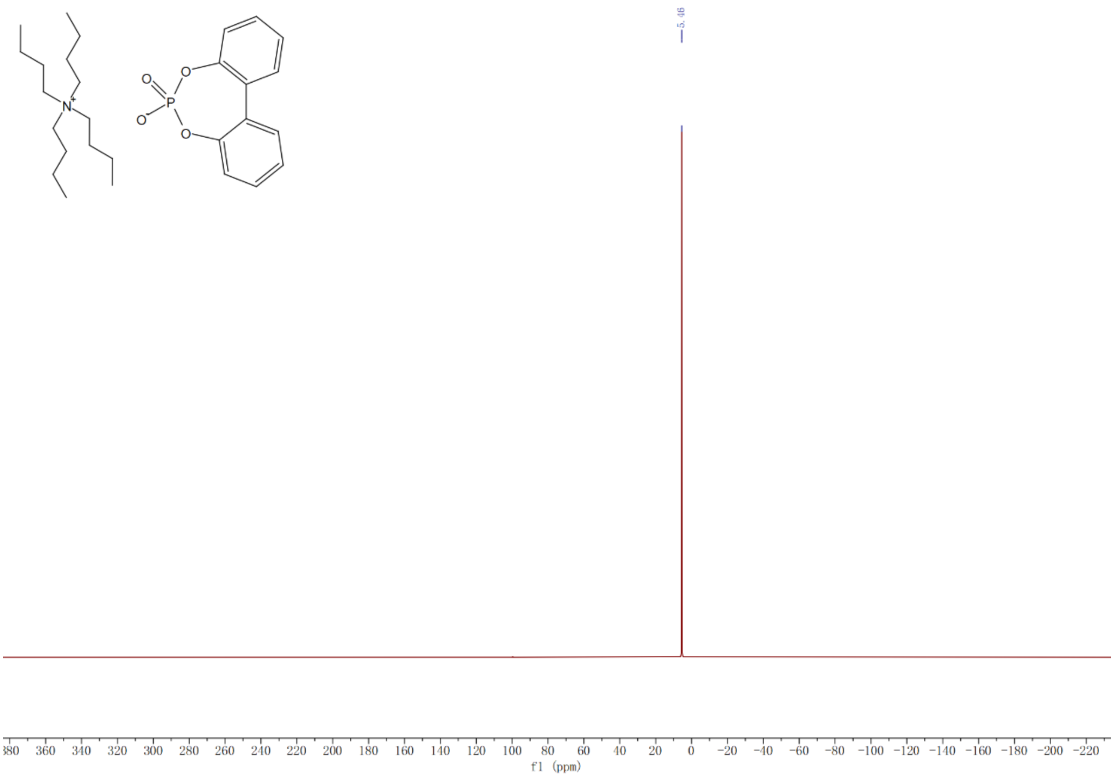

^{13}C NMR (600 MHz, $CDCl_3$)

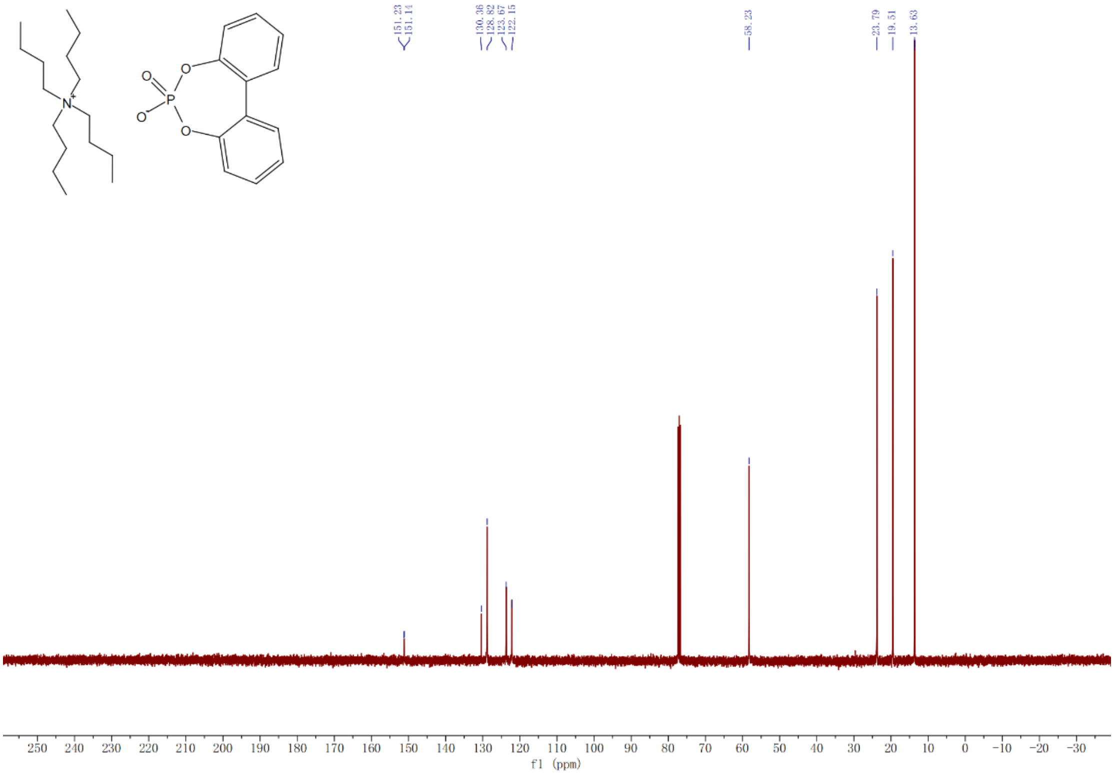

^{1}H NMR (400 MHz, DMSO-D_6)

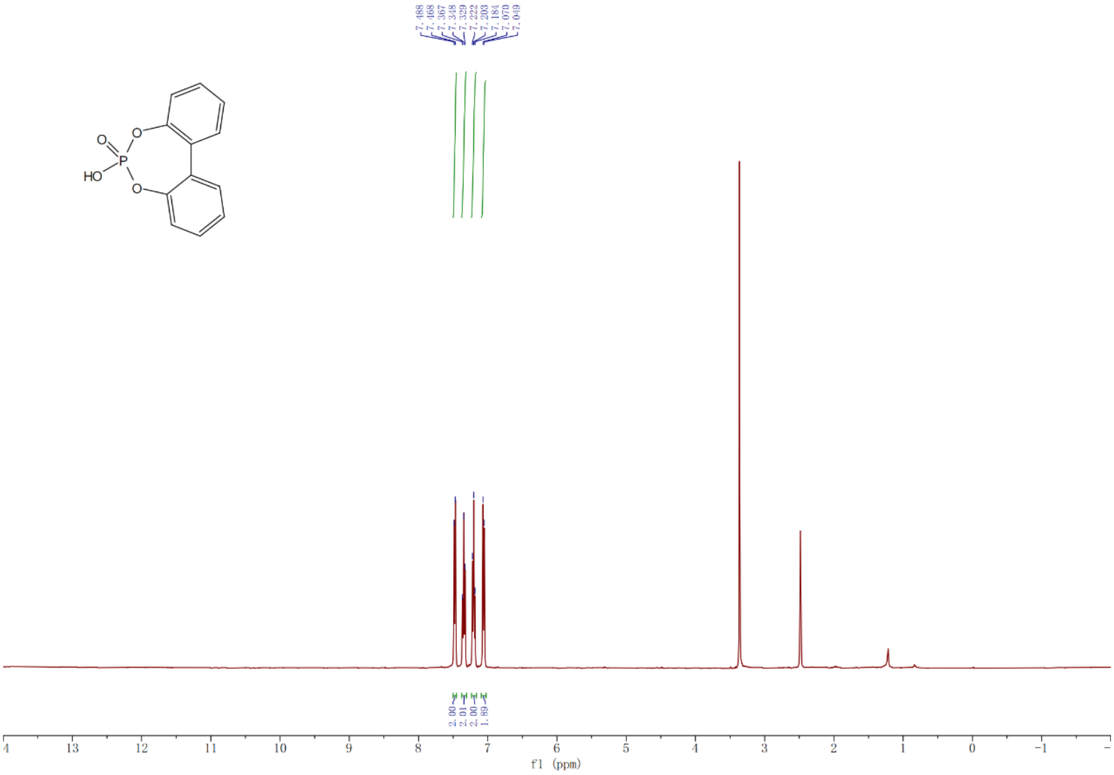

^{31}P NMR (400 MHz, DMSO-D$_6$)

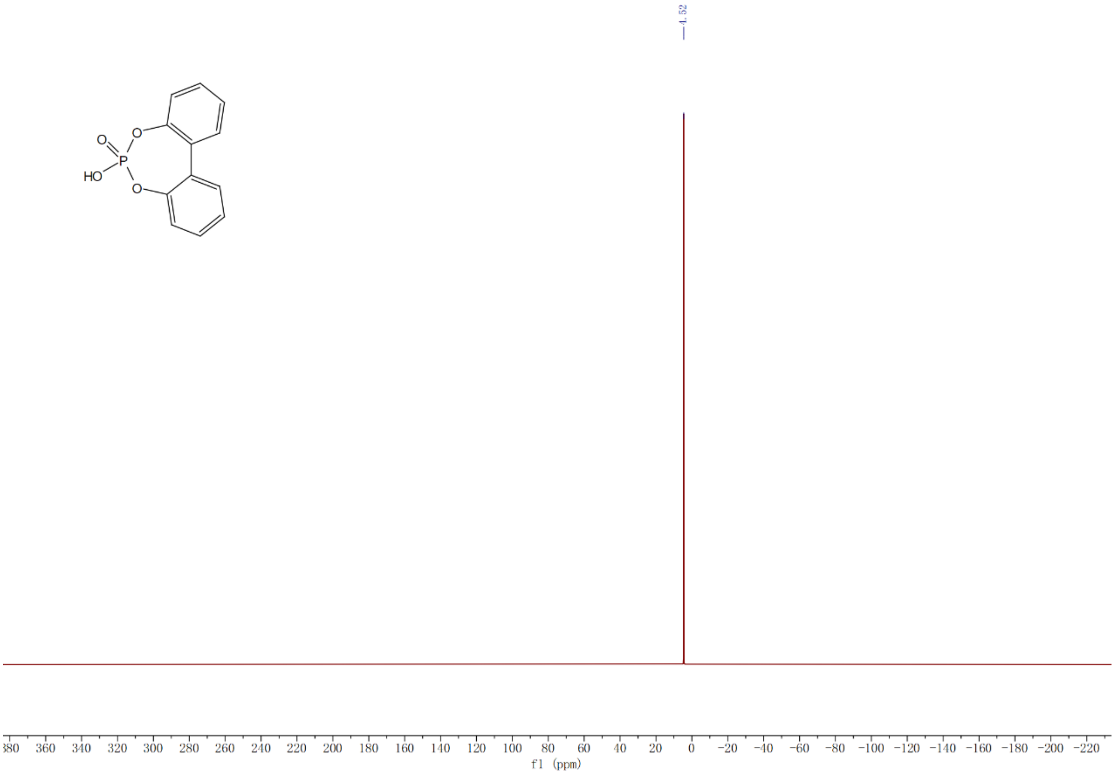

^{13}C NMR (600 MHz, DMSO-D$_6$)

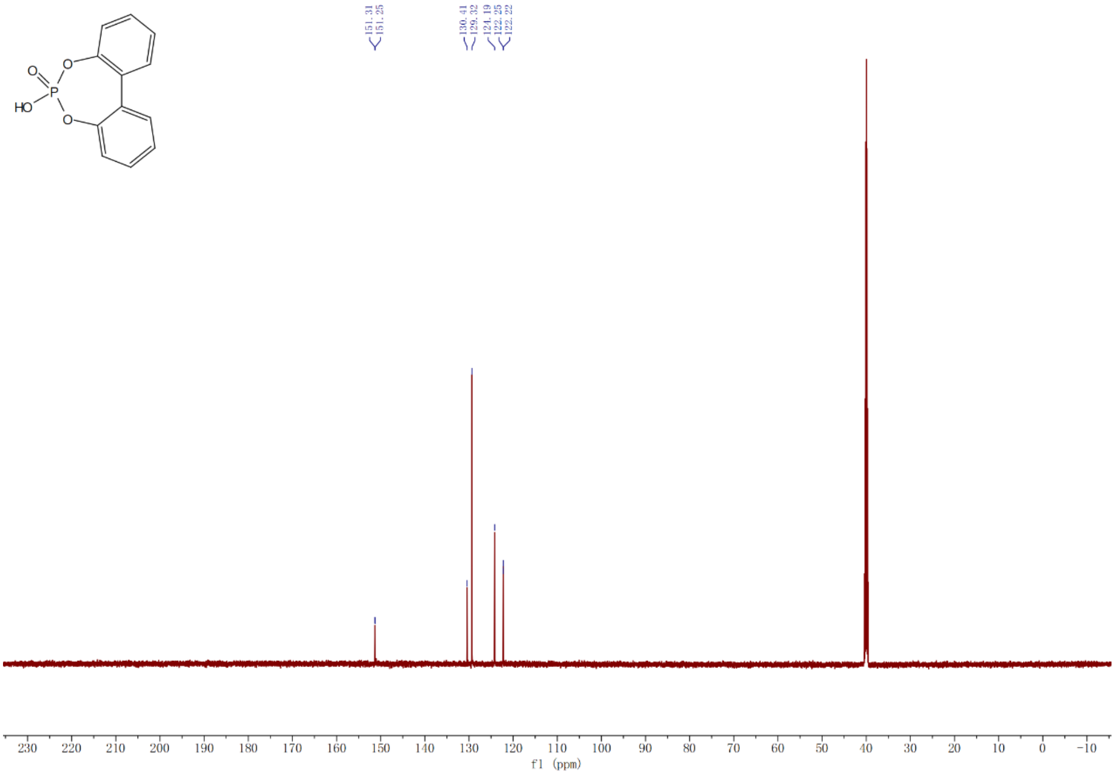

^{1}H NMR (400 MHz, $CDCl_3$)

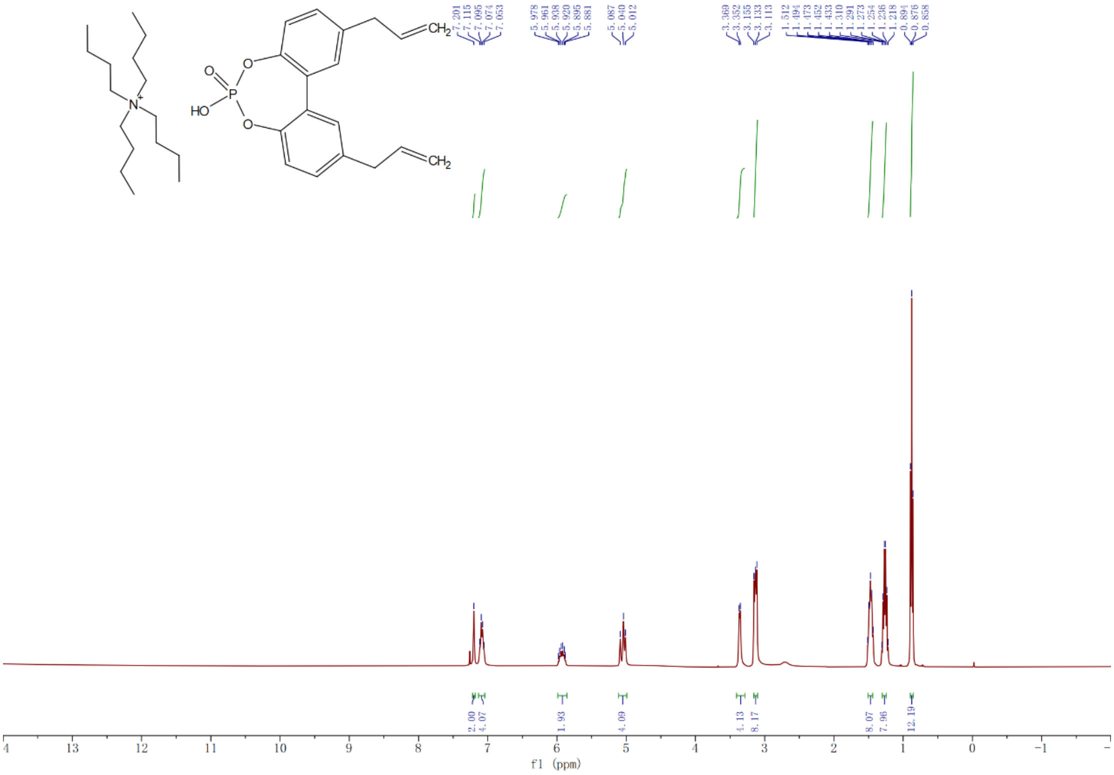

^{31}P NMR (400 MHz, $CDCl_3$)

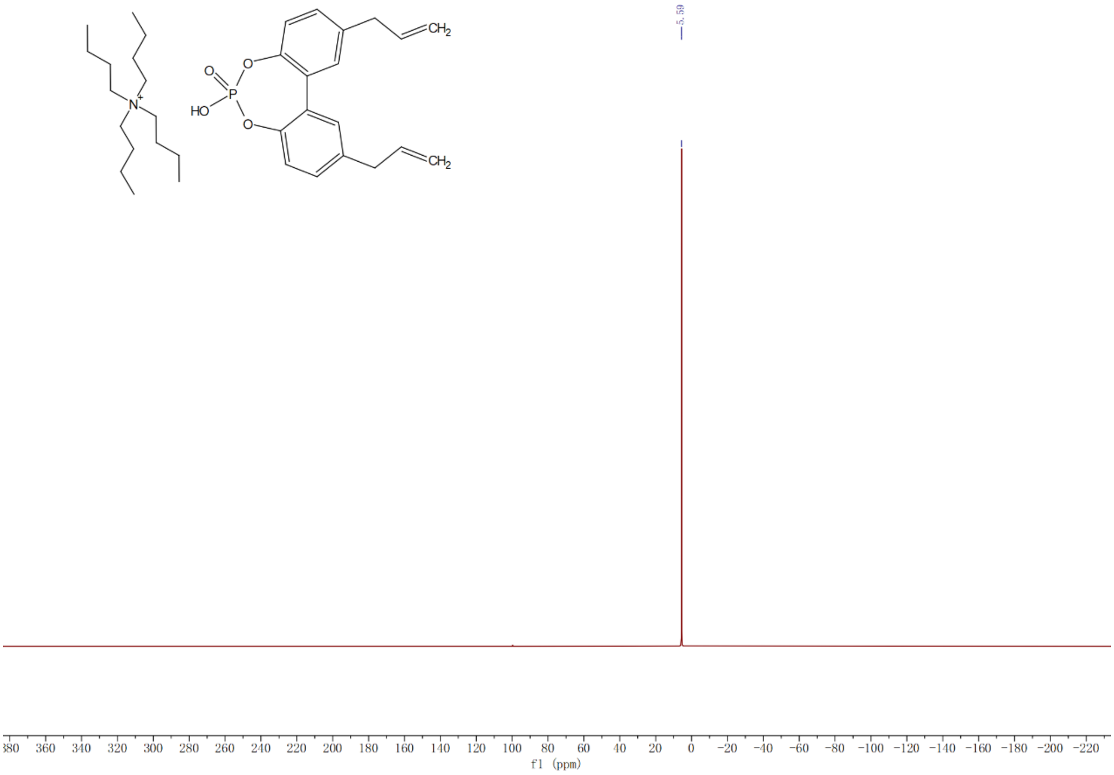

^{13}C NMR (600 MHz, CDCl$_3$)

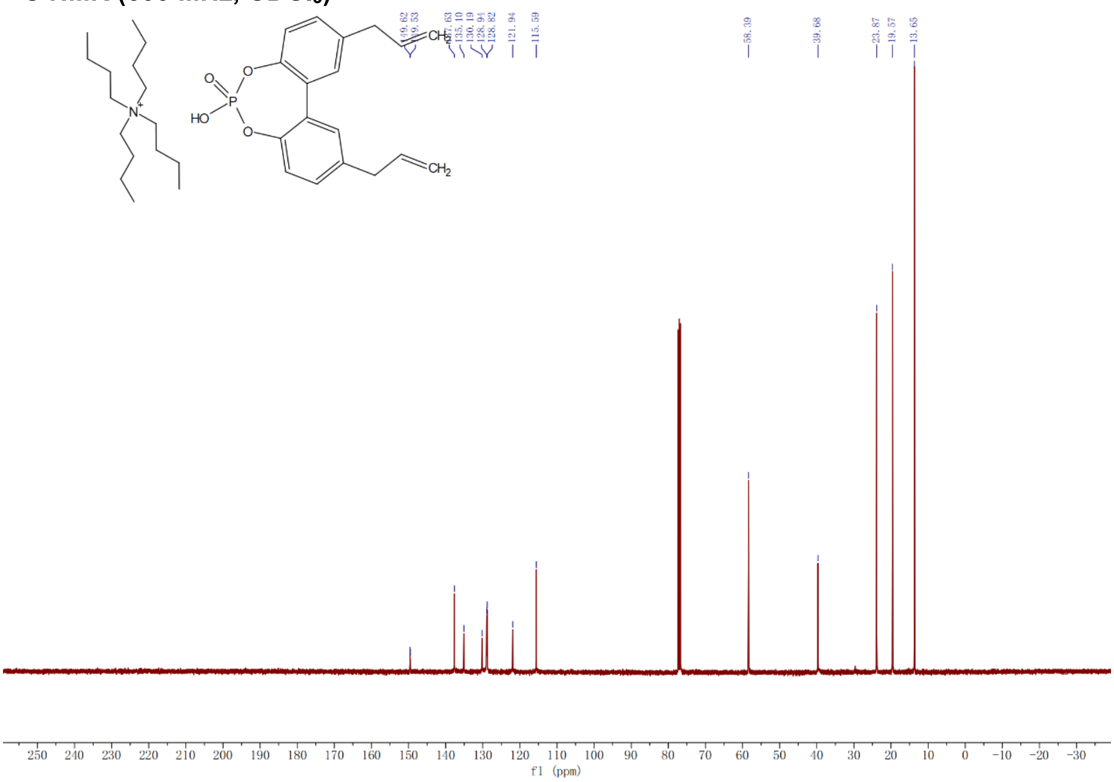

^{1}H NMR (400 MHz, DMSO-D$_6$)

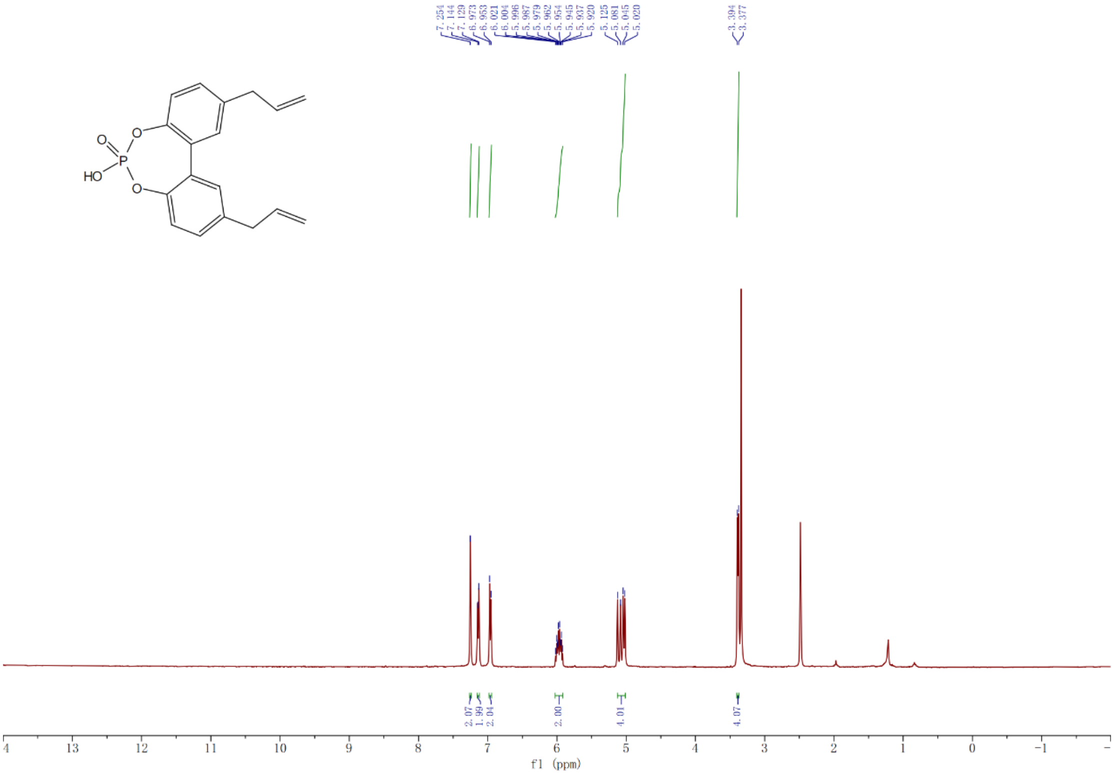

^{31}P NMR (400 MHz, DMSO-D$_6$)

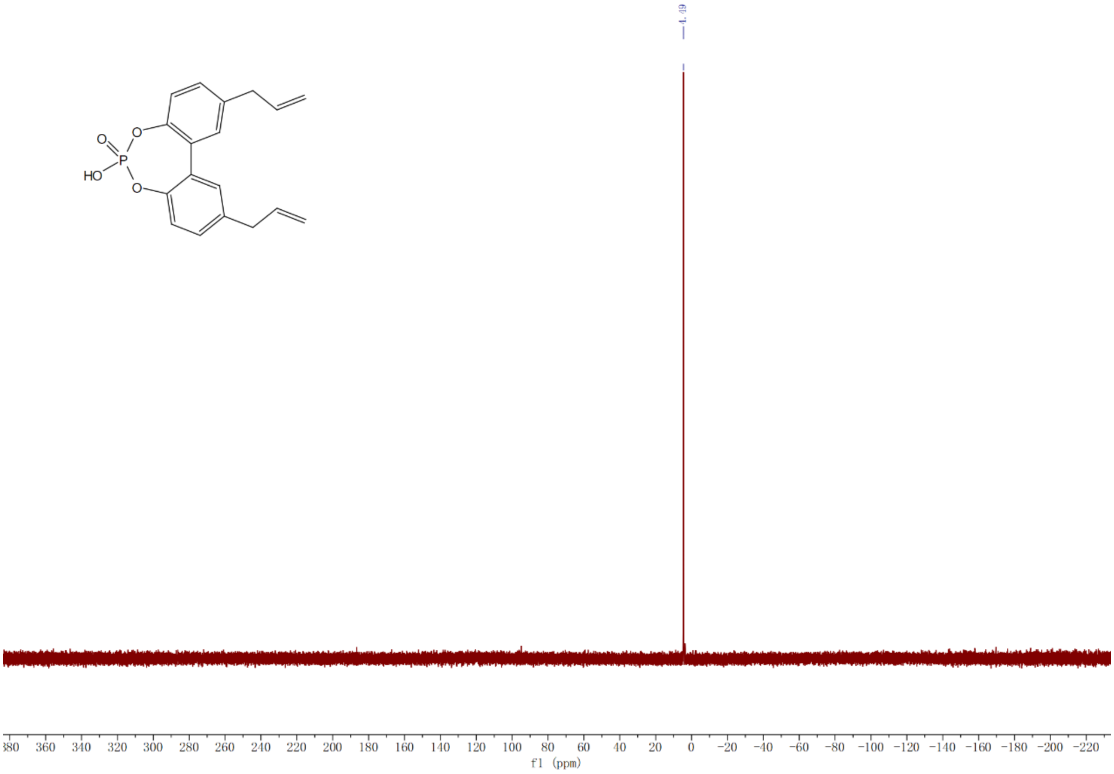

^{13}C NMR (600 MHz, DMSO-D$_6$)

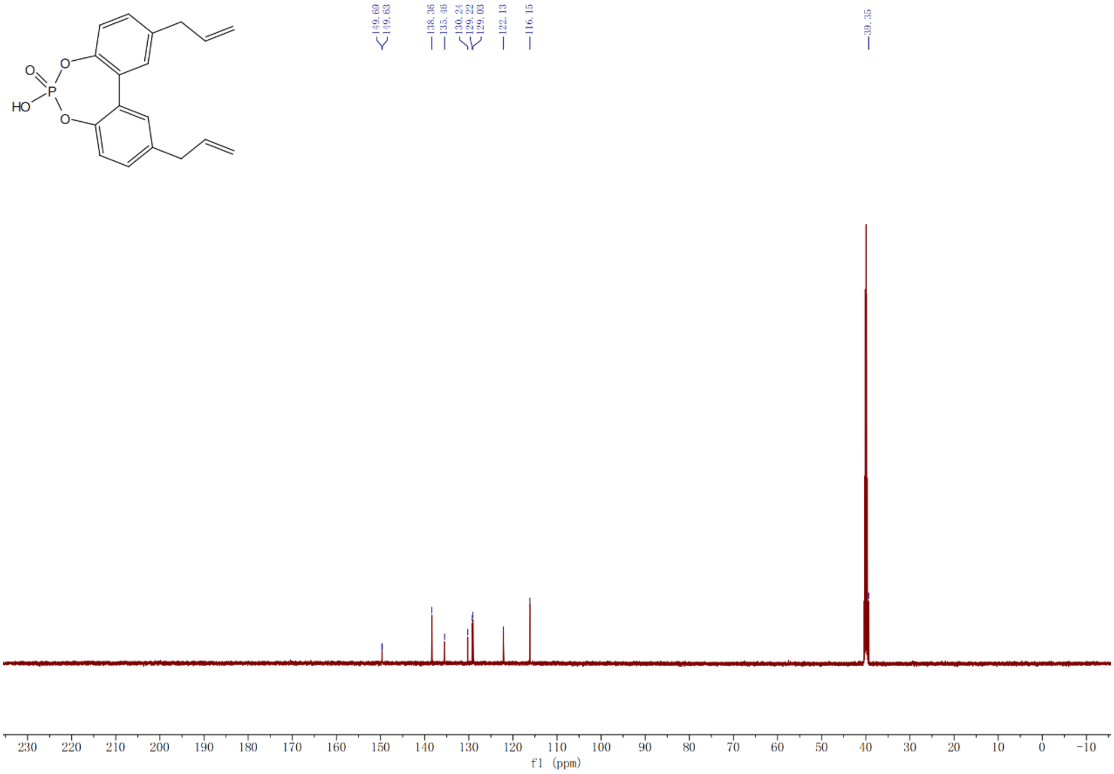

^1H NMR (400 MHz, $CDCl_3$)

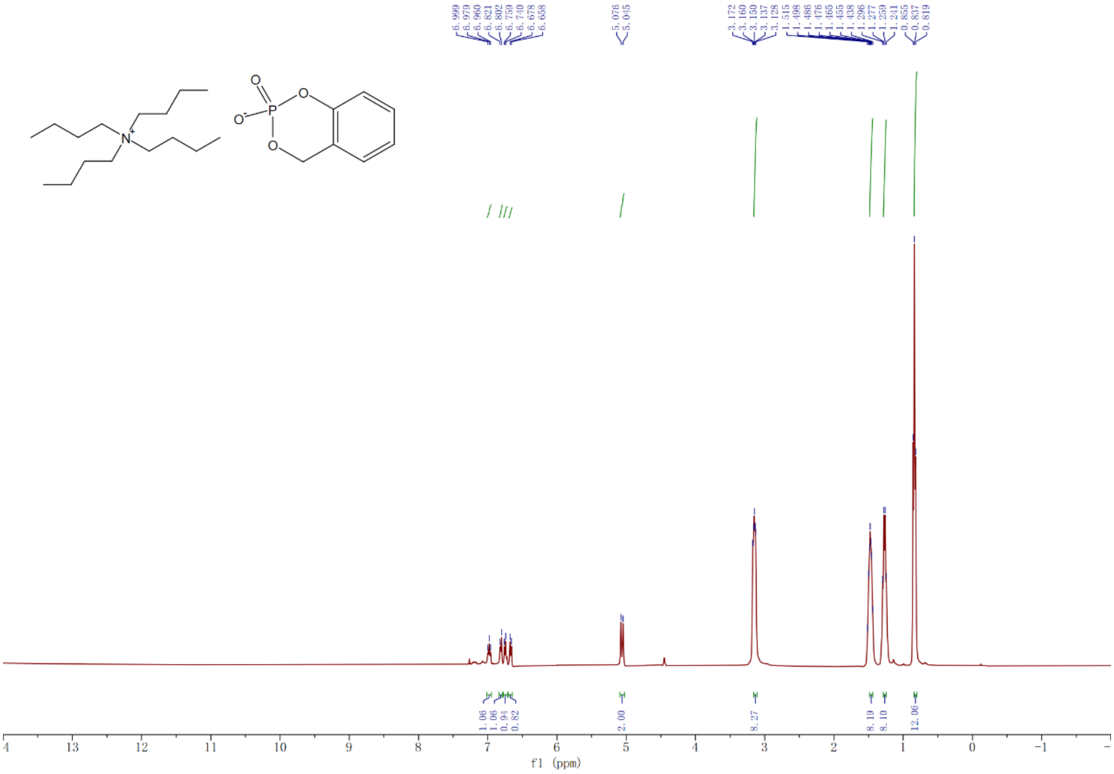

^{31}P NMR (400 MHz, $CDCl_3$)

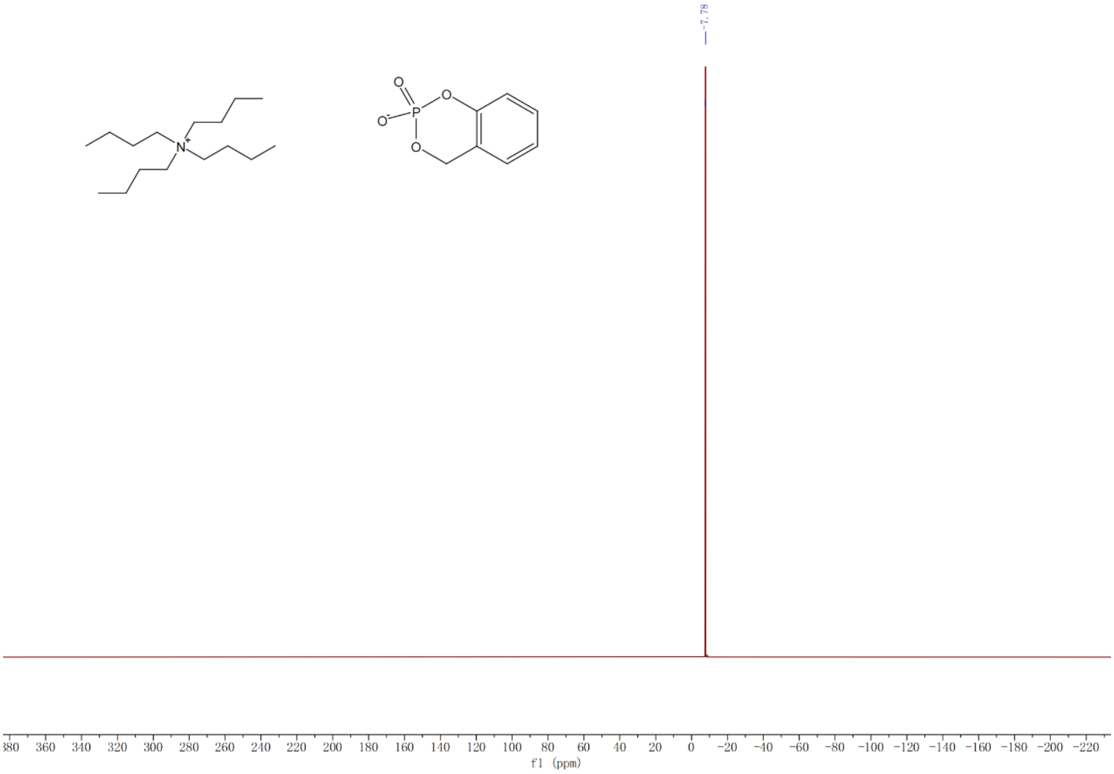

^{13}C NMR (600 MHz, $CDCl_3$)

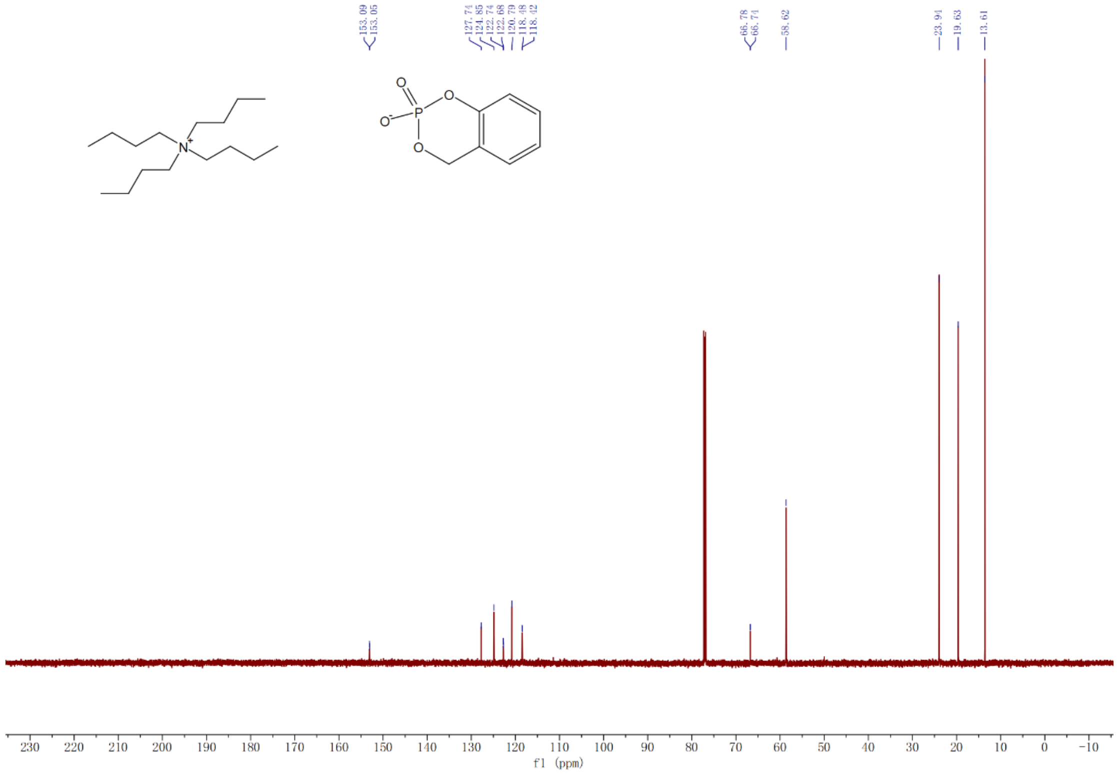

^{1}H NMR (400 MHz, $CDCl_3$)

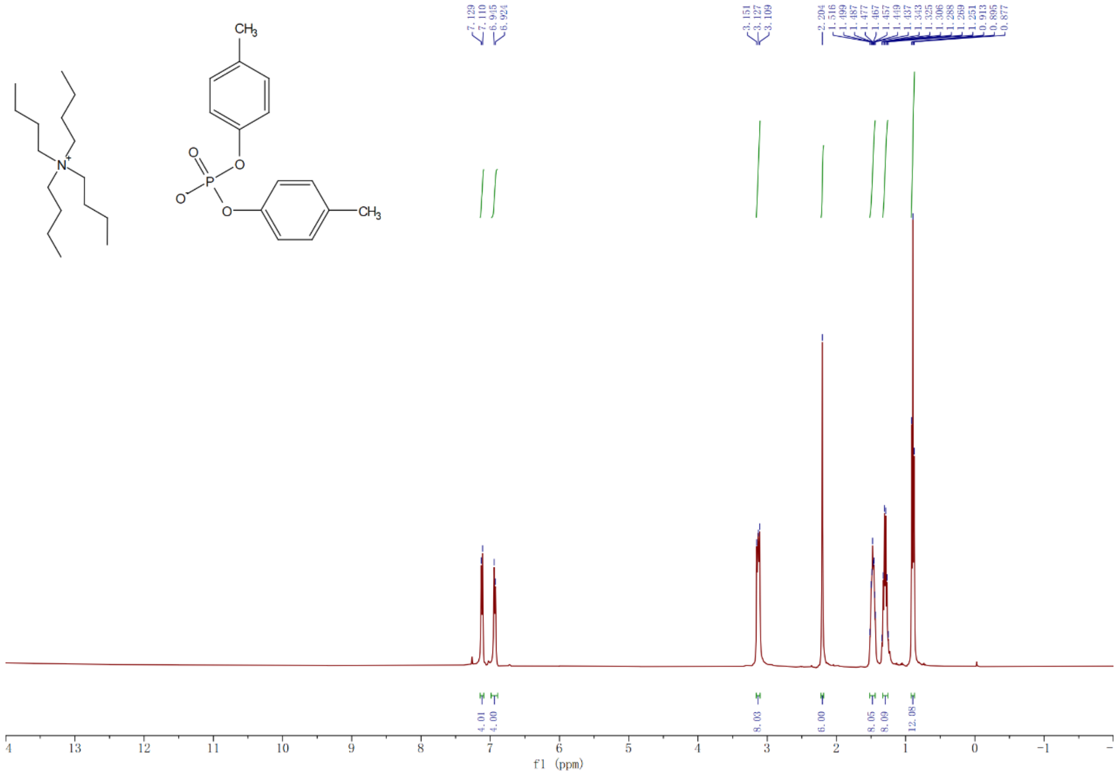

^{31}P NMR (400 MHz, $CDCl_3$)

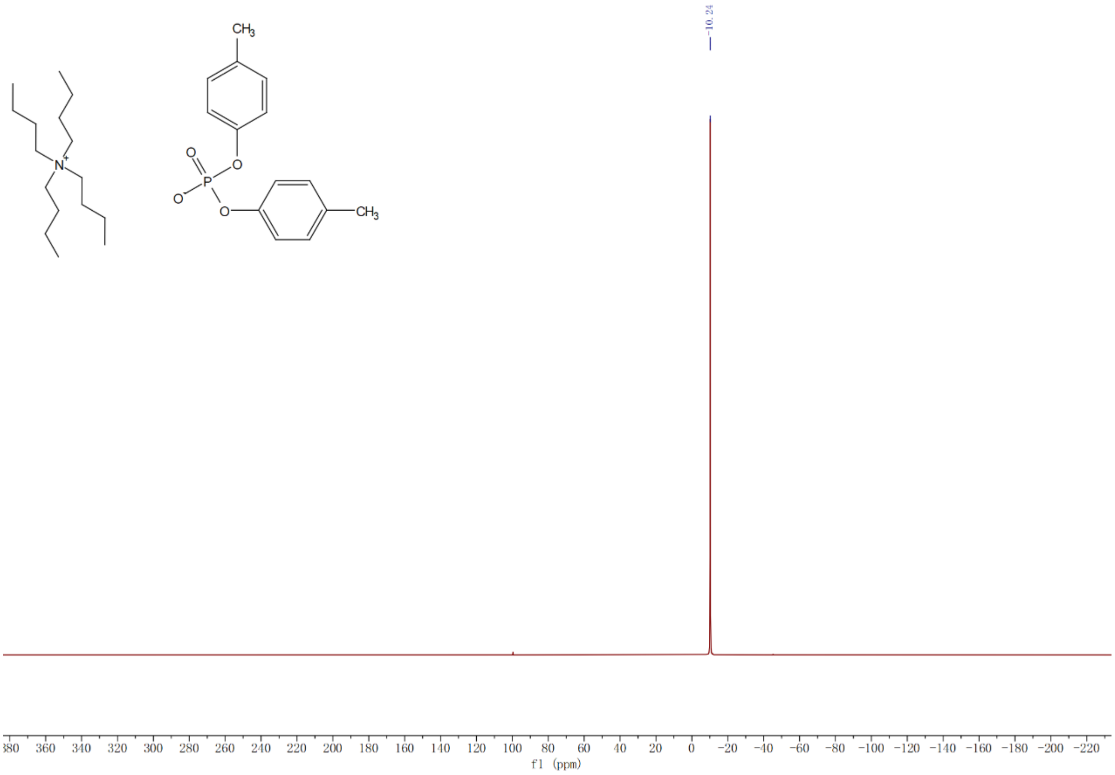

^{13}C NMR (600 MHz, $CDCl_3$)

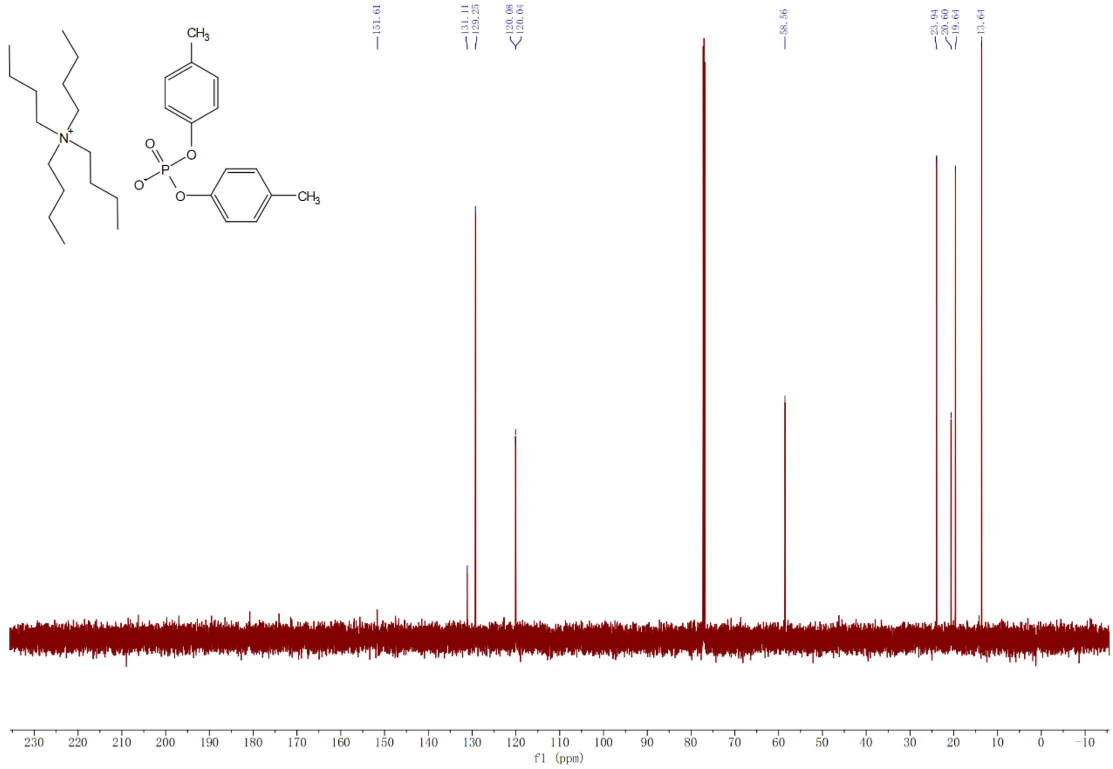

^1H NMR (400 MHz, $CDCl_3$)

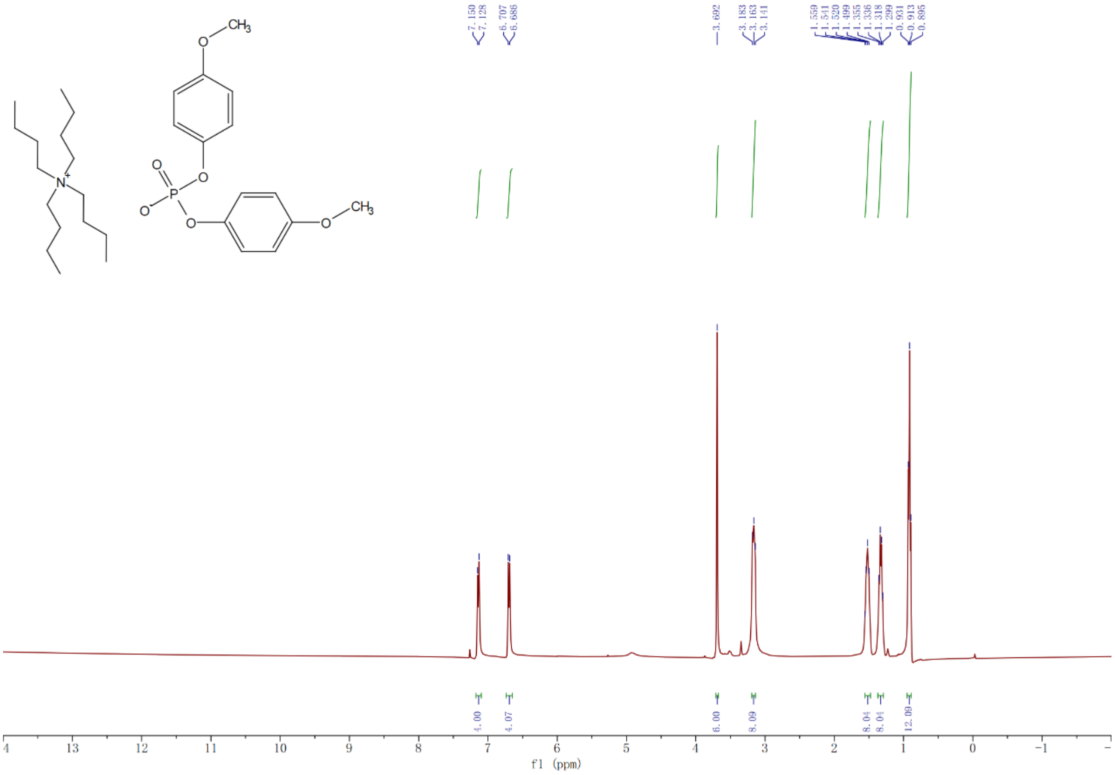

^{31}P NMR (400 MHz, $CDCl_3$)

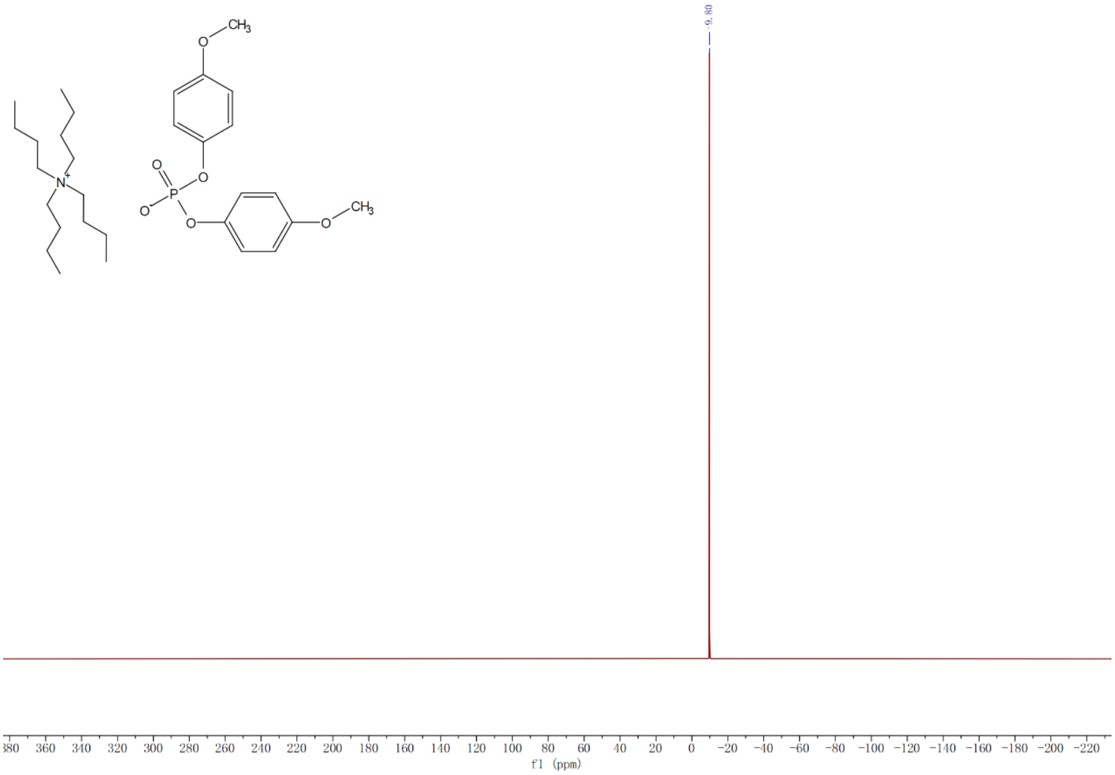

^{13}C NMR (600 MHz, $CDCl_3$)

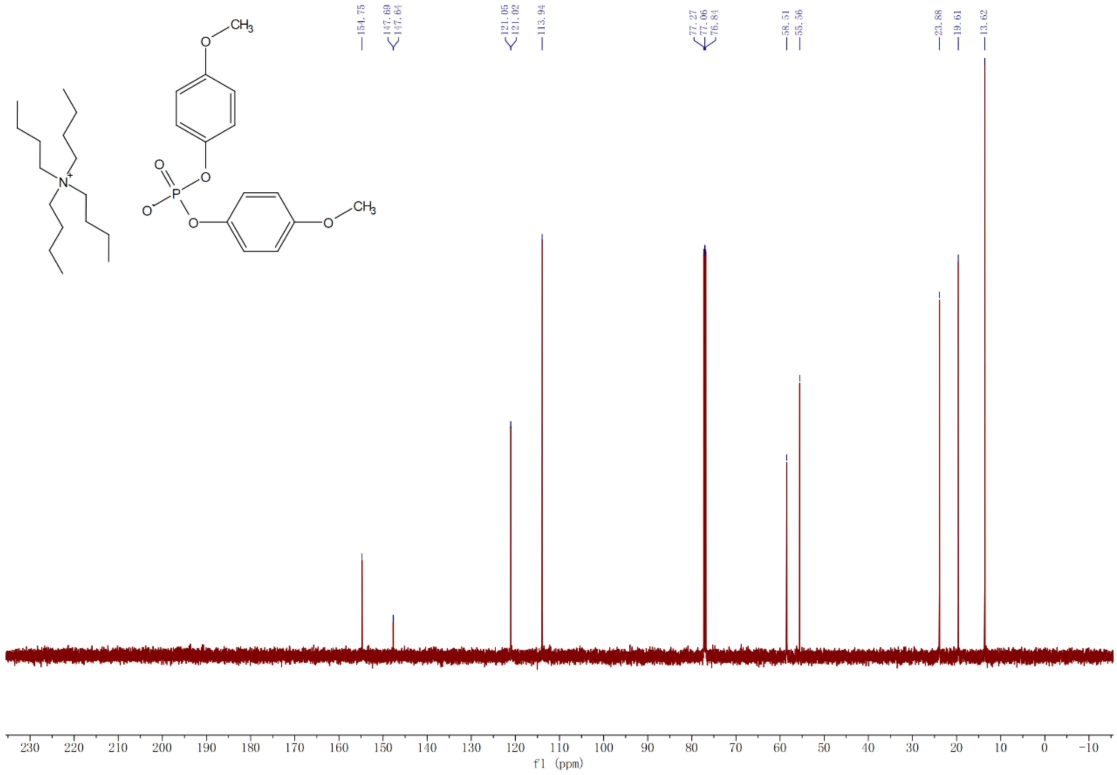

^{1}H NMR (400 MHz, DMSO-D_6)

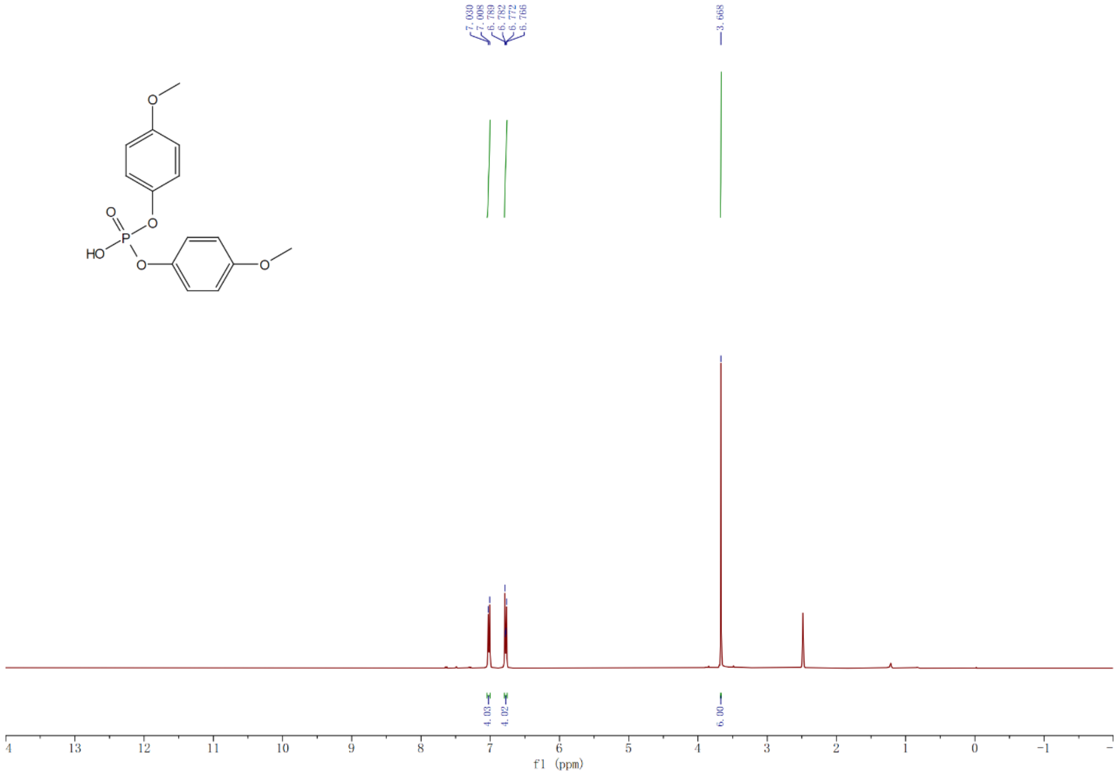

^{31}P NMR (400 MHz, DMSO-D$_6$))

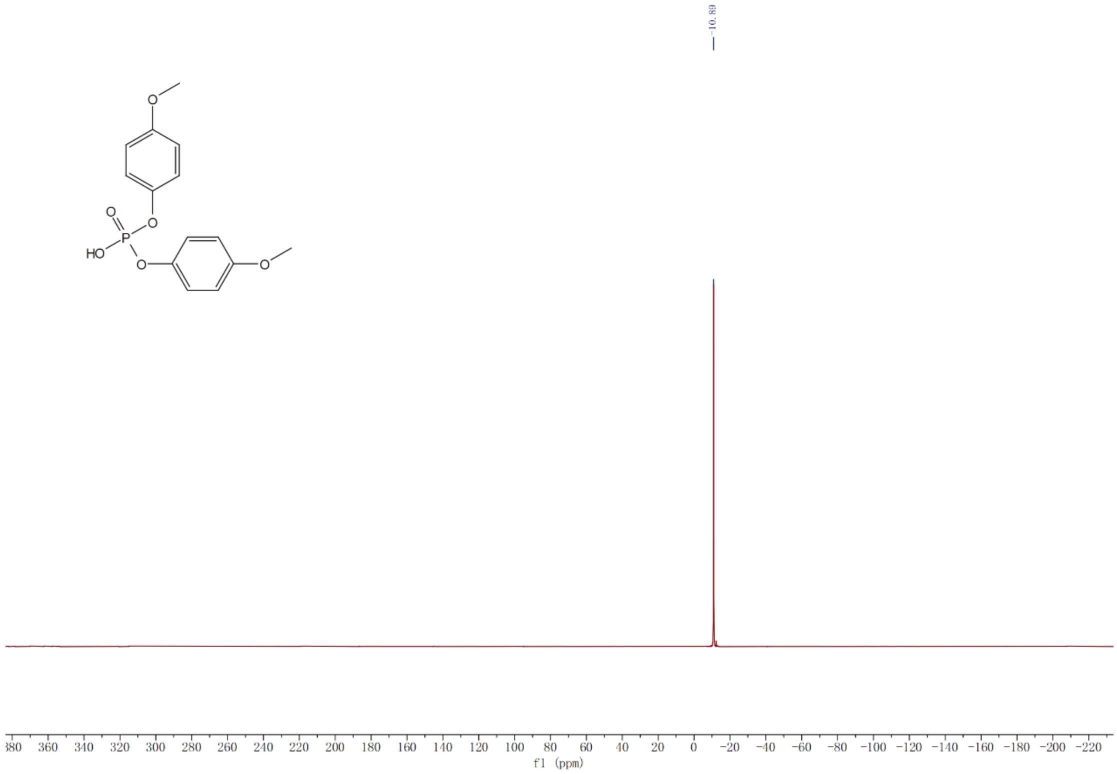

^{13}C NMR (600 MHz, DMSO-D$_6$)

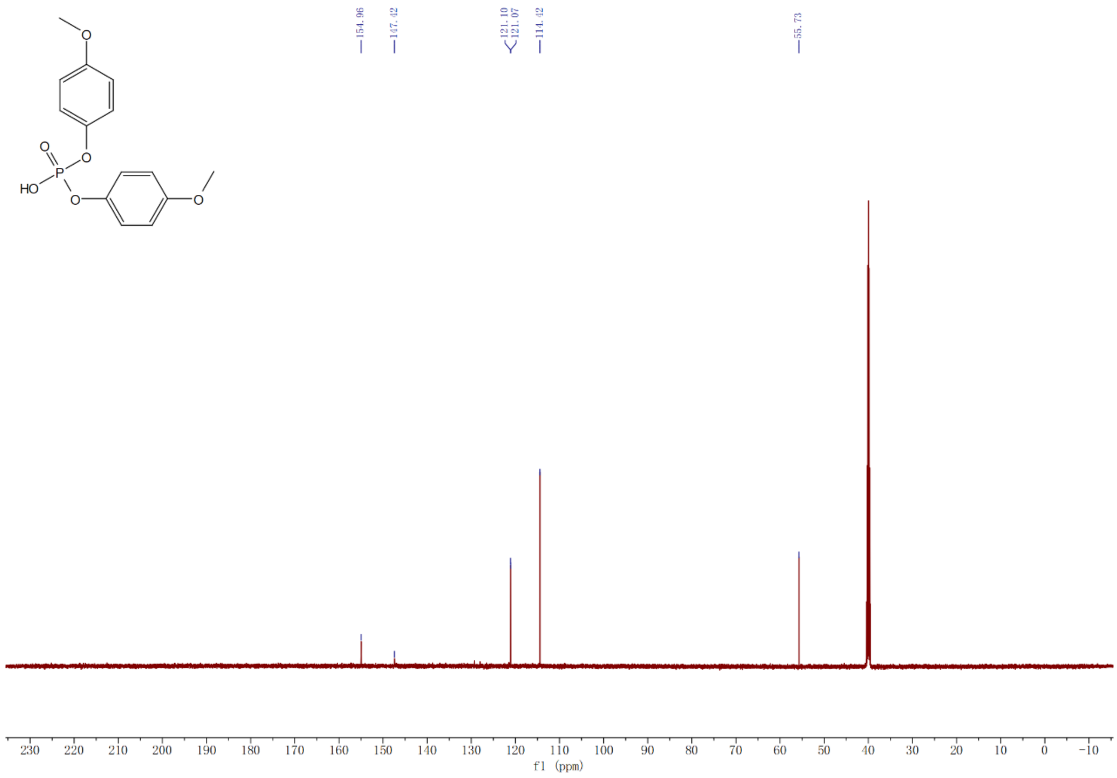

^{1}H NMR (400 MHz, $CDCl_3$)

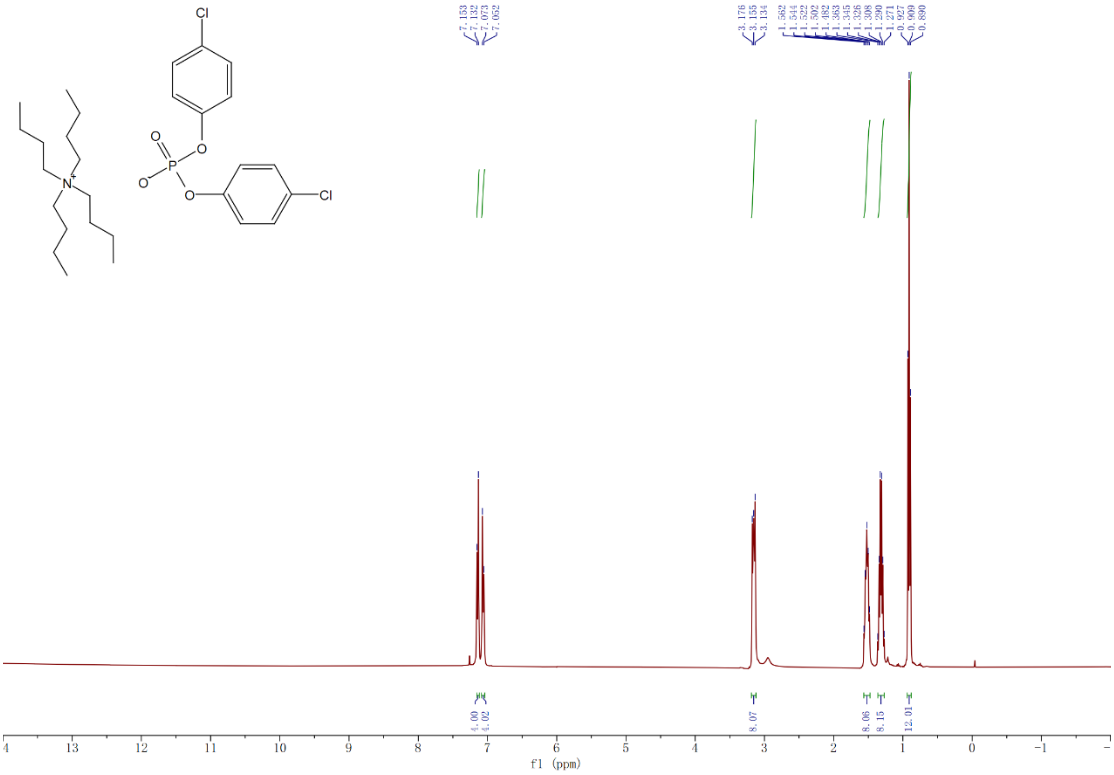

^{31}P NMR (400 MHz, $CDCl_3$)

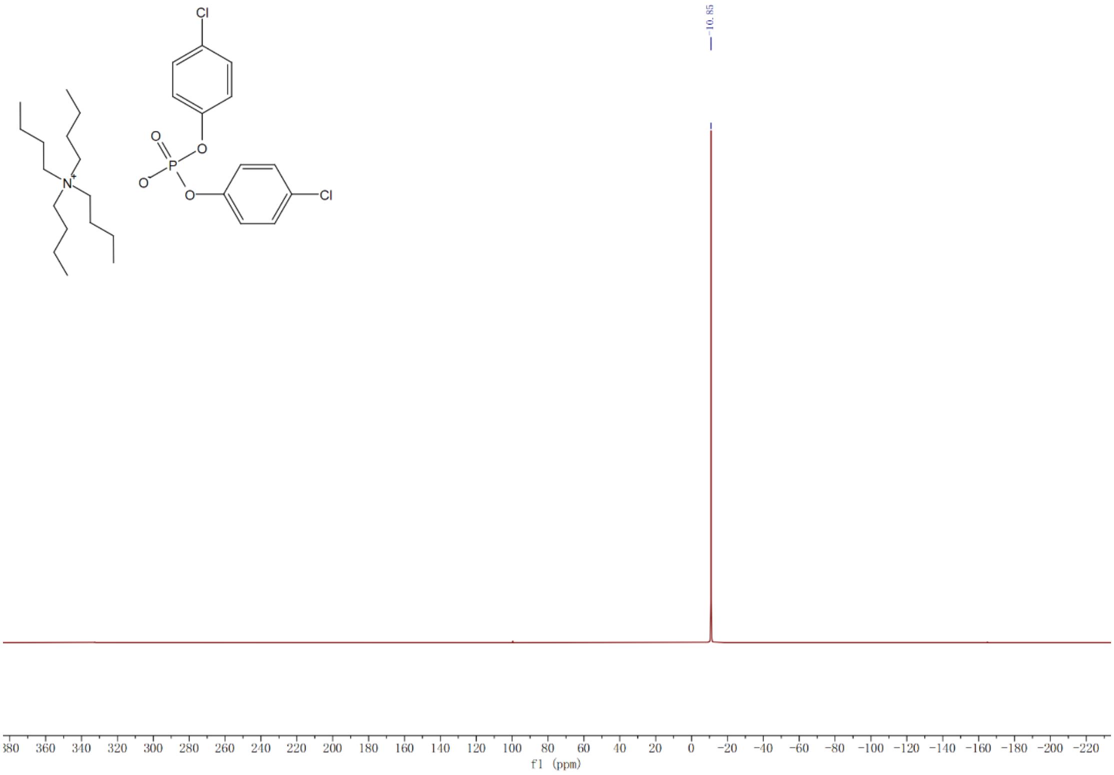

^{13}C NMR (600 MHz, $CDCl_3$)

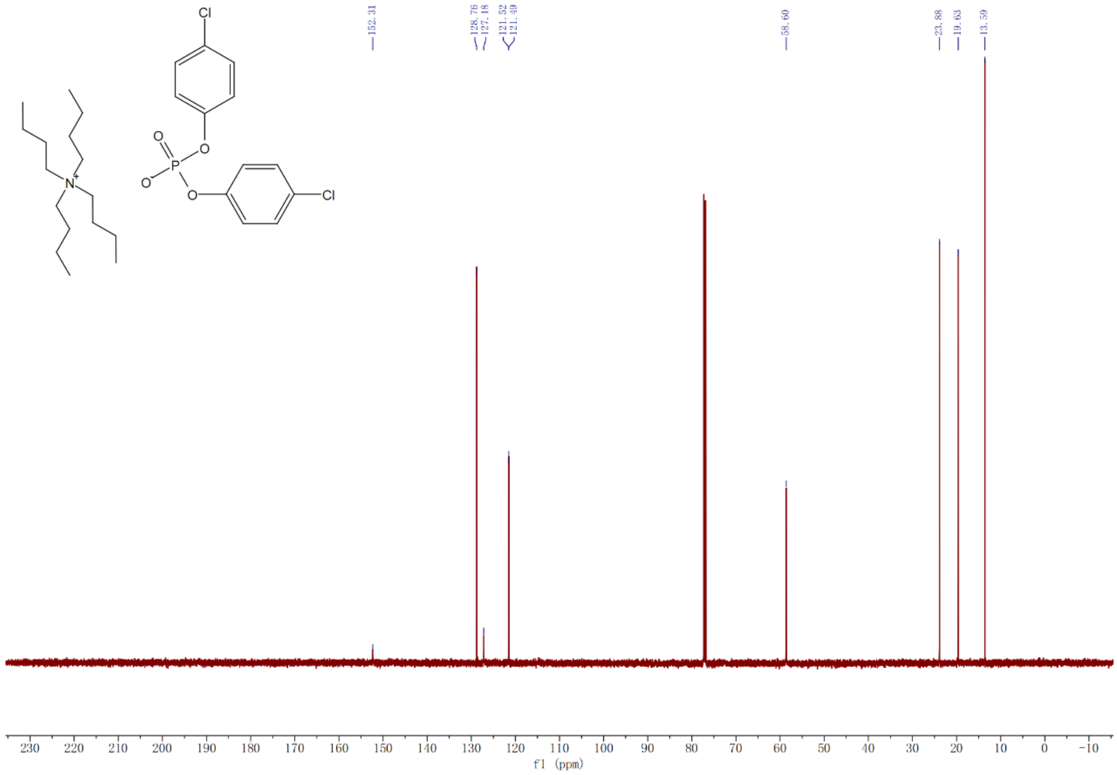

^{1}H NMR (400 MHz, DMSO-D_6)

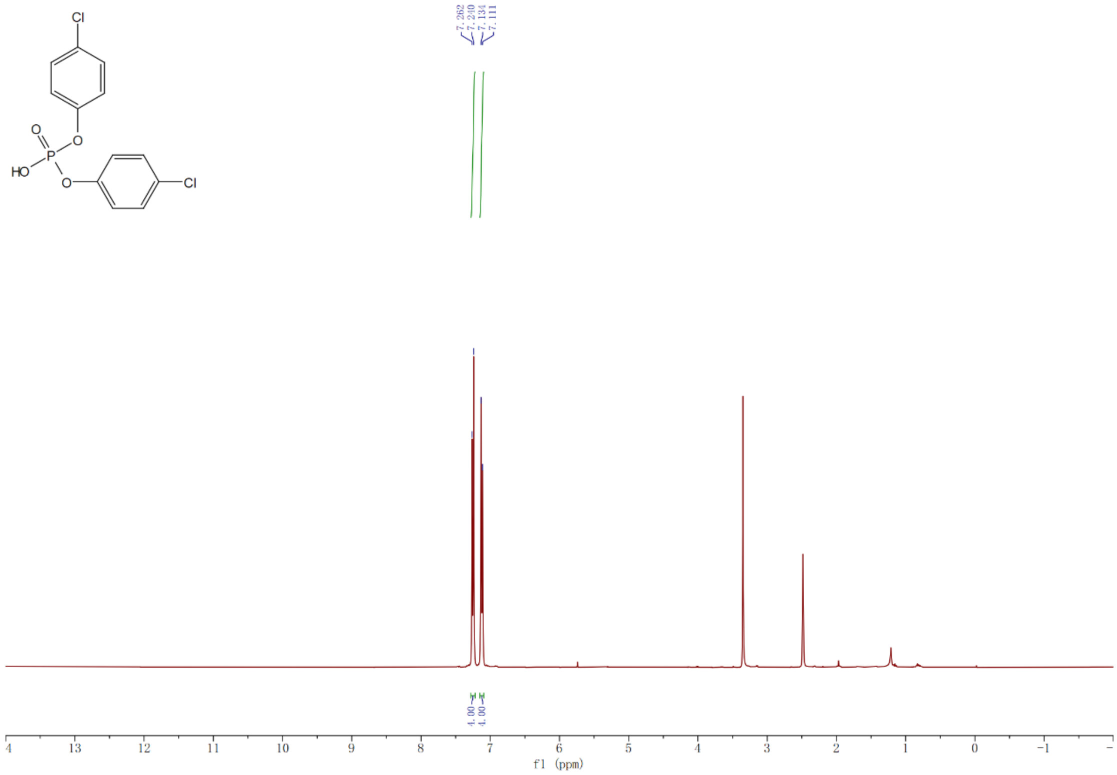

^{31}P NMR (400 MHz, DMSO-D$_6$)

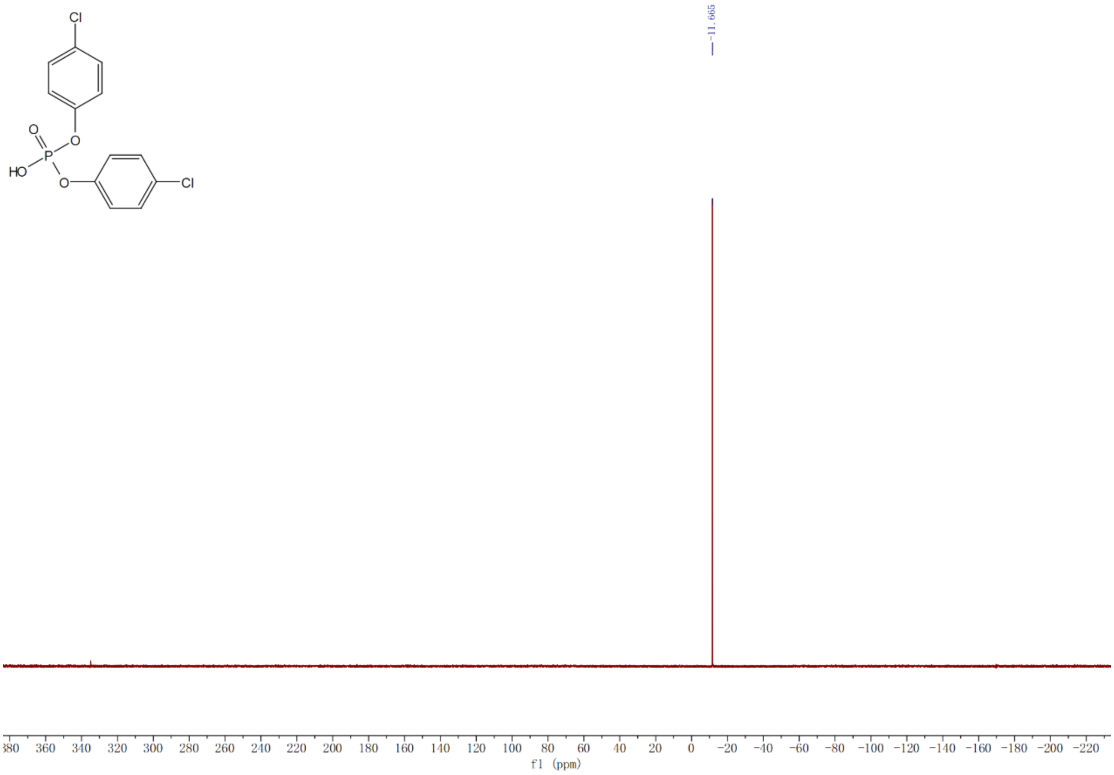

^{13}C NMR (600 MHz, DMSO-D$_6$)

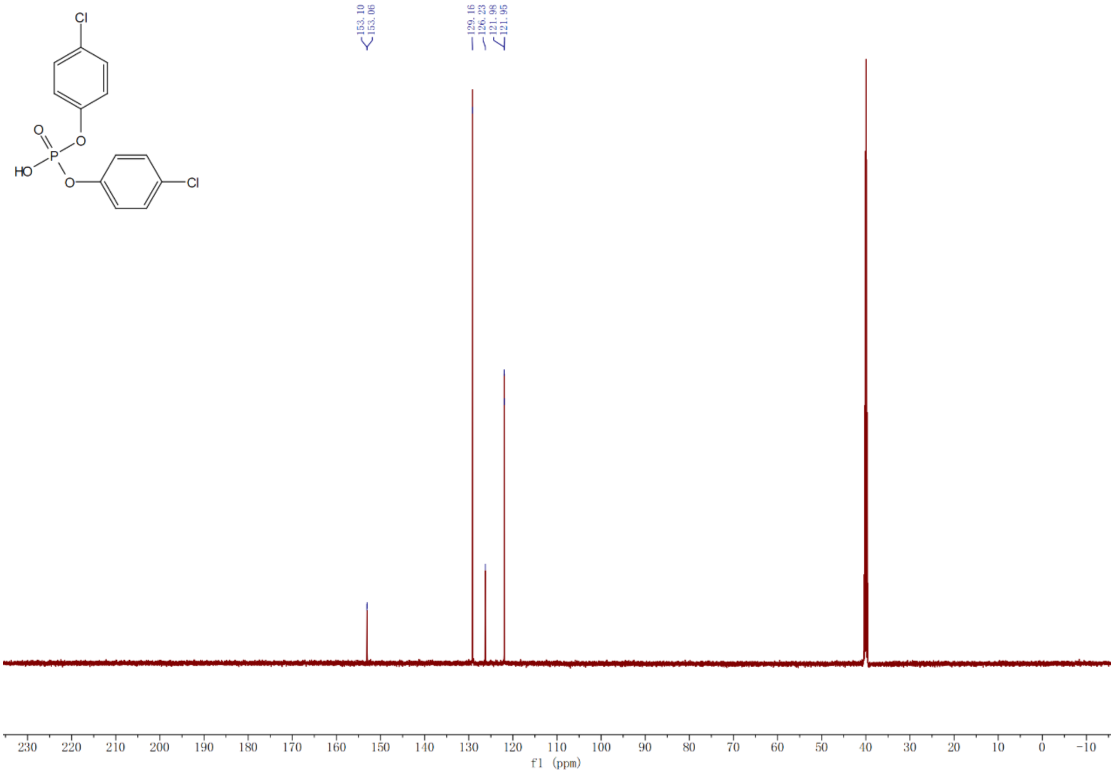

^{1}H NMR (400 MHz, $CDCl_3$)

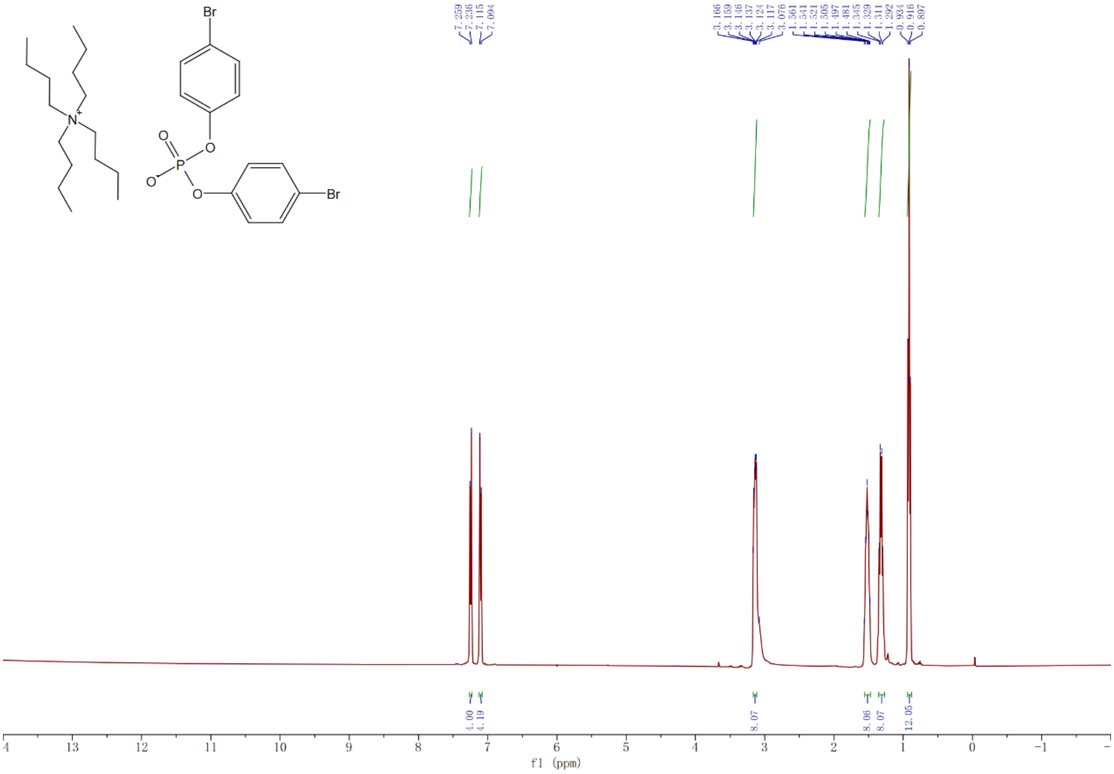

^{31}P NMR (400 MHz, $CDCl_3$)

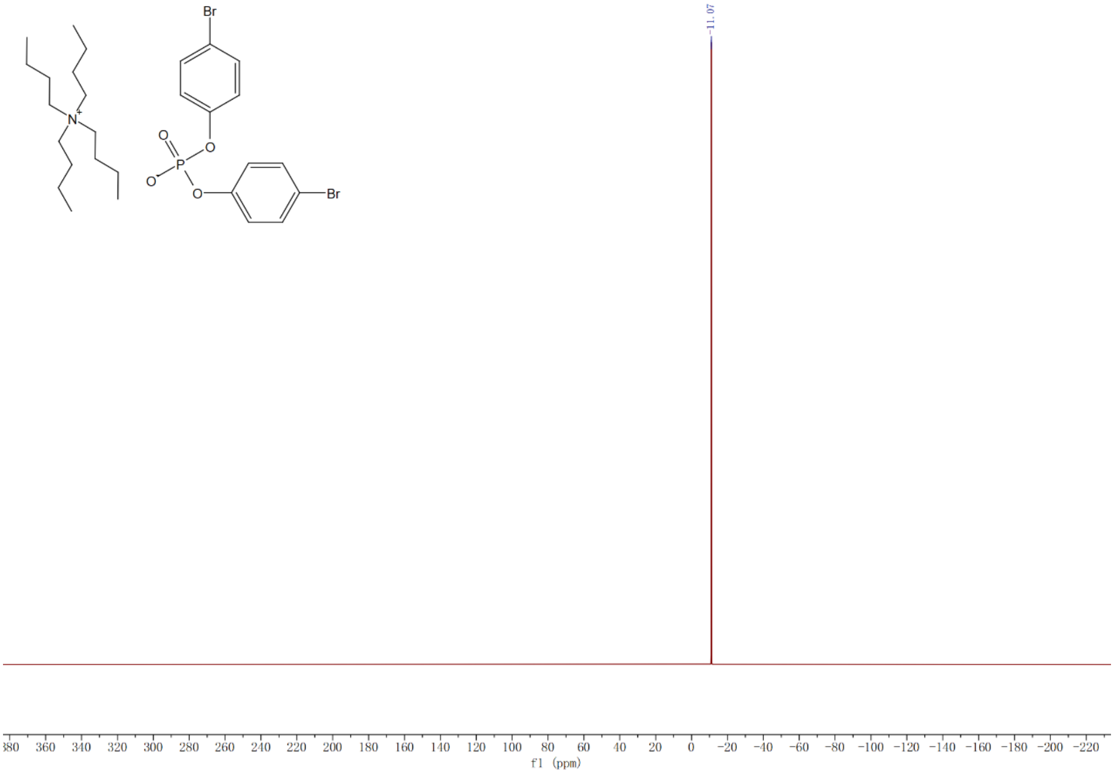

^{13}C NMR (600 MHz, CDCl$_3$)

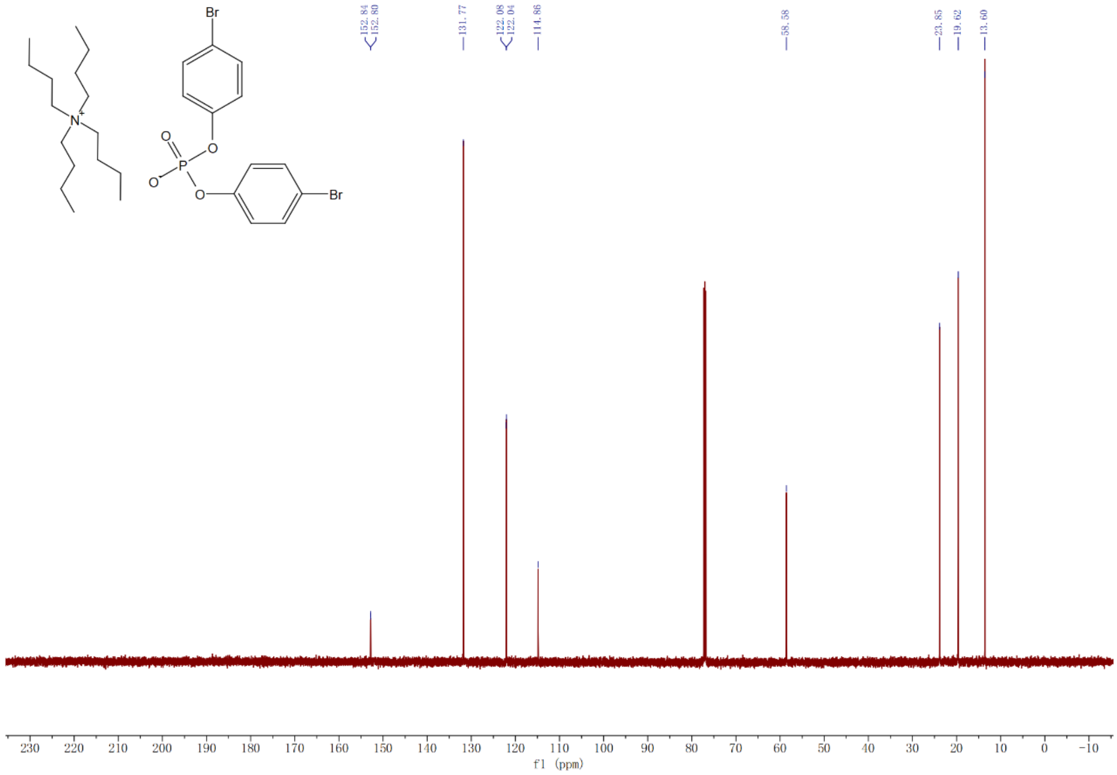

^{1}H NMR (400 MHz, CDCl$_3$)

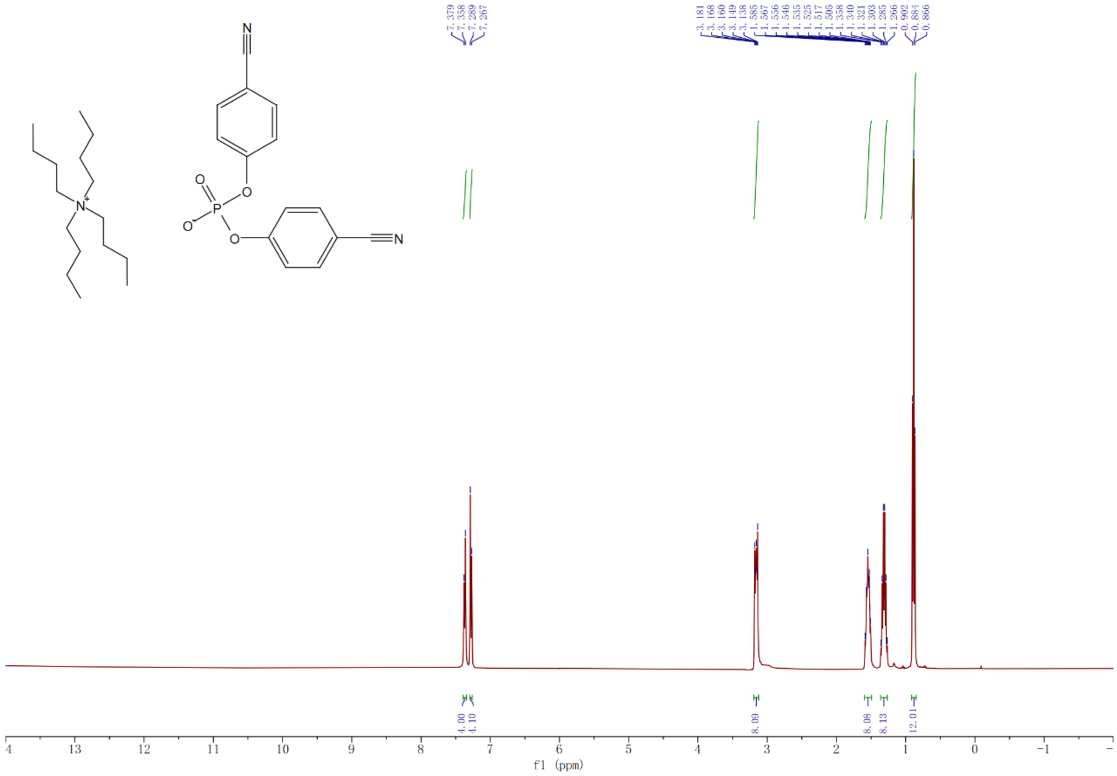

^{31}P NMR (400 MHz, $CDCl_3$)

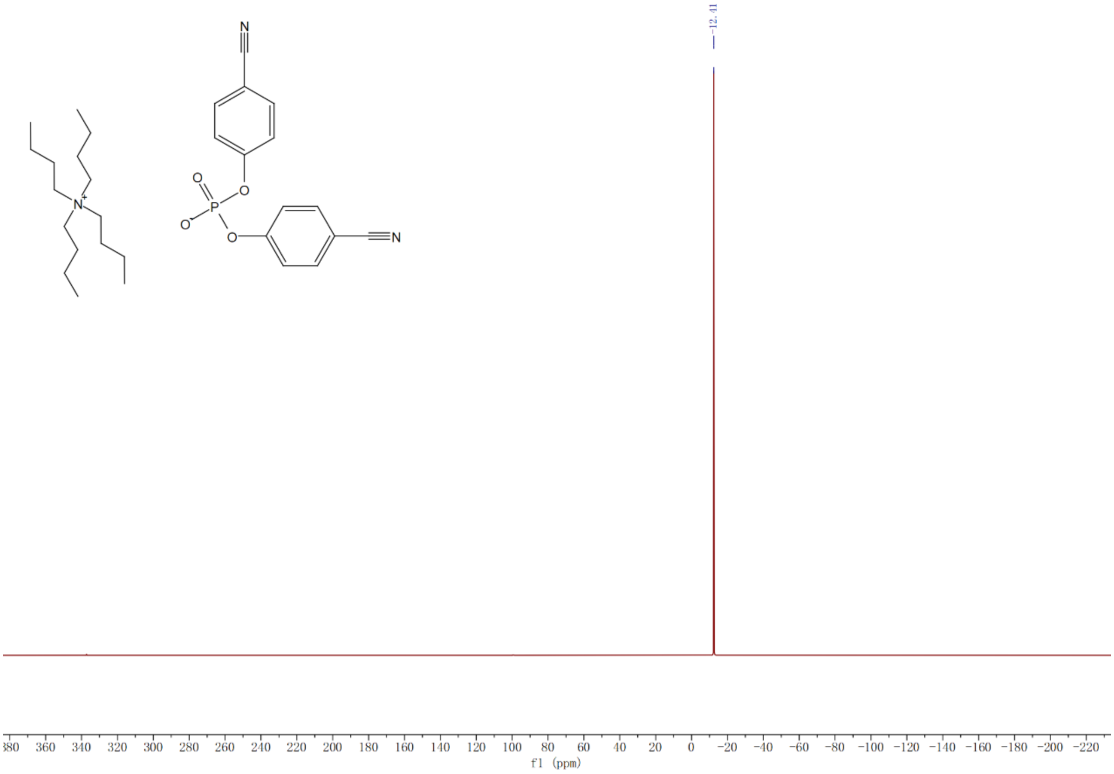

^{13}C NMR (600 MHz, $CDCl_3$)

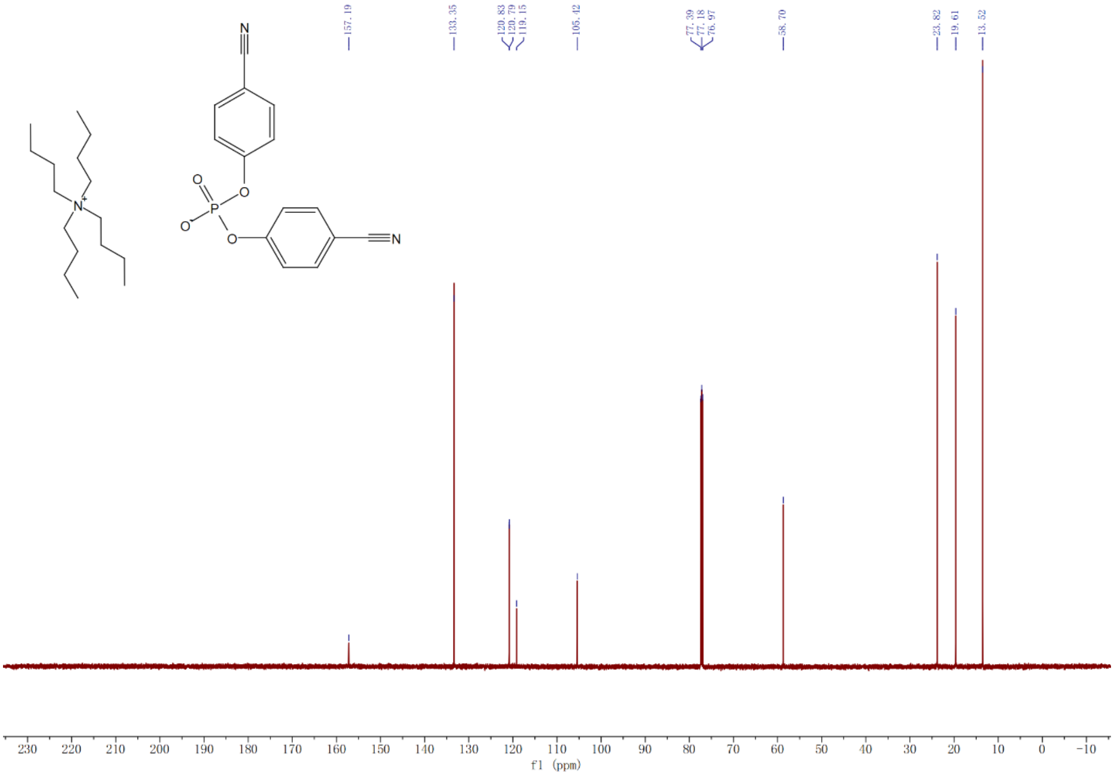

^{1}H NMR (400 MHz, $CDCl_3$)

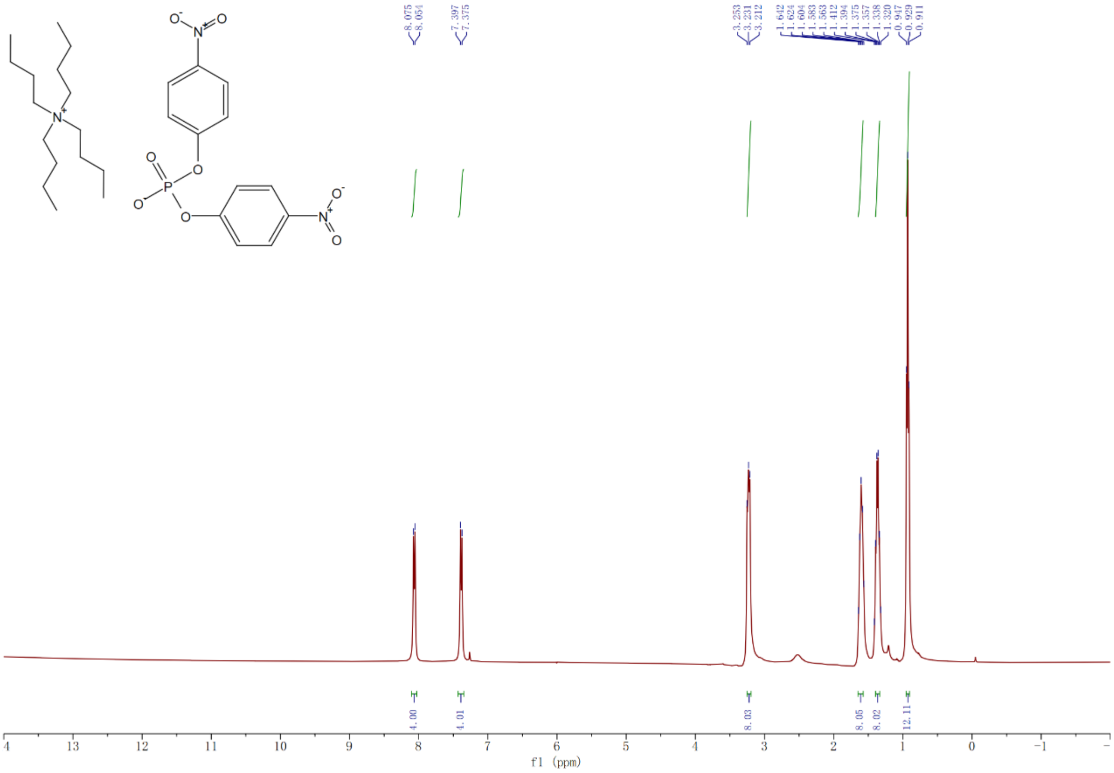

^{31}P NMR (400 MHz, $CDCl_3$)

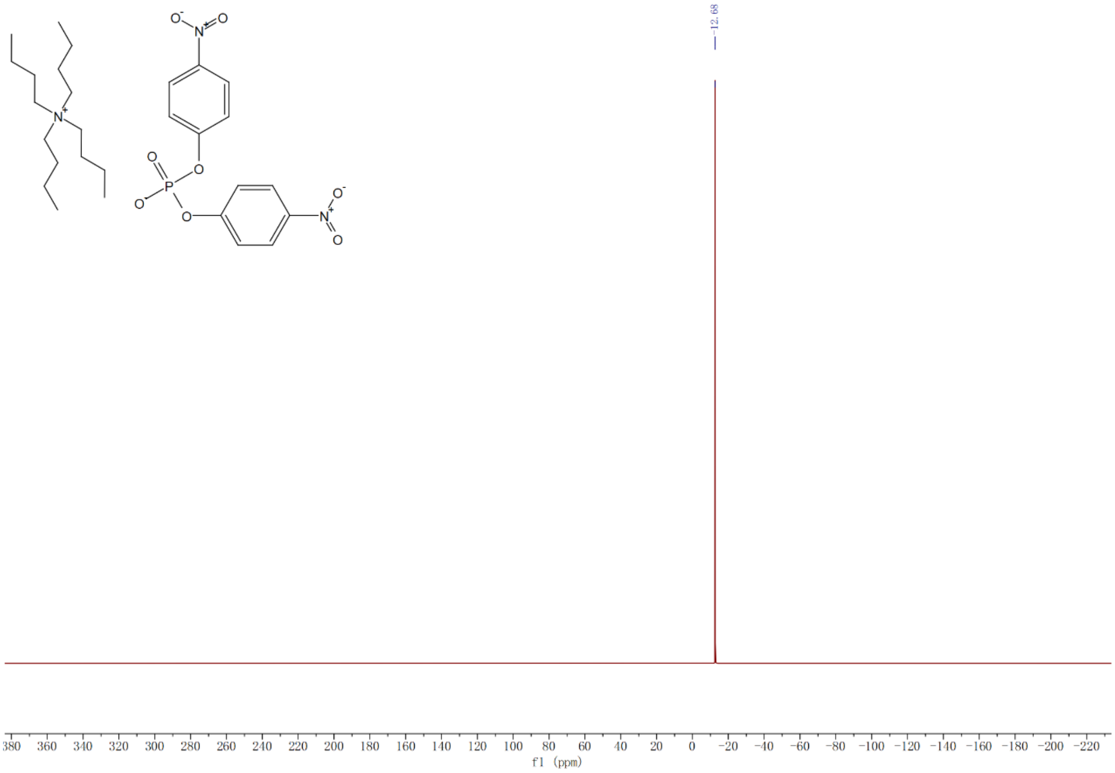

^{13}C NMR (600 MHz, $CDCl_3$)

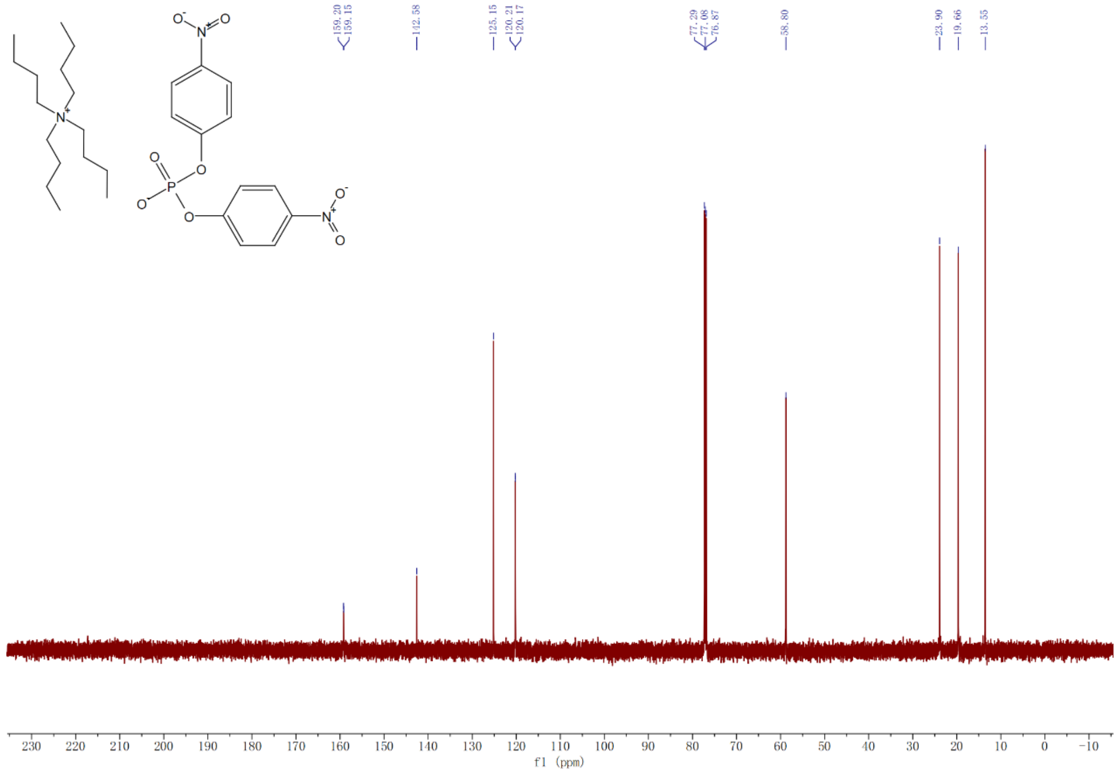

^{1}H NMR (400 MHz, DMSO-D_6)

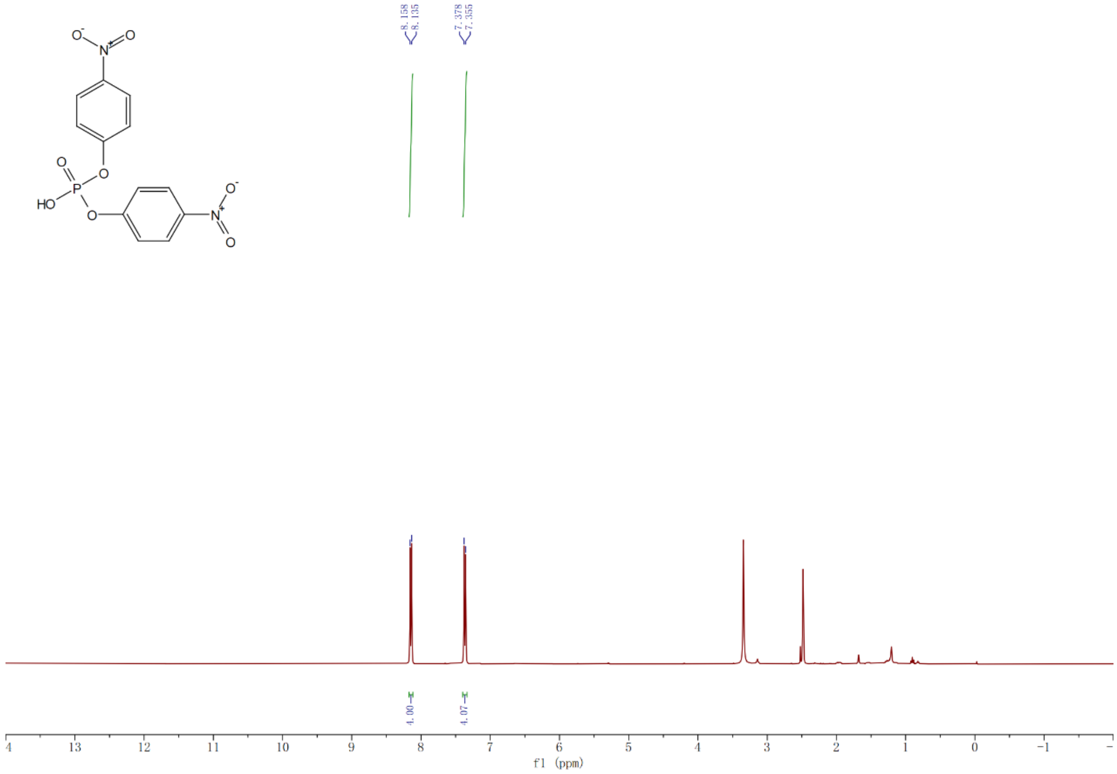

^{31}P NMR (400 MHz, DMSO-D_6))

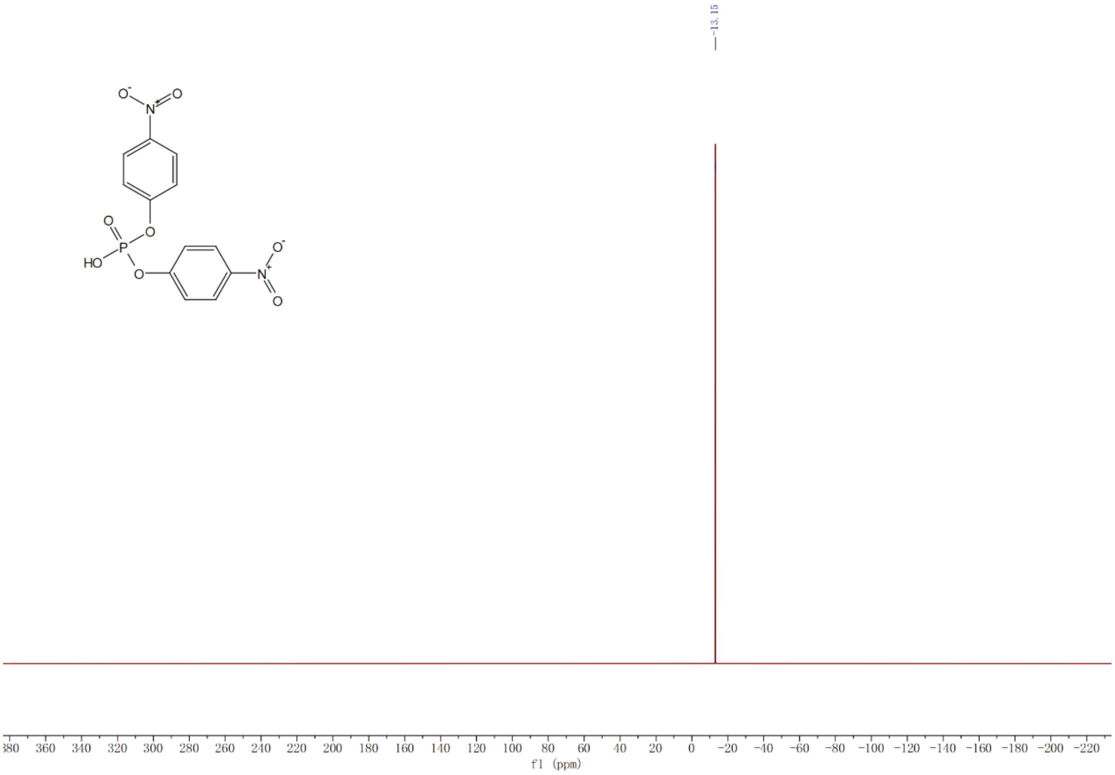

^{13}C NMR (600 MHz, DMSO-D_6) [1]

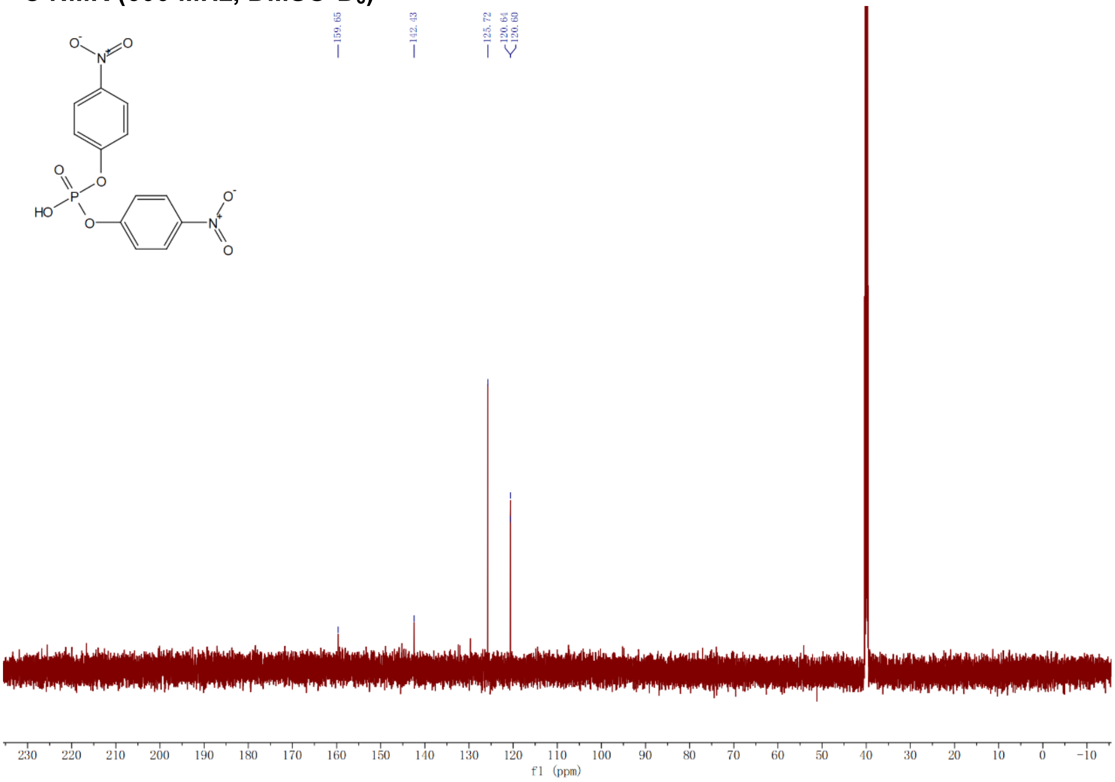

^{1}H NMR (400 MHz, $CDCl_3$)

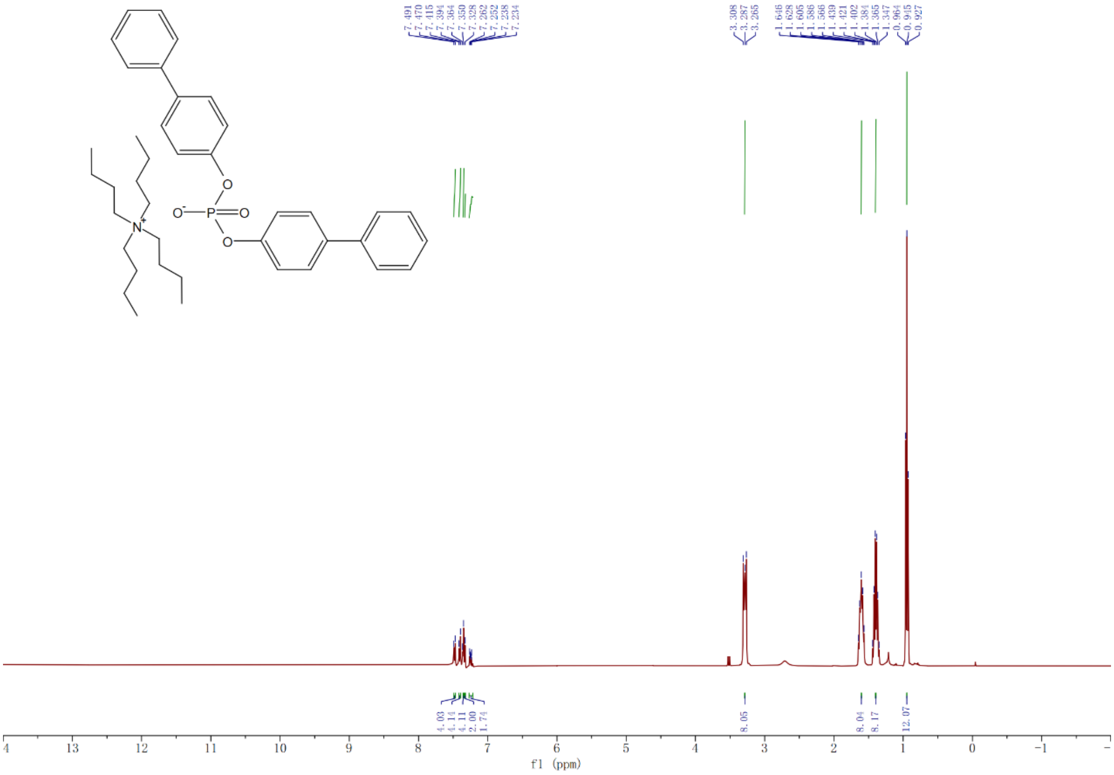

^{31}P NMR (400 MHz, $CDCl_3$)

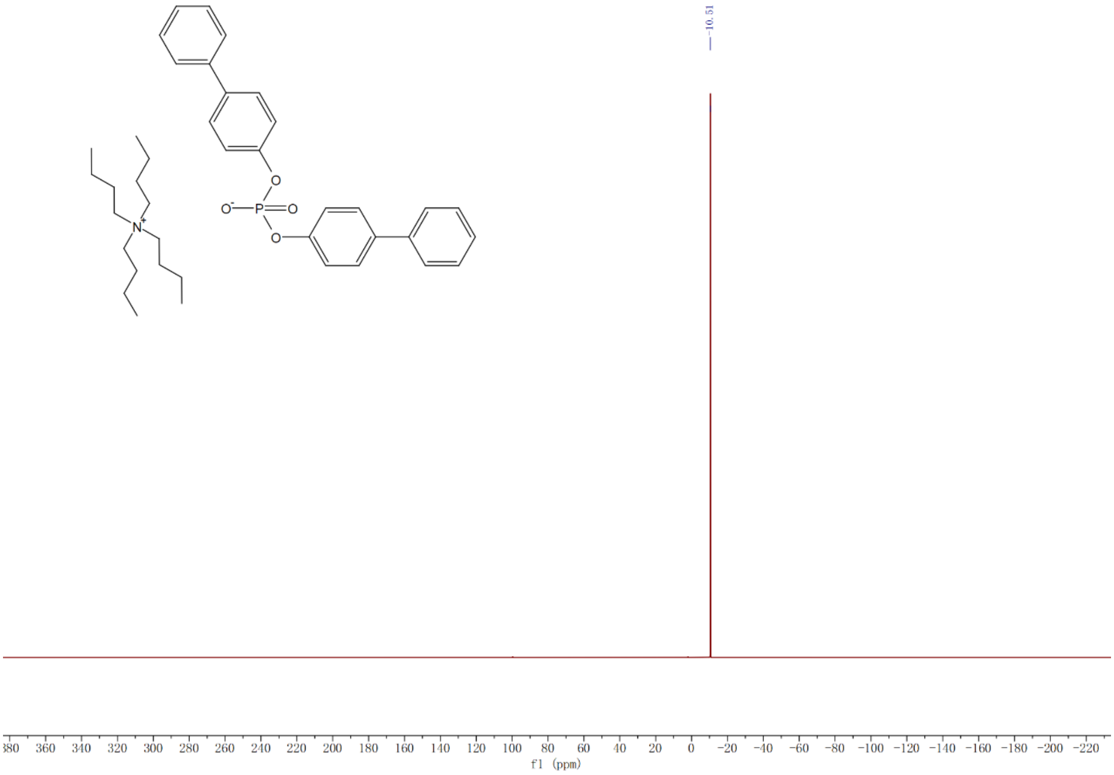

^{13}C NMR (600 MHz, $CDCl_3$)

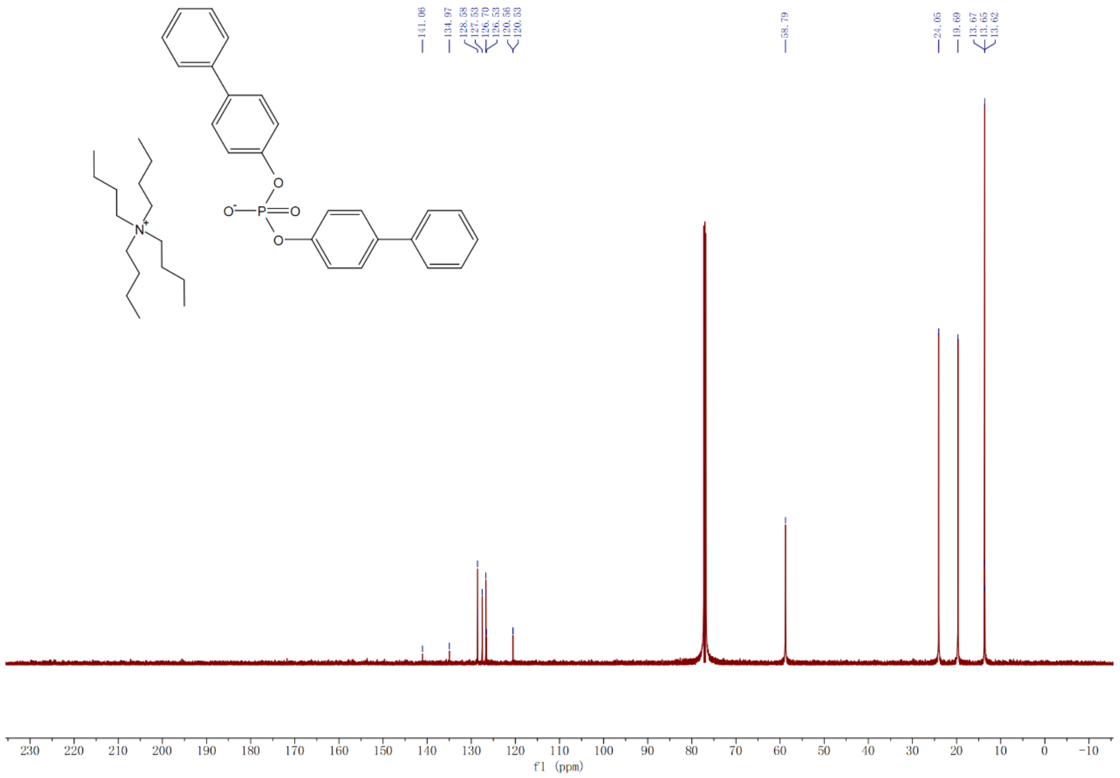

^{1}H NMR (400 MHz, $CDCl_3$)

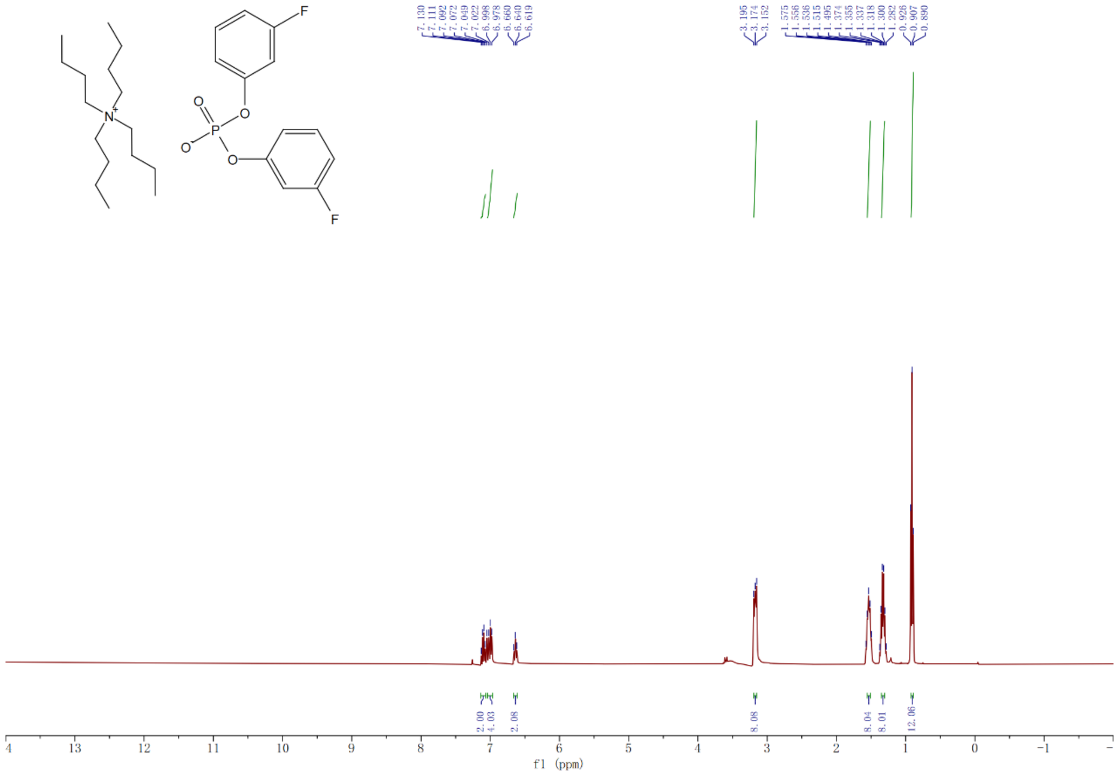

^{31}P NMR (400 MHz, $CDCl_3$)

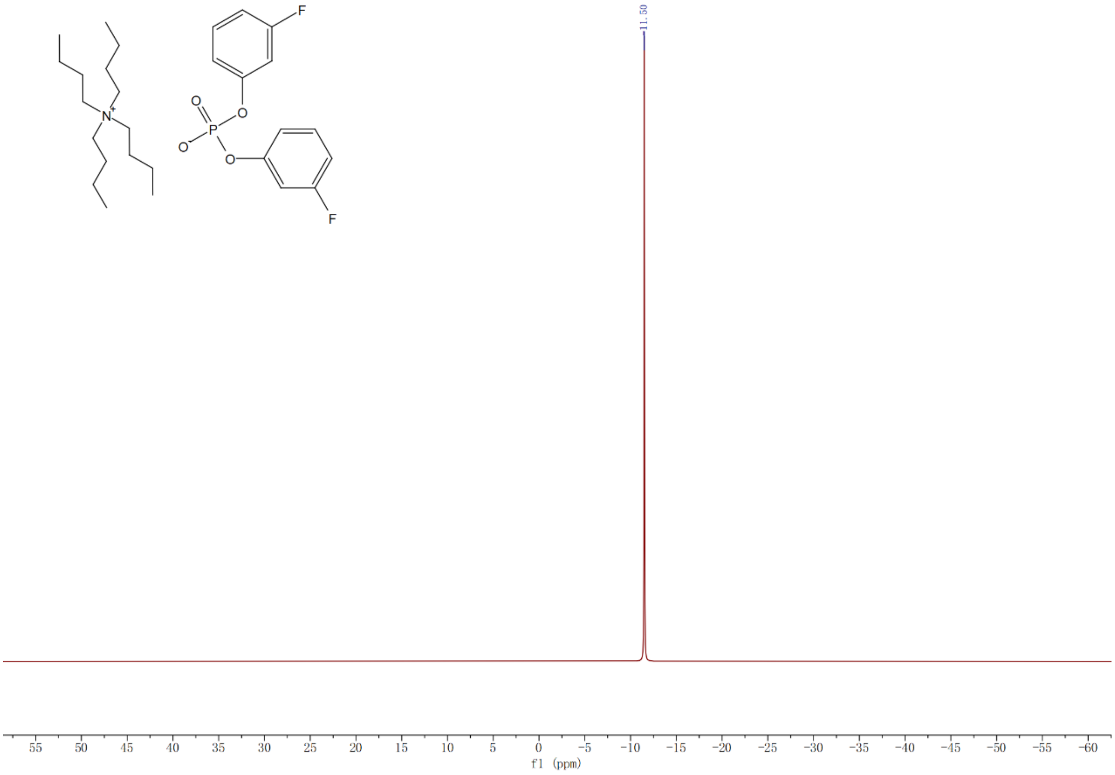

^{19}F NMR (400 MHz, $CDCl_3$)

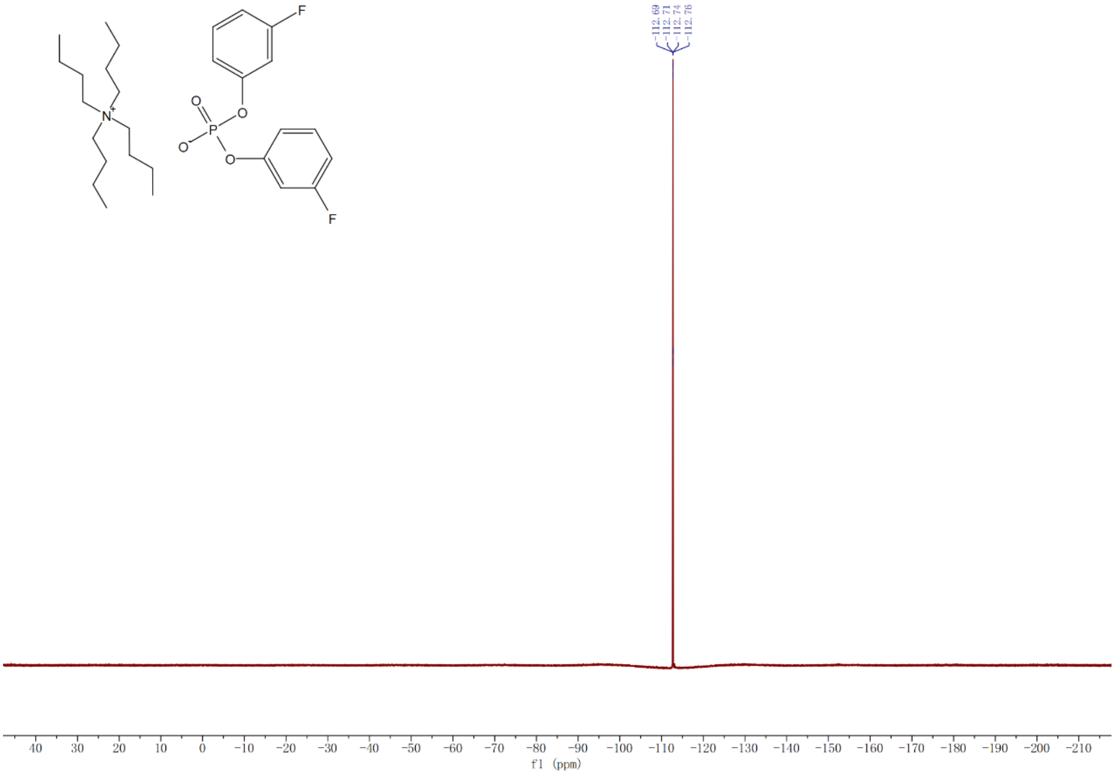

^{13}C NMR (600 MHz, CDCl$_3$)

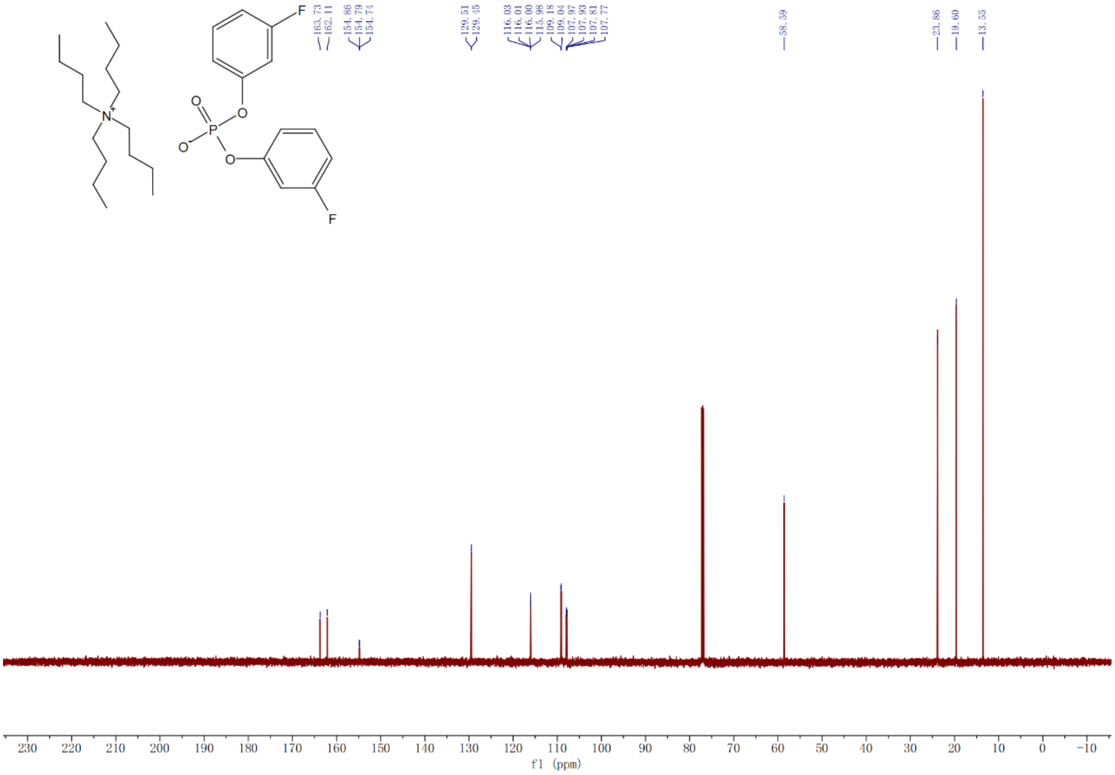

^{1}H NMR (400 MHz, CDCl$_3$)

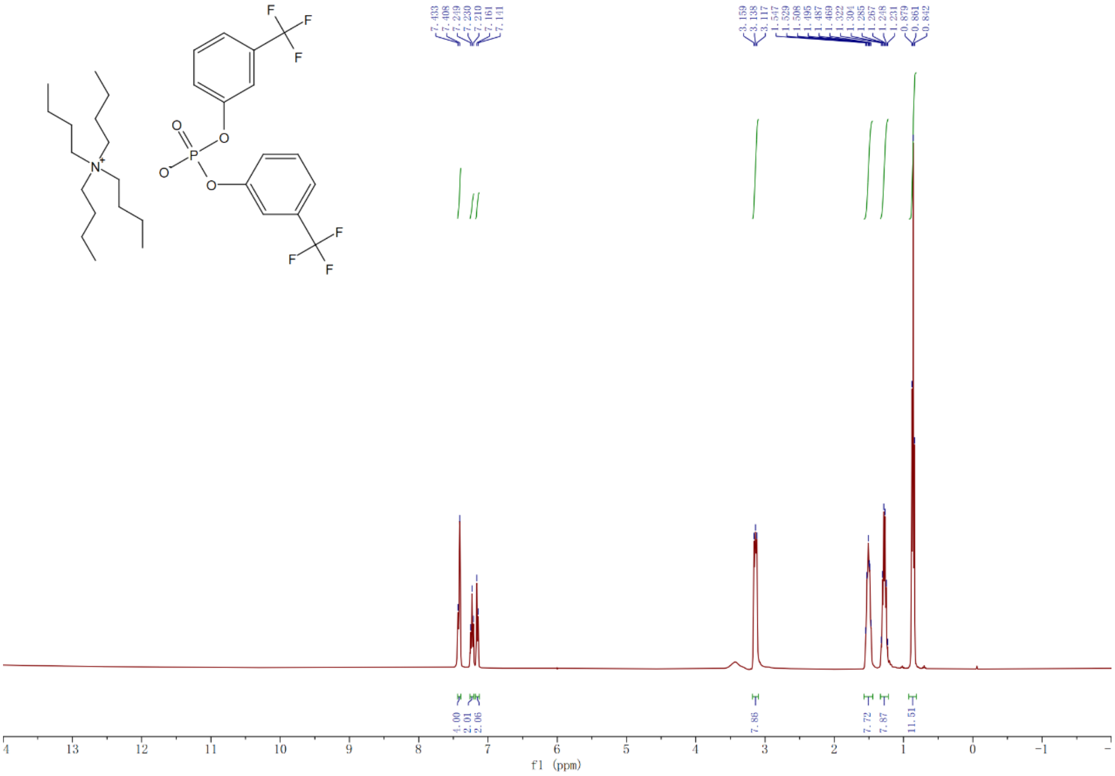

^{31}P NMR (400 MHz, $CDCl_3$)

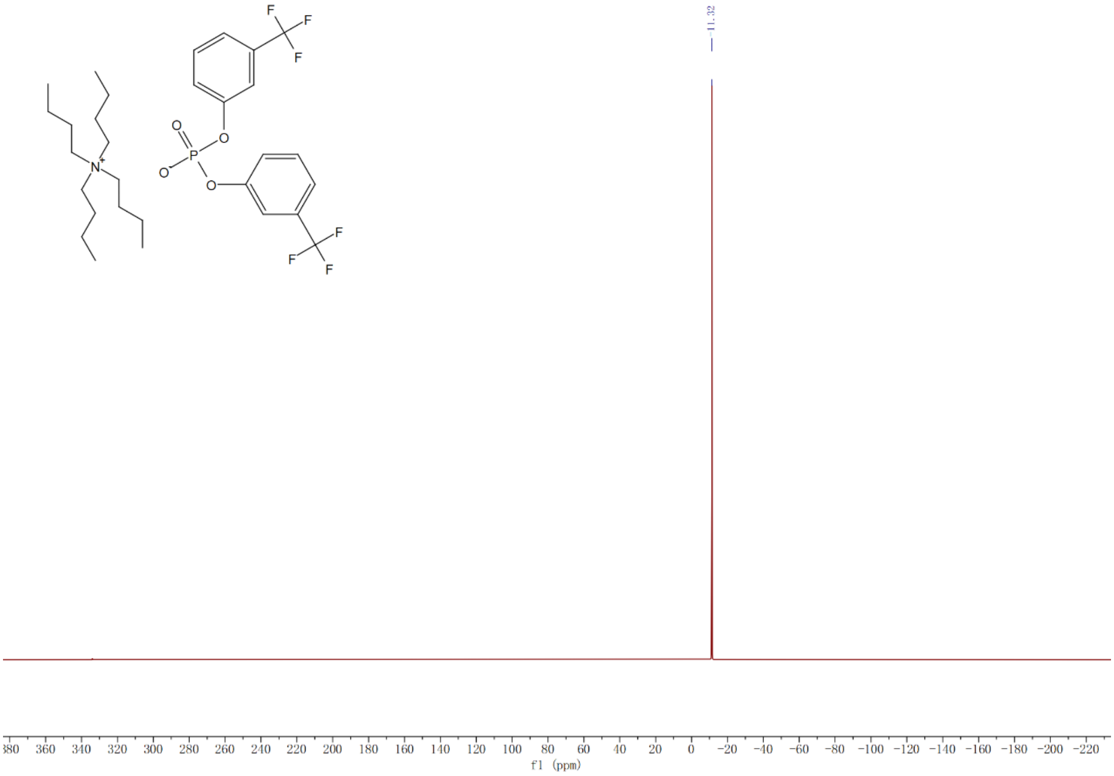

^{19}F NMR (400 MHz, $CDCl_3$)

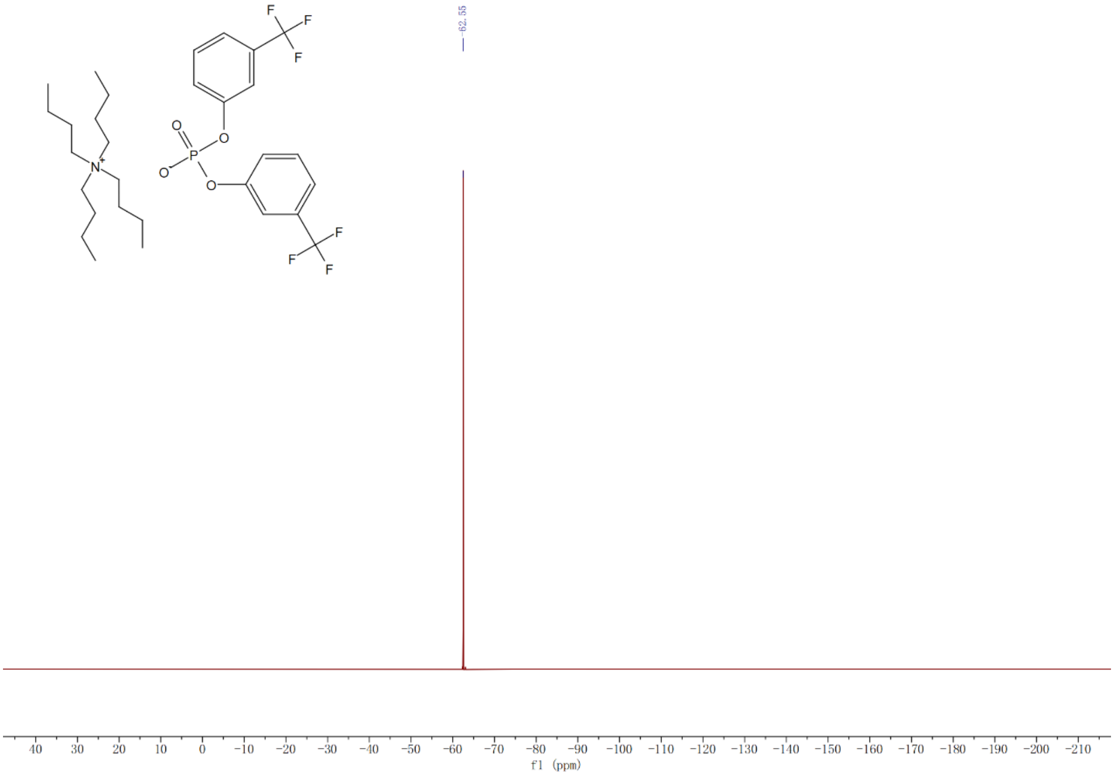

^{13}C NMR (600 MHz, $CDCl_3$)

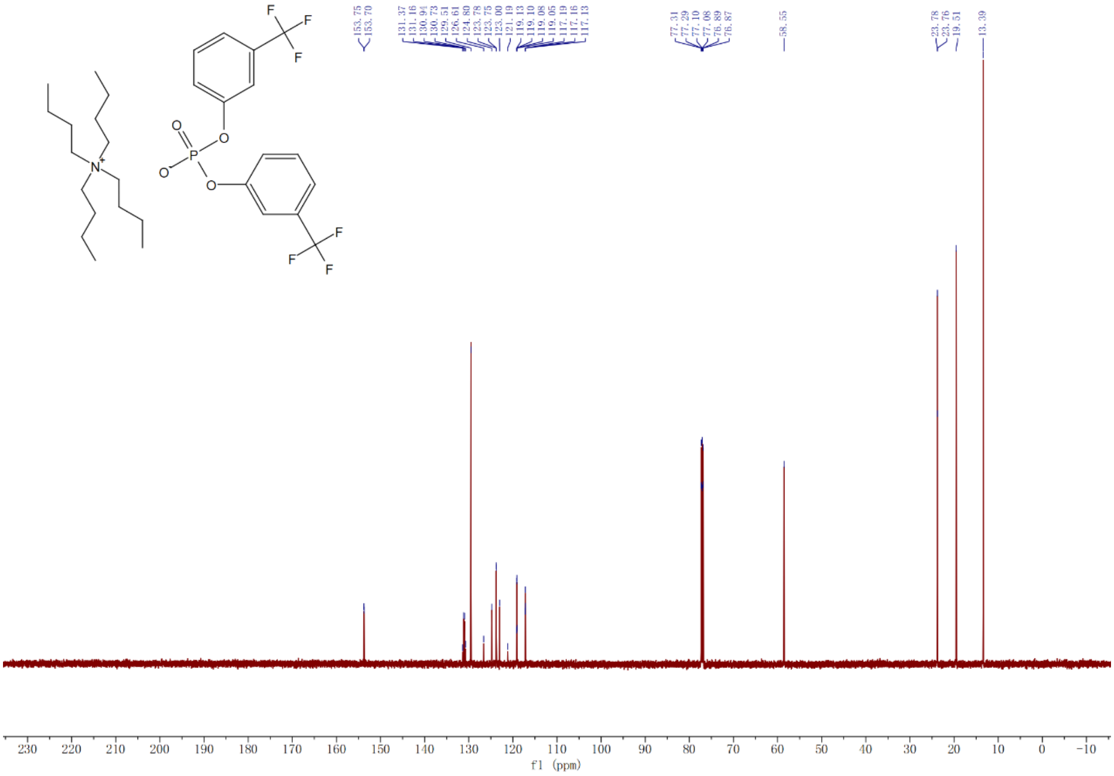

^{1}H NMR (400 MHz, DMSO-D_6)

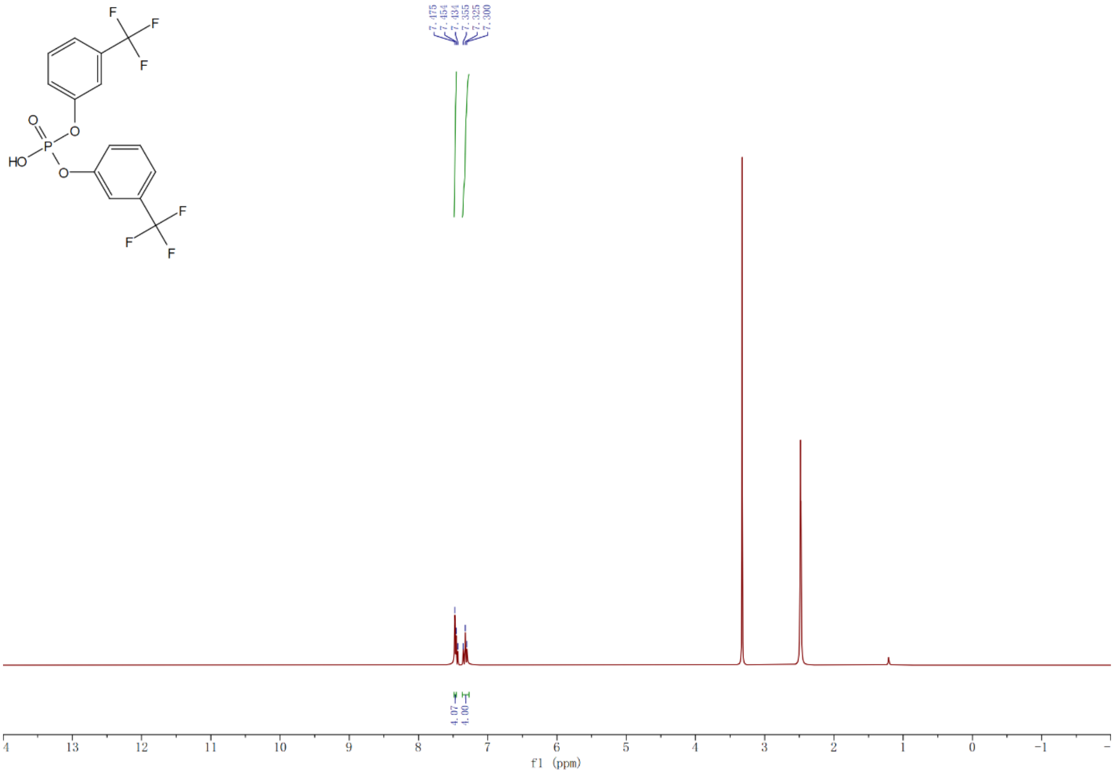

^{31}P NMR (400 MHz, DMSO-D$_6$))

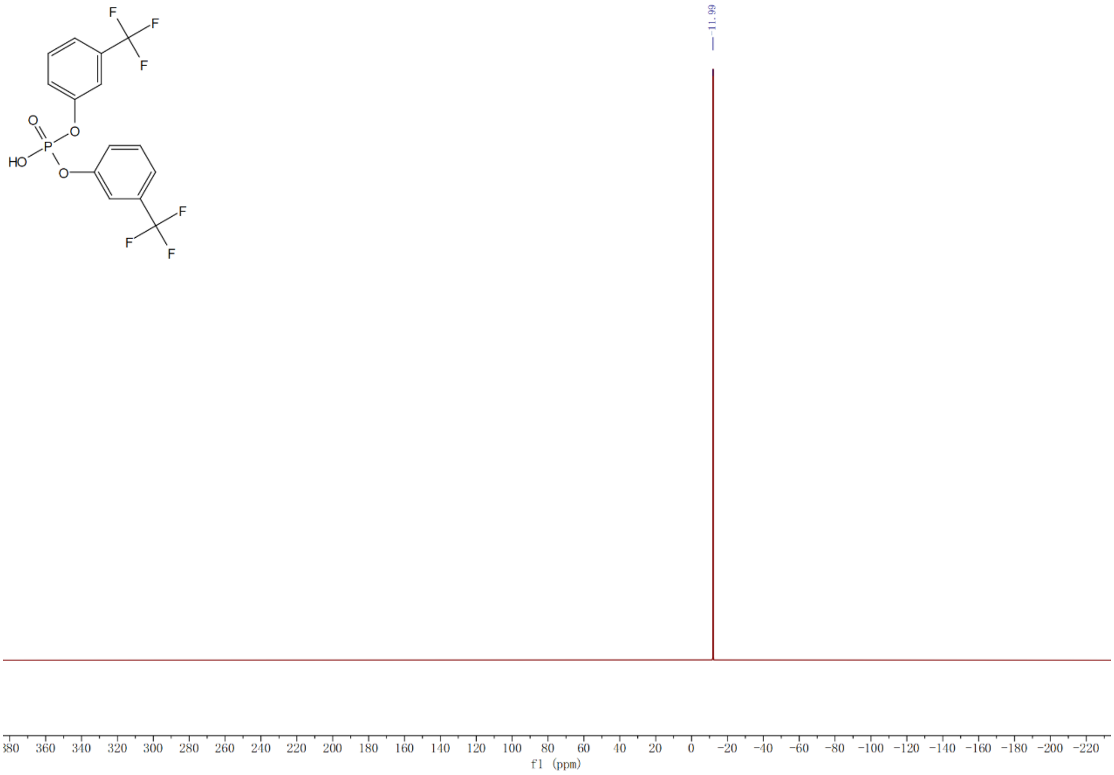

^{19}F NMR (400 MHz, DMSO-D$_6$)

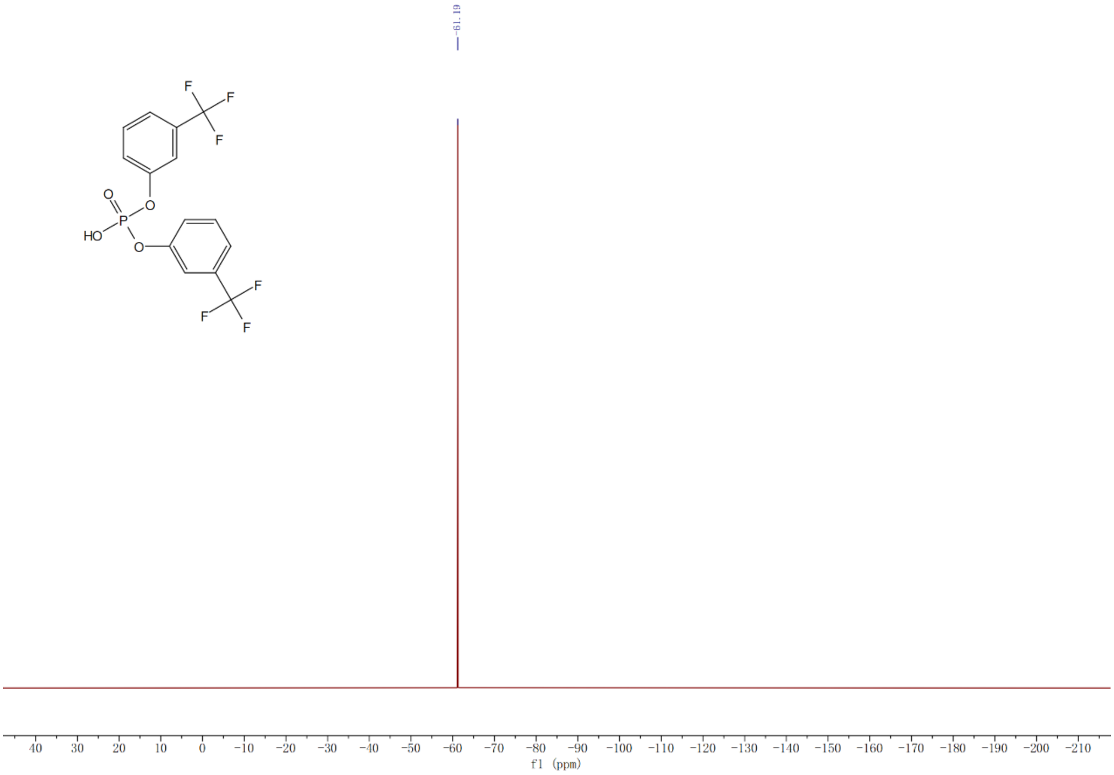

^{13}C NMR (600 MHz, DMSO-D$_6$)

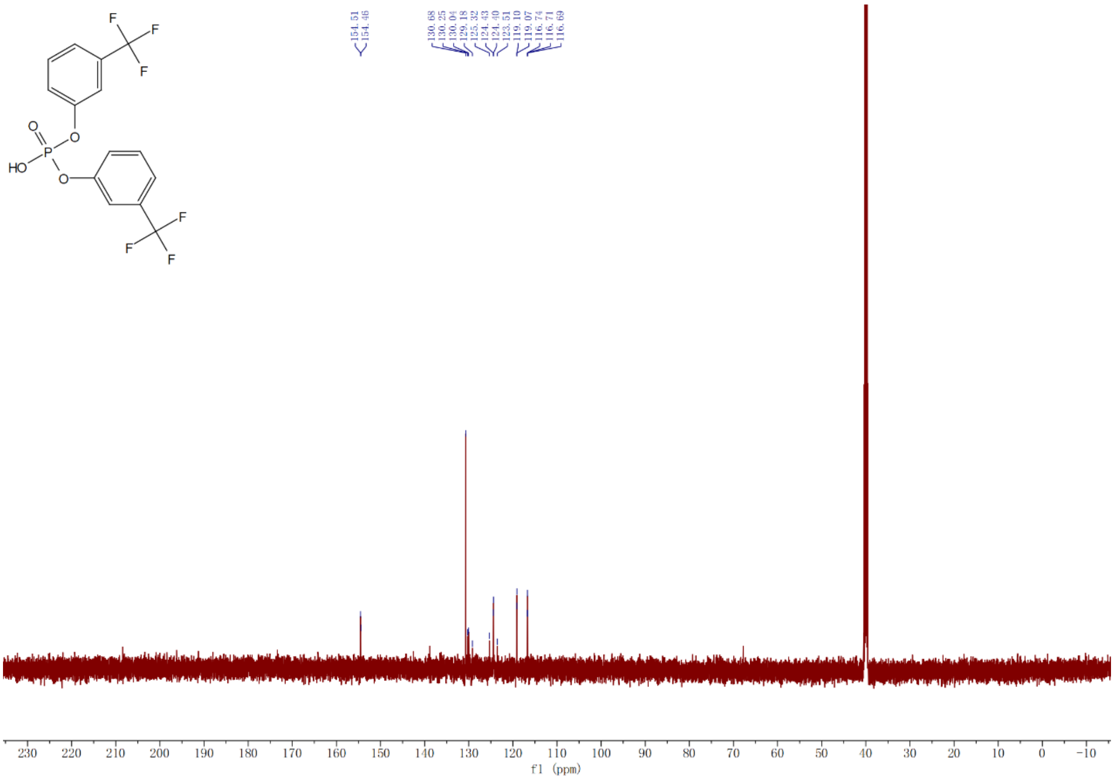

^{1}H NMR (400 MHz, CDCl$_3$)

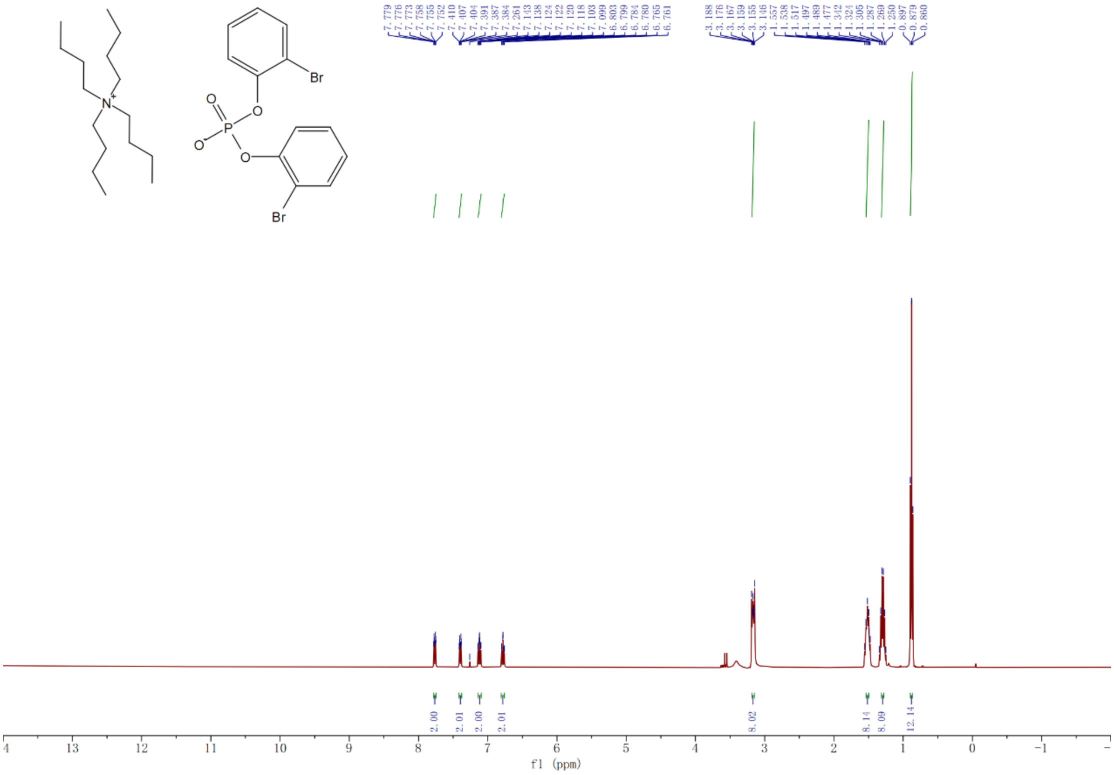

^{31}P NMR (400 MHz, $CDCl_3$)

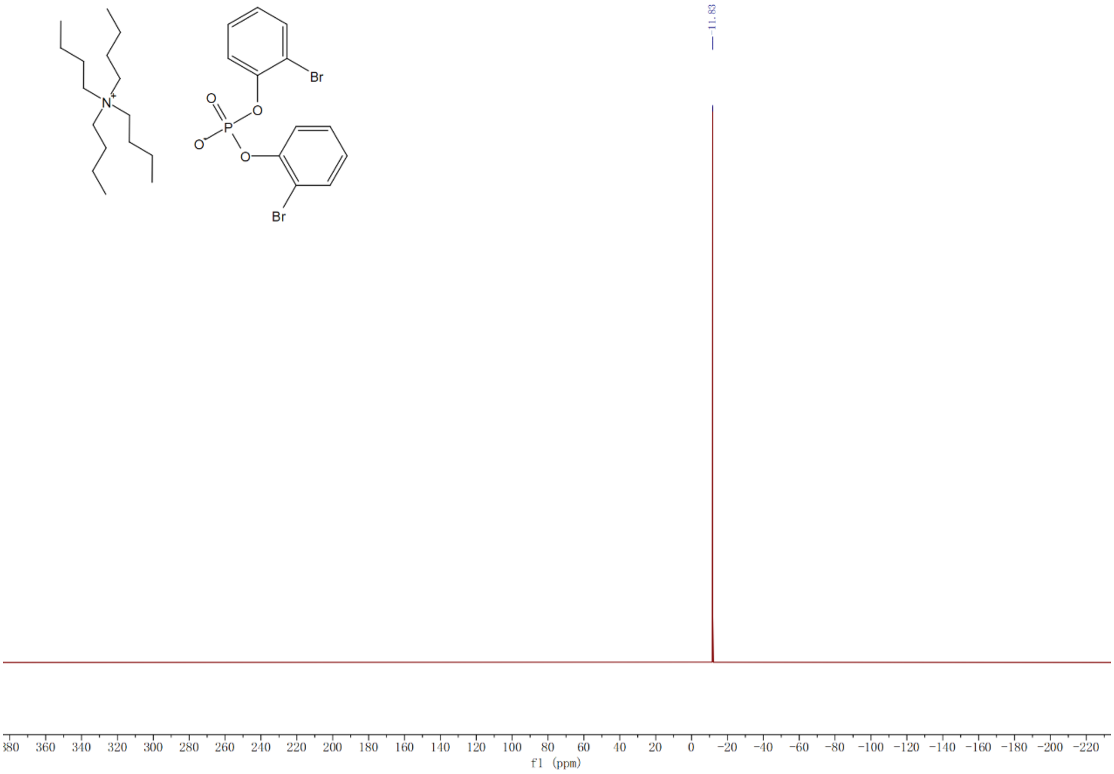

^{13}C NMR (600 MHz, $CDCl_3$)

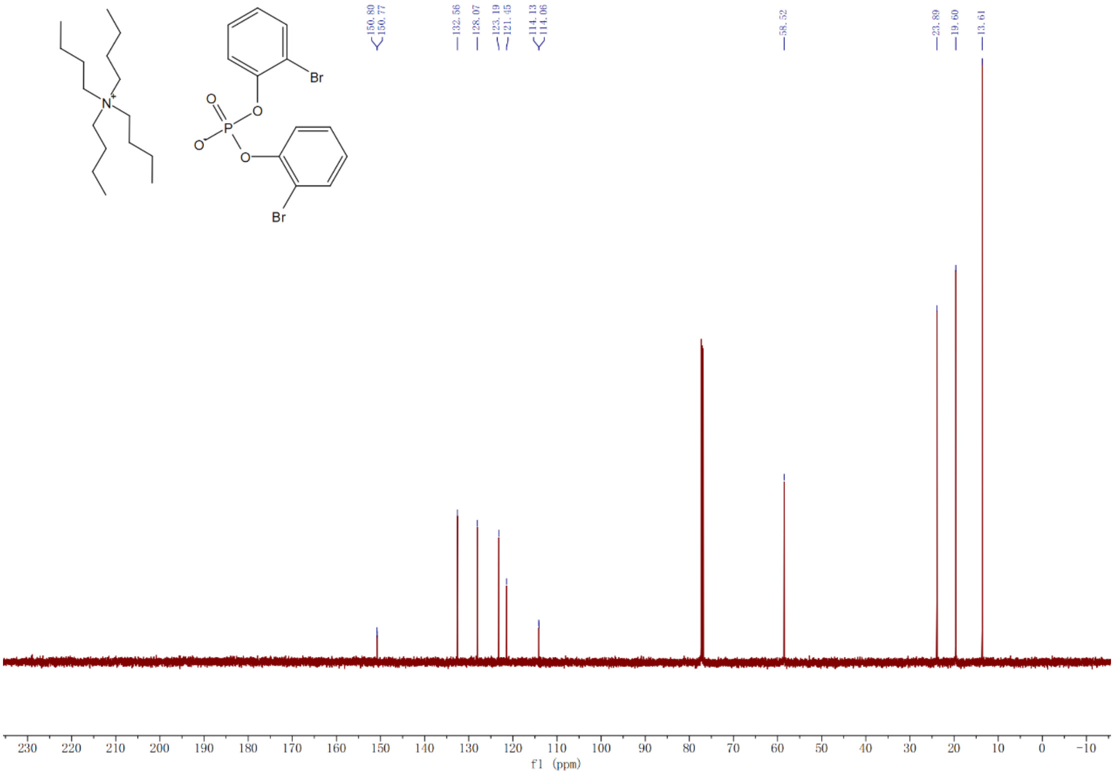

^{1}H NMR (400 MHz, $CDCl_3$)

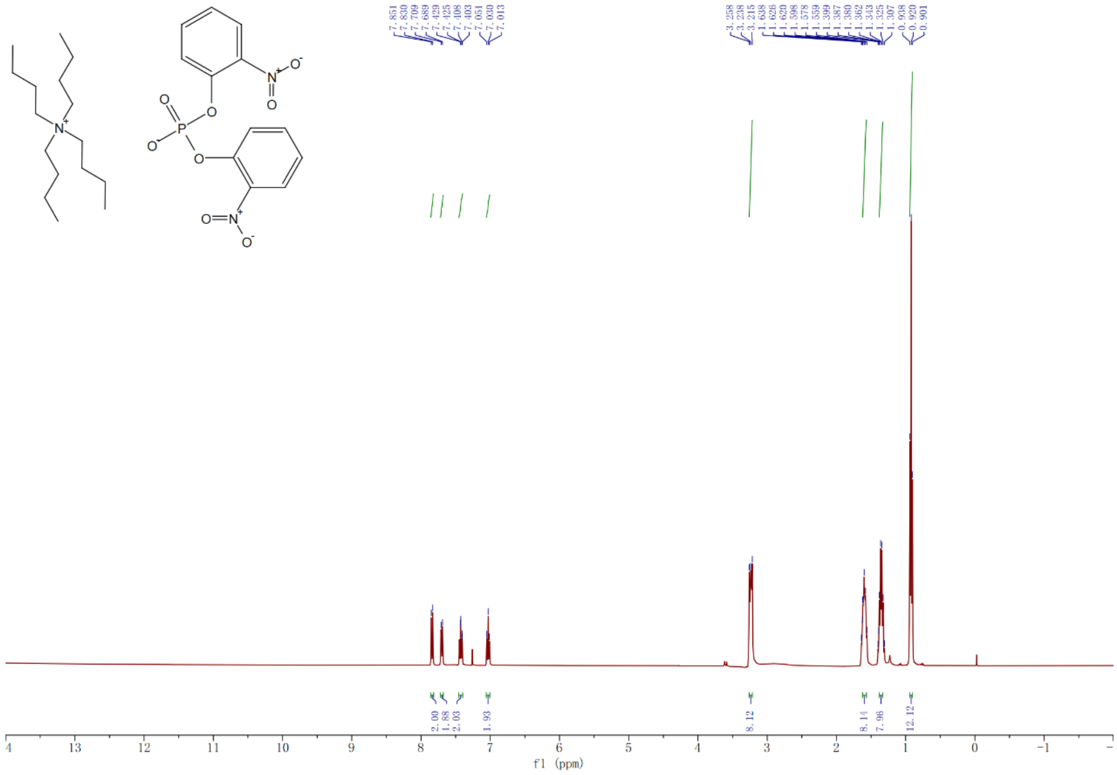

^{31}P NMR (400 MHz, $CDCl_3$)

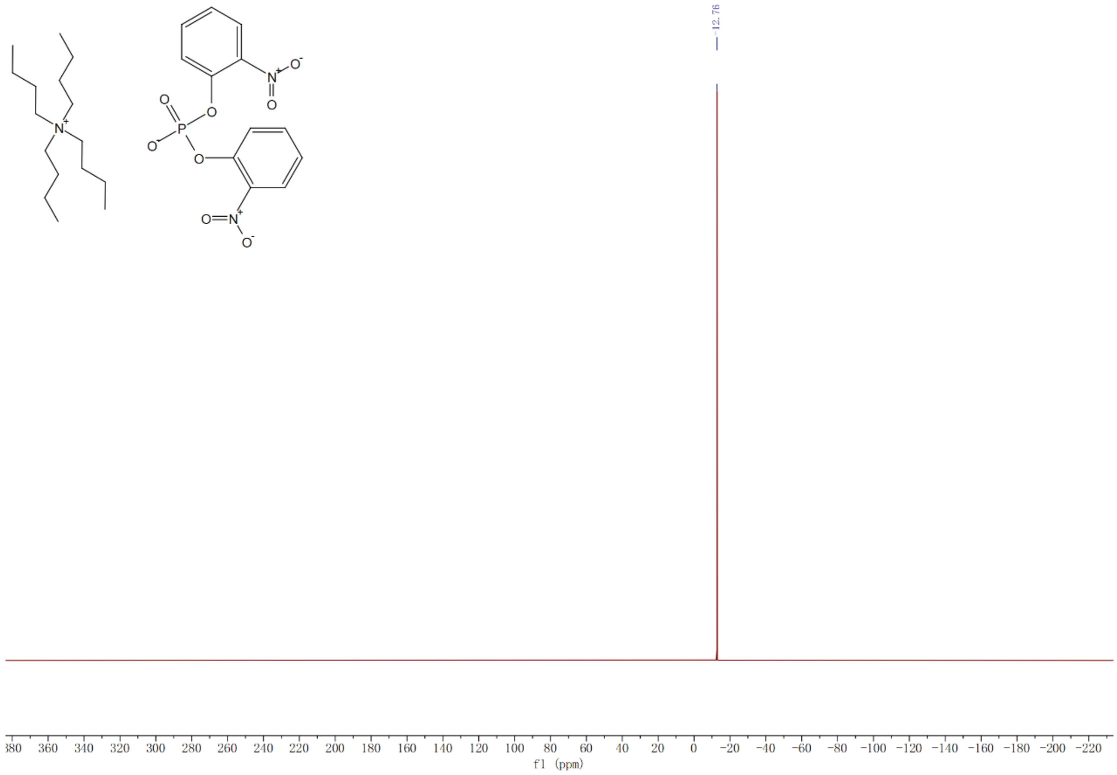

^{13}C NMR (600 MHz, $CDCl_3$)

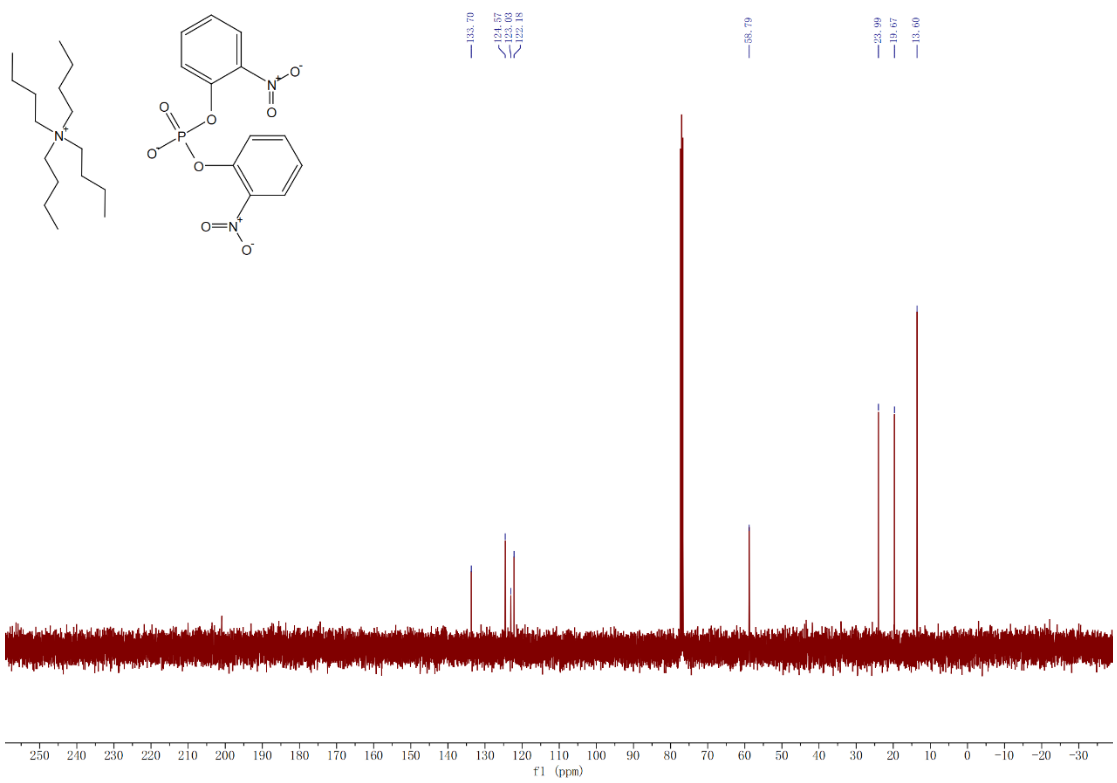

^{1}H NMR (400 MHz, DMSO-D_6)

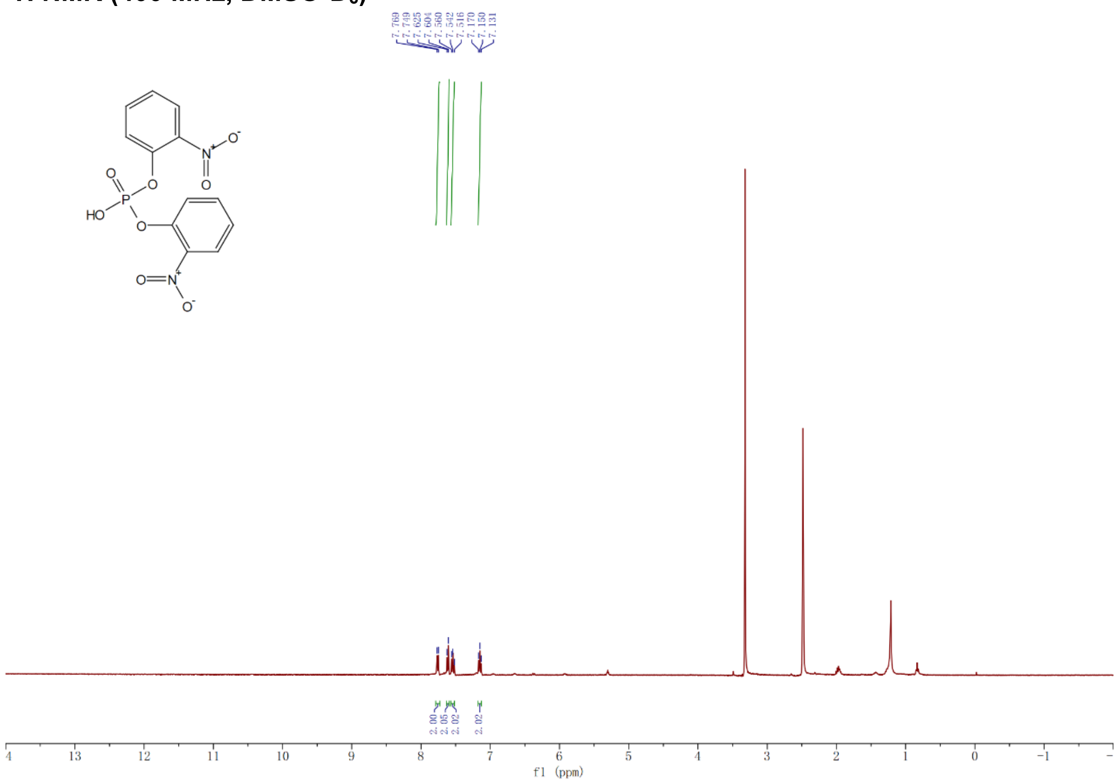

^{31}P NMR (400 MHz, DMSO-D$_6$)

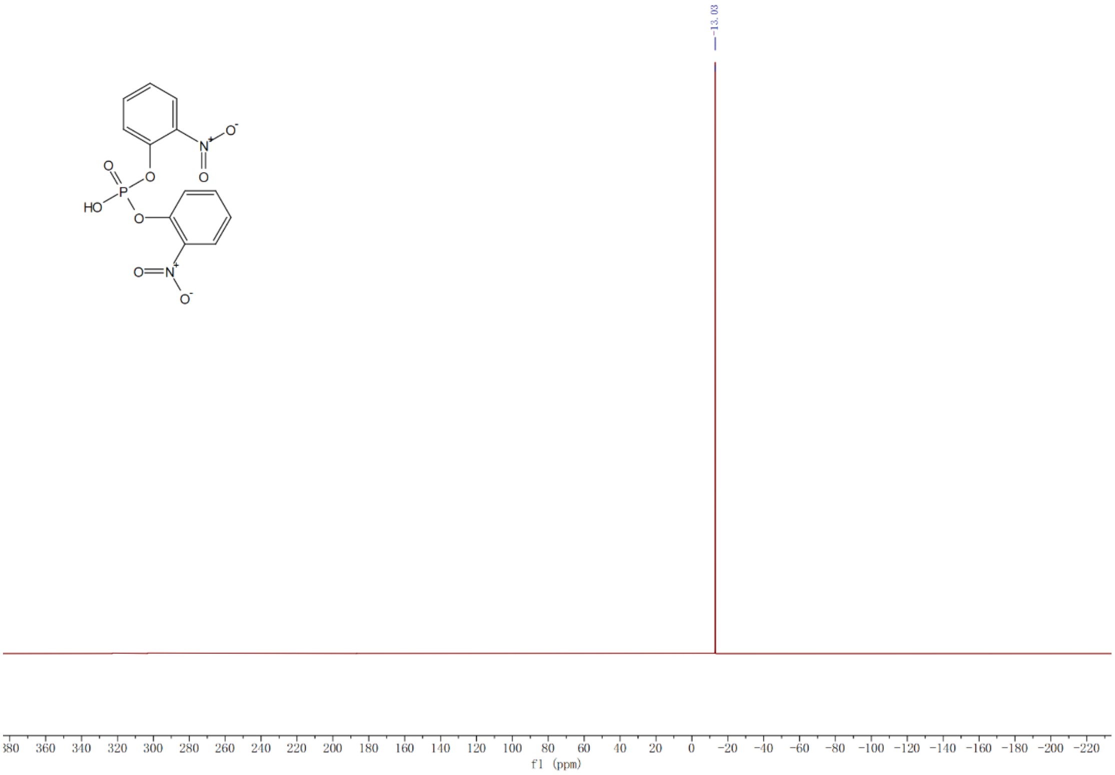

^{13}C NMR (600 MHz, DMSO-D$_6$)

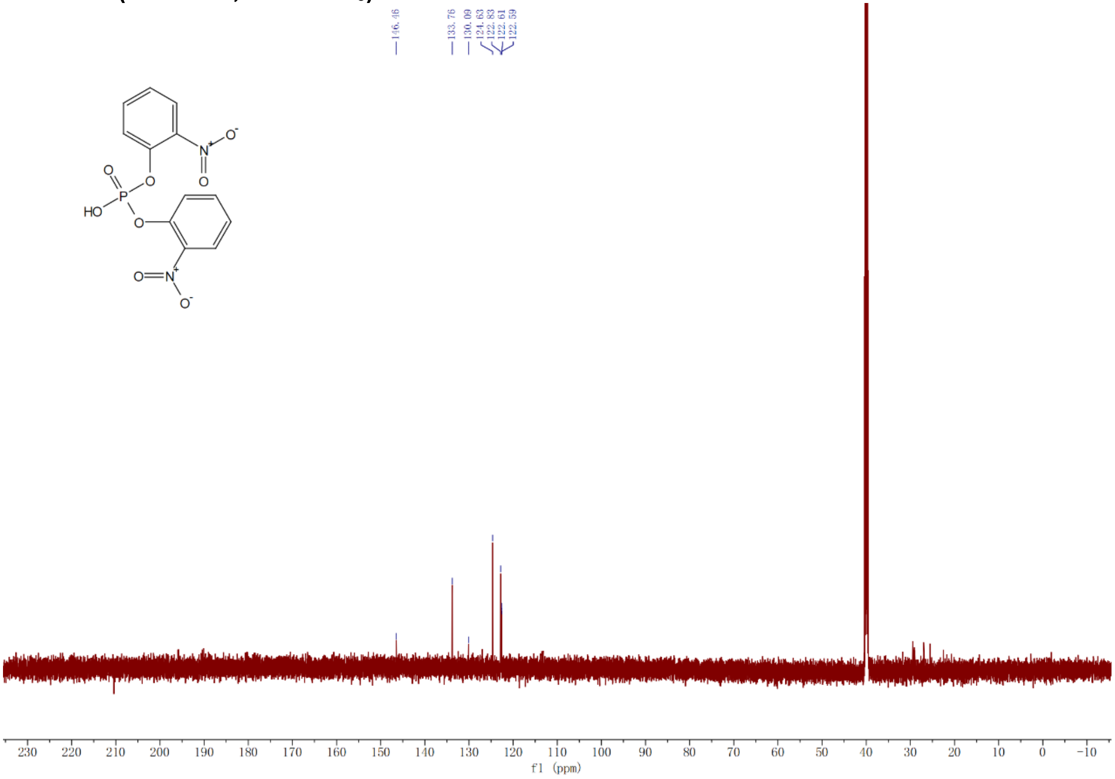

^1H NMR (400 MHz, $CDCl_3$)

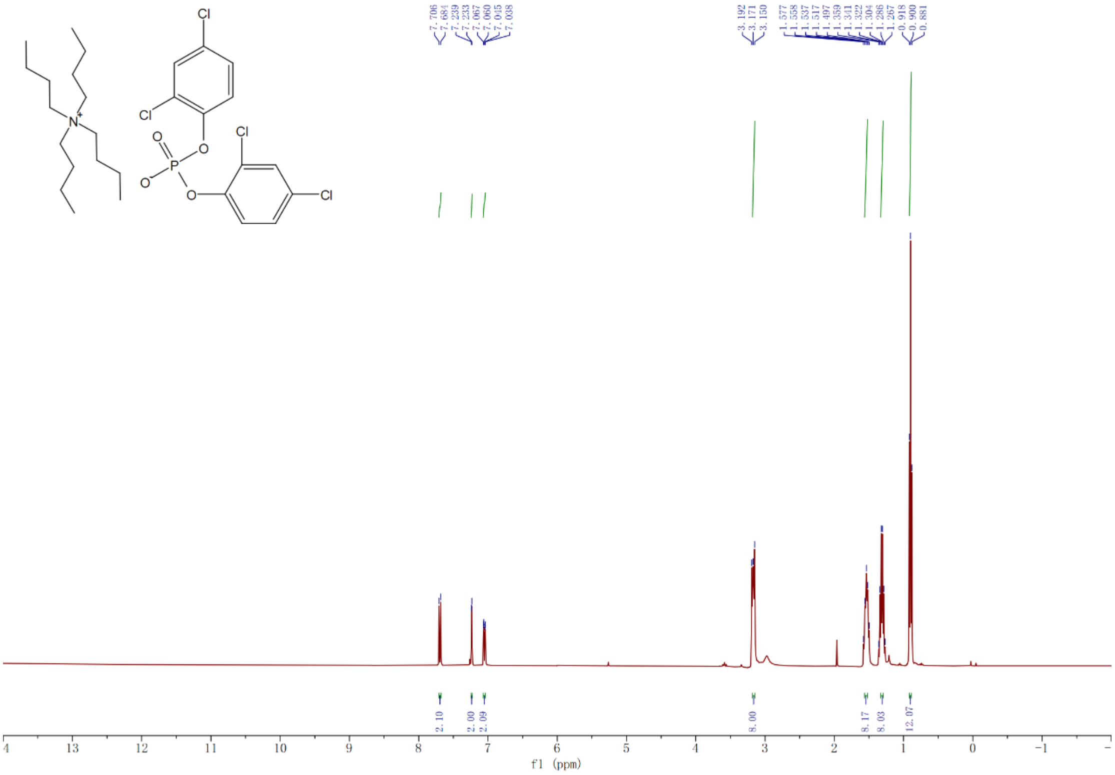

^{31}P NMR (400 MHz, $CDCl_3$)

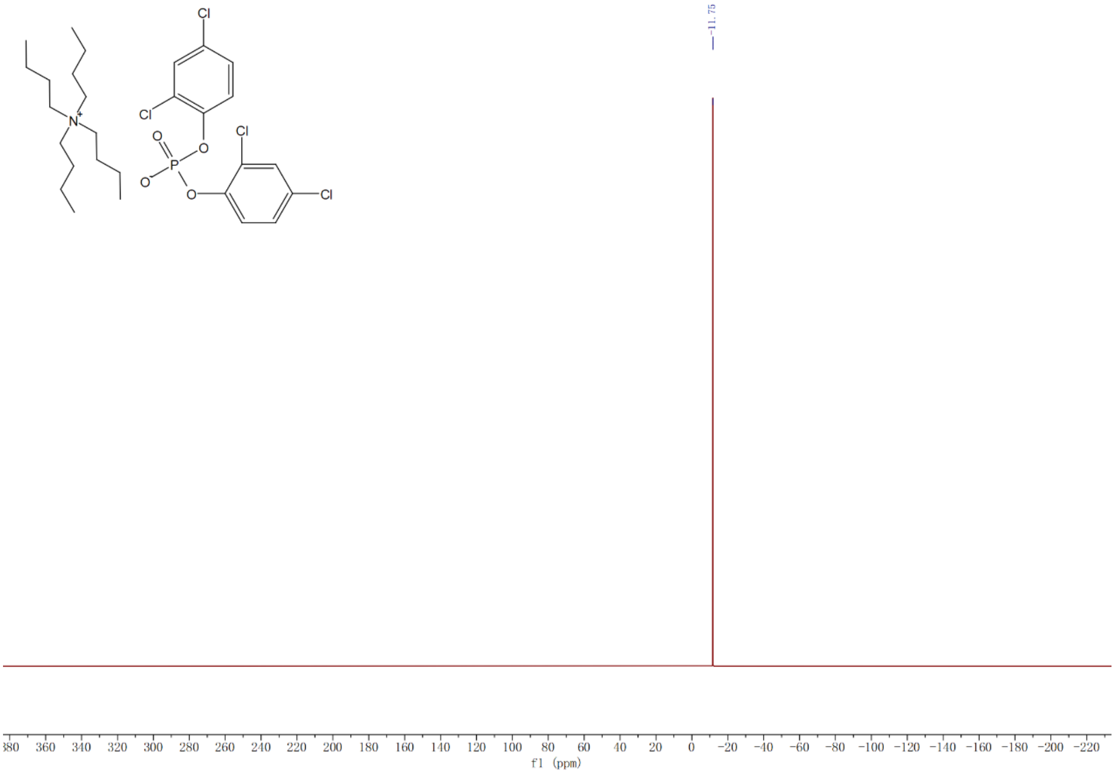

^{13}C NMR (600 MHz, $CDCl_3$)

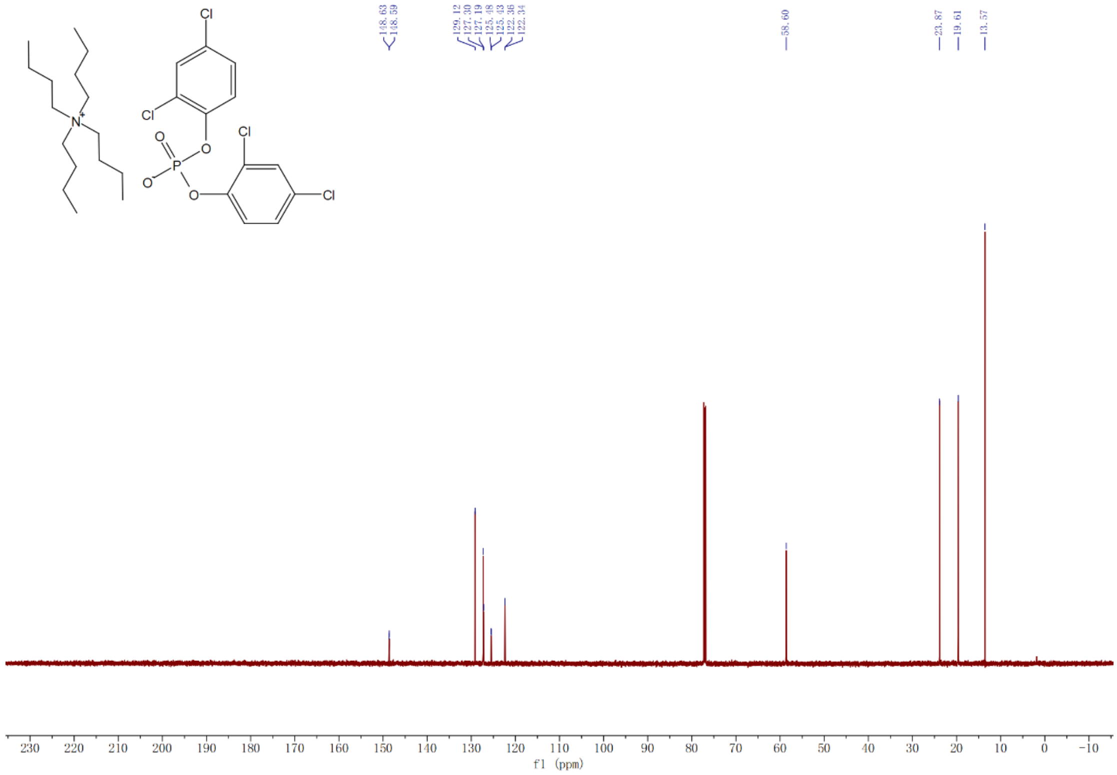

^{1}H NMR (400 MHz, DMSO-D_6)

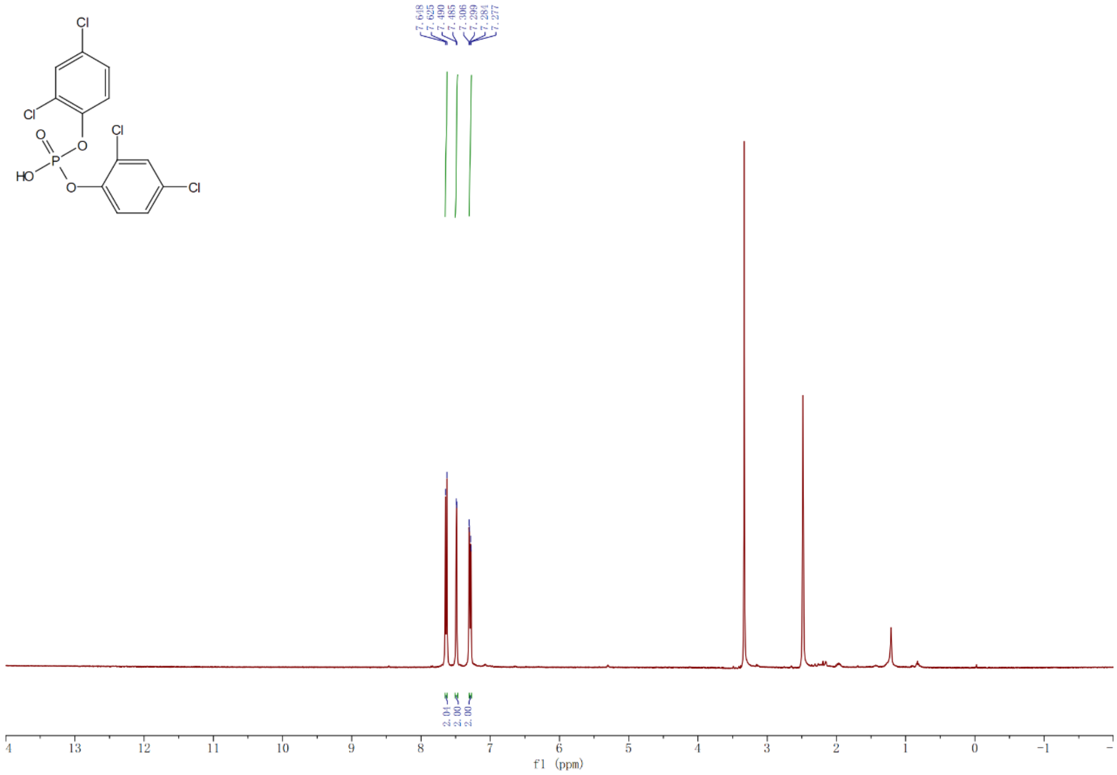

^{31}P NMR (400 MHz, DMSO-D$_6$)

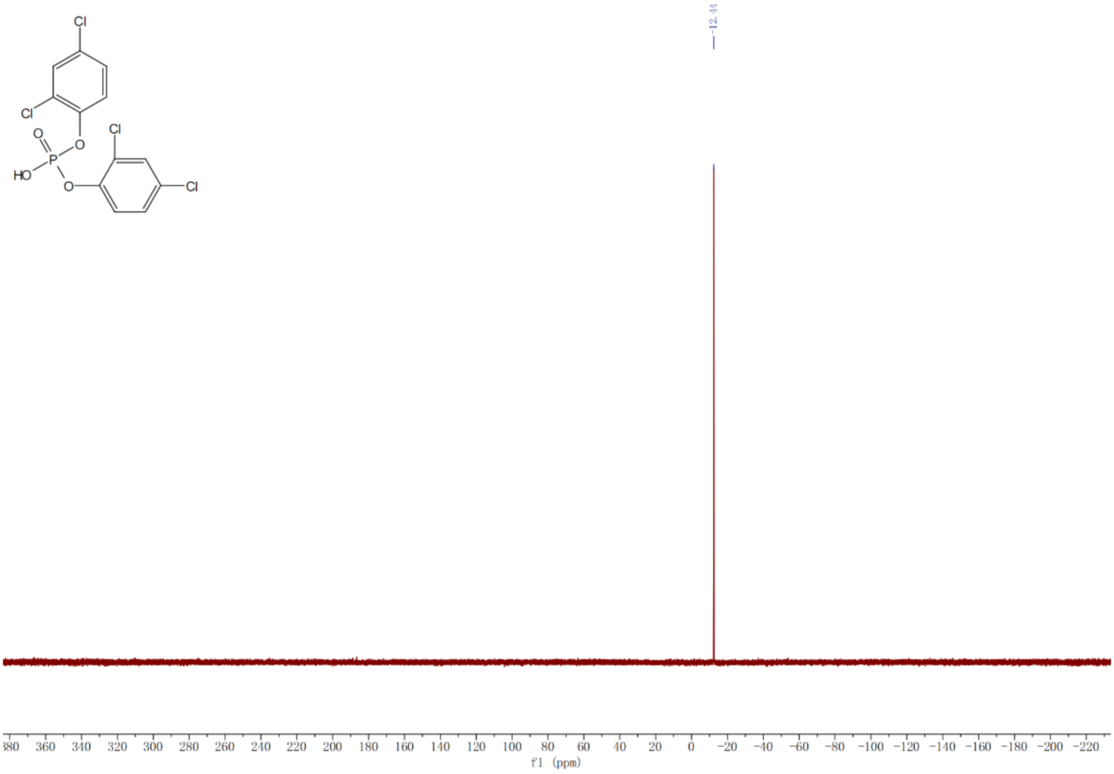

^{13}C NMR (600 MHz, DMSO-D$_6$)

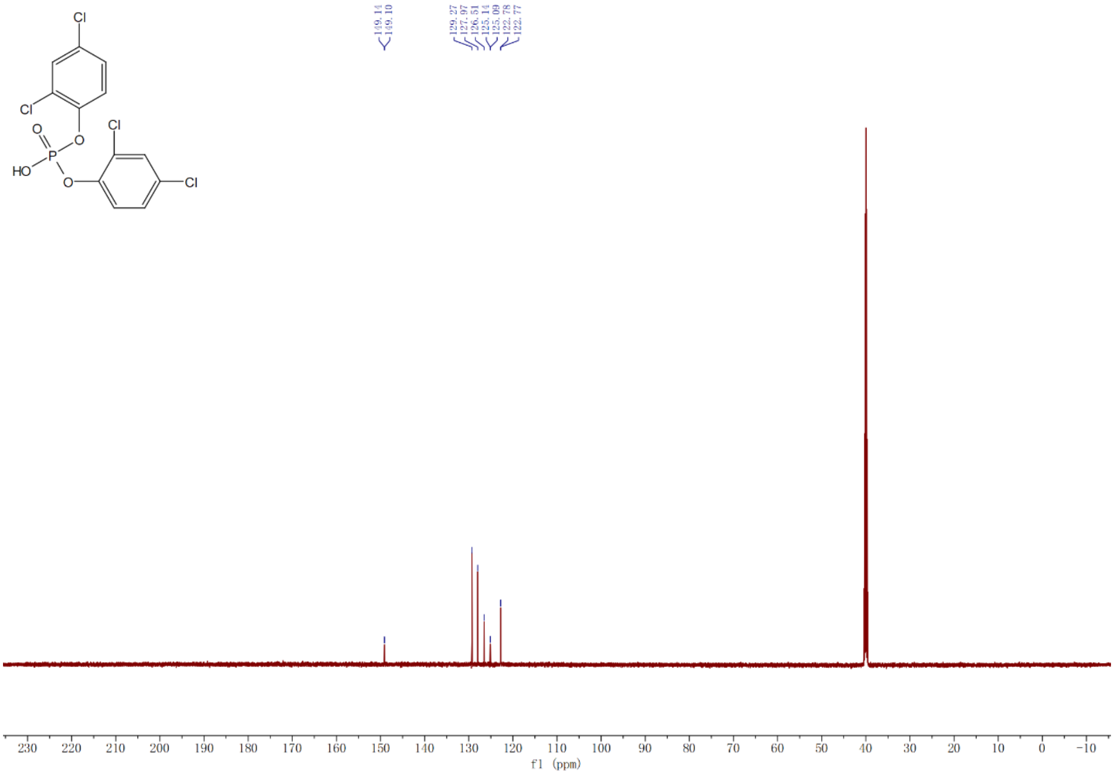

^1H NMR (400 MHz, $CDCl_3$)

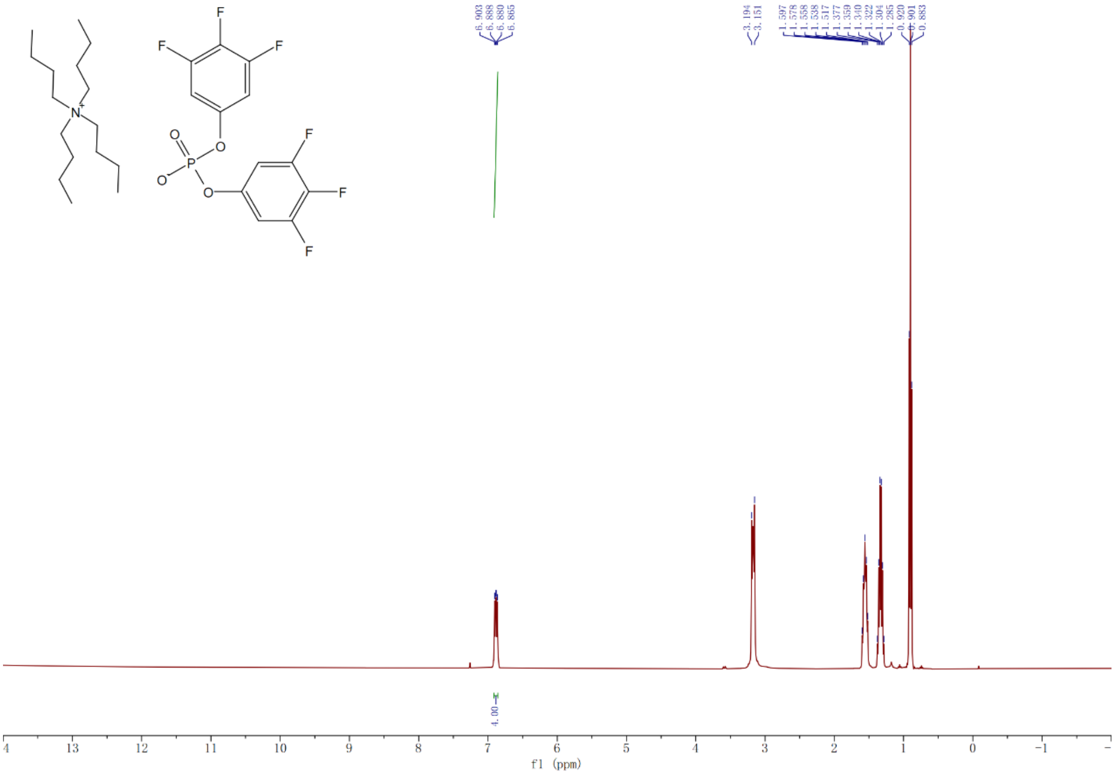

^{31}P NMR (400 MHz, $CDCl_3$)

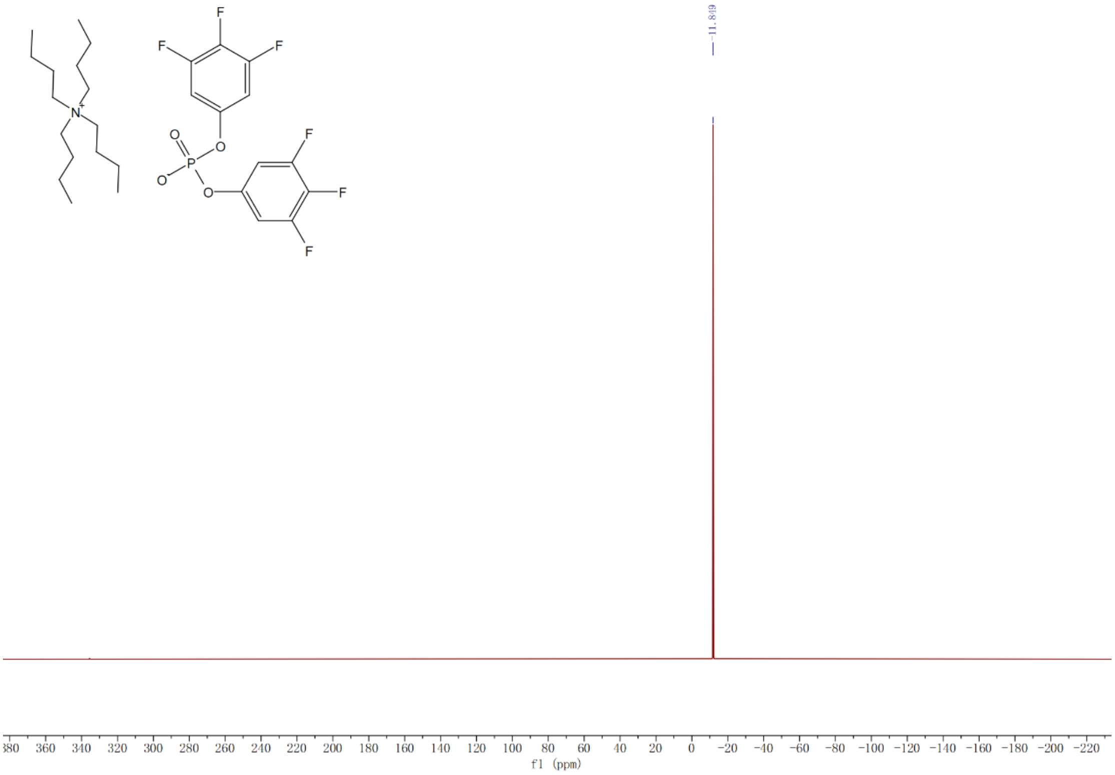

^{19}F NMR (600 MHz, $CDCl_3$)

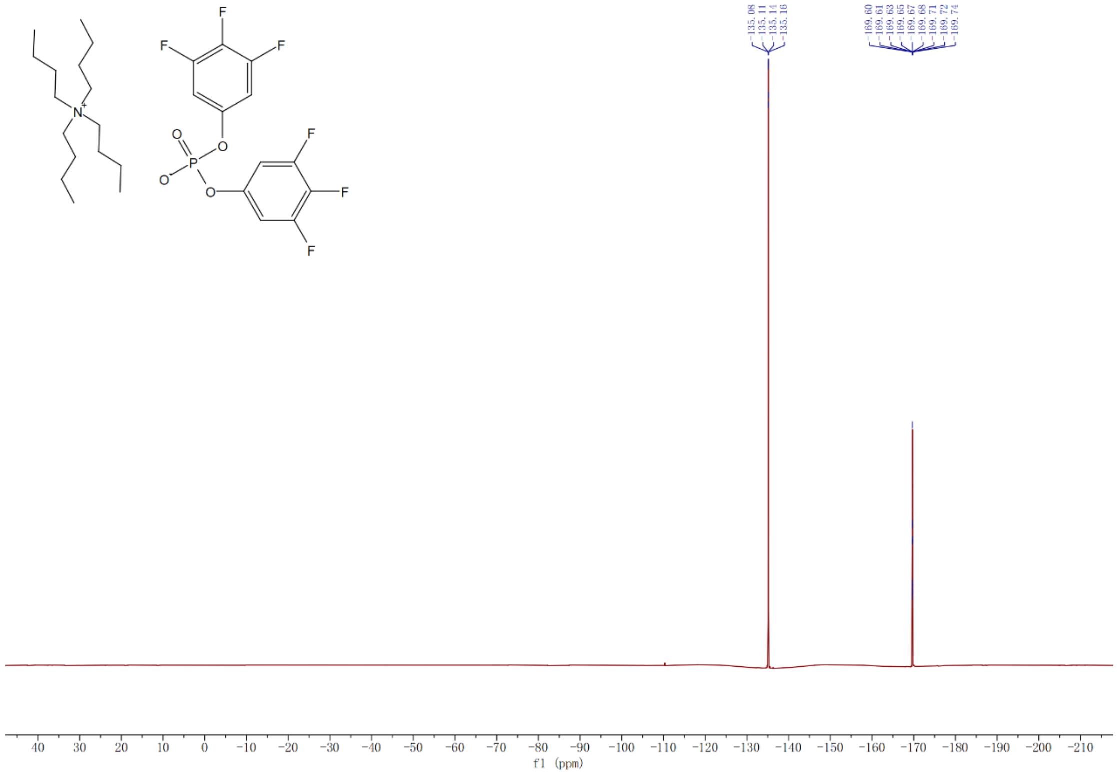

^{13}C NMR (600 MHz, $CDCl_3$)

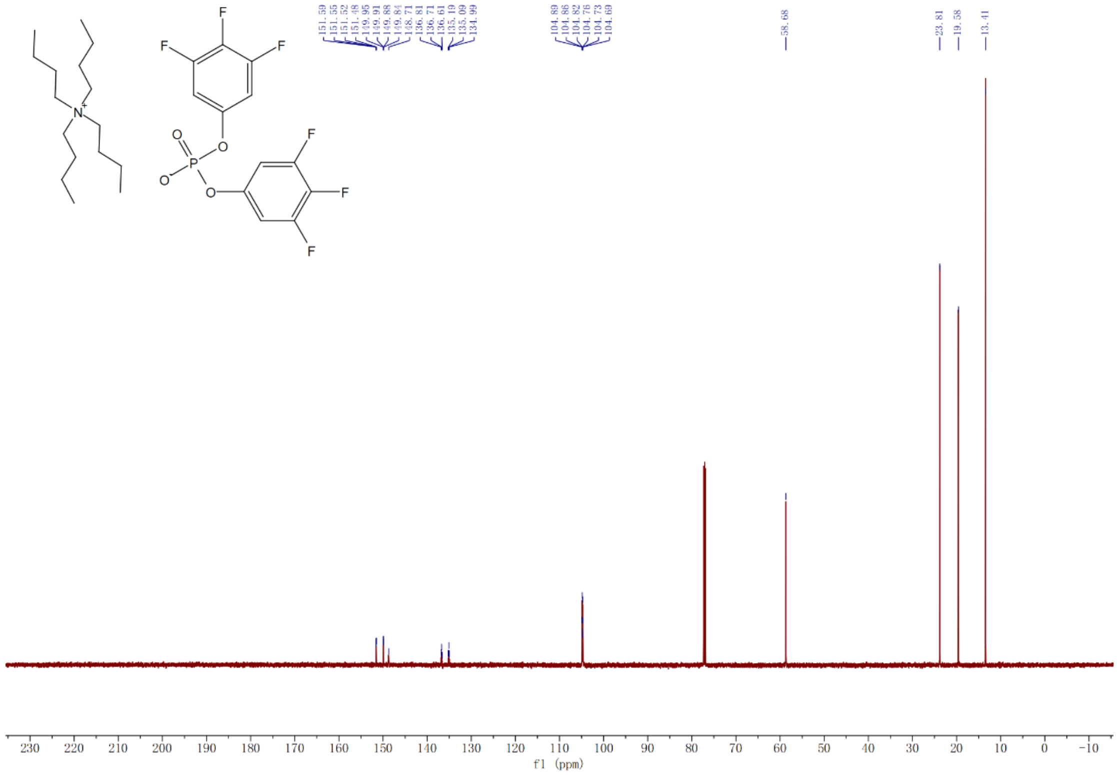

^{1}H NMR (400 MHz, DMSO-D_6)

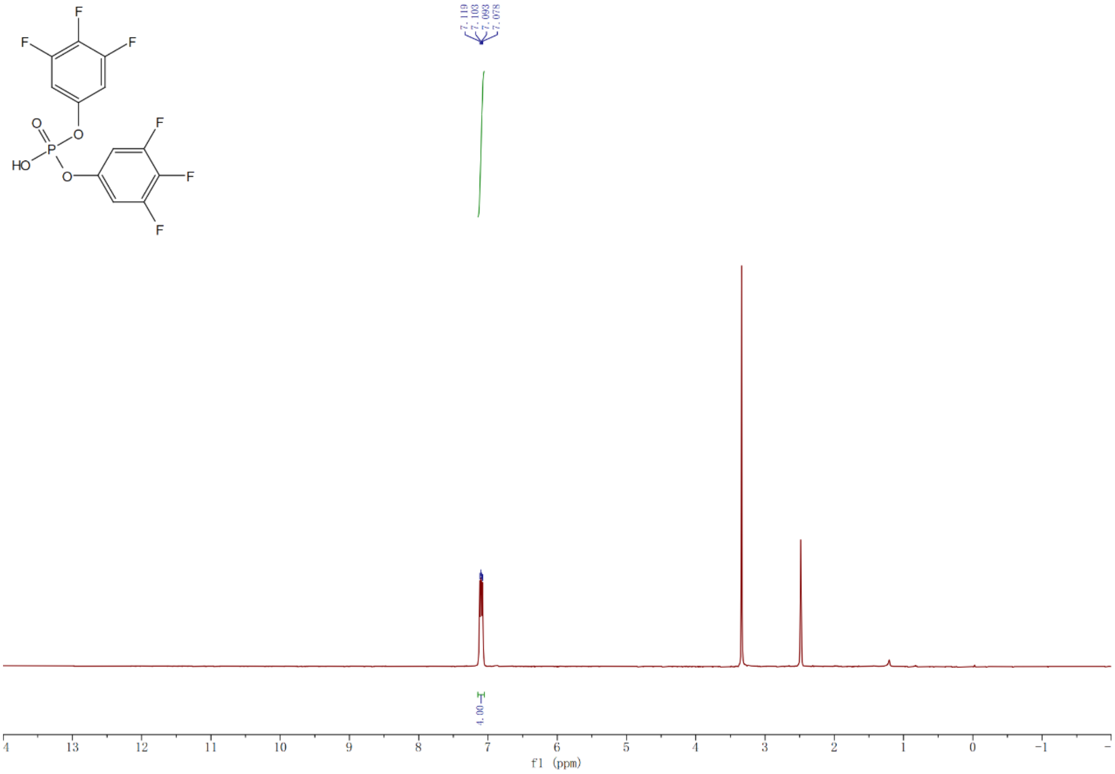

^{31}P NMR (400 MHz, DMSO-D_6))

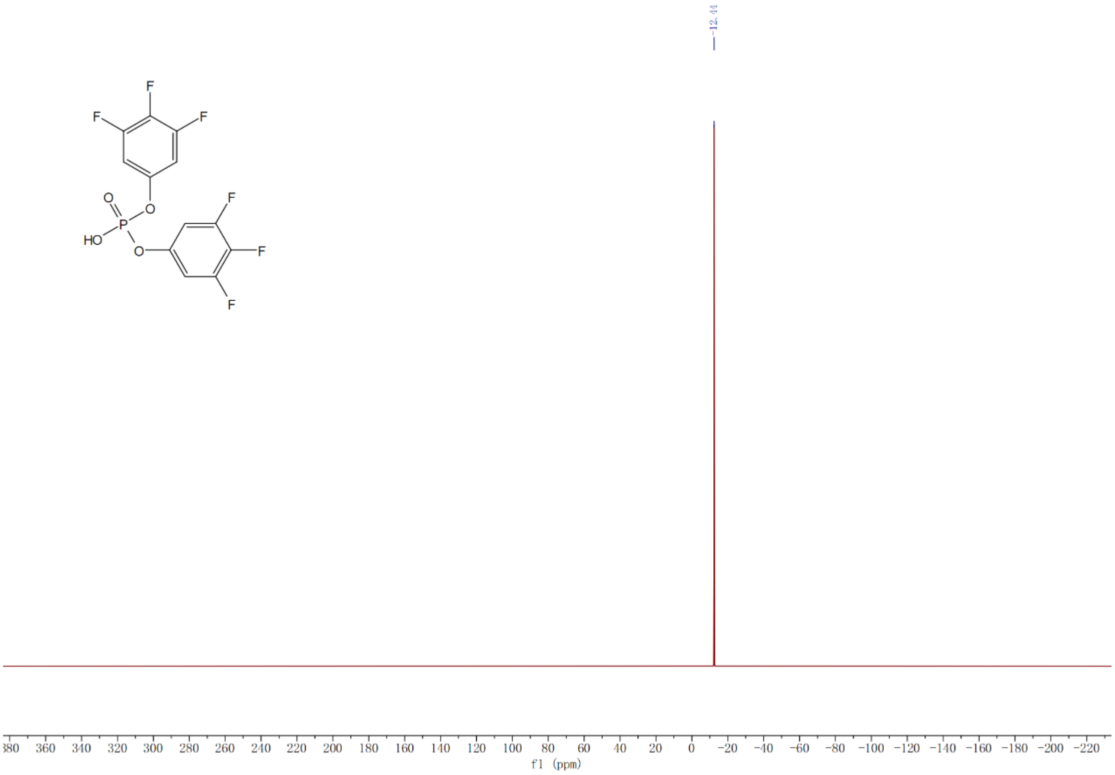

^{19}F NMR (400 MHz, DMSO-D$_6$)

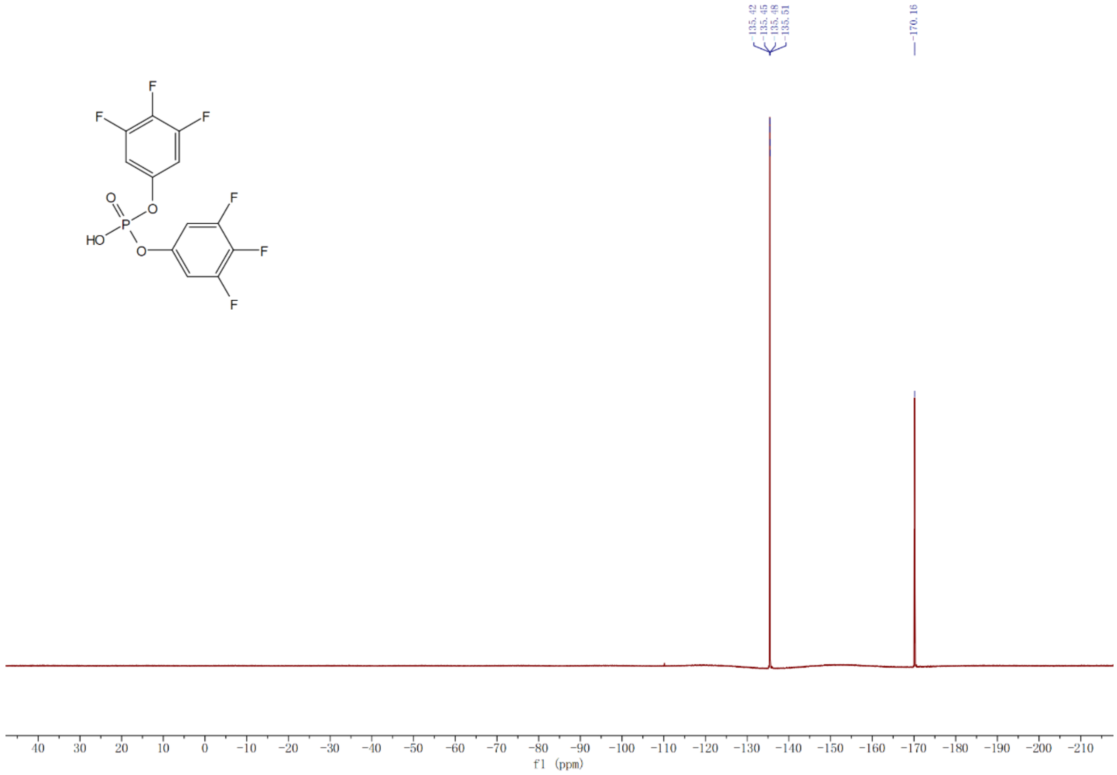

^{13}C NMR (600 MHz, DMSO-D$_6$)

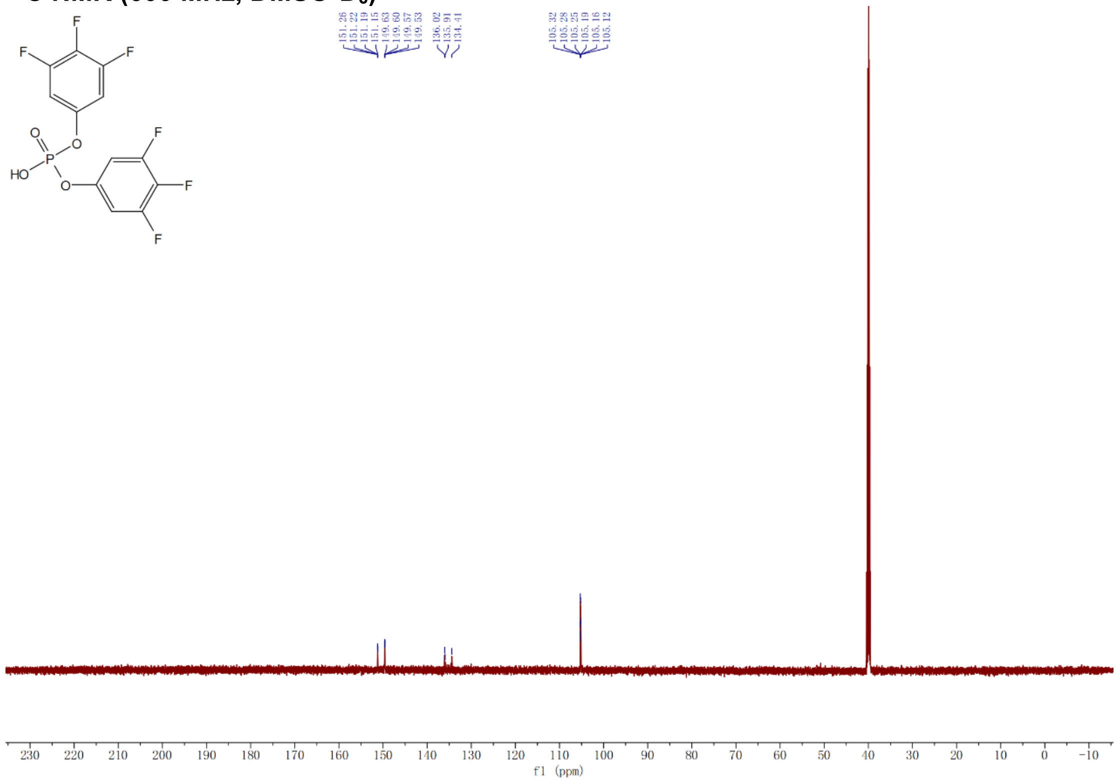

^{1}H NMR (400 MHz, $CDCl_3$)

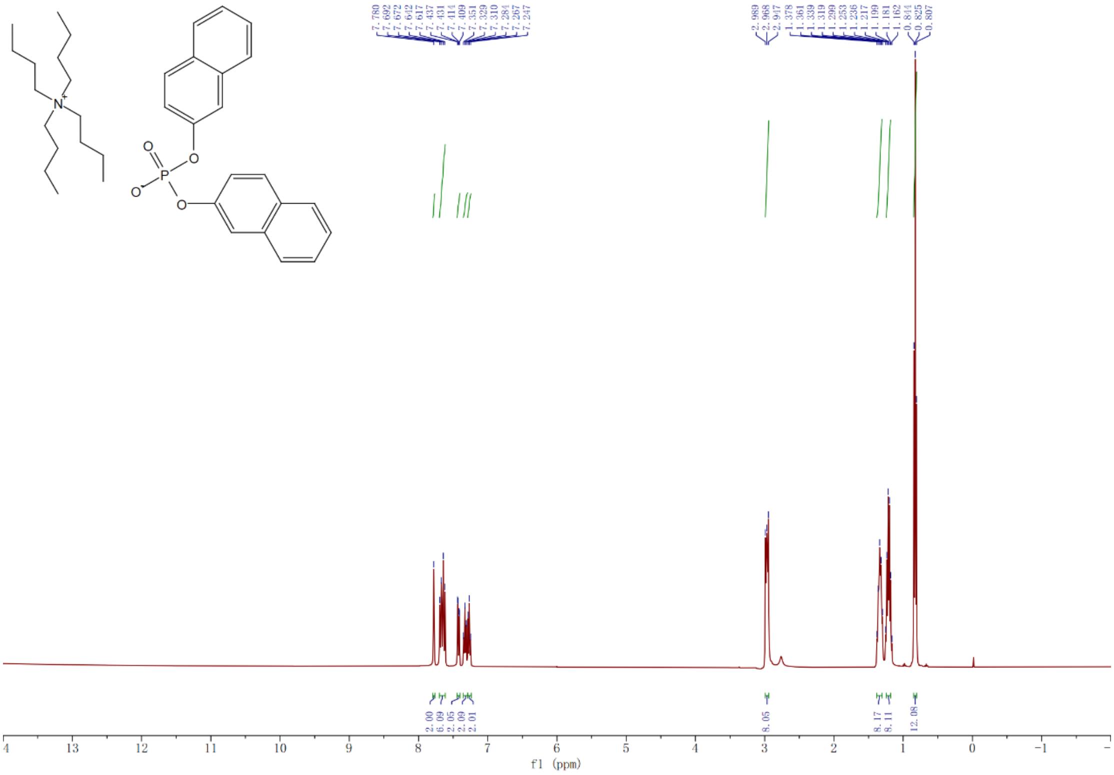

^{31}P NMR (400 MHz, $CDCl_3$)

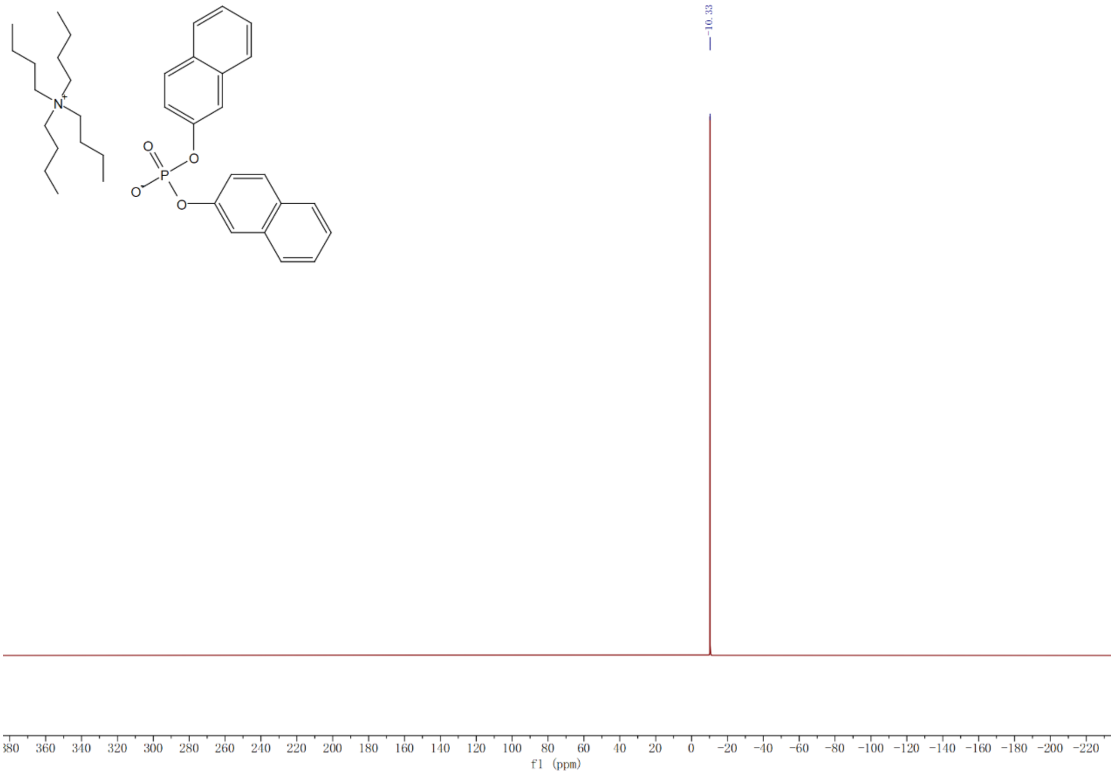

^{13}C NMR (600 MHz, $CDCl_3$)

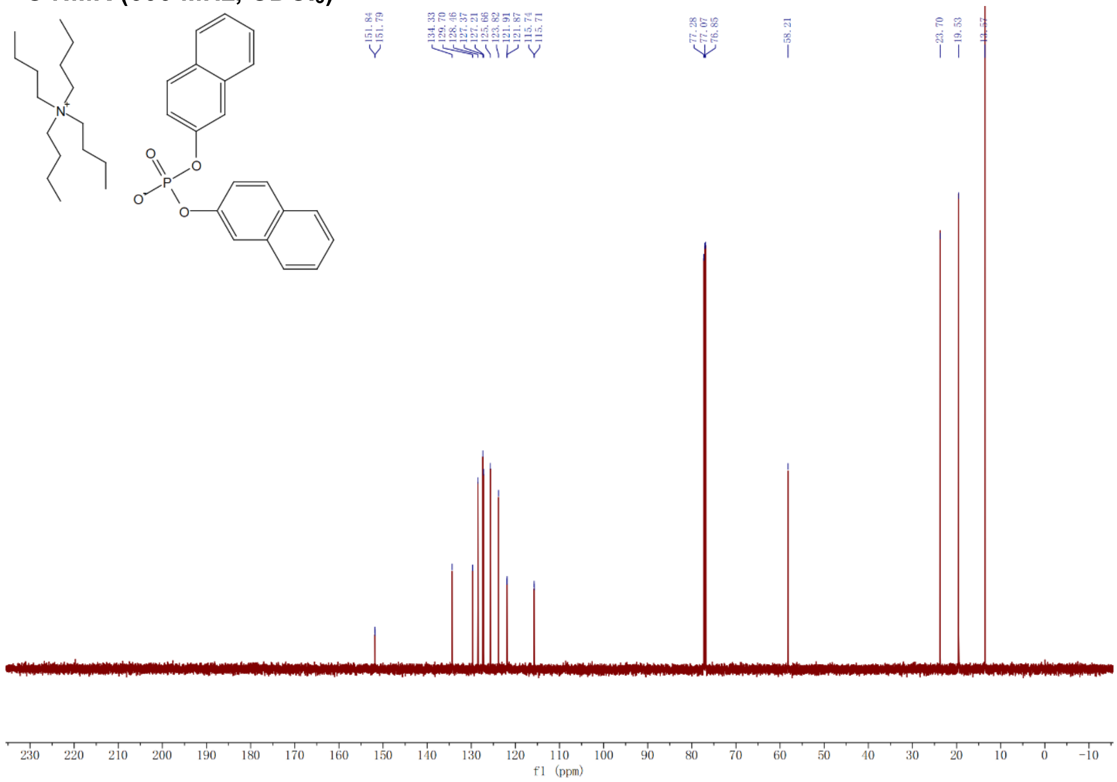

^{1}H NMR (400 MHz, $CDCl_3$)

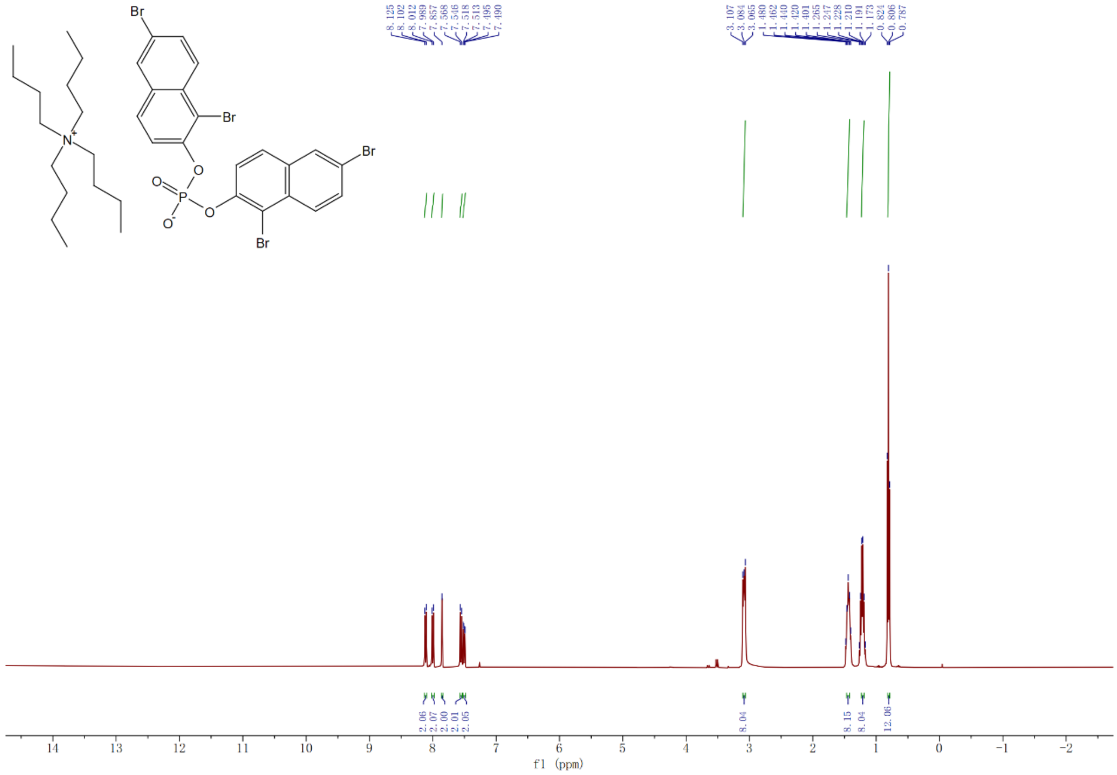

^{31}P NMR (400 MHz, $CDCl_3$)

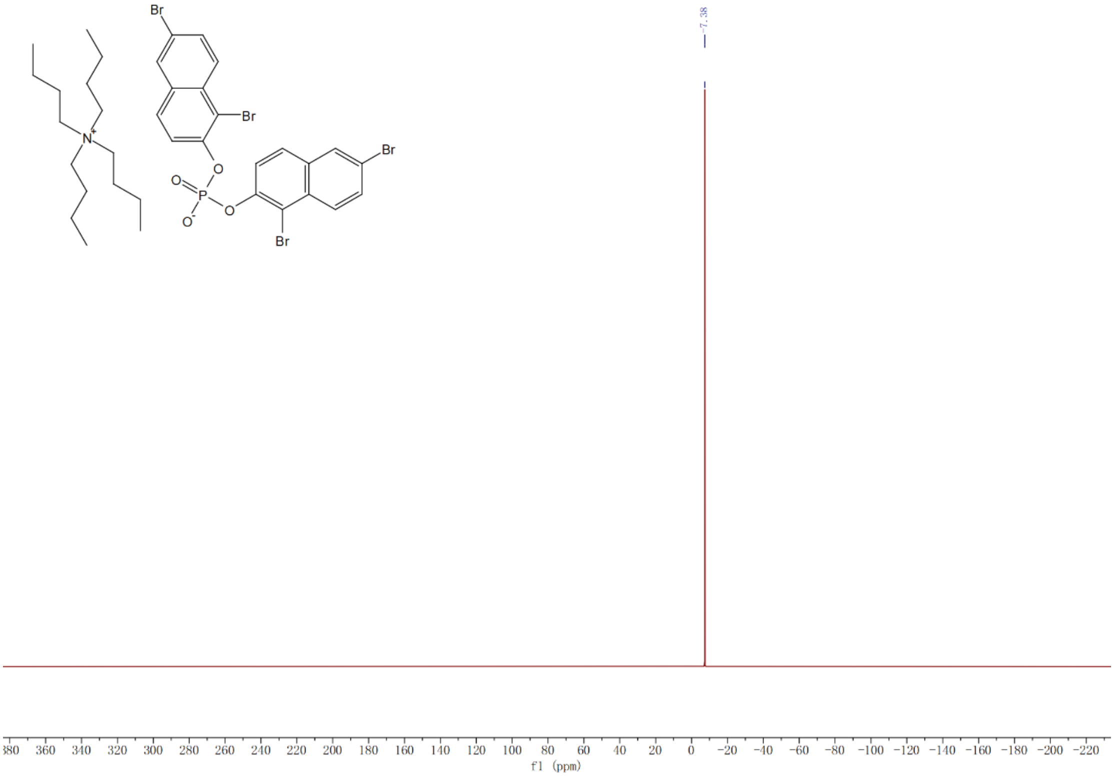

^{13}C NMR (600 MHz, $CDCl_3$)

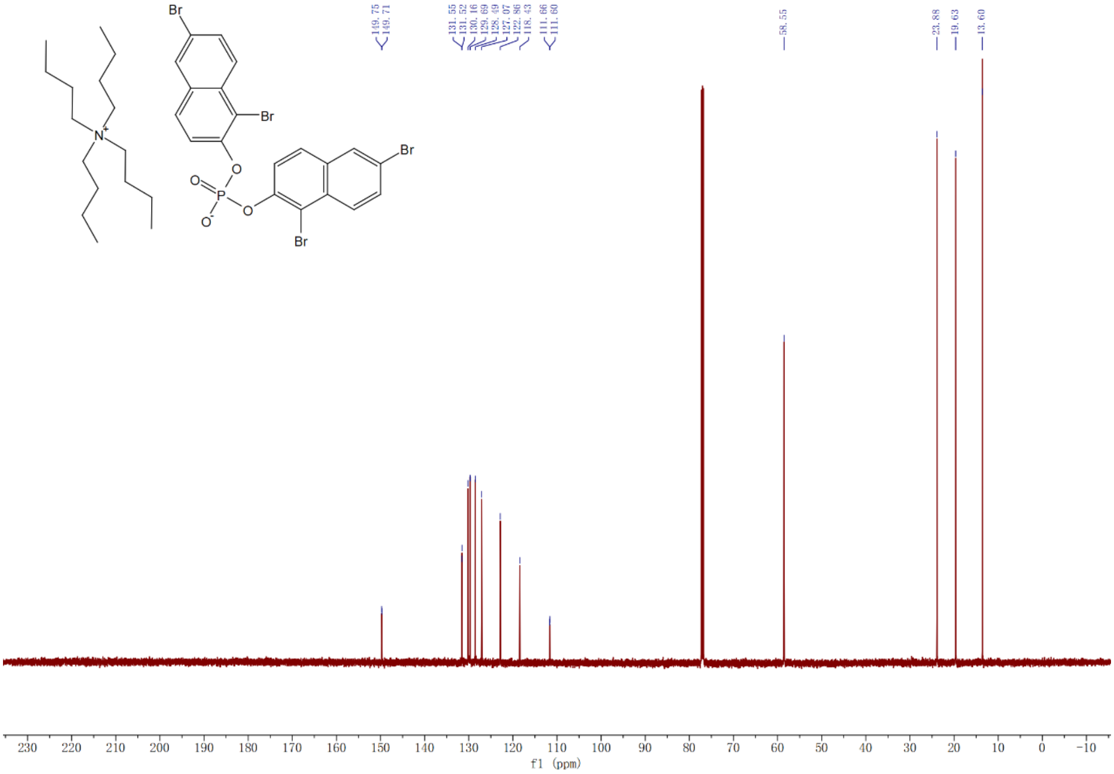

^{1}H NMR (400 MHz, $CDCl_3$)

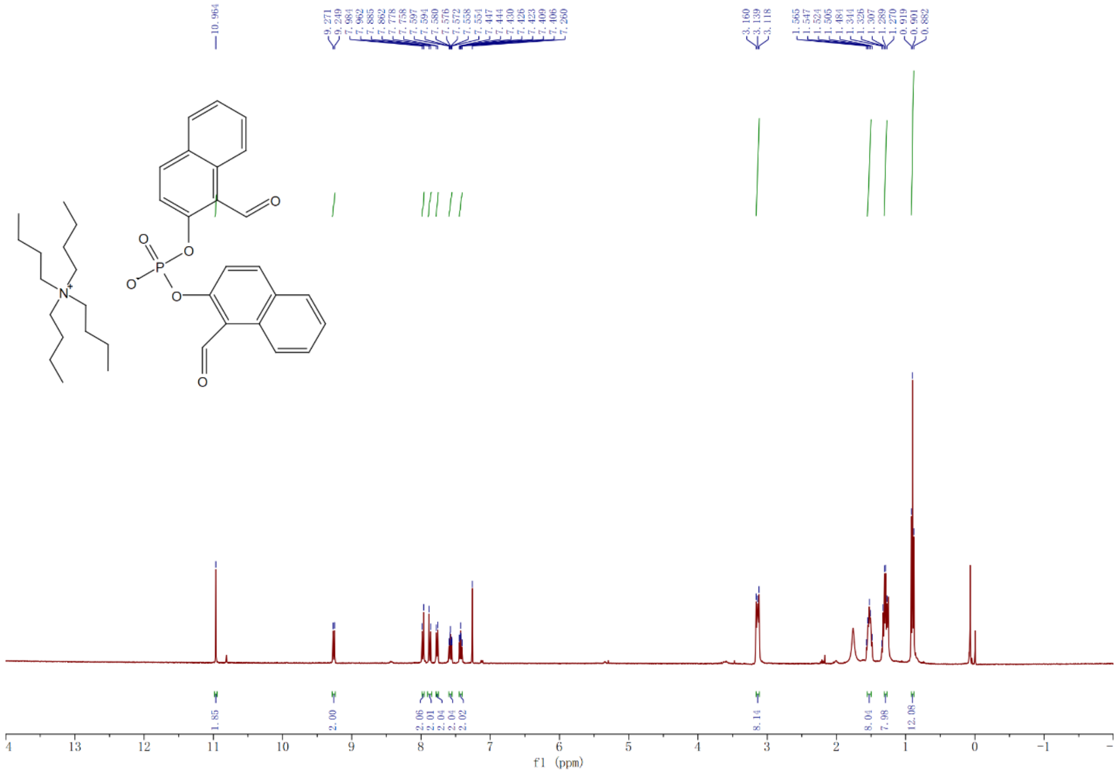

^{31}P NMR (400 MHz, $CDCl_3$)

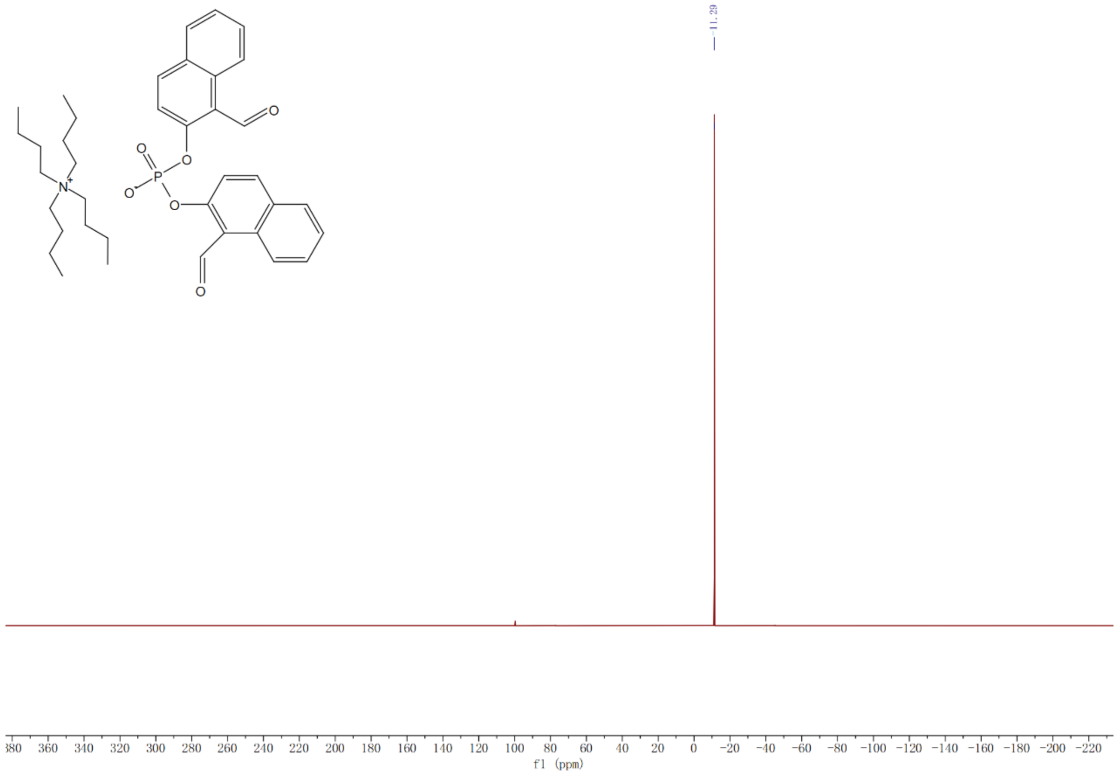

^{13}C NMR (600 MHz, $CDCl_3$)

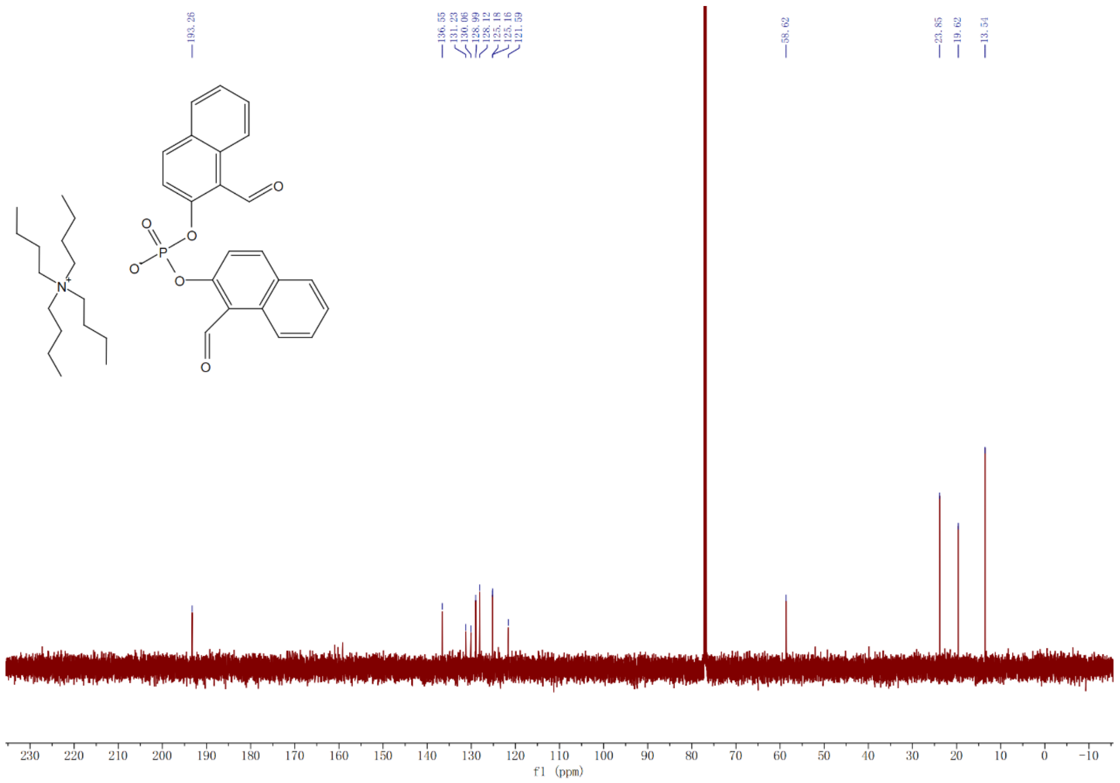

^{1}H NMR (400 MHz, $CDCl_3$)

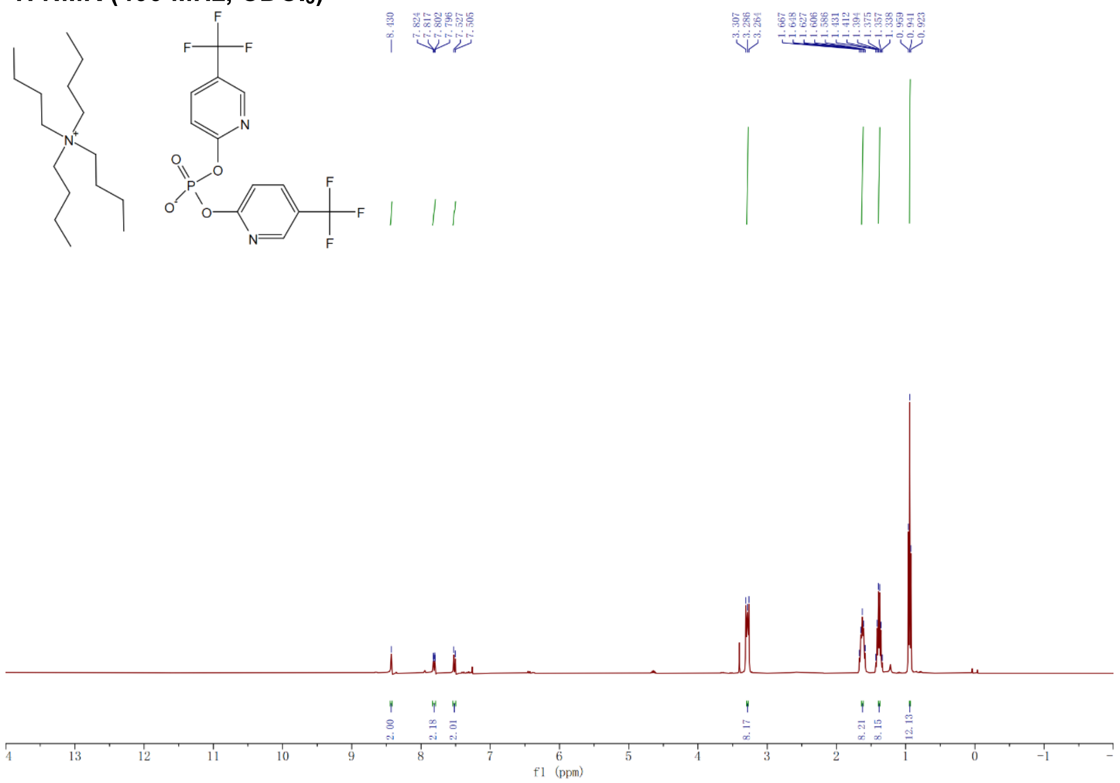

^{31}P NMR (400 MHz, $CDCl_3$)

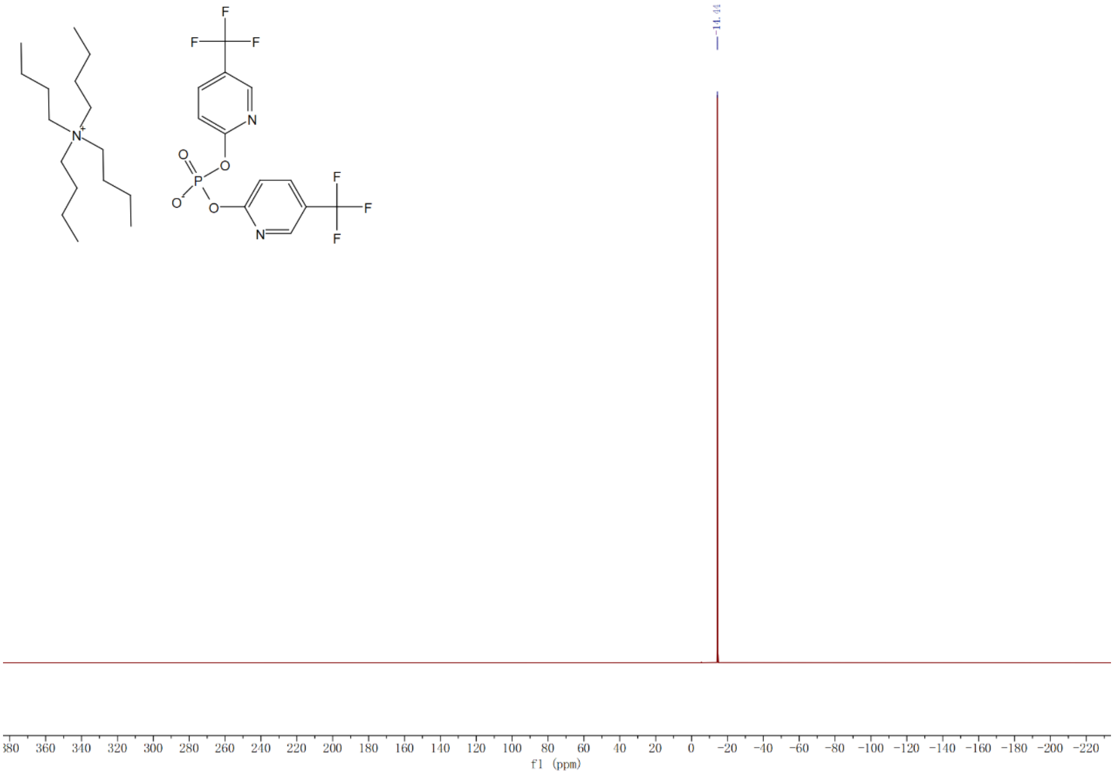

^{13}C NMR (600 MHz, $CDCl_3$)

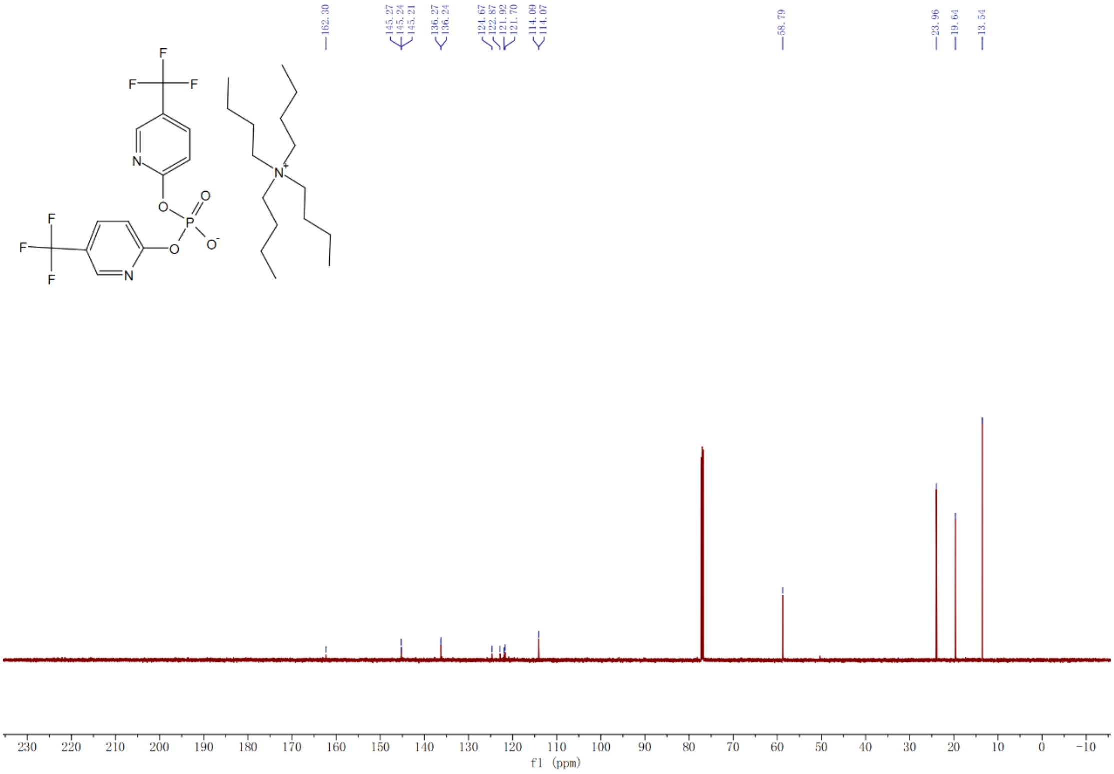

^{1}H NMR (400 MHz, $CDCl_3$)

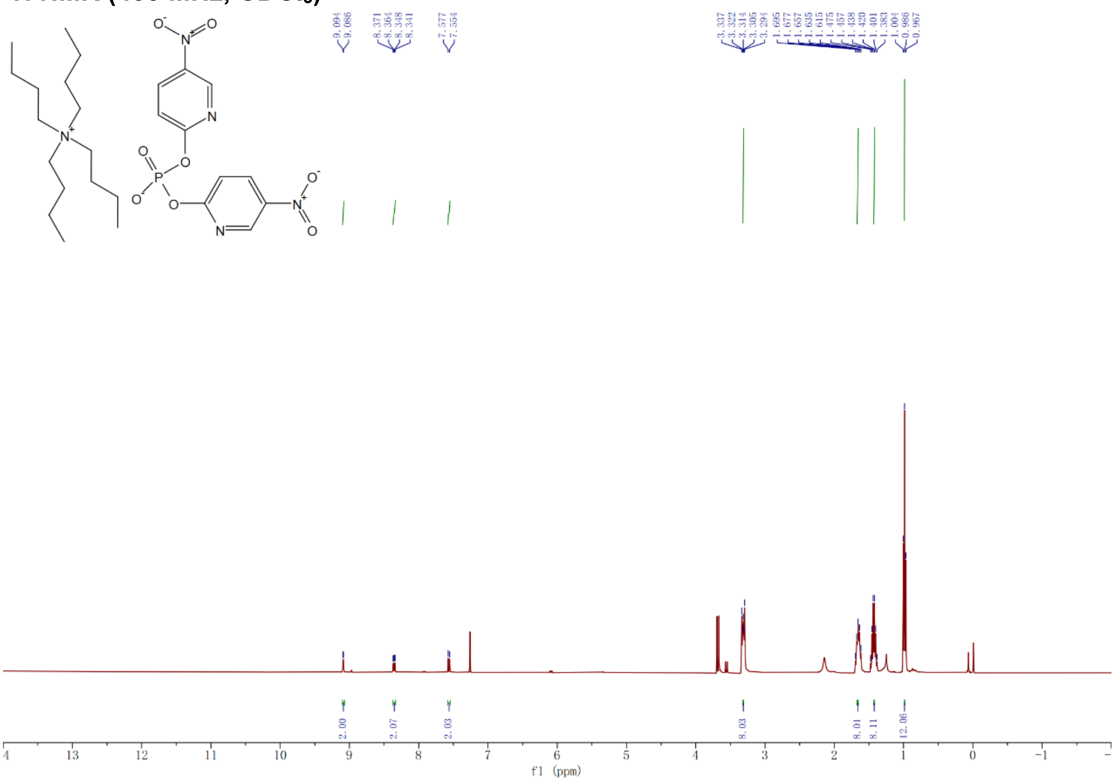

^{31}P NMR (400 MHz, $CDCl_3$)

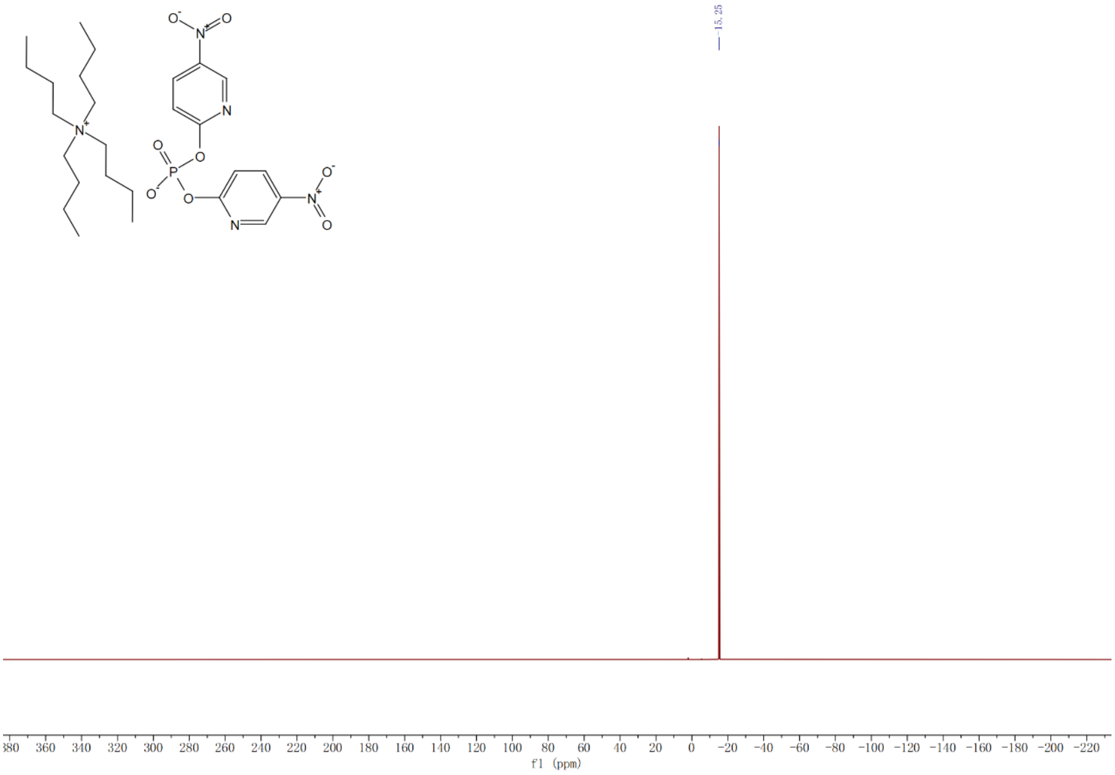

^{13}C NMR (600 MHz, $CDCl_3$)

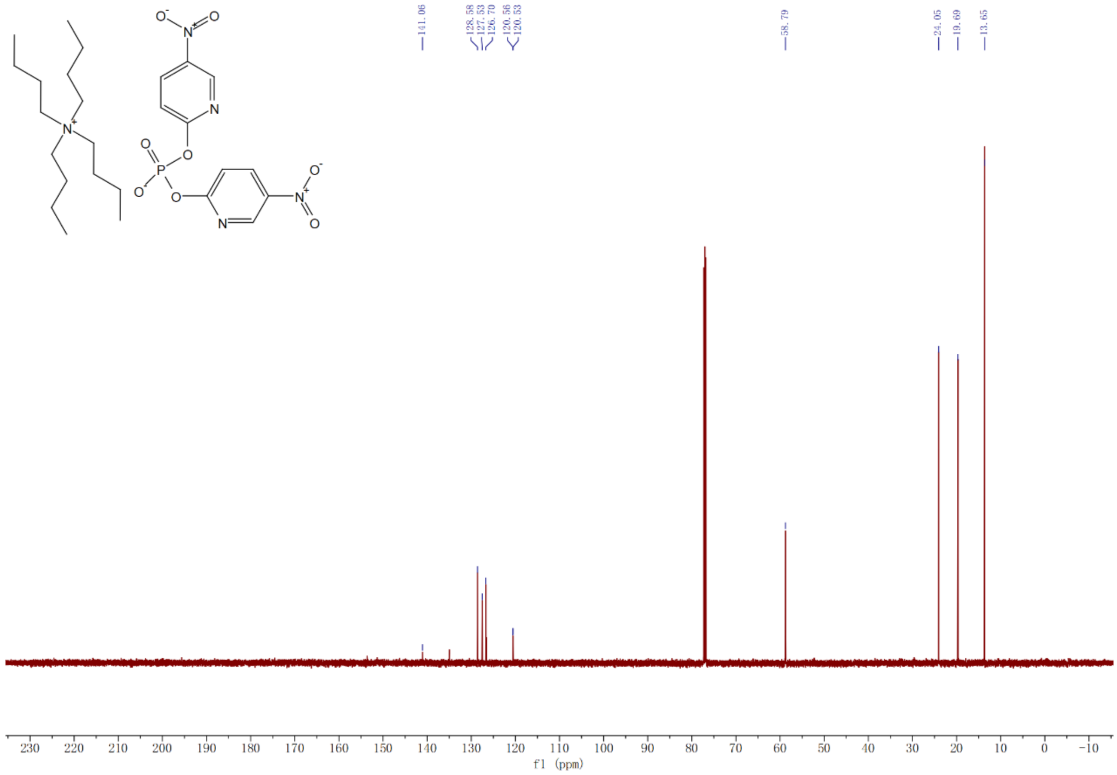

^{1}H NMR (400 MHz, $CDCl_3$)

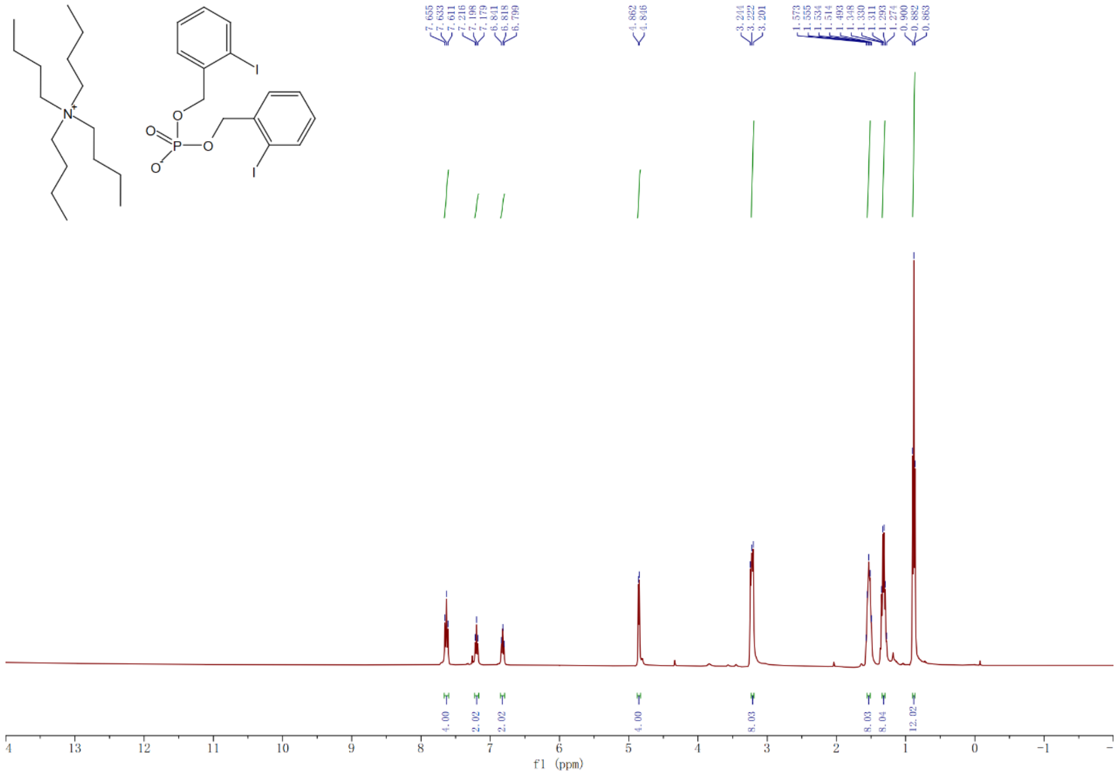

^{31}P NMR (400 MHz, $CDCl_3$)

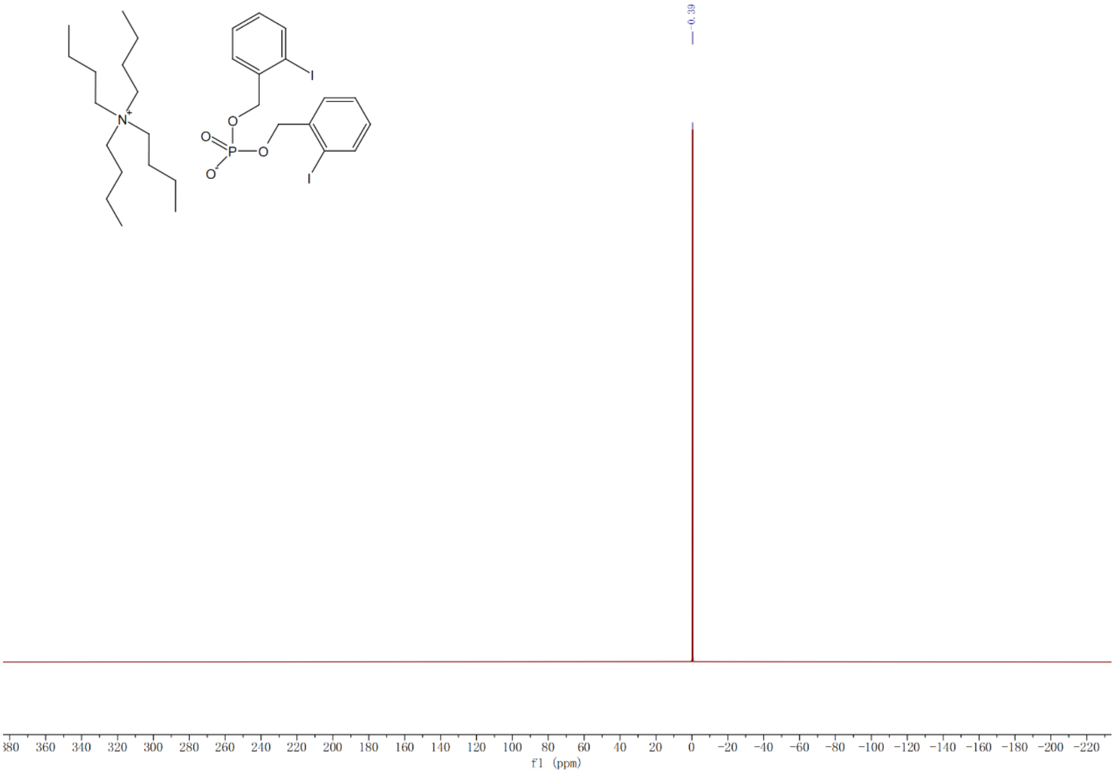

^{13}C NMR (600 MHz, $CDCl_3$)

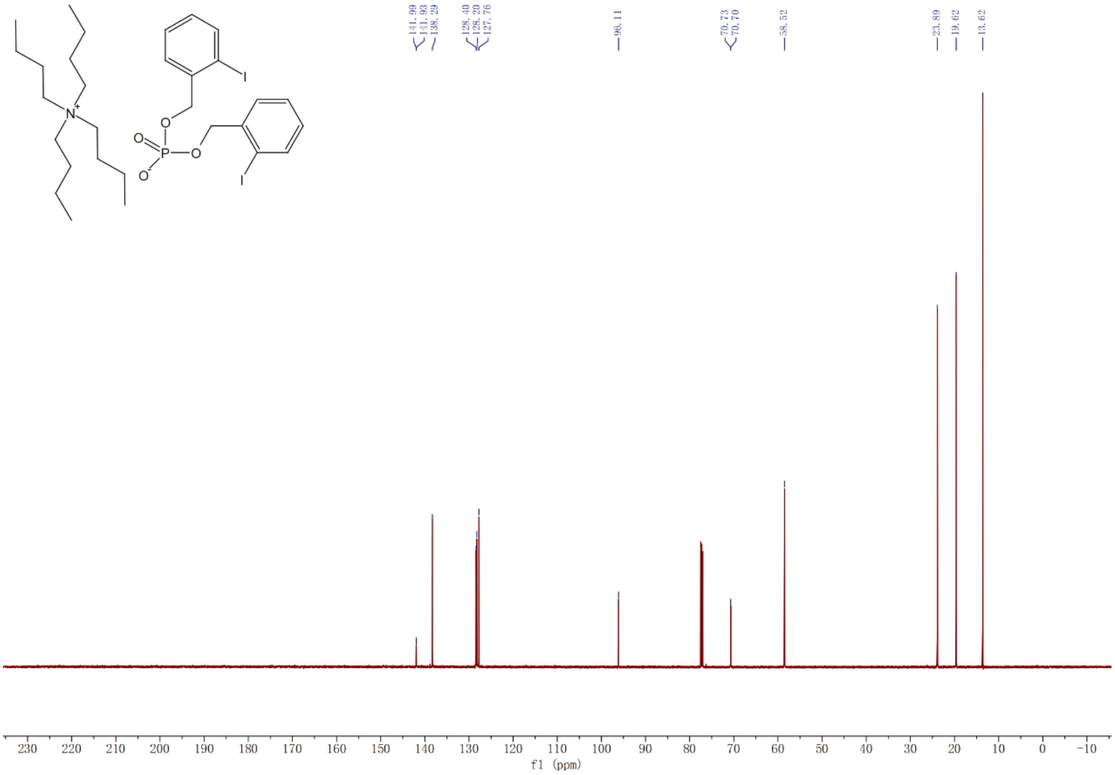

^1H NMR (400 MHz, $CDCl_3$)

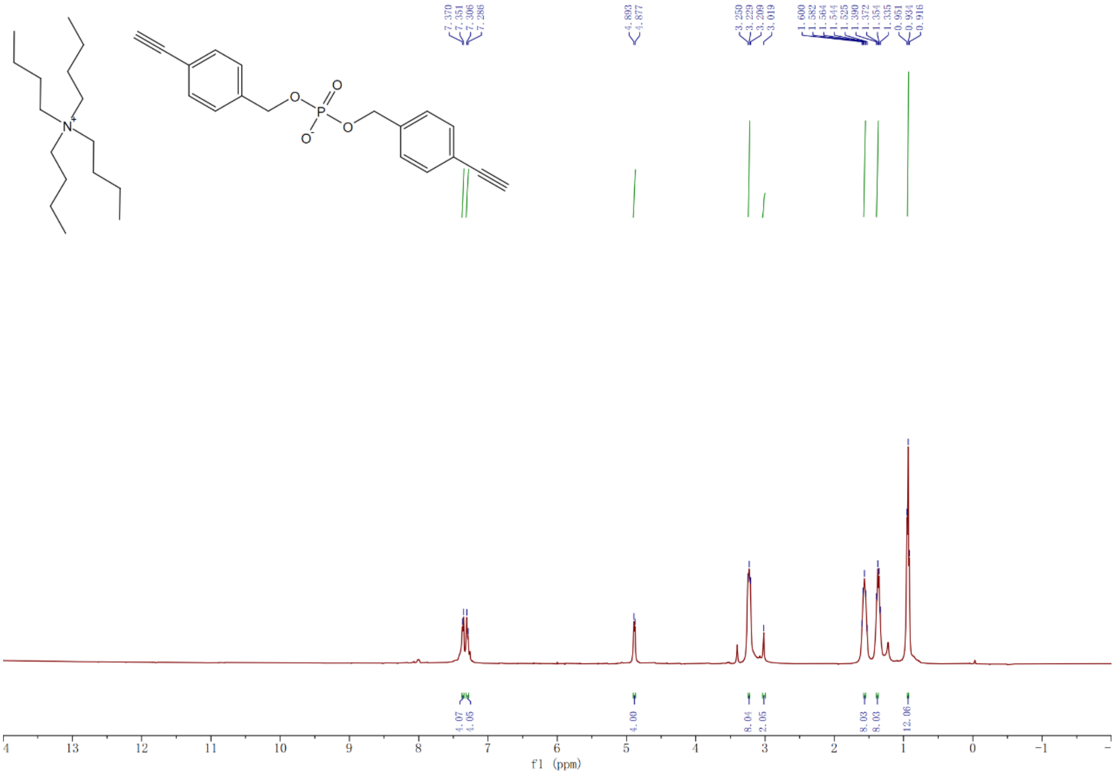

^{31}P NMR (400 MHz, $CDCl_3$)

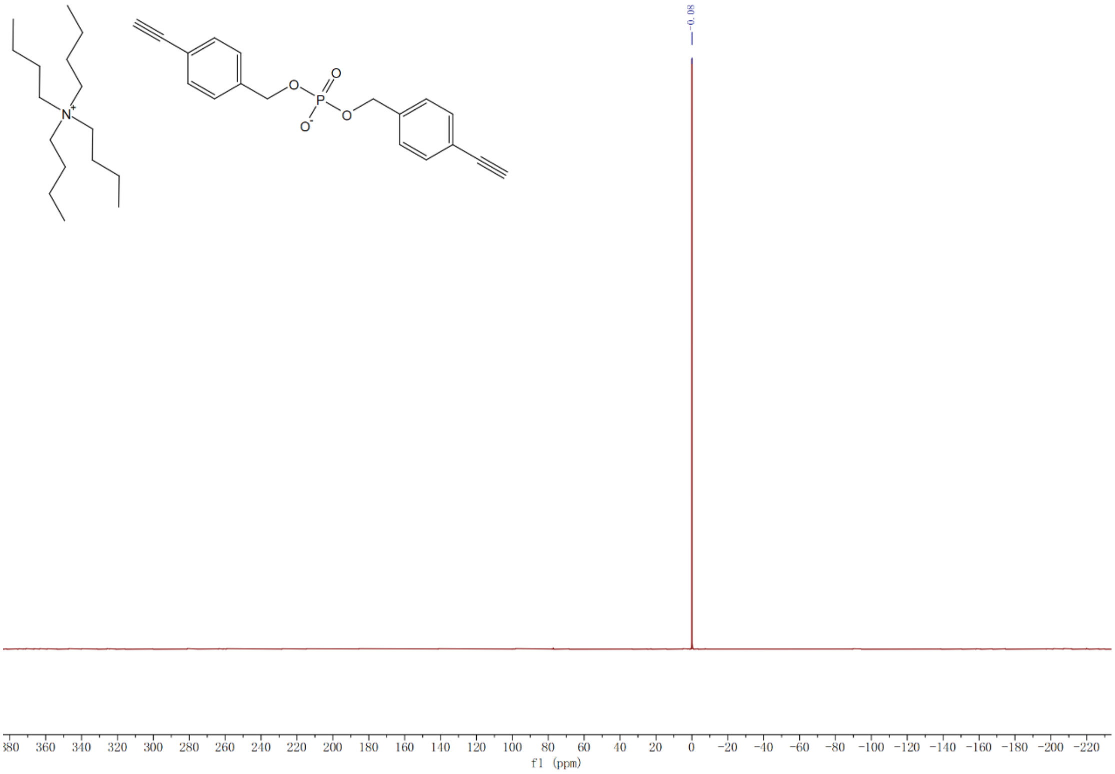

^{13}C NMR (600 MHz, $CDCl_3$)

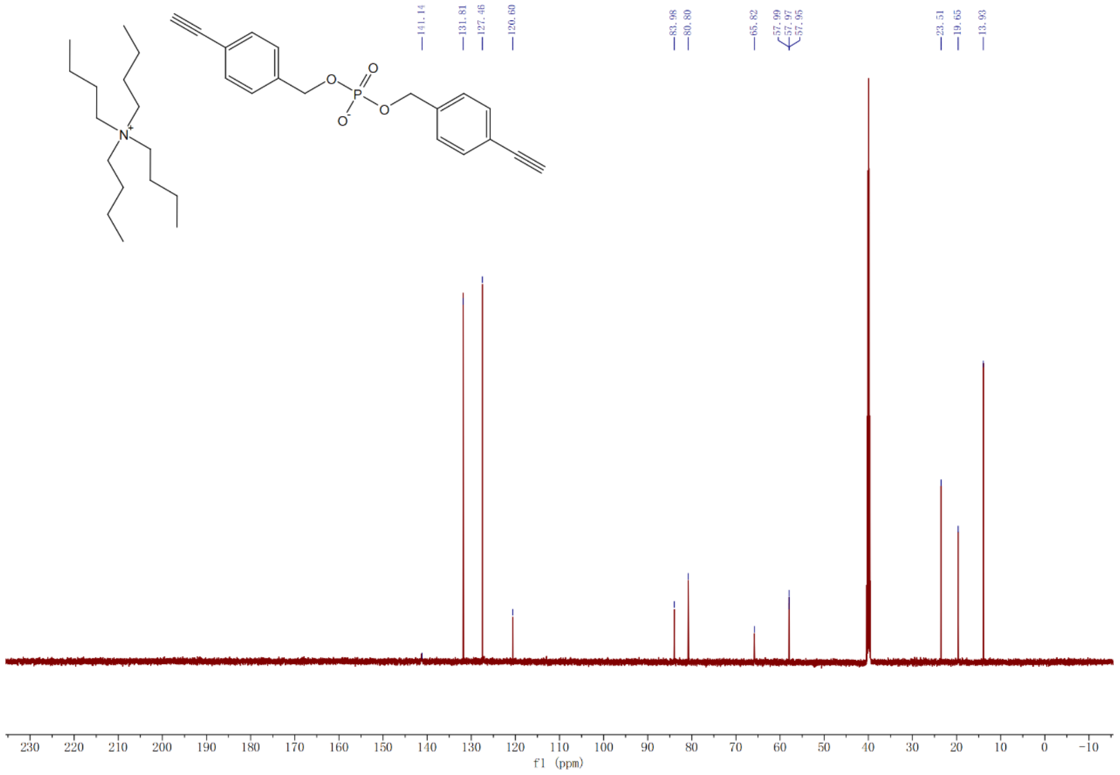

^{1}H NMR (400 MHz, $CDCl_3$)

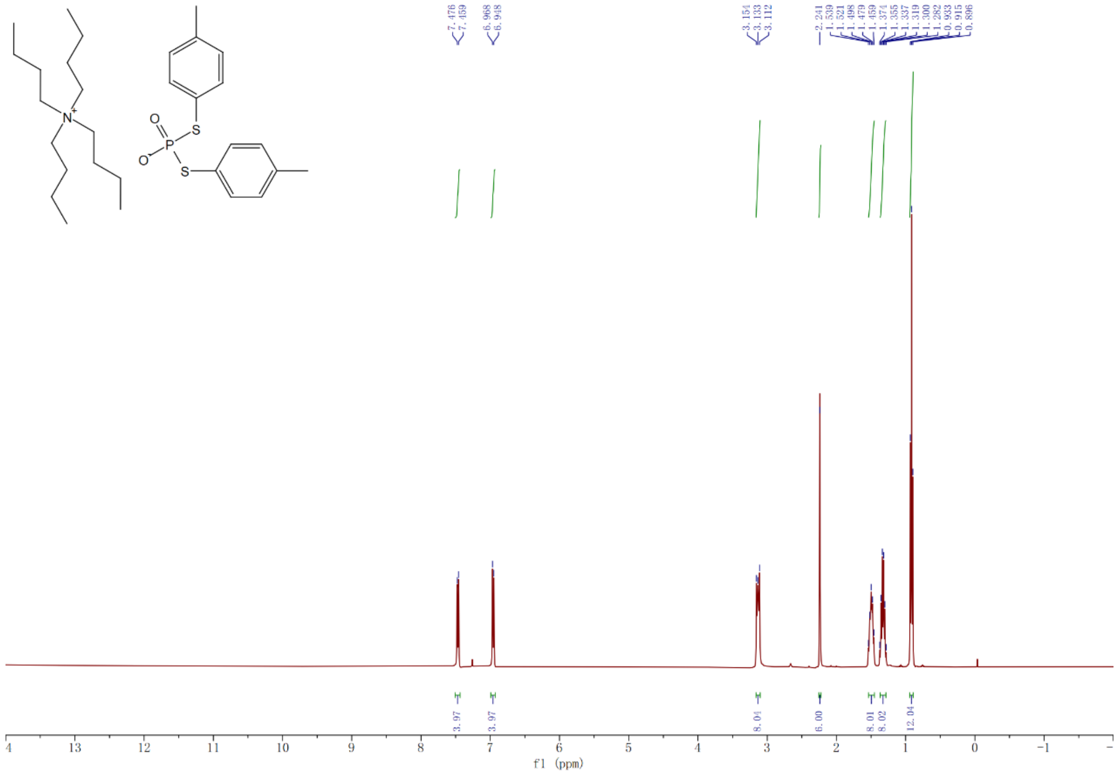

^{31}P NMR (400 MHz, CDCl$_3$)

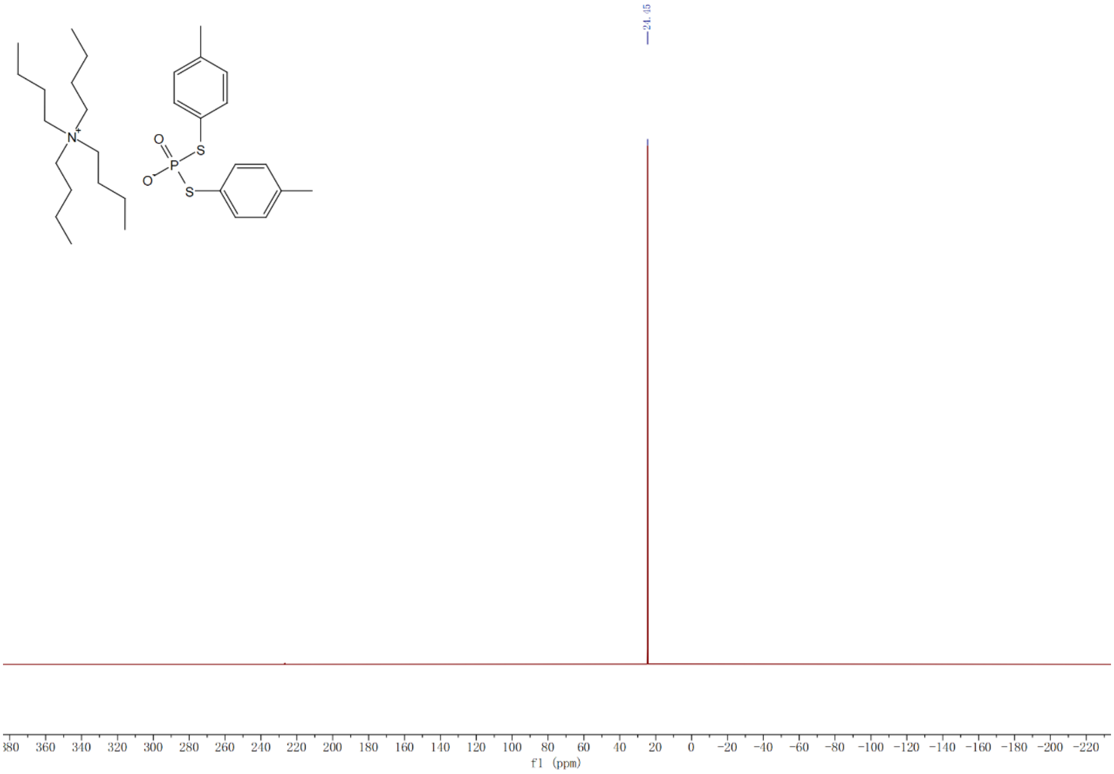

^{13}C NMR (600 MHz, CDCl$_3$)

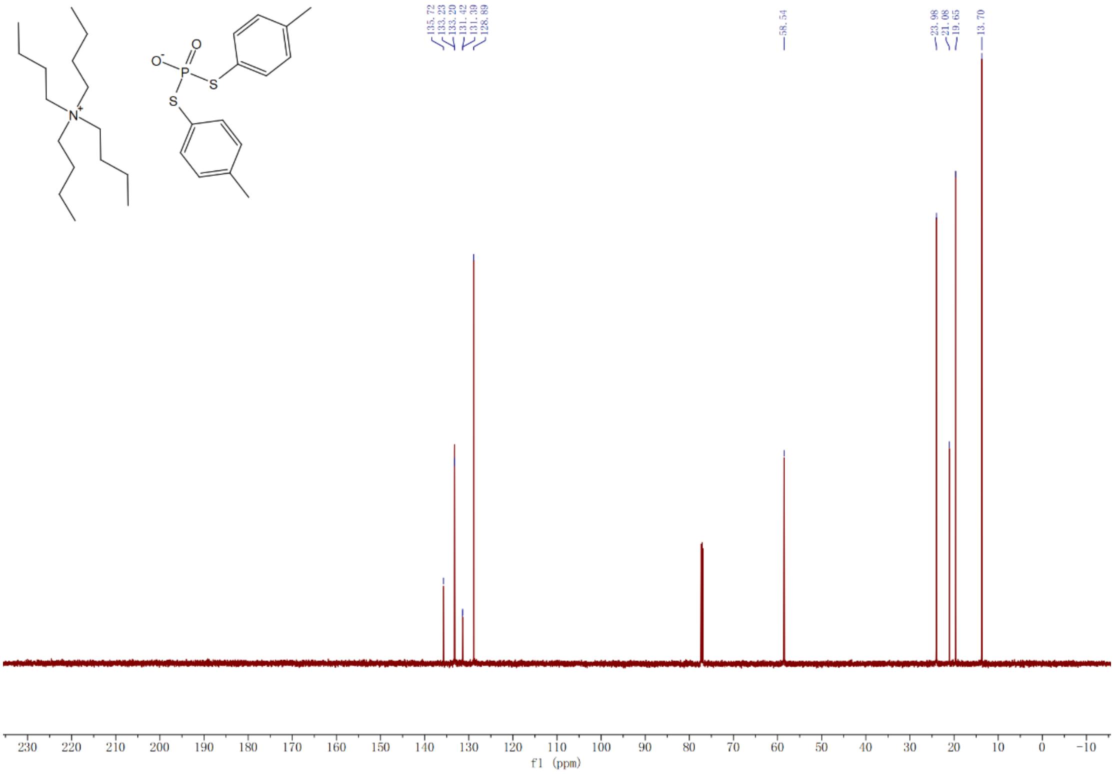

^{1}H NMR (400 MHz, DMSO-D$_6$)

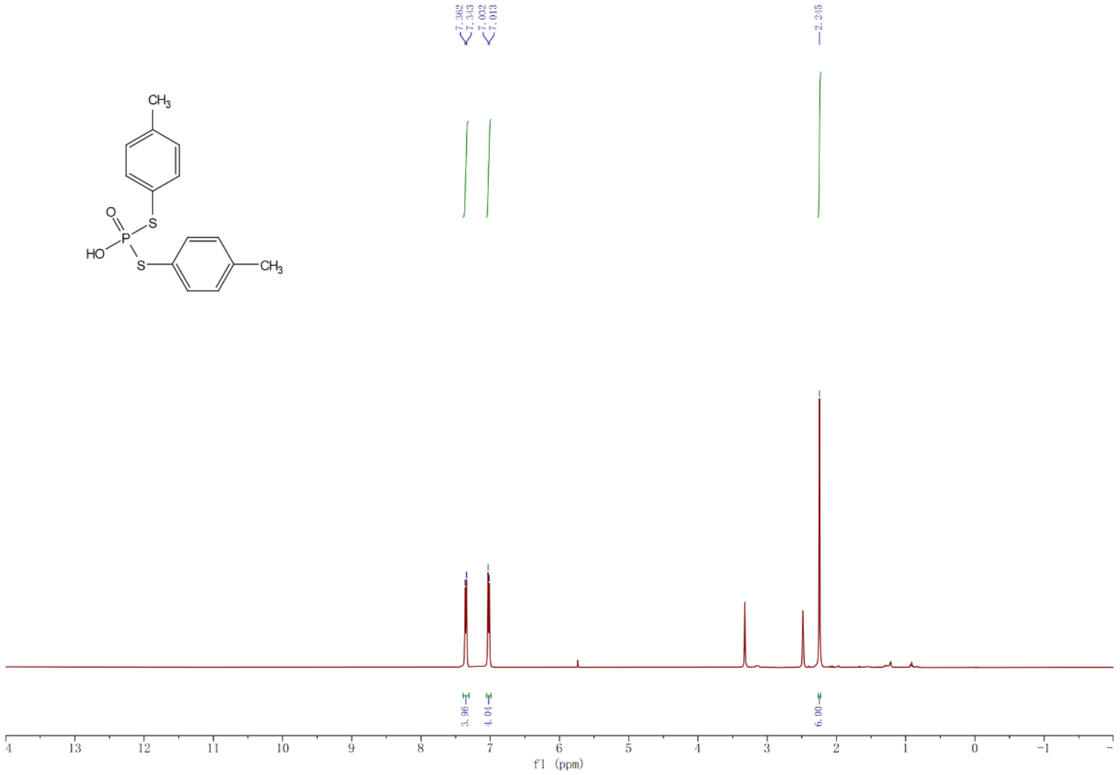

^{31}P NMR (400 MHz, DMSO-D$_6$)

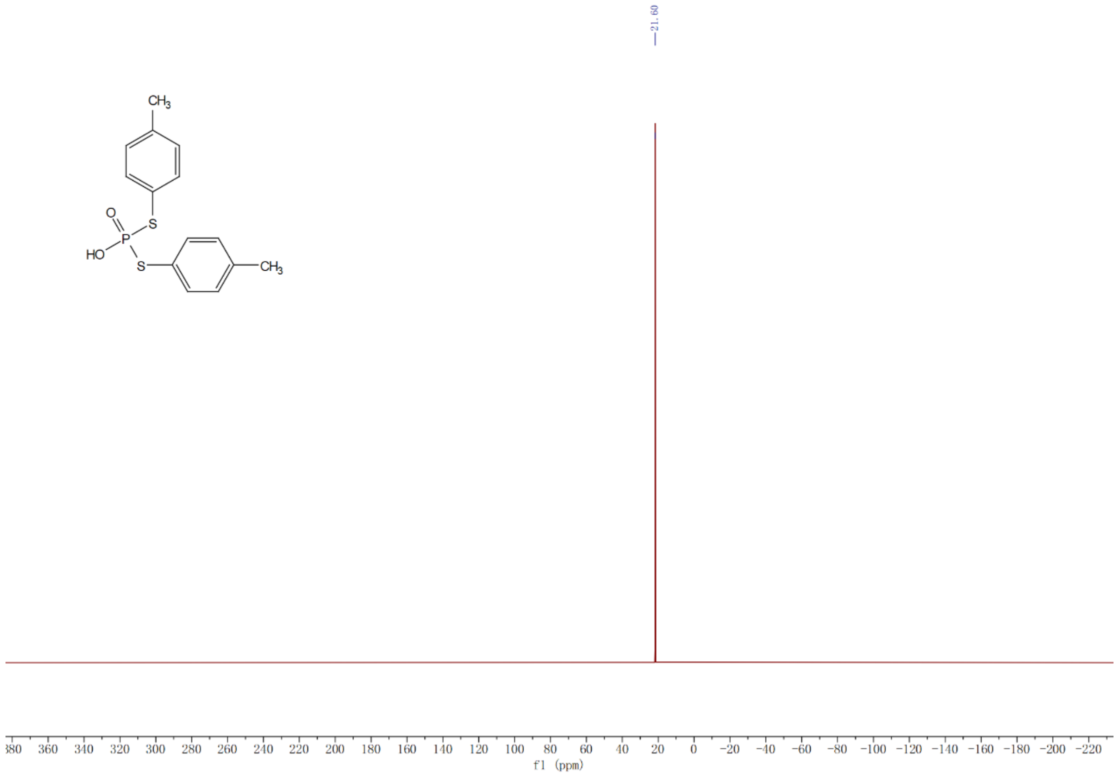

^{13}C NMR (600 MHz, DMSO-D$_6$)

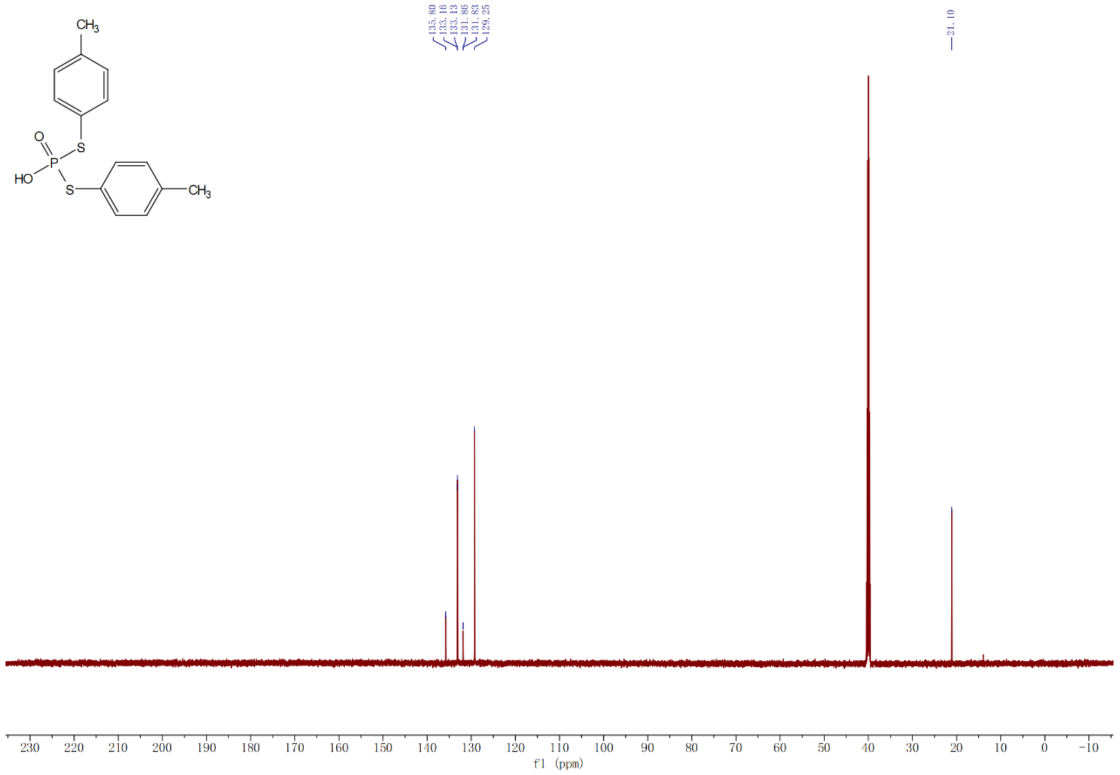

^{1}H NMR (400 MHz, CDCl$_3$)

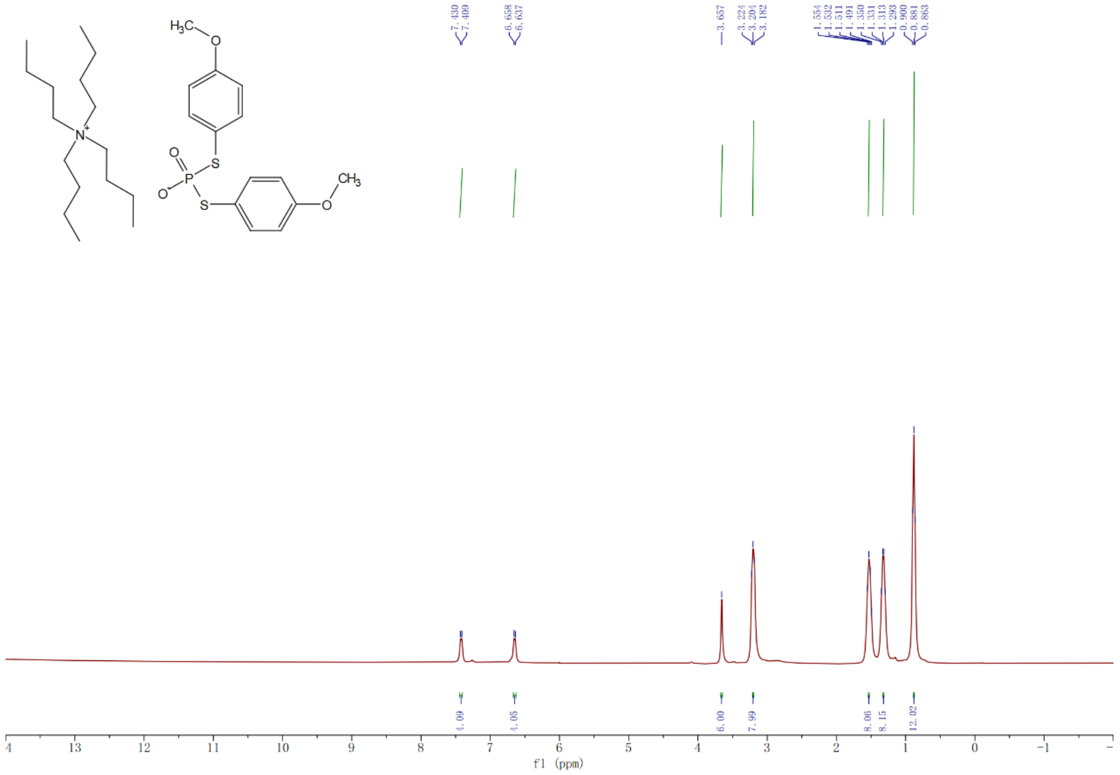

^{31}P NMR (400 MHz, $CDCl_3$)

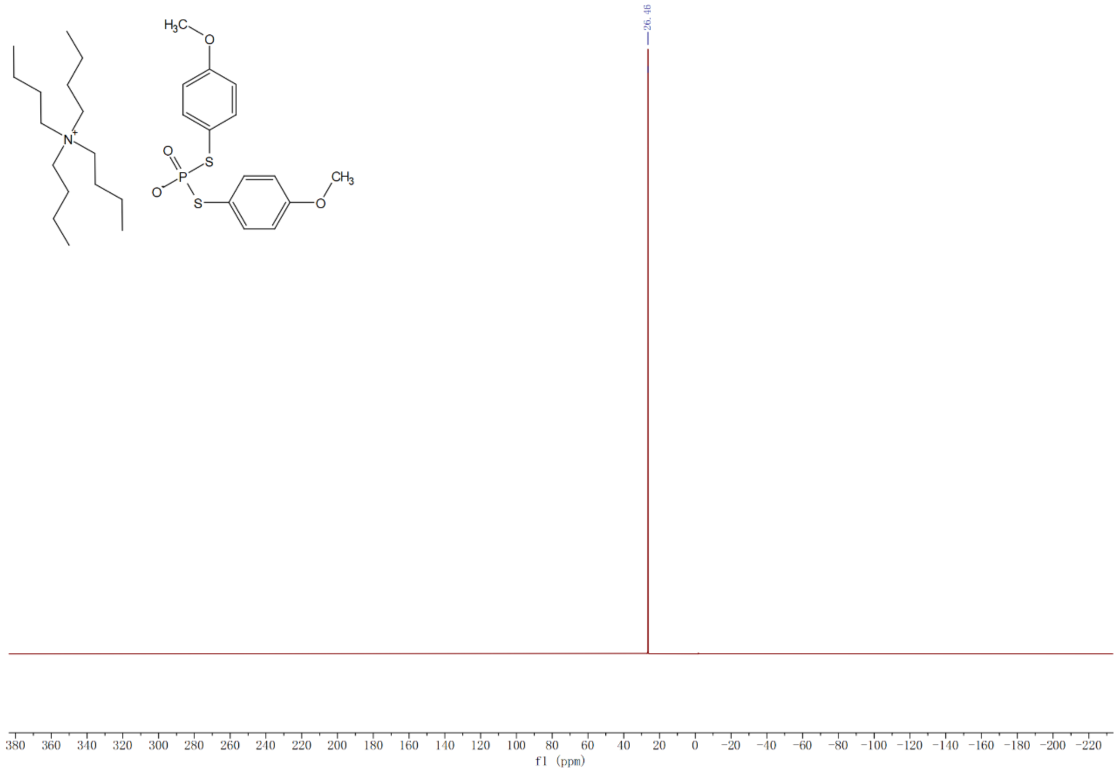

^{13}C NMR (600 MHz, $CDCl_3$)

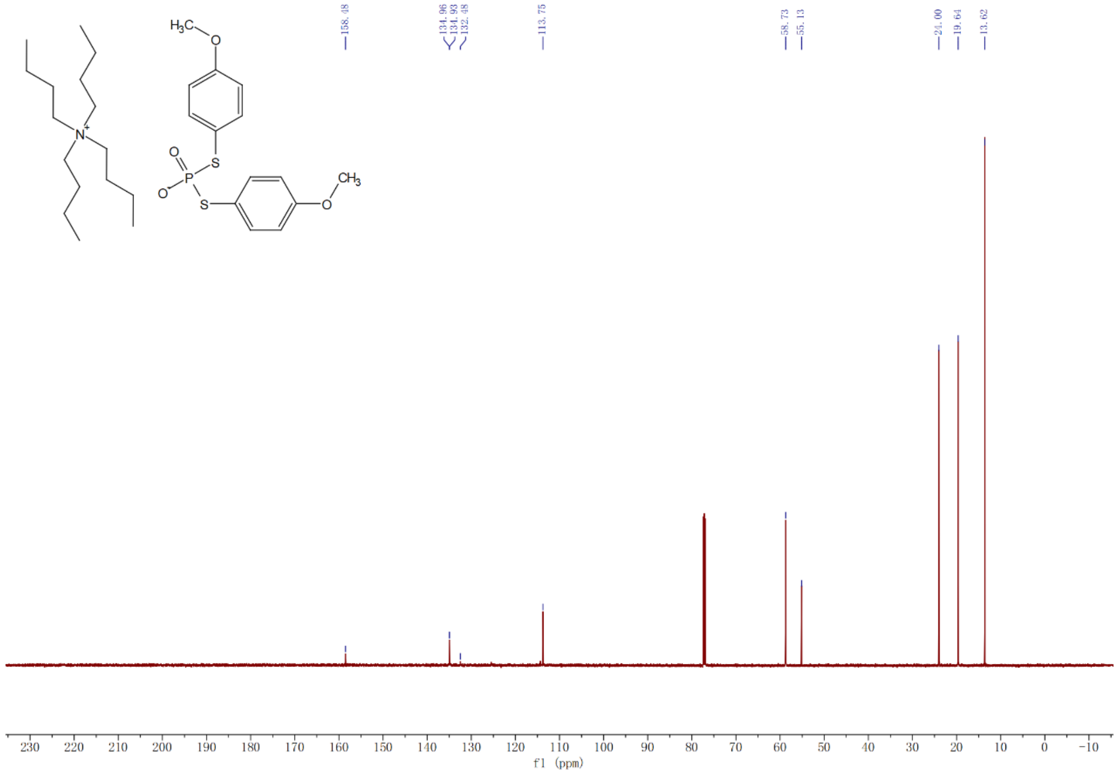

^{1}H NMR (400 MHz, CDCl3)

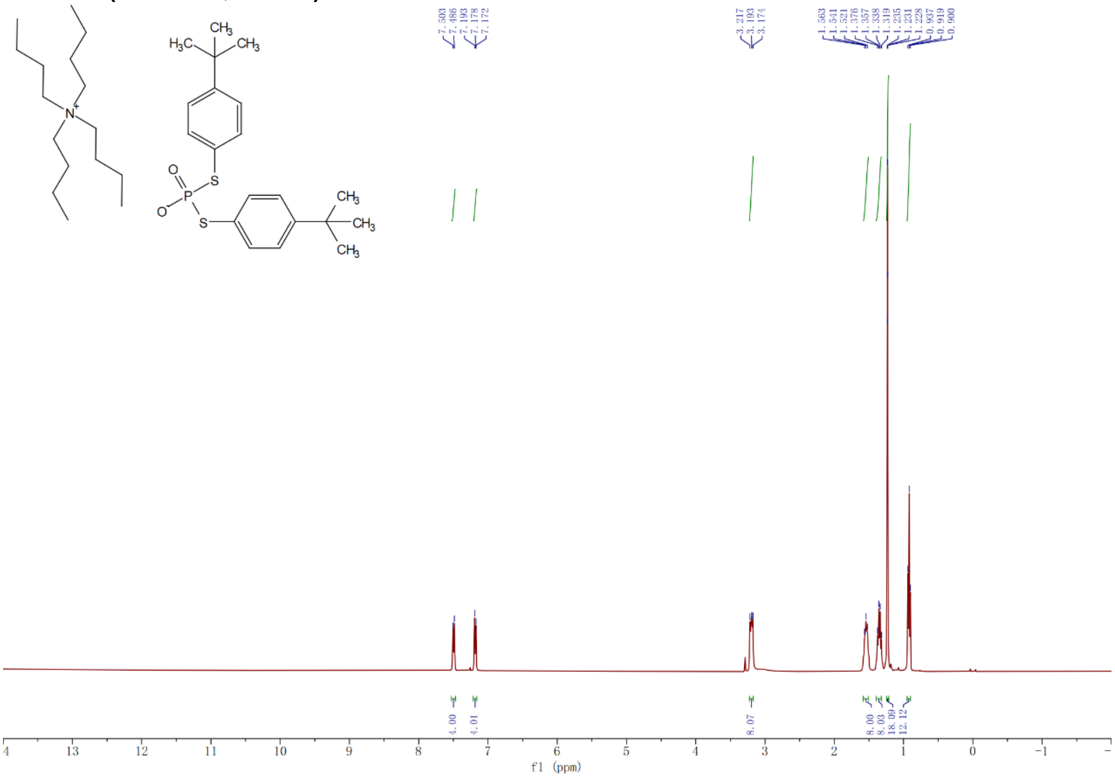

^{31}P NMR (400 MHz, CDCl3)

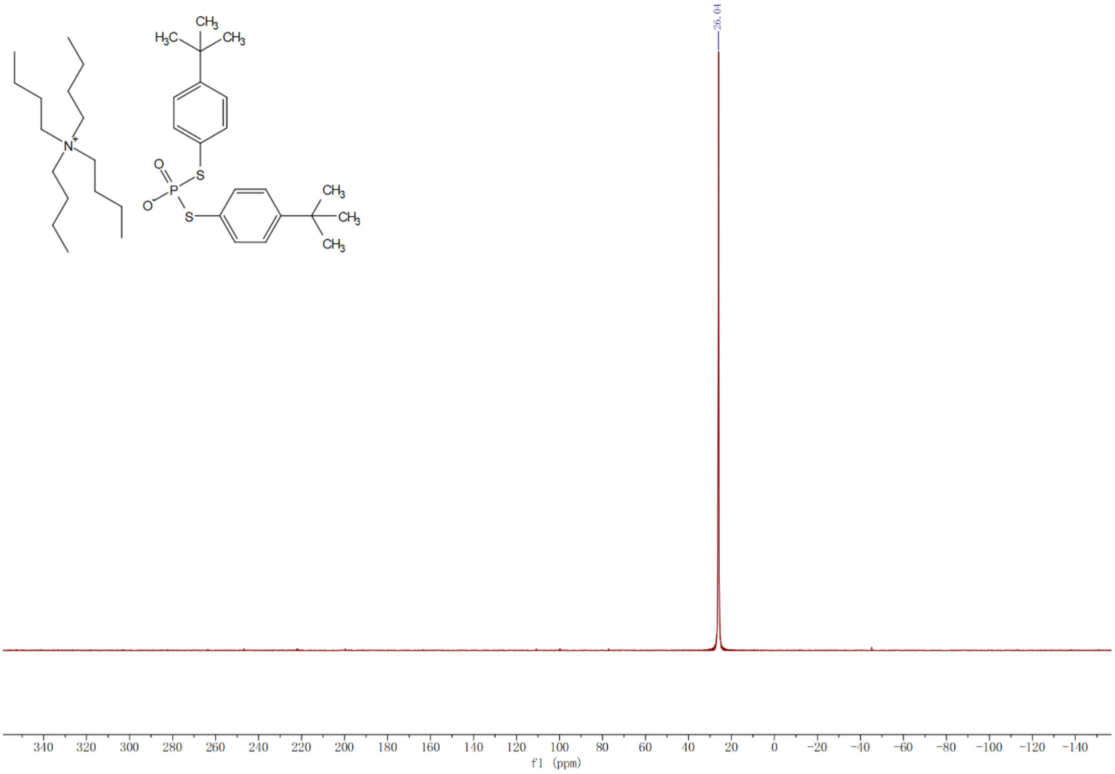

^{13}C NMR (600 MHz, CDCl$_3$)

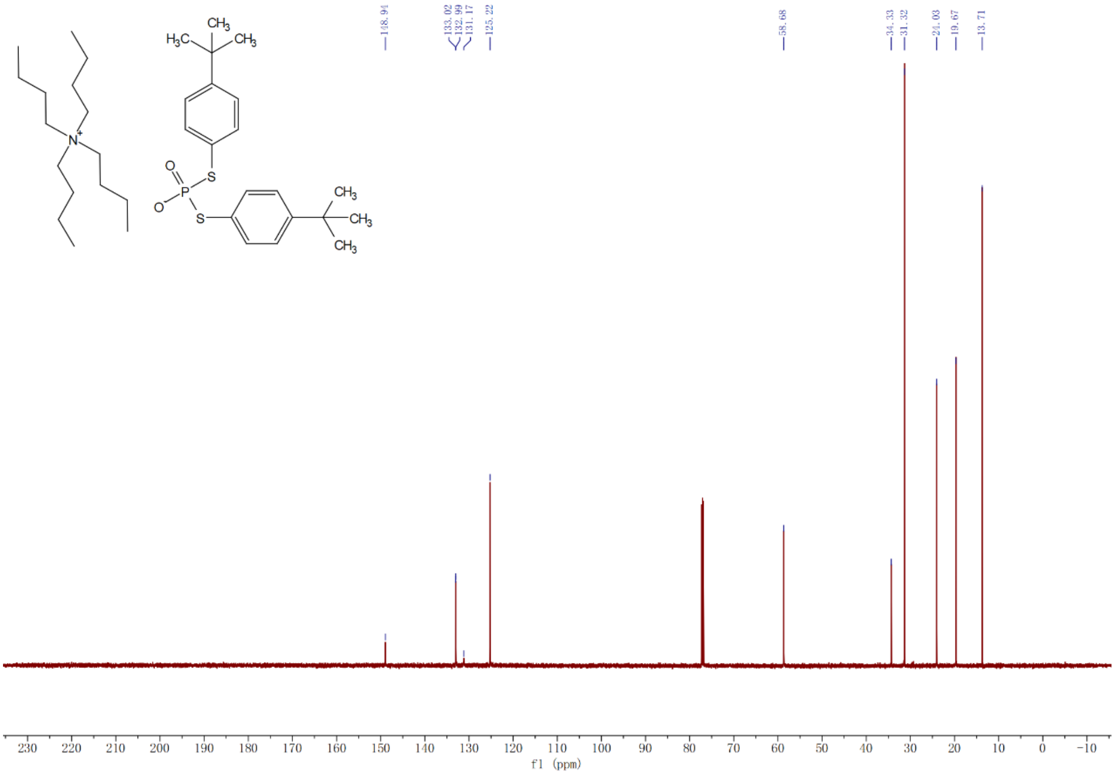

^{1}H NMR (400 MHz, DMSO-D$_6$)

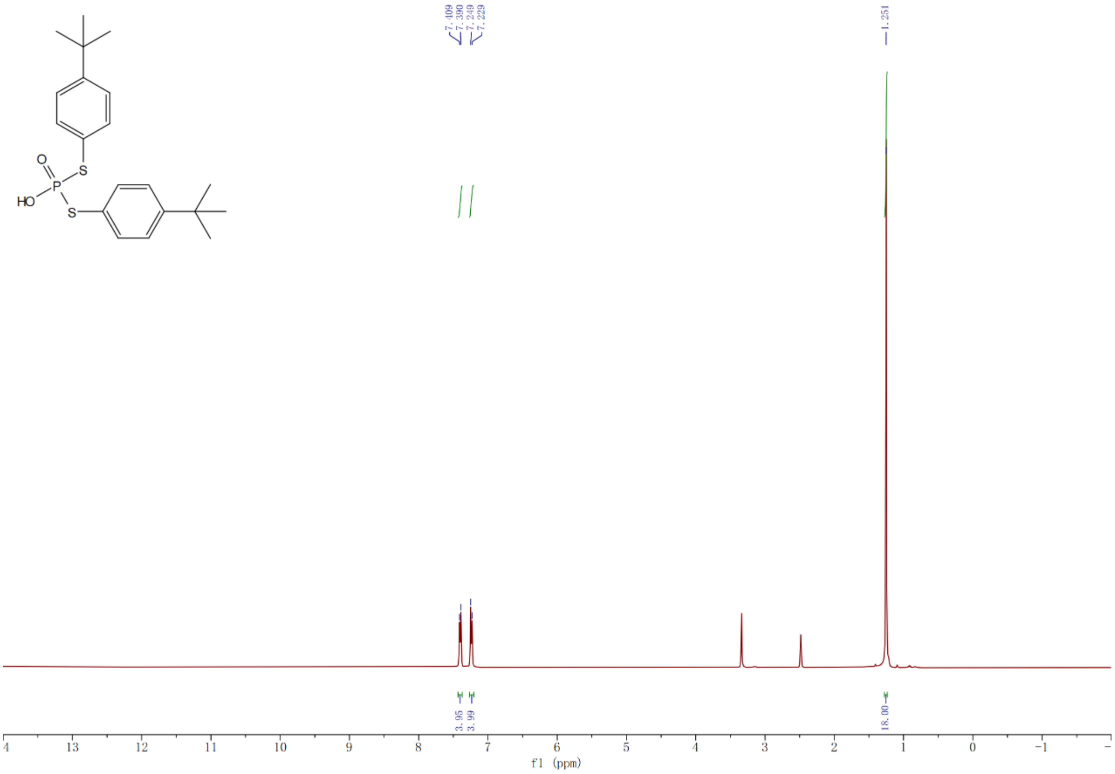

^{31}P NMR (400 MHz, DMSO-D$_6$))

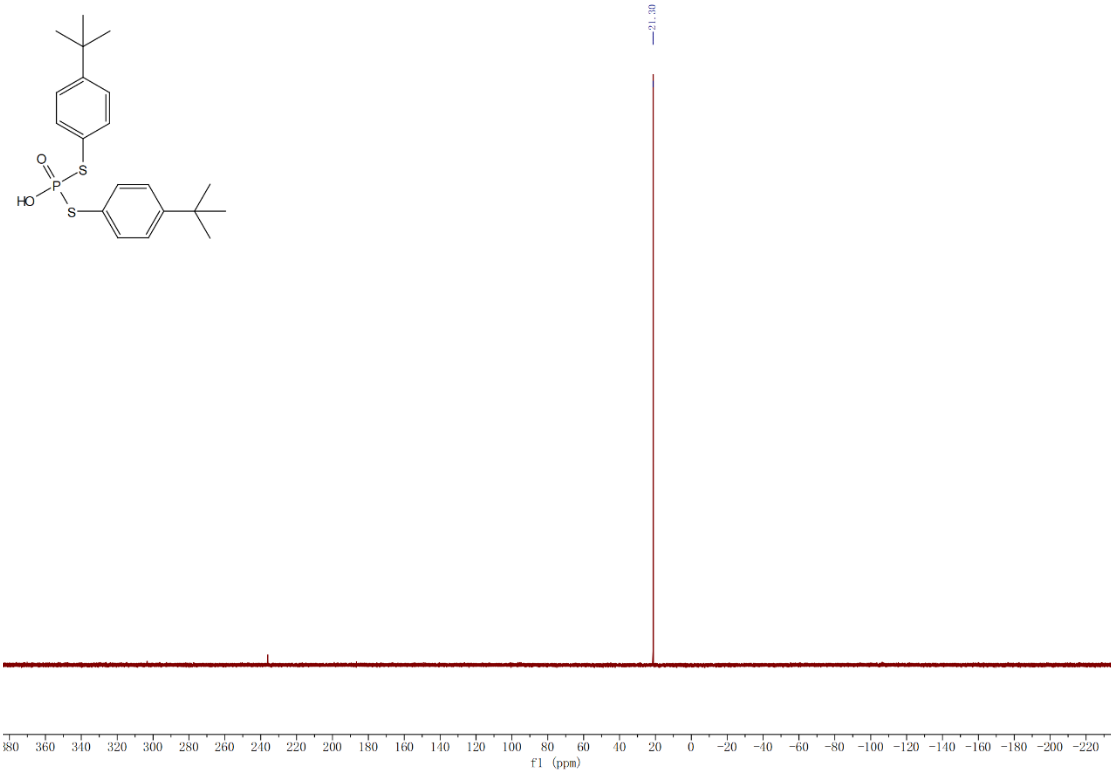

^{13}C NMR (600 MHz, DMSO-D$_6$)

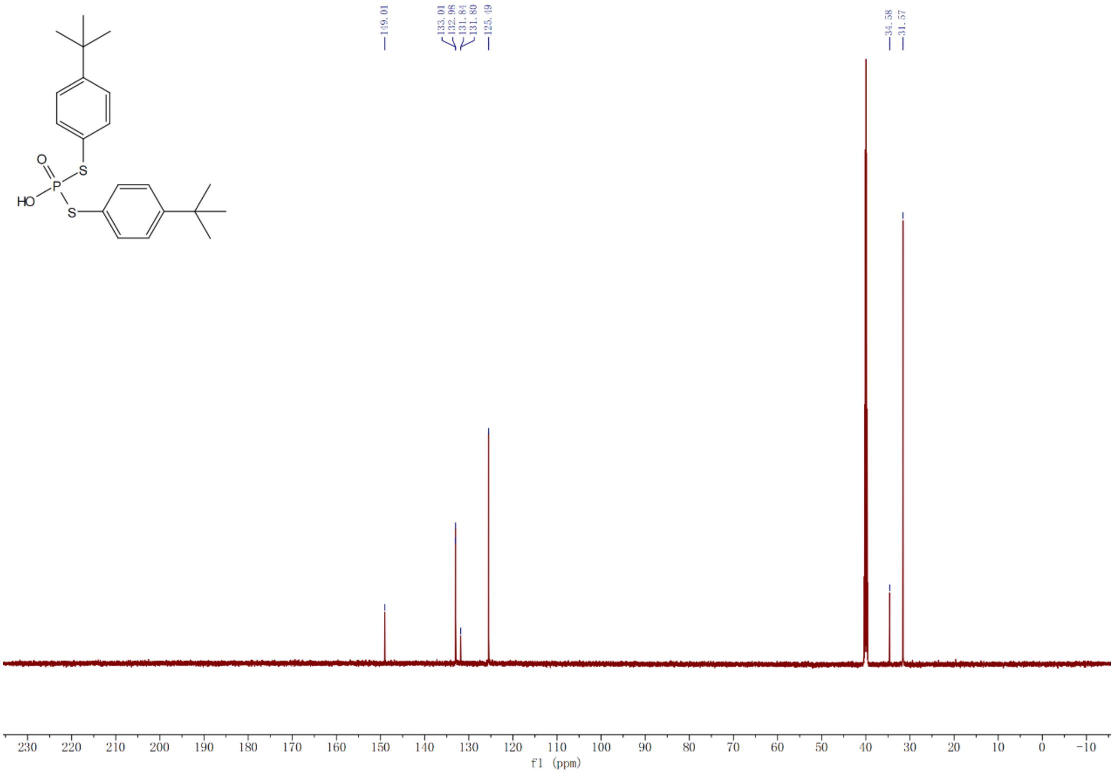

^{1}H NMR (400 MHz, $CDCl_3$)

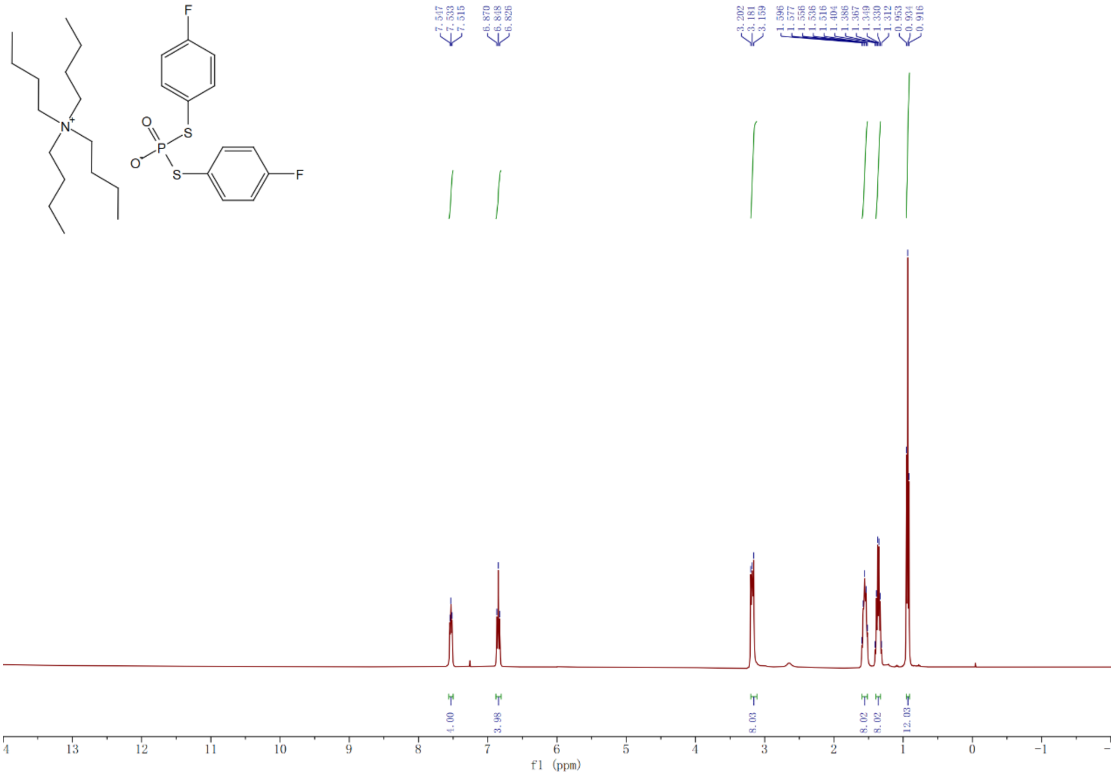

^{31}P NMR (400 MHz, $CDCl_3$)

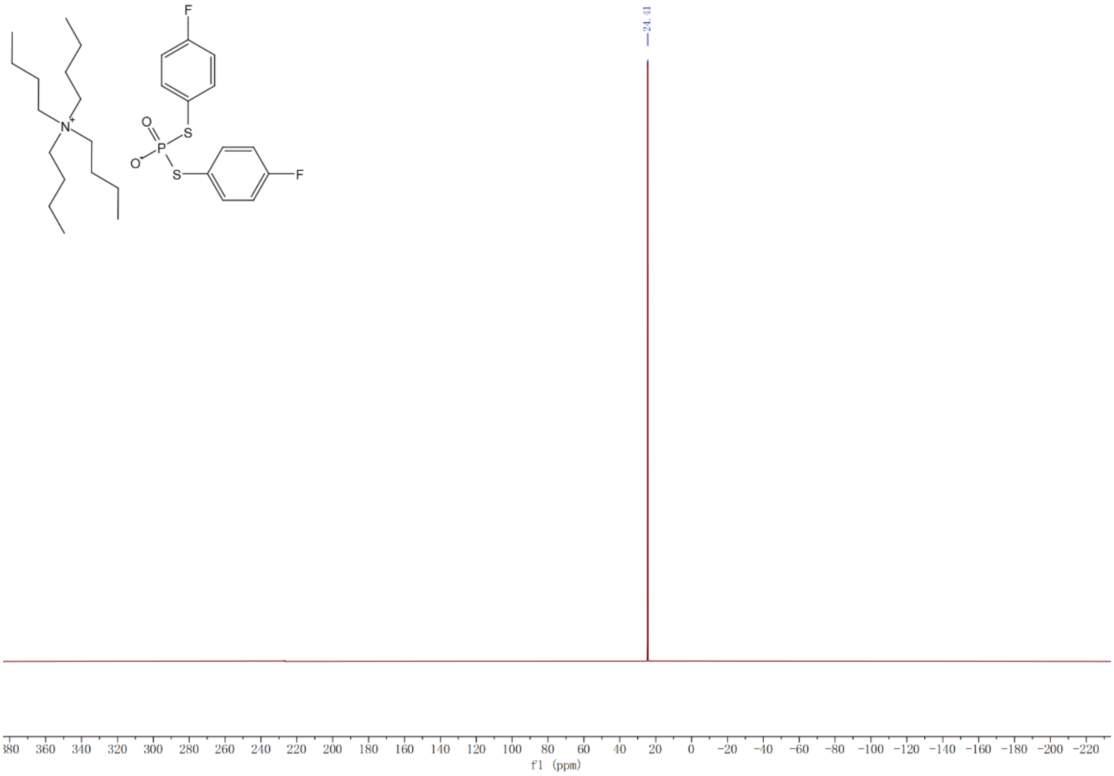

^{13}C NMR (600 MHz, $CDCl_3$)

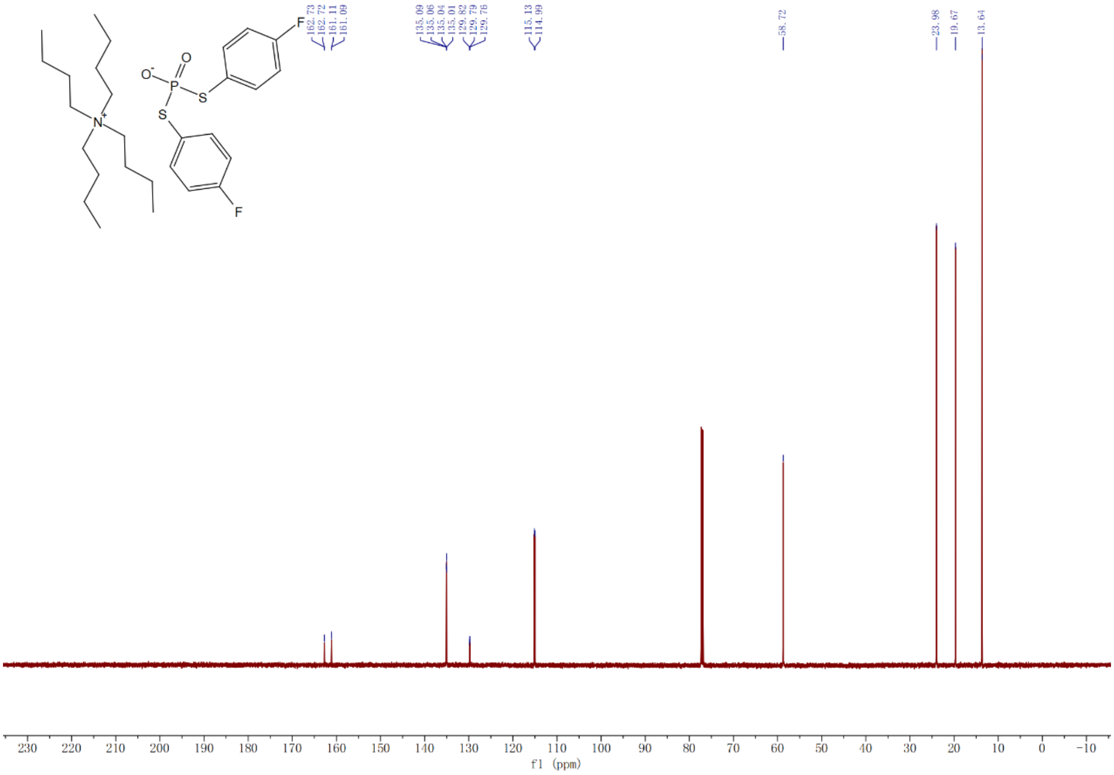

^{1}H NMR (400 MHz, DMSO-D_6)

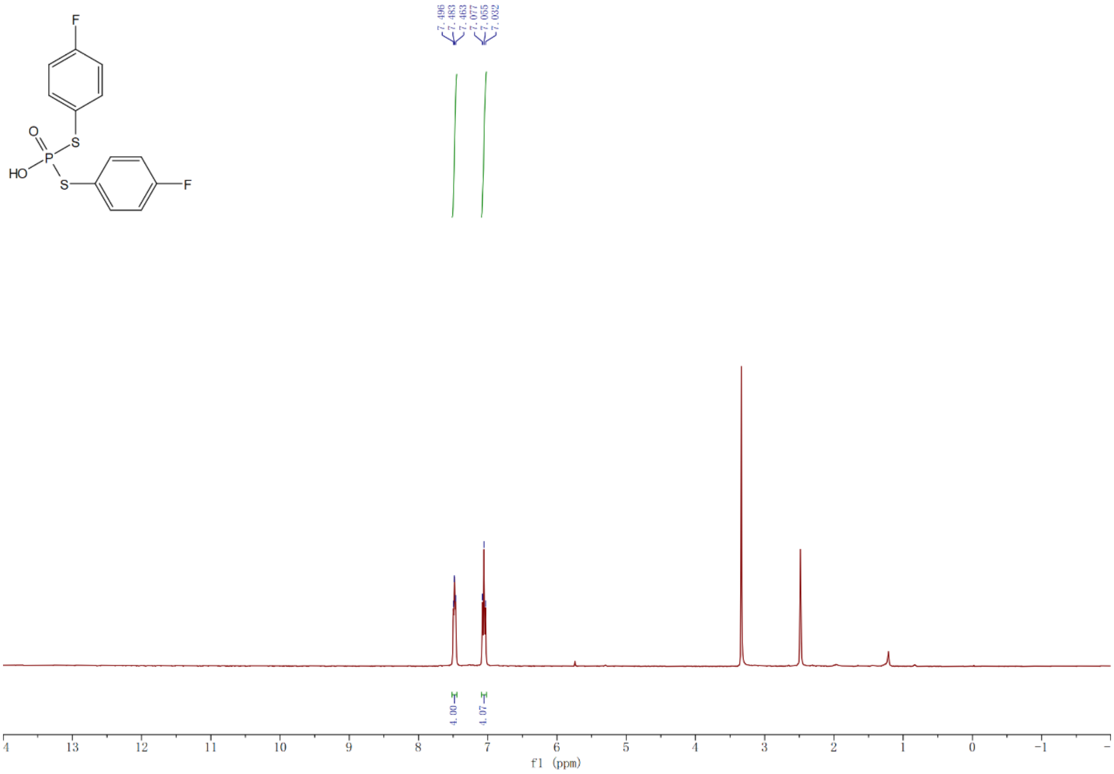

^{31}P NMR (400 MHz, DMSO-D$_6$)

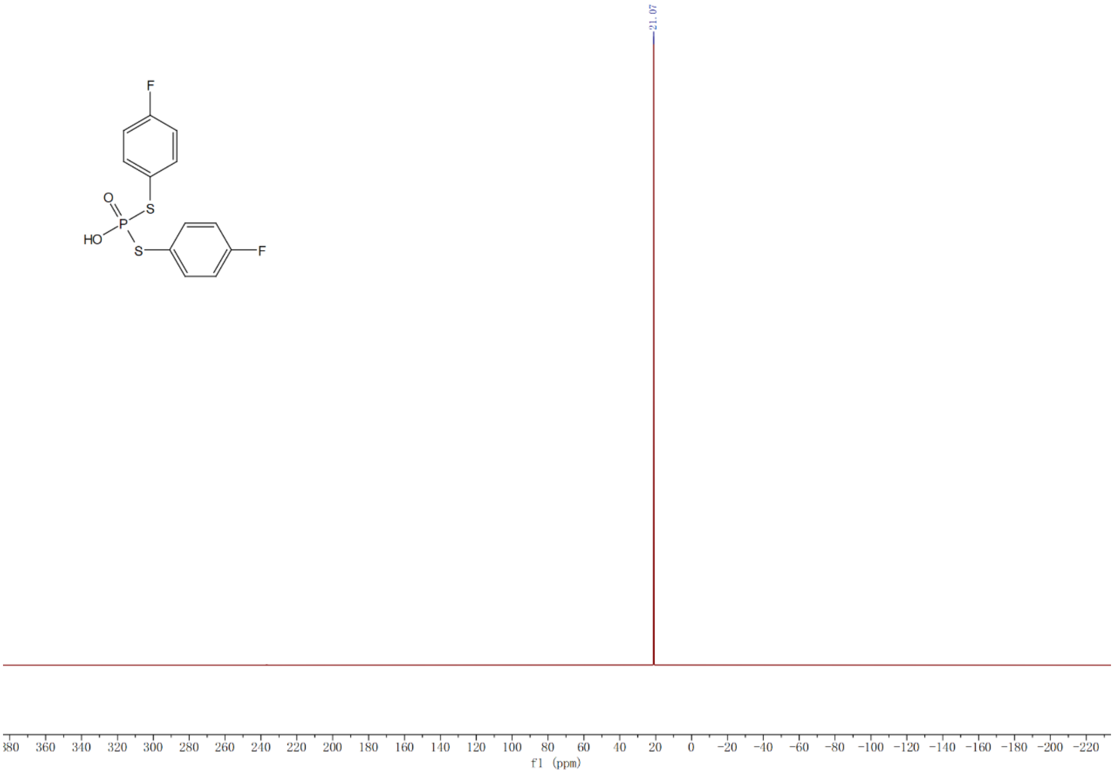

^{19}F NMR (400 MHz, DMSO-D$_6$)

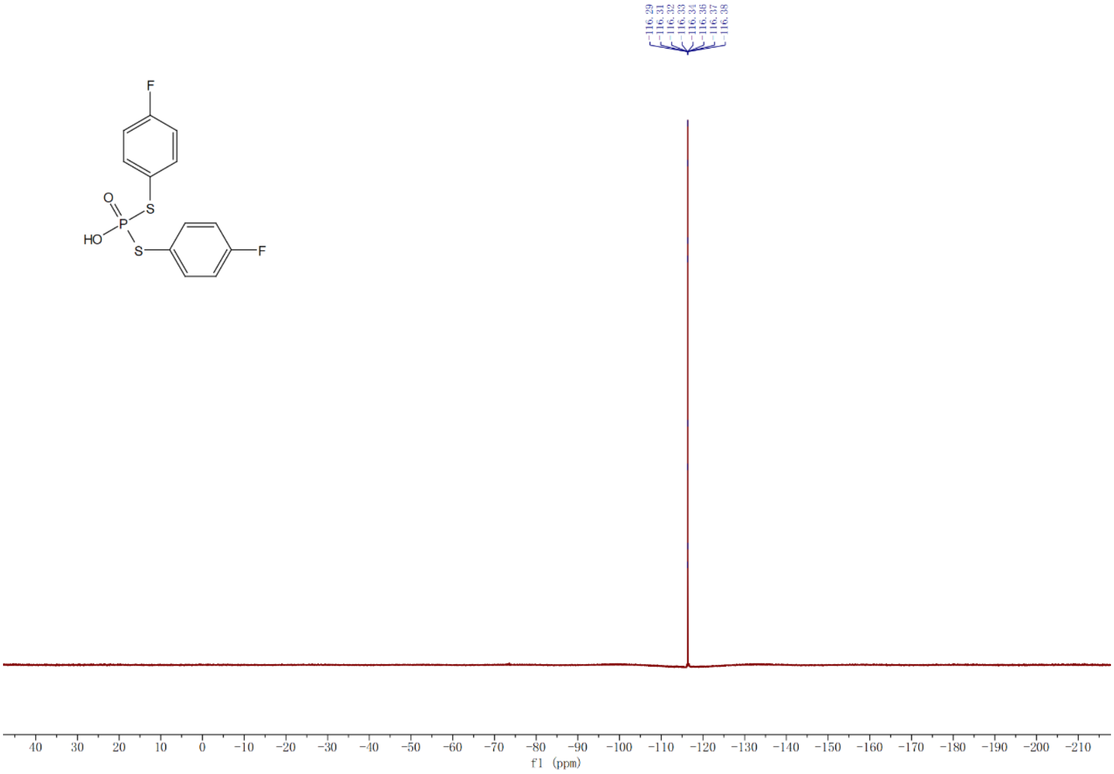

^{13}C NMR (600 MHz, DMSO-D$_6$)

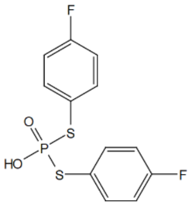

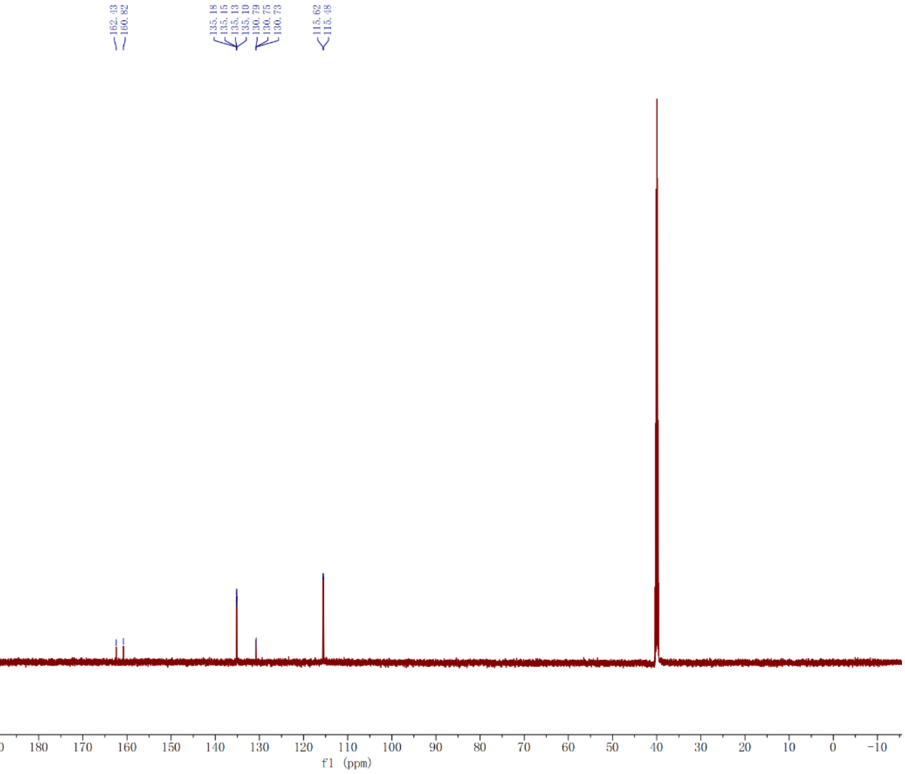

^1H NMR (400 MHz, CDCl$_3$)

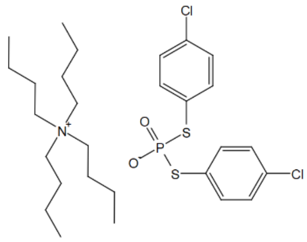

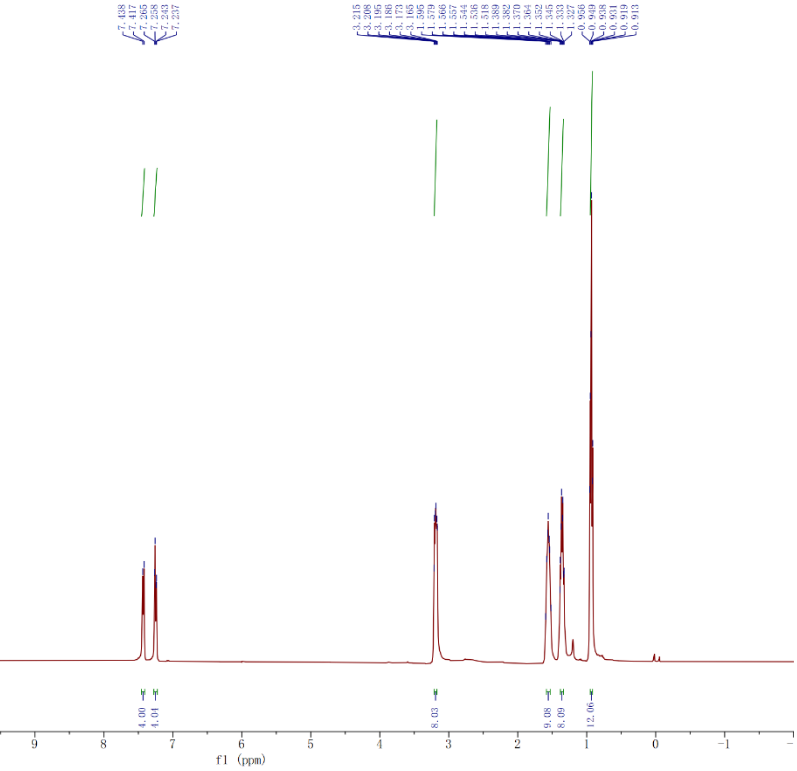

^{31}P NMR (400 MHz, $CDCl_3$)

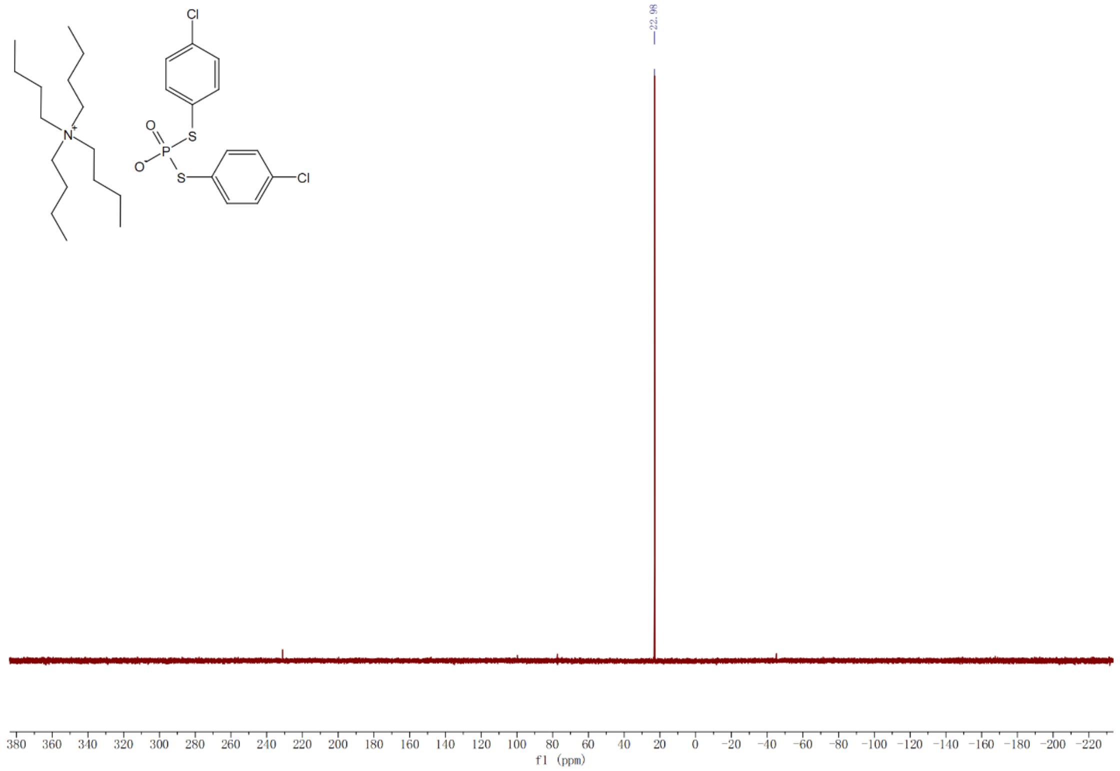

^{13}C NMR (600 MHz, $CDCl_3$)

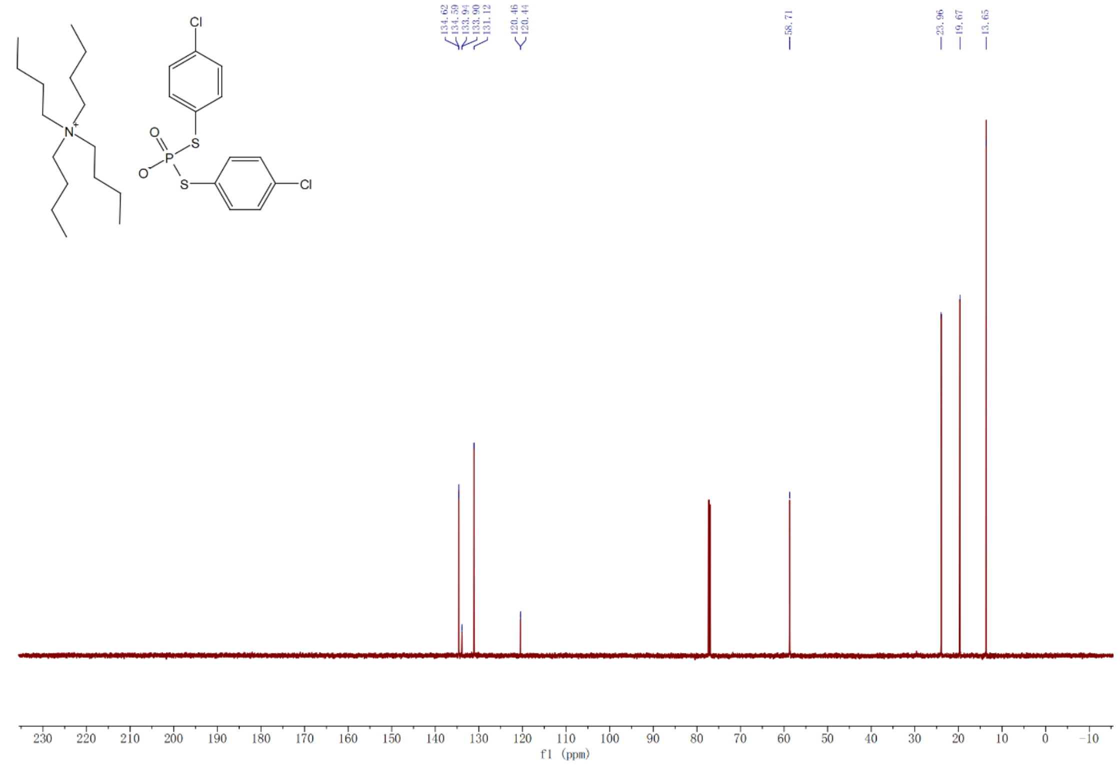

^{1}H NMR (400 MHz, $CDCl_3$)

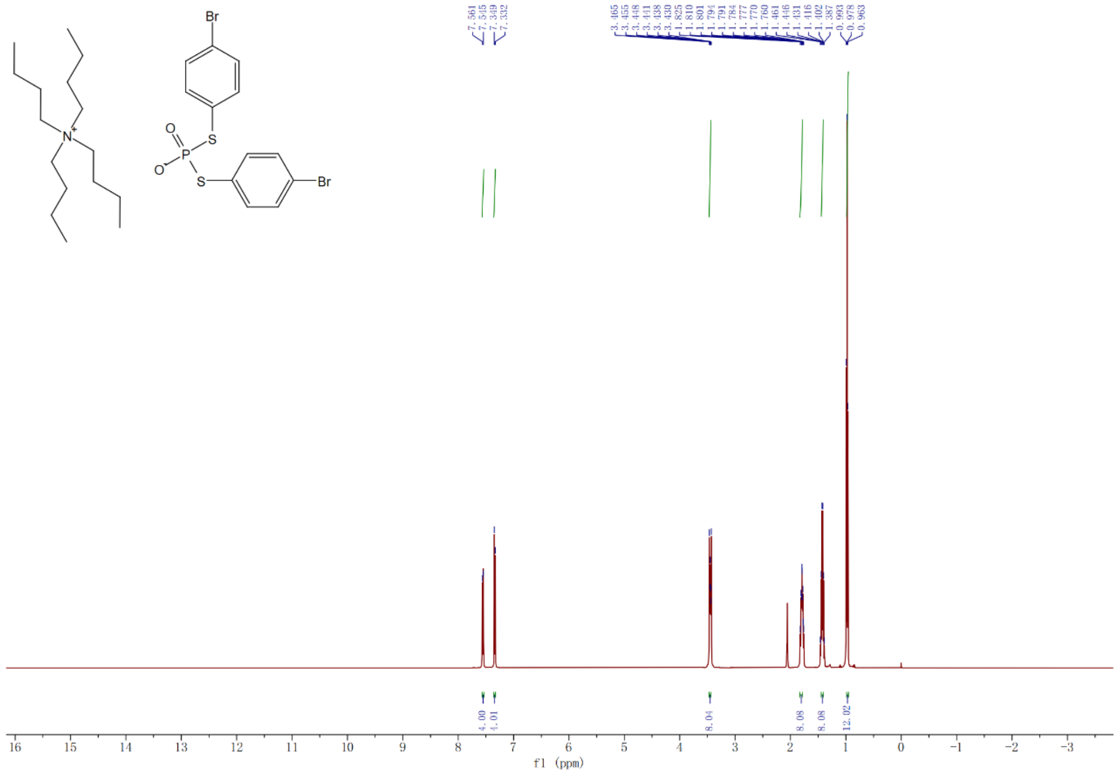

^{31}P NMR (400 MHz, $CDCl_3$)

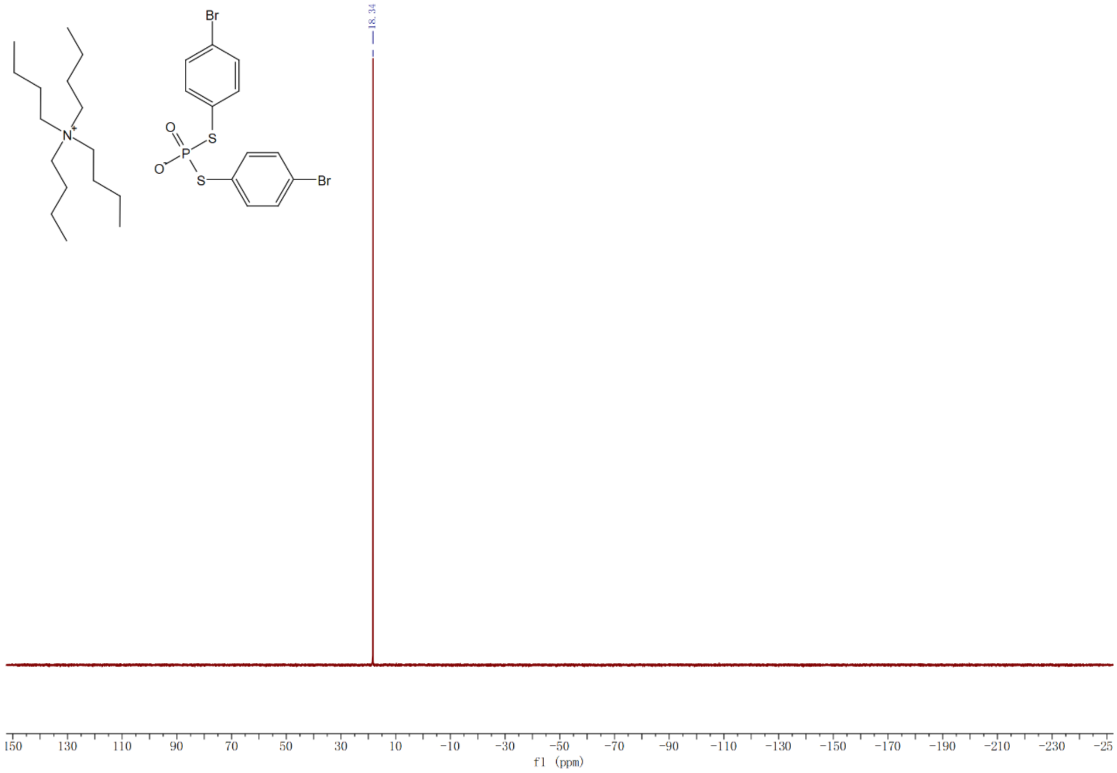

^{13}C NMR (600 MHz, $CDCl_3$)

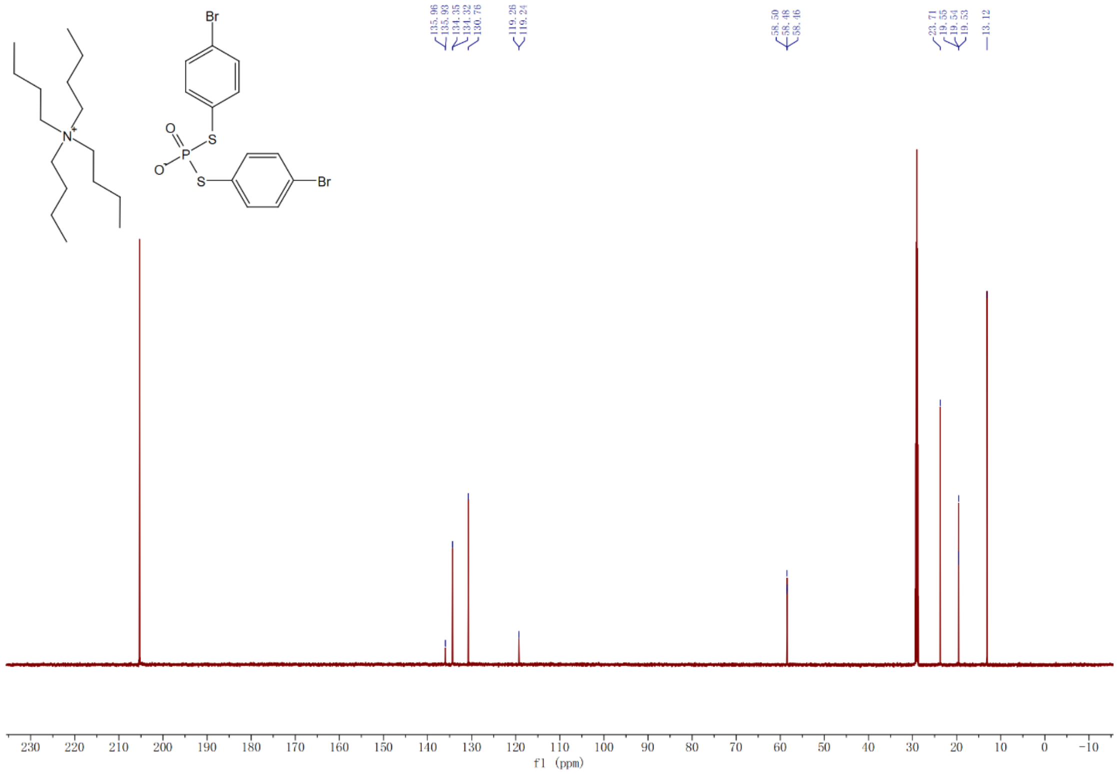

^{1}H NMR (400 MHz, $CDCl_3$)

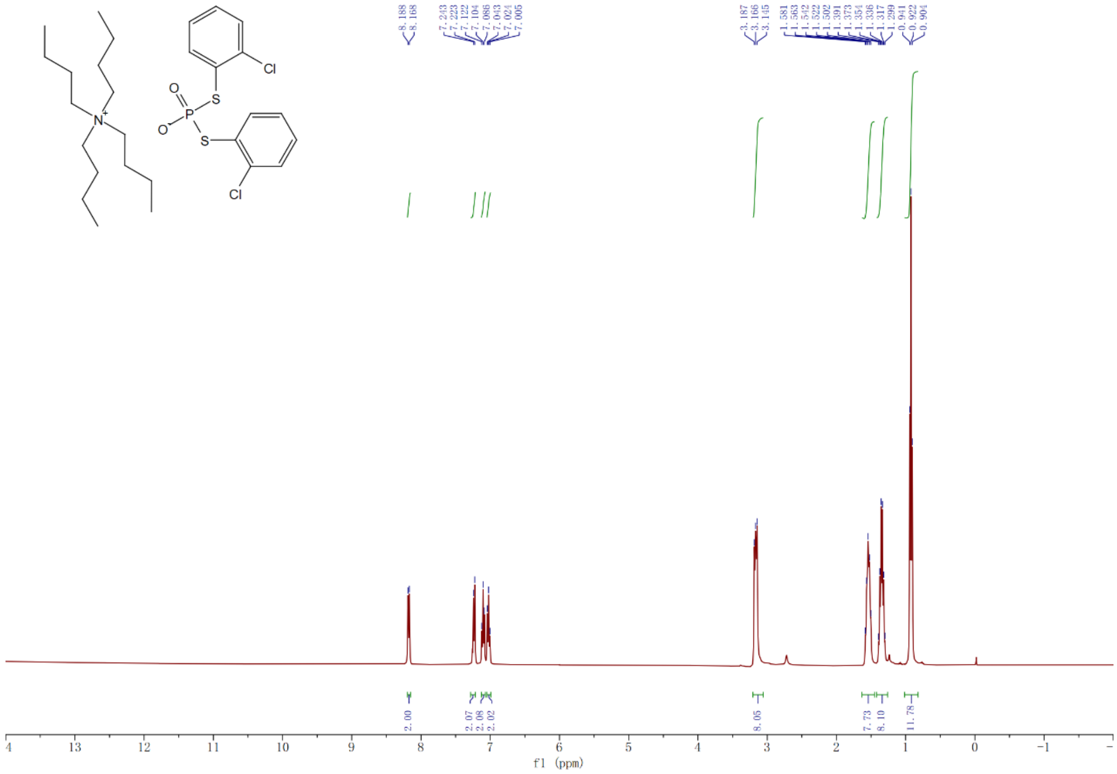

^{31}P NMR (400 MHz, $CDCl_3$)

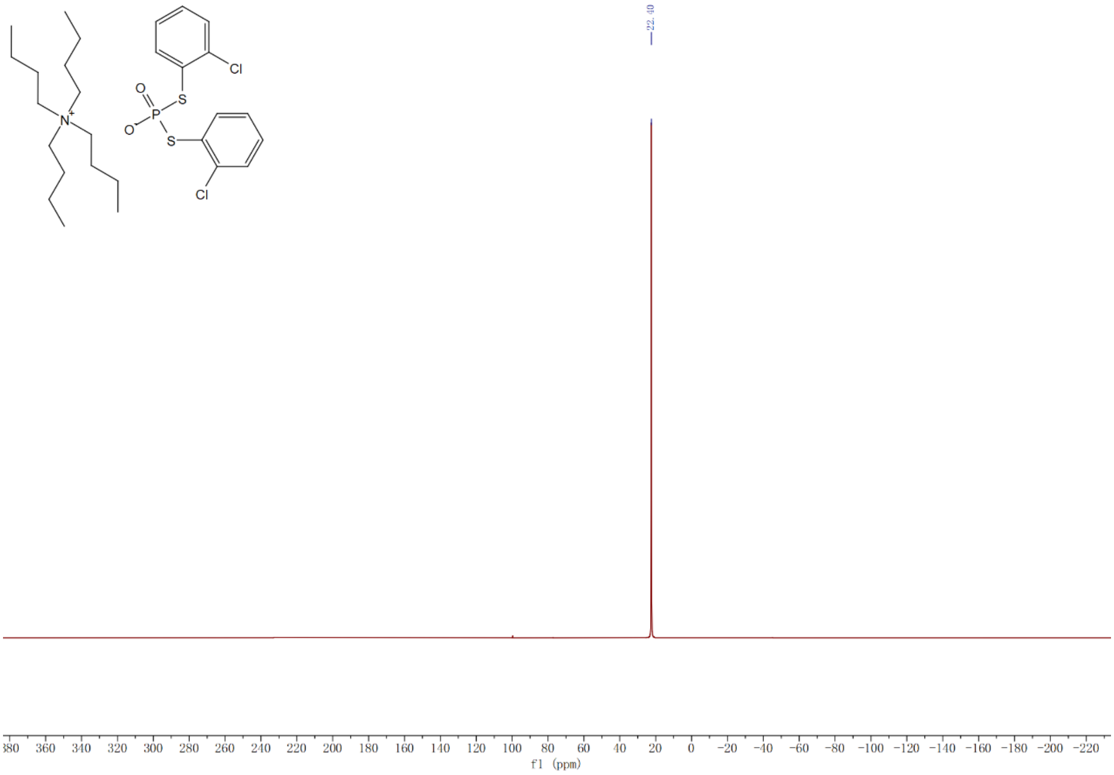

^{13}C NMR (600 MHz, $CDCl_3$)

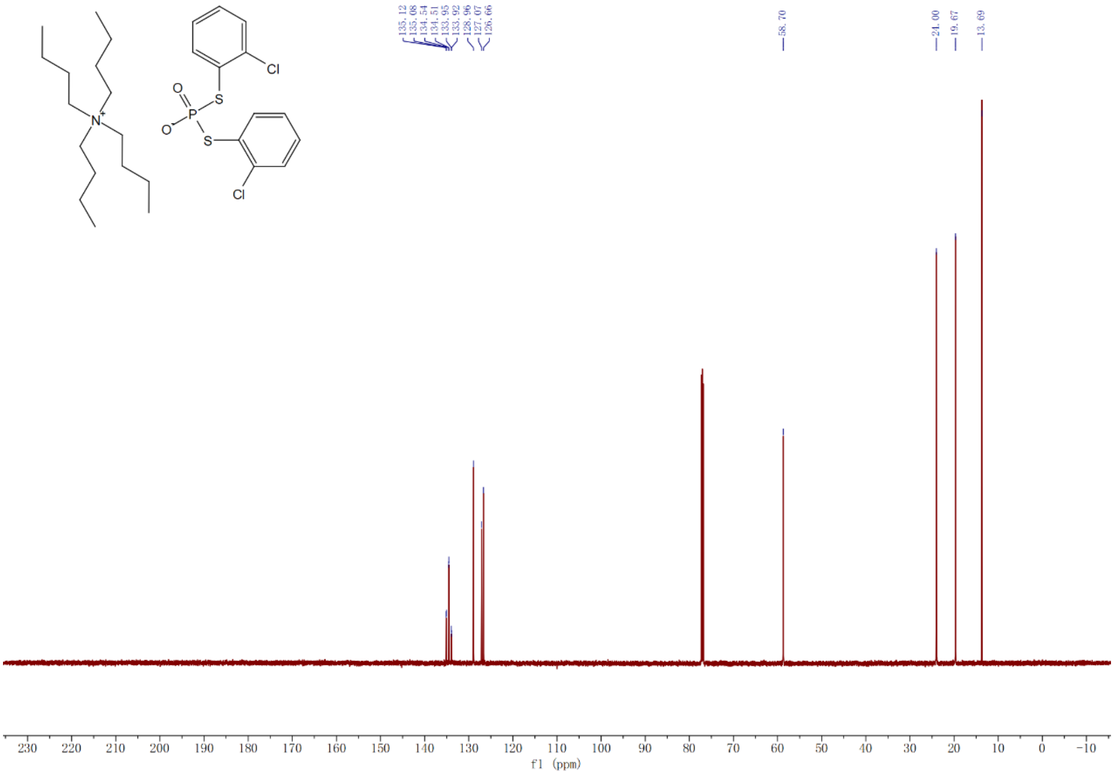

^1H NMR (400 MHz, $CDCl_3$)

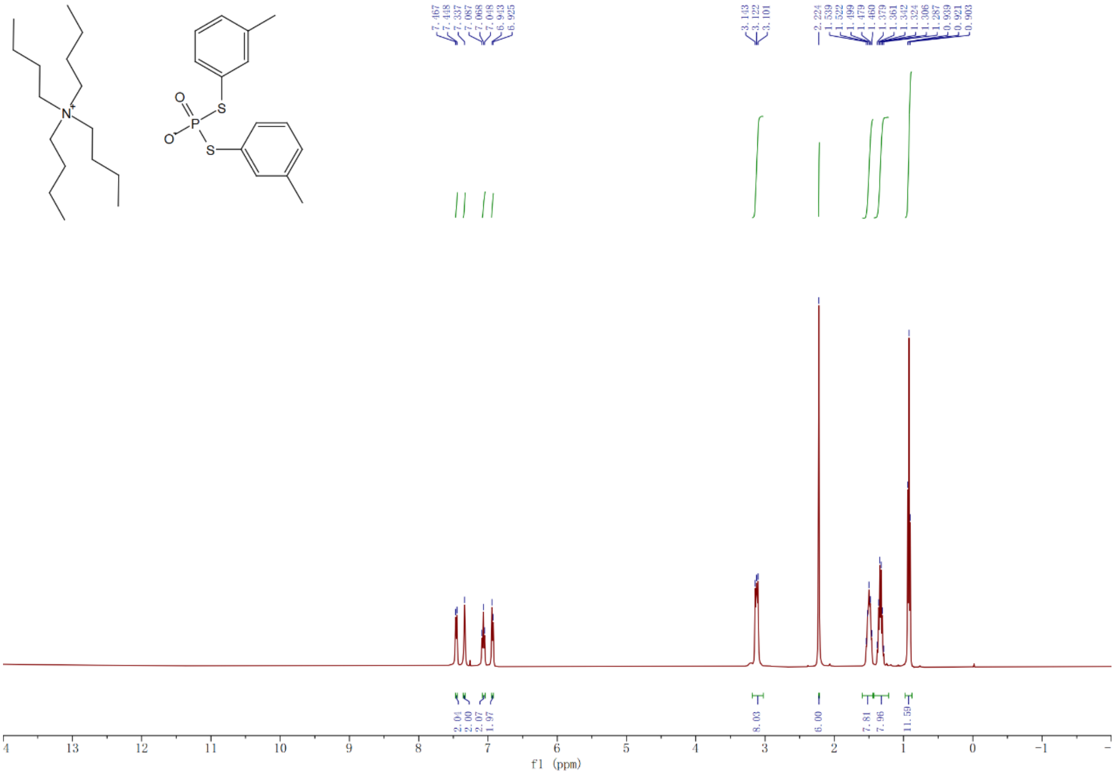

^{31}P NMR (400 MHz, $CDCl_3$)

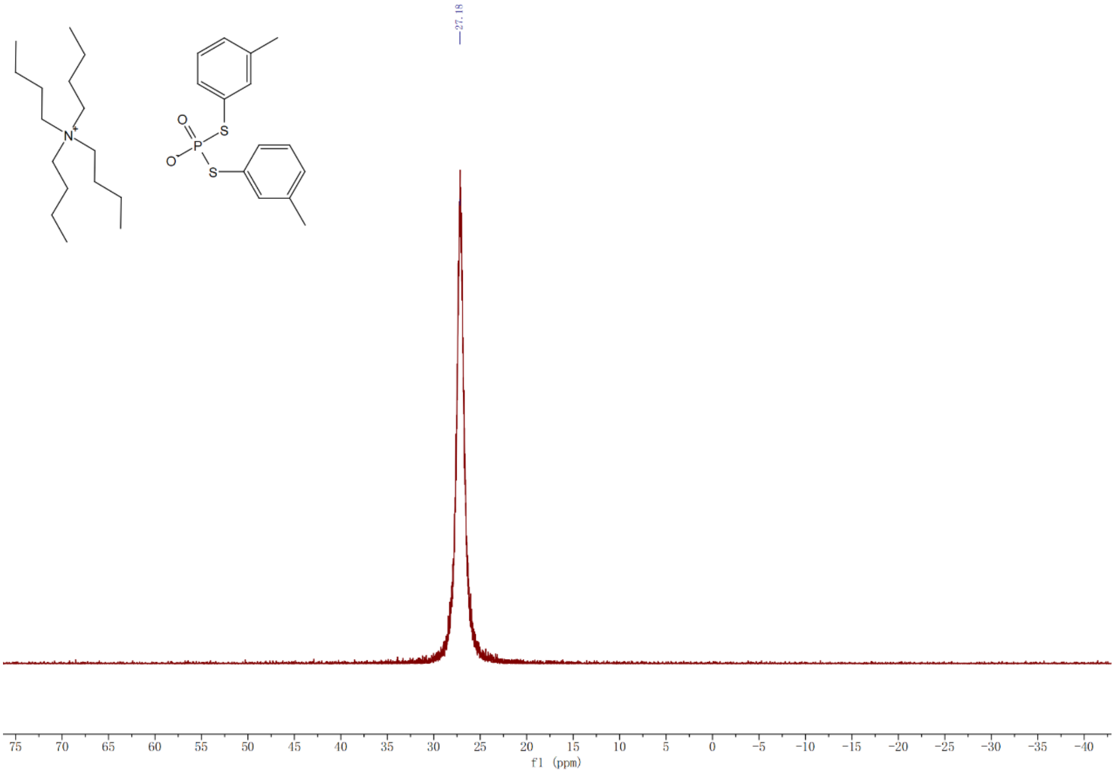

^{13}C NMR (600 MHz, $CDCl_3$)

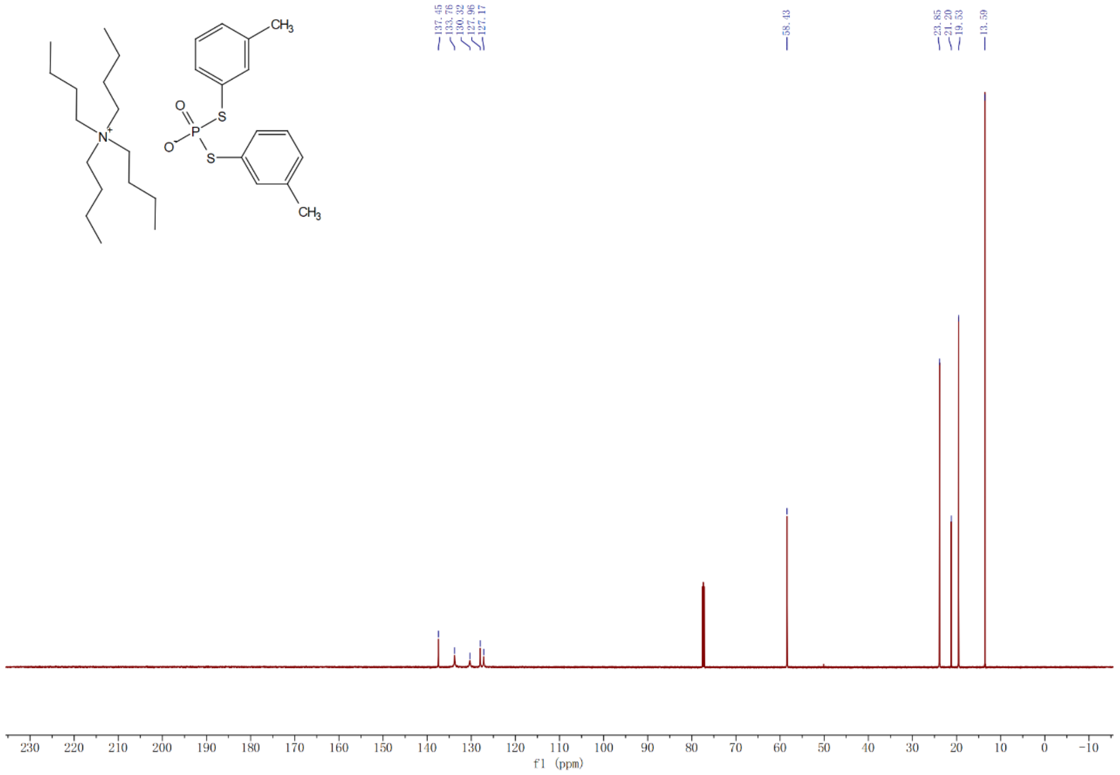

^{1}H NMR (400 MHz, $CDCl_3$)

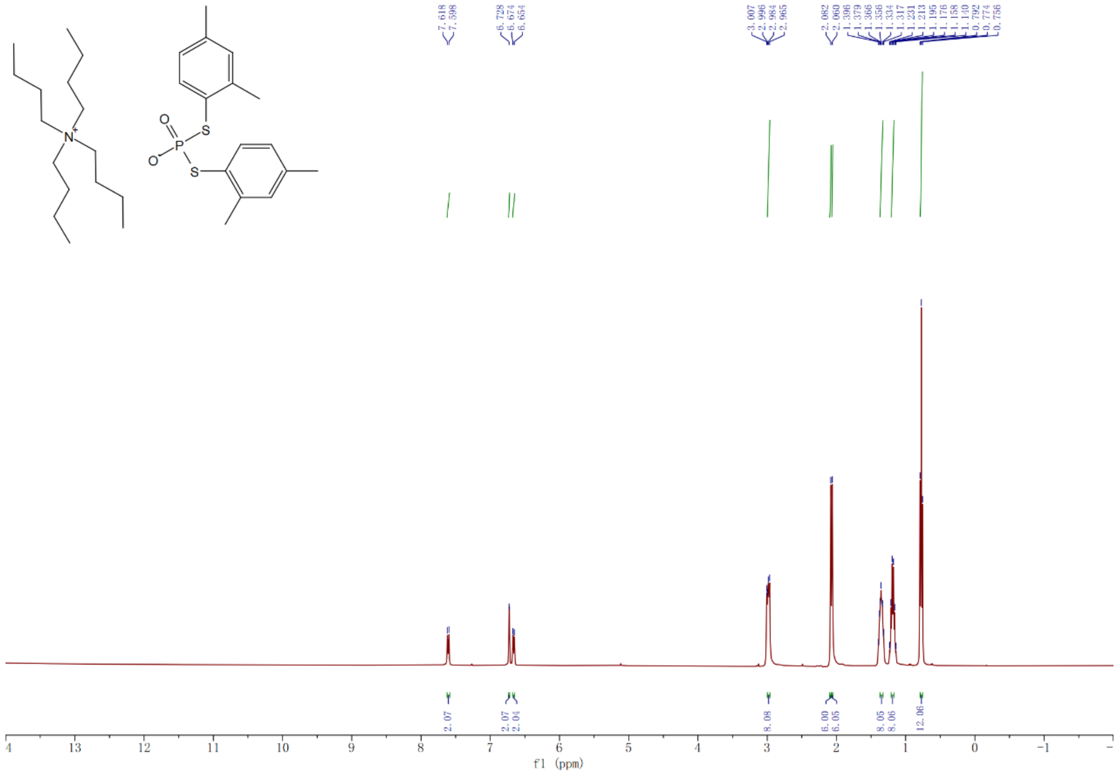

^{31}P NMR (400 MHz, $CDCl_3$)

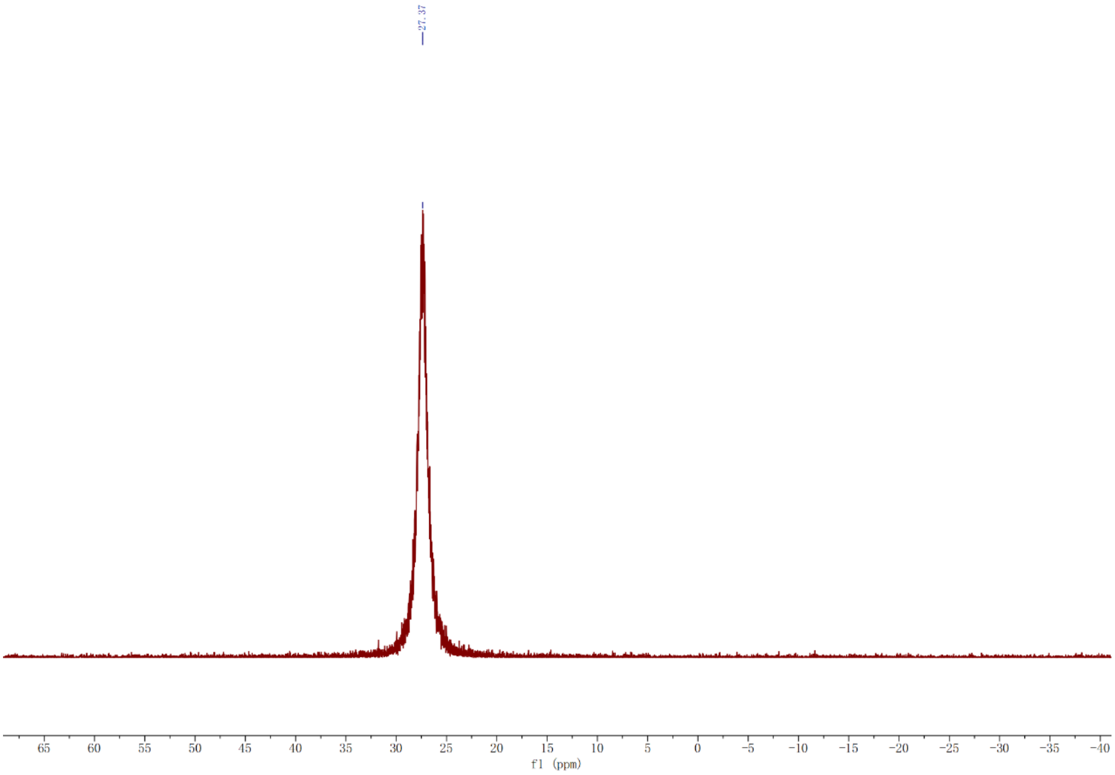

^{13}C NMR (600 MHz, $CDCl_3$)

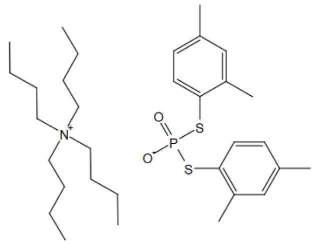

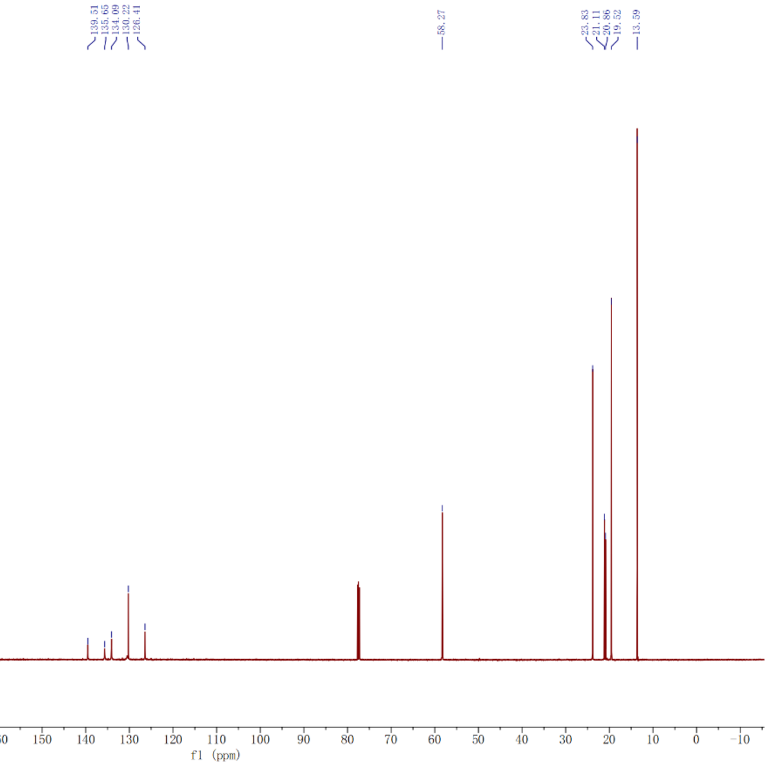

^{1}H NMR (400 MHz, DMSO-D$_6$)

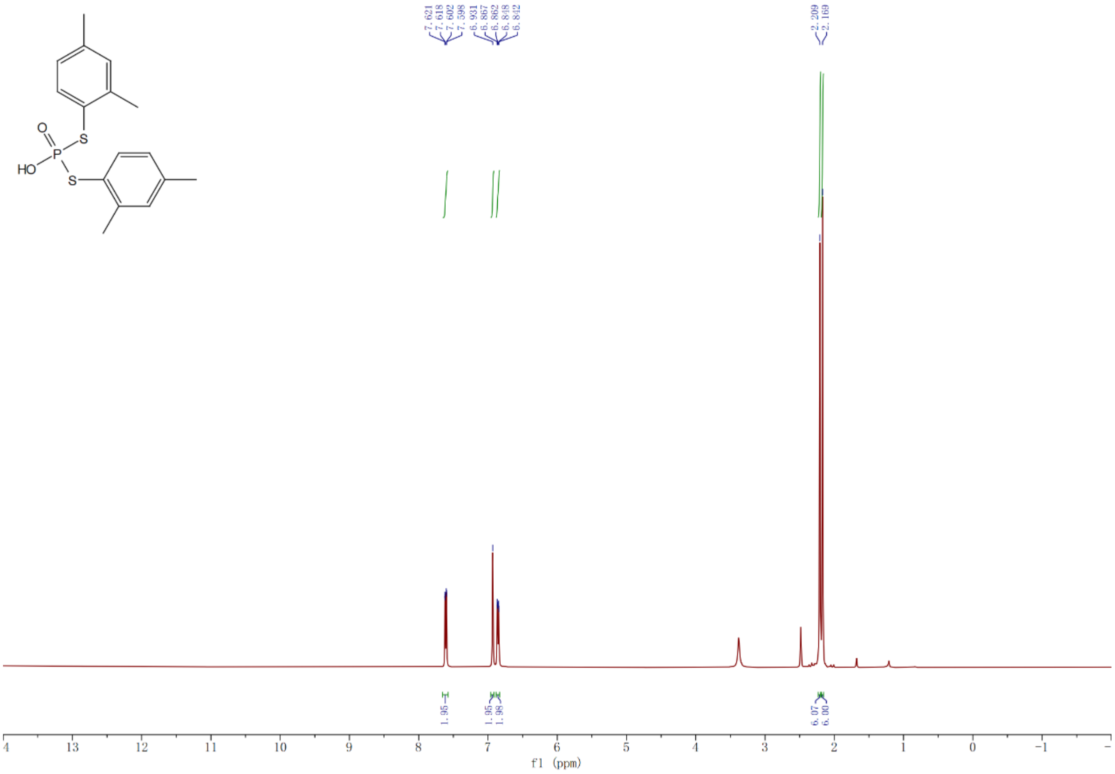

^{31}P NMR (400 MHz, DMSO-D$_6$)

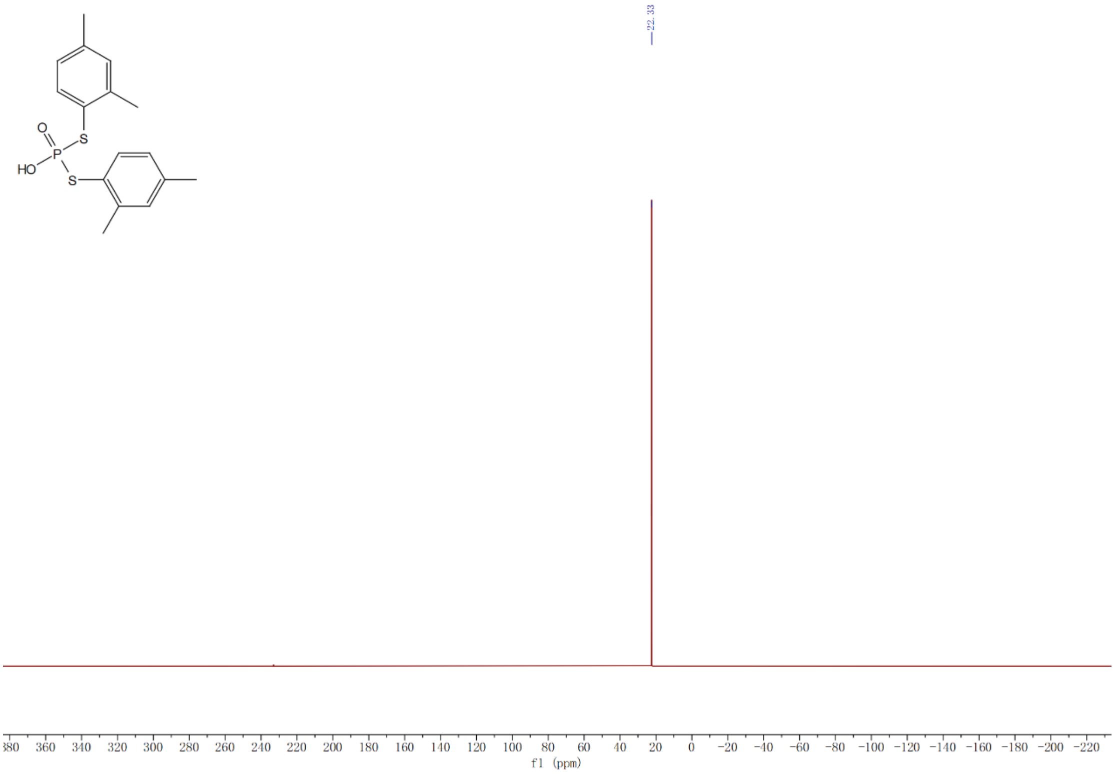

^{13}C NMR (600 MHz, DMSO-D_6)

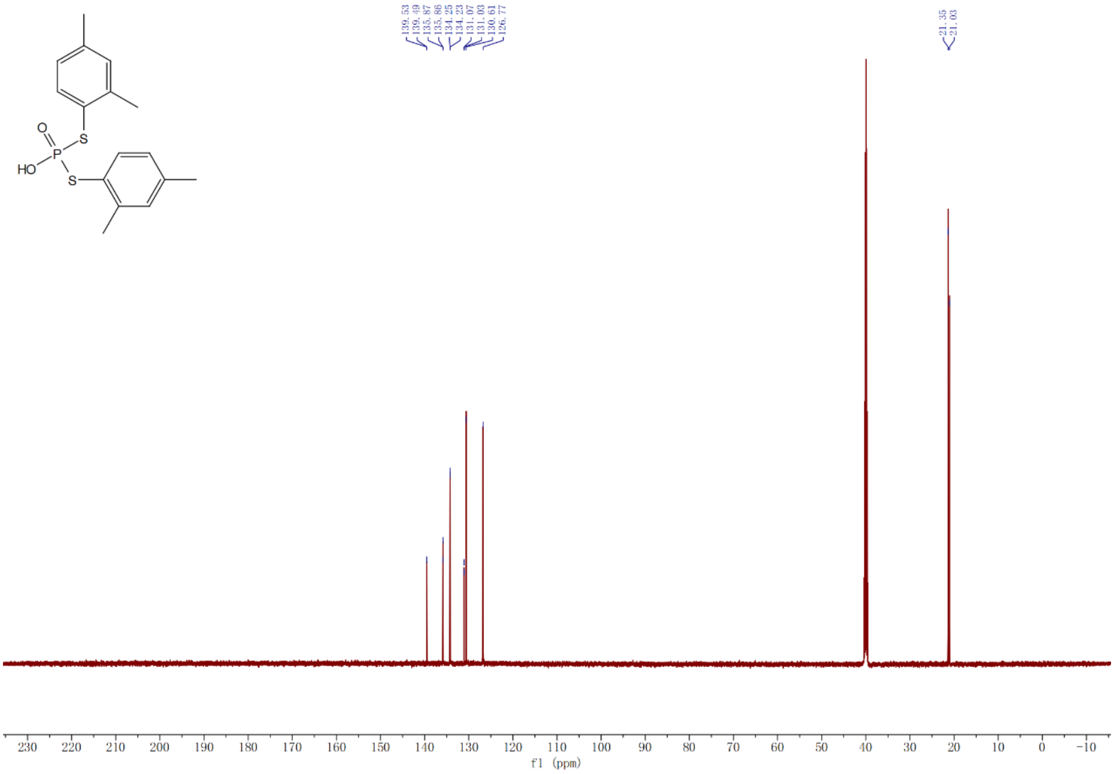

^{1}H NMR (400 MHz, $CDCl_3$)

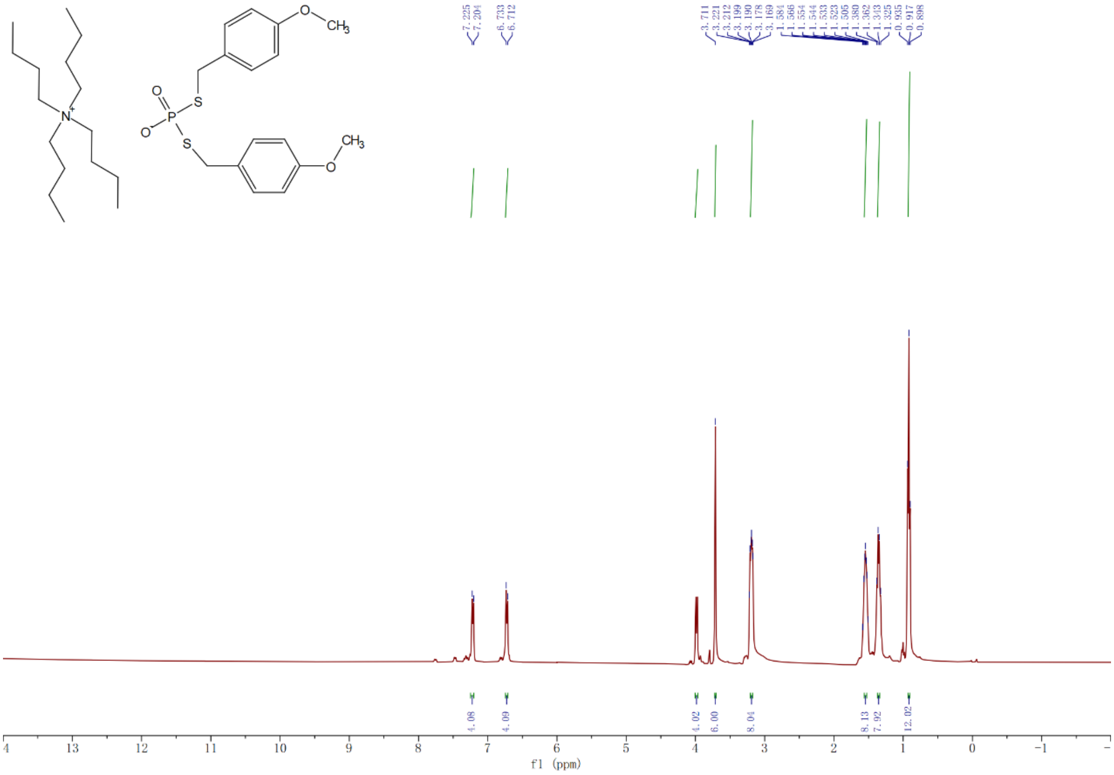

^{31}P NMR (400 MHz, $CDCl_3$)

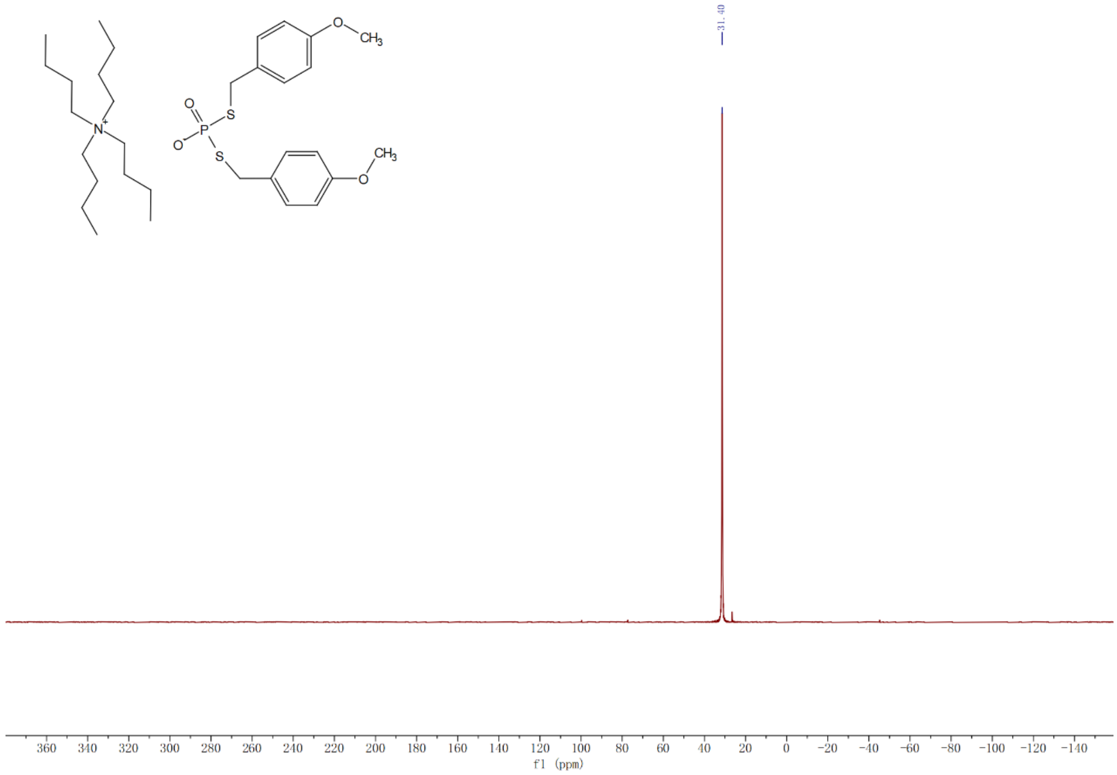

^{13}C NMR (600 MHz, $CDCl_3$)

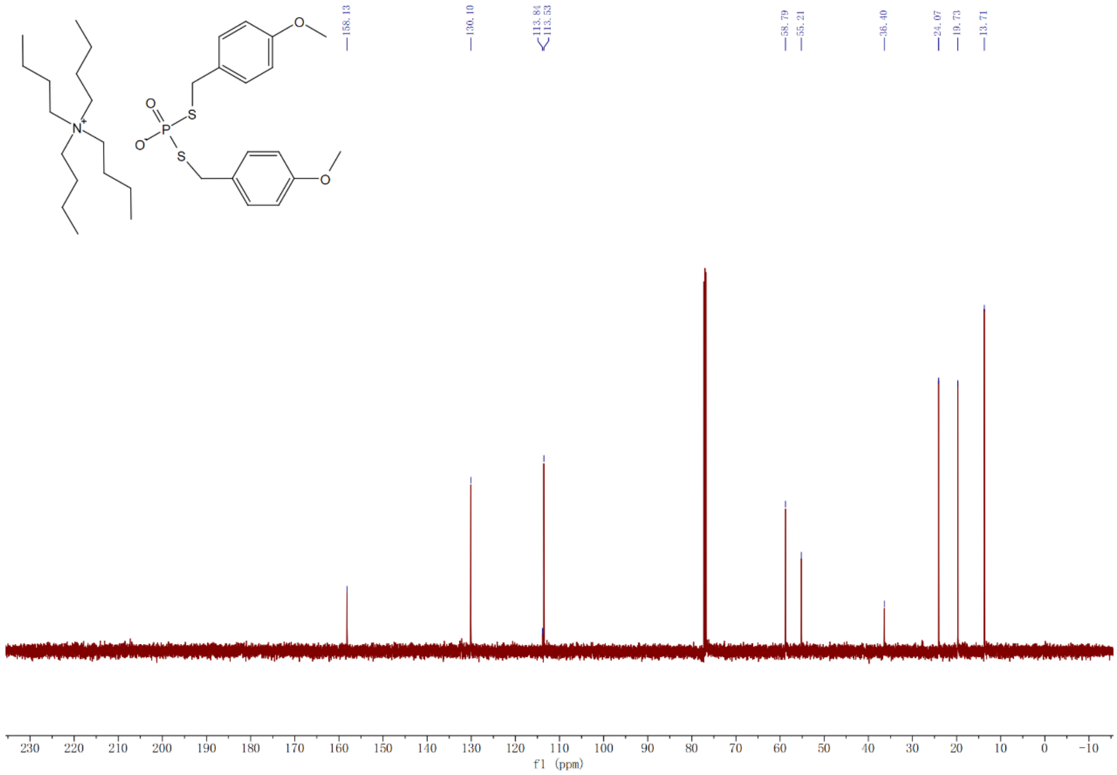

^1H NMR (400 MHz, $CDCl_3$)

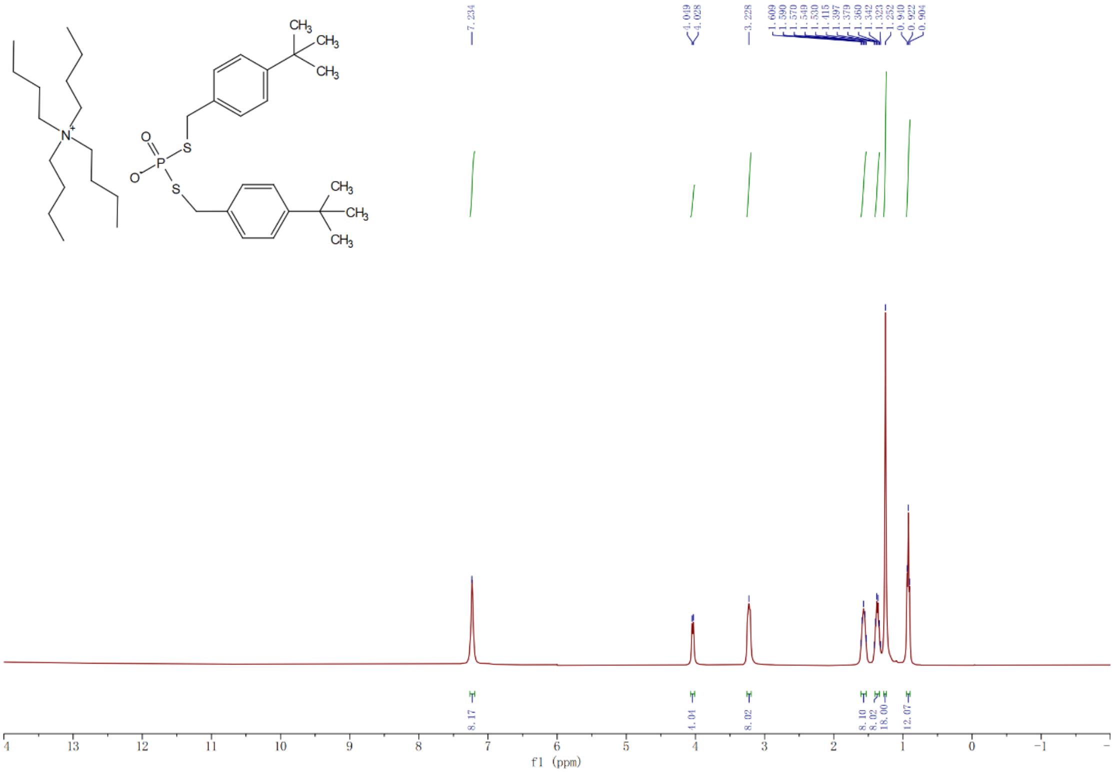

^{31}P NMR (400 MHz, $CDCl_3$)

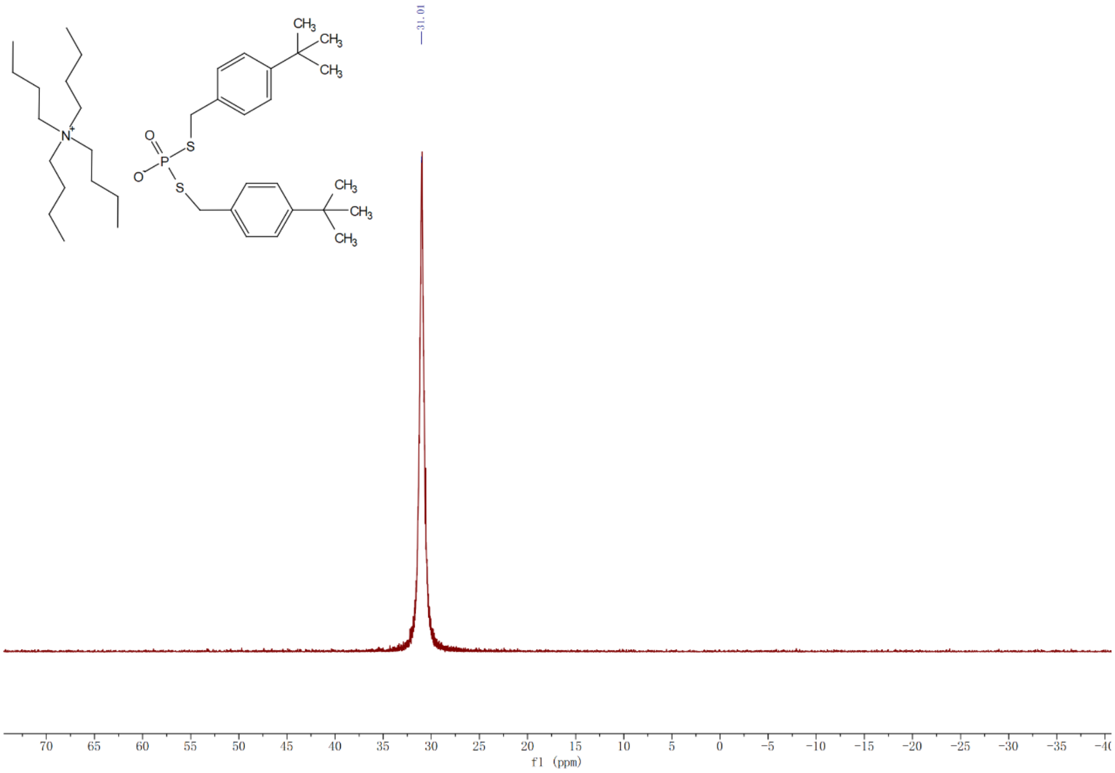

^{13}C NMR (600 MHz, $CDCl_3$)

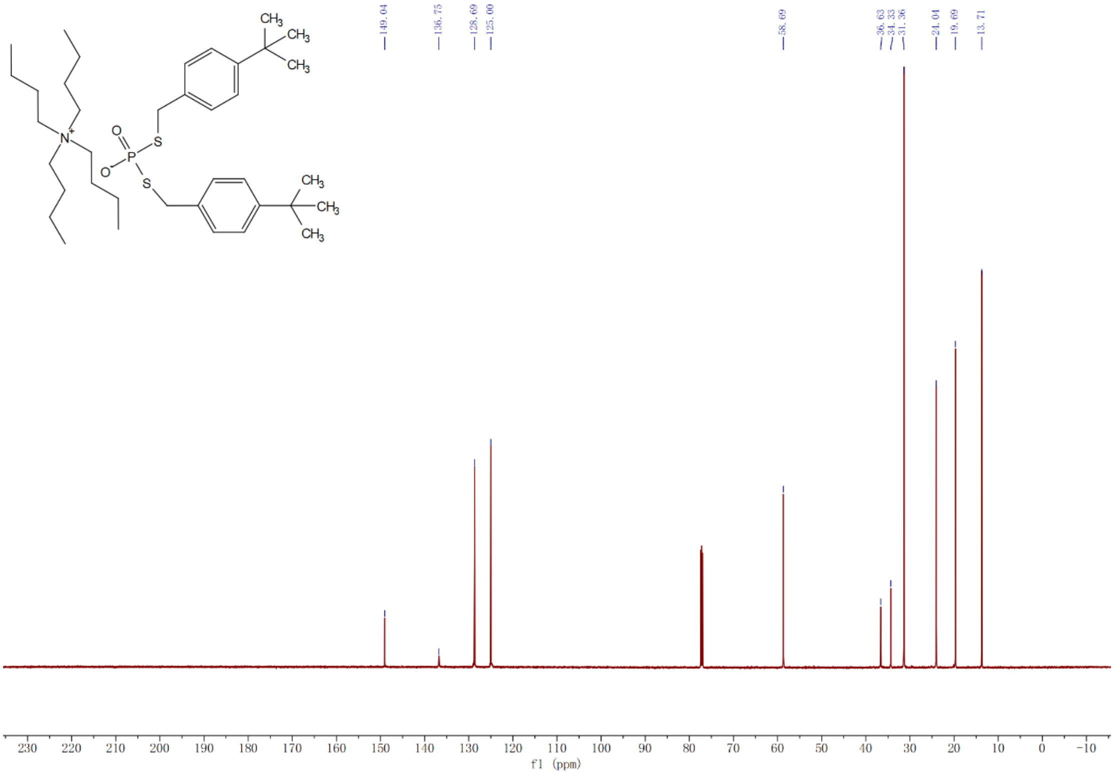

^{1}H NMR (400 MHz, DMSO-D_6)

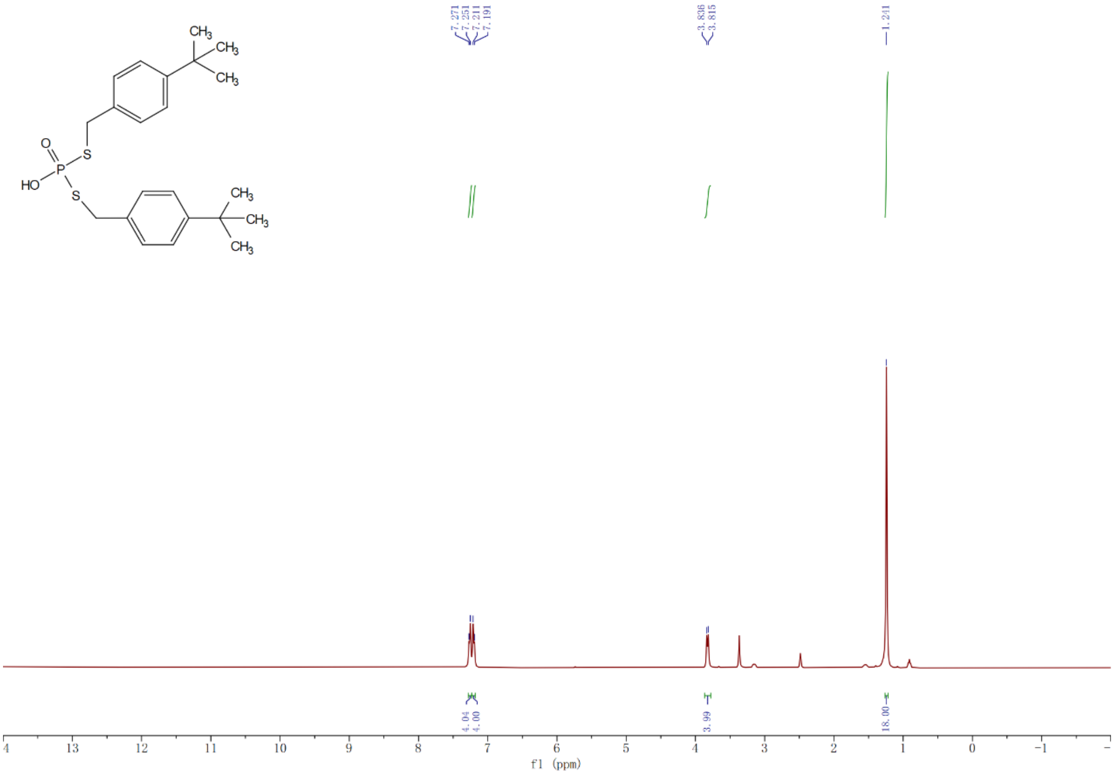

^{31}P NMR (400 MHz, DMSO-D$_6$)

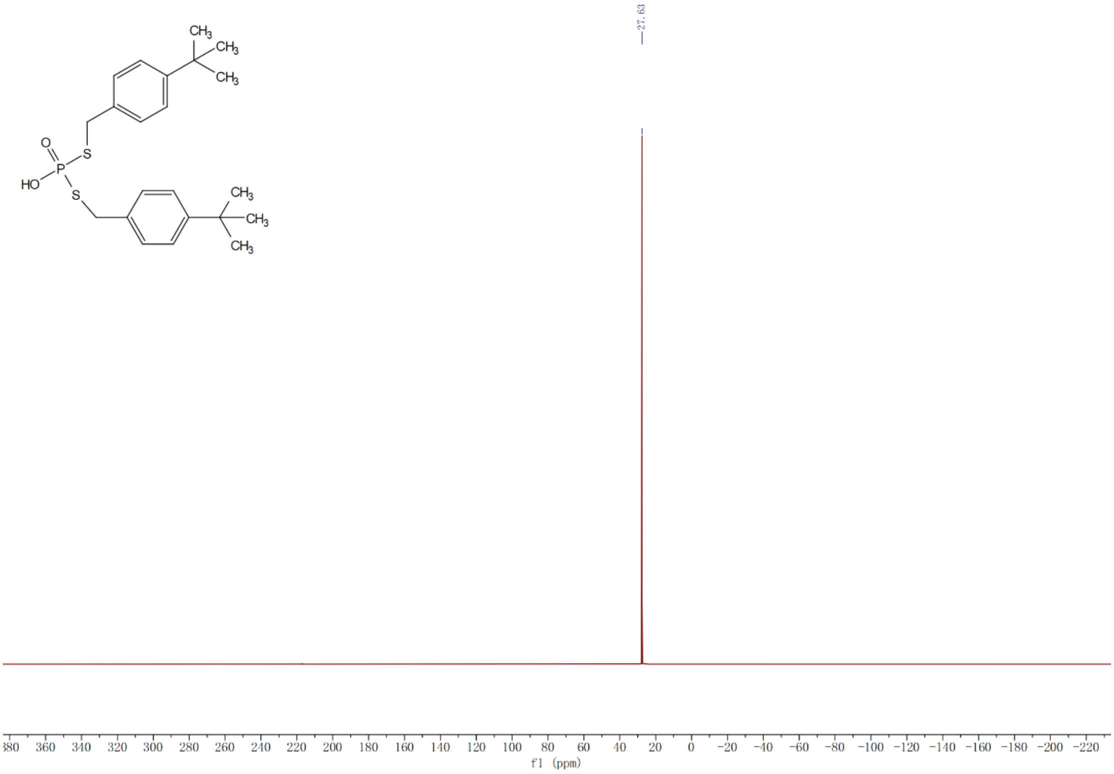

^{13}C NMR (600 MHz, DMSO-D$_6$)

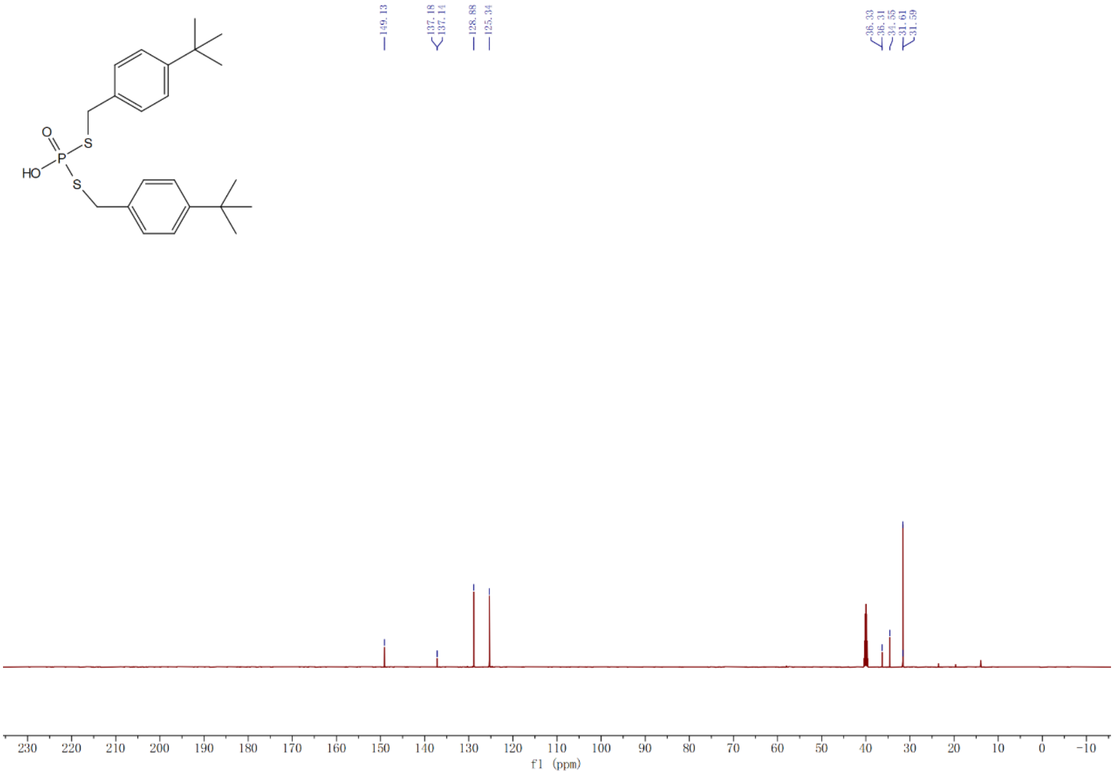

^{1}H NMR (400 MHz, $CDCl_3$)

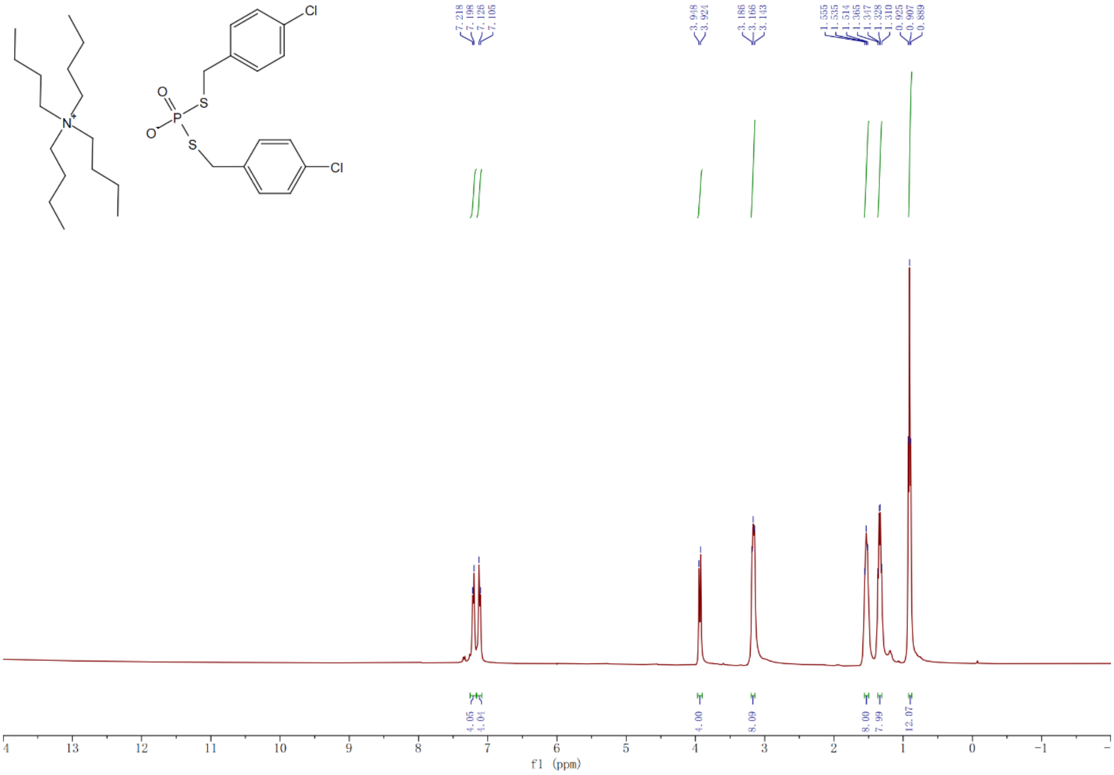

^{31}P NMR (400 MHz, $CDCl_3$)

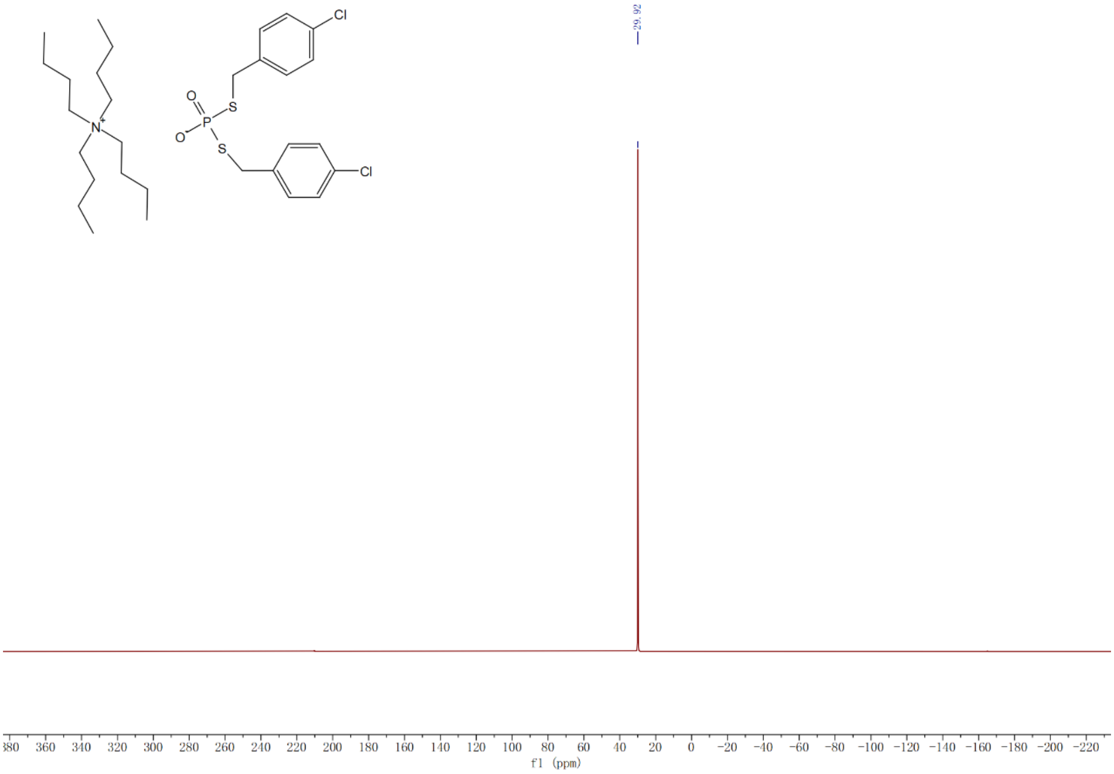

^{13}C NMR (600 MHz, $CDCl_3$)

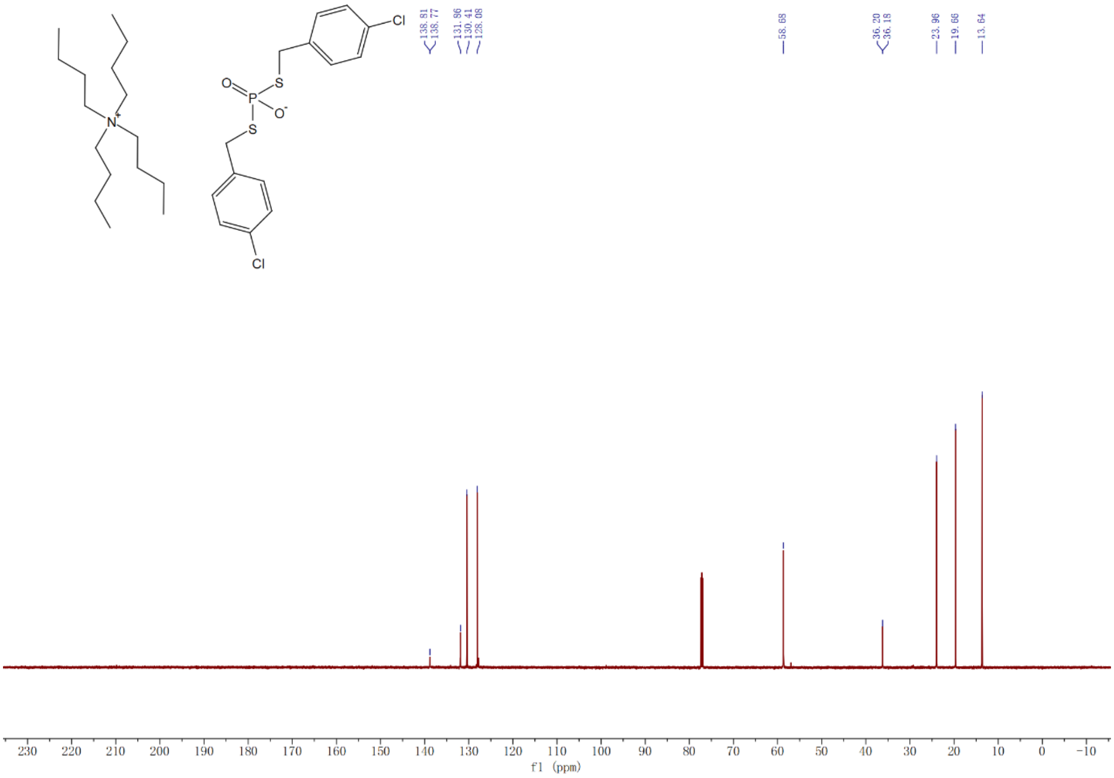

^{1}H NMR (400 MHz, DMSO-D_6)

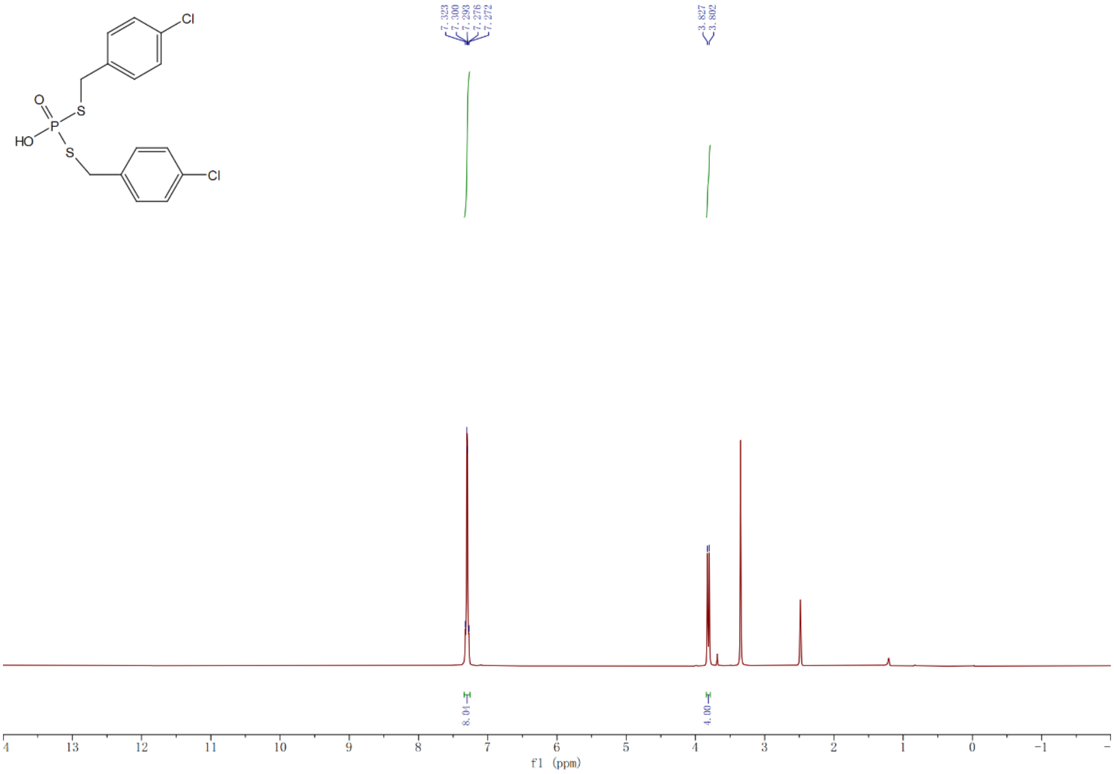

^{31}P NMR (400 MHz, DMSO-D$_6$))

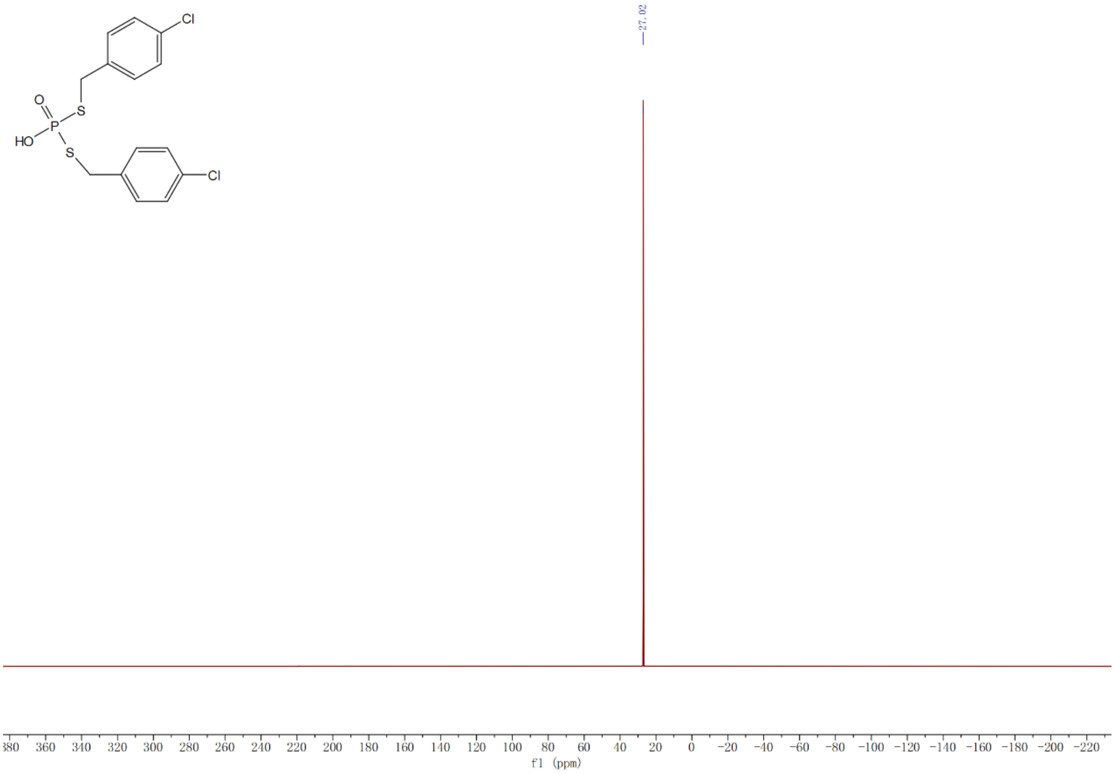

^{13}C NMR (600 MHz, DMSO-D$_6$)

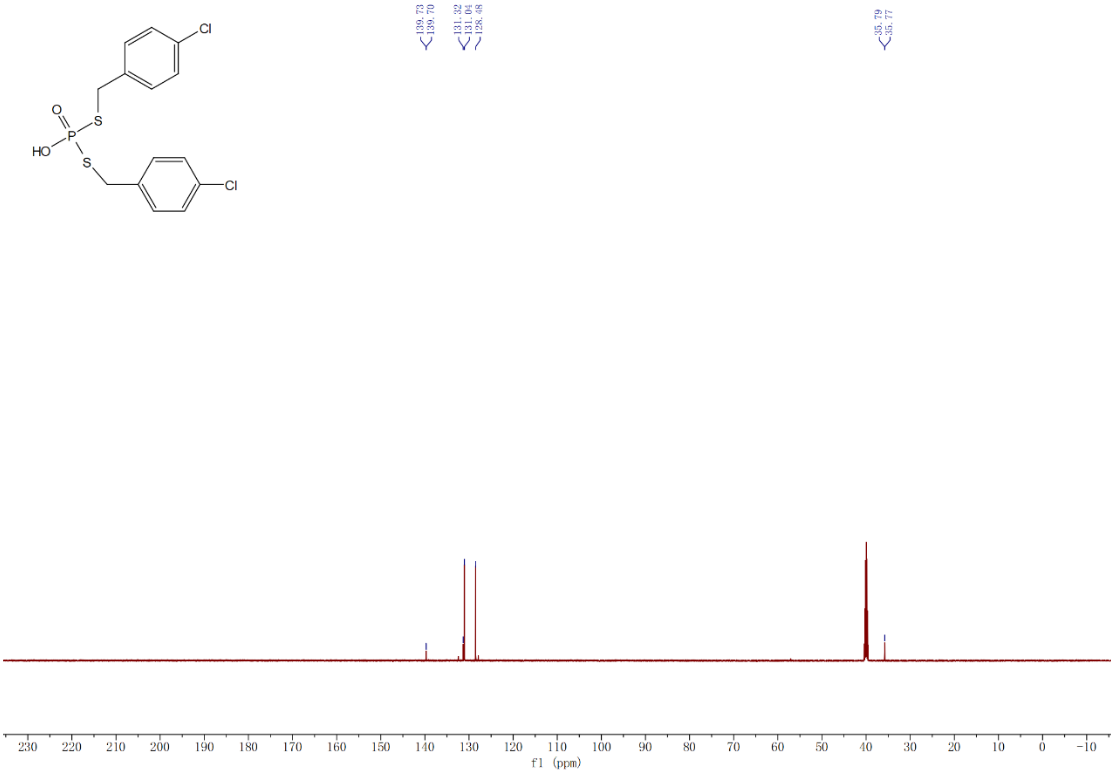

^{1}H NMR (400 MHz, DMSO-D_6)

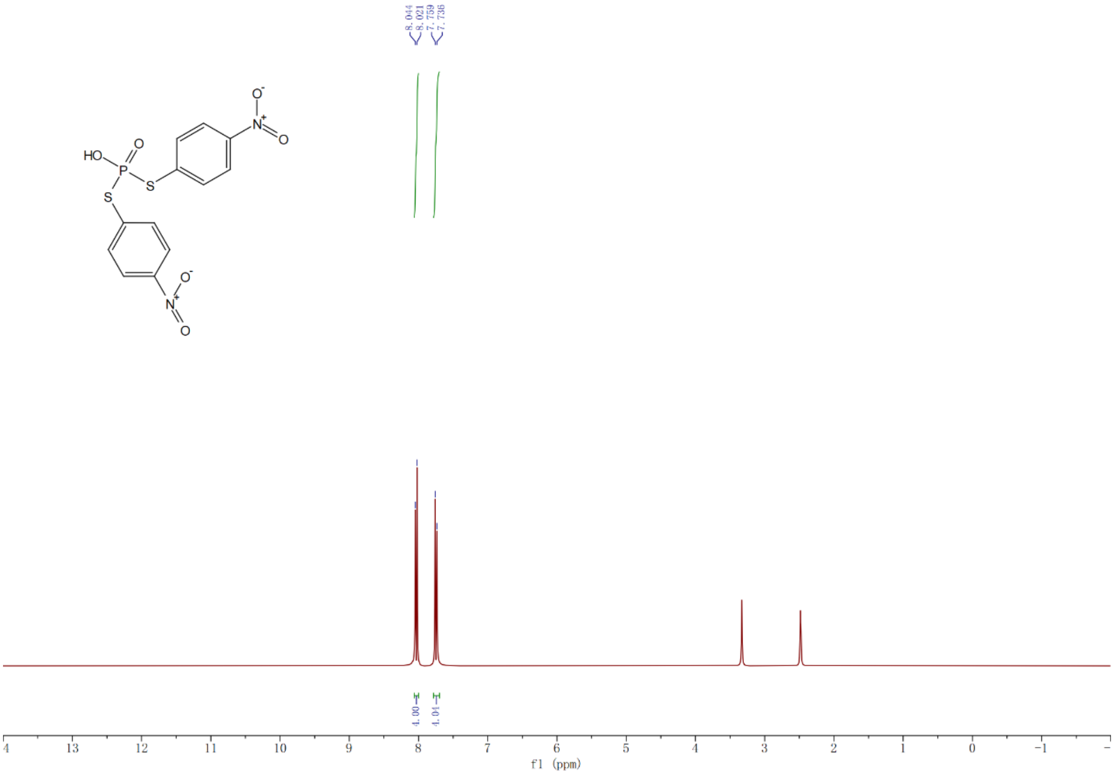

^{31}P NMR (400 MHz, DMSO-D_6)

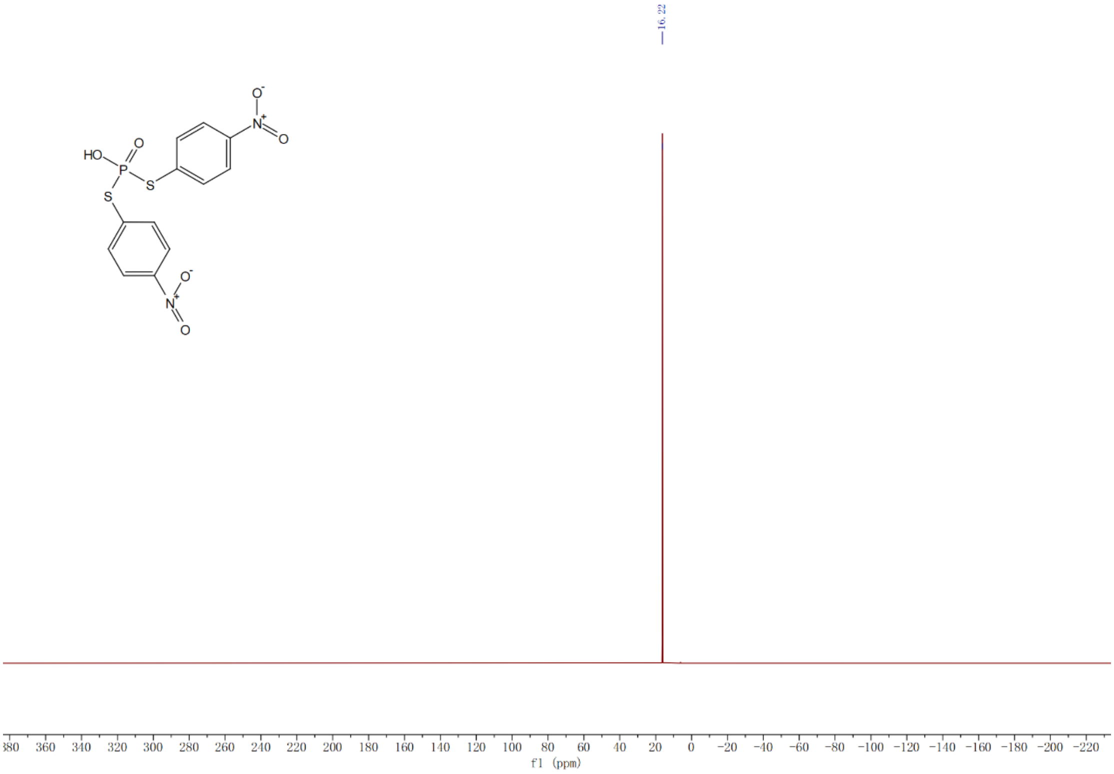

^{13}C NMR (600 MHz, DMSO-D$_6$)

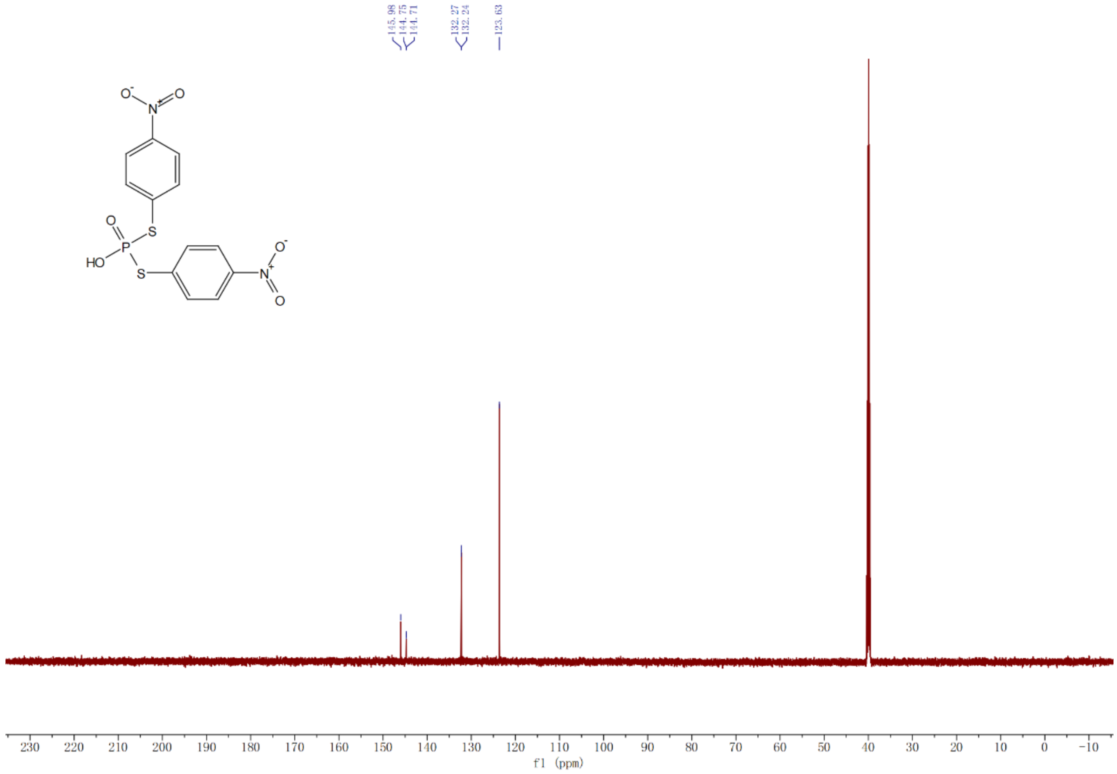

^{1}H NMR (400 MHz, DMSO-D$_6$)

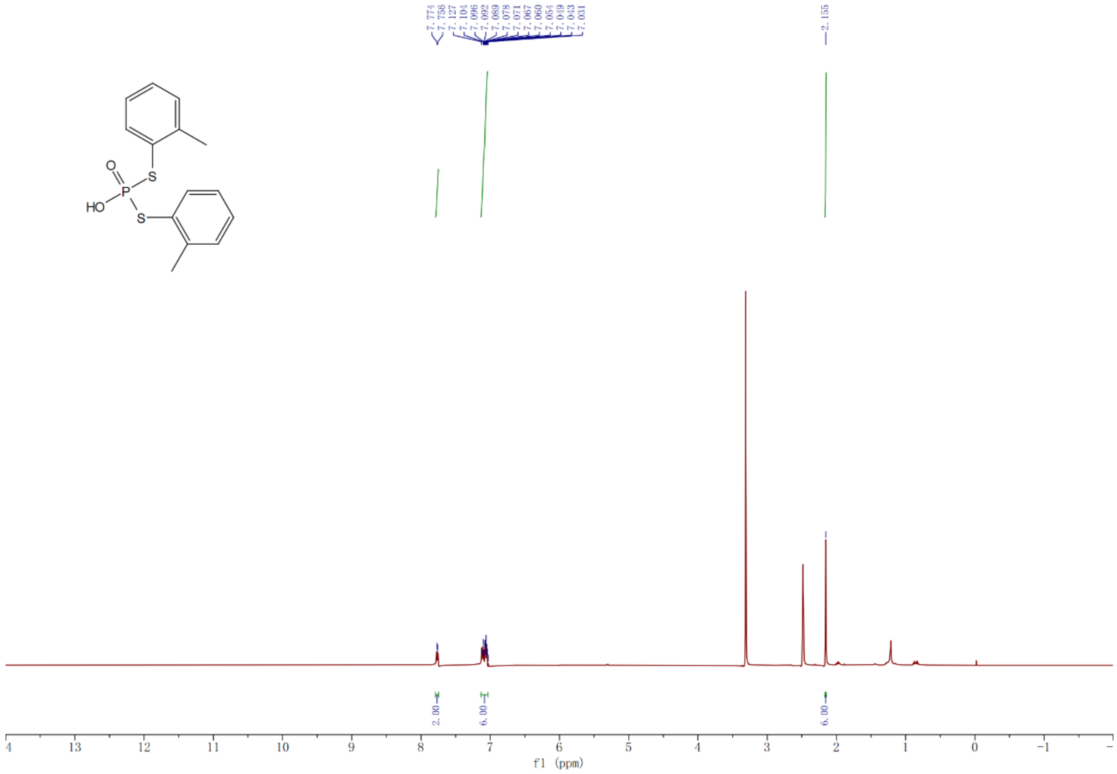

^{31}P NMR (400 MHz, DMSO-D$_6$)

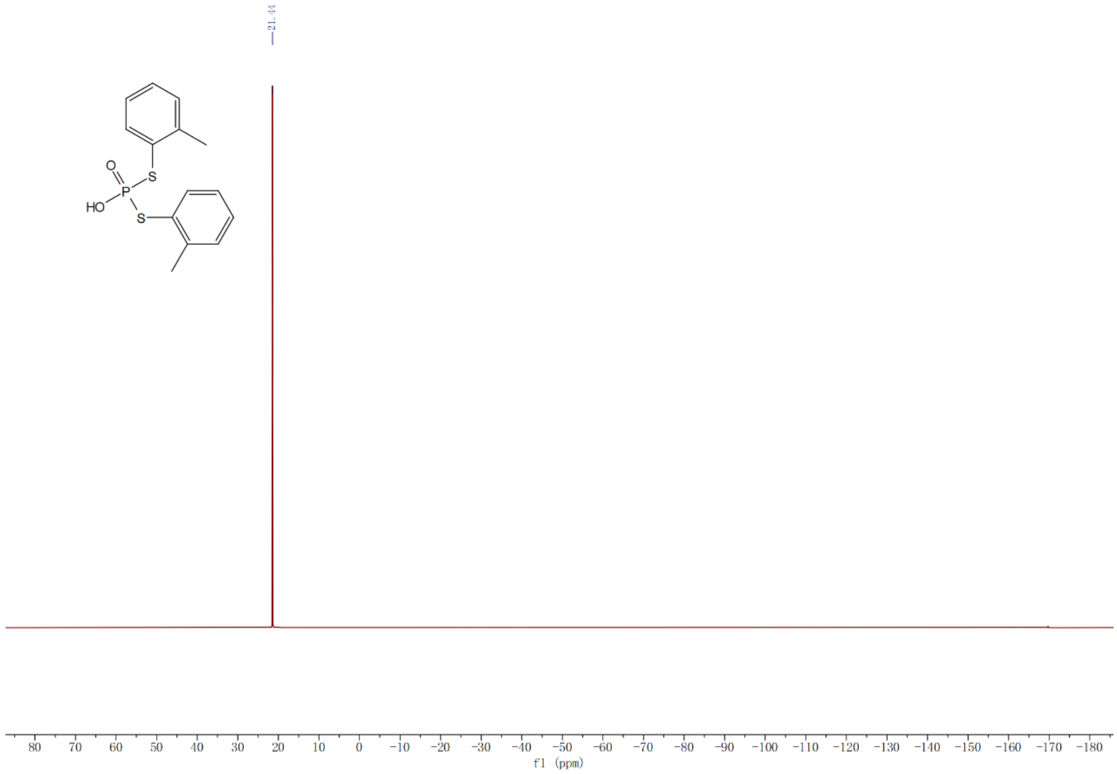

^{13}C NMR (600 MHz, DMSO-D$_6$))

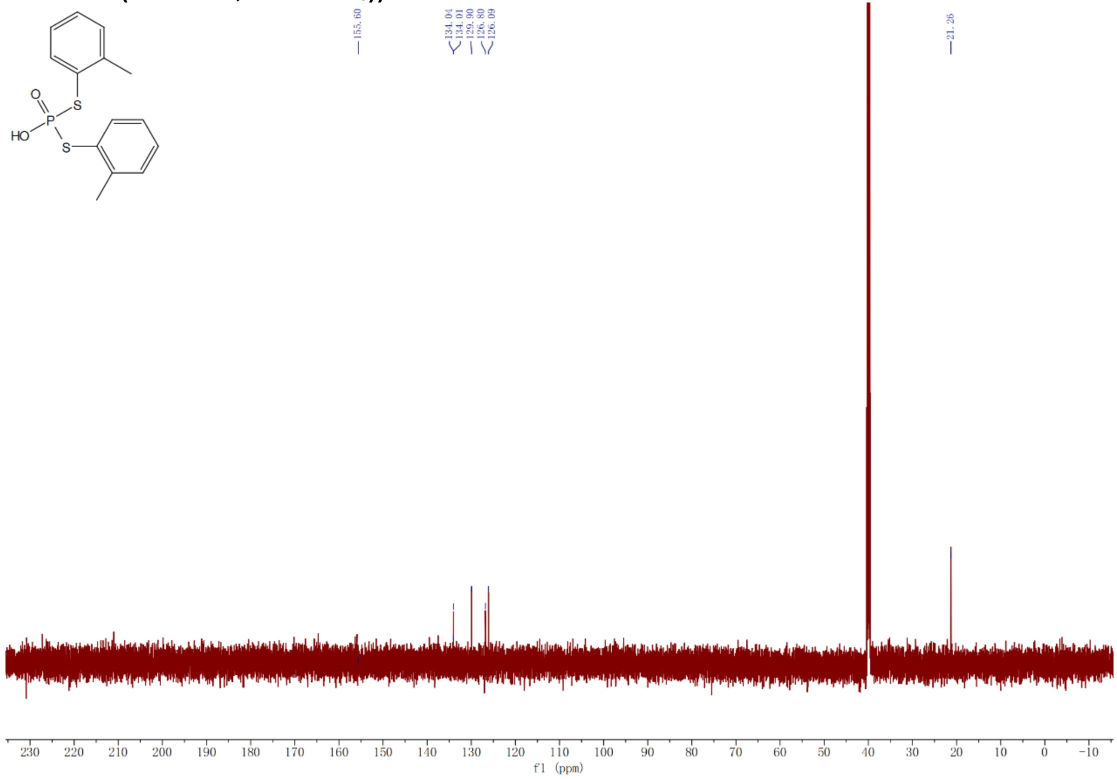

^{1}H NMR (400 MHz, $CDCl_3$)

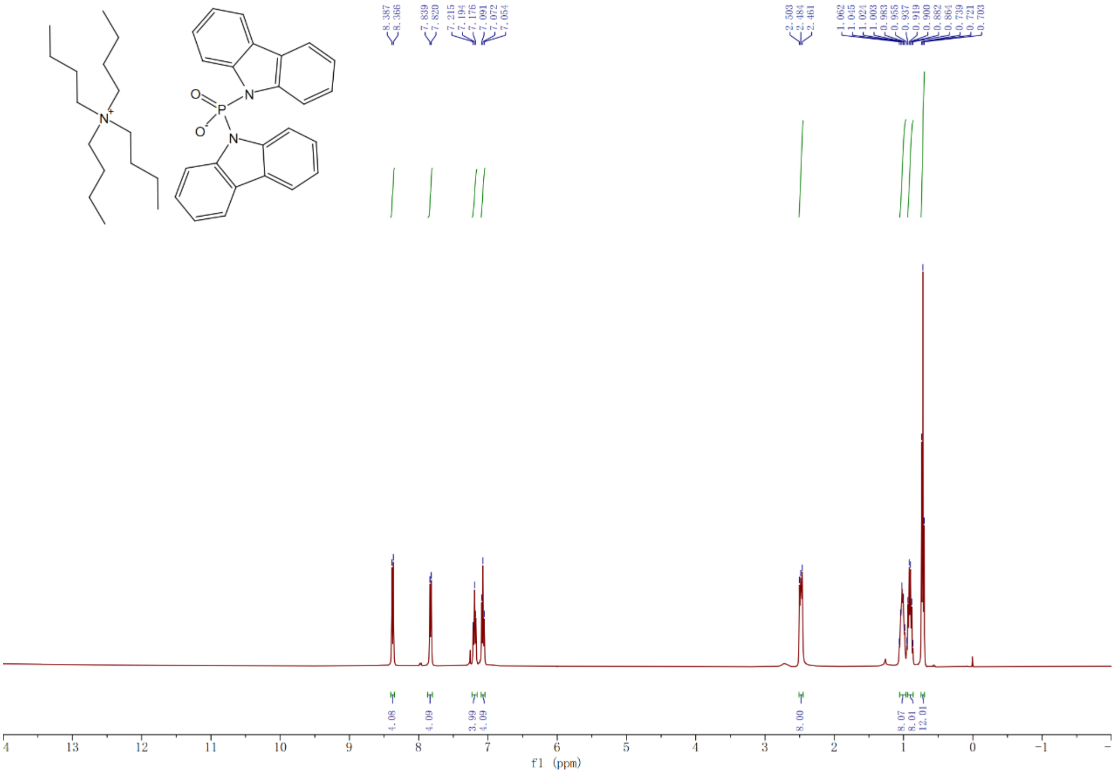

^{31}P NMR (400 MHz, $CDCl_3$)

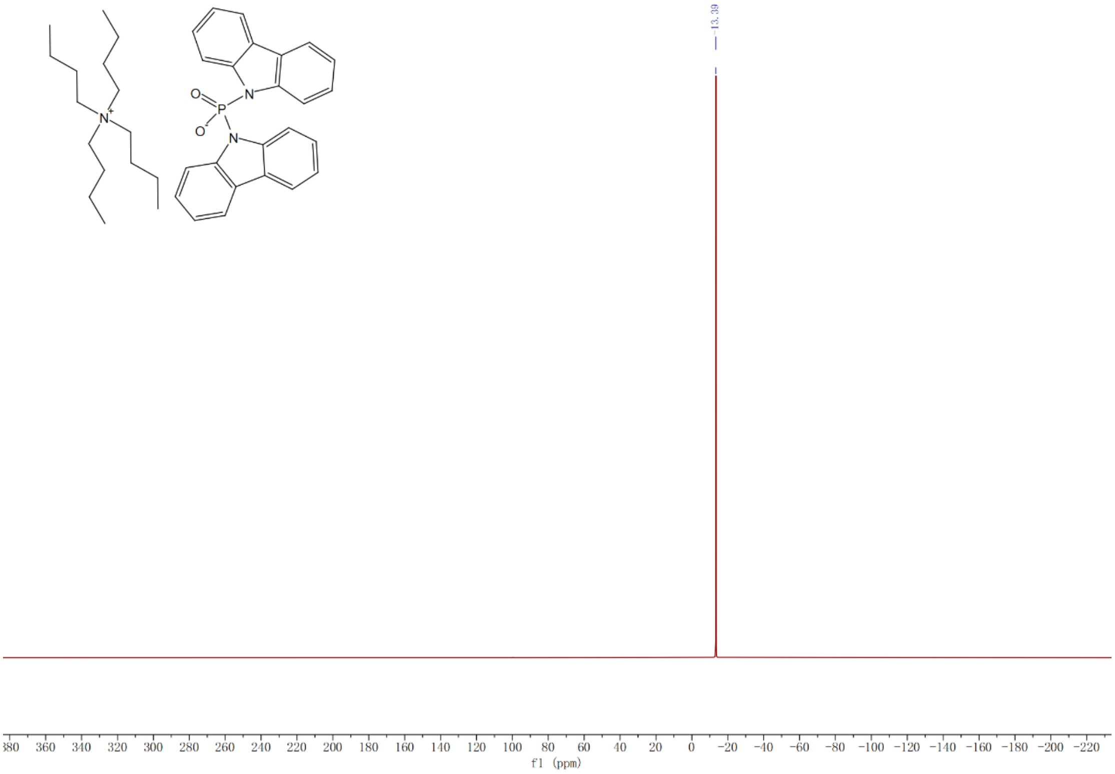

^{13}C NMR (600 MHz, $CDCl_3$)

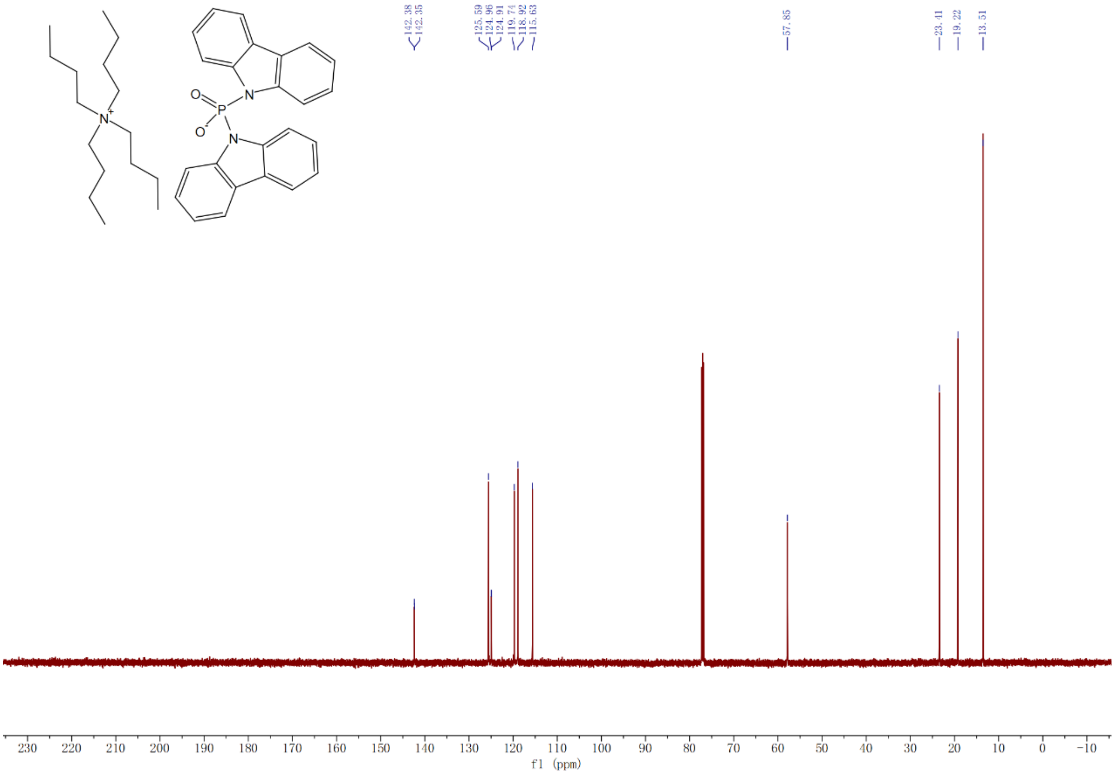

^1H NMR (400 MHz, DMSO-D_6)

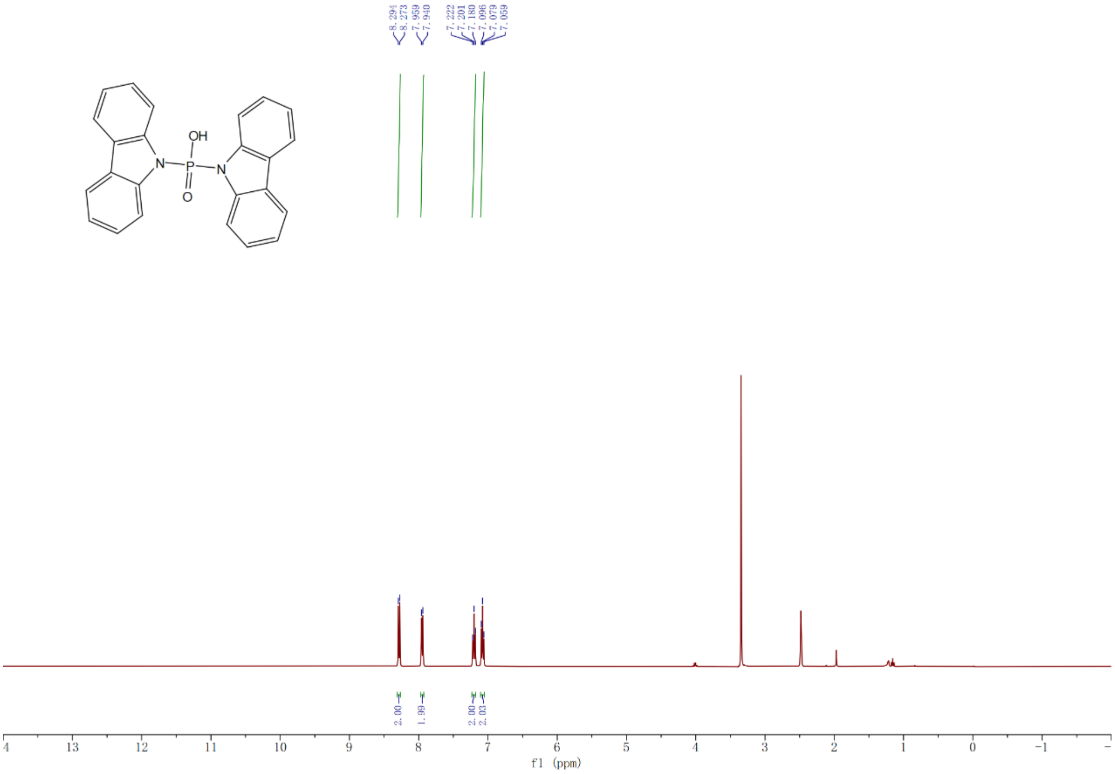

^{31}P NMR (400 MHz, DMSO-D$_6$)

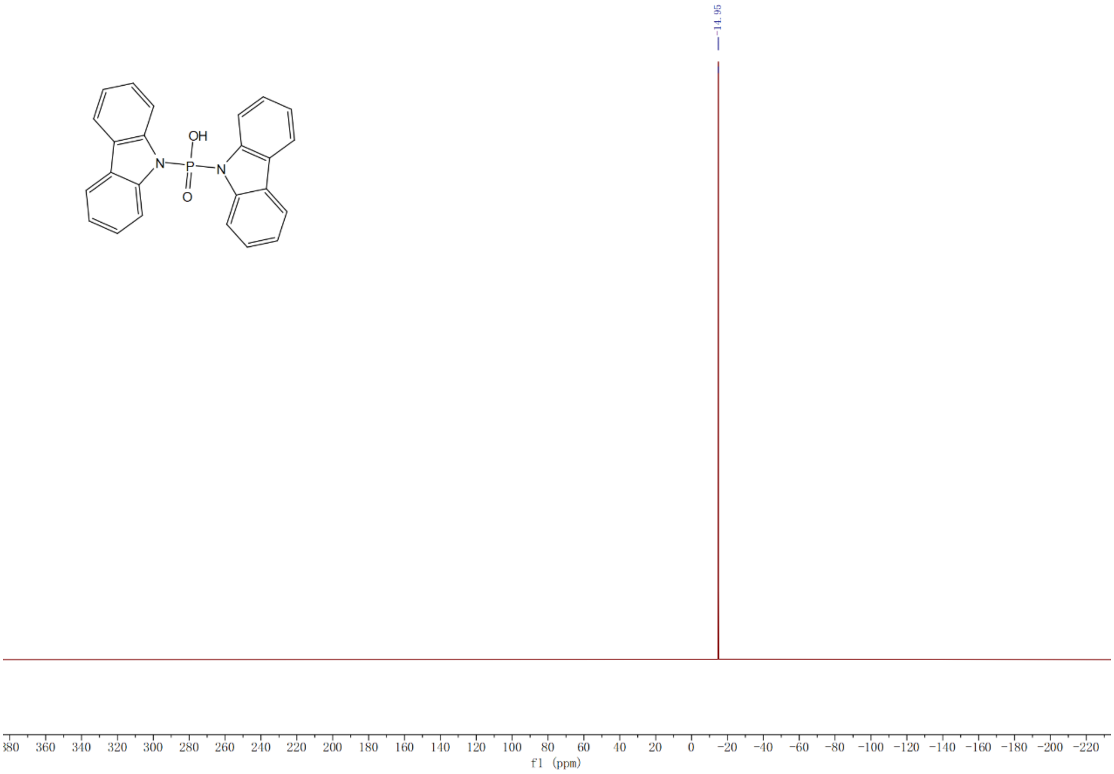

^{13}C NMR (600 MHz, DMSO-D$_6$)

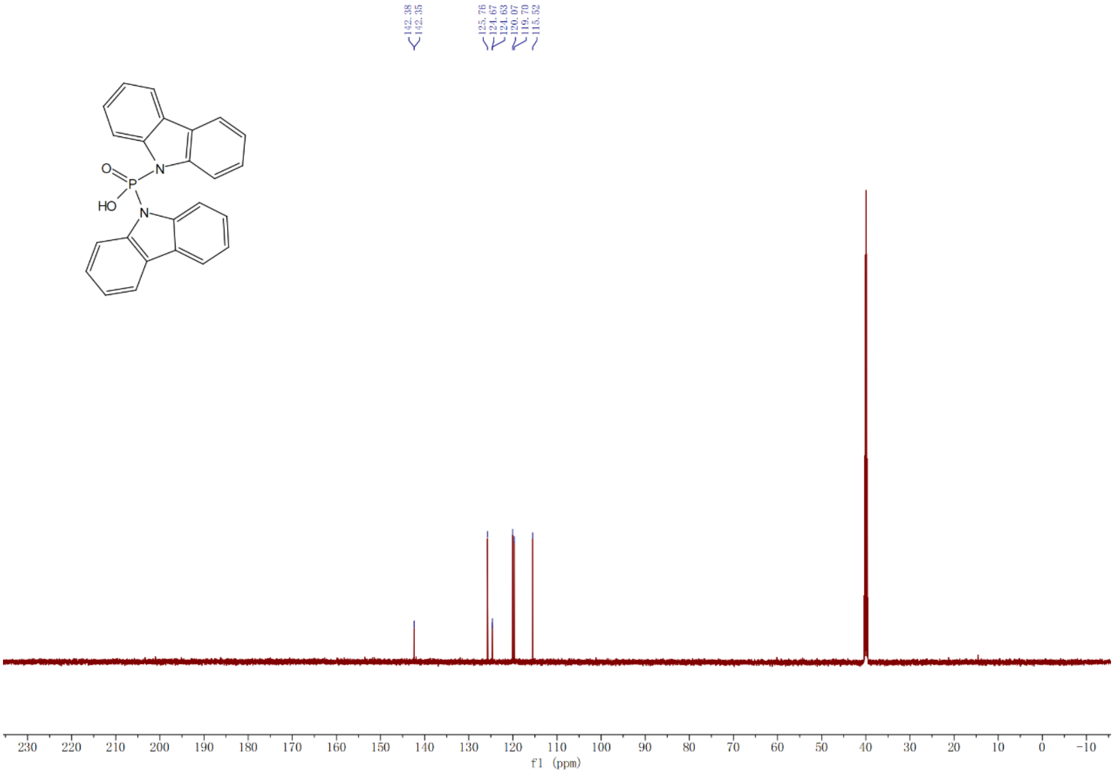

^{1}H NMR (400 MHz, $CDCl_3$)

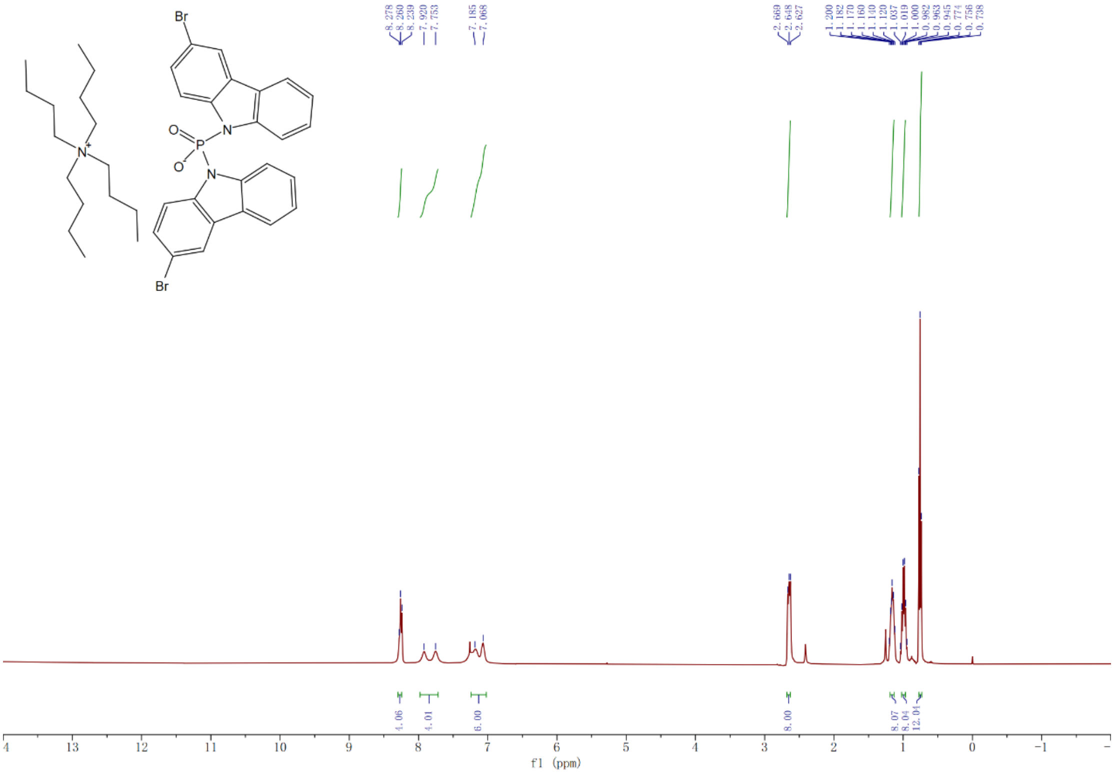

^{31}P NMR (400 MHz, $CDCl_3$)

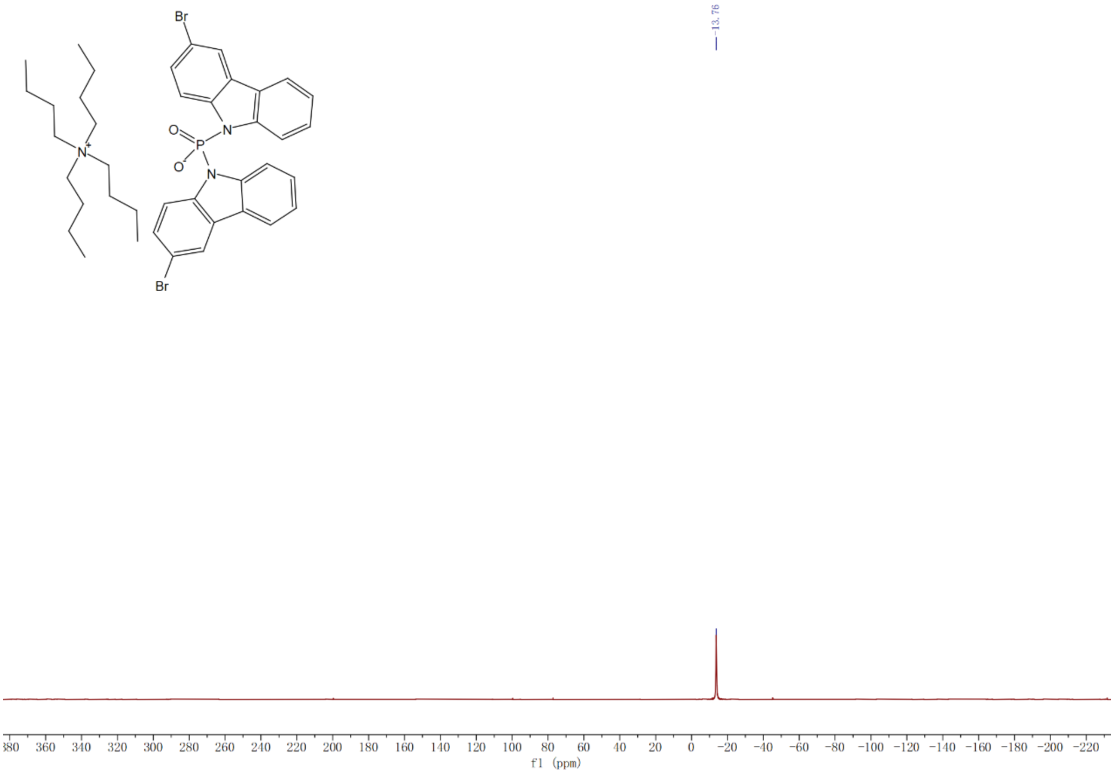

^{13}C NMR (600 MHz, CDCl$_3$)

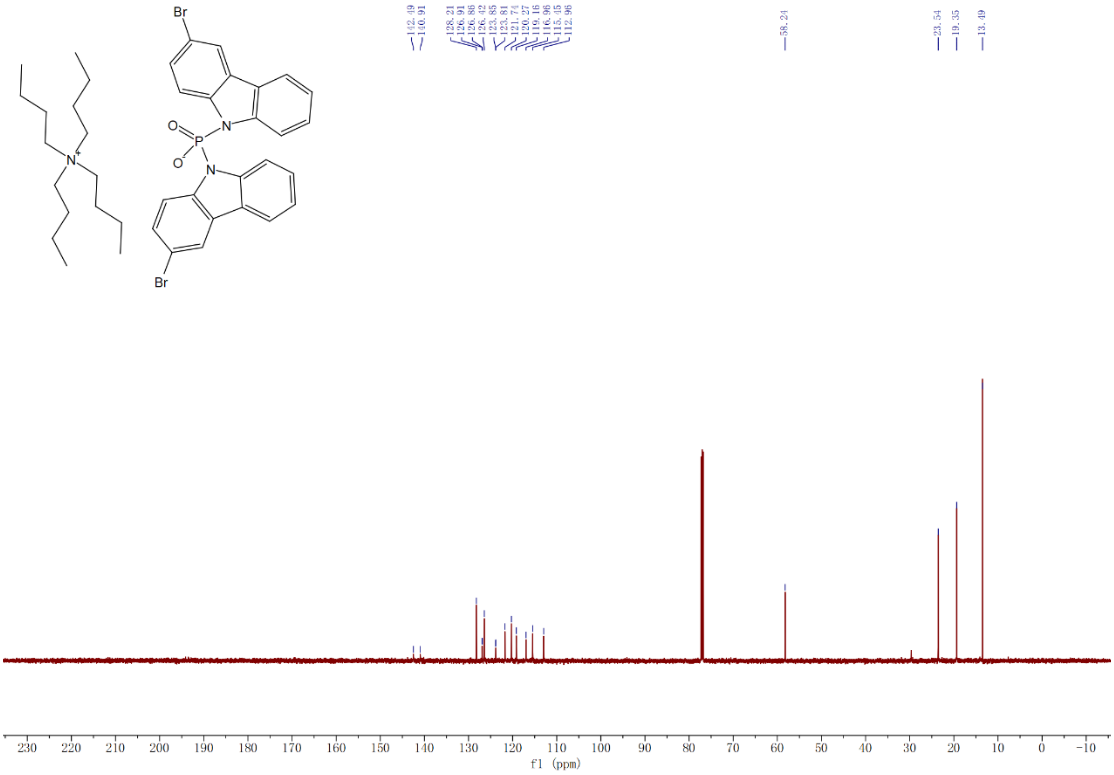

^{1}H NMR (400 MHz, CDCl$_3$)

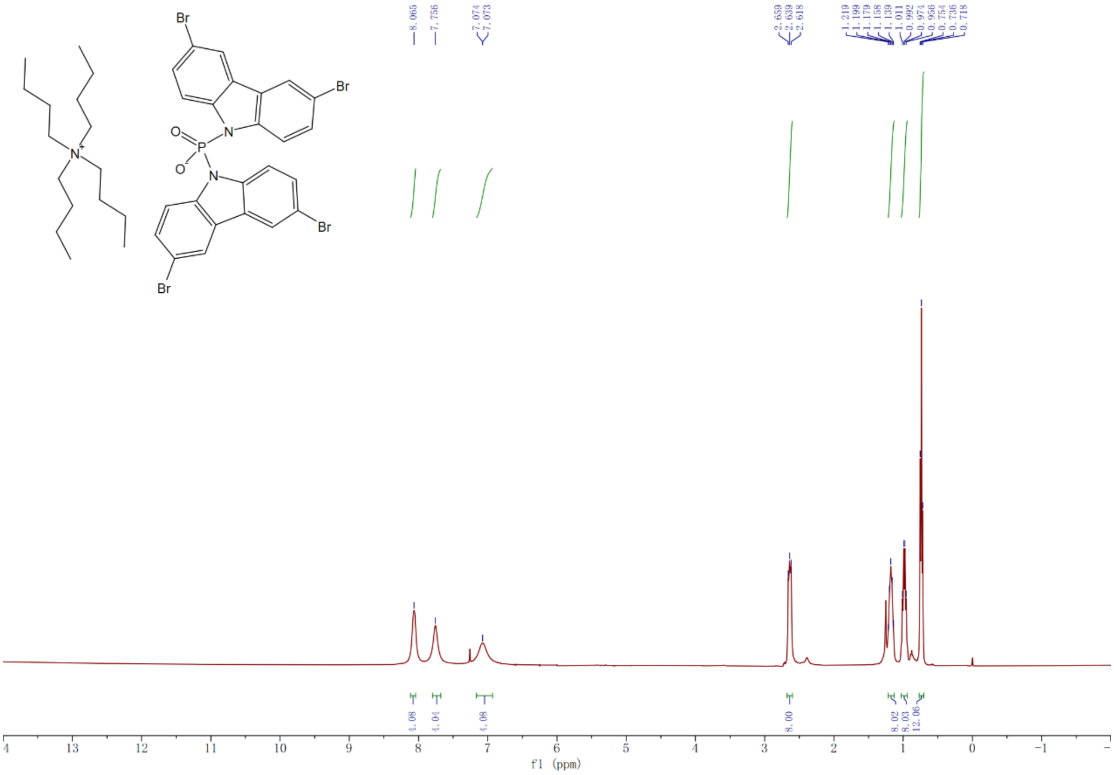

^{31}P NMR (400 MHz, $CDCl_3$)

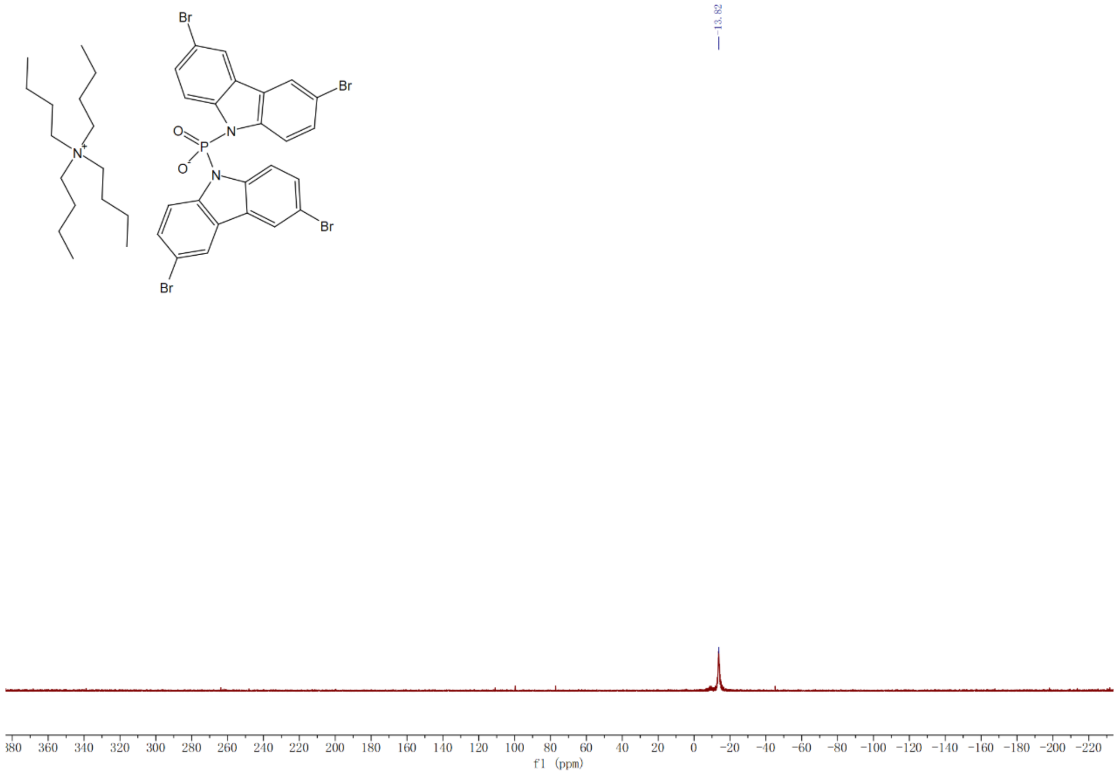

^{13}C NMR (600 MHz, $CDCl_3$)

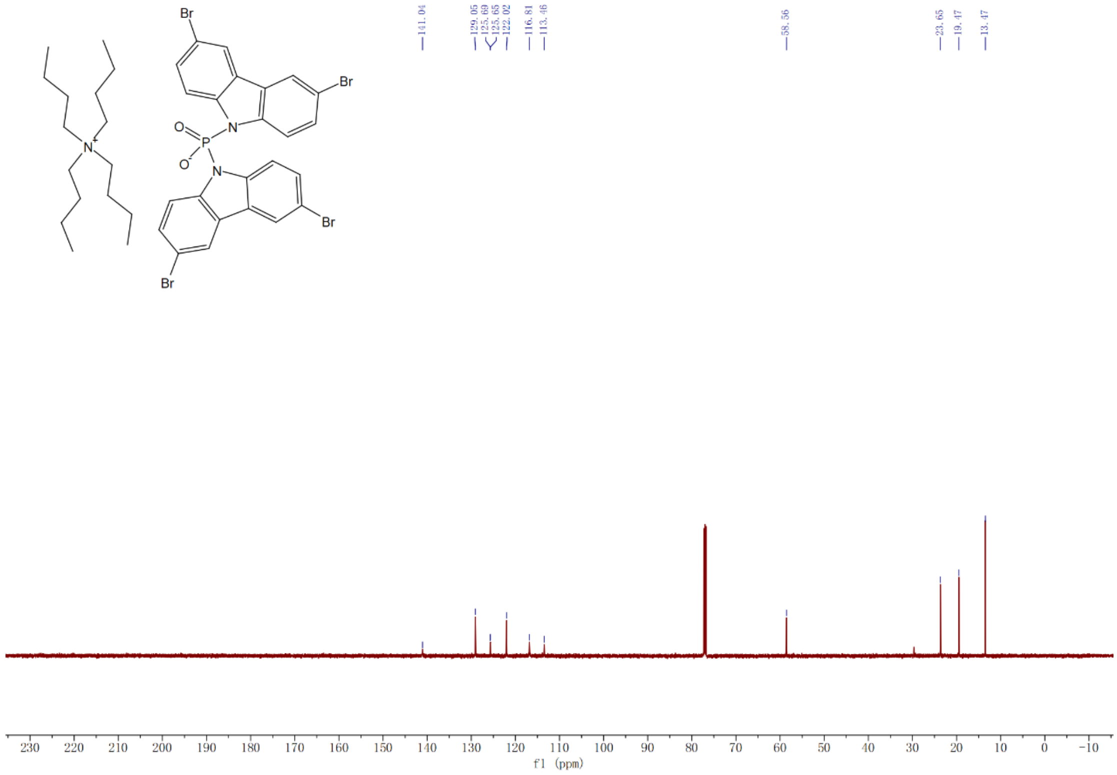

^{1}H NMR (400 MHz, $CDCl_3$)

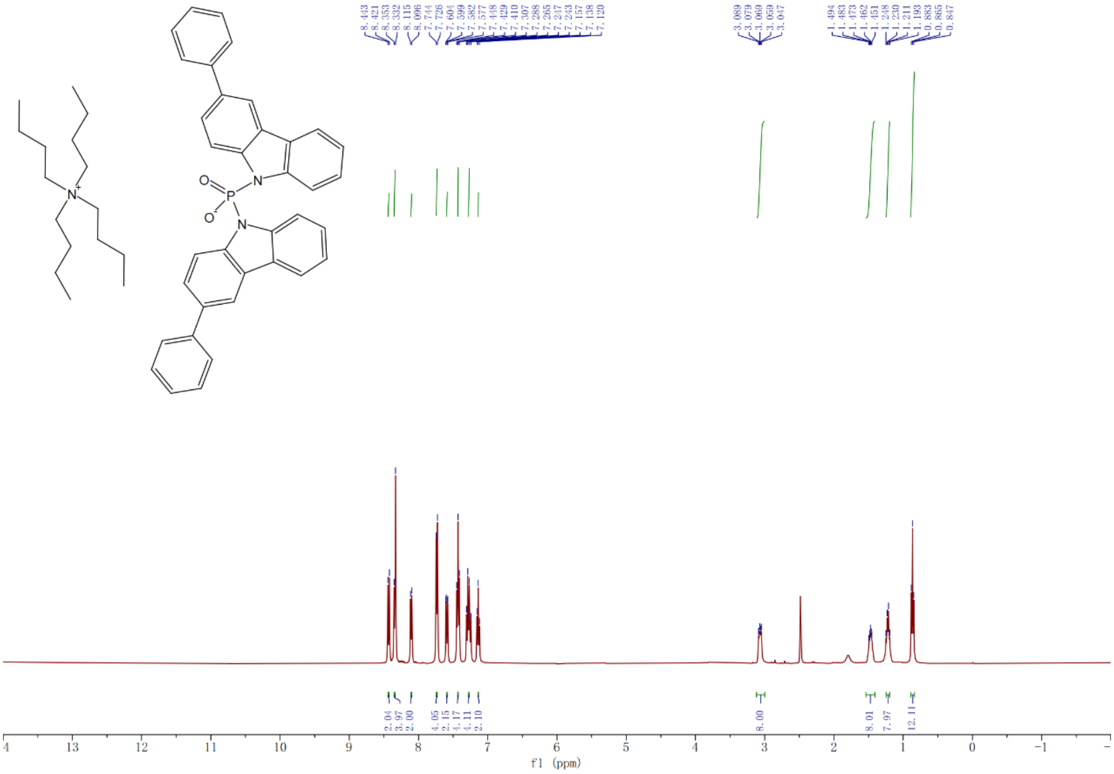

^{31}P NMR (400 MHz, $CDCl_3$)

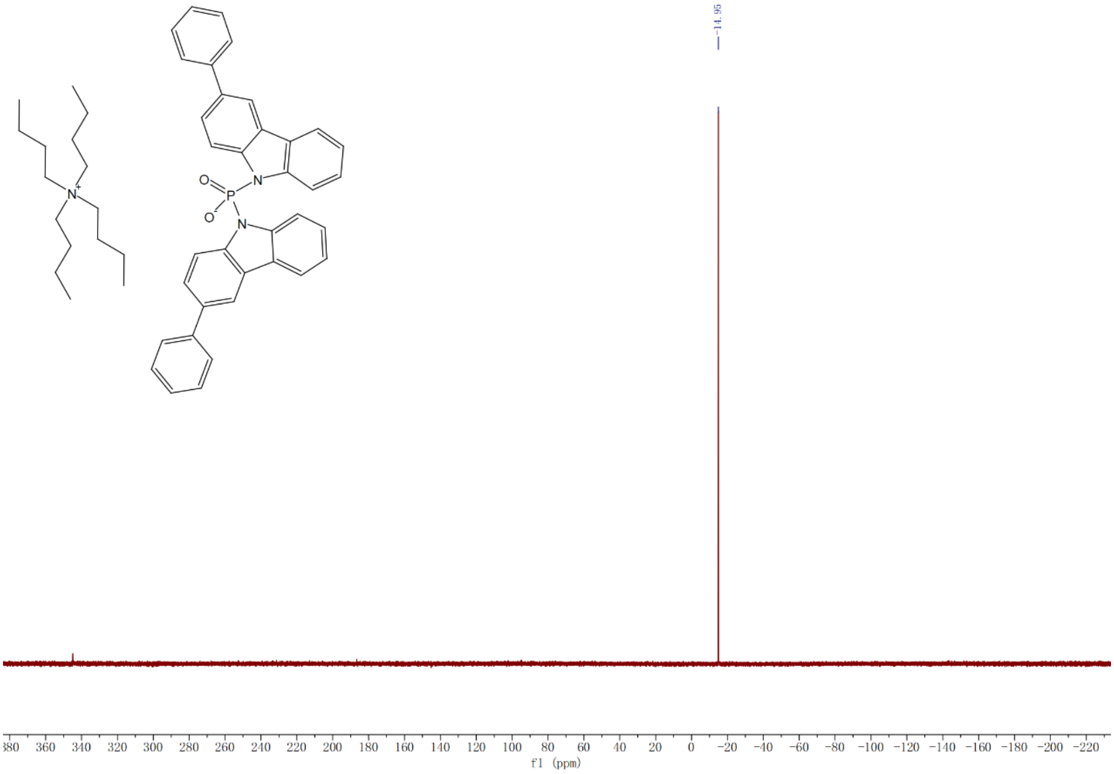

^{13}C NMR (600 MHz, $CDCl_3$)

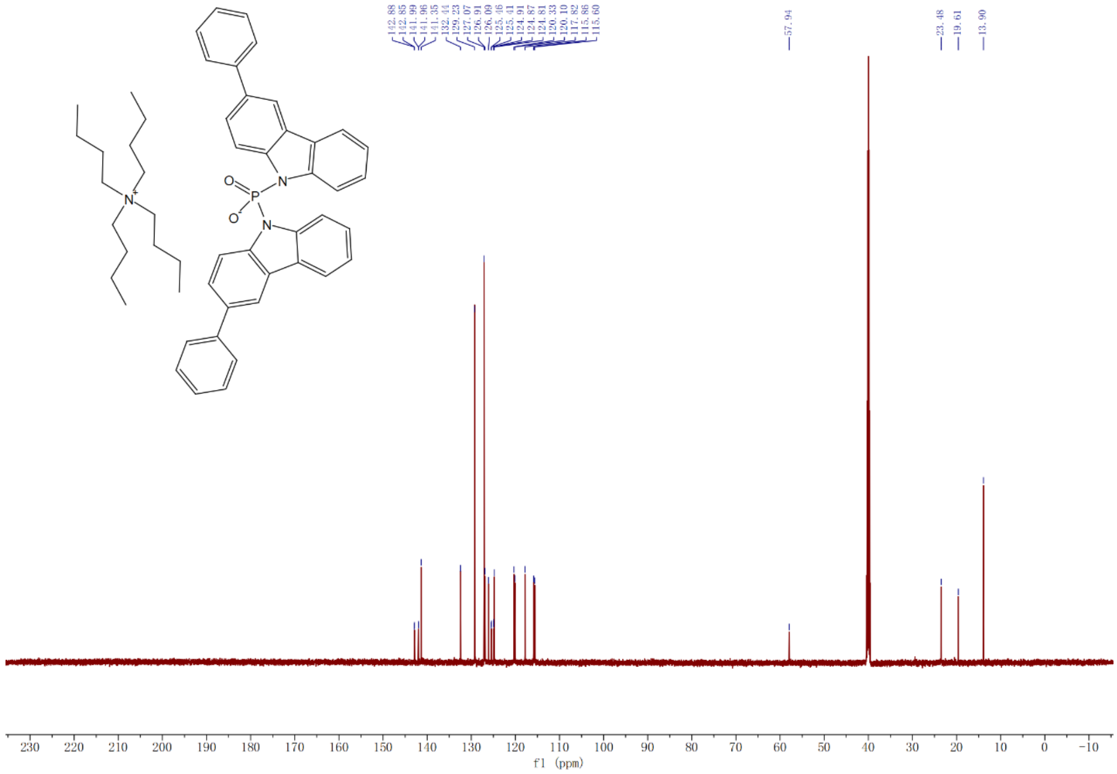

^{1}H NMR (400 MHz, $CDCl_3$)

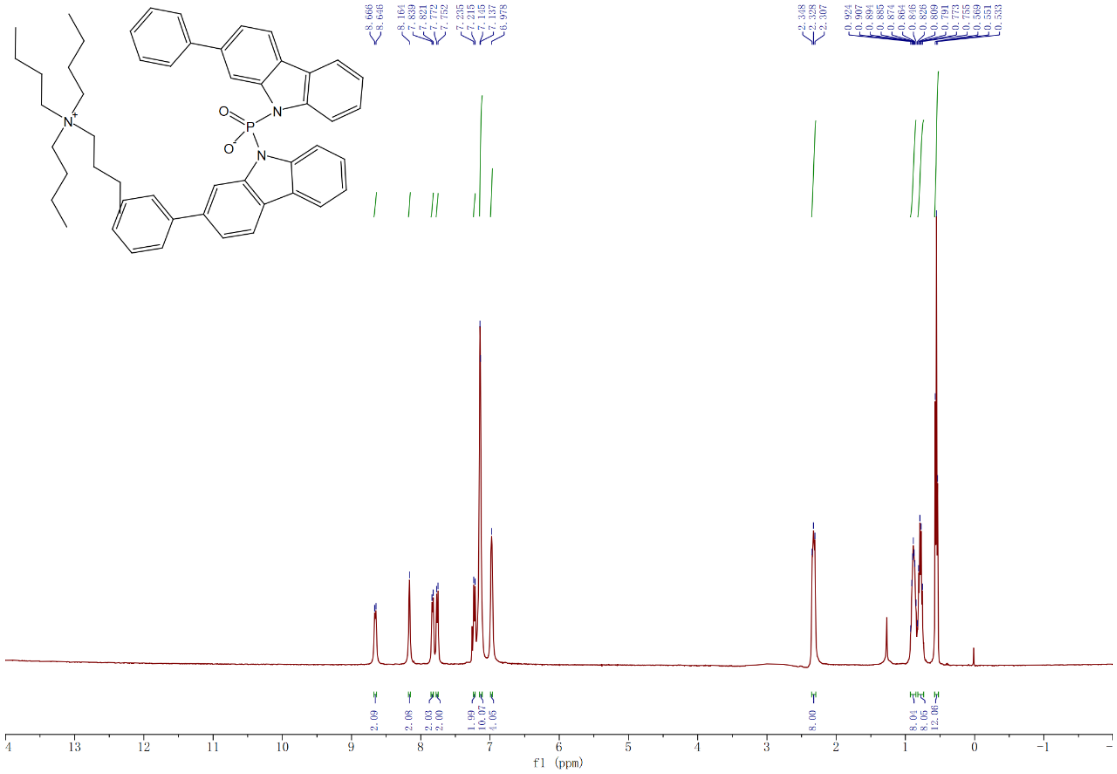

^{31}P NMR (400 MHz, CDCl$_3$)

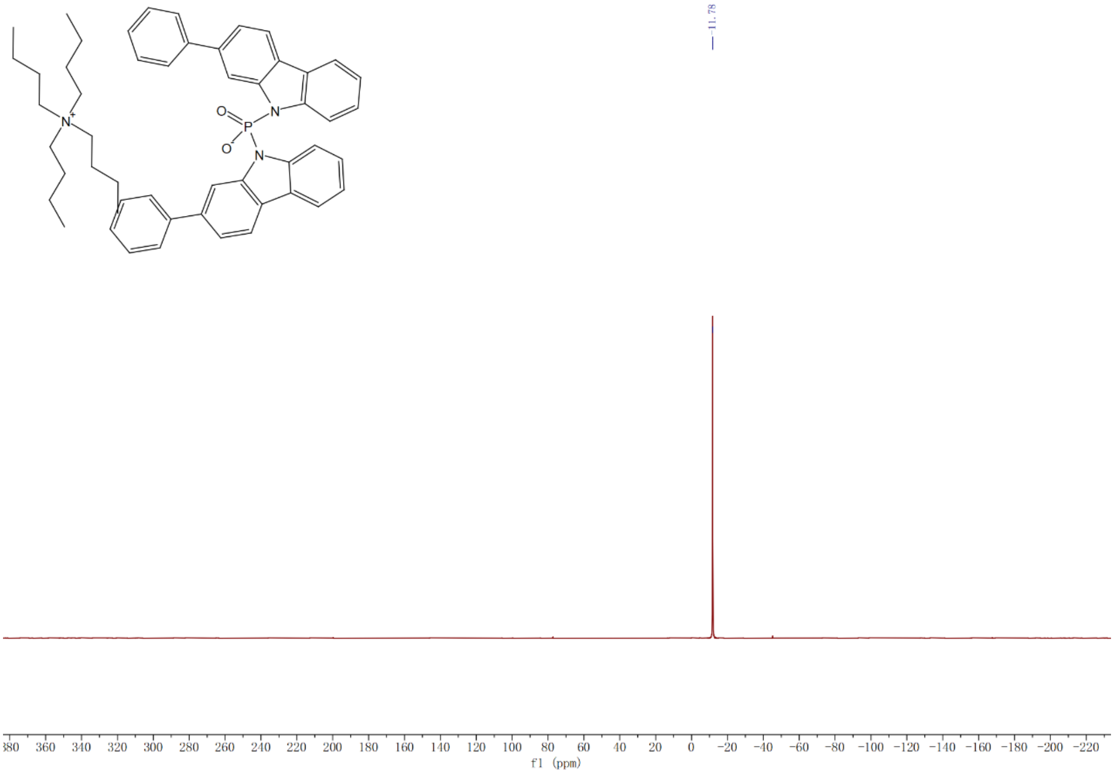

^{13}C NMR (600 MHz, CDCl$_3$)

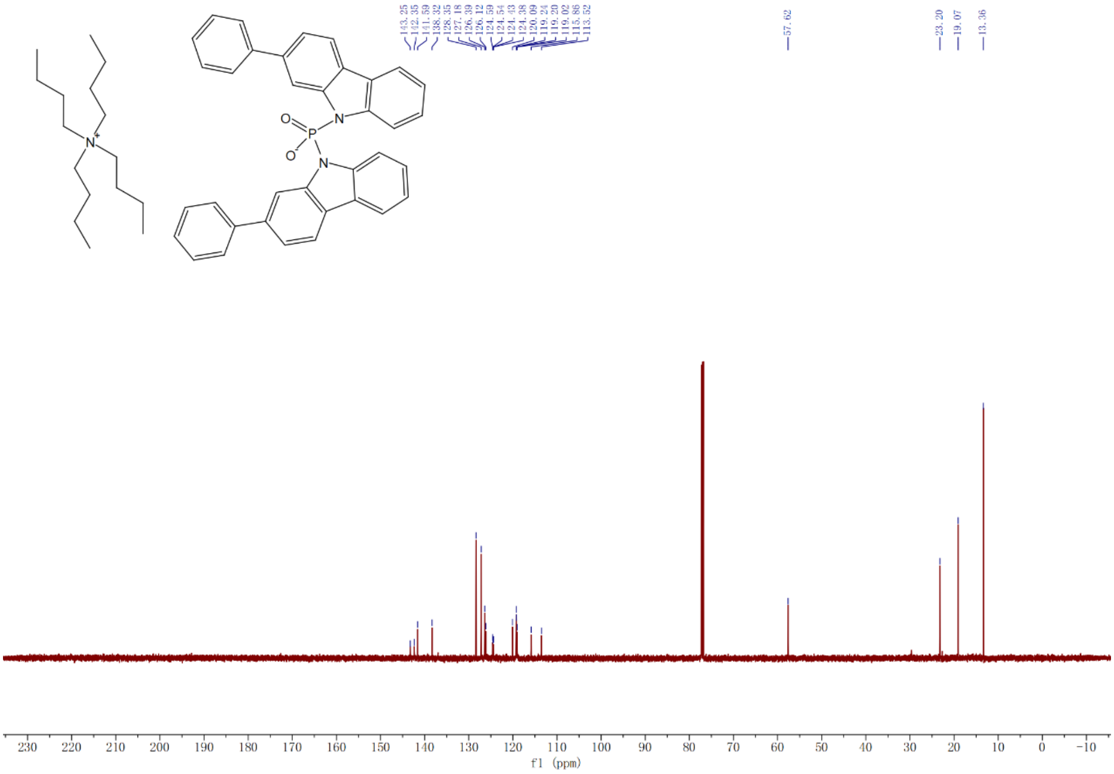

^{1}H NMR (400 MHz, $CDCl_3$) [3]

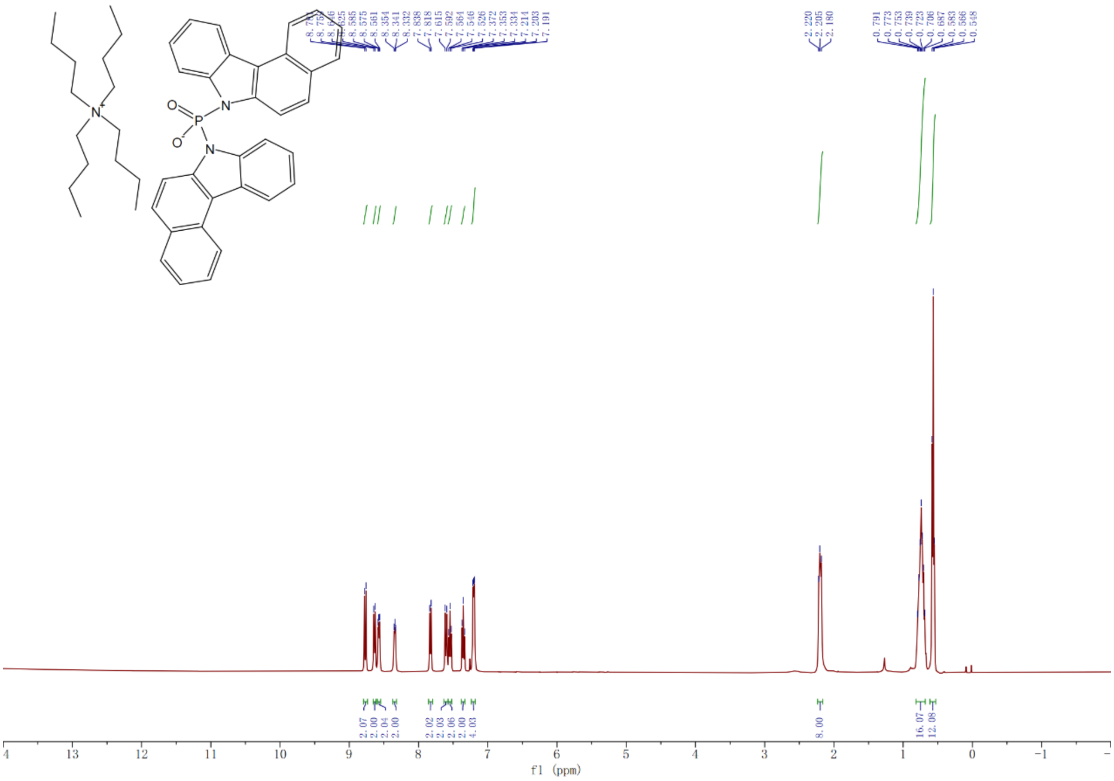

^{31}P NMR (400 MHz, $CDCl_3$)

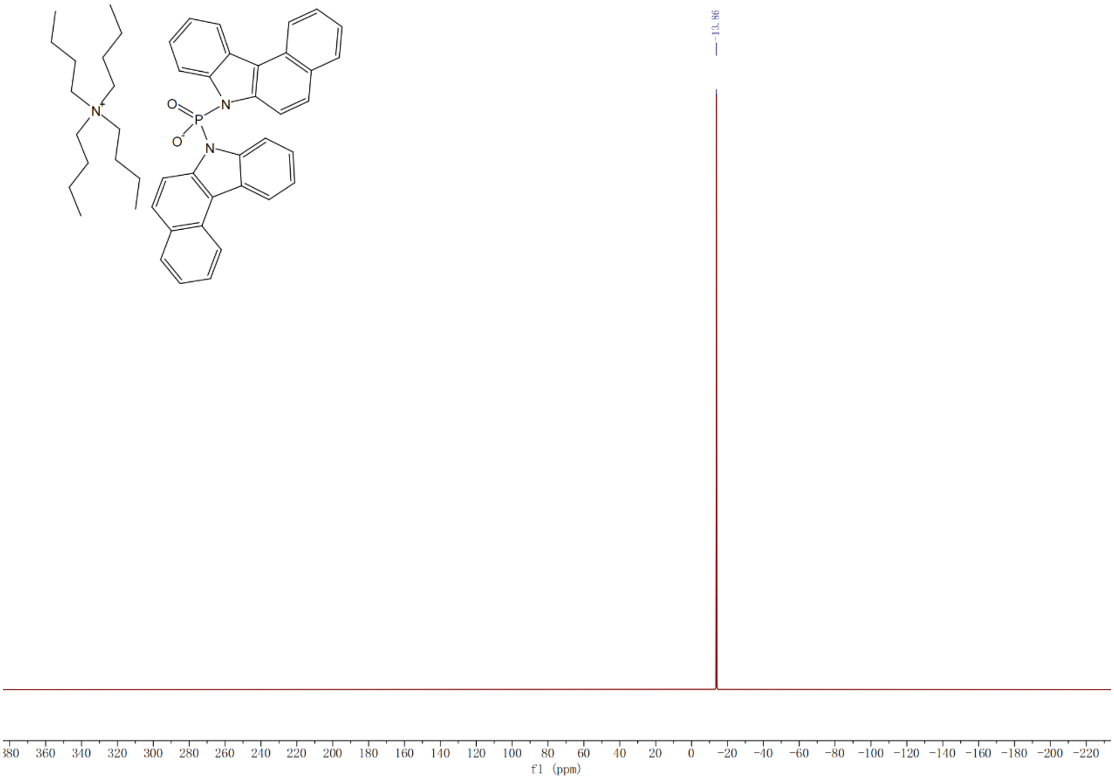

^{13}C NMR (600 MHz, $CDCl_3$)

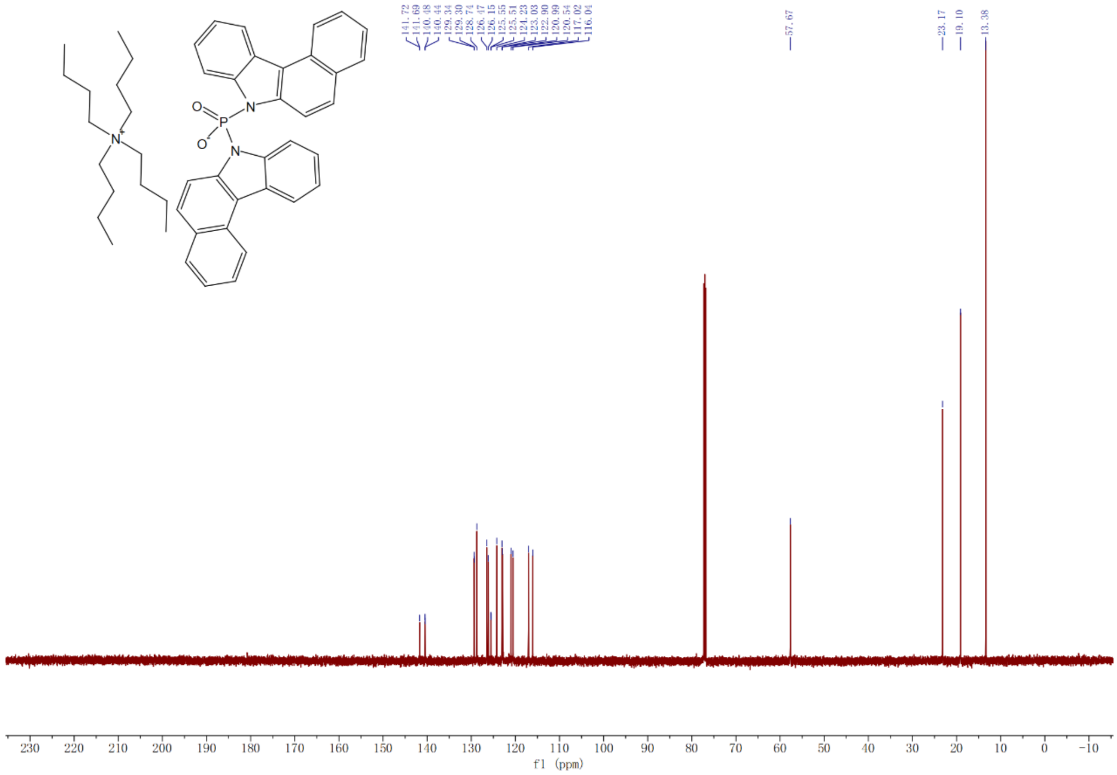

^{1}H NMR (400 MHz, $CDCl_3$)

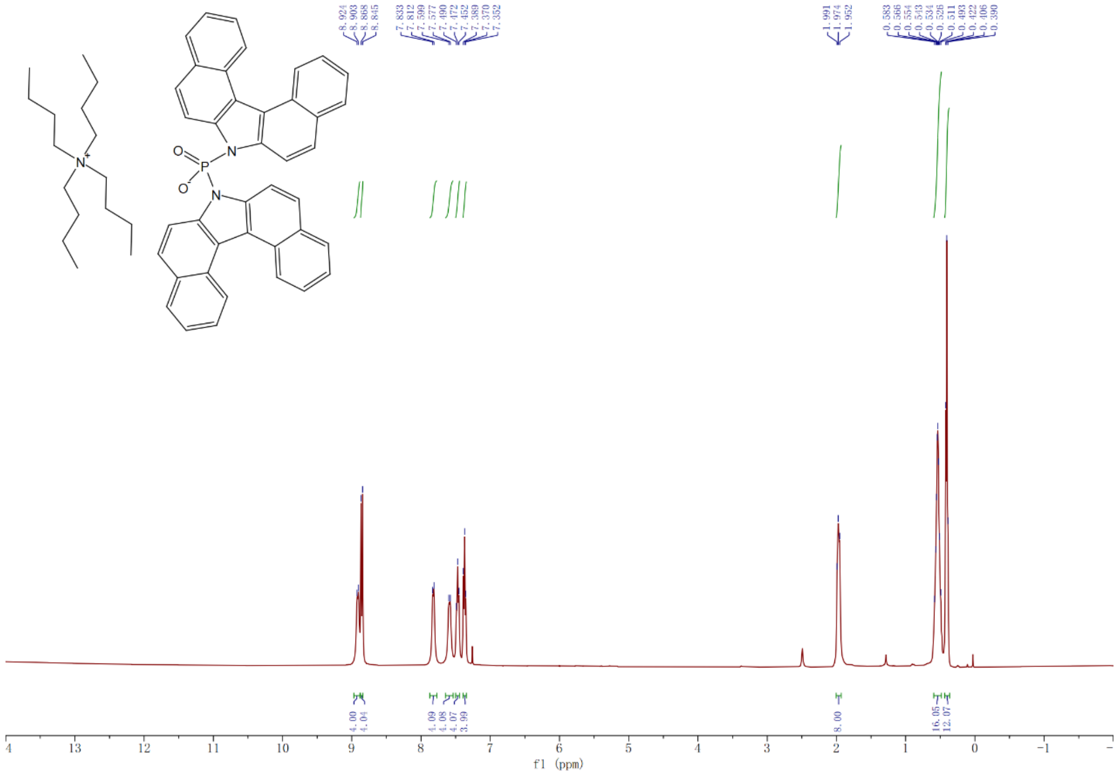

^{31}P NMR (400 MHz, CDCl$_3$)

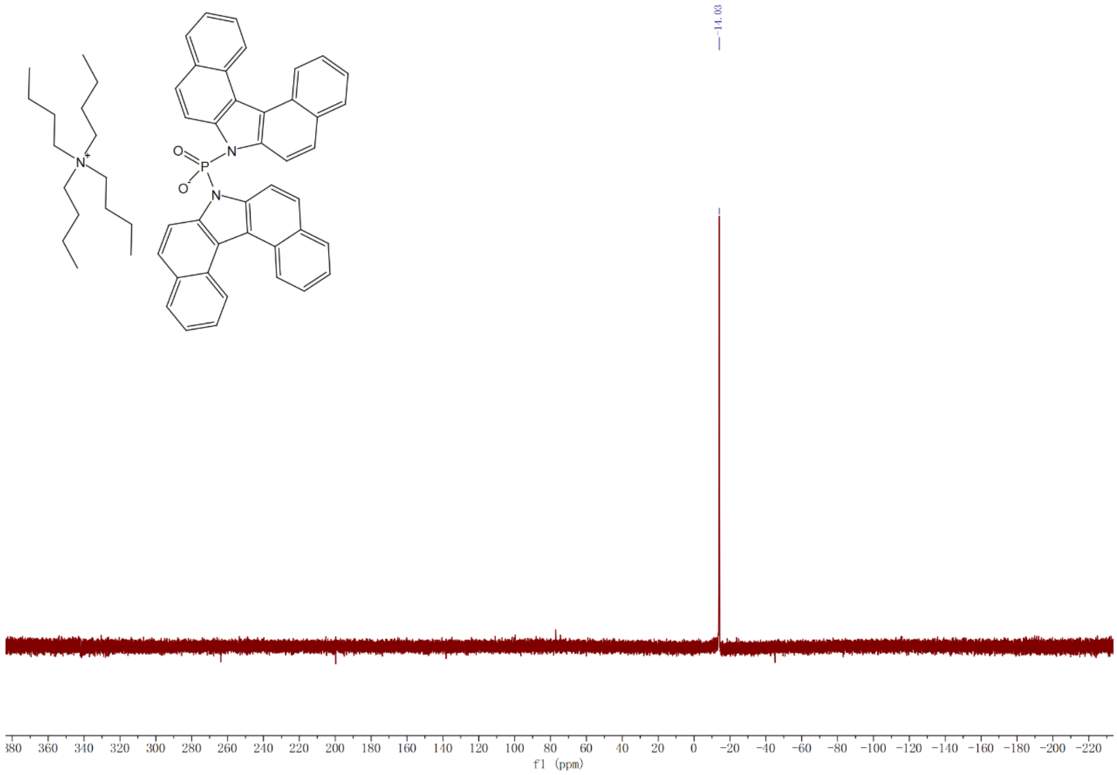

^{13}C NMR (600 MHz, CDCl$_3$)

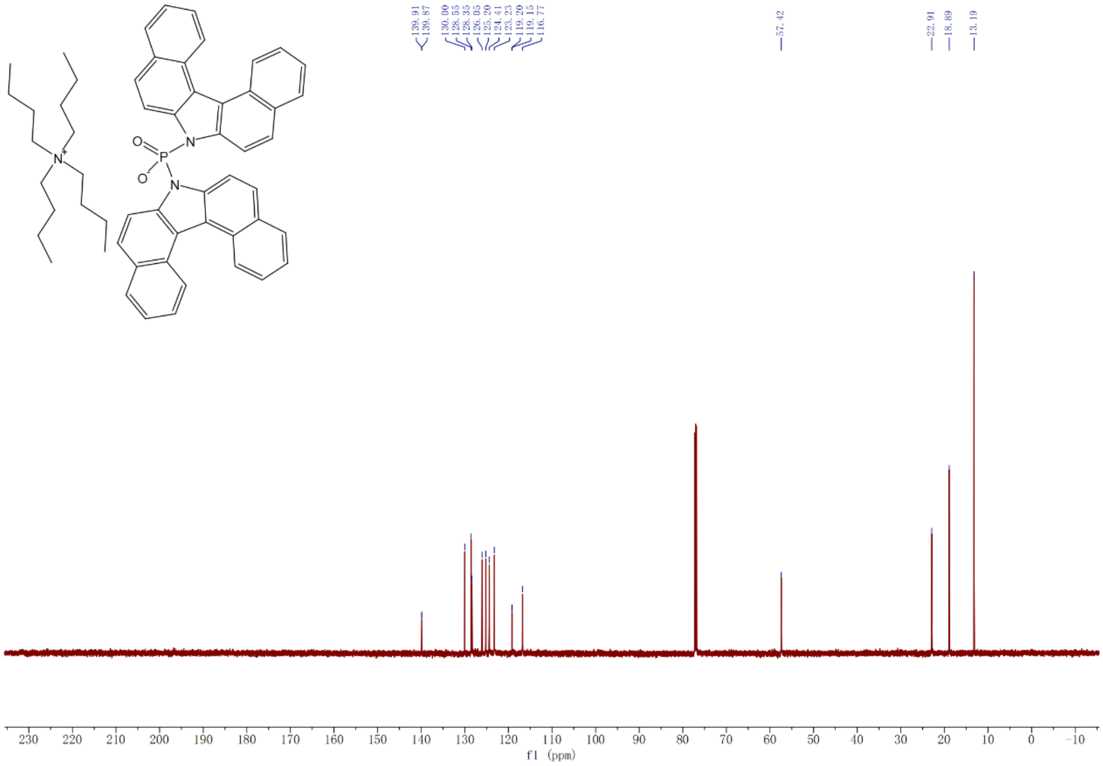

^{1}H NMR (400 MHz, $CDCl_3$)

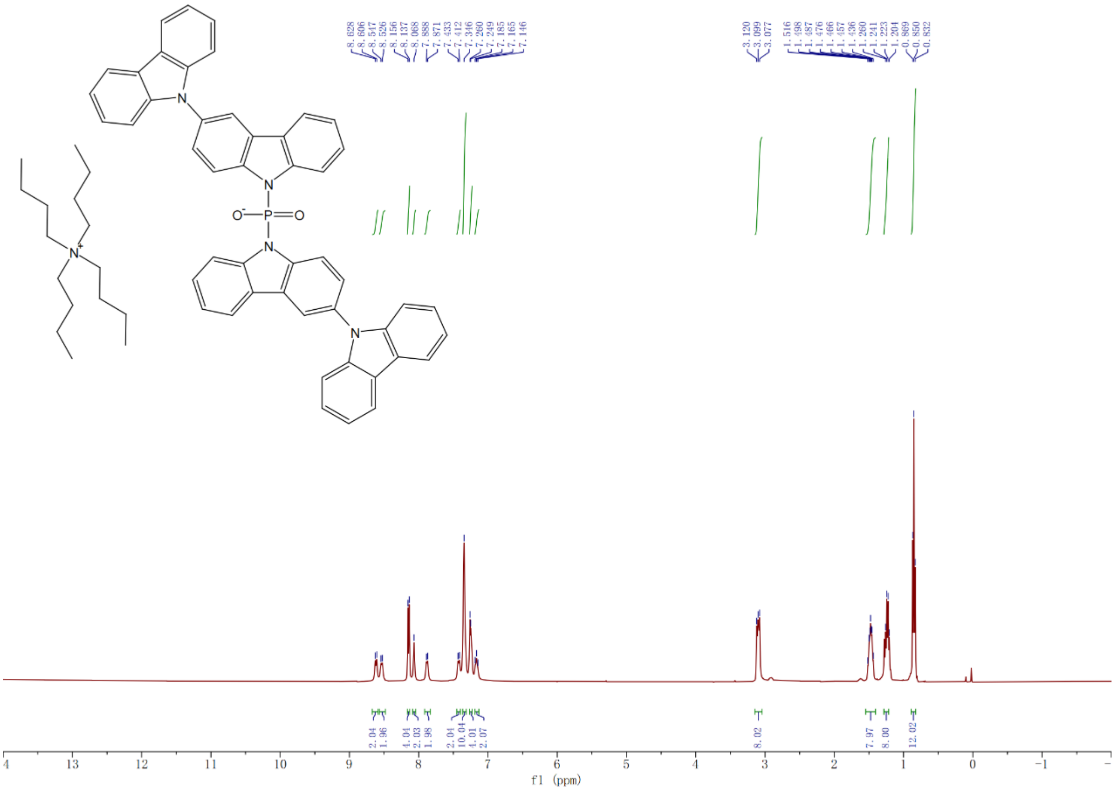

^{31}P NMR (400 MHz, $CDCl_3$)

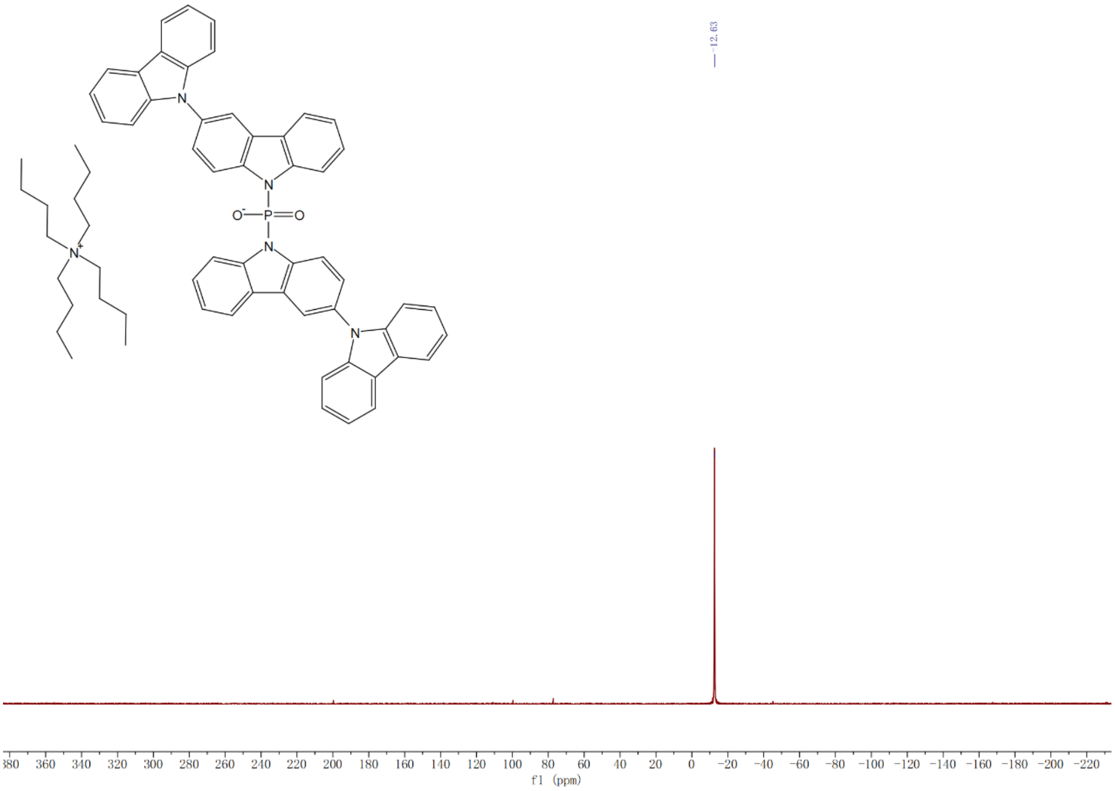

^{13}C NMR (600 MHz, $CDCl_3$)

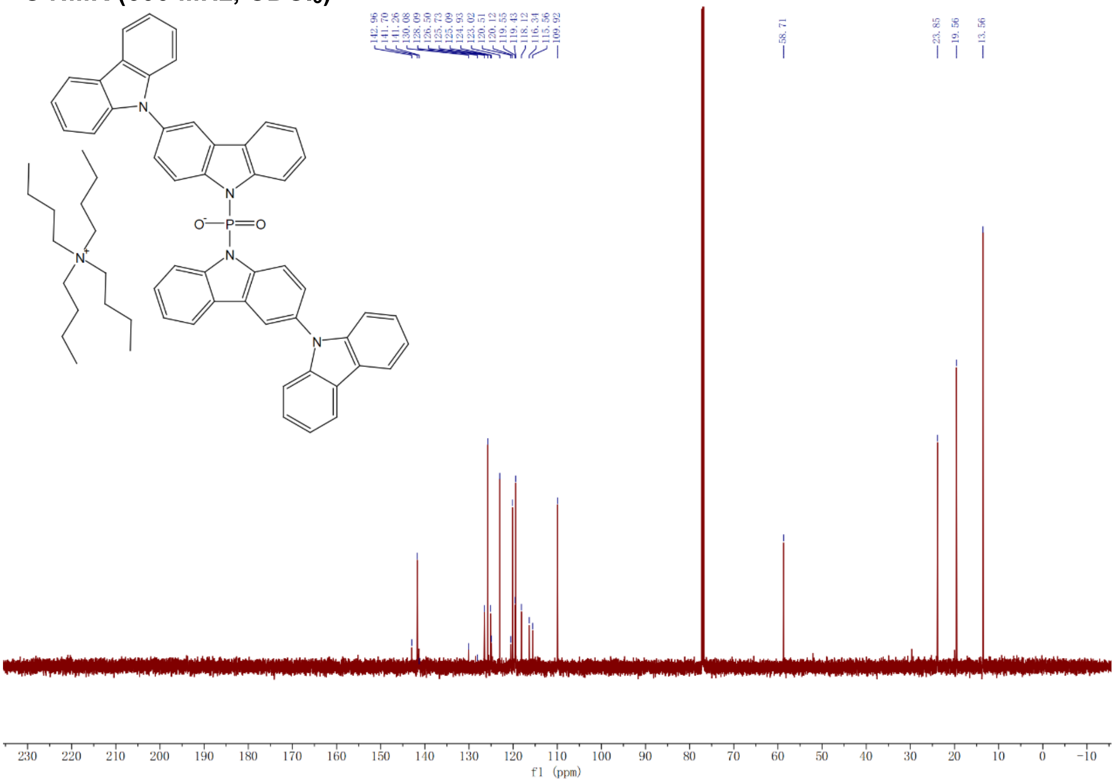

^{1}H NMR (400 MHz, $CDCl_3$)

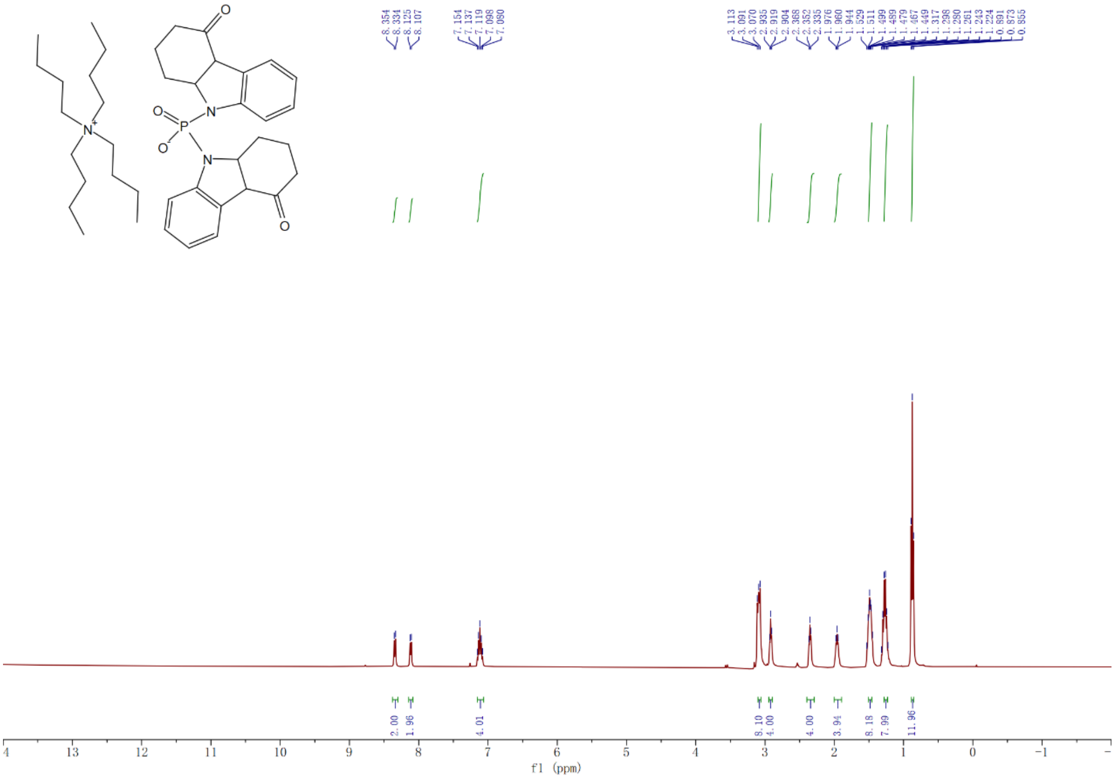

^{31}P NMR (400 MHz, CDCl$_3$)

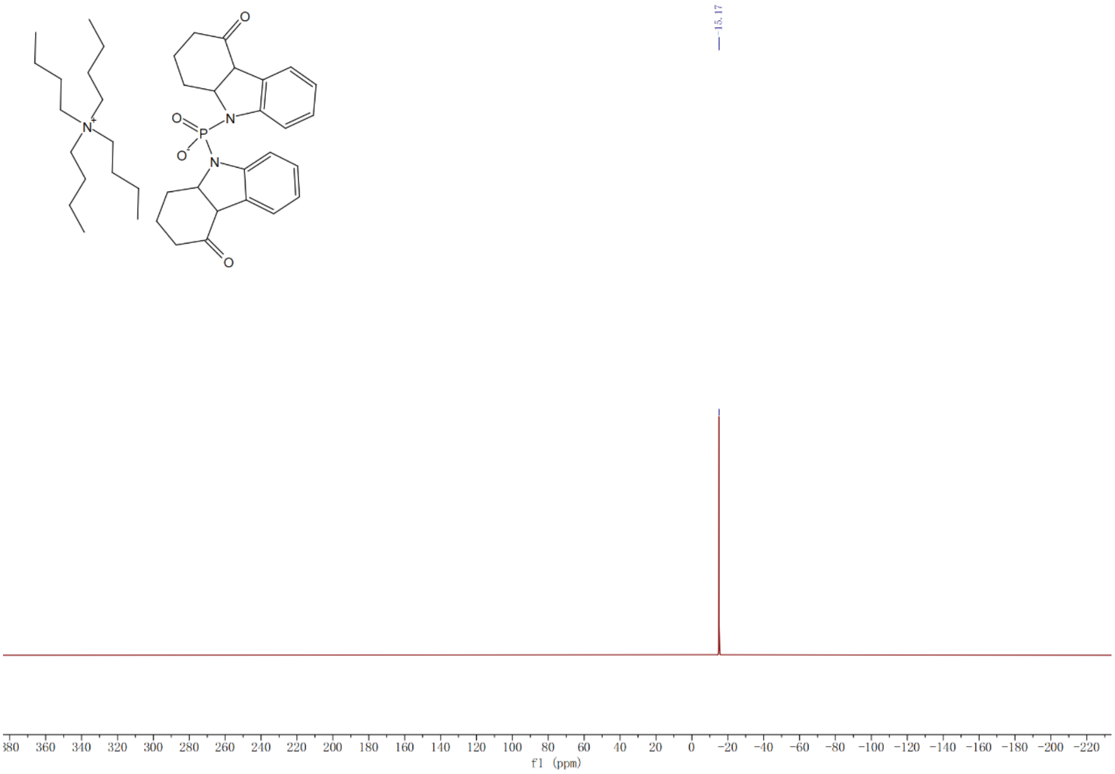

^{13}C NMR (600 MHz, CDCl$_3$)

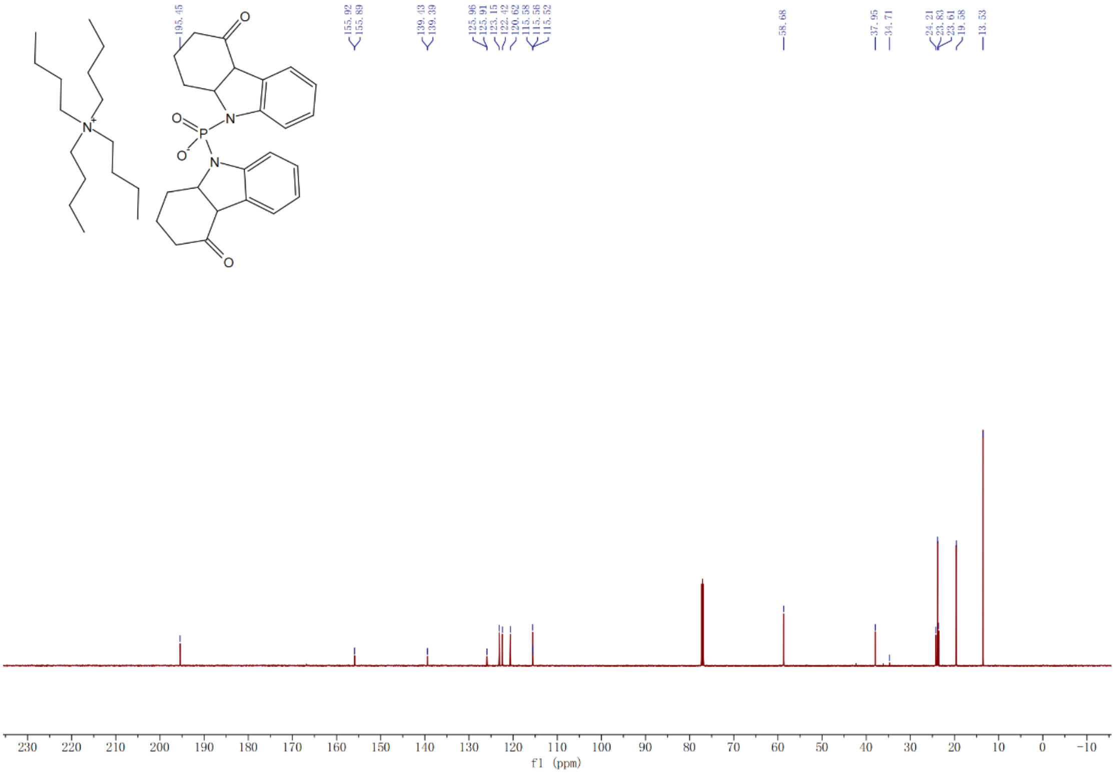

^{1}H NMR (400 MHz, $CDCl_3$)

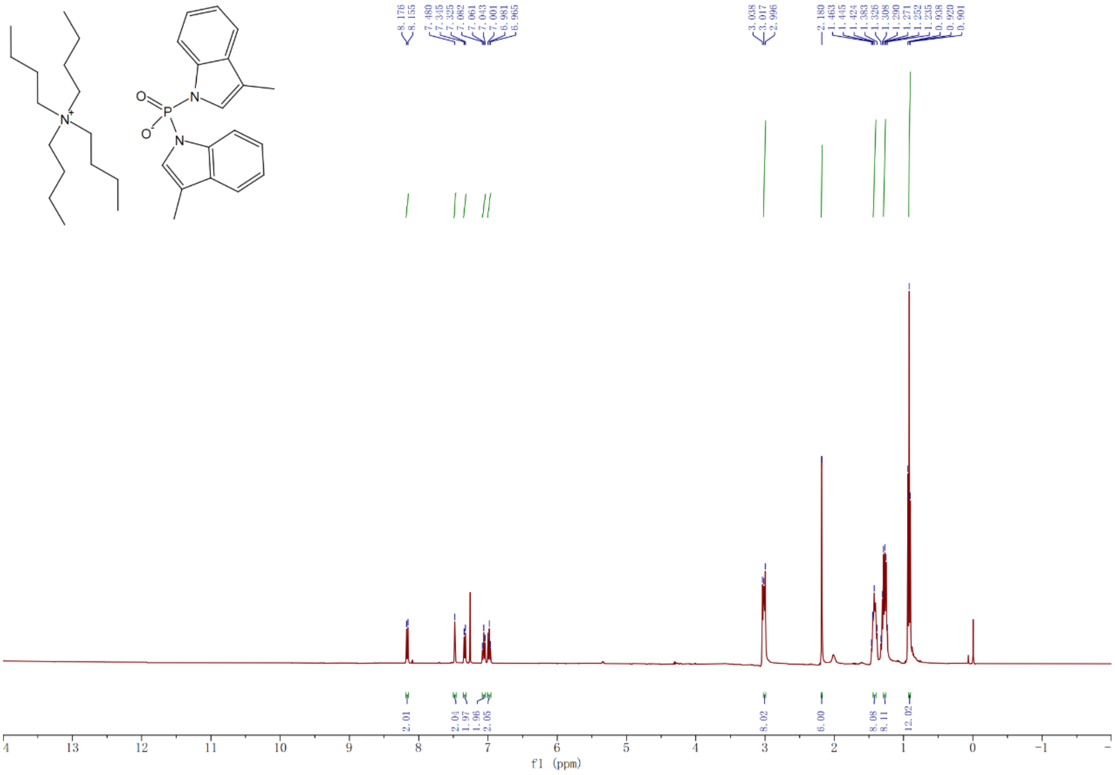

^{31}P NMR (400 MHz, $CDCl_3$)

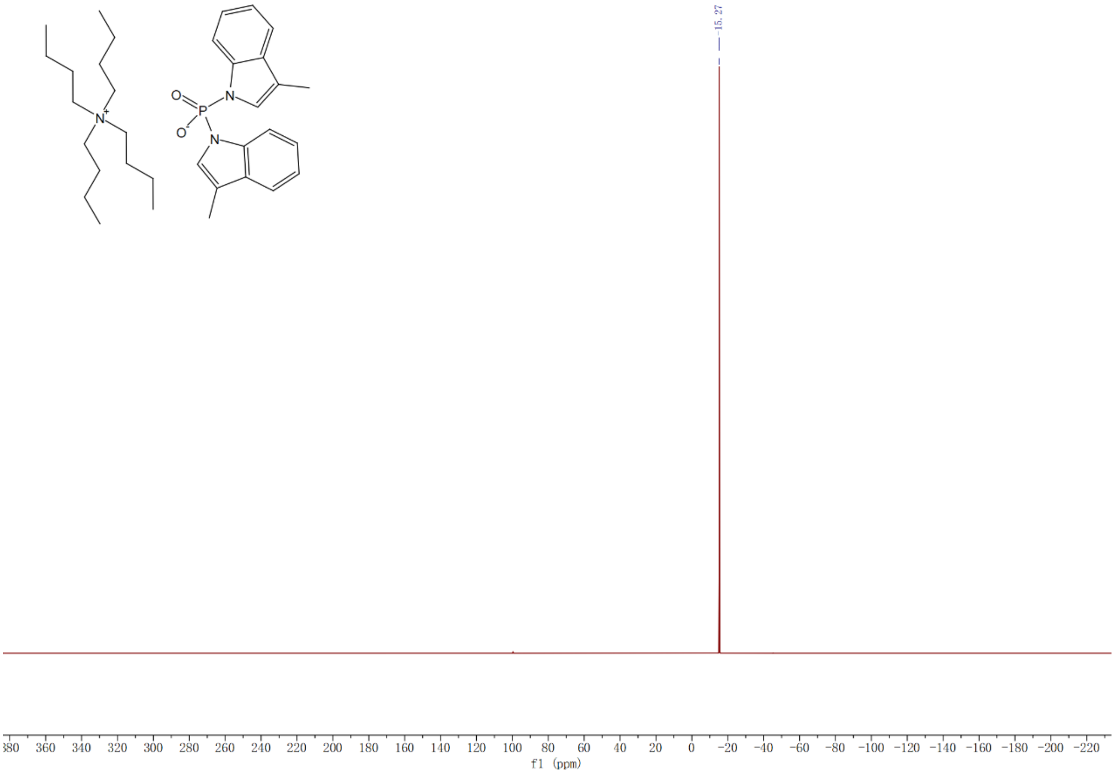

^{13}C NMR (600 MHz, $CDCl_3$)

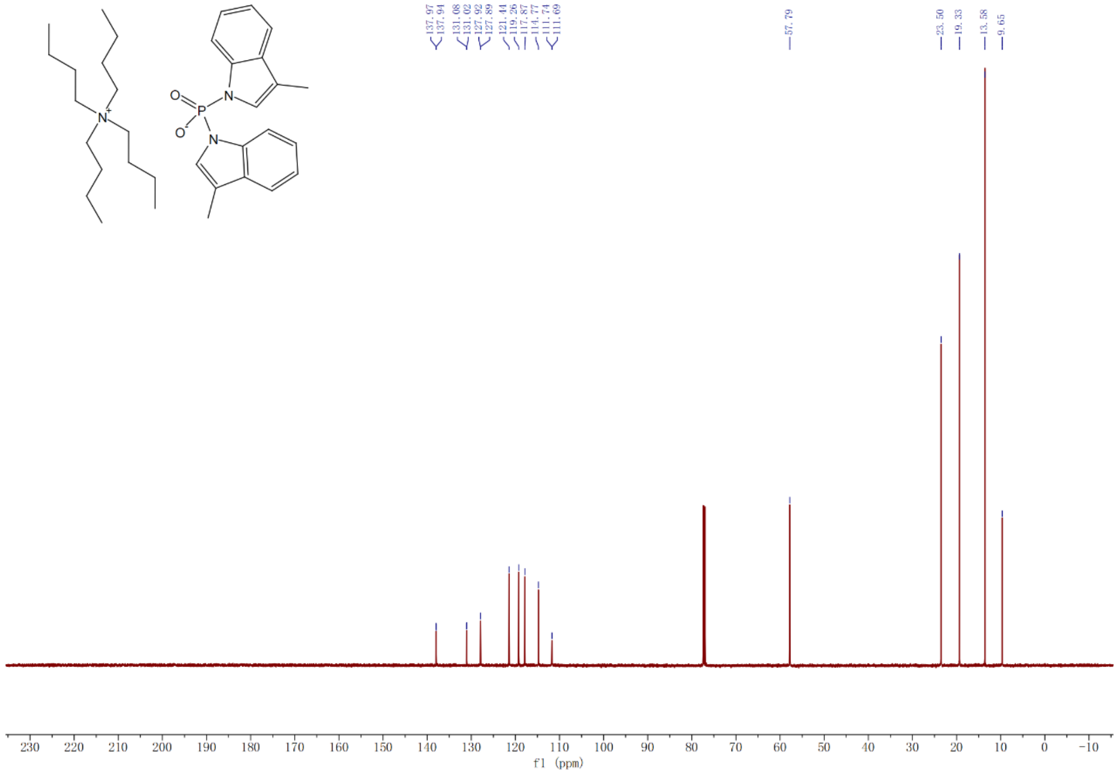

^{1}H NMR (400 MHz, DMSO-D_6)

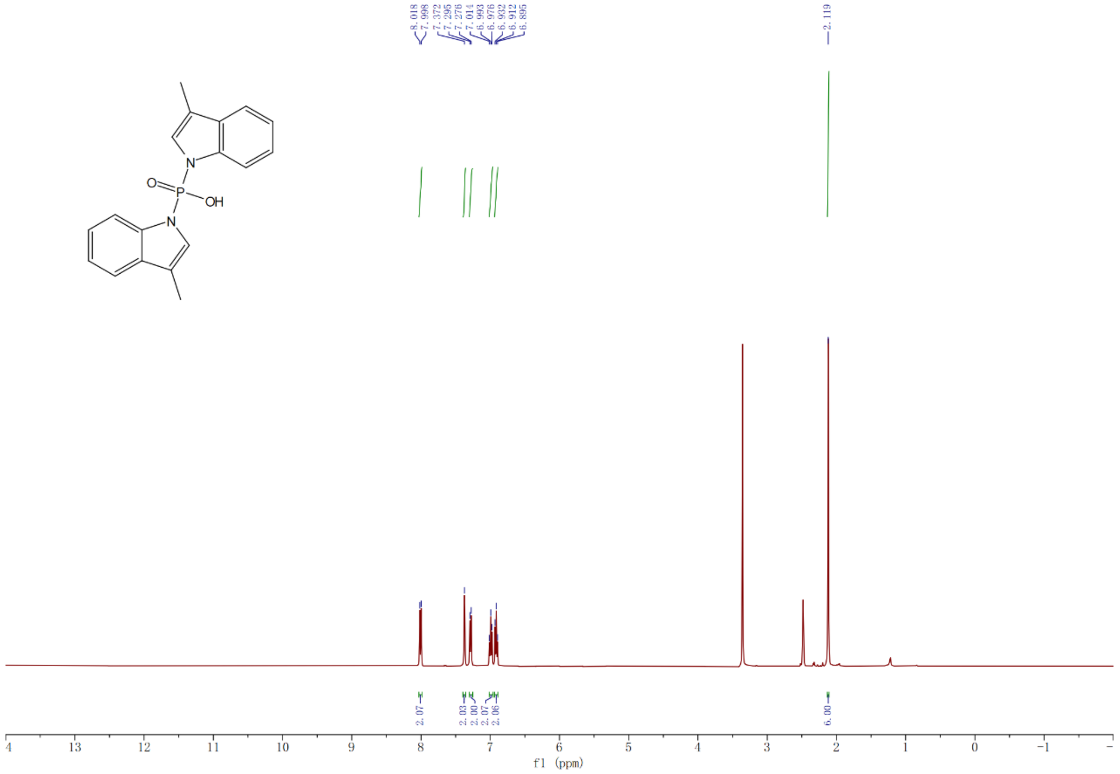

^{31}P NMR (400 MHz, DMSO-D$_6$)

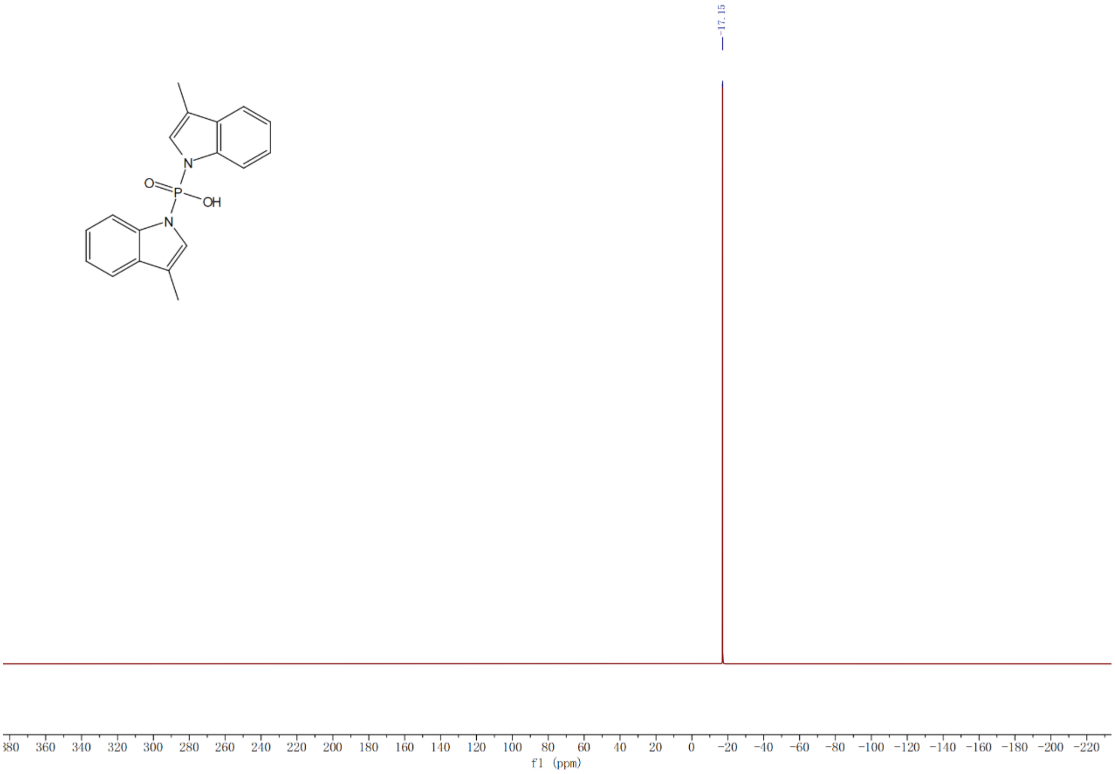

^{13}C NMR (600 MHz, DMSO-D$_6$)

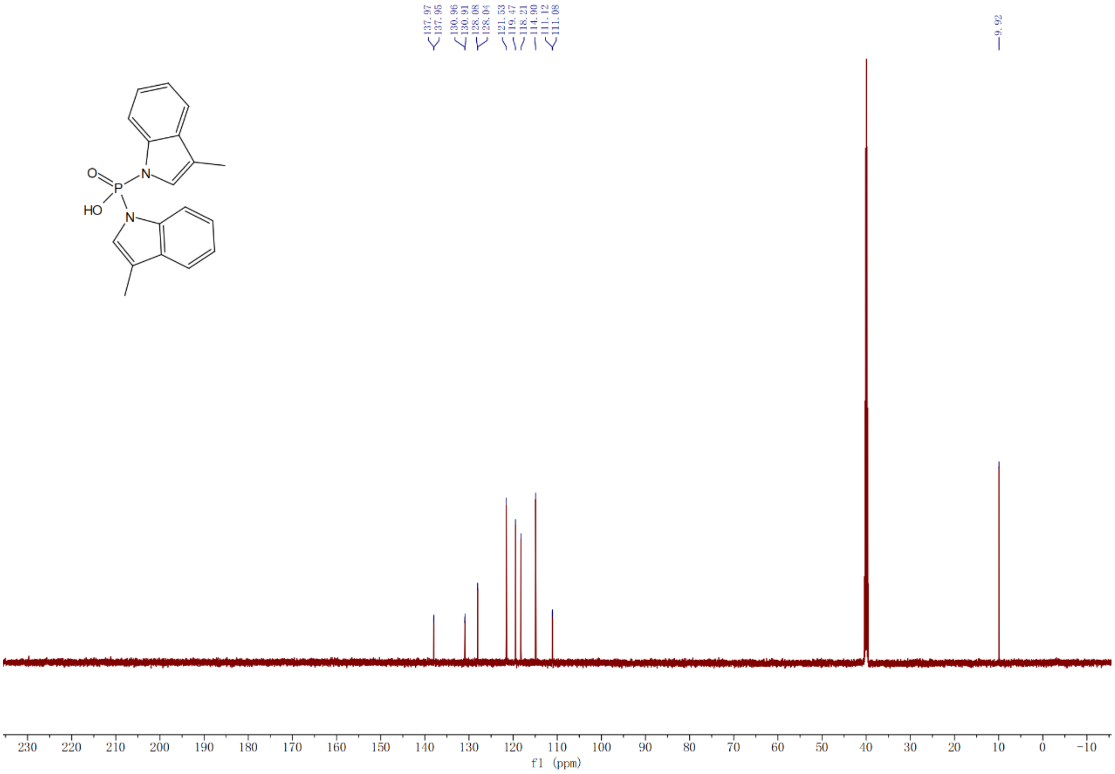

^{1}H NMR (400 MHz, $CDCl_3$)

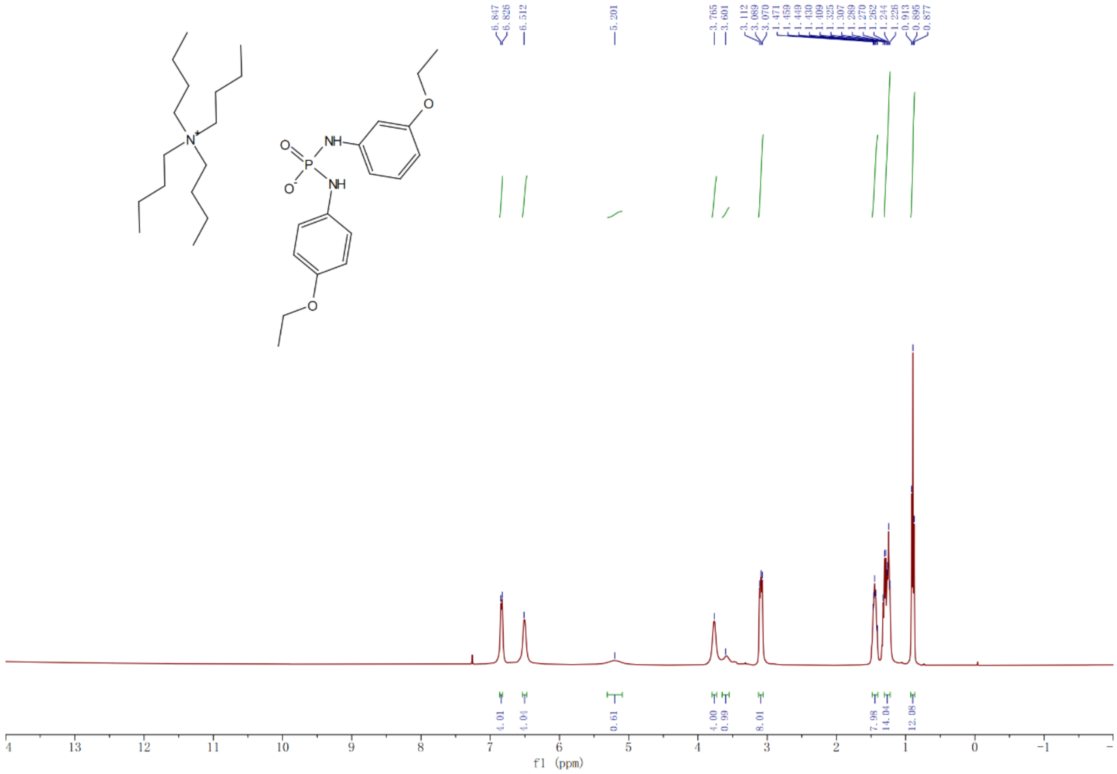

^{31}P NMR (400 MHz, $CDCl_3$)

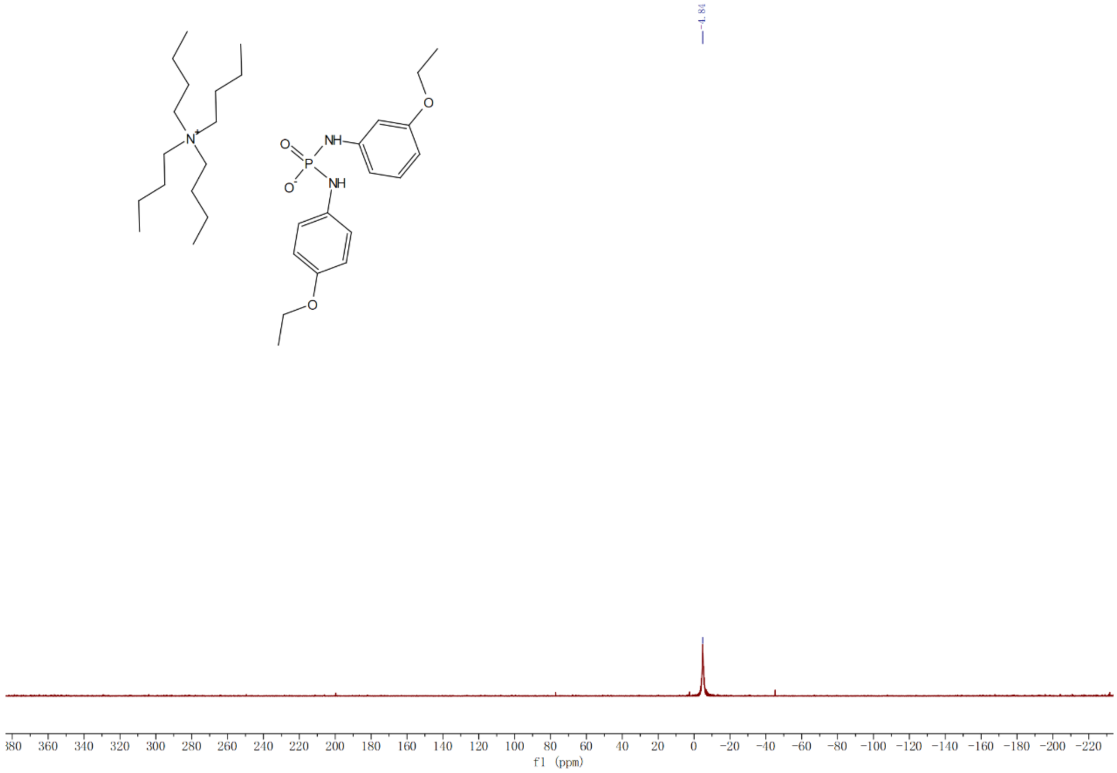

^{13}C NMR (600 MHz, $CDCl_3$)

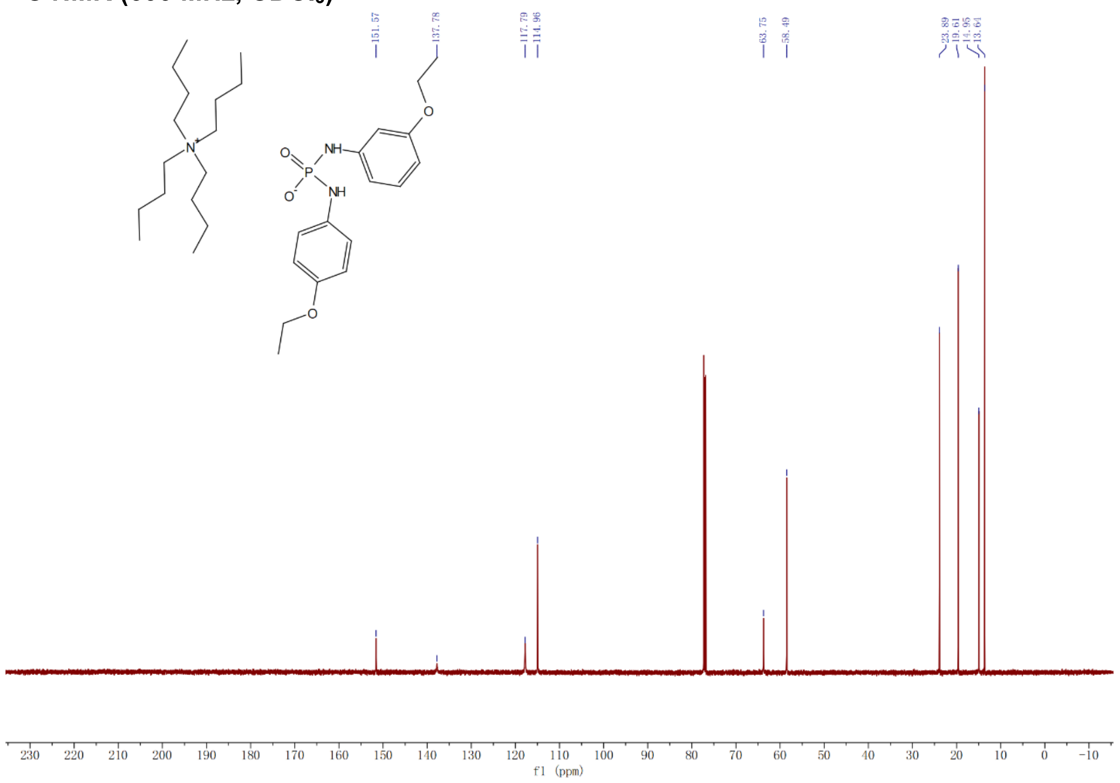

^{1}H NMR (400 MHz, DMSO-D_6)

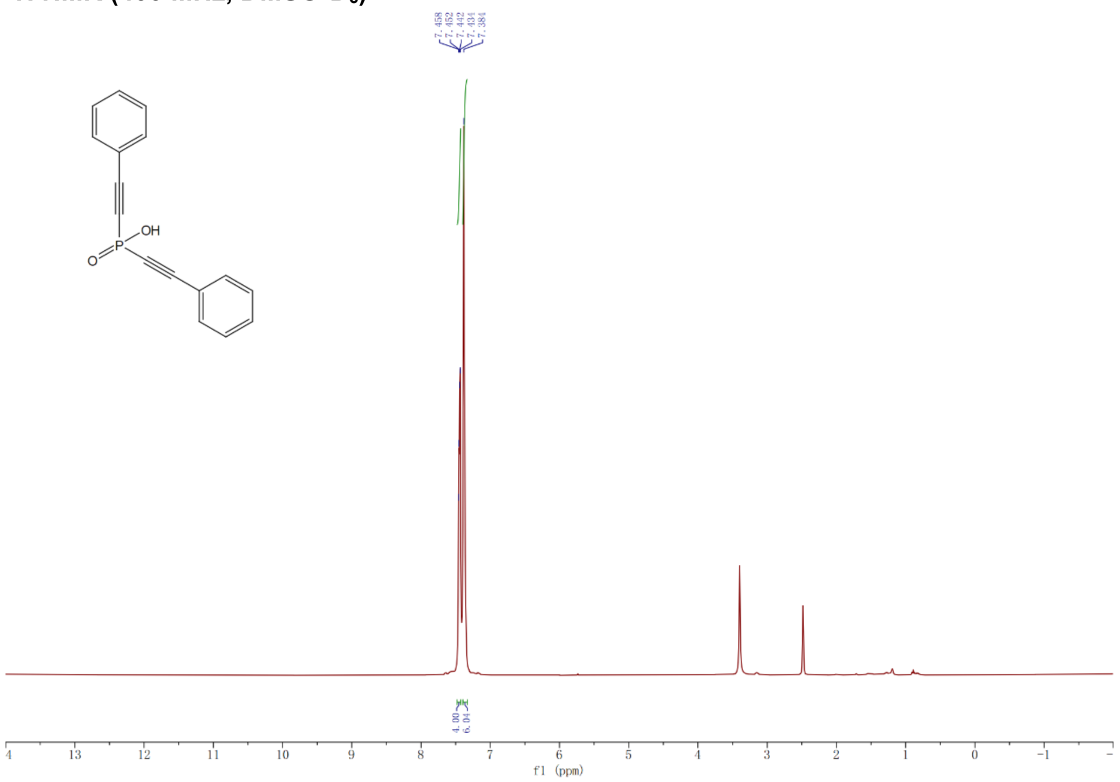

^{31}P NMR (400 MHz, DMSO-D$_6$)

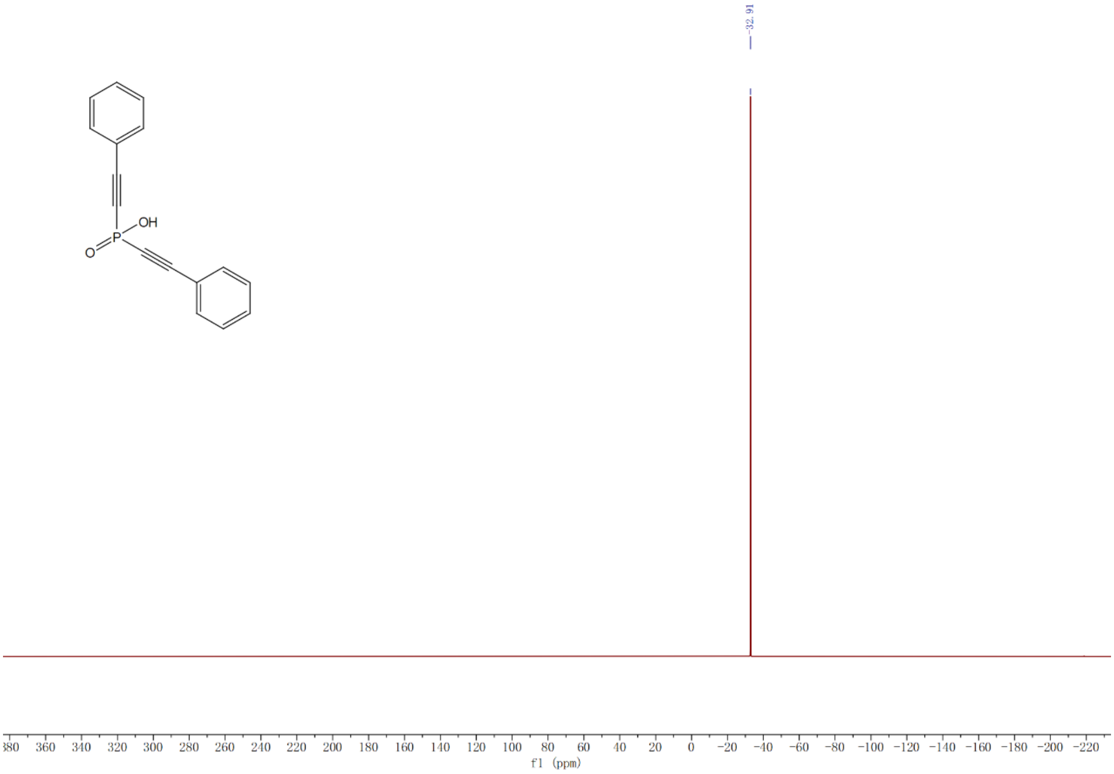

^{13}C NMR (600 MHz, DMSO-D$_6$)

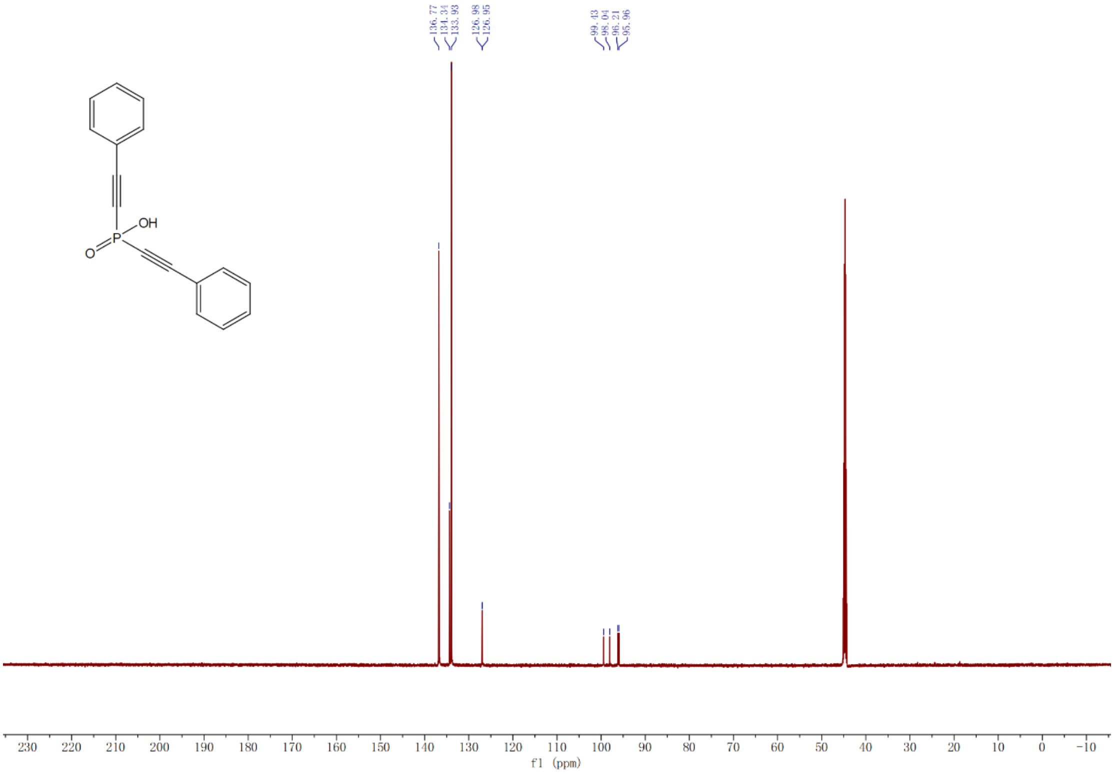

^{1}H NMR (400 MHz, DMSO-D$_6$)

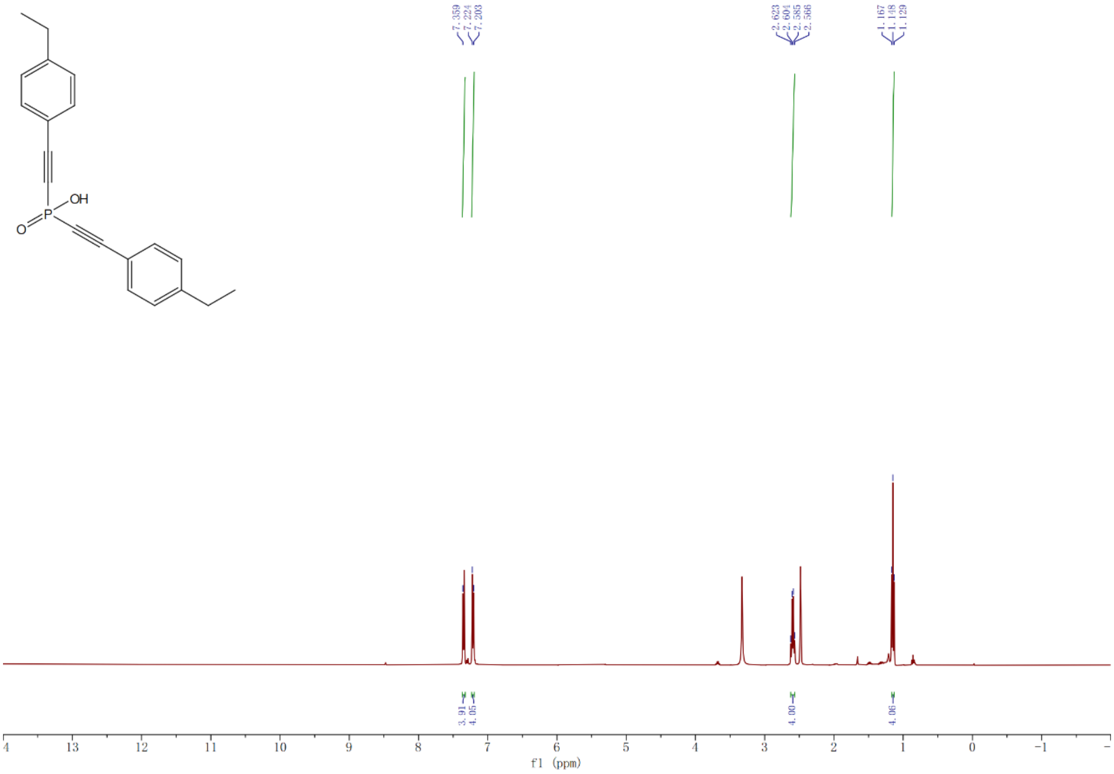

^{31}P NMR (400 MHz, DMSO-D$_6$)

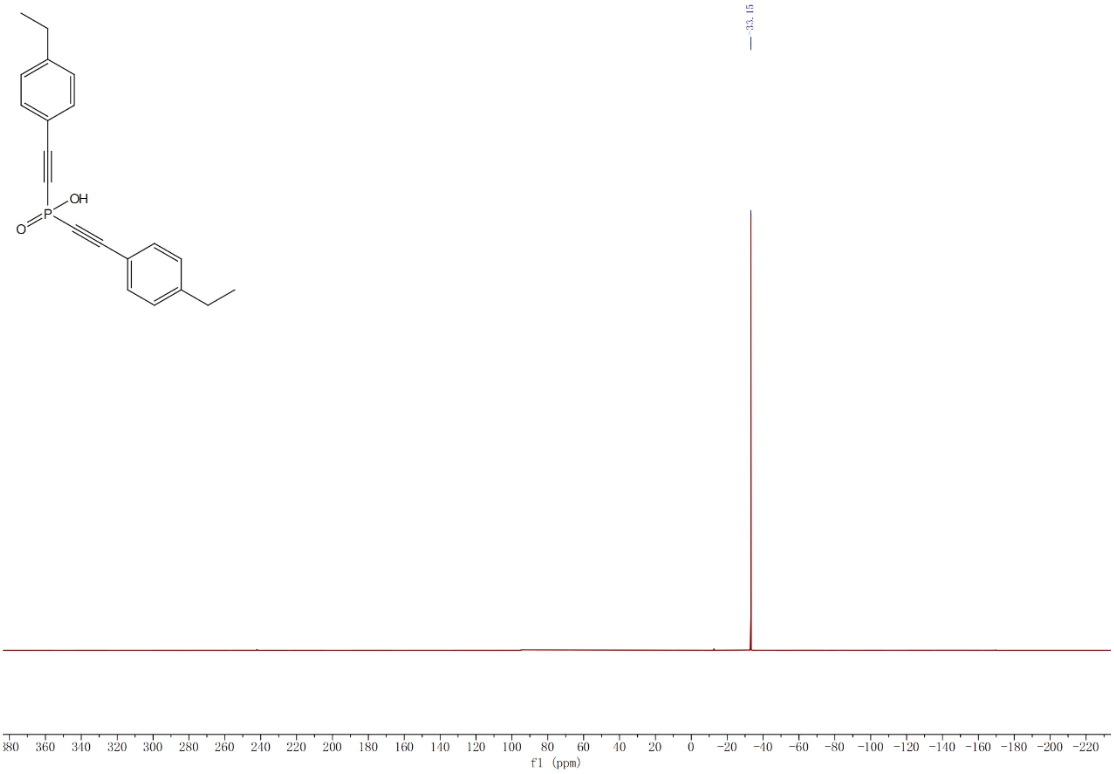

^{13}C NMR (600 MHz, DMSO-D$_6$)

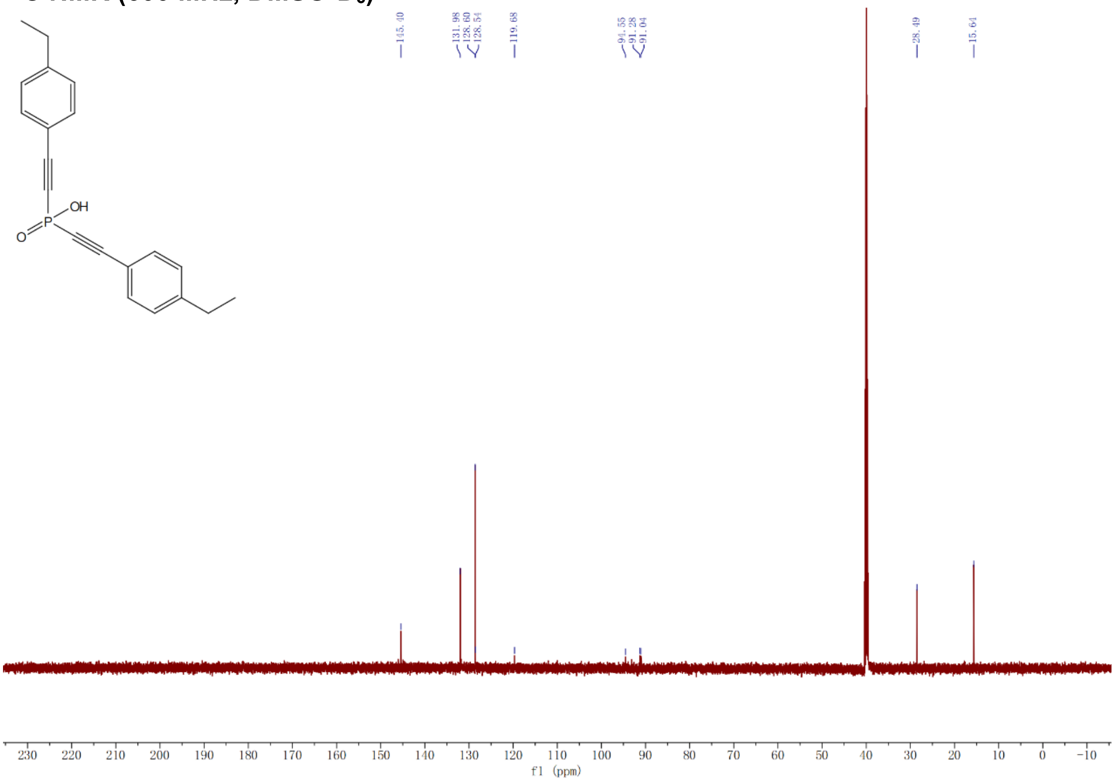

^{1}H NMR (400 MHz, DMSO-D$_6$)

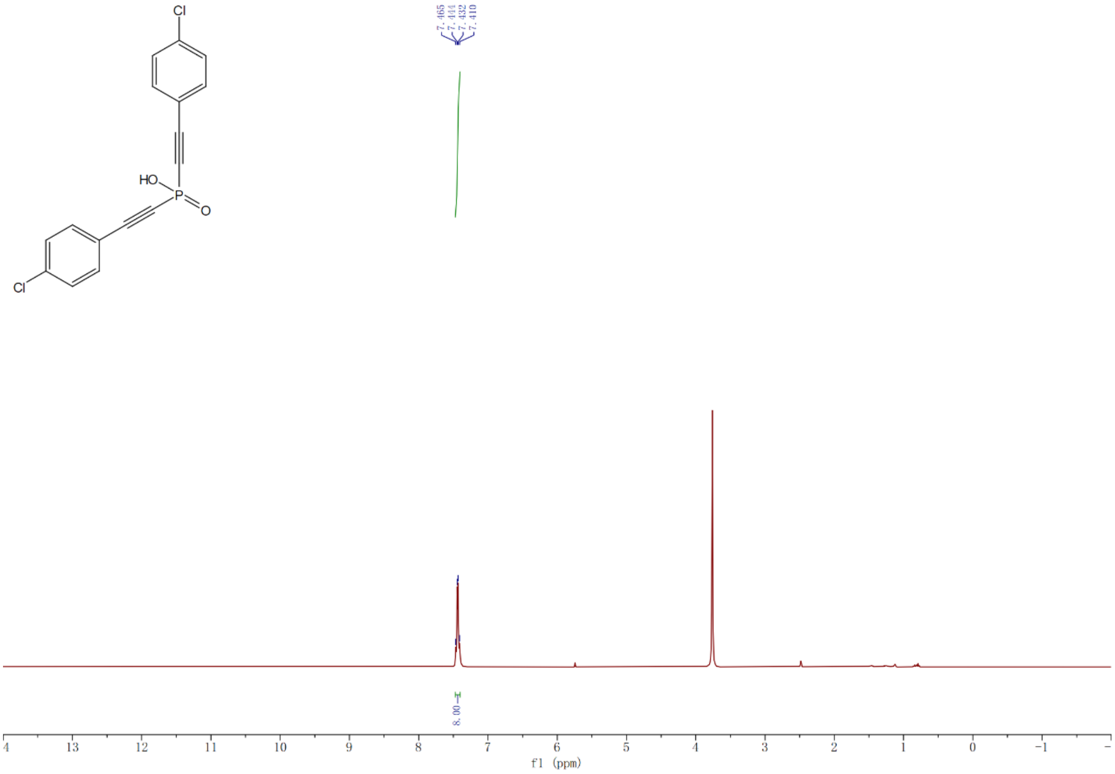

^{31}P NMR (400 MHz, DMSO-D$_6$)

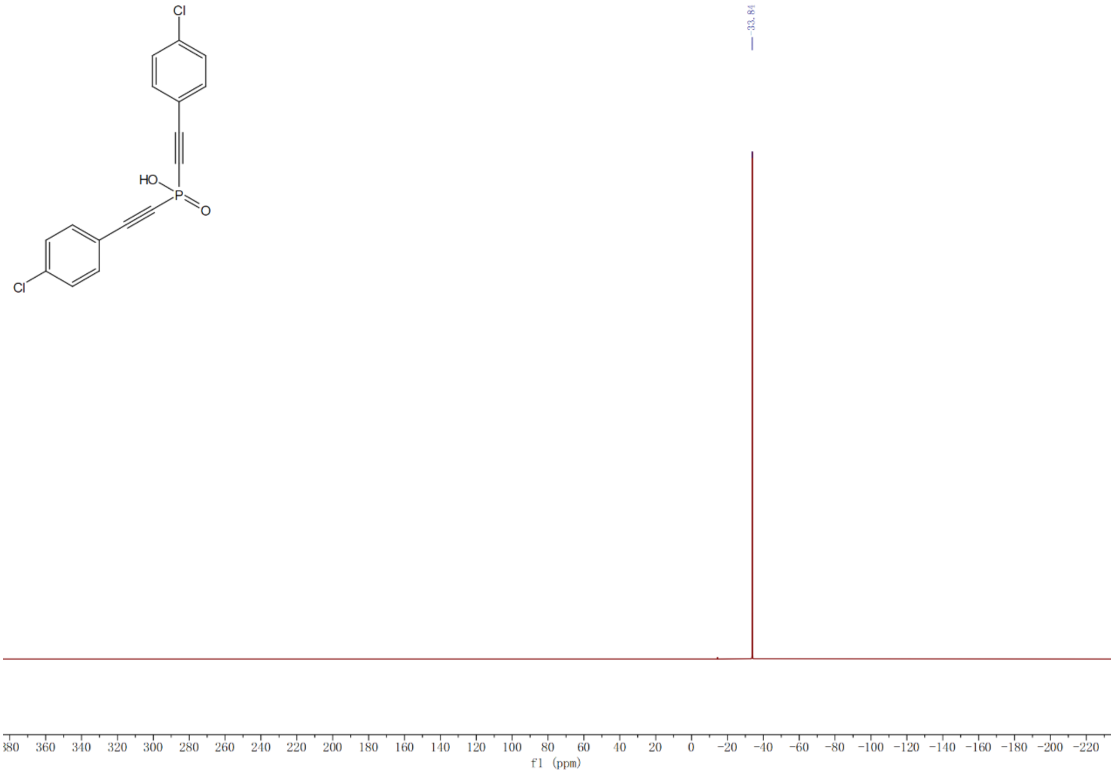

^{13}C NMR (600 MHz, DMSO-D$_6$)

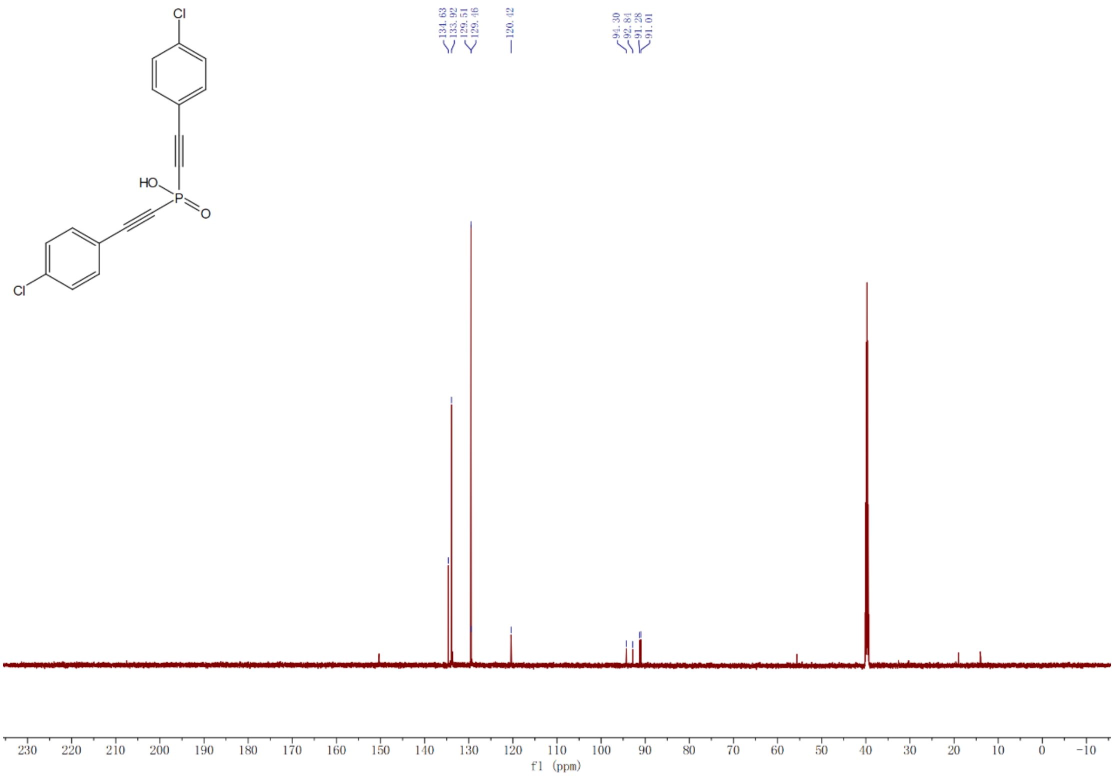

^{1}H NMR (400 MHz, DMSO-D$_6$)

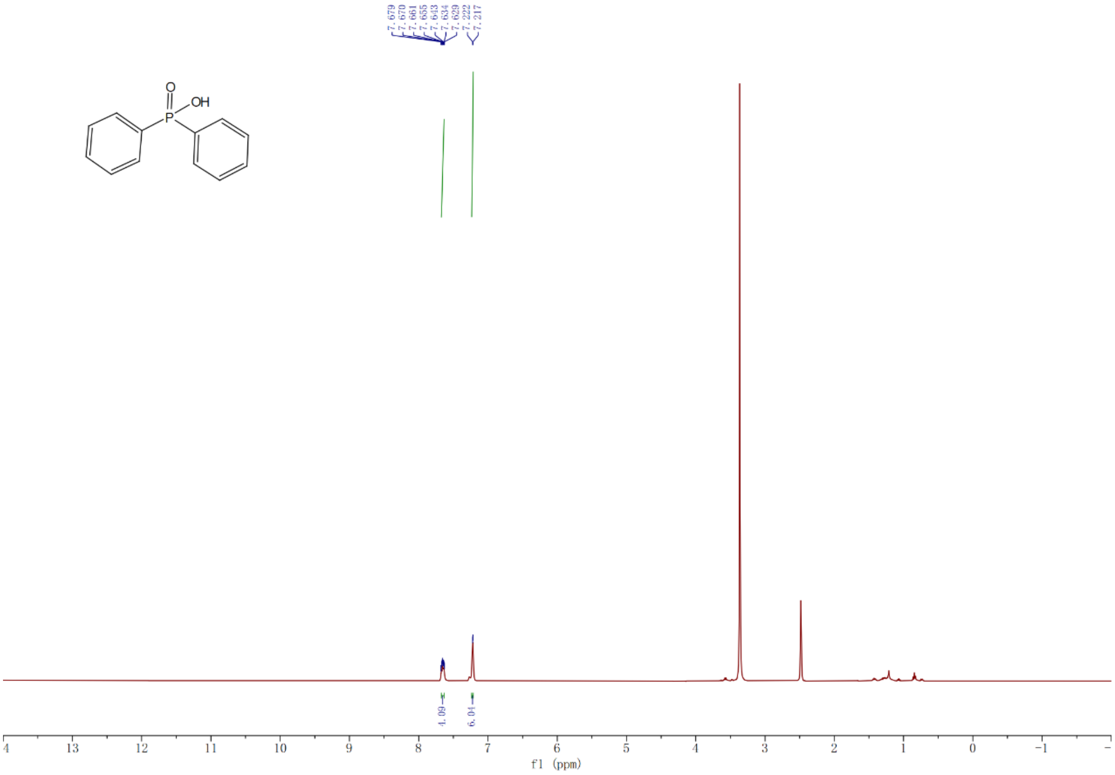

^{31}P NMR (400 MHz, DMSO-D$_6$)

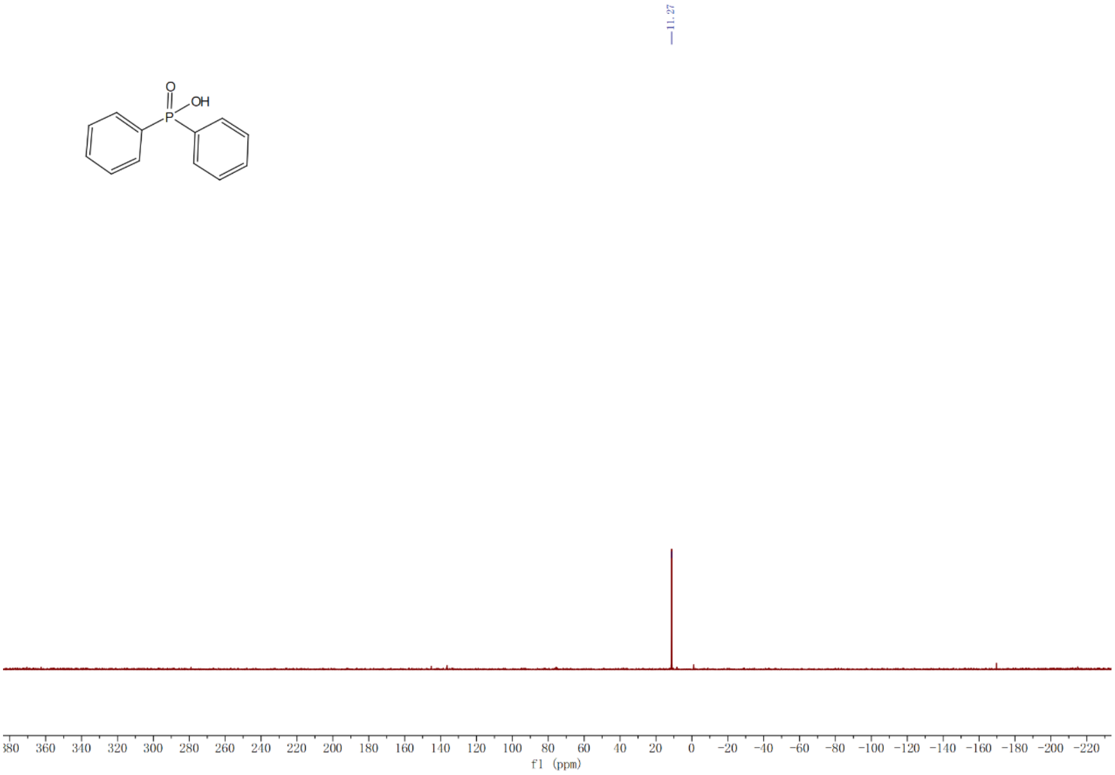

^{13}C NMR (600 MHz, DMSO-D$_6$)

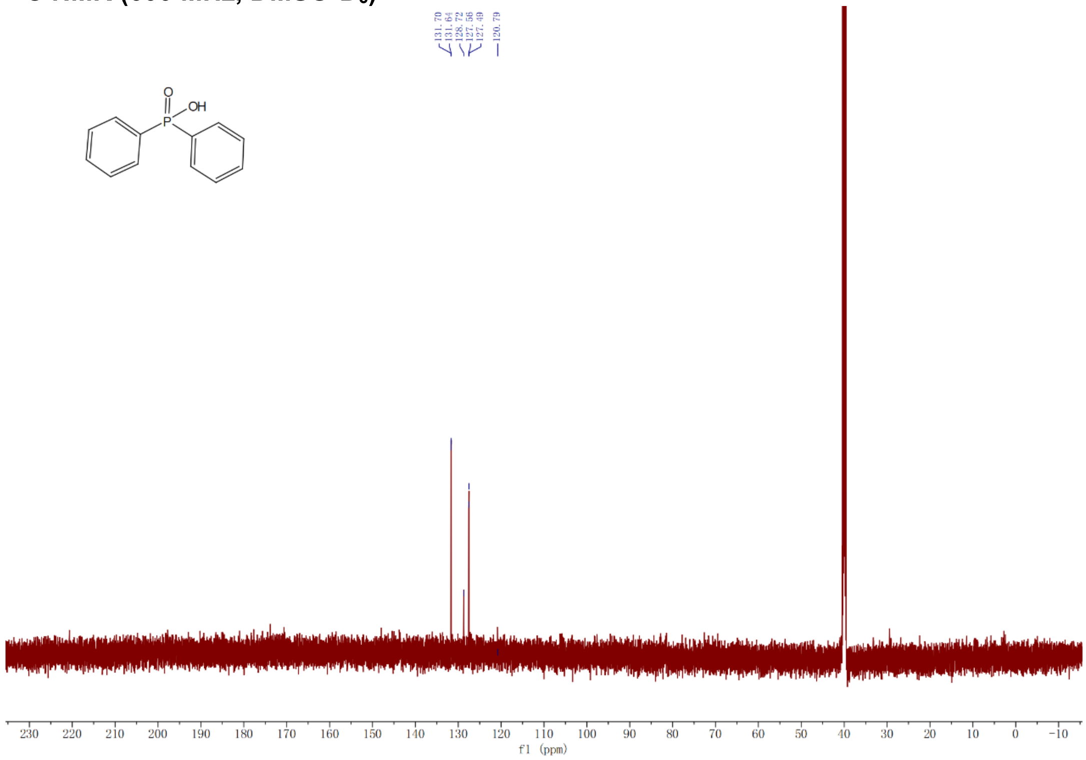

^{1}H NMR (400 MHz, DMSO-D$_6$)

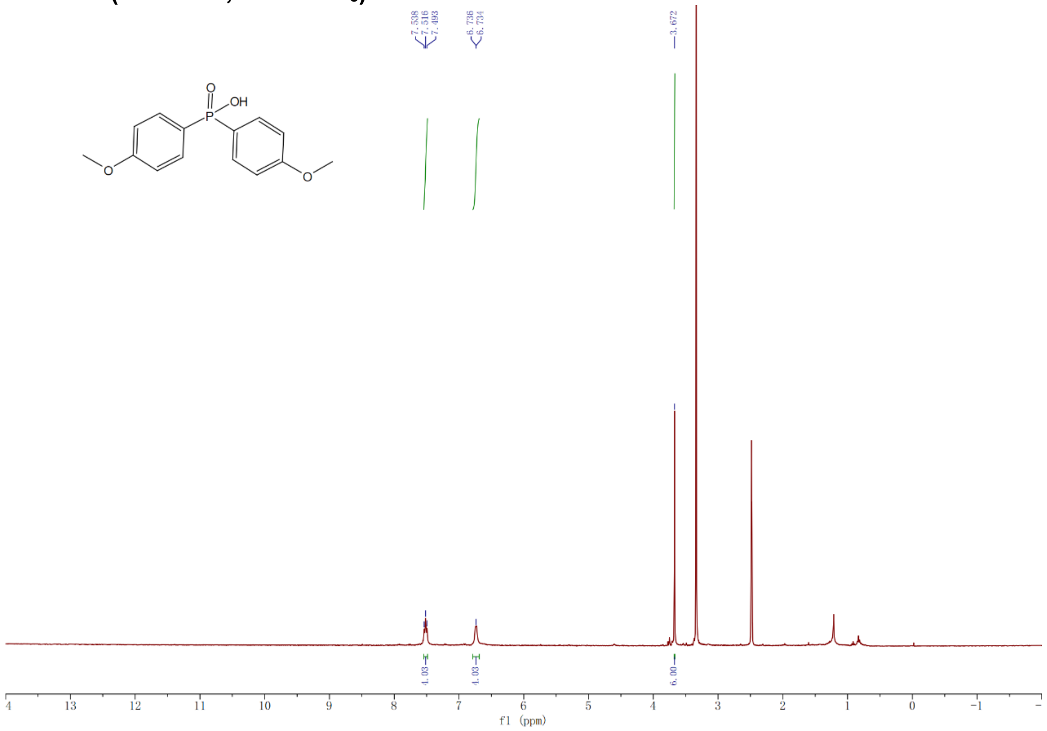

^{31}P NMR (400 MHz, DMSO-D$_6$)

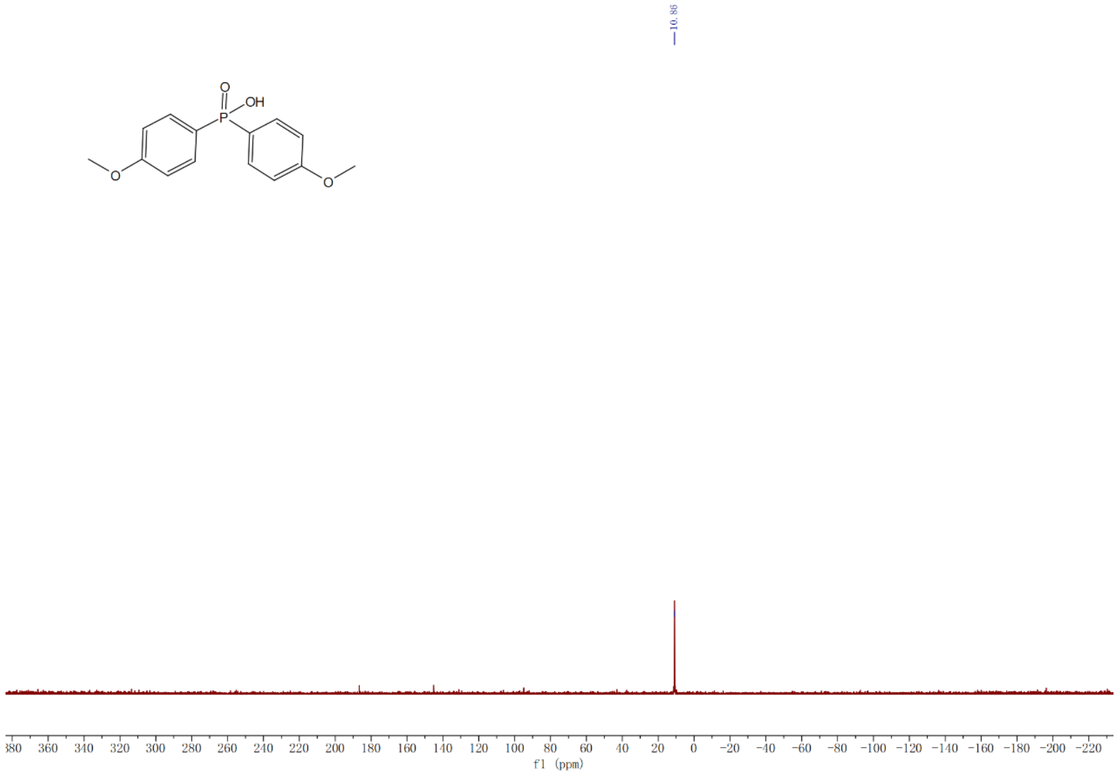

^{13}C NMR (600 MHz, DMSO-D$_6$)

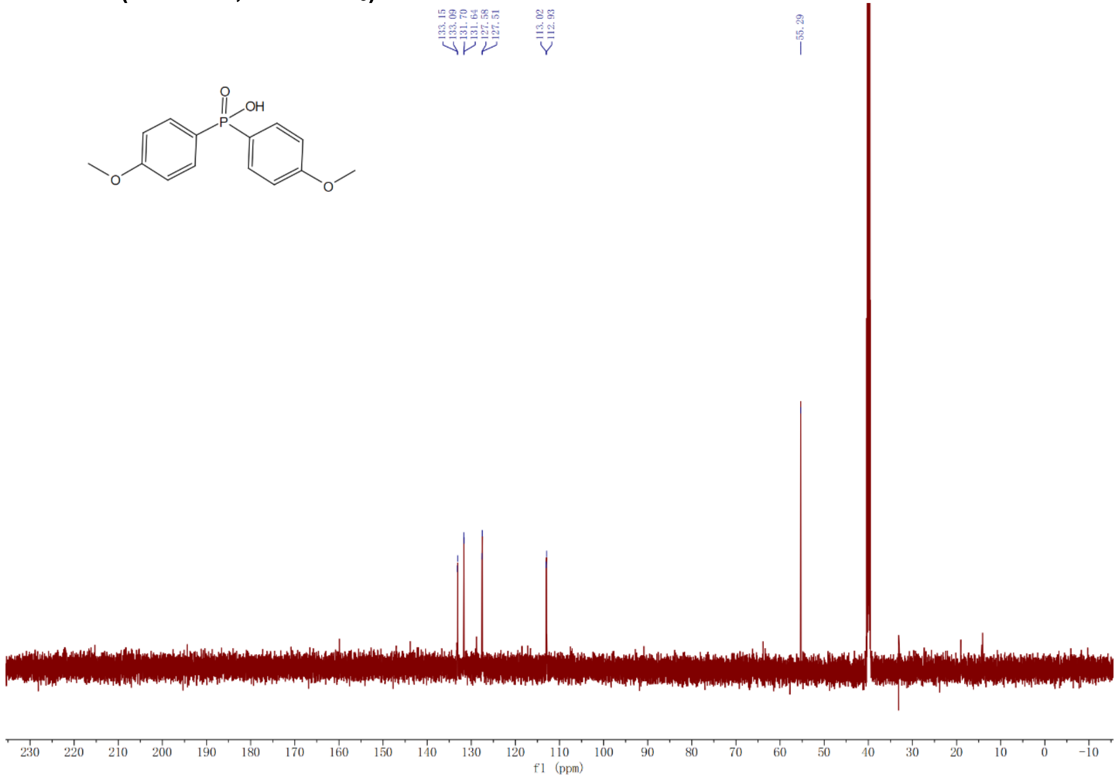

^{1}H NMR (400 MHz, DMSO-D$_6$)

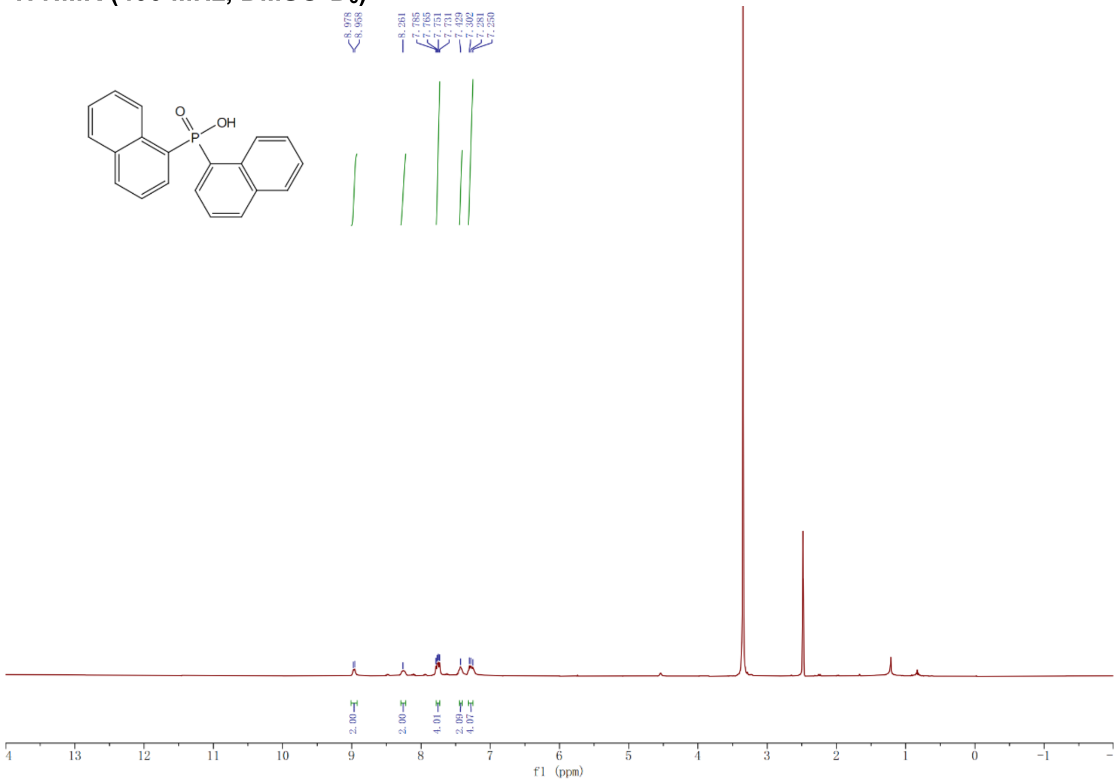

^{31}P NMR (400 MHz, DMSO-D$_6$)

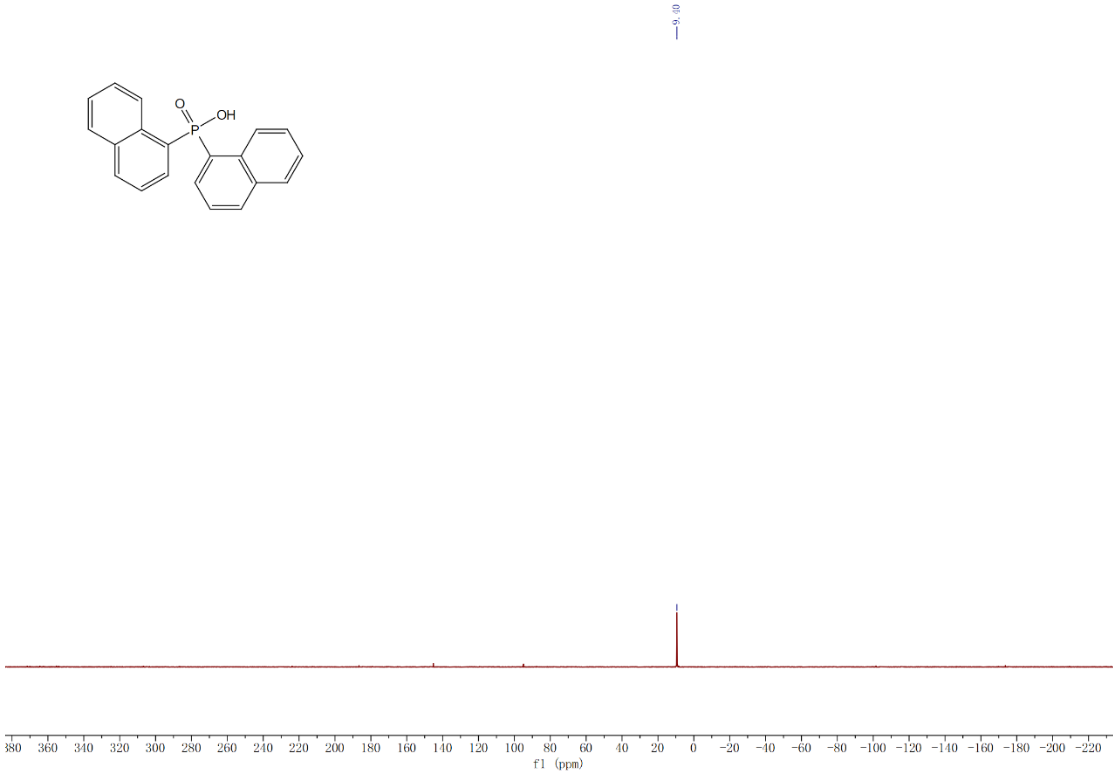

^{13}C NMR (600 MHz, DMSO-D$_6$)

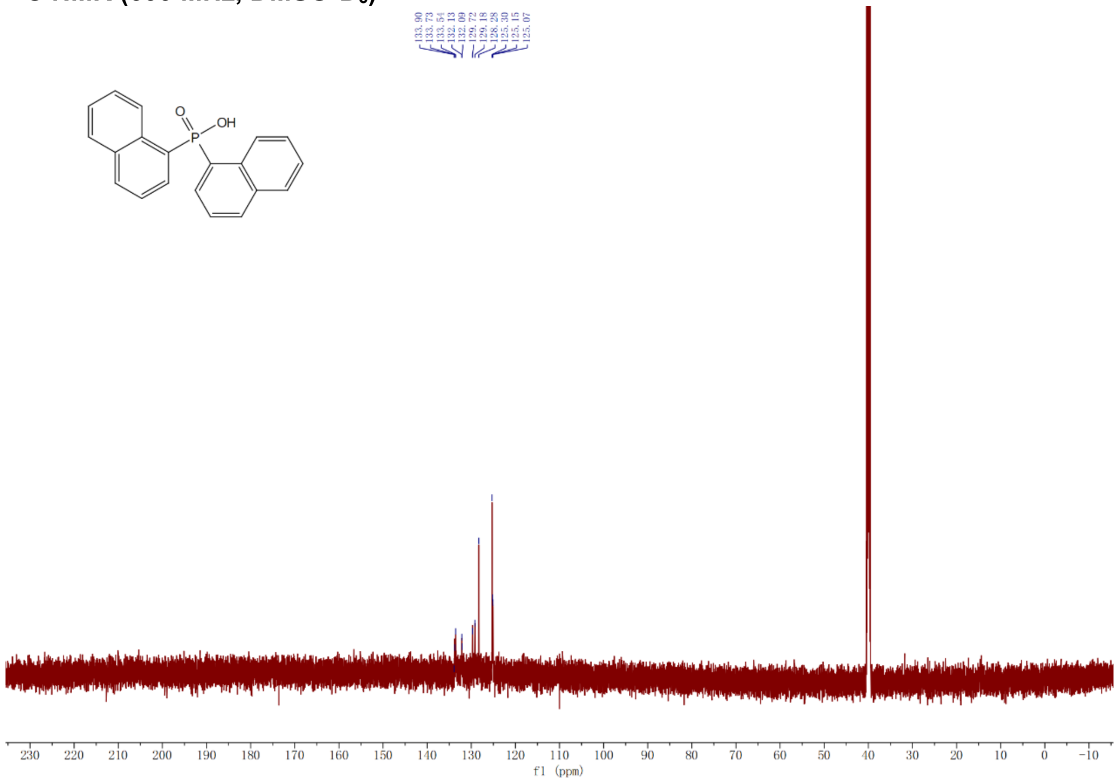

^{1}H NMR (400 MHz, DMSO-D$_6$)

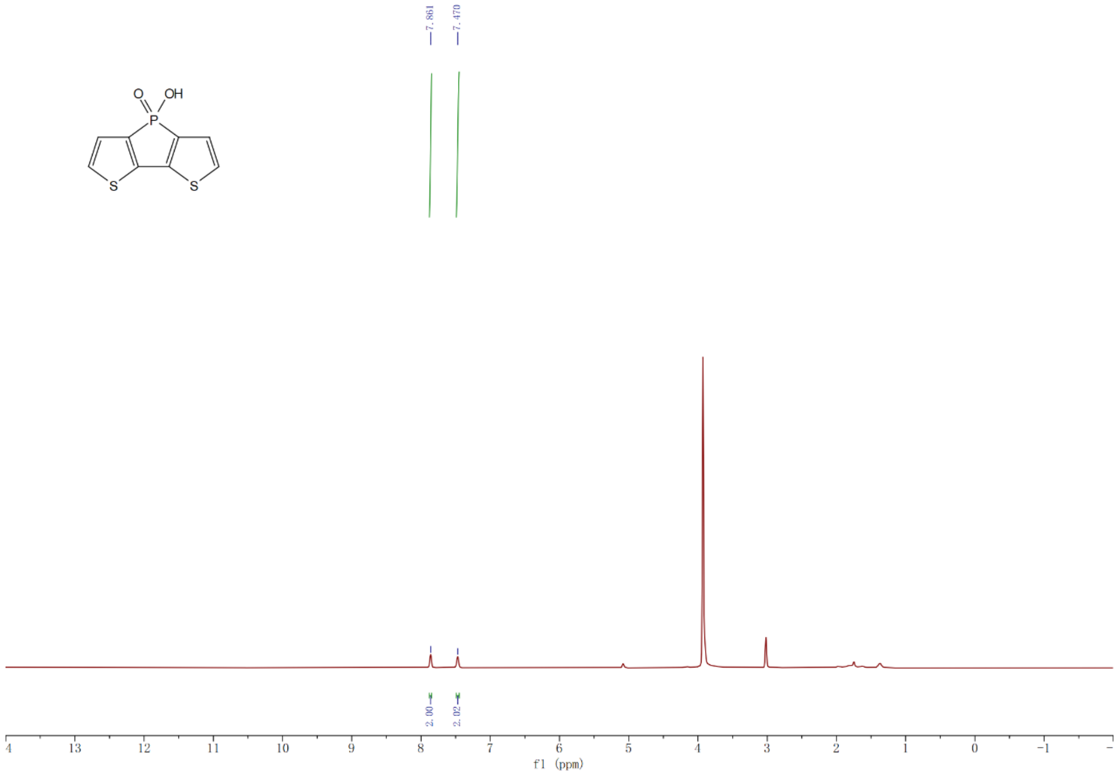

^{31}P NMR (400 MHz, DMSO-D$_6$)

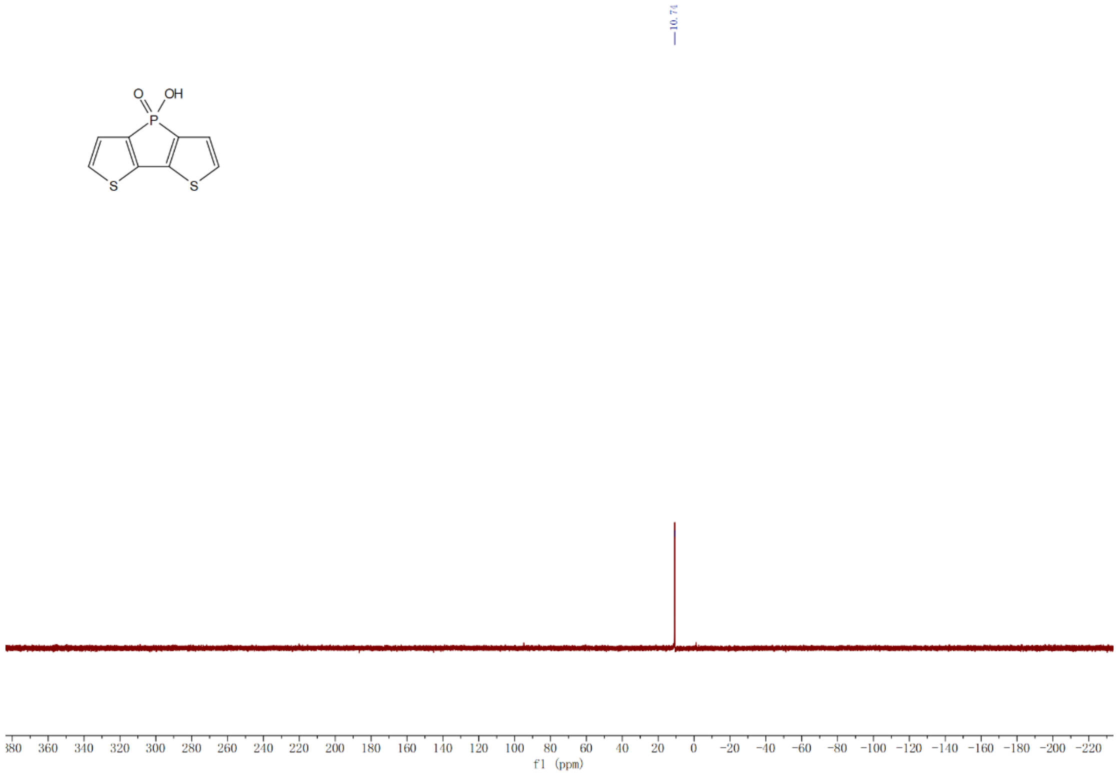

^{13}C NMR (600 MHz, DMSO-D$_6$)

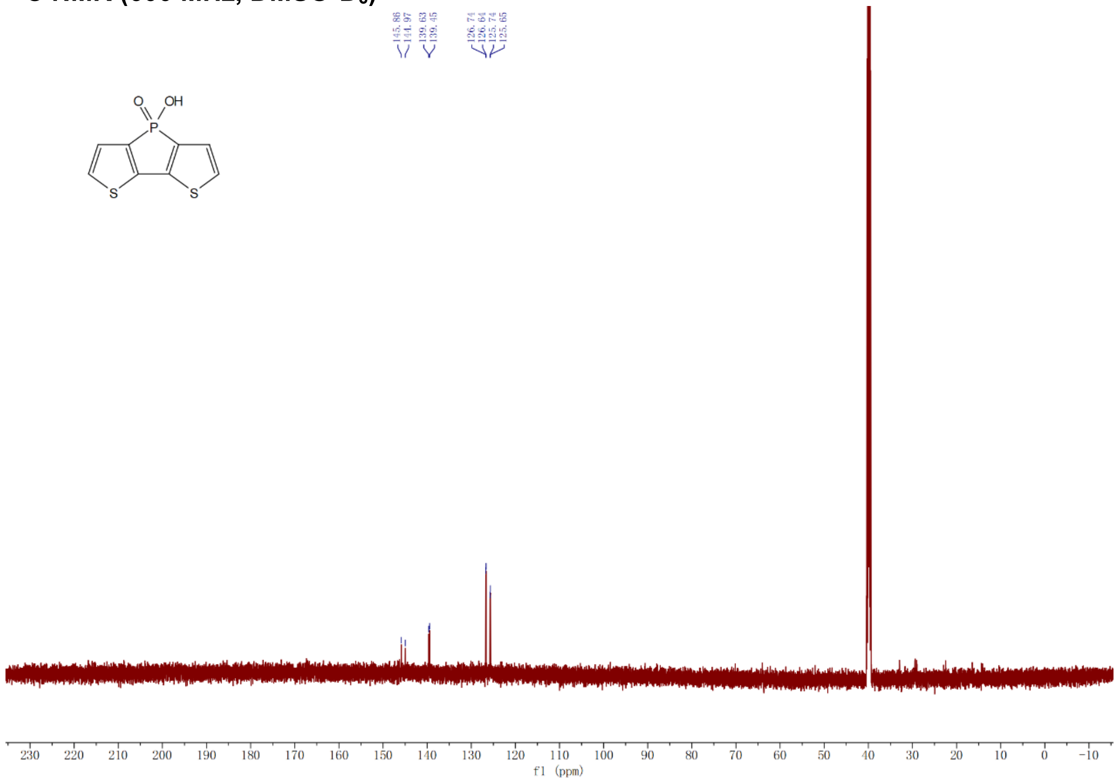

^{1}H NMR (400 MHz, DMSO-D_6)

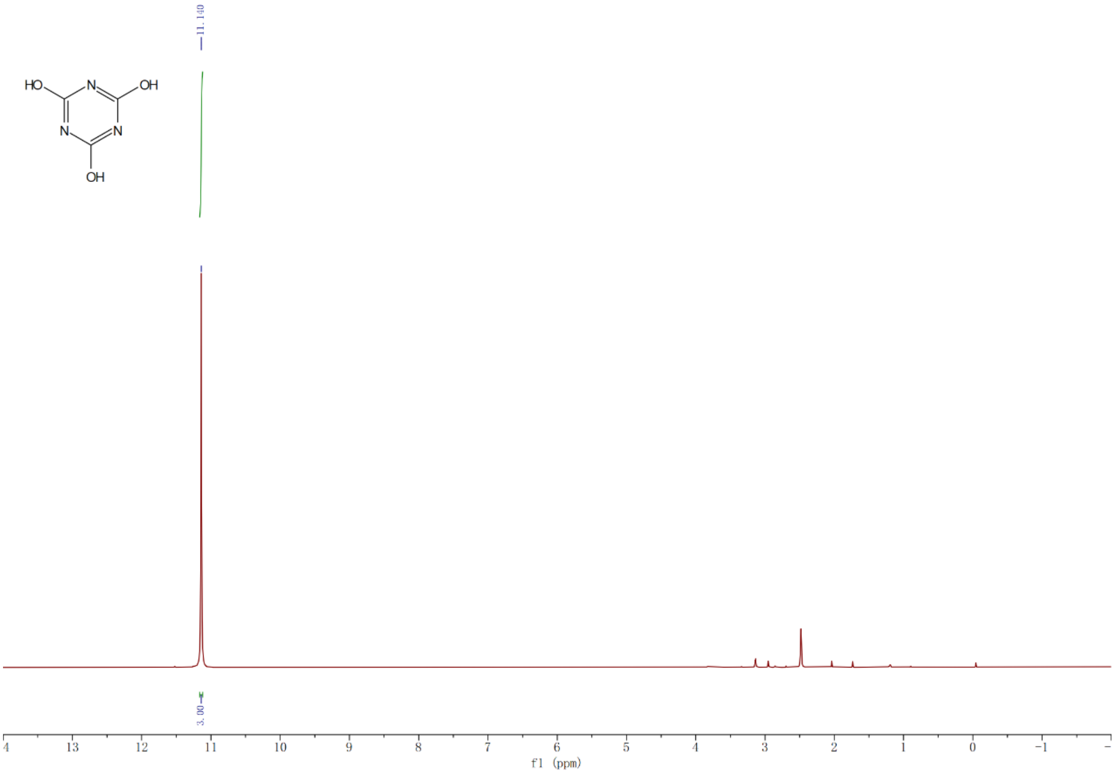

^{13}C NMR (600 MHz, DMSO-D_6)

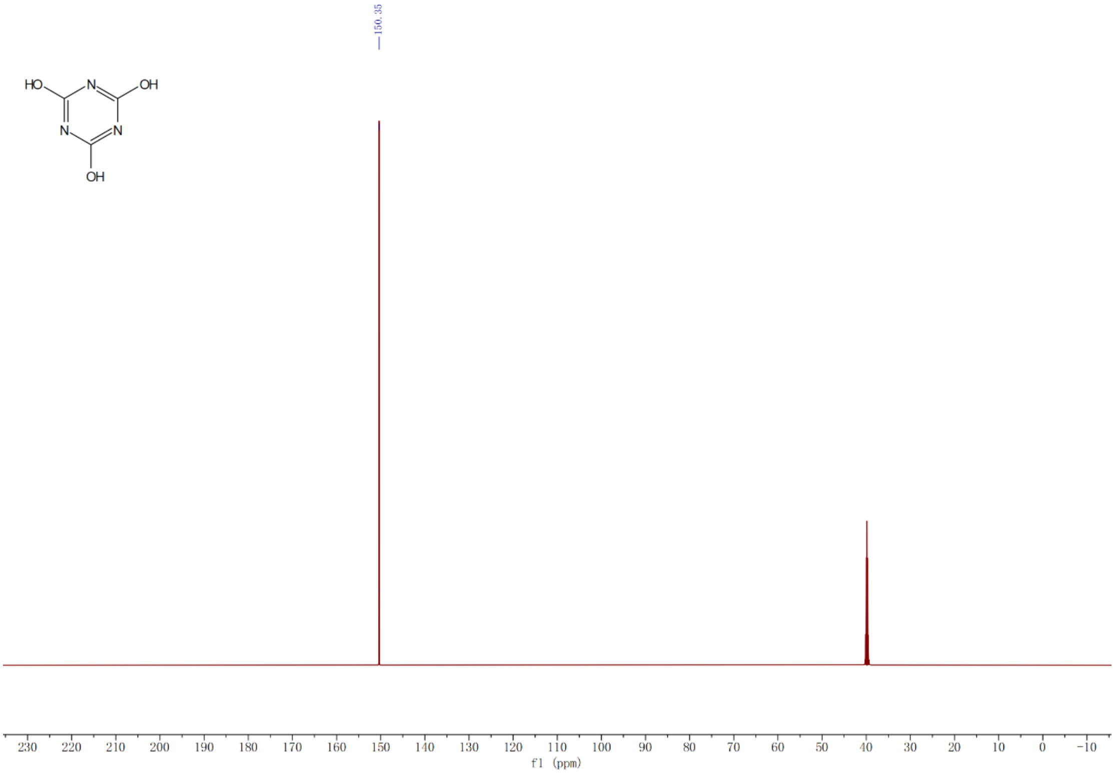

10 References

1 Aldersley, M. F., Joshi, P. C., Ott, E. L., McCallum, S. A., Kirby, A. J. The introduction of P–F bonds using aqueous fluoride ion and a water soluble carbodiimide: a convenient alternative synthesis of phosphorofluoridates and phosphonofluoridates. *Tetrahedron Lett.* **56***,* 5272-5274 (2015).

2 Schultz, C., Vedder, S., Streipert, B., Winter, M., Nowak, S. Quantitative investigation of the decomposition of organic lithium ion battery electrolytes with LC-MS/MS. *RSC Adv.* **7***,* 27853-27862 (2017).

3 Bornemann, D., Brüning, F., Bartalucci, N., Wettstein, L., Pitts, C. R. Examining Trichloroisocyanuric Acid and Oxalyl Chloride in Complementary Approaches to Fluorination of Group 15 Heteroatoms. *Helv. Chim. Acta* **104** (2021).

4 Kaźmierczak, M., Bilska-Markowska, M. Diethylaminosulfur Trifluoride (DAST) Mediated Transformations Leading to Valuable Building Blocks and Bioactive Compounds. *Eur. J. Org. Chem.* **2021***,* 5585-5604 (2021).

5 Li, Q. W., Zhang, X. Y., Lu, L., Wu, Z. Q., Li, J., Li, G. Z., Sun, K., Yang, S. D., Yang, B. TFAA/DMSO-Promoted Fluorination of P(O)−H and P(O)−OH Compounds: Compatible Access to Fluorophosphonates and Phosphonofluoridates. *Adv.Synth.Catal.* **364***,* 938-946 (2022).

6 Miller, L. P., Vogel, J. A., Harel, S., Krussman, J. M., Melvin, P. R. Rapid Generation of P(V)–F Bonds Through the Use of Sulfone Iminium Fluoride Reagents. *Org. Lett.* **25***,* 1834-1838 (2023).

7 Sun, S., Homer, J. A., Smedley, C. J., Cheng, Q.-Q., Sharpless, K. B., Moses, J. E. Phosphorus fluoride exchange: Multidimensional catalytic click chemistry from phosphorus connective hubs. *Chem* **9***,* 2128-2143 (2023).

8 Wang, H., Li, X., Lu, Y., Wang, L., Liu, S., Gao, W., Tang, Y., Wang, L., Niu, L., Chen, J. Et_4NCl-Promoted Electrochemical Atherton-Todd Type Reaction to Construct P(O)–F Bonds. *J. Org. Chem.* **88***,* 7006-7014 (2023).

9 Cao, L., Yu, B., Li, S., Zhang, P., Li, Q., Wang, L. Genetically enabling phosphorus fluoride exchange click chemistry in proteins. *Chem* **9***,* 2128– 2143 (2024).

10 Chappell, W. P., Schur, N., Vogel, J. A., Sammis, G. M., Melvin, P. R., Ball, N. D. Poison to promise: The resurgence of organophosphorus fluoride chemistry. *Chem* **10** (2024).

11 Bisschops, M. A. T., Hateren, S. H. v., Luyben, K. C. A. M., Wielen, L. A. M. v. d. Mass Transfer Performance of Centrifugal Adsorption Technology. *Ind. Eng. Chem. Res.* **39***,* 4376–4382 (2000).

12 S, R. K. Adsorption Behavior of Pb, Cd, Ca, Al, Zn And Co Metal Ions on Cation Exchange Resin Dowex 50 W_{x8}(Nh_4^+Form) From Aqueous Acetone-Ammonium Propionate Media. *Ijsrm* **9***,* 23-27 (2021).

13 Huy, P. H., Motsch, S., Kappler, S. M. Formamides as Lewis Base Catalysts in SNReactions-Efficient Transformation of Alcohols into Chlorides, Amines, and Ethers. *Angew. Chem. Int. Ed.* **55***,* 10145-10149 (2016).

14 Huy, P. H., Mbouhom, C. Formamide catalyzed activation of carboxylic acids – versatile and cost-efficient amidation and esterification. *Chem. Sci.* **10***,* 7399-7406 (2019).

15 Schneider T., S. K., Fidelius J., Weigand J.J. Redox-neutral Conversion of Ubiquitous P^v Sources to a Versatile PO_2^+ Phosphorylation Regent. *Nat. Synth.* **2***,* 972–979 (2023).

16 Dürr-Mayer, T., Qiu, D., Eisenbeis, V. B., Steck, N., Häner, M., Hofer, A., Mayer, A., Siegel, J. S., Baldridge, K. K., Jessen, H. J. The chemistry of branched condensed phosphates. *Nat. Commun.* **12***,* 5368 (2021).

17 Neese, F. Software update: the ORCA program system, version 4.0. *WIREs. Comput. Mol. Sci.* **8** (2017).

18 Becke, A. D. Density-functional thermochemistry. III. The role of exact exchange. *J. Chem. Phys.* **98***,* 5648-5652 (1993).

19 Petersson, G. A., Bennett, A., Tensfeldt, T. G., Al-Laham, M. A., Shirley, W. A., Mantzaris, J. A complete basis set model chemistry. I. The total energies of closed-shell atoms and hydrides of the first-row elements. *J. Chem. Phys.* **89***,* 2193-2218 (1988).

20 Zhao, Y., Schultz, N. E., Truhlar, D. G. Design of Density Functionals by Combining the Method of Constraint Satisfaction with Parametrization for Thermochemistry, Thermochemical Kinetics, and Noncovalent Interactions. *J. Chem. Theory Comput.* **2***,* 364-382 (2006).

21 Garcia-Ratés, M., Neese, F. Efficient implementation of the analytical second derivatives of hartree–fock and hybrid DFT energies within the framework of the conductor-like polarizable continuum model. *J. Comput. Chem.* **40***,* 1816-1828 (2019).

22 Fukui, K. The path of chemical reactions - the IRC approach. *Acc. Chem. Res.* **14***,* 363-368 (2002)
